# NHC–BIAN–Cu(I)-Catalyzed Friedländer-Type Annulation of 2-Amino-3-(per)fluoroacetylpyridines with Alkynes on Water

**DOI:** 10.1021/acs.joc.2c00380

**Published:** 2022-04-08

**Authors:** Magdalena Dolna, Michał Nowacki, Oksana Danylyuk, Artur Brotons-Rufes, Albert Poater, Michał Michalak

**Affiliations:** † Institute of Organic Chemistry, Polish Academy of Sciences, Kasprzaka 44/52, 01-224 Warsaw, Poland; ‡ Institute of Physical Chemistry, Polish Academy of Sciences, Kasprzaka 44/52, 01-224 Warsaw, Poland; § Institut de Química Computacional i Catàlisi and Departament de Química, Universitat de Girona, c/ M. Aurèlia Capmany 69, 17003 Girona, Catalonia, Spain

## Abstract

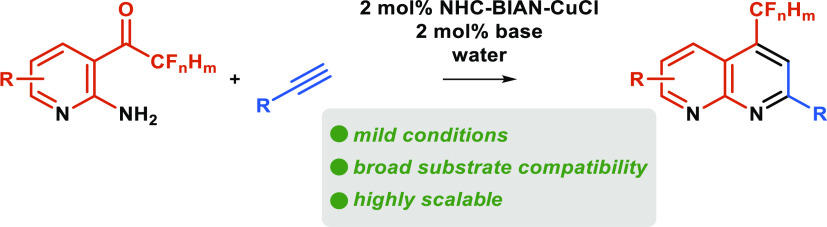

The direct catalytic alkynylation/dehydrative cyclization of 2-amino-3-trifluoroacetyl-pyridines on water was developed for the efficient synthesis of a broad range of fluorinated 1,8-naphthyridines from terminal alkynes. A novel N-heterocyclic carbene (NHC) ligand system that combines a π-extended acenaphthylene backbone with sterically bulky pentiptycene pendant groups was successfully utilized in a copper- or silver-mediated cyclization. Computational analysis of the reaction pathway supports our explanation of the different experimental conversions and yields for the set of copper and silver catalysts. The impact of steric hindrance at the metal center and the flexibility of substituents on the imidazole ring of the NHC on catalytic performance are also discussed.

## Introduction

**Figure 1:**
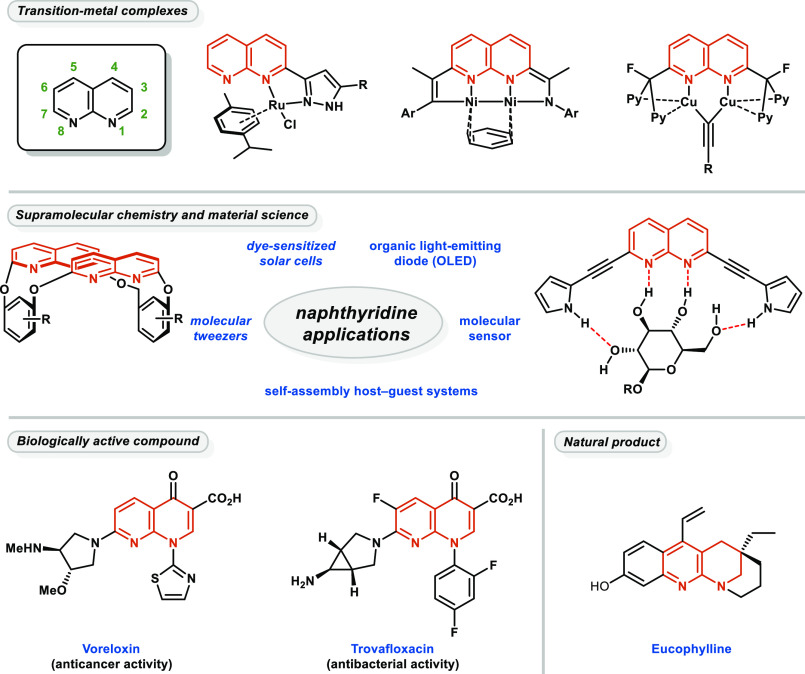
Applications of naphthyridines in catalysis, medicinal chemistry, and materials science.

Naphthyridines are a ubiquitous structural motif in modern medicinal chemistry, as well as in organic synthesis and catalysis. Transition-metal complexes of 1,8-naphthyridine-based ligands have been utilized in many efficient catalytic processes. Among them, rhodium-,^1^ iridium-,^2^ ruthenium-,^3^ copper-,^4^ and nickel-catalyzed^5^ reactions (Figure 1) have gained attention in recent years for enabling useful transformations. These heterocycles have also found widespread application as scaffolds in supramolecular chemistry, for example, as molecular tweezers,^6^ highly selective molecular receptors,^7^ or in self-assembly host–guest systems^8^ (Figure 1). Naphthyridine derivatives are also a central point of interest in modern material science as well as being utilized for the preparation of dye-sensitized solar cells^9^ and OLEDs.^10^ Furthermore, 1,8-naphthyridines can act as powerful hydrogen bond acceptors, which are often incorporated into pharmaceutical active substances, such as voreloxin,^11^ trovafloxacin,^12^ and many other antifungal,^13^ antibacterial,^14^ antiviral,^15^ anticancer,^16^ or antidepressant^17^ compounds. Although fewer in number, some natural products contain this motif; an example of such is eucophylline, which has a partially reduced 1,8-naphthyridine skeleton.^18^

**Scheme 1:**
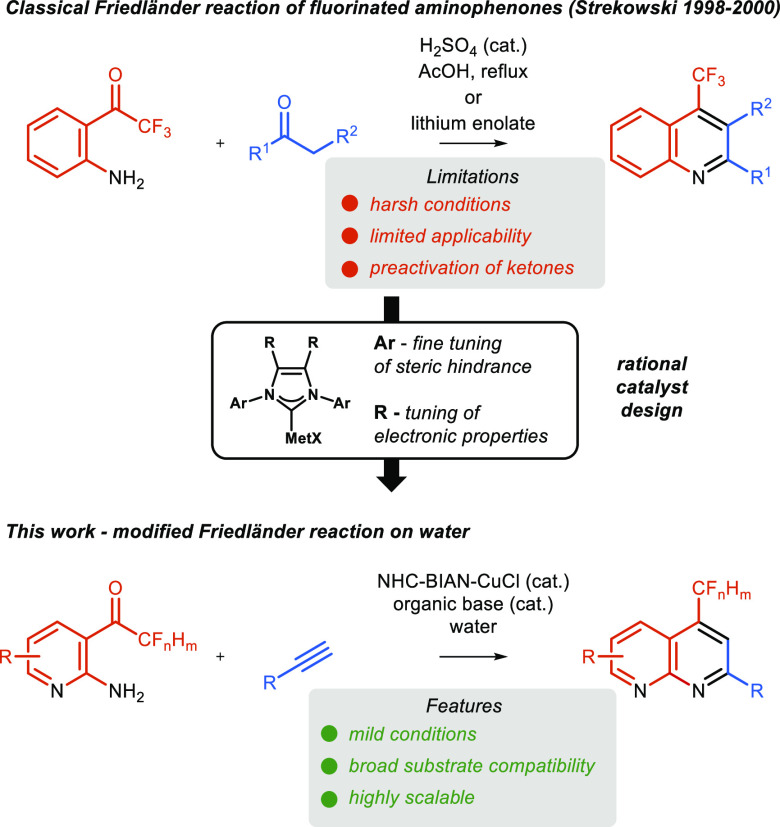
Friedländer Reaction Leading to Fluorinated Naphthyridines

The biological activity of naphthyridines or quinolines can be modified by the incorporation of one or more fluorine atoms into its structure. Synthesis of fluorinated naphthyridines has been demonstrated many times (for selected examples, see Scheme 1).^15,19^ Although many methods have been developed for direct fluorination of azaheterocycles,^20^ direct functionalization of naphthyridine using transition-metal-catalyzed processes remains challenging due to its ability to strongly bind transition metals and suppress their catalytic performance. Therefore, there is a need for a new and practical approach to the synthesis of fluorinated naphthyridines.

The classic Friedländer reaction between an *ortho*-amino aldehyde and an activated methylene compound remains the most obvious choice for the synthesis of many quinolines and naphthyridines.^21^ In contrast, the fluorinated *ortho*-aminophenones present a challenge under classical conditions due to the reactivity of the α-fluoroketone moiety, which can easily undergo hydration or nucleophilic addition when a strong mineral acid or base is used (Scheme 1). It appears that fluorinated *ortho*-aminophenones have been reported only by Strekowski in the late 90s with very limited scope (seven examples) for quinoline synthesis.^22^ Note that fluorinated *ortho*-aminophenones derived from aminopyridine (2-amino-3-trifluoroacetyl-pyridines) have not been utilized to prepare naphthyridines under those harsh conditions.

**Scheme 2:**
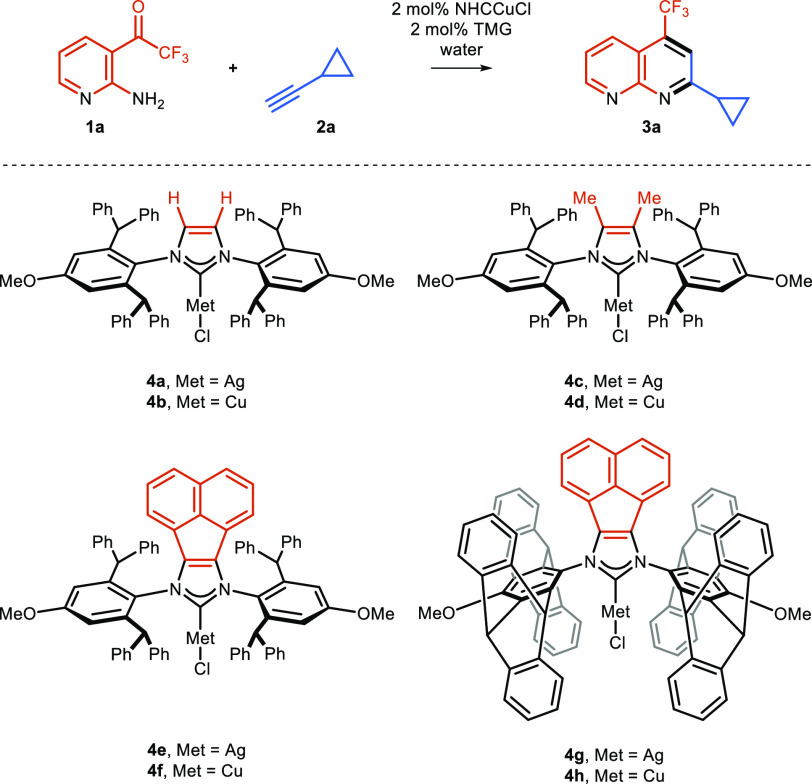
NHC–Cu–Cl and NHC–Ag–Cl Complexes Used for Optimization Studies for the Synthesis of the Naphthyridine Derivative **3a**

We anticipated that a modified Friedländer reaction between terminal alkynes and 2-amino-3-trifluoroacetyl-pyridines could provide a milder synthetic route to useful naphthyridine derivatives. Our previous work,^23^ and that of others,^24^ has demonstrated alkynylation catalyzed by copper and silver NHC complexes on water. We reason that an appropriate combination of steric and electronic tuning of the NHC ligand is critical to its performance in catalysis and may be adapted for this reaction. In particular, we observe a linear correlation between steric hindrance of N-heterocyclic carbene ligands expressed as the percentage of buried volume (%V_bur_)^25^ and the yield resulting from direct catalytic alkynylation of trifluoromethyl ketones leading to trifluoromethyl propargylic alcohols.^23b^ Because increased steric hindrance and donor character of the NHC ligand heavily influence the yield of the alkynylation process, we hypothesize that a more electron-rich NHC ligand (than standard IPr; Scheme 2) equipped with a polyaromatic skeleton should positively impact the alkynylation of pyridine-based *ortho*-aminophenones.

## Results and Discussion

To test our hypothesis, a series of NHC ligands **4a**–**h** were prepared from several sterically hindered aniline derivatives (for details, Scheme 5). For the initial catalytic performance test, unsubstituted 2-amino-3-trifluoroacetyl-pyridines (**1a**) and cyclopropyl acetylene **2a** were selected (Scheme 2). The optimization studies were conducted at an elevated temperature on water with 2 mol % of catalyst and an equimolar amount of TMG (1,1,3,3-tetramethylguanidine). Generally, silver and copper complexes **4a**–**d** did not perform well under these conditions (Table 1, entries 1–4) providing naphthyridine **3a** in marginal yield. Extension of the NHC ligand backbone by incorporating a rigid acenaphthylene subunit is known to increase σ-donation^26^ (this is an NHC–BIAN-type in reference to its bis(iminoacenaphthene) precursor). The resulting formation of the more nucleophilic metal acetylide had a beneficial effect on reactivity. In the series of complexes with NHC ligands bearing a π-extended backbone (**4e**–**h**, Table 1, entries 5–8), copper complexes **4f** and **4h** performed better than silver ones **4e** and **4g**. Finally, complex **4h** bearing pentiptycene as the N-wingtip substituent was observed to be the superior catalyst, providing naphthyridine **3a** in a 64% isolated yield. Further optimization proved that decreasing the amount of alkyne **2a** to 1.2 equiv afforded product **3a** with a comparable yield of 50%, while increasing up to 2.2 equiv returned a lower yield of 21% (Table 1, entries 12 and 13; for details on the optimization, see SI).

**Table 1:** Results of Optimization Studies for the Synthesis of the Naphthyridine Derivative **3a**

entry	**2a** (equiv)	**NHCCuCl**	time (h)	temp. (°C)	conv. (%)^a^	yield (%)^b^
1	1.8	**4a**	19	120	95	4
2	1.8	**4b**	19	120	33	4
3	1.8	**4c**	19	120	79	13
4	1.8	**4d**	19	120	19	1
5	1.8	**4e**	19	120	88	10
6	1.8	**4f**	19	120	63	**55**
7	1.8	**4g**	19	120	89	2
8	1.8	**4h**	19	120	88	**64**
9	1.8	**4h**	1	120	18	<1
10	1.8	**4h**	19	100	10	<1
11	1.8	**4h**	19	80	5	1
12	1.2	**4h**	19	120	80	50
13	2.2	**4h**	19	120	87	21

a Conversion based on GC with durene as the internal standard.

b Yield based on GC from the calibration curve.

To test our hypothesis, a series of NHC ligands **4a**–**h** were prepared from several sterically hindered aniline derivatives (for details, see Scheme 5). For the initial catalytic performance test, unsubstituted 2-amino-3-trifluoroacetyl-pyridines (**1a**) and cyclopropyl acetylene **2a** were selected (Scheme 2). The optimization studies were conducted at an elevated temperature on water with 2 mol % of catalyst and an equimolar amount of TMG (1,1,3,3-tetramethylguanidine). Generally, silver and copper complexes **4a**–**d** did not perform well under these conditions (Table 1, entries 1–4), providing naphthyridine **3a** in marginal yield. Extension of the NHC ligand backbone by incorporating a rigid acenaphthylene subunit is known to increase σ-donation^26^ (this is an NHC–BIAN-type in reference to its bis(iminoacenaphthene) precursor). The resulting formation of the more nucleophilic metal acetylide had a beneficial effect on reactivity. In the series of complexes with NHC ligands bearing a π-extended backbone (**4e**–**h**, Table 1, entries 5–8), copper complexes **4f** and **4h** performed better than silver ones **4e** and **4g**. Finally, complex **4h** bearing pentiptycene as the N-wingtip substituent was observed to be the superior catalyst, providing naphthyridine **3a** in a 64% isolated yield. Further optimization proved that decreasing the amount of alkyne **2a** to 1.2 equiv afforded product **3a** with a comparable yield of 50%, while increasing up to 2.2 equiv returned a lower yield of 21% (Table 1, entries 12 and 13; for details on the optimization, see SI, Table S1).

**Scheme 3:**
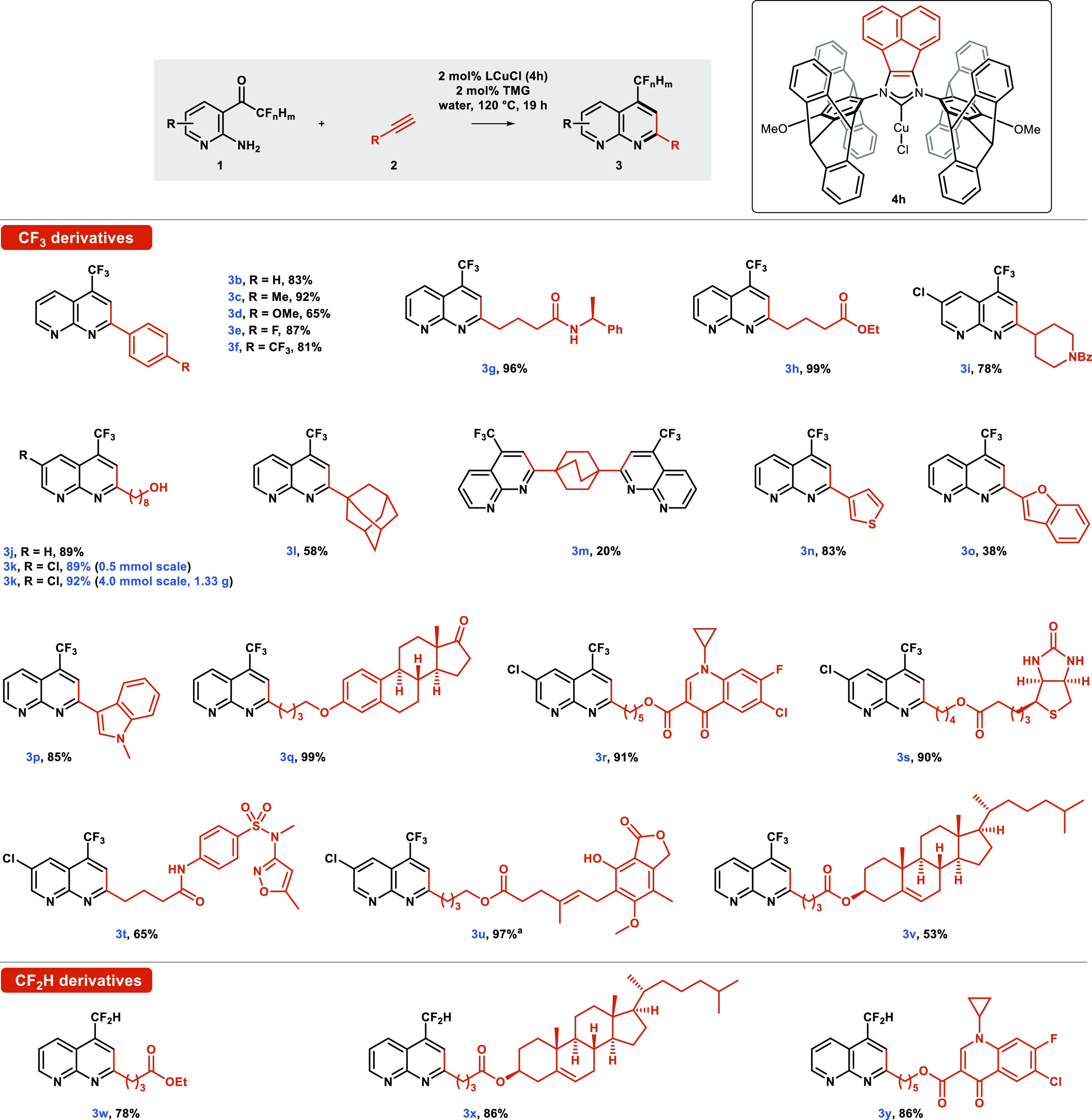
Scope of NHC-Catalyzed Naphthyridine Synthesis TBS-protected phenol was used at the alkyne substrate. Deprotection of phenol moiety occurred under the reaction conditions.

With the optimal catalyst and reaction conditions established, the scope of the NHC–BIAN–CuCl-catalyzed naphthyridine synthesis was investigated using a variety of terminal alkynes (Scheme 3). Initially, a series of phenylacetylene derivatives possessing electron-donating and electron-withdrawing groups in the *para* position to the triple bond were investigated. The desired heterocycles **3b**–**f** were obtained with excellent yields of 65–92%. Rather surprisingly, complex **4h** catalyzed the reaction with the substrate 4-CF_3_ phenylacetylene to give **3f**. This stands in contrast to the findings in our previous work on the alkynylation of nitrones in which phenylacetylene bearing electron-withdrawing groups (NO_2_, TsO) appeared to be unreactive.^23a^

**Scheme 4:**
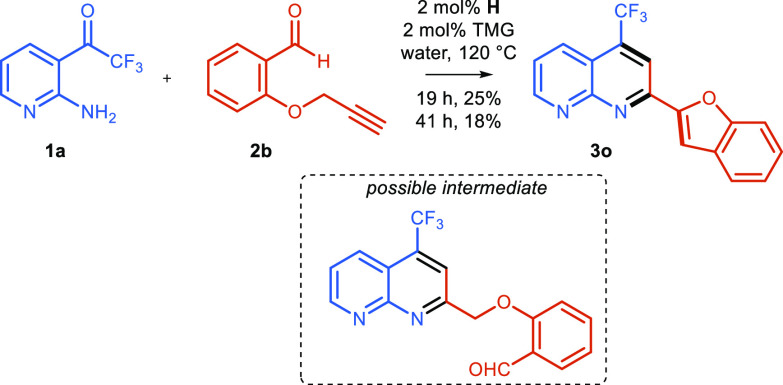
Tandem Direct Catalytic Alkynylation/Double Dehydrative Cyclization

Alkyl-substituted alkynes bearing functional groups such as an amide (**3g**), an ester (**3h**), a piperidine (**3i**), or an unprotected hydroxyl group (**3j**,**k**) were also tolerated. Further investigation demonstrated that a common bioisostere of disubstituted benzene^27^ could be directly attached to the naphthyridine core. A sterically encumbered adamantane derivative (**3l**) was obtained in a high yield of 58%, and a double Friedländer reaction afforded a dinaphthyridine derivative with two heterocyclic subunits connected via bicyclo[2.2.2]octane linker (**3m**). Finally, the synthetic utility of the method was demonstrated by successfully utilizing several structurally complex alkyne substrates derived from biologically active compounds as well as natural products, further supporting the excellent functional group tolerance of this methodology. Thus, androstane, cholesterol, biotin, mycophenolic acid, quinolonic acid, and sulfamethoxazole-derived alkynes afforded products **3q**–**v** in excellent yields in the range of 65–99%. It should be noted that no byproducts have been detected despite the presence of functional groups that are potentially reactive toward metal acetylides. These include 5-membered lactones and cyclopropyl rings that can undergo ring opening, and enones or ketone conjugate addition. Several of the substituents used are common ligands able to coordinate with copper and potentially suppress its catalytic activity such as the heterocycles tetrahydrofuran and oxazole or amide, urea, and hydroxyl groups. However, these did not appear to suppress the naphthyridine formation. Next, we examined whether difluoromethyl ketone derivatives could be engaged in the Friedländer reaction. These are potentially more challenging due to their slightly acidic character. The incorporation of the CF_2_H group into heterocycles has gained a lot of attention in medicinal chemistry^19a,28^ due to their ability to act as lipophilic hydrogen bond donors, modifying permeability, binding affinity, and bioavailability.^29^ To our delight, difluoromethyl naphthyridines **3w**–**y** were formed in high yields of around 80%. The method also demonstrated that ethynyl-substituted heterocycles could provide the respective naphthyridines. Usually, coupling two heterocyclic components is accomplished via a palladium-catalyzed protocol; however, the required 2-substituted azaheterocycles (e.g., 2-bromopyridine or its analogues) are challenging substrates.^30^ The protocol developed here offers an alternative. In our case, thiophene **3n** and indole **3p** derivatives were obtained in high yields, whereas the benzophenone-derived alkyne afforded product **3o** in lower yields of 38%. To improve the yield of the benzofuran-substituted naphthyridine **3o**, we investigated whether this could be prepared via a novel tandem catalytic alkynylation/double dehydrative cyclization from the salicylic aldehyde derivative **2b** (Scheme 4). Indeed, naphthyridine **3o** was formed by this approach but with a lower yield of 25%.

Finally, the robustness of the naphthyridines synthesis was demonstrated by the gram-scale synthesis of the octanol-substituted naphthyridine **3k**. It should be noted that the 8-fold scale-up did not impact the high yield (89% on a 0.5 mmol scale vs 92% on a 4.0 mmol scale) and afforded 1.3 g of **3k** in a single batch.

### Synthesis of NHC–BIAN-Type Complexes and Mechanistic Considerations

The key to the successful implementation of NHC metal catalysts in the development of a protocol for the synthesis of fluorinated naphthyridines in water was the rational design of the NHC ligand’s structure. We initially assumed that ligands exhibiting strong σ-donor properties and possessing sterically hindered N-wingtip substituents with additional electron-donating functionality such as methoxy groups should be the best combination. We were particularly interested in how the extension of the NHC backbone would influence the ligand’s electronic properties and hence the catalytic activity of its complex. An excellent example of how structural modification of an NHC ligand can significantly influence the catalytic activity of metal complexes is the IPr* ligand. This sterically encumbered NHC ligand, developed by Nolan and co-workers,^31^ had a profound effect on the development of processes catalyzed by Pd, Rh, Ir, Cu, and Au complexes, demonstrating remarkable catalytic performance in comparison to the commonly used IPr ligand.^32^

**Scheme 5:**
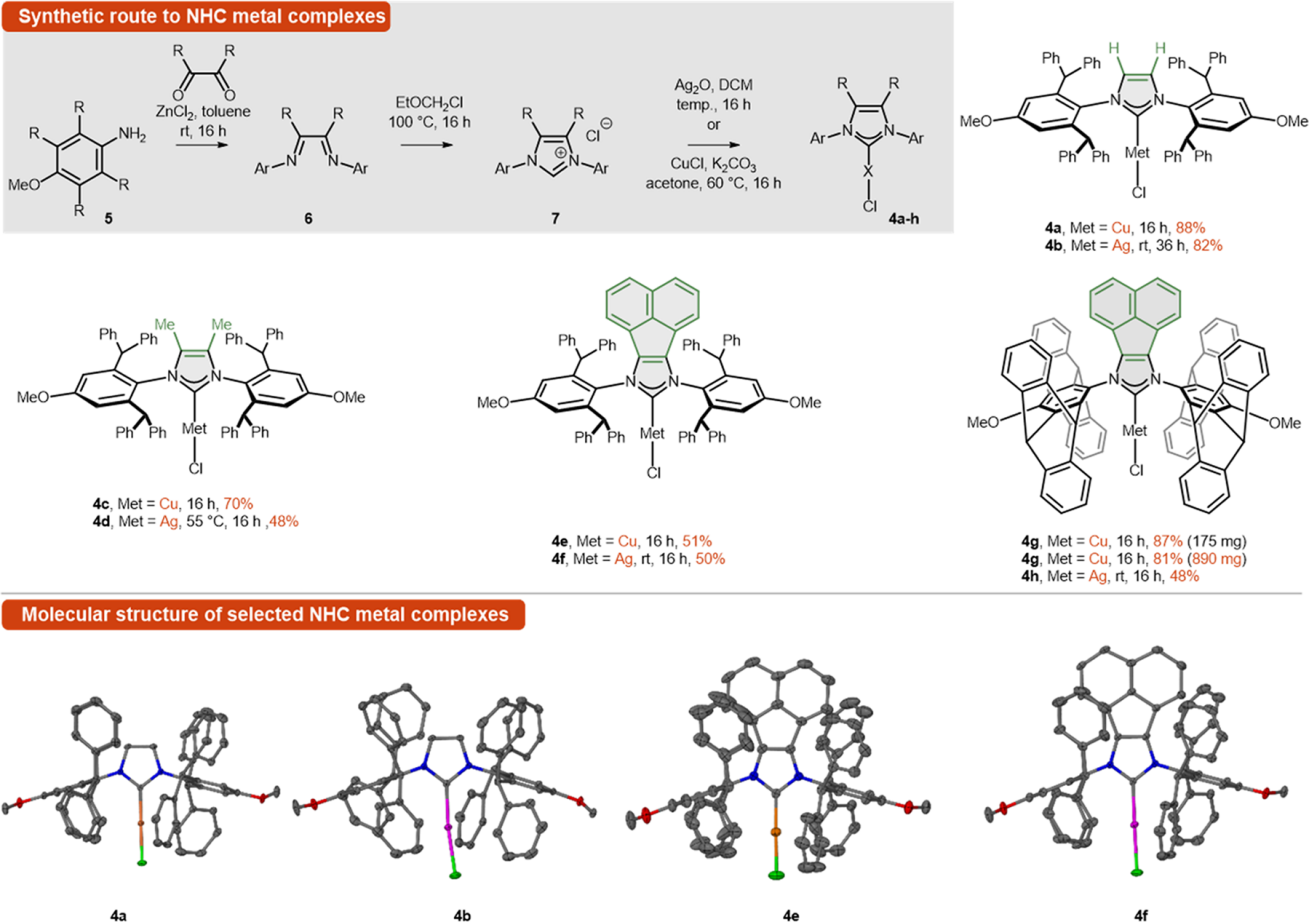
Synthesis of NHC–Cu–Cl and NHC–Ag–Cl Complexes and Selected Molecular Structure for Complexes **4a**, **4b**, **4e**, and **4f** Thermal ellipsoids represent 50% probability; hydrogen atoms on carbon are omitted for clarity.

A set of carbene precursors (**7**) were synthesized via bisimine (**6**) formation and subsequent cyclization with chloromethyl ethyl ether (EOMCl; Scheme 5). It should be noted that the aniline (**5**)- and pentiptycene^33^-derived starting materials are easily obtained on the multigram scale following literature procedures. The respective imidazolium salts (**7**) were also prepared in large quantities and isolated by precipitation from the reaction mixture (for details, see the SI). Copper and silver complexes of the NHC precursors (**7**) were prepared using Nolan’s^34^ and Lin’s^35^ well-established procedures. These afforded the pure complexes **4a**–**4h** without the need for chromatographic purification at any step (Scheme 5). Complex **4h**, which appeared to be optimal for naphthyridine synthesis (*vide infra*), was successfully prepared on a large scale (890 mg) without any reduction in yield (81%), underlining the scalability of the developed method. The structures of complexes **4a**, **4b**, **4e**, and **4f** were unequivocally confirmed by X-ray analysis. Unfortunately, all attempts to get monocrystals of the pentiptycene derivatives **4g** and **4h** failed due to the poor solubility of the complex in organic solvents.

**Scheme 6:**
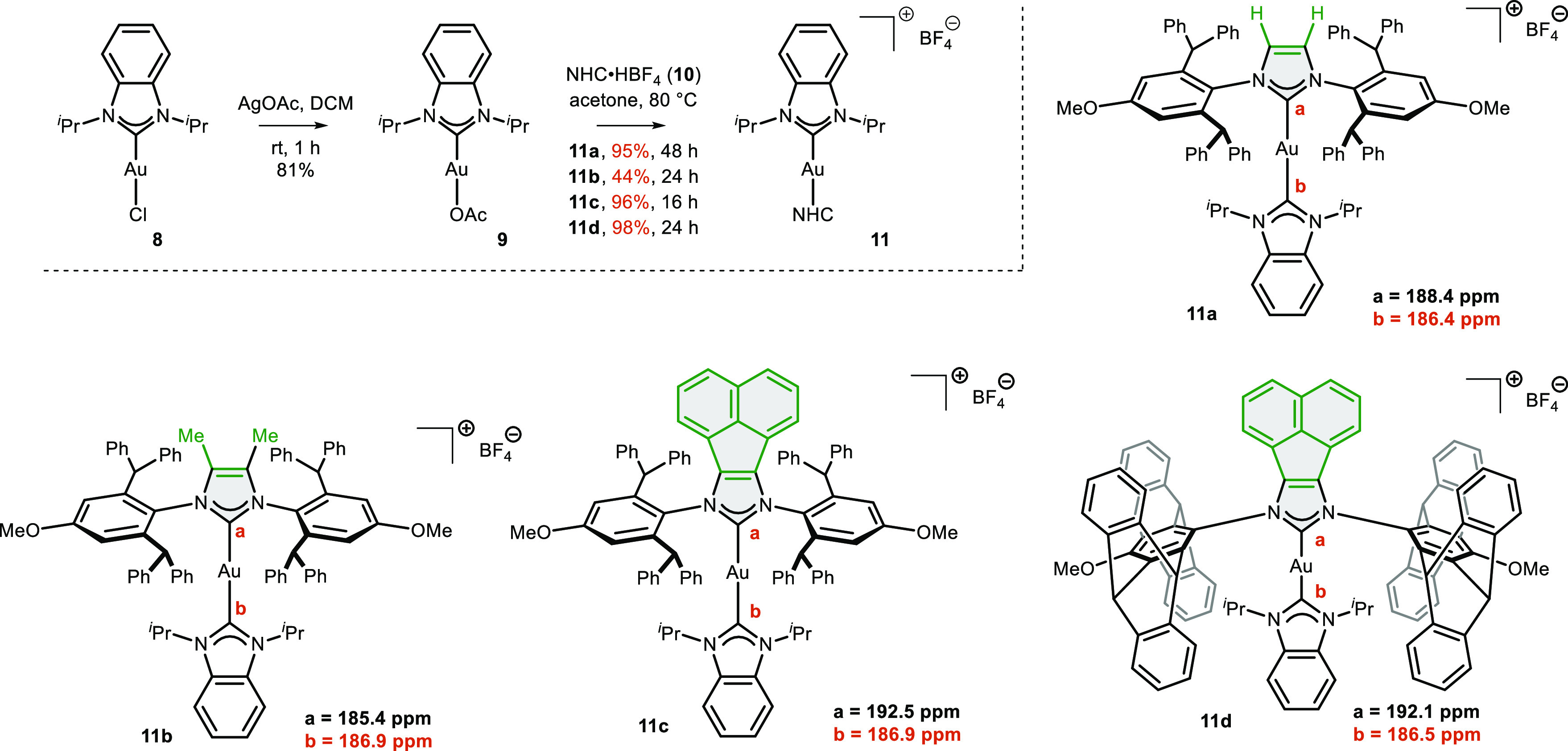
Synthesis of Heteroleptic [NHC(*i*-Pr_2_-bimy)Au]BF_4_ Complexes and Comparison of the ^13^C{^1^H} NMR Chemical Shifts of Their Carbene Carbon

To shed some light on the structure–reactivity relationship of these NHC ligands, we investigated their electronic and steric properties. First, we tried to estimate their σ-donor properties by measuring the ^13^C{^1^H} NMR spectra of heteroleptic gold complexes of the type [NHC(*i*-Pr_2_-bimy)Au]BF_4_, where *i*-Pr_2_-bimy is a 1,3-diisopropylbenzimidazolin-2-ylidene NHC ligand. The original methodology developed by Huynh and co-workers^36^ used the ^13^C{^1^H} NMR chemical shift of the carbene carbon atom of palladium(II) NHC complexes of the type *trans*-[PdBr_2_(*i*-Pr_2_-bimy)L]. If palladium complexes are not easily accessible, heteroleptic gold(I) complexes [NHC^1^(*i*-Pr_2_-bimy)Au]X could be used instead.^37^ Generally, a stronger donating ligand induces a downfield shift in the ^13^C{^1^H} NMR signal of the carbene atom of the probe, i.e., the *i*-Pr_2_-bimy ligand. Thus, four heteroleptic gold(I) complexes, **11a**–**11d**, were synthesized via a route employing (*i*-Pr_2_-bimy)AuOAc.^37^ The characteristic signal of the carbene *i*-Pr_2_-bimy ligand in [NHC^1^(*i*-Pr_2_-bimy)Au]X was assigned by HMBC analyses in each case (Scheme 6). It was found that replacing the hydrogen atoms (complex **11a**) bonded to the imidazolium core with methyl groups (complex **11b**) slightly increases the σ-donor character of the NHC ligand, which is consistent with literature data for structurally similar NHC ligands.^38^ Complexes **11c** and **11d** bearing a polyaromatic acenaphthylene backbone exhibited chemical shifts for the carbene carbon atom that were very similar to complex **11b**. It should be noted that Szostak and others^26^ have suggested that NHC–BIAN-type ligands are stronger σ-donors and have better π-acceptor character than the classical imidazolylidene NHCs, which is in accordance with our assumption.

Considering that the electronic properties of any NHC ligand used will be difficult to determine, we reasoned that the steric properties should be an important factor for catalytic activity. The steric bulk of NHCs can be expressed by the percent buried volume (%V_bur_), a general descriptor initially developed for NHCs^39^ by Cavallo and co-workers,^25a^ introduced for the first time in 2003 in a combined experimental/theoretical work by Nolan, Cavallo, and co-workers.^25c^ The calculated %V_bur_ values for the **4a**–**h** series are 62.6% (**4a**), 56.5% (**4b**), 65.3% (**4c**), 58.4% (**4d**), 62.5% (**4e**), 58.7% (**4f**), 50.8% (**4g**), and 48.7% (**4h**). We were surprised to find that the best-performing catalysts **4g** and **4h** had the lowest %V_bur_ in the series, despite having the bulkiest substituents. In part, the reason is based on the rigidity imposed by the NHC backbone in those systems, as well as in **4e** and **4f**.^40^ Thus, the N-appended aryl rings on the imidazole are sterically constrained and unable to rotate. Furthermore, the interaction of the imidazole with the aryl rings pushes them up.^41^ The most opened C–N–C angle between the linking carbon atom of the NHC ligand increases from 121.0° for **4a** to 123.1 and 125.7° for **4g** and **4h**, respectively. In fact, the correlation between %V_Bur_ and the observed catalytic conversions and yields did not result in any Pearson coefficients above 0.8. Worse correlations were obtained for the silver complexes. However, selectively removing the values of the most rigid species in the **4e**–**4g** series, i.e., **4g** and **4h**, we increase the *R*^2^ to 0.887. Also, removing **4e** and **4f** produces a greater increase to 0.985. For the copper series, even though the agreement was good, removing **4e** improves this to a near-perfect linear fit (*R*^2^ = 0.9997). However, with just three points, the statistical significance remains low. And since the same reasoning is not applicable to the silver series, no final judgment can be made.

The disparity between %V_bur_ values for the chloride compounds (**4a**–**h**) with both metals, copper and silver, hints that the series of NHC ligands are flexible. The values for silver are 5% larger than for copper. Tied to the flexibility of the substituents on the NHCs, the energy barrier of the rate-determining step (rds) gives insight into the measurement of %V_bur_, i.e., the NHC that is maximally tensioned but stable in that tensioned conformation. Thus, it is fundamental to also characterize the reaction pathway.

**Scheme 7:**
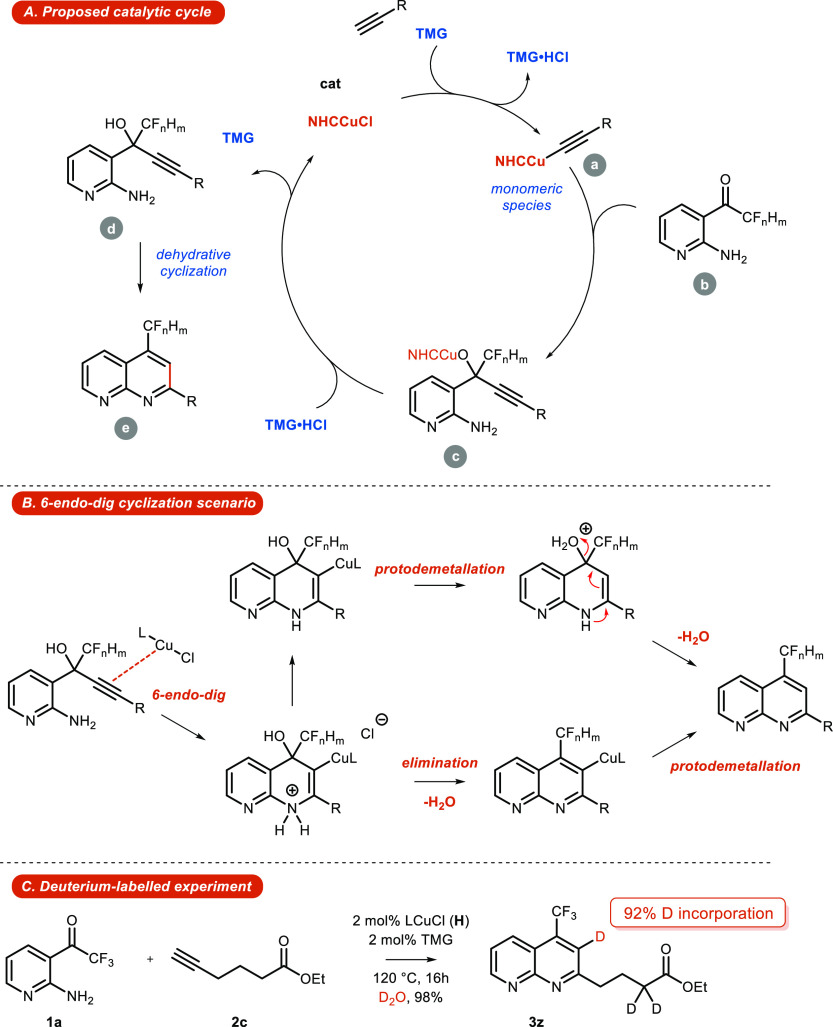
Plausible Catalytic Cycle and Scheme of Deuterium-Labeled Experiment

A plausible mechanistic cycle is depicted in Scheme 7. The catalytic process commences with the formation of the copper acetylide (**a**) proceeding via a well-established π-activation mode. Within a minute of combining the NHC–BIAN–CuCl complex with the terminal alkyne and base (**b**), the formation of a yellow solid was observed. The copper acetylide then undergoes 1,2-addition, producing a propargylic alkoxide (**c**). It should be noted that the NHC ligand plays a dual role in this process, forming a nucleophilic acetylide and promoting a mononuclear intermediate, enabling the addition. The mononuclear structure of the NHC copper acetylide was confirmed by Jones and co-workers by X-ray crystallographic analysis of an IPrCuC≡CPh complex.^42^ The role of the NHC ligand was further evidenced by the lack of formation of naphthyridine when the reaction is attempted with a stoichiometric amount of polymeric copper phenylacetylide (PhC≡CCu)*_n_* (see the SI). The last step of the catalytic cycle involves the protonation of copper alkoxide by TMG hydrochloride, regenerating the NHC–Cu–Cl catalyst. The propargylic alcohol (**d**) then might undergo either spontaneous or water-assisted dehydrative cyclization. Unfortunately, all experimental attempts to isolate this intermediate have failed. To confirm the beneficial role of copper in the 6-*endo*-*dig* cyclization step, preparation of **3z** was undertaken in D_2_O. This test reaction produced naphthyridine **3z** in a virtually quantitative yield with 92% deuterium incorporation into the aromatic ring (full proton–deuterium exchange was also detected in the α position of the ester functionality; Scheme 7).

**Scheme 8:**
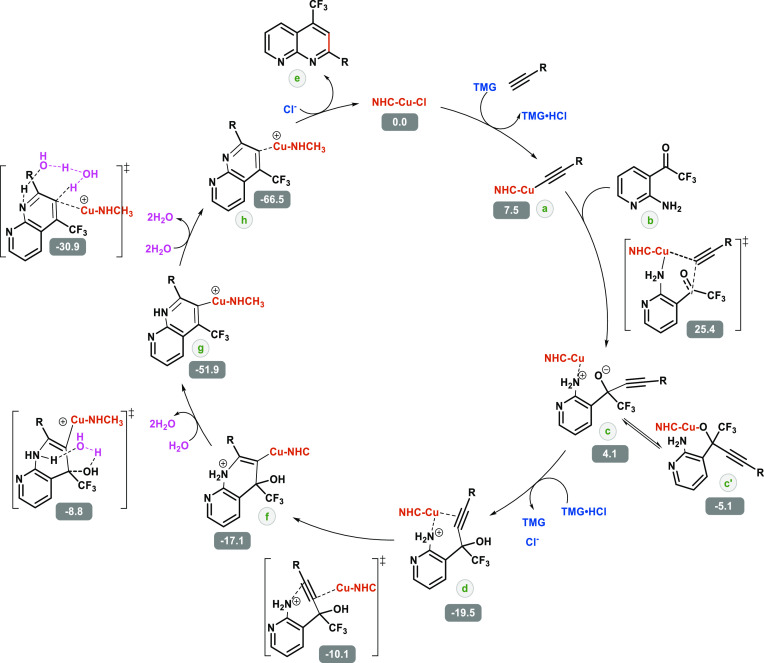
Reaction Mechanism of the NHC–Cu(I)-Catalyzed Friedländer-Type Annulation of 2-Amino-3-trifluoroacetyl-pyridines with a Terminal Alkyne Using the Pentiptycene NHC Complex Steps assisted by a molecule of water are shown in purple, with relative Gibbs energies in kcal/mol.

We conducted DFT calculations, screening the whole reaction pathway (Scheme 8) to find out the rds and any other kinetically significant steps. First, the reactant TMG is responsible for the deprotonation of the alkyne substrate, not as a single moiety, i.e., TMG·HCl, but as separate ions, the TMG·H^+^ cation and Cl^–^ anion, since the ionic scenario is more stable by 7.5 kcal/mol.^43^ Therefore, the proton is readily replaced by the cationic Cu–NHC moiety despite the large difference in size. The intermediate **a** then sees amine **b**, although its metallic center is hardly affected and does not lose the linear axis C–Cu–C until the transition state where the C–C bond between the former alkyne and the keto group of the 2-amino-3-trifluoroacetyl-pyridines is formed. This overcomes an energy barrier of 25.4 kcal/mol calculated from the initial NHC–Cu–Cl catalyst. Although the nitrogen of **b** has a favorable interaction with the metal in the transition state, the resulting intermediate **c** is in equilibrium with isomer **c′** where the oxygen of the former ketone group coordinates to the copper instead. The transition state where there is a Cu···O interaction was also studied, but it is less favorable by 2.9 kcal/mol (see Figure S6 for further details). For the protonation of oxygen by the proton previously extracted by TMG, an increase in a thermodynamic stability of 23.6 kcal/mol occurs in the transition from **c** to **d**. A transition-state energy barrier of 9.4 kcal/mol is determined for the cyclization forming the C–N bond. Formation of **g** from **f** is assisted by a molecule of water, which facilitates proton transfer from the positively charged nitrogen to the hydroxyl group, which leaves as water in a condensation step. This step requires 8.3 kcal/mol and leads to a thermodynamic stabilization of 34.8 kcal/mol. Again, two water molecules facilitate the transfer of the remaining proton in intermediate **g** from the nitrogen to the carbon attached to the metal, with the following kinetic and thermodynamic energies of 21.0 and 14.6 kcal/mol, respectively. This gives way to the release of the organic product, exchanging it for a chloride anion and thus closing the catalytic cycle. This step was also studied with one and three water molecules resulting in higher kinetic costs of 14.9 and 1.4 kcal/mol, respectively.

**Figure 2:**
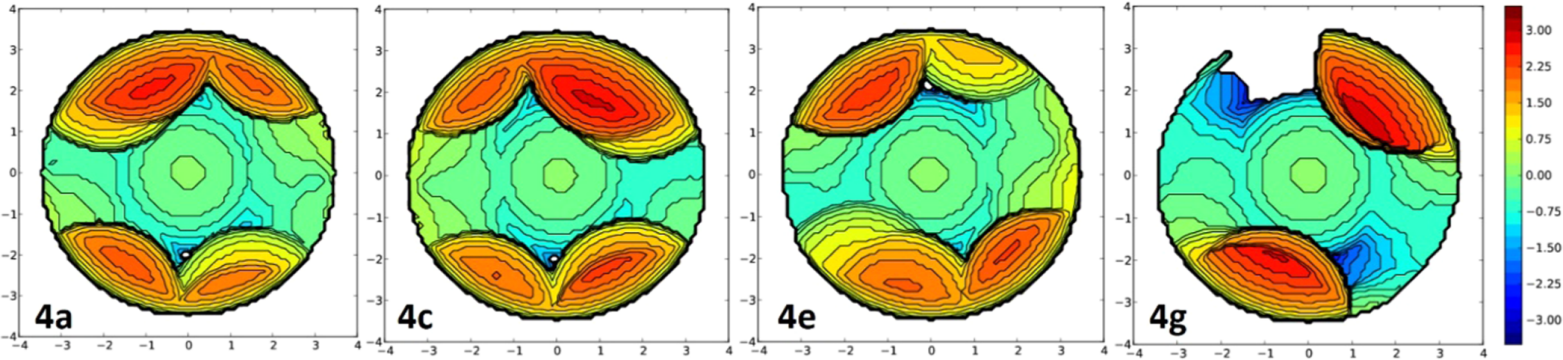
Steric maps of the *xy* plane for copper systems **4a**, **4c**, **4e**, and **4g** (centered on the metal, *z* axis defined by the C_NHC_, *xz* plane containing any of the N atoms of the imidazole ring; curves are given in Å).

The kinetic energy barrier of the rds for each of the complexes studied is compiled in Table 2 along with the anterior and posterior intermediates. This is not only to define the rds barrier but also to account for whether the formation of the **c′** isomer poses a problem for reaction efficiency. Thermodynamics does not seem to indicate anything, and therefore efforts must be based on kinetics, and understanding the transition state of the rds is fundamental. Apart from observing a significant difference of around 5 kcal/mol that explains the higher conversions for the copper catalytic systems, results do not follow a clear trend in any of the metal catalyst series. While there appears to be a correlation (*R*^2^ = 0.707) between this energy barrier and the conversion, it does not proceed to give high product yields. Going into further detail, it is shown that the ratio is maintained by copper complexes, with an acceptable correlation (*R*^2^ = 0.736), indicating that the higher the barrier, the lower the yield. Returning to %V_bur_, the combination of both variables, i.e., %V_bur_ and energy barrier, only gave good agreement for the catalytic conversion values (*R*^2^ = 0.809). For copper complexes, the correlation is good for yield (*R*^2^ = 0.819) and even better for conversion (*R*^2^ = 0.953). Although there are insufficient data to provide strong statistical significance, it does suggest that a lower %V_bur_ may improve the reaction studied and explain why the catalytic system **4g** is the best for this reaction. The steric maps in Figure 2 represent another validation of this hypothesis.^44^ Although there are two quadrants around the metal center that are sterically hindered for the pentiptycene-based NHC ligand system **4g**, overall it is less hindered than the other complexes. In fact, the other two quadrants are hardly affected by the corresponding NHC ligand with values of 28.1 and 41.0%, thus much lower compared to any of the other three systems (see Tables S2 and S3), as the least occupied quadrant is 55.7, 59.9, and 54.3% for **4a**, **4c**, and **4d**, respectively (see the SI for further details).

**Table 2:** Relative Gibbs Energies (in kcal/mol) of the C–C Bond Formation between the Alkyne and 2-Amino-3-trifluoroacetyl-pyridine Catalyzed by the Metal Catalysts

system	cat	a	a + b → c	c	c′
**4a**	0.0	5.9	27.3	14.4	–0.7
**4b**	0.0	5.8	20.7	13.5	5.7
**4c**	0.0	8.2	30.5	10.2	0.9
**4d**	0.0	9.6	25.8	16.3	6.5
**4e**	0.0	6.1	24.9	15.9	7.7
**4f**	0.0	8.6	19.6	16.1	0.2
**4g**	0.0	7.5	25.4	4.1	–5.1
**4h**	0.0	9.8	19.6	9.4	5.4

## Conclusions

A practical and scalable method for the synthesis of fluorinated naphthyridines has been developed based on a modified Friedländer reaction between terminal alkynes and fluorinated *ortho*-aminophenones, catalyzed by NHC–BIAN–Cu–Cl complexes on water. Utilizing a unique NHC ligand that combines the steric bulk of pentiptycene pendant groups with the π-extended backbone of acenaphthylene was crucial for the successful development of a direct catalytic alkynylation/dehydrative cyclization sequence. The established method accommodates a variety of terminal alkynes including those derived from natural sources or biologically active substances. In addition, mechanistic studies and computational calculations unveil the whole reaction pathway identifying the rds to be that of the C–C bond formation between the alkyne and fluorinated 2-amino-3-fluoroacetyl-pyridines. It was possible to describe in detail that the magnitude of the largest barrier was significantly lower for silver systems. Further, to explain the different performances of the eight studied systems, a correlation analysis of variables was done to explain the different conversions and experimental yields. This enabled the observation of certain trends linking the variables of steric congestion at the metal center, by means of the steric index %V_bur_, and also the energy barrier of the rds. DFT calculations also revealed the fundamental role of water as a proton shuttle in two steps.

## Experimental Section

### General Remarks

NMR spectra were recorded in CDCl_3_ or DMSO-*d*_6_ solutions (unless indicated otherwise); chemical shifts are quoted on the δ scale, ppm, with the solvent signal as the internal standard (CHCl_3_, ^1^H NMR 7.26 ppm; CDCl_3_, ^13^C{^1^H} NMR 77.00 ppm, DMSO-*d*_6_ 2.50 ppm, ^13^C{^1^H} NMR 39.40 ppm, ^13^C{^1^H} NMR CD_3_OD 49.00 ppm). High-resolution mass spectra (HR MS) were taken using the EI technique or electrospray ionization (ESI). Column chromatography was performed on Merck silica gel 60, 230–400 mesh. TLC was performed on aluminum sheets, Merck 60F 254. Anhydrous solvents were obtained by distillation over CaH_2_ (CH_2_Cl_2_) or Na/benzophenone (THF, hexane, MTBE, toluene). Air-sensitive reactions were performed in flame-dried glassware under an atmosphere of argon. Organic extracts were dried, and solvents were evaporated in a rotary evaporator.

Reagents were used as they were purchased unless otherwise indicated. Alkynes **2a**, **2c**, **S1**–**5**, **S9**, **S11** and **S21**, and **S23**–**25** were commercially available and used as received. Aminophenones **1a**(23c) and **1b**(23c) and alkynes **S6**,^23c^**S8**,^45^**S10**,^46^**S14**,^47^**S19**,^48^**S2**,^49^ and **S22**(50) were prepared according to the literature procedure (for details, see the SI, Schemes S1–S3). The names of compounds were generated using ACD Lab Name 12.0 software. Complexes **7a**(23c) and **4b**(23c) were synthesized according to the literature procedure.

### Synthesis of N-Heterocyclic Carbene Precursors Containing Chloride Anion

2,6-Bis(diphenylmethyl)-4-methoxyaniline (**5a**)^51^ and pentiptycene-derived bisimine^33^**6d** were synthesized according to the literature procedure.

#### (2*E*,3*E*)-*N*,*N***′**-Bis[2,6-bis(diphenylmethyl)-4-methoxyphenyl]butane-2,3-diimine (**6b**)

The compound was synthesized according to the modified literature procedure.^52^ To a two-necked round-bottom flask were charged with 2,6-dibenzhydryl-4-methoxyaniline (**5a**) (2.0 g, 4.39 mmol, 2.0 equiv), butano-2,3-dione (0.2 mL, 2.19 mmol, 1.0 equiv), *p*-TSA (15.1 mg, 0.09 mmol, 2 mol %), and toluene (50 mL). The resulting solution was heated at 80 °C for 24 h. Then, the flask was equipped with the Dean–Stark apparatus and heated to reflux for 3 days. Then, the solvent was evaporated and the residue was treated with MeOH (40 mL). The resulting yellow solid was washed with MeOH (3 × 5 mL) and dried under high vacuum to give **6b** as a yellow solid (990.9 mg, 23%). ^1^H NMR (200 MHz, CDCl_3_) δ 7.35–6.90 (m, 40H), 6.42 (s, 4H), 5.16 (s, 4H, C**H**Ph_2_), 3.53 (s, 6H, OC**H**_3_), 1.15 (s, 6H, N=CC**H**_3_). Spectral data are in agreement with those reported.^52^

#### (1*E*,2*E*)-*N*,*N***′**-Bis[2,6-bis(diphenylmethyl)-4-methoxyphenyl]acenaphthylene-1,2-diimine (**6c**)

The compound was synthesized according to the modified literature procedure.^53^ To a suspension of acenaphthoquinone (350.0 mg, 1.91 mmol) in glacial AcOH (30 mL) were added ZnCl_2_ (230.0 mg, 2.20 mmol, 1.0 equiv) and 2,6-dibenzhydryl-4-methoxyaniline (2.0 g, 4.39 mmol, 2.3 equiv). The resulting mixture was heated at 120 °C under an atmosphere of argon for 16 h. Thus, the formed zinc/bisimine complex was filtered, washed with AcOH (3 × 3 mL) and Et_2_O (3 × 10 mL), and subjected to decomplexation.

The resulting solid was suspended in DCM (27 mL), and potassium oxalate (809.0 mg) in water (4 mL) was added and stirred at rt for an additional 1 h. The resulting orange solution was extracted with DCM (2 × 10 mL), and the combined organic extracts were washed with water (3 × 10 mL), dried over MgSO_4_, and evaporated to give **6c** as an orange solid (1.33 g, 66%). ^1^H NMR (400 MHz, CDCl_3_) δ 7.51 (d, *J* = 8.2 Hz, 2H), 7.17–7.02 (m, 20H), 6.90–6.80 (m, 10H), 6.69 (s, 4H), 6.67–6.58 (m, 12H), 6.16 (d, *J* = 7.2 Hz, 2H), 5.71 (s, 4H, C**H**Ar_2_), 3.66 (s, 6H, OC**H**_3_). Spectral data are in agreement with those reported.^53^

**Salt 7b** was synthesized according to a modified literature procedure.^53^ A 50 mL sealed tube was charged with bisimine **6b** (1.0 g, 1.0 mmol) and CH_3_CH_2_OCH_2_Cl (3.0 mL, 32.40 mmol, 32.4 equiv) and heated at 100 °C (temp. of oil bath) for 16 h. Then, the reaction mixture was evaporated, and the residue was chromatographed on silica (5% MeOH/DCM) to give a light brown solid (978.3 mg, 93%). mp > 260 °C (decomposition, analytical sample was precipitated from a mixture of DCM/Et_2_O); ^1^H NMR (400 MHz, CDCl_3_) δ 12.40 (s, 1H, NC**H**N), 7.42–6.85 (m, 40H), 6.67 (s, 4H), 5.12 (s, 4H, C**H**Ph_2_), 3.59 (s, 6H, OC**H**_3_), 0.67 (s, 6H, C**H**_3_); ^13^C{^1^H} NMR (100 MHz, CDCl_3_) δ 160.6 (N**C**HN), 142.8, 141.6, 140.3, 129.8, 129.4, 128.5, 127.2, 126.6, 123.5, 115.7, 77.2, 55.1 (O**C**H_3_), 51.5 (**C**HPh_2_), 7.2 (**C**H_3_); HR MS (ESI TOF) *m*/*z* calcd for C_71_H_61_N_2_O_2_ [M – Cl]^+^: 973.4733; found: 973.4724.

**Salt 7c**: A 50 mL sealed ampule was charged with bisimine **6c** (940.0 mg, 0.89 mmol) and CH_3_CH_2_OCH_2_Cl (4.1 mL, 44.5 mmol, 50.0 equiv) and heated at 100 °C (temp. of oil bath) for 16 h. Then, the reaction mixture was evaporated, and the residue was treated with Et_2_O (10 mL). The resulting solid was filtered and washed with water (2 × 50 mL) to give a yellow solid (618.9 mg, 63%). mp 311.0–312.0 °C (analytical sample was precipitated from a mixture of DCM/Et_2_O); ^1^H NMR (400 MHz, CDCl_3_) δ 13.13 (s, 1H, NC**H**N), 7.60 (d, *J* = 8.2 Hz, 2H), 7.29–7.13 (m, 18H), 7.03–6.94 (m, 6H), 6.78–6.56 (m, 22H), 6.26 (d, *J* = 7.0 Hz, 2H), 5.31 (s, 4H, C**H**Ph_2_), 3.61 (s, 6H, OC**H**_3_); ^13^C{^1^H} NMR (100 MHz, CDCl_3_) δ 161.0 (N**C**HN), 143.1, 141.5, 140.5, 137.8, 129.7, 129.4, 129.2, 128.7, 128.6, 128.3, 128.1, 126.8, 126.7, 124.7, 123.0, 122.2, 115.7, 55.3 (O**C**H_3_), 51.8 (**C**HPh_2_); HR MS (ESI TOF) *m/z* calcd for C_79_H_61_N_2_O_2_ [M – Cl]^+^: 1069.4733; found: 1069.4744.

**Salt 7d** was synthesized according to the modified literature procedure.^33^ A 50 mL sealed tube was charged with pentiptycene-derived bisimine **6d**(33) (648.2 mg, 0.59 mmol) and CH_3_CH_2_OCH_2_Cl (4.0 mL, 43.3 mmol, 73.2 equiv) and heated at 80 °C (temp. of oil bath) for 16 h. Then, the reaction mixture was evaporated with Et_2_O (10 mL). The resulting solid was filtered, washed with Et_2_O (3 × 10 mL), and further purified by chromatography on silica (DCM, 5% MeOH/DCM) to give a yellow solid (285.2 mg, 42%). ^1^H NMR (400 MHz, CD_2_Cl_2_) δ 8.23 (d, *J* = 8.2 Hz, 2H), 8.07 (s, 1H, NC**H**N), 7.57–7.42 (m, 10H), 7.32 (d, *J* = 7.3 Hz, 4H), 7.24 (d, *J* = 7.2 Hz, 4H), 7.13–6.99 (m, 8H), 6.98–6.88 (m, 6H), 6.87–6.79 (m, 4H), 6.01 (d, *J* = 7.0 MHz, 8H, C**H**Ar_3_), 4.19 (s, 6H, OC**H**_3_). Spectral data are in agreement with those reported.^33^

### Synthesis of N-Heterocyclic Carbene Precursors Containing Tetrafluoroborate Anion

The experimental protocol for the anion exchange (from chloride to tetrafluoroborate) developed by Nolan^51^ was implemented in all cases described below.

**Salt 10a** was synthesized according to the modified literature procedure.^51^ To a suspension of salt **7a** (200.0 mg, 0.20 mmol, 1.0 equiv) in a mixture of THF (330 μL) and H_2_O (6 mL), 48% HBF_4(aq)_ (29.0 μL, 0.30 mmol, 1.5 equiv) was added and stirred at rt for 16 h (the progress of the reaction was monitored by TLC, **7a***R*_f_ = 0.26, **10a***R*_f_ = 0.53, 10% MeOH/DCM). Then, the reaction mixture was extracted with DCM (3 × 5 mL), and the combined organic extracts were dried over Na_2_SO_4_ and evaporated. The residue was washed with *n*-pentane (3 × 10 mL) and dried under high vacuum to give a white solid (171.2 mg, 83%). ^1^H NMR (400 MHz, CDCl_3_) δ 10.44 (br s, 1H, NC**H**N), 7.34–7.00 (m, 32H), 6.87–6.80 (m, 8H), 6.50 (s, 4H), 5.60 (s, 2H), 5.10 (s, 4H, C**H**Ph_2_), 3.53 (s, 6H, OC**H**_3_). Spectral data are in agreement with those reported.^51^

**Salt 10b**: To a suspension of salt **7b** (300.0 mg, 0.29 mmol, 1.0 equiv) in a mixture of THF (0.5 mL) and H_2_O (9 mL), 48% HBF_4(aq)_ (43.5 μL, 0.45 mmol, 1.5 equiv) was added and stirred for 16 h (the progress of the reaction was monitored by TLC, **7b***R*_f_ = 0.05, **10b***R*_f_ = 0.32, 5% MeOH/DCM). Then, the reaction mixture was extracted with DCM (3 × 5 mL), and the combined organic extracts were dried over Na_2_SO_4_, filtered, and evaporated to give salt **12b** as a light brown solid (310.0 mg, 98%). mp > 300 °C (decomposition, analytical sample was precipitated from a mixture of DCM/*n*-pentane); ^1^H NMR (400 MHz, CDCl_3_) δ 9.90 (s, 1H, NC**H**N), 7.40–6.88 (m, 40H), 6.66 (br s, 4H), 4.94 (s, 4H, C**H**Ph_2_), 3.60 (s, 6H, OC**H**_3_), 0.73 (s, 6H, C**H**_3_); ^13^C{^1^H} NMR (100 MHz, CDCl_3_) δ 160.9 (N**C**HN), 142.9, 141.3, 140.6, 137.7, 130.5, 129.6, 129.2, 128.7, 127.4, 127.0, 123.4, 115.9, 55.2 (O**C**H_3_), 51.7 (**C**HPh_2_), 7.5 (**C**H_3_); ^19^F NMR (376 MHz, CDCl_3_) δ −150.9 (×2); HR MS (ESI TOF) *m/z* calcd for C_71_H_61_N_2_O_2_ [M – BF_4_]^+^: 973.4733; found: 973.4733.

**Salt 10c**: To a suspension of salt **7c** (200.0 mg, 0.18 mmol, 1.0 equiv) in THF (0.3 mL) and H_2_O (6 mL), 48% HBF_4(aq)_ (26.1 μL, 0.27 mmol, 1.5 equiv) was added and stirred at rt for 16 h (the progress of the reaction was monitored by TLC, **7c***R*_f_ = 0.22, **10c***R*_f_ = 0.50, 5% MeOH/DCM). Then, the reaction mixture was extracted with DCM (5 × 5 mL), and the combined organic extracts were dried over Na_2_SO_4_, filtered, and evaporated to give salt **12c** as a yellow solid (170.1 mg, 81%). mp > 360 °C (decomposition, analytical sample was precipitated from a mixture of DCM/*n*-pentane); ^1^H NMR (400 MHz, CDCl_3_) δ 9.97 (s, 1H, NC**H**N), 7.70 (d, *J* = 8.3, 2H), 7.21–7.13 (m, 8H), 7.11–7.05 (m, 2H), 7.04–6.93 (m, 12H), 6.82–6.62 (m, 24H), 6.35 (d, *J* = 7.0 Hz, 2H), 5.13 (s, 4H, C**H**Ph_2_), 3.63 (s, 6H, OC**H**_3_); ^13^C{^1^H} NMR (50 MHz, CDCl_3_) δ 161.1 (N**C**HN), 143.0, 141.0, 140.7, 138.1, 129.5, 129.3, 129.2, 128.6, 128.4, 128.2, 127.1, 126.8, 124.4, 123.3, 121.9, 115.6, 55.3 (O**C**H_3_), 51.8 (**C**HAr_2_); ^19^F NMR (376 MHz, CDCl_3_) δ −150.1, −150.2; HR MS (ESI TOF) *m/z* calcd for C_79_H_61_N_2_O_2_ [M – BF_4_]^+^: 1069.4733; found: 1069.4733.

**Salt 10d**: To a suspension of salt **7d** (200.0 mg, 0.17 mmol, 1.0 equiv) in a mixture of THF (0.30 mL) and H_2_O (6 mL), 48% HBF_4(aq)_ (25.1 μL, 0.26 mmol, 1.5 equiv) was added, and the reaction mixture was stirred at rt for 16 h (the progress of the reaction was monitored by TLC, **7d***R*_f_ = 0.25, **10d***R*_f_ = 0.45, 5% MeOH/DCM). Then, the reaction mixture was extracted with DCM (4 × 5 mL), and the combined organic extracts were dried over Na_2_SO_4_ and evaporated. The residue was treated with *n*-pentane (10 mL), stirred for 5 min, filtered, and dried under high vacuum to give **10d** as a yellow solid (175.0 mg, 84%). mp > 350 °C (decomposition, analytical sample was precipitated from a mixture of DCM/*n*-pentane); ^1^H NMR (400 MHz, CDCl_3_) δ 8.16 (d, *J* = 8.0 MHz, 2H), 7.83 (s, 1H, NC**H**N), 7.56–7.33 (m, 9H), 7.32–7.13 (m, 9H), 7.12–6.68 (m, 18H), 5.96 (s, 4H, C**H**Ar_3_), 5.74 (s, 4H, C**H**Ar_3_), 4.19 (s, 6H, OC**H**_3_); ^13^C{^1^H} NMR (100 MHz, CDCl_3_) δ 152.7 (N**C**HN), 144.4, 144.0, 143.7, 143.4, 141.5, 139.1, 135.1, 131.6, 126.1, 126.0, 125.8, 125.7, 125.4, 124.2, 124.0, 123.7, 121.8, 120.3, 63.1 (O**C**H_3_), 49.5 (**C**HAr_3_), 48.2 (**C**HAr_3_); ^19^F NMR (376 MHz, CDCl_3_) δ −150.8 (×2); HR MS (ESI TOF) *m/z* calcd for C_83_H_53_N_2_O_2_ [M – BF_4_]^+^: 1109.4107; found: 1109.4117.

### Synthesis of N-Heterocyclic Carbene Copper(I) Complexes

**Complex 4a** was synthesized according to the literature procedure.^23c^^13^C{^1^H} NMR (50 MHz, CD_2_Cl_2_) δ 181.6 (N*C*N), 160.6, 143.4, 143.2, 142.9, 130.1, 130.0, 129.8, 129.1, 129.0, 127.3, 127.2, 124.0, 115.3, 55.7, 52.0. ^13^C{^1^H} NMR data are in agreement with those reported. However, the authors did not record a carbene atom in the ^13^C NMR spectrum. A prolonged acquisition time was required, at least 24 h (the equally long acquisition time was also needed for the other copper(I) and silver complexes).

**Complex 4c**: A 20 mL screw cap vial was charged with salt **7b** (150.0 mg, 0.15 mmol), CuCl (18.3 mg, 0.19 mmol, 1.2 equiv), and K_2_CO_3_ (42.6 mg, 0.31 mmol, 2.0 equiv). Then, the vial was transferred to a glovebox and anhydrous acetone (10.5 mL) was added. The resulting suspension was heated at 60 °C in an aluminum heating block (thermocouple was inserted into a separate vial filled with silicon oil) for 16 h. Then, the reaction mixture was filtered through a pad of Celite 545 (washing with acetone), and the solvent was evaporated. The residue was chromatographed on silica (1% acetone/DCM); however, some impurities have been still observed. The crude complex was dissolved in a minimal volume of DCM (1 mL) and precipitated with *n*-pentane (16 mL, precipitation was repeated twice) to give a light brown solid (82.5 mg, 50%). mp > 300 °C (decomposition, DCM/*n*-pentane); IR (KBr) 3058, 3024, 2926, 2838, 1642, 1599, 1493, 1469, 1445 cm^–1^; ^1^H NMR (400 MHz, CD_2_Cl_2_) δ 7.30–6.95 (m, 40H), 6.70 (s, 4H), 5.27 (s, 4H, C**H**Ar_2_), 3.64 (s, 6H, OC**H**_3_), 0.67 (s, 6H, C**H**_3_); ^13^C{^1^H} NMR (50 MHz, CD_2_Cl_2_) δ 186.7, 160.6, 143.9, 142.8, 141.8, 139.6, 130.1, 130.1, 129.9, 129.2, 128.9, 128.6, 128.5, 127.7, 127.1, 127.0, 126.9, 124.4, 122.2, 115.5, 55.8 (O**C**H_3_), 52.2 (**C**HAr_2_) (despite prolonged drying under high vacuum, residual signals of *n*-pentane were detected). HR MS (ESI TOF) *m/z* calcd for C_71_H_60_CuClN_2_O_2_Na [M + Na]^+^: 1093.3537; found: 1093.3538.

**Complex 4e**: A 4 mL screw cap vial was charged with salt **7c** (150.0 mg, 0.14 mmol), CuCl (16.6 mg, 0.17 mmol, 1.2 equiv), and K_2_CO_3_ (38.7 mg, 0.28 mmol, 2.0 equiv). Then, the vial was transferred to a glovebox, anhydrous acetone (1.5 mL) was added, and the resulting suspension was heated at 60 °C for 16 h in an aluminum heating block (thermocouple was inserted into a separate vial filled with silicon oil). Then, the solvent was evaporated, and the residue was treated with MeOH (5 mL) and centrifugated (6000 rpm, 15 min). The mother liquid was removed by Pasteur pipette, and thus, the obtained solid was treated with MeOH (11 mL) and centrifugated (6000 rpm, 15 min). The mother liquid was removed by Pasteur pipette, and the solid was treated with MeOH, and the suspension was transferred to a round-bottom flask. Then, the solvent was evaporated and the residue was dried under high vacuum to give a yellow solid (81.0 mg, 51%). mp > 351 °C (decomposition, analytical sample was precipitated from a mixture of DCM/*n*-pentane); IR (KBr) 3083, 3058, 3024, 2932, 2838, 1598, 1584, 1493, 1468, 1446 cm^–1^; ^1^H NMR (400 MHz, CD_2_Cl_2_) δ 7.55 (d, *J* = 8.4 Hz, 2H), 7.20–6.55 (m, 46H), 6.19 (d, *J* = 6.9 Hz, 2H), 5.44 (br s, 4H, C**H**Ar_2_), 3.67 (s, 6H, O**C**H_3_); ^13^C{^1^H} NMR (100 MHz, CD_2_Cl_2_) δ 160.1, 143.4, 142.3, 141.3, 139.2, 129.6, 129.3, 128.7, 128.4, 128.0, 127.2, 126.6, 126.4, 123.9, 121.7, 115.0, 55.2 (O**C**H_3_), 51.6 (**C**HAr_2_); HR MS *m/z* (APCI TOF) calcd for C_79_H_60_CuN_2_O_2_ [M – Cl]^+^: 1131.3951; found: 1131.3942.

**Complex 4g**: A 20 mL screw cap vial was charged with salt **7d** (191.0 mg, 0.17 mmol), CuCl (19.8 mg, 0.20 mmol, 1.2 equiv), and K_2_CO_3_ (46.0 mg, 0.33 mmol, 2.0 equiv). Then, the vial was transferred to a glovebox and anhydrous acetone (10 mL) was added, and the reaction mixture was heated at 60 °C in an aluminum heating block (thermocouple was inserted into a separate vial filled with silicon oil). After 16 h, the solvent was evaporated and the residue was treated with MeOH (11 mL), centrifugated (6000 rpm, 10 min), and the mother liquid was removed by means of Pasteur pipette. The crude complex was treated with an additional portion of MeOH (11 mL) and centrifugated (6000 rpm, 10 min). After the removal of the mother liquid, complex **4g** was transferred to a flask and dried under high vacuum to give a yellow-olive solid (174.9 mg, 87%).

The same procedure was repeated on a 1 g scale using salt **7d** (1 g, 0.87 mmol), CuCl (103.7 mg, 1.05 mmol, 1.2 equiv), K_2_CO_3_ (240.5 mg, 1.74 mmol, 2.0 equiv), and acetone (40 mL). The reaction mixture was heated to reflux for 48 h (while the formation of a yellow solid was observed). The reaction mixture was diluted with MeOH (30 mL) and centrifugated (6000 rpm, 15 min), and the mother liquid was removed by means of Pasteur pipette. The residue was treated with MeOH (40 mL) and centrifugated (6000 rpm, 15 min). After the removal of the solvent by Pasteur pipette, the resulting solid was suspended in MeOH, transferred to a round-bottom flask, and evaporated to give a yellowish solid (890.0 mg, 81%). mp > 300 °C (decomposition, DCM/*n*-pentane); IR (KBr) 3065, 3019, 2967, 2829, 1711, 1602, 1479, 1459 cm^–1^; ^1^H NMR (400 MHz, CD_2_Cl_2_) δ 7.85 (d, *J* = 8.3 Hz, 2H), 7.54–7.46 (m, 8H), 7.42 (d, *J* = 7.1 Hz, 4H), 7.12–6.90 (m, 14H), 6.84–6.71 (m, 8H), 6.20 (d, *J* = 7.0 Hz, 2H), 5.97 (s, 4H, C**H**Ar_3_), 5.47 (s, 4H, C**H**Ar_3_), 4.19 (s, 6H, OC**H**_3_); ^13^C{^1^H} NMR (50 MHz, CD_2_Cl_2_) δ 186.3, 152.0, 145.7, 145.0, 144.9, 144.8, 144.3, 141.9, 139.8, 138.3, 131.2, 130.2, 129.2, 127.9, 126.4, 126.2, 125.6, 125.4, 125.2, 124.6, 124.3, 124.3, 124.3, 123.6, 63.7, 50.9, 48.8, 31.2 (spectra of the suspension has been recorded due to poor solubility of complex **4h** in CD_2_Cl_2_); HR MS (ESI TOF) *m/z* calcd for C_83_H_52_CuN_2_O_2_ [M – Cl]^+^: 1171.3325; found: 1171.3312.

### Synthesis of N-Heterocyclic Carbene Silver Complexes

**Complex 4b**:^54^ A flame-dried Schlenk was charged with salt **7a** (313.7 g, 0.319 mmol) and Ag_2_O (44.4 mg, 0.0192 mmol, 0.6 equiv). Then, anhydrous DCM (20 mL) was added and stirred at rt for 36 h (Schlenk tube was protected from light by an aluminum foil). Then, the reaction mixture was passed through a pad of silica (washing with 5% MTBE/hexane). The crude complex was dissolved in a minimal volume of DCM (6 mL) and crashed with *n*-pentane (28 mL). The resulting white solid was filtered, washed with *n*-pentane (3 × 10 mL), and dried under high vacuum to give **4b** (285.6 mg, 82%). All manipulations with complex **4b** were carried out in air without any precaution. Complex **4b** was stored under air for more than 24 months in the fridge in a glass vial without any decomposition, as judged by ^1^H NMR. ^1^H NMR (400 MHz, CD_2_Cl_2_) δ 7.27–7.18 (m, 26H), 7.04–6.91 (m, 16H), 6.60 (m, 4H), 5.98 (br d, *J* = 1.9 Hz, 2H), 5.15 (s, 4H), 3.59 (s, 6H); ^13^C{^1^H} NMR (50 MHz, CD_2_Cl_2_) δ 185.8 (dd, *J*_C-Ag_ = 248.3, 17.9 Hz, N**C**N), 160.7, 143.3, 143.3, 142.7, 130.4, 130.3, 130.0, 129.8, 129.3, 129.1, 127.4, 127.3, 124.5, 124.3, 115.5, 55.7, 52.0. Spectral data are in agreement with those reported.^54^

**Complex 4d**: A Schlenk tube was charged with salt **7b** (250.0 mg, 0.26 mmol) and Ag_2_O (36.0 mg, 0.15 mmol, 0.6 equiv), and anhydrous DCM (6.3 mL) was added. The resulting suspension was stirred at 55 °C (temp. of oil bath) for 16 h. Then, the reaction mixture was filtered through a pad of Celite 545 (washing with DCM, 2 × 10 mL), and the solvent was evaporated. The residue was purified by chromatography on silica (1% acetone/DCM). Thus, the obtained crude complex **4d** was dissolved in a minimal volume of DCM (1 mL) and crashed with *n*-pentane (6.5 mL), filtered, and dried under high vacuum to give a light brown solid (137.9 mg, 48%). All manipulations with complex **4d** were carried out in air without any precaution. Complex **4d** was stored under air for more than 15 months in the fridge in a glass vial without any decomposition, as judged by ^1^H NMR. mp > 300 °C (decomposition, DCM/*n*-pentane); IR (KBr) 3058, 3024, 2928, 2839, 1598, 1492, 1469, 1446 cm^–1^; ^1^H NMR (400 MHz, CD_2_Cl_2_) δ 7.27–7.19 (m, 12H), 7.11–7.00 (m, 20H), 6.98–6.92 (m, 8H), 6.67 (s, 4H), 5.16 (s, 4H, C**H**Ar_2_), 3.61 (s, 6H, OC**H**_3_), 0.77 (s, 6H, C**H**_3_); ^13^C{^1^H} NMR (50 MHz, CD_2_Cl_2_) δ 182.3 (dd, *J*_C-Ag_ = 251.5, 18.1 Hz, N*C*N), 18.1 Hz, 160.4, 143.8, 143.3, 142.1, 130.3, 130.2, 129.9, 129.8, 129.4, 129.3, 129.2, 129.1, 129.0, 127.5, 127.3, 127.2, 115.8, 55.7 (O**C**H_3_), 51.8 (**C**HAr_2_), 8.8(**C**H_3_); HR MS (ESI TOF) *m/z* calc for C_71_H_60_AgN_2_O_2_ [M – Cl]^+^: 1079.3706; found: 1079.3724.

**Complex 4f**: A flame-dried Schlenk tube was charged with salt **7c** (150.0 mg, 0.14 mmol) and Ag_2_O (19.5 mg, 0.08 mmol, 0.6 equiv). Then, anhydrous DCM (3.5 mL) was added and the resulting suspension was stirred at rt for 16 h. Then, the reaction mixture was filtered through a pad of Celite 545 (washing with DCM, 2 × 10 mL), and the solvent was evaporated. The crude complex was treated with MeOH (ca. 12 mL), centrifugated (6000 rpm, 15 min), and the mother liquid was removed by means of Pasteur pipette. The crude complex was treated with an additional portion of MeOH (11 mL) and centrifugated (6000 rpm, 15 min). After the removal of the mother liquid, complex **4f** was transferred to a flask and dried under high vacuum to give a bright yellow solid (82.1 mg, 50%). mp > 336 °C (decomposition, analytical sample was precipitated from a mixture of DCM/*n*-pentane); IR (KBr): 3082, 3060, 3024, 3000, 2840, 1598, 1582, 1493, 1469, 1447 cm^–1^; ^1^H NMR (500 MHz, CD_2_Cl_2_) δ 7.58 (d, *J* = 8.3 Hz, 2H), 7.10–7.00 (m, 14H), 6.95–6.88 (m, 8H), 6.85–6.75 (m, 20H), 6.71 (s, 4H), 6.24 (d, *J* = 6.9 Hz, 2H), 5.36 (s, 4H, C**H**Ar_2_), 3.66 (s, 6H, OC**H**_3_); ^13^C{^1^H} NMR (50 MHz, CD_2_Cl_2_) δ 190.8 (dd, *J*_C-Ag_ = 251.1, 18.2 Hz, N**C**N), 160.7, 143.8, 142.6, 142.0, 140.2, 140.0, 130.1, 129.8, 129.4, 129.1, 128.6, 127.9, 127.2, 127.0, 124.3, 124.3, 122.3, 115.6, 55.8 (O**C**H_3_), 52.1 (**C**HAr_2_); HR MS (APCI TOF) *m/z* calcd for C_79_H_60_AgN_2_O_2_ [M]^•+^: 1175.3706; found: 1175.3713. ^1^H NMR confirmed the presence of a residual amount of *n*-pentane after rigorous drying under high vacuum overnight.

**Complex 4h** was synthesized according to a modified literature procedure.^33^ A flame-dried Schlenk was charged with salt **7d** (219.5 mg, 0.19 mmol) and Ag_2_O (132.2 mg, 0.57 mmol, 6.0 equiv). Then, anhydrous DCM (10 mL) was added and the resulting suspension was stirred at rt for 16 h. The reaction mixture was filtered through a pad of Celite 545, washing with 10% MeOH/DCM (CAUTION: silver complex **4h** is poorly soluble), and solvents were evaporated. The resulting complex was filtered through a pad of silica (DCM, 10% MeOH/DCM), the solvent was evaporated, and the residue was dissolved in a minimal volume of 10% MeOH/DCM (16 mL) and precipitated with *n*-pentane (24 mL) to give a bright yellow solid (116.0 mg, 48%). mp > 300 °C (decomposition, MeOH/DCM/*n*-pentane); IR (KBr) 3648, 3064, 3018, 2966, 2828, 1730, 1602, 1479, 1459 cm^–1^; ^1^H NMR (400 MHz, CD_2_Cl_2_) δ 7.89 (d, *J* = 8.2 Hz, 2H), 7.55–7.48 (m, 8H), 7.37 (d, *J* = 7.2 Hz, 4H), 7.15–6.91 (m, 14H), 6.86–6.75 (m, 8H), 6.30 (d, *J* = 7.0 Hz, 2H), 5.99 (s, 4H, C**H**Ar_3_), 5.46 (s, 4H, C**H**Ar_3_), 4.21 (s, 6H, OC**H**_3_); ^13^C{^1^H} NMR (100 MHz, CD_2_Cl_2_) δ 151.6, 145.1, 144.5, 144.2, 143.7, 141.3, 137.8, 128.8, 127.4, 126.1, 125.9, 125.7, 125.0, 124.3, 124.1, 123.9, 123.7, 123.3, 116.6, 63.2 (O**C**H_3_), 50.3, 48.3; HR MS (ESI TOF) *m/z* calcd for C_83_H_52_AgN_2_O_2_ [M – Cl]^+^: 1215.3080; found: 1215.3101. Carbene carbon atom has not been recorded due to poor solubility of complex **4h** in CD_2_Cl_2_.

### Synthesis of Gold(I) Complexes

**Complex 8**: A 4 mL screw cap vial was charged with NHC precursor (300.0 mg, 1.1 mmol), AuCl·Me_2_S (312.0 mg, 1.1 mmol, 1.0 equiv), and K_2_CO_3_ (152.0 mg, 1.1 mmol, 1.0 equiv). Then, the vial was transferred to a glovebox, and anhydrous acetone was added (2 mL). The resulting suspension was vigorously stirred at 60 °C for 16 h. Then, the solvent was evaporated, and the residue was filtered through a pad of silica (washing with DCM, 2 × 5 mL). The resulting solution was evaporated and dried under high vacuum to give complex **8** as a white solid (438.7 mg, 92%). ^1^H NMR (400 MHz, CDCl_3_) δ 7.67–7.62 (m, 2H), 7.40–7.34 (m, 2H), 5.51 (sept, *J* = 7.0 Hz, 2H), 1.74 (d, *J* = 7.0 Hz, 12H). The spectral data are in agreement with those reported^55^ (known compound CAS: 953820-59-2).

**Complex 9** was prepared according to the literature procedure.^56^ Gold(I) complex **8** (50.0 mg, 0.12 mmol) and AgOAc (23.4 mg, 0.14 mmol, 1.2 equiv) were placed in a flame-dried Schlenk flask. Then, anhydrous DCM (12 mL) was added and the resulting mixture was stirred for 1 h at rt (Schlenk tube was protected from light by an aluminum foil). The resulting suspension was passed through a pad of Celite 545, and the solvent was evaporated and dried under high vacuum to give a white solid (39.5 mg, 72%). ^1^H NMR (400 MHz, CDCl_3_) δ 7.66–7.58 (m, 2H), 7.38–7.30 (m, 2H), 5.56–5.43 [m, 2H, NC**H**(CH_3_)_2_], 2.10 (s, 3H, C**H**_3_), 1.76 and 1.75 and 1.73 (s, 12H, C**H**_3_). The spectra data are in agreement with those reported.^56^

**Complex 11a**: To a solution of NHC·BF_4_ salt **10a** (83.4 mg, 0.08 mmol) in anhydrous acetone (30 mL), gold(I) complex **9** (37.0 mg, 0.08 mmol, 1.0 equiv) was added and stirred at 80 °C (temp. of aluminum heating block) for 48 h. The solvent was evaporated, dissolved in a minimal volume of DCM, and crashed with *n*-pentane to give complex **11a** as a creamy solid (110.3 mg, 95%). mp 216.0–218.0 °C (DCM/*n*-pentane); ^1^H NMR (600 MHz, CDCl_3_) δ 7.70–7.66 (m, 2H), 7.50–7.46 (m, 2H), 7.19–7.11 (m, 32H), 6.87–6.83 (dd, 8H), 6.68 (s, 4H), 5.49 (s, 2H), 5.34 (s, 4H, C**H**Ar_2_), 4.47 [sept, *J* = 7.0 Hz, 2H, NC**H**(CH_3_)_2_], 3.67 (s, 6H, OC**H**_3_), 1.18 (d, *J* = 7.0 Hz, 12H, C**H**_3_); ^13^C{^1^H} NMR (125 MHz, CDCl_3_) δ 188.4 (**C**^**a**^NCH), 186.4 (**C**^**b**^Nar), 161.2, 143.9, 142.8, 142.6, 133.1, 130.2, 129.4, 129.4, 129.3, 128.1, 128.0, 125.8, 124.8, 115.3, 114.2, 56.2 (O**C**H_3_), 54.5 [**C**H(CH_3_)_2_], 52.8 (**C**HAr_2_), 22.7 (**C**H_3_); ^19^F NMR (376 MHz, CDCl_3_) δ −154.5 (×2); HR MS (ESI TOF) *m/z* calcd for C_82_H_74_AuN_4_O_2_ [M – BF_4_]^+^: 1343.5477; found: 1343.5482.

**Complex 11b**: To a solution of NHC·BF_4_ salt **10b** (46.3 mg, 0.04 mmol) in anhydrous acetone (20 mL), gold(I) complex **9** (20.0 mg, 0.04 mmol, 1.0 equiv) was added and the resulting mixture was heated to 80 °C for 24 h. Then, the solvent was evaporated and the residue was chromatographed on silica (DCM, 5% MeOH/DCM) to give a light brown solid (28.1 mg, 44%). Purification of complex **11b** appeared to be difficult due to decomposition observed. mp > 300 °C (decomposition, DCM/*n*-pentane); ^1^H NMR (600 MHz, CDCl_3_); δ 7.44–7.39 (m, 2H), 7.25–7.19 (m, 2H), 7.04–6.89 (m, 20H), 6.85–6.69 (m, 20H), 6.58 (s, 4H), 5.24 (s, 4H, C**H**Ar_2_), 4.10 [sept, *J* = 7.0 Hz, 2H, NC^b^**H**(CH_3_)_2_], 3.50 (s, 6H, OC**H**_3_), 1.00 (s, 6H, C^a^**H**_3_), 0.86 (d, *J* = 7.0 Hz, 12H, C**H**_3_); ^13^C{^1^H} NMR (125 MHz, CDCl_3_) δ 186.9 (**C**^d^Nar), 185.4 (**C**^c^NCH), 160.8, 144.5, 143.3, 141.2, 133.1, 131.2, 130.4, 130.0, 129.9, 129.4, 129.3, 128.5, 128.1, 127.9, 125.7, 116.6, 115.8, 114.6, 56.3 (O**C**H_3_), 55.1, 52.8, 22.2; ^19^F NMR (376 MHz, CDCl_3_) δ −153.7, −153.8; HR MS (ESI TOF) *m/z* calcd for C_84_H_78_AuN_4_O_2_ [M – BF_4_]^+^: 1371.5790; found: 1371.5786.

**Complex 11c**: To a solution of NHC·BF_4_ salt **10c** (50.5 mg, 0.04 mmol) in anhydrous acetone (10 mL), gold(I) complex **9** (20.0 mg, 0.04 mmol, 1.0 equiv) was added and stirred at 80 °C for 48 h. Then, the solvent was evaporated, and the residue was chromatographed on silica (DCM, 5% MeOH/DCM) to give a yellow solid (65.4 mg, 96%). mp > 300 °C (decomposition, analytical sample was precipitated from a mixture of DCM/*n*-pentane); ^1^H NMR (600 MHz, CDCl_3_) δ 7.69–7.64 (m, 2H), 7.47–7.42 (m, 2H), 7.32 (d, *J* = 8.2, 2H), 7.12 (br d, *J* = 7.4, 8H), 7.02–6.92 (m, 12H), 6.84 (s, 4H), 6.79–6.61 (m, 22H), 5.74 (d, *J* = 6.9 Hz, 2H), 5.60 (s, 4H, C**H**Ar_2_), 4.42 [sept, *J* = 7.0 Hz, 2H, NC**H**(CH_3_)_2_], 3.74 (s, 6H, OC**H**_3_), 1.19 (d, *J* = 7.0 Hz, 12H, C**H**_3_); ^13^C{^1^H} NMR (125 Hz, CDCl_3_) δ 192.5 (**C**^**a**^NCH), 186.8 (**C**^**b**^NAr), 161.0, 144.7, 142.8, 140.8, 140.4, 133.1, 130.0, 129.7, 129.3, 129.0, 128.6, 128.5, 128.2, 127.9, 127.7, 126.8, 125.8, 123.0, 122.7, 115.5, 114.7, 56.3 (O**C**H_3_), 55.3 [NC**H**(CH_3_)_2_], 53.2 (C**H**Ar_2_), 22.3 (**C**H_3_); ^19^F NMR (376 MHz, CDCl_3_) δ −154.5 (×2); HR MS (ESI TOF) *m/z* calcd for C_92_H_78_AuN_4_O_2_ [M – BF_4_]^+^: 1468.5869; found: 1468.5852.

**Complex 11d**: To a solution of NHC·BF_4_ salt **10d** (112.3 mg, 0.09 mmol) in anhydrous acetone (12 mL), gold(I) complex **9** (43.0 mg, 0.09 mmol, 1.0 equiv) was added and stirred for 24 h at 80 °C. Then, DCM (20 mL) was added, and the resulting green solid (impurities) was filtered. Then, the solution was evaporated and dried under high vacuum to give a yellow solid (143.7 mg, 98%). mp > 300 °C (decomposition, analytical sample was precipitated from a mixture of DCM/*n*-pentane); ^1^H NMR (600 MHz, CDCl_3_) δ 8.04 (d, *J* = 8.1 Hz, 2H), 7.54 (d, *J* = 7.3 Hz, 4H), 7.49 (d, *J* = 7.2 Hz, 4H), 7.34–7.21 (m, 10H), 7.08–7.03 (m, 4H), 6.90 (d, *J* = 7.1 Hz, 4H), 6.87–6.82 (m, 4H), 6.81–6.75 (m, 4H), 6.65 (d, *J* = 7.0 Hz, 2H), 6.42 (t, *J* = 7.3, 7.2 Hz, 4H), 6.01 (s, 4H, C**H**Ar_3_), 5.44 (s, 4H, C**H**Ar_3_), 4.26 (s, 6H, OC**H**_3_), 3.70–3.62 [m, 2H, NC**H**(CH_3_)_2_], 0.21 (d, *J* = 6.8 Hz, 12H, C**H**_3_); ^13^C{^1^H} NMR (125 MHz, CDCl_3_) δ 192.1 (**C**^**a**^NCH), 186.5 (**C**^**b**^NAr), 152.4, 145.4, 144.5, 144.3, 142.2, 139.8, 138.7, 132.5, 131.1, 130.7, 130.6, 128.5, 126.9, 126.8, 126.0, 125.6, 125.1, 125.0, 124.9, 124.8, 124.7, 124.6, 123.6, 112.7, 64.2 (O**C**H_3_), 52.3 [**C**H(CH_3_)_2_], 51.2 (C**H**Ar_3_), 48.9 (C**H**Ar_3_), 21.8 (**C**H_3_); ^19^F NMR (376 MHz, CDCl_3_) δ −153.8, −153.9; HR MS (ESI TOF) *m/z* calcd for C_96_H_70_AuN_4_O_2_ [M – BF_4_]^+^: 1507.5164; found: 1507.5171.

### Single-Crystal X-ray Diffraction

The crystals were embedded in the inert perfluoropolyalkylether (viscosity 1800 cSt; ABCR GmbH) and mounted using Hampton Research Cryoloops. The crystals were flash-cooled to 100.0(1) K in a nitrogen gas stream and kept at this temperature during the experiments. The X-ray data were collected on a SuperNova Agilent diffractometer using Mo Kα radiation (λ = 0.71073 Å) or Cu Kα radiation (λ = 1.54184 Å). The data were processed with CrysAlisPro.^57^ Structures were solved by direct methods and refined using SHELXL^58^ under WinGX.^59^ The figures were prepared using X-seed.^60^

#### Crystal Data for **4a**

(C_69_H_56_N_2_O_2_AgCl)·2(CH_2_Cl_2_), *M*_r_ = 1258.3, colorless prisms, orthorhombic, space group **Pbca**, *a* = 19.2621(2), *b* = 24.6506(5), *c* = 25.2385(3) Å, *V* = 11983.8(3) Å^3^, *Z* = 8, ρ_calc_ = 1.39 g cm^–3^, μ(Mo Kα) = 0.61 mm^–1^, θ_max_ = 26.3°, 32 921 reflections measured, 12 236 unique, 760 parameters, *R* = 0.041, *w*R** = 0.093 (*R* = 0.062, *w*R** = 0.106 for all data), GooF = 1.01. CCDC 2143426.

#### Crystal Data for **4b**

(C_69_H_56_N_2_O_2_CuCl)·2(CH_2_Cl_2_), *M*_r_ = 1214.0, colorless prisms, monoclinic, space group **P**21/**n**, *a* = 15.8854(3), *b* = 18.7151(2), *c* = 20.2401(2) Å, β = 94.392(1)°, *V* = 5999.6(1) Å^3^, *Z* = 4, ρ_calc_ = 1.34 g cm^–3^, μ(Mo Kα) = 0.63 mm^–1^, θ_max_ = 26.3°, 47 930 reflections measured, 12 250 unique, 732 parameters, *R* = 0.047, *w*R** = 0.118 (*R* = 0.059, *w*R** = 0.126 for all data), GooF = 1.02. CCDC 2143425.

#### Crystal Data for **4e**

(C_79_H_60_N_2_O_2_CuCl)·2(CH_2_Cl_2_), *M*_r_ = 1338.1, yellow plates, orthorhombic, space group **Pbca**, *a* = 19.0302(2), *b* = 24.2764(2), *c* = 29.9917(4) Å, *V* = 13855.7(3) Å^3^, *Z* = 8, ρ_calc_ = 1.28 g cm^–3^, μ(Cu Kα) = 2.59 mm^–1^, θ_max_ = 66.6°, 79 445 reflections measured, 12 219 unique, 850 parameters, *R* = 0.059, *w*R** = 0.146 (*R* = 0.073, *w*R** = 0.157 for all data), GooF = 1.01. CCDC 2143428.

#### Crystal Data for **4f**

(C_79_H_60_N_2_O_2_AgCl)·3(CH_2_Cl_2_), *M*_r_ = 1467.4, yellow prisms, orthorhombic, space group **Pbca**, *a* = 19.2448(1), *b* = 24.1491(2), *c* = 29.9815(4) Å, *V* = 13933.7(2) Å^3^, *Z* = 8, ρ_calc_ = 1.40 g cm^–3^, μ(Mo Kα) = 0.61 mm^–1^, θ_max_ = 27.5°, 209 158 reflections measured, 15 937 unique, 849 parameters, *R* = 0.041, *w*R** = 0.097 (*R* = 0.055, *w*R** = 0.107 for all data), GooF = 1.08. CCDC 2143427.

### Synthesis of Alkynes

#### 1-Benzofuran-2-carbaldehyde (**S26**)

To a solution of benzofuran (2.3 mL, 21.2 mmol) in anhydrous THF (100 mL), cooled to −78 °C, *n-*BuLi (10.5 mL, 25.4 mmol, 2.5 M in hexane) was added dropwise. After 1 h, anhydrous DMF (3.3 mL, 42.3 mmol, 2.0 equiv) was added dropwise, and the reaction mixture was allowed to warm to rt within 16 h. Then, the reaction mixture was quenched with sat. solution of NH_4_Cl (100 mL), and the aqueous phase was extracted with EtOAc (3 × 20 mL). The combined organic extracts were dried over MgSO_4_ and evaporated. The residue was chromatographed on silica (10% EtOAc/hexane–100% EtOAc, Combi-Flash, 40 g column) to give **S26** as a yellow solid (2.7 g, 87%). ^1^H NMR (200 MHz, CDCl_3_) δ 9.86 (s, 1H), 7.80–7.69 (m, 1H), 7.66–7.44 (m, 3H), 7.40–7.25 (m, 1H). Spectral data are in agreement with those reported.^61^

#### 2-(2,2-Dibromoethyl)-1-benzofuran (**S27**)

The compound was synthesized according to a modified literature procedure.^62^ To a solution of aldehyde **S26** (2.7 g, 18.0 mmol) in anhydrous DCM (50 mL), CBr_4_ (12.0 g, 36.0 mmol, 2.0 equiv) was added in one portion. Then, the reaction mixture was cooled to 0 °C, and PPh_3_ (19.0 g, 72.0 mmol, 4.0 equiv) was added in portions. Then, the reaction mixture was stirred at 0 °C for 2 h and quenched with water (40 mL). The aqueous phase was separated and extracted with DCM (2 × 40 mL). The combined organic extracts were dried over Na_2_SO_4_ and evaporated. The residue was chromatographed on silica (hexane) to give **S27** as a white solid (2.0 g, 37%). ^1^H NMR (400 MHz, CDCl_3_) δ 7.62–7.58 (m, 1H), 7.54–7.52 (m, 1H), 7.48–7.43 (m, 1H), 7.36–7.31 (m, 1H), 7.30–7.28 (m, 1H), 7.27–7.21 (m, 1H). Spectral data are in agreement with those reported.^62^

#### 2-Ethynyl-1-benzofuran (**S12**)

The compound was synthesized according to the modified literature procedure.^62^ To a solution of dibromide **S27** (2.0 g, 6.6 mmol) in anhydrous MeCN (16 mL), DBU (3.9 mL, 26.4 mmol, 4.0 equiv) was added and stirred at rt for 16 h. Then, the reaction mixture was cooled to 15 °C and quenched with 5% HCl (10 mL). After 5 min of vigorous stirring, the aqueous phase was extracted with a mixture of EtOAc/hexane (2 × 50 mL, EtOAc/hexane = 1/1, v/v). The combined organic extracts were washed with water (1 × 100 mL), dried over Na_2_SO_4_, and evaporated. The residue was chromatographed on silica (hexane, Combi-Flash, 40 g column) to give alkyne **S12** as a brown oil (211.0 mg, 22%). ^1^H NMR (400 MHz, CDCl_3_) δ 7.60–7.53 (m, 1H), 7.49–7.43 (m, 1H), 7.38–7.32 (m, 1H), 7.28–7.22 (m, 1H), 7.02–7.00 (m, 1H), 3.49 (s, 1H); ^13^C{^1^H} NMR (100 MHz, CDCl_3_) δ 154.8, 137.7, 127.2, 125.9, 123.4, 121.4, 112.6, 111.4, 83.6, 74.1. Spectral data are in agreement with those reported.^62^

#### 1-Methyl-3-{[tri(propan-2-yl)silylo]ethnyl}c-*1H*-indol (**S29**)

The compound was synthesized according to the modified literature procedure.^63^ A 24 mL stainless ball milling vessel was charged with iodonium salt **S28** (925.3 mg, 2.16 mmol, 1.2 equiv), AuCl (8.4 mg, 0.036 mmol, 2 mol %), 1-methylindole (236 μL, 1.80 mmol), and grinding balls (five stainless still balls, diameter 9 mm). The ball milling vessel was placed in a Retsch PM100 ball mill (500 rpm, 99 min). The crude reaction mixture was dissolved in Et_2_O (20 mL) and diluted with water (50 mL). The aqueous phase was extracted with Et_2_O (2 × 20 mL), and the combined ethereal extracts were washed with NaOH (0.1 M, 2 × 50 mL), sat. soln of citric acid (1 × 50 mL), brine (1 × 50 mL), dried over MgSO_4_, and evaporated. The residue was chromatographed on silica (30% EtOAc/hexane) to give **S29** as a green solid (416.0 mg, 93%). ^1^H NMR (400 MHz, CDCl_3_) δ 7.73 (br d, *J* = 7.7 Hz, 1H), 7.35–7.23 (m, 3H), 7.23–7.16 (m, 1H), 3.77 (s, 3H), 1.17 (br s, 18H). Spectral data are in agreement with those reported.^63^

#### 3-Ethynyl-1-methyl-1*H*-indole (**S13**)

A solution of indole derivatives **S29** (295.9 mg, 0.95 mmol) was dissolved in anhydrous DCM (1.0 mL), and Bu_4_NF in THF (0.95 mL, 0.95 mmol, 1.0 equiv, 1.0 M in THF) was added. The reaction mixture was stirred at rt for 4 h, and an additional portion of Bu_4_NF in THF (0.95 mL, 0.95 mmol) was added, and the reaction mixture was left for 16 h with stirring at rt. Then, solvents were evaporated, and the residue was chromatographed on silica (1–2% EtOAc/hexane) to give alkyne **S13** as a green oil (112.1 mg, 76%). ^1^H NMR (400 MHz, CDCl_3_) δ 7.76–7.72 (m, 1H), 7.34–7.24 (m, 3H), 7.23–7.18 (m, 1H), 3.78 (s, 3H), 3.21 (s, C≡C–H, 1H). Spectral data are in agreement with those reported.^64^

#### 1-Benzoylpiperidine-4-carboxylic Acid (**S31**)

The compound was synthesized according to a slightly modified literature procedure.^65^ A solution of isonipecotic acid (**S30**) (3.23 g, 25.0 mmol) was added to a mixture of THF (25 mL) and water (25 mL), and K_2_CO_3_ (10.4 g, 75.0 mmol, 3.0 equiv) was added and cooled to 0 °C. Then, BzCl (2.9 mL, 25.0 mmol, 1 equiv) was added dropwise, and the cooling bath was removed and stirred overnight at rt. The reaction mixture was acidified with 5% HCl_aq_ (up to pH = 1–2), saturated with solid NaCl, and extracted with EtOAc (4 × 50 mL). The combined organic phases were dried with Na_2_SO_4_ and concentrated. The residue was treated with hexane to precipitate pure acid **S31** as a white solid (4.14 g, 71%). ^1^H NMR (400 MHz, CDCl_3_) δ 11.09 (bs, 1H, CO_2_**H**), 7.43–7.35 (m, 5H, Ar**H**), 4.50 (bs, 1H), 3.74 (bs, 1H), 3.15–3.01 (m, 2H), 2.65–2.56 (m, 1H), 2.12–1.83 (m, 2H), 1.74 (bs, 2H). Spectral data are in agreement with those reported.^66^

#### 4,5,6,7-Tetrachloro-1,3-dioxoisoindolin-2-yl 1-benzoylpiperidine-4-carboxylate (**S33**)

The compound was synthesized according to a slightly modified literature procedure.^67^ To a vigorously stirred suspension of 1-benzoylpiperidine-4-carboxylic acid (**S31**) (1.50 g, 6.43 mmol, 1.0 equiv), hydroxyimidate **S32** (1.94 g, 6.43 mmol, 1.0 equiv), and DMAP (78.6 mg, 0.64 mmol, 10 mol %) in DCM (60 mL), DIC (1.1 mL, 7.07 mmol, 1.2 equiv) was added dropwise and stirred for 17 h. The mixture was concentrated, filtered, and the solid was washed with DCM. The combined filtrates were concentrated, and the solid was precipitated using *n*-pentane to give a pale yellow solid (3.32 g, 71%). This amide was used without further purification in the next step (purification by column chromatography on silica has failed; decomposition was observed). ^1^H NMR (400 MHz, CDCl_3_) δ 7.44–7.37 (m, 5H), 4.43 (bs, 1H), 3.83 (bs, 1H), 3.31–3.22 (m, 2H), 3.11–3.01 (m, 1H), 2.29–1.81 (m, 4H). Spectral data are in agreement with those reported.^67^

#### 1-Benzoyl-4-ethynylpiperidine (**S7**)

The compound was prepared according to the literature procedure.^67^ A round-bottom flask was charged with NiCl_2_·6H_2_O (188.3 mg 0.79 mmol, 20 mol %) and 4,4′-dimethoxy-2-2′-bipyridine (171.3 mg, 0.79 mmol, 20 mol %), and dry DMF (20 mL) was added and stirred till the mixture became a homogeneous green solution. In another flask was prepared 1.0 M ZnCl_2_/LiCl in THF by dissolving ZnCl_2_ (1.35 g, 9.90 mmol, 2.5 equiv) and LiCl (420 mg, 9.90 mmol, 2.5 equiv) in 10 mL of THF. After cooling to rt, ethynylmagnesium bromide (19.8 mL, 9.90 mmol, 2.5 equiv, 0.5 M THF solution) was added dropwise to a flask containing (4-MeOByPy)·NiCl_2_ complex, and the resulting solution was stirred at rt for 30 min (until it became homogeneous).

Another flask, charged with ester **S33** (2.04 g, 3.96 mmol), was evacuated and backfilled with argon. Then, the premixed nickel/ligand and ethynyl zinc chloride solution were added in succession. After stirring at rt for 15 h, 1.0 M HCl_aq_ (40 mL) and Et_2_O (50 mL) were added. The layers were separated, and the aqueous layer was further extracted with Et_2_O (50 mL), AcOEt (2 × 50 mL), and DCM (2 × 50 mL). The combined organic extracts were washed with brine (1 × 100 mL), dried with Na_2_SO_4_, and evaporated. The crude product was chromatographed on silica (10–50% EtOAc/hexane), and the resulting solid was treated with DCM (10 mL) and stirred for 1 h at rt. The solid impurities were filtered and washed with DCM (1 × 5 mL). The filtrates were evaporated to give a white solid (0.55 g, 65%). ^1^H NMR (400 MHz, DMSO-*d*_6_) δ 7.57–7.23 (m, 5H), 3.95 (bs, 1H), 3.46 (bs, 1H), 3.34–3.07 (m, 2H), 2.98 (s 1H), 2.70 (s, 1H), 1.79 (bs, 2H), 1.50 (br s, 2H). Spectral data are in agreement with those reported.^68^

#### 6-Chloro-1-cyclopropyl-7-fluoro-4-oxo-1,4-dihydroquinoline-3-carbonyl Chloride (**S35**)

To a suspension of 6-chloro-1-cyclopropyl-7-fluoro-4-oxo-1,4-dihydroquinoline-3-carboxylic acid (**S34**) (2.25 g, 8.0 mmol) in toluene (20 mL), (COCl)_2_ (1.0 mL, 12.0 mmol, 1.5 equiv) and catalytic amount of DMF (2 drops) were added. The resulting reaction mixture was heated at 40 °C for 3 h. The reaction mixture was cooled to rt, and the resulting solid was filtered, washed with toluene (2 × 10 mL), and dried in vacuo to give acid chloride **S35** as a light yellow solid (2.39 g). Acid chloride **S35** was used in the next step without further purification.

#### Hept-6-yn-1-yl 6-chloro-1-cyclopropyl-7-fluoro-4-oxo-1,4-dihydroquinoline-3-carboxylate (**S15**)

To a solution of hept-6-yne-1-ol (1.1 g, 9.6 mmol, 1.2 equiv) and Et_3_N (1.3 mL, 9.6 mmol, 1.2 equiv) in DCM (20 mL), cooled to 0 °C, acid chloride **S35** (2.4 g, 8.0 mmol) was added in portions. Then, the reaction mixture was stirred for 16 h at rt and diluted with water. The aqueous phase was extracted with DCM (2 × 20 mL), dried over Na_2_SO_4_, and evaporated. The residue was chromatographed on silica (5% EtOAc/DCM) to give a light yellow solid, which was further purified by crystallization from mixture benzene/*n*-heptane (1.65 g). ^1^H NMR indicated some impurities, and ester **S15** was chromatographed on silica (1% MeOH/DCM) to give pure **S15** as a white solid (1.53 g, 51%). mp 150–153 °C (*n*-heptane/DCM); ^1^H NMR (400 MHz, CDCl_3_) δ 8.54–8.47 (m, 1H), 8.13 (dd, *J* = 9.1, 4.8 Hz, 2H), 7.97 d, (*J* = 5.9 Hz, 1H), 4.30 (t, *J* = 6.8 Hz, 2H, C**H**_2_O), 3.51–3.39 [m, 1H, NC**H**(CH_2_)], 2.27–2.14 (m, 2H, C**H**_2_), 1.95–1.89 (m, 1H, CH_2_CC**H**), 1.84–1.72 (m, 2H, C**H**_2_), 1.66–1.48 (m, 4H, C**H**_2_), 1.41–1.30 (m, 2H, C**H**_2_), 1.19–1.09 (m, 2H, C**H**_2_); ^13^C{^1^H} NMR (100 MHz, CDCl_3_) δ 172.7, 165.2, 155.7 (d, *J*_CF_ = 249.2 Hz), 148.9, 137.3 (d, *J*_CF_ = 2.1 Hz), 128.7 (d, *J*_CF_ = 5.8 Hz), 127.0 (d, *J*_CF_ = 20.2 Hz), 119.1, 113.9 (d, *J*_CF_ = 22.7 Hz), 110.8, 84.4, 68.5, 65.0, 34.9, 28.3, 28.2, 25.2, 18.4, 8.4; ^19^F NMR (376 MHz, CDCl_3_) δ −118.0. HR MS (ESI TOF) *m*/*z* calcd for C_20_H_19_ClFNO_3_Na [M + Na]^+^: 398.0935; found: 398.0927.

#### Hex-5-ynoyl Chloride (**S37**)

To a solution of hex-5-ynoic acid (**S36**) (2.0 mL, 18.2 mmol) in THF (20 mL), oxalyl chloride (2.4 mL, 27.2 mmol) and one drop of DMF were added. The resulting solution was stirred for 2 h at rt. Then, the solvent and excess of oxalyl chloride were evaporated, and the crude product was twice evaporated with DCM (2 × 5 mL) to give acid chloride **S37**, which was used in the next step without further purification.

#### *N*-{4-[(5-Methyl-1,2-oxazol-3-yl)sulfamoyl]phenyl}hex-5-ynamide (**S39**)

To a precooled (−40 °C) solution of 4-amino-*N*-(5-methyl-1,2-oxazol-3-yl)benzenesulfonamide **S38** (3.5 g, 14.0 mmol, 1.0 equiv) in anhydrous pyridine (40 mL), a solution of crude 5-pentynoic chloride (18.2 mmol, 1.3 equiv) in DCM (12 mL) was added dropwise. The cooling bath was removed, and the suspension was stirred for 18.5 h at rt. The reaction mixture was diluted with water (100 mL) and extracted with DCM (3 × 40 mL). The combined organic phases were washed with 5% HCl_aq_ (3 × 100 mL) and sat. NaHCO_3aq_ (1 × 100 mL, NaHCO_3_ appeared to be a strong enough base to deprotonate sulfonamide). The aqueous phase was washed with DCM (1 × 40 mL, organic phase was disposed of) and acidified with 10% HCl_aq_. The precipitated solid was washed with water (1 × 50 mL) and DCM (1 × 20 mL). The aqueous solution was washed with DCM (4 × 40 mL). The combined organic phases were dried with Na_2_SO_4_ and evaporated. The crude product and that described above were combined and boiled with EtOAc giving a white solid (1.66 g, 34%). mp 204.0 °C (decomposition, AcOEt); ^1^H NMR (400 MHz, CD_3_OD) δ 7.84–7.79 (m, 2H), 7.75–7.71 (m, 2H), 6.10 (s, 1H), 2.53 (t, *J* = 7.5 Hz, C**H**_2_), 2.30 (s, 3H, C**H**_3_) overlapping 2.29–2.24 (m, 3H, C**H**_2_ and CC**H**), 1.92–1.83 (m, 2H, C**H**_2_); ^13^C{^1^H} NMR (100 MHz, CD_3_OD) 174.1, 171.9, 159.4, 144.6, 135.3, 129.3, 120.4, 96.5, 84.1, 70.3, 36.6, 25.3, 18.6, 12.2. HR MS (ESI TOF) *m*/*z* calcd for C_16_H_17_N_3_O_4_SNa [M + Na]^+^: 370.0837; found: 370.0835.

#### *N*-{4-[Methyl(5-methyl-1,2-oxazol-3-yl)sulfamoyl]phenyl}hex-5-ynamide (**S17**)

To the stirred solution of alkyne (**S39**) (1.0 g, 3.0 mmol) in MeCN (20 mL), K_2_CO_3_ (1.24 g, 9.0 mmol, 3.0 equiv), MeI (1.9 mL, 30.0 mmol, 10.0 equiv), and Bu_4_N^+^I^–^ (110.8 mg, 0.3 mmol, 10 mol %) were added. The suspension was stirred for 21 h at rt. The solvent was evaporated, and the residue was partitioned between water (10 mL) and DCM (30 mL). The aqueous phase was separated and extracted with DCM (4 × 30 mL). The combined organic phases were dried with Na_2_SO_4_, evaporated, and the residue was chromatographed on silica (1% MeOH/DCM) to give a white solid (1.08 g, 90%). mp 130–131 °C (*n*-heptane/DCM); ^1^H NMR (400 MHz, CD_3_OD) δ 7.76 (d, *J* = 8.6 Hz, 2H), 7.69 (d, *J* = 8.5 Hz, 2H), 6.49 (s, 1H), 3.21 (s, 3H, C**H**_3_), 2.52 (t, *J* = 7.4 Hz, 2H, C**H**_2_), 2.36 (s, 3H, C**H**_3_), 2.31–2.20 (m 3H, C**H**_2_, CC**H**), 1.93–1.81 (m, 2H, C**H**_3_); ^13^C{^1^H} NMR (100 MHz, CD_3_OD) δ 174.1, 172.2, 162.2, 145.2, 132.0, 129.5, 120.5, 98.4, 84.1, 70.3, 36.6, 35.6, 25.3, 18.6, 12.3. HR MS (ESI TOF) *m*/*z* calcd for C_17_H_19_N_3_O_4_SNa [M + Na]^+^: 384.0994; found: 384.0989.

#### *tert*-Butyl(dimethyl)silyl (4*E*)-6-(4-{[*tert*-butyl(dimethyl)silyl]oxy}-6-methoxy-7-methyl-3-oxo-1,3-dihydro-2-benzofuran-5-yl)-4-methylhex-4-enoate (**S41**)

The compound was synthesized according to the modified literature procedure.^69^ To the solution of mycophenolic acid (**S40**) (2.0 g, 12.5 mmol) and TBSCl (11.3 g, 74.9 mmol) in dry DMF (20 mL), imidazole (6.8 g, 99.9 mmol) was added portionwise, and the reaction mixture was stirred at rt for 2.5 h. Then, the solution was cooled to 0 °C, and water (60 mL) was slowly added followed by Et_2_O (120 mL). The ethereal phase was separated and washed with water (5 × 40 mL). Every aqueous phase was washed with the same portion of Et_2_O (1 × 50 mL). The combined organic phases were dried over Na_2_SO_4_, and the solvent was evaporated. Crude **S41** was used in the next step without further purification.

#### (4*E*)-6-(4-{[*tert*-Butyl(dimethyl)silyl]oxy}-6-methoxy-7-methyl-3-oxo-1,3-dihydro-2-benzofuran-5-yl)-4-methylhex-4-enoic Acid (**S42**)

Silyl ether **S41** was dissolved in THF (20 mL), and water (20 mL) and AcOH (20 mL) were added. The resulting solution was stirred at rt for 1.5 h. Then, the reaction mixture was diluted with water (60 mL) and Et_2_O (120 mL), and the organic phase was separated and washed with water (5 × 40 mL). Every aqueous phase was extracted with the same portion of Et_2_O (1 × 50 mL). The combined organic phases were dried over Na_2_SO_4_, and the solvents were evaporated. The residue was purified by chromatography on silica (DCM, 3% MeOH/DCM) to give a white powder (5.55 g, ∼100%). ^1^H NMR (400 MHz, CDCl_3_) δ 5.20 (t, *J* = 5.2 Hz, 1H, C=CH), 5.06 (s, 2H, OCH_2_Ar), 3.73 (s, 3H, ArOCH_3_), 3.38 (d, *J* = 6.4 Hz, 2H, ArCH_2_CH=C), 2.44–2.37 (m, 2H, CH_2_), 2.32–2.25 (m, 2H, CH_2_), 2.14 (s, 3H, ArCH_3_), 1.75 (s, 3H, C=CCH_3_), 1.02 [s, 9H, SiC(CH_3_)_3_], 0.23 (s, 6H, Si(CH_3_)_2_]. Spectral data are in agreement with those reported.^69^

#### Hex-5-yn-1-yl (4*E*)-6-(4-{[*tert*-butyl(dimethyl)silyl]oxy}-6-methoxy-7-methyl-3-oxo-1,3-dihydro-2-benzofuran-5-yl)-4-methylhex-4-enoate (**S18**)

To the stirred solution of silyl ether **S42** (2.0 g, 4.6 mmol) in anhydrous DCM (40 mL), 5-hexyn-1-ol (0.6 mL, 5.5 mmol, 1.2 equiv) was added. The resulting solution was cooled to 0 °C, EDC·HCl (1.1 g, 5.5 mmol, 1.2 equiv) and DMAP (67.3 mg, 0.55 mmol, 12 mol %) were added, followed by Et_3_N (1.4 mL, 10.1 mmol, 2.2 equiv). After 1.5 h, the cooling bath was removed, and the mixture was stirred for 15 h at rt. Then, water (25 mL) and sat. soln. of NH_4_Cl_aq_ (50 mL) were added, and the phases were separated. The aqueous phase was washed with DCM (3 × 40 mL), and the combined organic phases were dried over Na_2_SO_4_ and evaporated. The residue was chromatographed on silica (2–3% EtOAc/toluene) to give **S18** as a colorless oil (1.27 g, 54%). ^1^H NMR (400 MHz, CDCl_3_) δ 5.17 (t, *J* = 5.8 Hz, 1H, C=CH), 5.05 (s, 2H, OCH_2_Ar), 4.03 (t, *J* = 6.5 Hz, 2H, OCH_2_CH_2_), 3.73 (s, 3H, ArOCH_3_), 3.37 (d, *J* = 6.4 Hz, 2H, ArCH_2_CH=C), 2.40–2.32 (m, 2H, CH_2_), 2.31–2.24 (m, 2H, CH_2_), 2.19 (td, *J* = 7.0, 2.7 Hz, 2H, CH_2_), 2.14 (s, 3H, ArCH_3_), 1.93 (t, *J* = 2.7 Hz, 1H, CH_2_CCH), 1.77–1.65, m, 2H, CH_2_) overlapping 1.74 (s, 3H, C=CCH_3_), 1.60–1.50, m, 2H, CH_2_), 1.02 [s, 9H, SiC(CH_3_)_3_], 0.23 (s, 6H, Si(CH_3_)_2_]; ^13^C{^1^H} NMR (100 MHz, CDCl_3_) δ 173.4, 169.3, 163.3, 151.9, 146.2, 133.8, 127.8, 123.7, 118.0, 111.8, 84.0, 68.8, 67.7, 63.9, 60.8, 34.6, 33.2, 27.8, 26.2, 25.1, 23.8, 18.9, 18.2, 16.5, 11.5, −3.4. HR MS (ESI TOF) *m*/*z* calcd for C_29_H_42_O_6_SiNa [M + Na]^+^: 537.2648; found: 537.2646.

#### Hex-5-yn-1-yl 5-[(3*aS*,4*S*,6*aR*)-2-oxohexahydro-1*H*-thieno[3,4-*d*]imidazol-4-yl]pentanoate (**S16**)

To a suspension of biotin (500 mg, 2.21 mmol) in anhydrous DMF (20 mL), HOBt·H_2_O (338.4 mg, 3.32 mmol, 1.5 equiv) was added in one portion at rt. After 15 min, EDC·HCl (635.5 mg, 3.32 mmol, 1.5 equiv) was added, and the reaction mixture was stirred for an additional 15 min (all solids have been dissolved). Then, alcohol (426.0 μL, 433.8 mg, 4.42 mmol) and DMAP (540.0 mg, 4.42 mmol, 2.0 equiv) were added and stirred for 16 h at rt. Then, the reaction mixture was diluted with water (100 mL) and brine (100 mL) and extracted with EtOAc (3 × 30 mL). The combined organic extracts were washed with brine (2 × 50 mL), dried over Na_2_SO_4_, and evaporated. The residue was chromatographed on silica (EtOAc/5% MeOH/EtOAc) to give an ester **S16** as a waxy solid (611.5 mg, 90%). [*a*]_D_^29^ = 49.5 (*c* = 1.0, DCM); ^1^H NMR 50 MHz, CDCl_3_) δ 6.18 (br s, 2H), 4.56–4.41 (m, 1H), 4.35–4.21 (m, 1H), 4.13–3.98 (m, 2H), 3.22–3.04 (m, 1H), 2.97–2.62 (m, 3H), 2.41–2.12 (m, 4H), 1.98–1.92 (m, 1H), 1.85–1.28 (m, 11H); ^13^C{^1^H} NMR (50 MHz, CDCl_3_) δ 173.7, 164.1–163.7 (m), 83.8, 68.7, 63.7, 62.0, 60.1, 55.4, 40.4, 33.8, 28.3, 28.1, 27.6, 24.8, 24.7, 18.0. HR MS (EI EBE double focusing geometry mass analyzer) *m/z* calcd C_16_H_24_N_2_O_3_SNa [M + Na]^+^: 347.1405; found: 347.1403.

### Synthesis of Fluorinated Naphthyridines

#### General Procedure 1 (**GP1**)

A 4 mL screw cap vial was charged with aminophenone **1a**,**b** (0.5 mmol), alkyne (0.6 mmol, 1.2 equiv), and complex **4a–h** (0.005 mmol, 2 mol %). Then, the solution of TMG (*N*,*N*,*N***′**,*N***′**-tetramethylguanidine, 1.25 μL, 2 mol %) in degassed water (2 mL) was added. The resulting biphasic mixture was stirred at 120 °C in an aluminum heating block (thermocouple was inserted in a separate vial filled with silicon oil M350) for the indicated time (usually 19 h) with vigorous stirring. Then, the reaction mixture was diluted with brine and extracted with EtOAc (3×) or DCM (3×). The combined organic extracts were dried over Na_2_SO_4_, evaporated, and the residue was chromatographed on silica (unless indicated otherwise) using an appropriate eluting system to afford the product.

#### 2-Cyclopropyl-4-(trifluoromethyl)-1,8-naphthyridine (**3a**)

The title compound was obtained according to GP1 using aminophenone **1a** (570.4 mg, 3.0 mmol), alkyne **2a** (280.0 μL, 3.6 mmol, 1.2 equiv), complex **4h** (62.7 mg, 0.1 mmol, 2 mol %), TMG (7.5 μL, 0.1 mmol, 2 mol %), and water (10 mL). The resulting reaction mixture was heated at 120 °C for 16 h. Then, the reaction mixture was diluted with EtOAc (5 mL), and the aqueous phase was separated and extracted with EtOAc (3 × 5 mL). The combined organic extracts were washed with brine (1 × 5 mL), dried over Na_2_SO_4_, and evaporated. The residue was chromatographed on silica (20% EtOAc/hexane, Combi-Flash, 40 g column) to give **3a** as a yellow solid (127.9 mg, 18%). ^1^H NMR (400 MHz, CDCl_3_) δ 9.10 (dd, *J* = 4.2, 1.8 Hz, 1H), 8.44–8.37 (m, 1H), 7.68 (s, 1H), 7.49 (dd, *J* = 8.2, 4.2 Hz, 1H), 2.35–2.22 (m, 1H), 1.52–1.36 (m, 2H), 1.28–1.12 (m, 2H). Spectral data are in agreement with those reported.^23c^ For details on optimization studies, see Table S1 in the SI.

#### 2-Phenyl-4-(trifluoromethyl)-1,8-naphthyridine (**3b**)

The compound was obtained according to GP1 using aminopyridine **1a** (95.0 mg, 0.50 mmol, 1.0 equiv), alkyne **S1** (66.0 μL, 0.60 mmol, 1.2 equiv), copper(I) complex **4h** (12.1 mg, 0.01 mmol, 2 mol %), and TMG (1.25 μL, 0.01 mmol, 2 mol %) in water (2 mL). Then, the reaction mixture was diluted with brine (2 mL) and extracted with EtOAc (3 × 1 mL). The residue was chromatographed on silica (15% EtOAc/hexane) to give naphthyridine **3b** as an orange solid (113.3 mg, 83%). To test the remarkable effect of the NHC ligand on the course of direct catalytic alkynylation/dehydrative cyclization, a polymeric (PhC≡C–Cu)*_n_* (generated prior to use) was reacted with aminophenone **1a** on water in the presence of TMG (or without an external base) at 120 °C. Unfortunately, the formation of product **3a** has not been detected. mp 154.0–155.0 °C (*n*-heptane); ^1^H NMR (400 MHz, CDCl_3_) δ 9.26–9.18 (m, 1H), 8.55–8.46 (m, 1H), 8.37–8.27 (m, 3H), 7.64–7.49 (m, 4H); ^13^C{^1^H} NMR (100 MHz, CDCl_3_) δ 159.8, 156.4, 154.5, 137.3, 136.2 (q, *J*_CF_ = 31.9 Hz), 133.3 (q, *J*_CF_ = 2.0 Hz), 130.8, 129.0, 127.8, 123.0 (q, *J*_CF_ = 273.3 Hz), 122.8, 116.9, 116.7 (q, *J*_CF_ = 5.1 Hz); ^19^F NMR (376 MHz, CDCl_3_) δ −60.9; HR MS (EI EBE double focusing geometry mass analyzer) *m/z* calcd C_15_H_9_F_3_N_2_ [M]^•+^: 274.0718; found: 274.0714.

#### 2-(4-Methylphenyl)-4-(trifluoromethyl)-1,8-naphthyridine (**3c**)

The compound was obtained according to GP1 using aminopyridine **1a** (95.0 mg, 0.50 mmol), alkyne **S2** (76.1 μL, 0.60 mmol, 1.2 equiv), copper(I) complex **4h** (12.1 mg, 0.01 mmol, 2 mol %), and a solution of TMG (1.25 μL, 0.01 mmol, 2 mol %) in water (2 mL). The reaction mixture was heated at 120 °C for 19 h. Then, the reaction mixture was extracted with EtOAc (3 × 1 mL), and the combined organic extracts were dried over Na_2_SO_4_, filtered, and evaporated. The residue was chromatographed on silica (1% acetone/DCM) to afford naphthyridine **3c** as an orange solid (132.5 mg, 92%). mp 74.0–175.0 °C (*n*-heptane); ^1^H NMR (400 MHz, CDCl_3_) δ 9.21–9.17 (m, 1H), 8.50–8.44 (m, 1H), 8.28–8.19 (m, 3H), 7.59–7.52 (m, 1H), 7.38–7.32 (m, 2H), 2.43 (s, 3H, C**H**_3_); ^13^C{^1^H} NMR (100 MHz, CDCl_3_) δ 159.8, 156.5, 154.4, 141.4, 136.1 (q, *J*_CF_ = 31.6 Hz), 134.6, 133.3 (q, *J*_CF_ = 2.0 Hz), 129.8, 127.8, 123.1 (q, *J*_CF_ = 273.2 Hz), 122.6, 116.8, 116.6 (q, *J*_CF_ = 5.1 Hz), 21.4; ^19^F NMR (376 MHz, CDCl_3_) δ −61.0; HR MS (EI EBE double focusing geometry mass analyzer) *m/z* calcd for C_16_H_11_F_3_N_2_ [M]^•+^: 288.0874; found: 288.0871.

#### 2-(4-Methoxyphenyl)-4-(trifluoromethyl)-1,8-naphthyridine (**3d**)

The compound was obtained according to GP1 using aminopyridine **1a** (95.0 mg, 0.50 mmol), alkyne **S3** (77.8 μL, 0.60 mmol, 1.2 equiv), copper(I) complex **4h** (12.1 mg, 0.01 mmol, 2 mol %), and solution of TMG (1.25 μL, 0.01 mmol, 2 mol %) in water (2 mL). The biphasic reaction mixture was heated at 120 °C for 19 h. Then, the reaction mixture was extracted with EtOAc (4 × 1 mL), dried over Na_2_SO_4_, and evaporated. The residue was chromatographed on silica (20% EtOAc/hexane) to give naphthyridine **3d** as a yellow solid (98.8 mg, 65%). mp 138.0–139.0 °C (*n*-heptane); ^1^H NMR (400 MHz, CDCl_3_) δ 9.18–9.13 (m, 1H), 8.47–8.40 (m, 1H), 8.32–8.24 (m, 2H), 8.21 (br s, 1H), 7.55–7.49 (m, 1H), 7.06–6.99 (m, 2H), 3.87 (s, 3H, OC**H**_3_); ^13^C{^1^H} NMR (100 MHz, CDCl_3_) δ 162.1, 159.3, 156.6, 154.4, 136.0 (q, *J*_CF_ = 31.7 Hz), 133.2 (q, *J*_CF_ = 2.1 Hz), 129.8, 129.5, 123.1 (q, *J*_CF_ = 273.3 Hz), 122.3, 116.5, 116.2 (q, *J*_CF_ = 5.1 Hz), 114.4, 55.4; ^19^F NMR (376 MHz, CDCl_3_) δ −61.0; HR MS (EI EBE double focusing geometry mass analyzer) *m/z* calcd for C_16_H_11_F_3_N_2_O [M]^•+^: 304.0823; found: 304.0827.

#### 2-(4-Fluorophenyl)-4-(trifluoromethyl)-1,8-naphthyridine (**3e**)

The compound was obtained according to GP1 using aminopyridine **1a** (95.0 mg, 0.50 mmol), alkyne **S4** (68.7 μL, 0.60 mmol, 1.2 equiv), copper(I) complex **4h** (12.1 mg, 0.01 mmol, 2 mol %), and a solution of TMG (1.25 μL, 0.01 mmol, 2 mol %) in water (2 mL). The reaction mixture was heated at 120 °C for 19 h. Then, the reaction mixture was diluted with brine (3 mL) and extracted with EtOAc (3 × 1 mL), and the combined organic extracts were dried over Na_2_SO_4_ and evaporated. The residue was chromatographed on silica (30% MTBE/hexane) to give naphthyridine **3e** as a light brown solid (126.8 mg, 87%). mp 173.0–174.0 °C (*n*-heptane); ^1^H NMR (400 MHz, CDCl_3_) δ 9.29–9.18 (m, 1H), 8.57–8.48 (m, 1H), 8.42–8.30 (m, 2H), 8.27 (br s, 1H), 7.67–7.57 (m, 1H), 7.32–7.20 (m, 2H); ^13^C{^1^H} NMR (100 MHz, CDCl_3_) δ 168.0 (d, *J* = 250.0 Hz), 158.7, 156.4, 154.7, 136.5 (q, *J*_CF_ = 31.5 Hz), 133.5 (d, *J*_CF_ = 3.1 Hz), 133.3 (q, *J*_CF_ = 2.1 Hz), 130.0 (d, *J*_CF_ = 8.7 Hz), 123.0 (q, *J*_CF_ = 273.3 Hz), 122.9, 116.9, 116.4 (q, *J*_CF_ = 4.9 Hz), 116.1 (d, *J*_CF_ = 21.7 Hz); ^19^F NMR (376 MHz, CDCl_3_) δ −61.0, −109.5; HR MS (EI EBE double focusing geometry mass analyzer) m/z calcd for C_15_H_8_F_4_N_2_ [M]^•+^: 292.0624; found: 292.0626.

#### 4-(Trifluoromethyl)-2-[4-(trifluoromethyl)phenyl]-1,8-naphthyridine (**3f**)

The compound was obtained according to GP1 using aminopyridine **1a** (95.0 mg, 0.50 mmol), alkyne **S5** (97.8 μL, 0.60 mmol, 1.2 equiv), copper(I) complex **4h** (12.1 mg, 0.01 mmol, 2 mol %), and a solution of TMG (1.25 μL, 0.01 mmol, 2 mol %) in water (2 mL). The reaction mixture was heated at 120 °C for 19 h. Then, the reaction mixture was diluted with brine (3 mL) and extracted with EtOAc (4 × 1 mL). The combined organic extracts were dried over Na_2_SO_4_ and evaporated. The residue was chromatographed on aluminum oxide (10% EtOAc/hexane, Brockmann activity I) to give naphthyridine **3f** as a white solid (138.7 mg, 81%). mp 221.0–222.0 °C (precipitated from a mixture of *n*-pentane/DCM); ^1^H NMR (400 MHz, CDCl_3_) δ 9.27–9.23 (m, 1H), 8.56–8.49 (m, 1H), 8.43 (br d, *J* = 8.2 Hz, 2H), 8.29 (s, 1H), 7.80 (br d, *J* = 8.3 Hz, 2H), 7.66–7.60 (m, 1H); ^13^C{^1^H} NMR (100 MHz, CDCl_3_) δ 158.3, 156.4, 155.0, 140.5, 136.8 (q, *J*_CF_ = 32.0 Hz), 133.4 (q, *J*_CF_ = 2.2 Hz), 132.5 (q, *J*_CF_ = 32.5 Hz), 128.2, 126.0 (q, *J*_CF_ = 31.8 Hz), 123.9 (q, *J*_CF_ = 270.8 Hz), 122.9 (q, *J*_CF_ = 273.5 Hz), 123.4, 117.4, 116.6 (q, *J*_CF_ = 5.1 Hz); ^19^F NMR (376 MHz, CDCl_3_) δ −60.9, −62.9; HR MS (EI EBE double focusing geometry mass analyzer) *m/z* calcd for C_16_H_8_F_6_N_2_ [M]^•+^: 342.0592; found: 342.0599.

#### *N*-[(1*S*)-1-Phenylethyl]-4-[4-(trifluoromethyl)-1,8-naphthyridin-2-yl]butanamide (**3g**)

The compound was obtained according to GP1 using aminopyridine **1a** (95.0 mg, 0.50 mmol), alkyne **S6** (129.2 mg, 0.60 mmol, 1.2 equiv), copper(I) complex **4h** (12.1 mg, 0.01 mmol, 2 mol %), and a solution of TMG (1.25 μL, 0.01 mmol, 2 mol %) in water (2 mL). Then, the reaction mixture was heated at 120 °C for 19 h and extracted with EtOAc (4 × 1 mL). The residue was chromatographed on silica (30–100% EtOAc/hexane) to give amid**e 3g** as a white solid (185.8 mg, 96%). mp 143–144 °C (precipitation form *n*-pentane/DCM); [*a*]_D_^23^ = −21.8 (*c* = 0.2, CHCl_3_); ^1^H NMR (500 MHz, CDCl_3_) δ 9.17 (br s, 1H), 8.48 (br s, 1H), 7.69 (br s, 1H), 7.59 (br s, 1H), 7.39–7.17 (m, 5H), 6.20 (br d, *J* = 6.0 Hz, 1H), 5.19–5.07 (m, 1H, C**H**CH_3_), 3.22–3.02 (m, 2H), 2.44–2.16 (m, 4H), 1.49 (d, *J* = 6.9 Hz, 3H, CHC**H**_3_); ^13^C{^1^H} NMR (125 MHz, CDCl_3_) δ 171.4, 165.4, 156.0, 154.1, 147.6, 143.4, 136.3–135.1 (m), 133.4, 128.6, 127.3, 126.3, 122.7, 119.8, 116.5, 48.9, 37.9, 35.6, 24.6, 22.0 (characteristic quartets are not visible due to strong broadening of signals); ^19^F NMR (376 MHz, CDCl_3_) δ −60.9; HR MS (ESI TOF) *m/z* calcd for C_21_H_20_F_3_N_3_Na [M + Na]^+^: 410.1456; found: 410.1446.

#### Ethyl 4-[4-(Trifluoromethyl)-1,8-naphthyridin-2-yl]butanoate (**3h**)

The compound was obtained according to GP1 using aminopyridine **1a** (95.0 mg, 0.50 mmol), alkyne **2c** (84.1 mg, 0.6 mmol, 1.2 equiv), copper(I) complex **4h** (12.1 mg, 0.01 mmol, 2 mol %), and a solution of TMG (1.25 μL, 0.01 mmol, 2 mol %) in water (2 mL). The resulting reaction mixture was heated at 120 °C for 19 h. Then, the reaction mixture was extracted with EtOAc (4 × 1 mL). The residue was chromatographed on silica (30–40% EtOAc/hexane) to give ester **3h** as a light yellow oil (155.9 mg, 99%). ^1^H NMR (200 MHz, CDCl_3_) δ 9.25–9.14 (m, 1H), 8.55–8.40 (m, 1H), 7.71 (s, 1H), 7.60 (dd, *J* = 8.5, 4.3 Hz, 1H), 4.14 (q, *J =* 7.2 Hz, 2H, CO_2_C**H**_2_CH_3_), 3.18 (t, *J* = 7.3 Hz, 2H), 2.55–2.40 (m, 2H), 2.38–2.20 (m, 2H), 1.26 (t, *J* = 7.1 Hz, 3H). Spectral data are in agreement with those reported.^23c^

#### {4-[6-Chloro-4-(trifluoromethyl)-1,8-naphthyridin-2-yl]piperidin-1-yl}(phenyl)methanone (**3i**)

The compound was obtained according to GP1 using aminopyridine **1b** (112.3 mg, 0.50 mmol), alkyne **S7** (128.0 mg, 0.60 mmol, 1.2 equiv), copper(I) complex **4h** (12.1 mg, 0.01 mmol, 2 mol %), and a solution of TMG (1.25 μL, 0.01 mmol, 2 mol %) in water (2 mL). The resulting reaction mixture was heated at 120 °C for 19 h. Then, the reaction mixture was saturated with solid NaCl and extracted with EtOAc (8 × 1.5 mL). The combined organic extracts were evaporated, and the residue was chromatographed on silica (50% EtOAc/hexane) to give naphthyridine **3i** as a beige solid (162.7 mg, 78%). mp 172–174 °C (*n*-heptane/MeOH); ^1^H NMR (500 MHz, DMSO-*d*_6_; 85 °C) δ 9.18 (br d, *J* = 2.6 Hz, 1H), 8.47–8.44 (m, 1H), 8.11 (s, 1**H**), 7.47–7.42 (m, 5H), 4.22 (br s, 2H, C**H**_2_), 3.43 (tt, *J* = 11.5, 3.8 Hz, 1H, ArC**H**), 3.15 (t, *J* = 12.6 Hz, 2H, C**H**_2_), 2.13–2.04 (m, 2H, C**H**_2_), 1.93 (dd, *J* = 11.8, 4.3, Hz, 1H, C**H**_2_), 1.88 (dd, *J* = 11.9, 4.3 Hz, 1H, C**H**_2_); ^13^C{^1^H} NMR (125 MHz, DMSO-*d*_6_, 85 °C) δ 168.7, 168.0, 153.3, 152.7, 136.2, 133.4 (q, *J*_CF_ = 31.8 Hz), 130.3 (q, *J*_CF_ = 2.7 Hz), 129.2, 128.8, 127.8, 126.2, 122.4 (q, *J*_CF_ = 273.6 Hz), 119.7 (q, *J*_CF_ = 5.1 Hz), 115.8, 43.6, 30.2 (one of the signals was covered by DMSO); ^19^F NMR (376 MHz, CDCl_3_) δ −61.0. HR MS (ESI TOF) *m*/*z* calcd for C_21_H_17_ClF_3_N_3_ONa [M + Na]^+^: 442.0910; found: 442.0900.

#### 8-{4-[4-(Trifluoromethyl)-1,8-naphthyridin-2-yl]phenyl}octan-1-ol (**3j**)

The compound was obtained according to GP1 using aminopyridine **1a** (95.0 mg, 0.50 mmol), alkyne **S9** (92.6 mg, 0.60 mmol, 1.2 equiv), copper(I) complex **4h** (12.1 mg, 0.01 mmol, 2 mol %), and a solution of TMG (1.25 μL, 0.01 mmol, 2 mol %) in water (2 mL). The reaction mixture was heated at 120 °C for 19 h. Then, the reaction mixture was extracted with EtOAc (4 × 1 mL), and the combined organic extracts were dried over Na_2_SO_4_ and evaporated. The residue was chromatographed on silica (40–50% EtOAc/hexane) to give alcohol **3j** as a white solid (144.8 mg, 89%). mp 106–107 °C (precipitation from a mixture of *n*-pentane/DCM); ^1^H NMR (400 MHz, CDCl_3_) δ 9.17–9.10 (m, 1H), 8.47–8.40 (m, 1H), 7.64 (s, 1H), 7.57–7.51 (m, 1H), 3.61 (t, *J* = 6.6 Hz, 2H), 3.08 (t, *J* = 7.9 Hz, 2H), 1.97–1.83 (m, 2H), 1.81 (br s, 1H), 1.60–1.48 (m, 2H), 1.46–1.26 (m, 8H); ^13^C{^1^H} NMR (100 MHz, CDCl_3_) δ 166.6, 156.2, 154.0, 135.4 (q, *J*_CF_ = 31.9 Hz), 133.3 (q, *J*_CF_ = 2.0 Hz), 123.0 (q, *J*_CF_ = 273.3 Hz), 122.5, 119.5 (q, *J*_CF_ = 4.9 Hz), 116.3, 62.9, 39.3, 32.7, 29.2 (×3), 29.0, 25.6; ^19^F NMR (376 MHz, CDCl_3_) δ −60.9; HR MS (EI EBE double focusing geometry mass analyzer) *m/z* calcd for C_17_H_21_F_3_N_2_O [M]^•+^: 326.1606; found: 326.1609.

### Gram-Scale Synthesis of 3k

#### 8-[6-Chloro-4-(trifluoromethyl)-1,8-naphthyridin-2-yl]octan-1-ol (**3k**)

The compound was obtained according to GP1 using aminopyridine **1b** (898.0 g, 4.0 mmol), alkyne **S9** (740.0 mg, 4.8 mmol, 1.2 equiv), copper(I) complex **4h** (97.0 mg, 0.08 mmol, 2 mol %), and a solution of TMG (10 μL, 0.08 mmol, 2 mol %) in water (16 mL). The glass pressure ampoule, attached to the Schlenk line, was charged with copper(I) complex and aminopyridine **1b** and then evacuated and backfilled with argon three times. A solution of TMG in water and alkyne **S9** were added, the ampoule was closed, and the reaction mixture was heated at 120 °C in an oil bath for 19 h. Then, the reaction mixture was saturated with solid NaCl and extracted with EtOAc (6 × 25 mL) and then with DCM (6 × 25 mL). The combined organic extracts were evaporated, dried over Na_2_SO_4_, and the residue was chromatographed on silica (30–50% EtOAc/hexane, then 3% MeOH/DCM) to give **3k** as a beige solid (1.33 g, 92%). mp 136–139 °C (*n*-heptane/DCM); ^1^H NMR (400 MHz, CDCl_3_) δ 9.07 (br s, 1H), 8.41 (br s, 1H), 7.69 (br s, 1H), 3.63 (t, *J* = 6.6 Hz, C**H**_2_OH, 2H), 3.63 (t, *J* = 7.7 Hz, ArC**H**_2_, 2H), 1.95–1.85 (m, 2H, C**H**_2_), 1.61–1.51 (m, 2H, C**H**_2_) overlapping 1.62–1.28 (m, 9H, 4 × C**H**_2_, 1 × O**H**). ^13^C{^1^H} NMR (50 MHz, CDCl_3_) δ 166.9, 154.7–153.9 (m), 153.4, 134.9 (q, *J* = 32.2 Hz), 131.6, 130.7–129.9 (m), 122.8 (*J* = 273.6 Hz), 121.1–120.1 (m), 116.6 (q, *J* = 5. 0 Hz), 63.0, 39.4, 32.8, 29.4, 29.3 (×2), 29.1, 25.7 (not all of the characteristic quartets have been precisely detected); ^13^C{^1^H} NMR spectrum could not be recorded at higher temperature due to the low solubility also at higher temp.; attempts to record the spectrum in DMSO-*d*_6_ or toluene-*d*_8_ failed for the same reason); ^19^F NMR (376 MHz, CDCl_3_) δ −61.1; HR MS (EI EBE double focusing geometry mass analyzer) *m/z* calcd for C_17_H_20_ClF_3_N_2_ONa [M + Na]^+^: 383.1114; found: 383.1116.

#### 2-(Tricyclo[3.3.1.13,7]dec-1-yl)-4-(trifluoromethyl)-1,8-naphthyridine (**3l**)

The compound was obtained according to GP1 using aminopyridine **1a** (95.0 mg, 0.50 mmol), alkyne **S8** (96.2 mg, 0.60 mmol, 1.2 equiv), copper(I) complex **4h** (12.1 mg, 0.01 mmol, 2 mol %), and a solution of TMG (1.25 μL, 0.01 mmol, 2 mol %) in water (2 mL). The reaction mixture was heated at 120 °C for 19 h. The reaction mixture was extracted with EtOAc (3 × 1 mL), and the combined organic extracts were dried over Na_2_SO_4_ and evaporated. The residue was chromatographed on silica (0.6% acetone/40% DCM/hexane = v/v/v) to give naphthyridine **3l** as a light brown solid (96 mg, 58%). mp 143.0–144.0 °C (*n*-heptane); ^1^H NMR (400 MHz, CDCl_3_) δ 9.18–9.12 (m, 1H), 8.48–8.41 (m, 1H), 7.86 (br s, 1H), 7.57–7.50 (m, 1H), 2.16 (s, 9H), 1.82 (s, 6H); ^13^C{^1^H} NMR (100 MHz, CDCl_3_) δ 172.7, 156.0, 153.9, 135.5 (q, *J*_CF_ = 31.5 Hz), 133.2 (q, *J*_CF_ = 2.2 Hz), 123.2 (q, *J*_CF_ = 273.4 Hz), 122.5, 116.2 (q, *J*_CF_ = 1.2 Hz), 116.0 (q, *J*_CF_ = 5.1 Hz), 41.5, 40.5, 36.6, 28.6; ^19^F NMR (376 MHz, CDCl_3_) δ −60.8; HR MS (EI EBE double focusing geometry mass analyzer) *m/z* calcd for C_19_H_19_F_3_N_2_ [M]^•+^: 332.1500; found: 332.1505.

#### 2,2**′**-Bicyclo[2.2.2]octane-1,4-diylbis[4-(trifluoromethyl)-1,8-naphthyridine] (**3m**)

The compound was obtained according to GP1 using aminopyridine **1a** (123.6 mg, 0.65 mmol, 2.6 equiv), alkyne **S10** (39.6 mg, 0.25 mmol), copper(I) complex **4h** (15.7 mg, 0.01 mmol, 2 mol %), and a solution of TMG (1.25 μL, 0.01 mmol, 2 mol %) in water (2 mL). Then, the reaction mixture was heated at 120 °C for 19 h, diluted with brine, and extracted with EtOAc (4 × 1 mL). The residue was chromatographed on silica (DCM to 5–10% acetone/DCM) to give bisnaphthyridine **3m** as a beige solid (25.0 mg, 20%). mp > 180 °C (decomposition, precipitation *n*-pentane/DCM); ^1^H NMR (500 MHz, CDCl_3_) δ 9.20 (br s, 2H), 8.53–8.47 (m, 2H), 7.92 (s, 2H), 7.63–7.57 (m, 2H), 2.33 (s, 12H); ^13^C{^1^H} NMR (125 MHz, CDCl_3_) δ 171.7, 155.7, 154.0, 135.7 (q, *J*_CF_ = 31.8 Hz), 133.5 (q, *J*_CF_ = 2.0 Hz), 123.1 (q, *J*_CF_ = 273.3 Hz), 122.7, 116.7 (q, *J*_CF_ = 5.0 Hz), 116.4 (q, *J* = 0.9 Hz), 40.0, 31.1; ^19^F NMR (376 MHz, CDCl_3_) δ −60.7; HR MS (EI EBE double focusing geometry mass analyzer) *m/z* calcd for C_26_H_20_F_6_N_4_ [M]^•+^: 502.1592; found: 502.1595.

#### 2-(Thiophen-3-yl)-4-(trifluoromethyl)-1,8-naphthyridine (**3n**)

The compound was obtained according to GP1 using aminopyridine **1a** (95.0 mg, 0.50 mmol), alkyne **S11** (59.1 μL, 0.60 mmol, 1.2 equiv), copper(I) complex **4h** (12.1 mg, 0.01 mmol, 2 mol %), and a solution of TMG (1.25 μL, 0.01 mmol, 2 mol %) in water (2 mL). The reaction mixture was heated at 120 °C for 19 h. Then, it was diluted with brine and extracted with EtOAc (5 × 1 mL). The combined organic extracts were dried over Na_2_SO_4_ and evaporated. The residue was chromatographed on silica (30% EtOAc/hexane, Combi-Flash) to give a light orange solid (117.0 mg, 83%). mp 178.0–179.0 °C (precipitation from *n*-pentane/DCM); ^1^H NMR (400 MHz, CDCl_3_) δ 9.17 (br s, 1H), 8.45 (br d, *J* = 8.2 Hz 1H), 8.27 (br d, *J* = 2.0 Hz, 1H), 8.12 (s, 1H), 7.98 (d, *J* = 4.9 Hz, 1H), 7.60–7.50 (m, 1H), 7.48–7.43 (m, 1H); ^13^C{^1^H} NMR (100 MHz, CDCl_3_) δ 156.6, 155.7, 154.7–154.4 (m), 140.8, 133.2, 128.8–128.4 (m), 117.2–117.0 (m), 116.9–116.7 (m) (none of the characteristic quartets have been detected due to broadening of signals); ^19^F NMR (376 MHz, CDCl_3_) δ −61.1; HR MS (EI EBE double focusing geometry mass analyzer) *m*/*z* calcd for C_13_H_7_F_3_N_2_S [M]^•+^: 280.0282; found: 280.0283.

#### 2-(1-Benzofuran-2-yl)-4-(trifluoromethyl)-1,8-naphthyridine (**3o**)

The compound was obtained according to GP1 using aminopyridine **1a** (95.0 mg, 0.50 mmol), alkyne **S12** (85.3 mg, 0.60 mmol, 1.2 equiv), copper(I) complex **4h** (12.1 mg, 0.01 mmol, 2 mol %), and a solution of TMG (1.25 μL, 0.01 mmol, 2 mol %) in water (2 mL). Then, the reaction mixture was heated to 120 °C for 19 h, diluted with brine, and extracted with EtOAc (4 × 1 mL). The residue was chromatographed on silica (15–25% EtOAc/hexane, Combi-Flash, 24 g column) to give naphthyridine **3o** as a brown solid (59.9 mg, 38%).

#### 2-(1-Benzofuran-2-yl)-4-(trifluoromethyl)-1,8-naphthyridine (**3o**)

The compound was obtained according to GP1 using aminopyridine **1a** (95.0 mg, 0.50 mmol), 2-(prop-2-yn-1-yloxy)benzaldehyde **2b** (85.3 mg, 0.60 mmol, 1.2 equiv), copper(I) complex **4h** (12.1 mg, 0.01 mmol, 2 mol %), and a solution of TMG (1.25 μL, 0.01 mmol, 2 mol %) in water (2 mL). The reaction mixture was heated to 120 °C for 19 h. Then, it was diluted with brine and extracted with DCM (4 × 1 mL). The residue was chromatographed on silica (20% EtOAc/hexane, Combi-Flash, 12 g column) to give naphthyridine **3o** as a brown solid (27.7 mg, 25%). Prolonged reaction time (41 h) afforded product **3o** with a slightly lower 18% yield. mp 200–207 °C (precipitation from *n*-pentane/DCM); ^1^H NMR (400 MHz, CDCl_3_) δ 9.24–9.16 (m, 1H), 8.55–8.40 (m, 2H), 7.92 (s, 1H), 7.72 (br d, *J* = 7.7 Hz, 1H), 7.66–7.52 (m, 2H), 7.48–7.36 (m, 1H), 7.35–7.18 (m, 1H); ^13^C{^1^H} NMR (100 MHz, CDCl_3_) δ 156.4, 155.9, 154.8, 153.4, 151.9, 136.5 (q, *J*_CF_ = 32.4 Hz), 133.4 (q, *J*_CF_ = 1.9 Hz), 128.6, 126.6, 123.7, 122.9, 122.8 (q, *J*_CF_ = 273.1 Hz), 122.5, 117.4 (q, *J*_CF_ = 1.0 Hz), 116.2 (q, *J*_CF_ = 5.2 Hz), 111.8, 108.9; ^19^F NMR (376 MHz, CDCl_3_) δ −61.0; HR MS (EI EBE double focusing geometry mass analyzer) *m/z* calcd for C_17_H_9_F_3_N_2_O [M]^•+^: 314.0667; found: 314.0667.

#### 2-(1-Methyl-1*H*-indol-3-yl)-4-(trifluoromethyl)-1,8-naphthyridine (**3p**)

The compound was obtained according to GP1 using aminopyridine **1a** (47.5 mg, 0.25 mmol), alkyne **S13** (46.6 mg, 0.30 mmol, 1.2 equiv), copper(I) complex **4h** (6.1 mg, 0.01 mmol, 2 mol %), and a solution of TMG (1.25 μL, 0.01 mmol, 2 mol %) in water (1 mL). The resulting reaction mixture was heated at 120 °C for 19 h. Then, the reaction mixture was diluted with brine and extracted with EtOAc (4 × 1 mL). The combined organic extracts were dried over Na_2_SO_4_ and evaporated. The residue was chromatographed on silica (30% EtOAc/hexane) to give naphthyridine **3p** as a yellow solid (69.5 mg, 85%). mp 239.0–241.0 °C (precipitated from a mixture *n*-pentane/DCM); ^1^H NMR (400 MHz, CDCl_3_) δ 9.16–9.11 (m, 1H), 8.88–8.81 (m, 1H), 8.43–8.36 (m, 1H), 8.12 (s, 1H), 7.96 (s, 1H), 7.50–7.43 (m, 1H), 7.40–7.32 (m, 3H), 3.89 (s, 3H, C**H**_3_); ^13^C{^1^H} NMR (100 MHz, CDCl_3_) δ 157.7, 157.0, 153.9, 138.1, 134.9 (q, *J*_CF_ = 31.5 Hz), 133.2 (q, *J*_CF_ = 2.0 Hz), 131.8, 126.1, 123.1 (q, *J*_CF_ = 273.1 Hz), 123.1, 122.9, 121.9, 121.3, 117.2 (q, *J*_CF_ = 5.1 Hz), 115.6 (q, *J*_CF_ = 1.0 Hz), 114.9, 109.6, 33.4 (**C**H_3_); ^19^F NMR (376 MHz, CDCl_3_) δ −61.2; HR MS (EI EBE double focusing geometry mass analyzer) calc for C_18_H_12_F_3_N_3_ [M]^•+^: 327.0983; found: 327.0978.

#### 3-{4-[4-(Trifluoromethyl)-1,8-naphthyridin-2-yl]butoxy}estra-1(10),2,4-trien-17-one (**3q**)

The compound was obtained according to GP1 using aminopyridine **1a** (47.5 mg, 0.25 mmol), alkyne **S14** (105.2 mg, 0.30 mmol, 1.2 equiv), copper(I) complex **4h** (6.1 mg, 0.01 mmol, 2 mol %), and a solution of TMG (1.25 μL, 0.01 mmol, 2 mol %) in water (1 mL). The resulting reaction mixture was heated at 120 °C for 19 h. Then, the reaction mixture was diluted with brine and extracted with EtOAc (4 × 1 mL). The residue was chromatographed on silica (40–50% EtOAc/hexane) to give naphthyridine **3q** as a dark red oil (129.3 mg, 99%). [*a*]_D_^23^ = 99.2 (*c* = 0.2, CHCl_3_); ^1^H NMR (400 MHz, CDCl_3_) δ 9.18–9.12 (m, 1H), 8.48–8.41 (m, 1H), 7.68 (s, 1H), 7.59–7.53 (m, 1H), 7.14 (d, *J* = 2.1 Hz, 1H), 6.70–6.64 (m, 1H), 6.62–6.57 (m, 1H), 3.98 (t, *J* = 6.2 Hz, 2H), 3.18 (t, *J* = 7.7 Hz, 2H), 2.90–2.78 (m, 2H), 2.53–2.42 (m, 1H), 2.41–2.32 (m, 1H), 2.27–1.82 (m, 9H), 1.66–1.32 (m, 6H), 0.88 (s, 3H, C**H**_3_); ^13^C{^1^H} NMR (100 MHz, CDCl_3_) δ 166.1, 156.9, 156.1, 154.0, 137.7, 135.5 (q, *J* = 32.0 Hz), 133.4 (q, *J*_CF_ = 1.9 Hz), 132.0, 126.3, 122.9 (q, *J*_CF_ = 1.9 Hz), 122.6, 119.6 (q, *J*_CF_ = 4.7 Hz), 116.4 (q, *J*_CF_ = 1.0 Hz), 114.5, 112.1, 67.4, 50.4, 48.0, 44.0, 38.8, 38.4, 35.8, 31.6, 29.6, 28.9, 26.5, 25.9, 25.6, 21.6, 13.8; ^19^F NMR (376 MHz, CDCl_3_) δ −60.9; HR MS (EI EBE double focusing geometry mass analyzer) *m/z* calcd for C_31_H_33_F_3_N_2_O_2_ [M]^•+^: 522.2494; found: 522.2488.

#### 5-[6-Chloro-4-(trifluoromethyl)-1,8-naphthyridin-2-yl]pentyl 6-chloro-1-cyclopropyl-7-fluoro-4-oxo-1,4-dihydroquinoline-3-carboxylate (**3r**)

The compound was obtained according to GP1 using aminopyridine **1b** (56.1 mg, 0.50 mmol), alkyne **S15** (112.7 mg, 0.60 mmol, 1.2 equiv), copper(I) complex **4h** (6.0 mg, 0.01 mmol, 2 mol %), and a solution of TMG (0.625 μL, 0.01 mmol, 2 mol %) in water (2 mL). The resulting reaction mixture was heated at 120 °C for 19 h. Then, the reaction mixture was saturated with solid NaCl and extracted with EtOAc (4 × 1.5 mL) and DCM (8 × 1.5 mL). The combined organic extracts were evaporated, and the residue was chromatographed on silica (75% MTBE/hexane, then MTBE, then 66% MTBE/DCM) to give naphthyridine **3r** as a beige solid (133.0 mg, 91%). mp 162–164 °C (*n*-heptane/DCM); ^1^H NMR (500 MHz, DMSO-*d*_6_, 85 °C) δ 9.12–9.10 (m, 1H), 8.45–8.43 (m, 1H), 8.42–8.39 (m, 1H), 8.24–8.21 (m, 1H), 8.02 (s, 1H, C=CHN), 7.96–7.92 (m, 1H), 4.23 (t, *J* = 6.5 Hz, 2H, CH_2_O), 3.69–3.64 [m, 1H, NCH(CH_2_)_2_], 3.13 (t, *J* = 7.6 Hz, 2H, CH_2_Ar), 1.98–1.90 (m, 2H, CH_2_), 1.81–1.74 (m, 2H, CH_2_), 1.61–1.53 (m, 2H, CH_2_), 1.30–1.25 [m, 2H, NCH(CH_2_)_2_], 1.12–1.06 [m, 2H, NCH(CH_2_)_2_]; ^13^C{^1^H} NMR (125 MHz, DMSO-*d*_6_, 85 °C) δ 170.8, 166.3, 163.7, 154.2 (d, *J*_CF_ = 245.9 Hz), 153.4, 152.5, 148.0, 137.2, 132.9 (q, *J*_CF_ = 31.8 Hz), 130.3 (q, *J*_CF_ = 2.6 Hz), 128.9, 127.7 (d, *J*_CF_ = 5.5 Hz), 124.7 (d, *J*_CF_ = 19.8 Hz), 122.4 (q, *J*_CF_ = 273.6 Hz), 120.6 (q, *J*_CF_ = 5.1 Hz) 119.6, 115.5, 111.9, (d, *J*_CF_ = 22.5 Hz), 109.9, 63.4, 37.7, 34.5, 27.6, 27.2, 24.7, 7.1. ^19^F NMR (376 MHz, CDCl_3_) δ −61.6, −118.1. HR MS (ESI TOF) m/z calcd for C_27_H_21_Cl_2_F_4_N_3_O_3_Na [M + Na]^+^: 604.0794; found: 604.0789.

#### 4-[6-Chloro-4-(trifluoromethyl)-1,8-naphthyridin-2-yl]butyl 5-[(3*aS*,4*S*,6*aR*)-2-oxohexahydro-1*H*-thieno[3,4-*d*]imidazol-4-yl]pentanoate (**3s**)

The compound was obtained according to GP1 using aminopyridine **1b** (112.3 mg, 0.50 mmol), alkyne **S16** (194.5 mg, 0.60 mmol, 1.2 equiv), copper(I) complex **4h** (12.1 mg, 0.01 mmol, 2 mol %), and a solution of TMG (1.25 μL, 0.01 mmol, 2 mol %) in water (2 mL). The resulting reaction mixture was heated at 120 °C for 19 h. Then, the reaction mixture was saturated with solid NaCl and extracted with EtOAc (4 × 1.5 mL) and then with DCM (4 × 1.5 mL). The combined organic extracts were evaporated, and the residue was chromatographed on silica (1–2% MeOH/EtOAc) to give naphthyridine **3s** as a white solid (238.5 mg, 90%). mp > 110 °C (decomposition, *n*-heptane/DCM). ^1^H NMR (200 MHz, DMSO-*d*_6_) δ 9.19 (d, *J* = 2.5 Hz, 1H), 8.49–8.42 (m, 1H), 8.10 (br s, 1H), 6.44 (s, 1H, NH), 6.37 (s, 1H, NH), 4.34–4.22 (m, 1H), 4.16–4.00 (m, 1H) overlapping 4.06 (t, 2H, CH_2_O), 3.17–3.00 (m, 3H), 2.79 (dd, *J* = 12.4, 5.0 Hz, 1H), 2.28 (t, *J* = 7.2 Hz, 2H), 1.98–1.78 (m, 2H), 1.77–1.21 (m, 9H). ^13^C{^1^H} NMR (50 MHz, DMSO-*d*_6_) δ 172.9, 166.5, 162.7, 153.7, 153.2, 133.2 (q, *J*_CF_ = 31.8 Hz), 130.9, 129.5, 122.7 (q, *J*_CF_ = 273.3 Hz), 121.3 (q, *J*_CF_ = 4.9 Hz), 115.9, 63.5, 61.0, 59.2, 55.4, 38.3, 37.6, 33.3, 29.6, 28.0, 27.8, 24.7, 24.5. ^19^F NMR (376 MHz, CDCl_3_) δ −61.1. HR MS (ESI TOF) *m*/*z* calcd for C_23_H_26_ClF_3_N_4_O_3_SNa [M + Na]^+^: 553.1254; found: 553.1264.

#### 4-[6-Chloro-4-(trifluoromethyl)-1,8-naphthyridin-2-yl]-*N*-{4-[methyl(5-methyl-1,2-oxazol-3-yl)sulfamoyl]phenyl}butanamide (**3t**)

The compound was obtained according to GP1 using aminopyridine **1b** (56.1 mg, 0.50 mmol), alkyne **S17** (108.4 mg, 0.60 mmol, 1.2 equiv), copper(I) complex **4h** (6.0 mg, 0.01 mmol, 2 mol %), and a solution of TMG (0.625 μL, 0.01 mmol, 2 mol %) in water (2 mL). The resulting reaction mixture was heated at 120 °C for 19 h. Then, the reaction mixture was saturated with solid NaCl and extracted with EtOAc (8 × 1.5 mL). The combined organic extracts were evaporated, and the residue was chromatographed on silica (3–5% acetone/DCM) to give naphthyridine **3t** as a white foam (92.2. mg, 65%). ^1^H NMR (400 MHz, DMSO-*d*_6_) δ 10.33 (s, 1H), 9.16 (s, 1H), 8.45–8.40 (m, 1H), 8.08 (s, 1H), 7.77–7.65 (m, 4H), 6.48 (br s, 1H), 3.17 (s, 3H, CH_3_), 3.15 (t, *J* = 7.4 Hz, 2H, CH_2_), 2.48 (t, *J* = 7.2 Hz, 2H, CH_2_, partially overlapped by residual peaks of DMSO), 2.35 (s, 3H, CH_3_), 2.22–2.13 (m, 2H, CH_2_); ^13^C{^1^H} NMR (50 MHz, DMSO-*d*_6_) δ 171.7, 170.9, 166.2, 160.2, 153.7, 153.2, 144.1, 133.2 (q, *J*_CF_ = 31.7 Hz), 130.9, 129.5, 129.3, 128.3, 122.7 (q, *J*_CF_ = 273.4 Hz), 121.5 (q, *J*_CF_ = 5.1 Hz), 118.8, 116.0, 97.0, 37.4, 35.7, 35.0, 23.7, 12.2. ^19^F NMR (376 MHz, DMSO-*d*_6_) δ −60.0. HR MS (ESI TOF) *m*/*z* calcd for C_24_H_21_ClF_3_N_5_O_4_SNa [M + Na]^+^: 590.0853; found: 590.0853.

#### 4-[6-Chloro-4-(trifluoromethyl)-1,8-naphthyridin-2-yl]butyl (4*E*)-6-(4-hydroxy-6-methoxy-7-methyl-3-oxo-1,3-dihydro-2-benzofuran-5-yl)-4-methylhex-4-enoate (**3u**)

The compound was obtained according to GP1 using aminopyridine **1b** (56.1 mg, 0.50 mmol), alkyne **S18** (154.4 mg, 0.60 mmol, 1.2 equiv), copper(I) complex **4h** (6.0 mg, 0.01 mmol, 2 mol %), and a solution of TMG (0.625 μL, 0.01 mmol, 2 mol %) in water (2 mL). The resulting reaction mixture was heated at 120 °C for 19 h. Then, the reaction mixture was saturated with solid NaCl and extracted with EtOAc (8 × 1.5 mL). The combined organic extracts were evaporated. The residue was chromatographed on silica (50–66% MTBE/hexane) to give naphthyridine **3u** as a brown oil (147.1 mg, 97%). ^1^H NMR (200 MHz, DMSO-*d*_6_) δ 9.36 (s, 1H), 9.17 (br s, 1H), 8.47–8.40 (m, 1H), 8.11–8.04 (m, 1H), 5.22 (s, 2H, ArCH_2_O), 5.11 (br t, *J* = 6.9 Hz, 1H, C = CH), 3.99 (t, *J* = 6.4 Hz, 2H, RCH_2_O), 3.65 (s, 3H, OCH_3_), 3.23 (d, *J* = 6.8 Hz, 2H, C=CHCH_2_Ar), 3.06 (t, *J* = 7.4 Hz, 2H, CH_2_), 2.34 (br t, *J* = 6.9 Hz, 2H, CH_2_), 2.16 (br t, *J* = 7.1 Hz, 2H, CH_2_), 2.04 (s, 3H, CH_3_), 1.90–1.51 (m, 4H, CH_2_) overlapping 1.70 (s, 3H, CH_3_); ^13^C{^1^H} NMR (50 MHz, DMSO-*d*_6_) δ 172.5, 170.1, 166.5, 162.5, 153.7, 153.2, 152.7, 145.8, 133.2 (q, *J*_CF_ = 31.7 Hz), 133.2, 130.9, 129.5 (×2), 123.0, 122.7 (q, *J*_CF_ = 273.6 Hz), 122.3, 121.3 (q, *J*_CF_ = 4.0 Hz), 115.9, 106.9, 68.6, 63.5, 60.6, 37.6, 34.1, 32.3, 27.7, 24.7, 22.4, 15.9, 11.0; ^19^F NMR (376 MHz, CDCl_3_) δ −61.1. HR MS (ESI TOF) *m*/*z* calcd for C_30_H_30_ClF_3_N_2_O_6_Na [M + Na]^+^: 629.1642; found: 629.1638.

#### (3β)-Cholest-5-en-3-yl 4-[4-(trifluoromethyl)-1,8-naphthyridin-2-yl]butanoate (**3v**)

The compound was obtained according to GP1 using aminopyridine **1a** (47.5 mg, 0.25 mmol), alkyne **S19** (144.0 mg, 0.30 mmol, 1.2 equiv), copper(I) complex **4h** (6.1 mg, 0.01 mmol, 2 mol %), and a solution of TMG (1.25 μL, 0.01 mmol, 2 mol %) in water (1 mL). Then, the reaction mixture was heated at 120 °C for 19 h, diluted with brine, and extracted with EtOAc (4 × 1 mL). The residue was chromatographed on silica (10% EtOAc/hexane, Combi-Flash, 24 g column) to give naphthyridine **3v** as a colorless oil (86.8 mg, 53%). [*a*]_D_^23^ = −19.0 (*c* = 0.2, CHCl_3_); ^1^H NMR (400 MHz, CDCl_3_) δ 9.18–9.13 (m, 1H), 8.49–8.42 (m, 1H), 7.68 (s, 1H), 7.59–7.53 (m, 1H), 5.37–5.32 (m, 1H, **C6**), 4.66–4.54 (m, 1H, **C3**), 3.15 (t, *J* = 1.9 Hz, 2H), 2.42 (t, *J* = 1.8 Hz, 2H), 2.33–2.20 (m, 4H), 2.03–1.90 (m, 2H), 1.88–1.74 (m, 3H), 1.63–1.39 (m, 7H), 1.38–1.20 (m, 5H), 1.19–0.97 (m, 9H) overlapping 0.99 (s, 3H), 0.90 (d, *J* = 1.6 Hz, 3H), 0.85 (d, *J* = 0.4 Hz, 3H), 0.84 (d, *J* = 0.4 Hz, 3H), 0.66 (s, 3H); ^13^C{^1^H} NMR (100 MHz, CDCl_3_) δ 172.5, 165.3, 156.2, 154.1, 139.6, 135.6 (q, *J*_CF_ = 31.9 Hz), 133.3 (q, *J*_CF_ = 2.0 Hz), 122.9 (q, *J*_CF_ = 273.3 Hz), 122.6, 119.6 (q, *J*_CF_ = 4.8 Hz), 118.8, 116.5 (q, *J*_CF_ = 2.9 Hz), 74.0, 56.7, 56.1, 50.0, 42.3, 39.7, 39.5, 38.3, 38.1, 37.0, 36.6, 36.2, 35.8, 33.9, 31.9, 31.8, 28.2, 28.0, 27.8, 24.3, 24.0, 23.8, 22.8, 22.5, 21.0, 19.3, 18.7, 11.8; ^19^F NMR (376 MHz, CDCl_3_) δ −60.9; HR MS (EI EBE double focusing geometry mass analyzer) *m/z* calcd for C_40_H_55_F_3_N_2_O_2_ [M]^•+^: 652.4216; found: 652.4196.

#### Ethyl 4-[4-(Difluoromethyl)-1,8-naphthyridin-2-yl]butanoate (**3w**)

The compound was obtained according to GP1 using aminopyridine **1a** (86.0 mg, 0.50 mmol), alkyne **2c** (84.1 mg, 0.60 mmol, 1.2 equiv), copper(I) complex **4h** (12.1 mg, 0.01 mmol, 2 mol %), and a solution of TMG (1.25 μL, 0.01 mmol, 2 mol %) in water (2 mL). The resulting reaction mixture was heated at 120 °C for 19 h. Then, the reaction mixture was diluted with brine (1 mL) and extracted with EtOAc (3 × 1 mL). The combined organic extracts were dried over Na_2_SO_4_ and evaporated. The residue was chromatographed on silica (30–40% EtOAc/hexane) to give naphthyridine **3w** as a creamy solid (115.5 mg, 78%). mp 74.0–75.0 °C (*n*-heptane); ^1^H NMR (400 MHz, CDCl_3_) δ 9.15 (dd, *J* = 4.2, 1.9 Hz, 1H), 8.49–8.44 (m, 1H), 7.57–7.50 (m, 2H), 7.07 (t, *J*_HF_ = 54.3 Hz, 1H, CF_2_**H**), 4.12 (q, *J* = 7.2 Hz, 2H, CO_2_C**H**_2_), 3.15 (t, *J* = 7.4 Hz, 2H), 2.45 (t, *J* = 7.3 Hz, 2H), 2.31–2.21 (m, 2H), 1.24 (t, *J* = 7.1 Hz, 3H, CO_2_CH_2_C**H**_3_); ^13^C{^1^H} NMR (100 MHz, CDCl_3_) δ 173.1, 165.5, 156.0, 153.7, 139.3 (t, *J*_CF_ = 22.0 Hz), 133.0, 122.1, 119.9 (t, *J* = 7.5 Hz), 117.4 (t, *J* = 2.3 Hz), 113.3 (t, *J* = 239.6 Hz), 60.3, 38.2, 33.6, 23.9, 14.1; ^19^F NMR (376 MHz, CDCl_3_) δ −113.2; HR MS (EI EBE double focusing geometry mass analyzer) calcd for C_15_H_16_F_2_N_2_O_2_ [M]^•+^: 294.1180; found: 294.1179.

#### (3β)-Cholest-5-en-3-yl 4-[4-(difluoromethyl)-1,8-naphthyridin-2-yl]butanoate (**3x**)

The compound was obtained according to GP1 using aminopyridine **1a** (43.0 mg, 0.25 mmol), alkyne **S19** (144.2 mg, 0.30 mmol, 1.2 equiv), copper(I) complex **4h** (6.1 mg, 0.01 mmol, 2 mol %), and a solution of TMG (1.25 μL, 0.01 mmol, 2 mol %) in water (1 mL). The resulting reaction mixture was heated at 120 °C for 19 h. Then, the reaction mixture was extracted with EtOAc (3 × 1 mL). The residue was chromatographed on silica (40% EtOAc/hexane) to give naphthyridine **3x** as a brown oil (137.0 mg, 86%). [*a*]_D_^23^ = −23.6 (*c* = 0.2, CHCl_3_); ^1^H NMR (400 MHz, CDCl_3_) δ 9.14 (dd, *J* = 4.2, 1.9 Hz, 1H), 8.49–8.43 (m, 1H), 7.57–7.49 (m, 2H), 7.06 (t, *J*_HF_ = 54.3 Hz, 1H, CF_2_**H**), 5.38–5.33 (m, 1H, **C6**), 4.66–4.54 (m, 1H, **C3**), 3.14 (t, *J* = 7.5 Hz, 2H), 2.42 (t, *J* = 7.3 Hz, 2H), 2.33–2.20 (m, 4H), 2.05–1.91 (m, 2H), 1.89–1.74 (m, 3H), 1.68–1.40 (m, 21H) overlapping 1.00 (s, 3H) and 0.91 (d, *J* = 6.5 Hz, 3H) and 0.86 (d, *J* = 1.8 Hz, 3H) and 0.85 (d, *J* = 1.7 Hz, 3H), 0.67 (s, 3H); ^13^C{^1^H} NMR (100 MHz, CDCl_3_) δ 172.6, 165.5, 156.2, 153.8, 139.6, 139.3 (t, *J*_CF_ = 21.8 Hz), 133.0, 122.6, 122.2, 119.9 (t, *J*_CF_ = 7.6 Hz), 117.4 (t, *J*_CF_ = 2.3 Hz), 113.4 (t, *J*_CF_ = 239.7 Hz), 74.0, 56.7, 56.2, 50.0, 42.3, 39.7, 39.5, 38.3, 38.1, 37.0, 36.6, 36.2, 35.8, 34.0, 31.9 (d, *J* = 3.4 Hz), 28.2, 28.0, 27.8, 24.3, 24.1, 23.8, 22.8, 22.5, 21.0, 19.3, 18.7, 11.8; ^19^F NMR (376 MHz, CDCl_3_) δ −113.2; HR MS (EI EBE double focusing geometry mass analyzer) calcd for C_40_H_56_F_2_N_2_O_2_ [M]^•+^: 634.4310; found: 634.4286.

#### 5-[4-(Difluoromethyl)-1,8-naphthyridin-2-yl]pentyl 6-chloro-1-cyclopropyl-7-fluoro-4-oxo-1,4-dihydroquinoline-3-carboxylate (**3y**)

The compound was obtained according to GP1 using aminopyridine **1a** (43.1 mg, 0.50 mmol), alkyne **S15** (112.7 mg, 0.60 mmol, 1.2 equiv), copper(I) complex **4h** (6.0 mg, 0.01 mmol, 2 mol %), and a solution of TMG (0.625 μL, 0.01 mmol, 2 mol %) in water (2 mL). The resulting reaction mixture was heated at 120 °C for 43 h. Then, the reaction mixture was diluted with toluene and evaporated (to remove water—2 times). The residue was chromatographed on silica (DCM to 5% MeOH/DCM) to give naphthyridine **3y** as a beige solid (121.9 mg, 86%). mp 154–156 °C (*n*-heptane/DCM); ^1^H NMR (400 MHz, CDCl_3_) δ 9.13 (s, 1H), 8.62–8.39 (m, 2H), 8.16 (d, *J* = 9.0 Hz, 1H), 7.98 (d, *J* = 5.8 Hz, 1H), 7.65–7.45 (m, 2H), 7.07 (t, 54.3 Hz, 1H, CHF_2_), 4.33 (t, *J* = 6.5 Hz, 2H, CH_2_O), 3.48–3.41 [m 1H, NCH(CH_2_)_2_], 3.12 (bs, 2H, CH_2_), 2.01 (bs, 2H, CH_2_), 1.91–1.81 (m, 2H, CH_2_), 1.69–1.56 (m, 2H, CH_2_), 1.36 [dd, *J* = 13.4, 6.7 Hz, 2H, NCH(CH_2_)_2_], 1.14 [dd, *J* = 9.2, 6.5 Hz, 2H, NCH(CH_2_)_2_]; ^13^C{^1^H} NMR (50 MHz, DMSO-*d*_6_) δ 171.3 (d, *J*_CF_ = 2.2 Hz), 166.0, 164.1, 155.4, 154.5 (d. *J*_CF_ = 245.6 Hz) 153.7, 148.9, 139.1 (t, *J*_CF_ = 21.8 Hz), 137.5, 133.3, 127.9 (d, (*J*_CF_ = 5.7 Hz), 125.1 (d, *J*_CF_ = 20.1 Hz), 122.3 (t, *J*_CF_ = 3.4 Hz), 120.2, 119.7 (t, *J*_CF_ = 6.1 Hz), 116.9 (t, *J*_CF_ = 4.6 Hz), 113.5 (t, *J*_CF_ = 236.6 Hz), 112.3 (d, *J*_CF_ = 22.5 Hz), 109.6, 79.2, 63.9, 35.1, 28.2, 28.0, 25.3, 7.6; ^19^F NMR (376 MHz, CDCl_3_) δ −113.04, −118.07. HR MS (ESI TOF) *m*/*z* calcd for C_27_H_23_ClF_3_N_3_O_3_Na [M + Na]^+^: 552.1278; found: 552.1276.

## Supporting Information Available

Experimental procedures, compound characterization data, and computational data (PDF)
Copies of NMR spectra (PDF)

## Accession Codes

CCDC 2143425–2143428 contain the supplementary crystallographic data for this paper. These data can be obtained free of charge via www.ccdc.cam.ac.uk/data_request/cif, or by emailing data_request@ccdc.cam.ac.uk, or by contacting The Cambridge Crystallographic Data Centre, 12 Union Road, Cambridge CB2 1EZ, UK; fax: +44 1223 336033.
