# Supplementary material for: NHC–BIAN–Cu(I)-Catalyzed Friedländer-Type Annulation of 2-Amino-3-(per)fluoroacetylpyridines with Alkynes on Water
Source: J Org Chem. 2022 Apr 8;87(9):6115–36. doi: 10.1021/acs.joc.2c00380 (PMC9087358; doi:10.1021/acs.joc.2c00380)
Supplement: Supplementary file 1 — jo2c00380_si_001.pdf [file jo2c00380_si_001.pdf]

Electronic Supplementary Information

for

NHC-BIAN-Cu(I)-Catalyzed Friedländer-Type Annulation of 2-Amino-3-(per)fluoroacetylpyridines with Alkynes on Water

Magdalena Dolna,[a] Michał Nowacki,[a] Oksana Danyluk,[b] Artur Brotons-Rufes,[c]
Albert Poater,*[c] and Michał Michalak*[a]
*e-mail: albert.poater@udg.edu, michal.michalak@icho.edu.pl

[a] Institute of Organic Chemistry, Polish Academy of Sciences, 01-224 Warsaw, Kasprzaka 44/52, Poland
[b] Institute of Physical Chemistry, Polish Academy of Sciences, 01-224 Warsaw, Kasprzaka 44/52, Poland
[c] Institut de Química Computacional i Catàlisi and Departament de Química, Universitat de Girona, Campus Montilivi, 17071 Girona, Catalonia, Spain

Table of contents

1. Synthetic route leading to NHC precursors, gold(I) complexes, alkynes and structure of the respective products. 2
2. Optimization studied leading to naphthyridines **3a**. 5
3. Single crystal X-ray diffraction 7
4. Computational details 9
5. Computational results 9
6. Steric Maps 12
7. References 197

1. Synthetic route leading to NHC precursors, gold(I) complexes, alkynes and structure of the respective products.

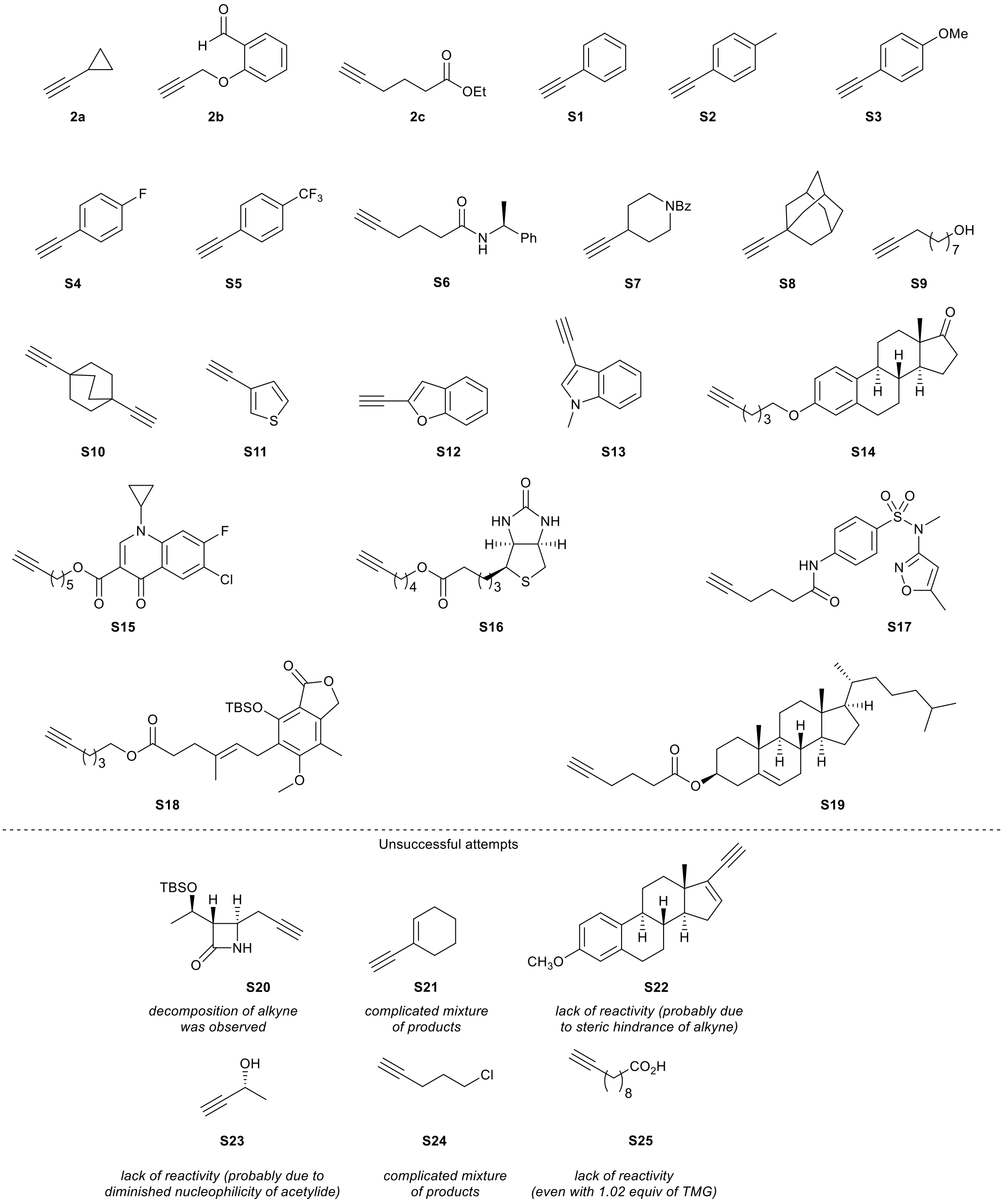

Figure S1. Alkynes used in this studies

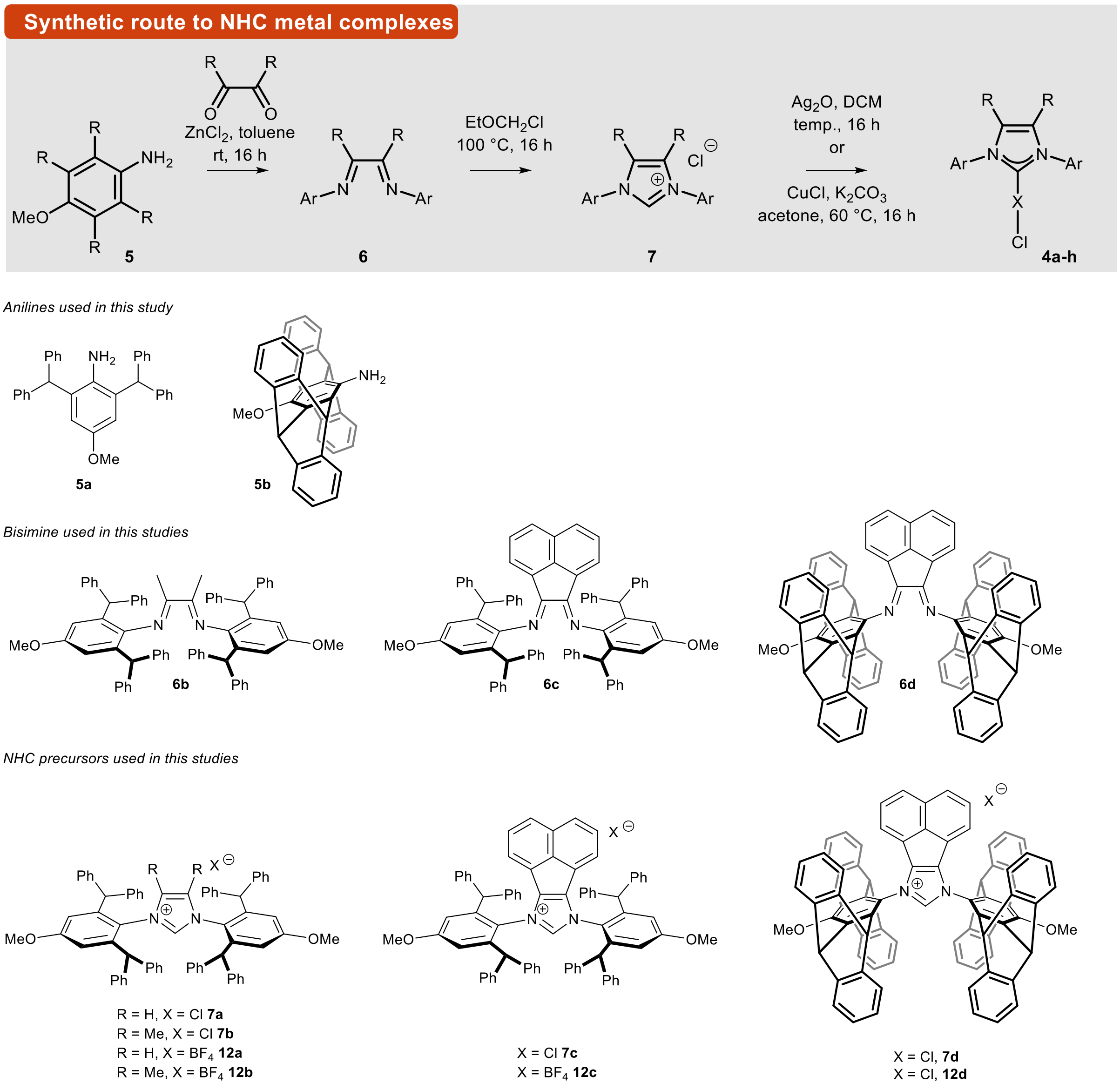

Scheme S1. Synthetic route leading to NHC precursors and structure of bisimines and NHC precursors.

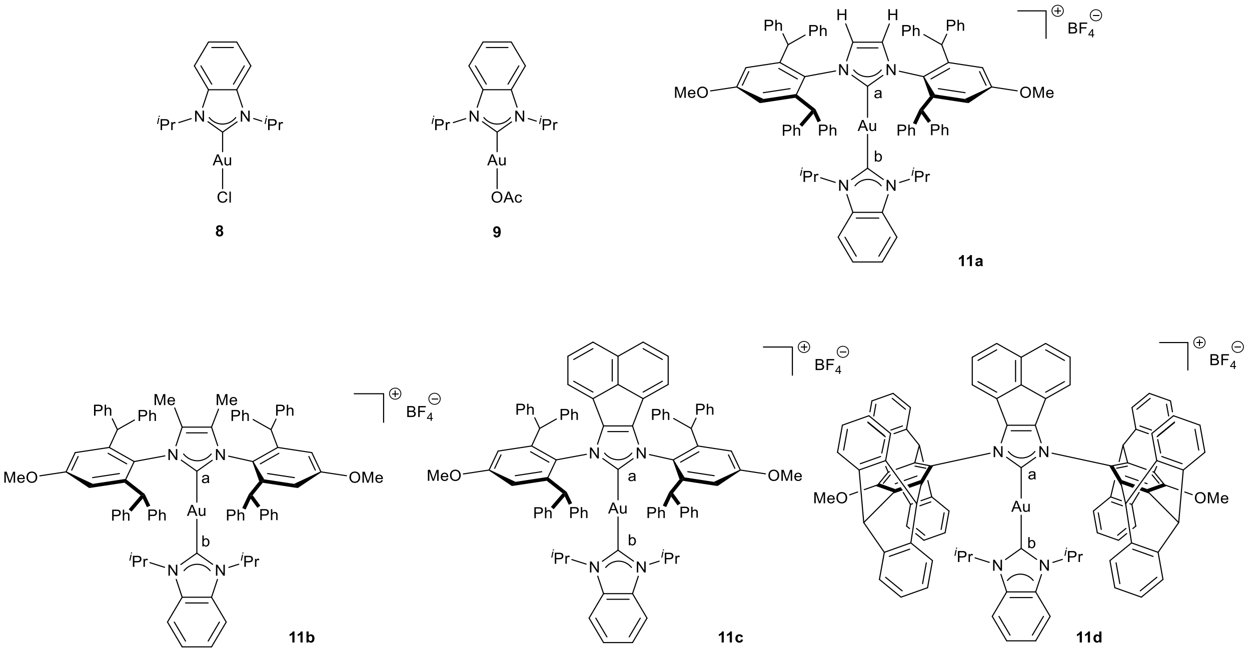

Scheme S2. Gold(I) complexes used in this studies.

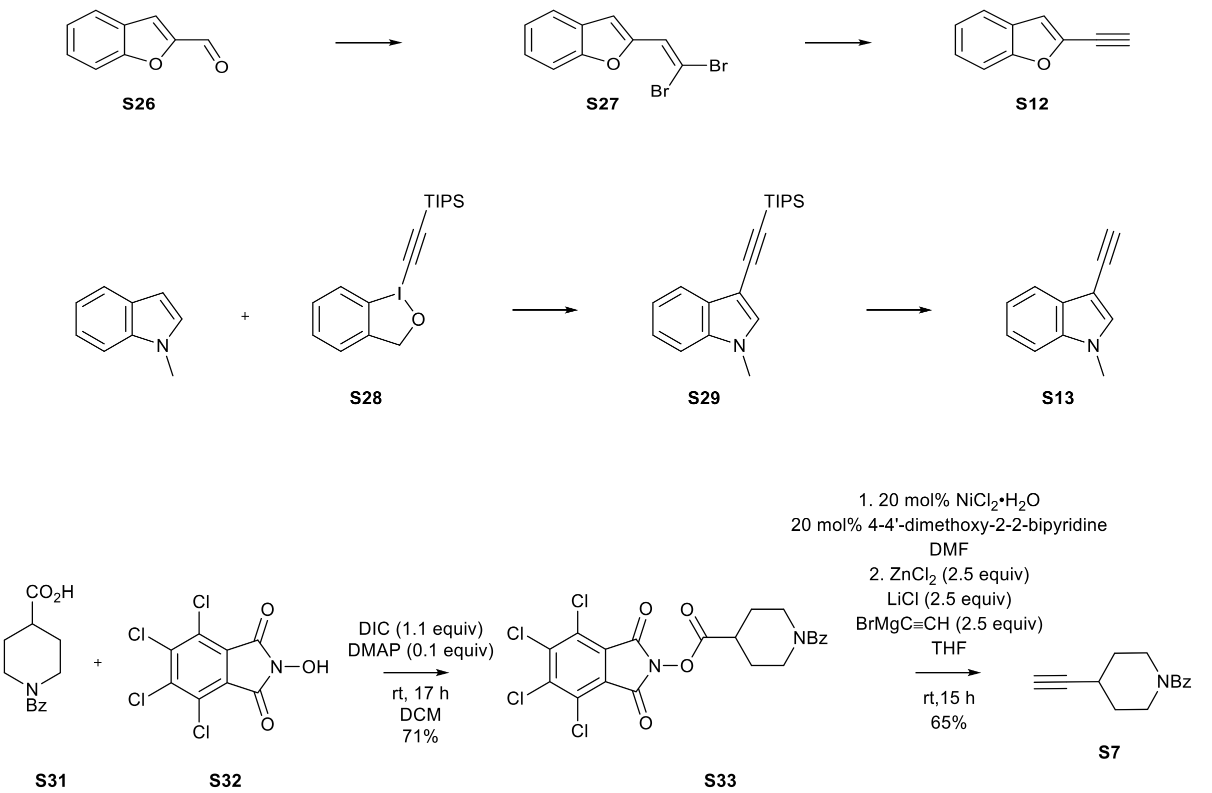

Scheme S3. Synthetic route to alkynes.

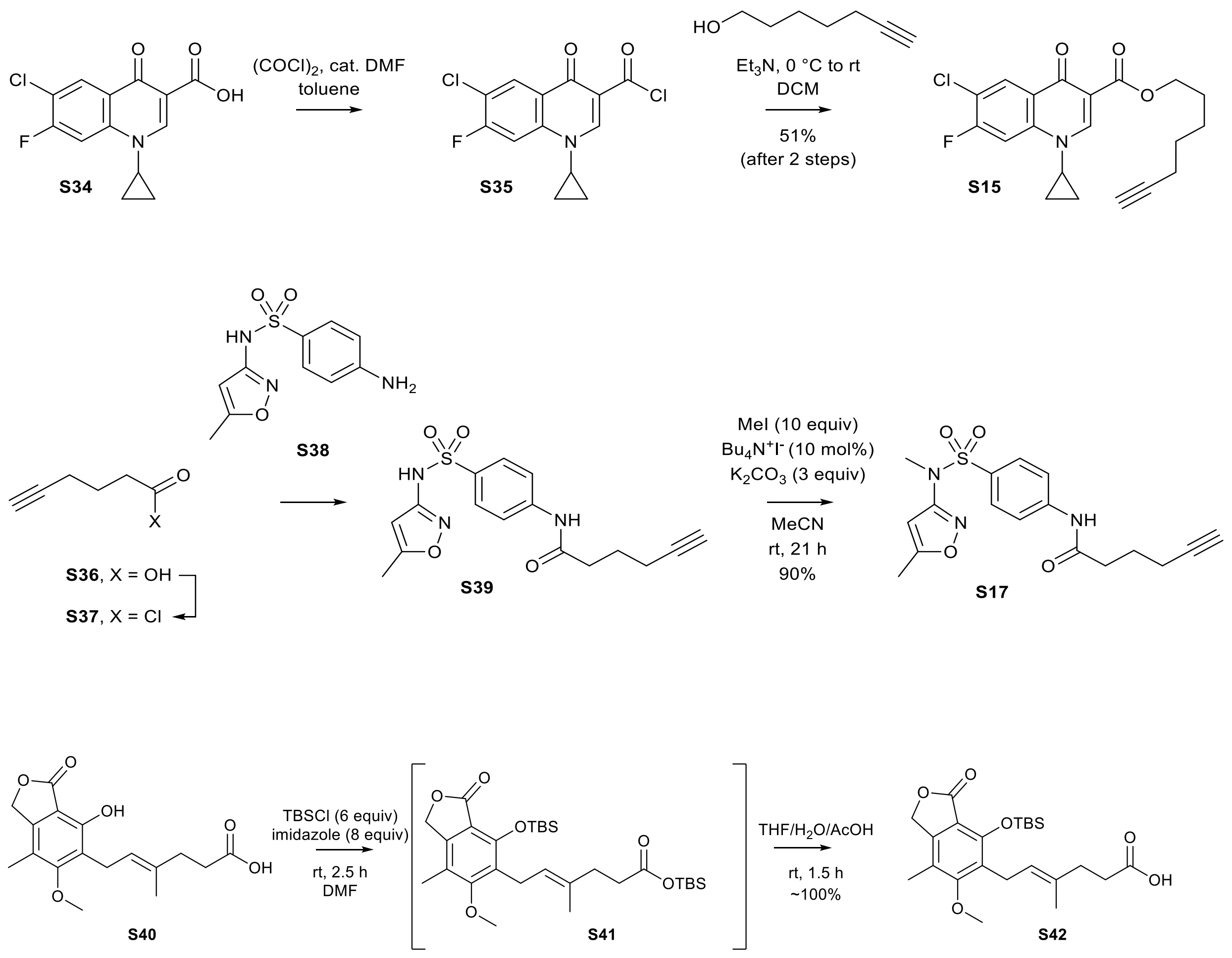

Scheme S3 (*cont.*). Synthetic route to alkynes.

2. Optimization studied leading to naphthyridines 3a.

Conversion and yield of model reaction were determined by GC analyses (naphthyridine **3a** and alkyne **1a**, Table S1) performed on Clarus PerkinElmer 680 gas chromatograph equipped with a split-mode capillary injection system and flame ionization detector using capillary column ZB-5HT Inferno (30 m × 0.25 mm x 0.25 µm, Phenomenex). Chromatography conditions: carrier gas – helium (1mL/min.), 100 °C/9 min. – 20°/min. – 280°C/5 min.; injector temperature: 270 °C (*const*); detector temperature 200 °C. Retention time of the respective compounds are as follow: alkyne **2a** 3.51 min.; aminophenone **1a** 6.37 min.; internal standard durene – 6.56 min.; naphthyridine **3a** – 10.02 min.

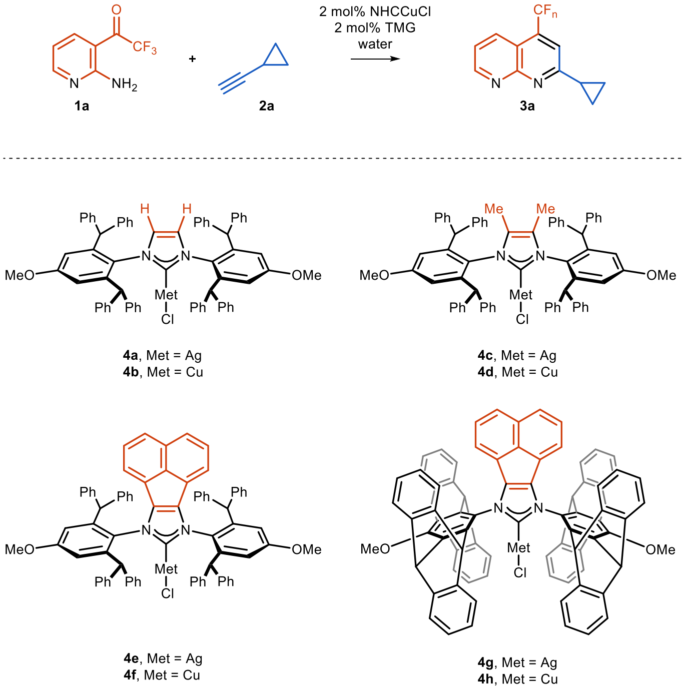

Scheme S4. NHC metal complexes used for the optimization studies.

Table S1. In-depth optimization studies of the synthesis of naphthyridine **3a**.

Entry	**2a** (equiv)	NHCCuCl	Time (h)	Temp. (°C)	Conv. (%)[a]	Yield (%)[b]
1	1.8	**4a** (2 mol%)	19	120	95%	4%
2	1.8	**4b** (2 mol%)	19	120	33%	4%
3	1.8	**4c** (2 mol%)	19	120	79%	13%
4	1.8	**4d** (2 mol%)	19	120	19%	1%
5	1.8	**4e** (2 mol%)	19	120	88%	10%
6	1.8	**4f** (2 mol%)	19	120	63%	**55%**
7	1.8	**4g** (2 mol%)	19	120	89%	2%
8	1.8	**4h** (2 mol%)	19	120	88%	**64%**
9	1.8	**4h** (2 mol%)	1	120	18%	< 1%
10	1.8	**4h** (2 mol%)	19	100	10%	< 1%
11	1.8	**4h** (2 mol%)	19	80	5%	1%
12	1.2	**4h** (2 mol%)	19	120	80%	50%
13	2.2	**4h** (2 mol%)	19	120	87%	21%
14	1.8	**4h** (1 mol%)	19	120	39%	5%
15	1.8	**4h** (4 mol%)	19	120	96%	62%
Complex **4a** - optimization studies						
16	1.8	**4a** (2 mol%)	1	120	42%	28%
17	1.8	**4a** (2 mol%)	19	100	52%	32%

18	1.8	**4a** (2 mol%)	19	80	70%	43%
Complex **4g -** optimization studies						
19	1.8	**4g** (2 mol%)	19	120	88%	64%
20	1.8	**4g** (2 mol%)	19	100	47%	5%
21	1.8	**4g** (2 mol%)	19	80	32%	3%

[a] conversion based on GC with durene as internal standard; [b] yield based on GC from calibraton curve

3. Single crystal X-ray diffraction

The crystals were embedded in the inert perfluoropolyalkylether (viscosity 1800cSt; ABCR GmbH) and mounted using Hampton Research Cryoloops. The crystals were flash cooled to 100.0(1) K in a nitrogen gas stream and kept at this temperature during the experiments. The X-ray data were collected on a SuperNova Agilent diffractometer using Mo*Kα* radiation (λ = 0.71073 Å) or Cu*Kα* radiation (λ = 1.54184 Å). The data were processed with *CrysAlisPro*.[1] Structures were solved by direct methods and refined using *SHELXL*[2] under *WinGX*.[3] The figures were prepared using *X-seed*.[4]

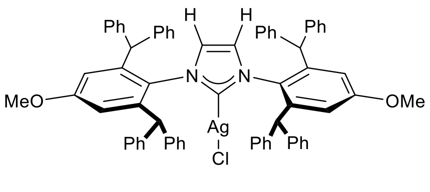

4a

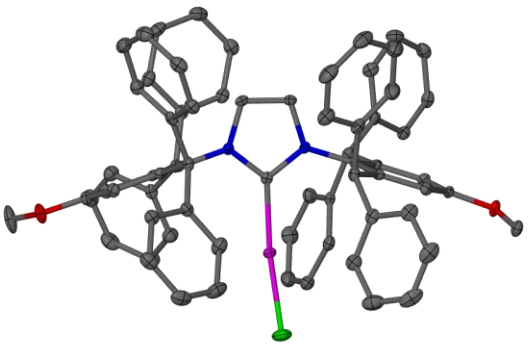

Fig. S2 Crystal data for 4a: $(C_{69}H_{56}N_2O_2AgCl)\cdot 2(CH_2Cl_2)$, *Mr* =1258.3, colorless prisms, orthorhombic, space group *Pbca*, *a* = 19.2621(2), *b* = 24.6506(5), *c* = 25.2385(3) Å, *V* = 11983.8(3) Å^3, *Z* = 8, ρ_{calc} = 1.39 g cm^{-3}, μ(MoKα) = 0.61 mm^{-1}, θ_{max} = 26.3°, 32921 reflections measured, 12236 unique, 760 parameters, *R* = 0.041, *wR* = 0.093 (*R* = 0.062, *wR* = 0.106 for all data), GooF = 1.01. CCDC 2143426.

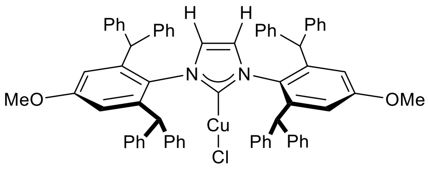

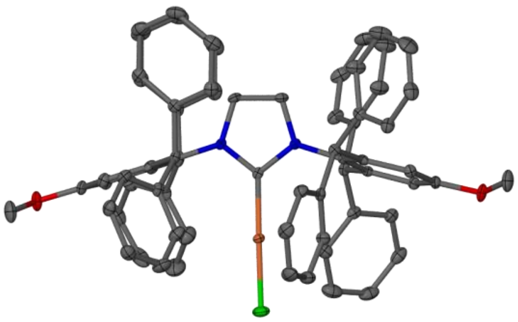

4b

Fig. S3 Crystal data for 4b: $(C_{69}H_{56}N_2O_2CuCl)\cdot 2(CH_2Cl_2)$, *Mr* =1214.0, colorless prisms, monoclinic, space group $P2_1/n$, a = 15.8854(3), b = 18.7151(2), c = 20.2401(2) Å, β = 94.392(1)°, V = 5999.6(1) Å^3, Z = 4, ρ_{calc} = 1.34 g cm^{-3}, μ(MoKα) = 0.63 mm^{-1}, θ_{max} = 26.3°, 47930 reflections measured, 12250 unique, 732 parameters, R = 0.047, wR = 0.118 (R = 0.059, wR = 0.126 for all data), GooF = 1.02. CCDC 2143425.

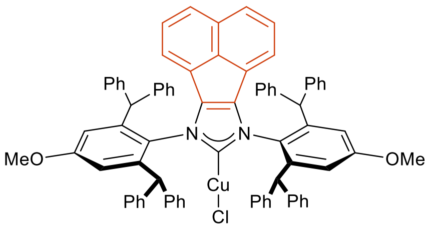

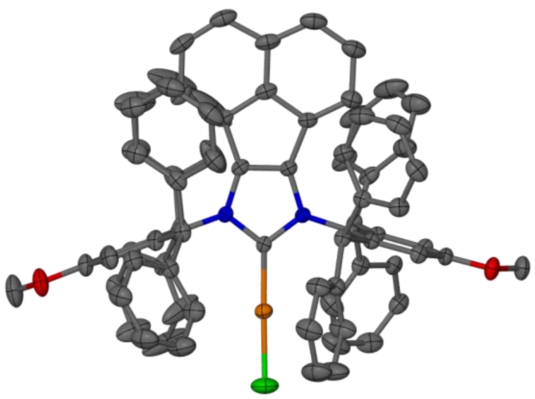

4e

Fig. S4 Crystal data for 4e: $(C_{79}H_{60}N_2O_2CuCl)\cdot 2(CH_2Cl_2)$, *Mr* =1338.1, yellow plates, orthorhombic, space group *Pbca*, a = 19.0302(2), b = 24.2764(2), c = 29.9917(4) Å, V = 13855.7(3) Å^3, Z = 8, ρ_{calc} = 1.28 g cm^{-3}, μ(CuKα) = 2.59 mm^{-1}, θ_{max} = 66.6°, 79445 reflections measured, 12219 unique, 850 parameters, R = 0.059, wR = 0.146 (R = 0.073, wR = 0.157 for all data), GooF = 1.01. CCDC 2143428.

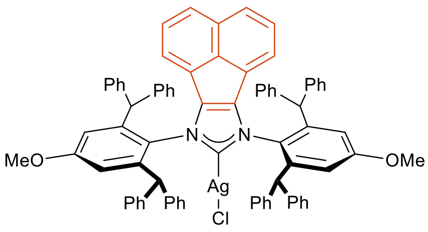

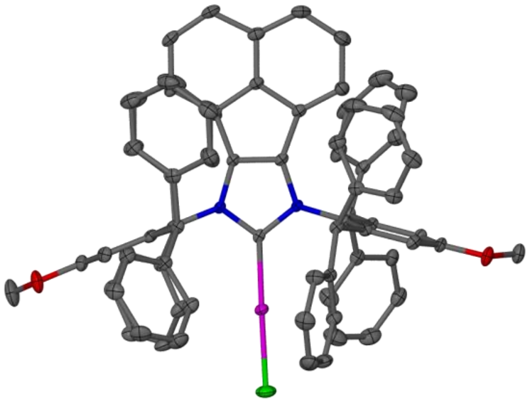

4f

Fig. S5 Crystal data for 4f: ($C_{79}H_{60}N_2O_2AgCl$)•3(CH_2Cl_2), *Mr* =1467.4, yellow prisms, orthorhombic, space group *Pbca*, a = 19.2448(1), b = 24.1491(2), c = 29.9815(4) Å, V = 13933.7(2) Å^3, Z = 8, ρ_{calc} = 1.40 g cm^{-3}, μ(MoKα) = 0.61mm^{-1}, θ_{max} = 27.5°, 209158 reflections measured, 15937 unique, 849 parameters, R = 0.041, wR = 0.097 (R = 0.055, wR = 0.107 for all data), GooF = 1.08. CCDC 2143427.

4. Computational details

All DFT calculations were performed with the Gaussian16 package.[5] The geometry optimizations were done without constraints via the spin-restricted Kohn-Sham (RKS) formalism and the BP86 functional of Becke and Perdew[6] with the Grimme D3 correction term to the electronic energy.[7] The split-valence basis set (Def2SVP keyword in Gaussian) was used for all non-metal atoms,[8] while for Cu and Ag atoms the small-core quasi-relativistic Stuttgart/Dresden effective core potential was applied, including an associated valence basis set (standard SDD keywords in Gaussian16).[9] In all cases, frequency calculations were carried out in order to approve the nature of the stationary points (minima without imaginary frequencies). Solvent effects were assessed based on the polarizable solvation model (SMD), variation of IEFPCM of Truhlar and co-workers,[10] using water as the solvent; employing the B3LYP, hybrid GGA functional of Becke-Lee, Parr, and Yang[11], and the triple-ζ plus one polarization function basis set proposed by Ahlrichs basis set (Def2TZVP).[8]

%V_{bur} Calculations: The buried volume calculations were performed with the SambVca package developed by Cavallo et al.[12] The radius of the sphere around the metal center was set to 3.5 Å, while for the atoms we adopted the Bondi radii scaled by 1.17, and a mesh of 0.1 Å was used to scan the sphere for buried voxels.

5. Computational results

The rate determining step has two approaches, either with the N or the O atoms bonded to the metal or interacting with it (the bigger the NHC the weaker the interaction with the N atom is). In Fig. S6 we reproduce both types for **4f**, being the latter less stable by 5.5 kcal/mol.

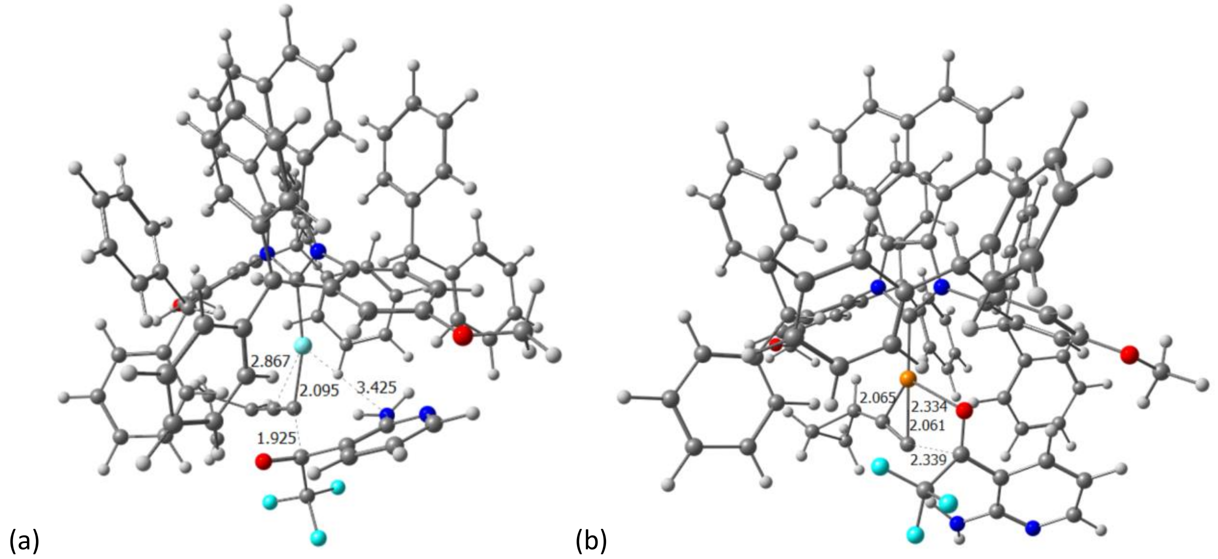

Fig. S6 Transition states of the C-C bond formation for species **4f**, with selected distances in Å, via the (a) N atom and the (b) O atom.

The results of the X-ray are due in part to the packaging. We just have to keep that in mind. However, since the discussion with catalysts with the chlorine ligand is certainly not the most real chemically speaking, as the NHC ligand where it will affect the most is in some intermediate/transition state where there will be other ligands bonded to the metal. Actually, in past studies, to simulate the "real" scenario different ligands were added to compare the behaviour of a series of NHC ligands. In particular 2 CO and 1 Cl ligands were used with iridium to reproduce how crowded is the metal sphere because of an NHC ligand.[12]

First, in Table S2 we have checked the differences between the $\%V_{Bur}$ of the NHC in the X-Rays and once optimized by DFT calculations. There is a difference of less than 1 % in any of the conformations, up to 3% for some particular case with argentum based complexes, and obviously higher for the X-Ray due to its packing. Actually, the correlation of the Pearson coefficient is 0.957.

Table S2. $\%V_{Bur}$ (X-Ray) vs $\%V_{Bur}$ (DFT optimized).

	$\%V_{bur}$ (DFT)	$\%V_{bur}$ (X-Ray)
**4a**	62.6	62.4
**4c**	65.3	65.3
**4e**	62.5	62.6
**4g**	50.8	49.9
**4b**	56.5	57.4
**4d**	58.4	61.7
**4f**	58.7	58.7

**4h**	48.7	48.6

The reactant TMG•HCl responsible of the deprotonation of the alkyne substrate seems too unstable as a single whole moiety, *i.e.* the $TMGH^+$ cation and Cl anion is 8.4 kcal/mol more stable. Actually, the Cl atom is dissociated in covalent Ru-Cl bonds,[13] thus, here with ionic nature even more. Anyway, to point out that the consequence is that DFT calculations find out lower barriers if instead of TMG•HCl, the separate species $TMGH^+$ and Cl^- are used.

The $\%V_{bur}$ was calculated for the chloride compounds (**4a-h**) and the disparity of values in Figure S1 with both metals, copper and argentum, is a hint that the series of NHC ligands are flexible, and thus, it is better its evaluation in the rate determining intermediate or transition state. However, the values are roughly 5% larger for copper than argentum.

In fact, the correlation of $\%V_{bur}$ with conversion and experimental yields did not yield any Pearson coefficient above 0.8, but slightly better per metal separately, except for argentum with the yield (R^2 = 0.541 (Cu), 0.647 (Ag)). The correlation with the conversion for copper goes up to R^2 = 0.773, but it fails for argentum (R^2 = 0.077). However, removing selectively the values of the more rigid **4e-g** species, in particular **4g** and **4h** we go up to R^2 = 0.887. And also removing E and F, to R^2 = 0.985. Analysing by metals, for copper, even though the agreement was good yet, removing **4e** goes up to 0.9997, but with just three points the statistical significance is still low. And since the same reasoning is not applicable to the silver series, no final judgment can be made.

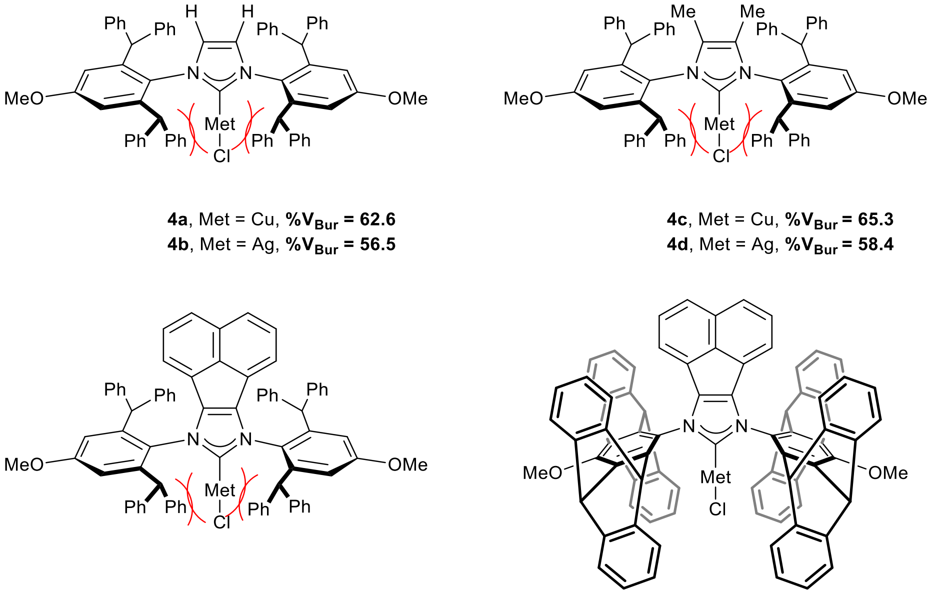

4a, Met = Cu, **%V$_{Bur}$ = 62.6**
4b, Met = Ag, **%V$_{Bur}$ = 56.5**

4c, Met = Cu, **%V$_{Bur}$ = 65.3**
4d, Met = Ag, **%V$_{Bur}$ = 58.4**

4e, Met = Cu, **%V$_{Bur}$ = 62.5**
4f, Met = Ag, **%V$_{Bur}$ = 58.7**

4g, Met = Cu, **%V$_{Bur}$ = 50.8**
4h, Met = Ag, **%V$_{Bur}$ = 48.7**

Fig. S7. $\%V_{bur}$ for the chloride catalysts.

The total $\%V_{bur}$, as well as the quadrant values, together with the steric maps centered on the metal are collected in Table S3.

6. Steric Maps

Table S3. $\%V_{bur}$ values (total and by quadrants), and steric maps of the xy plane (centered on the metal, z axis defined by the C_{NHC}, xz plane containing any of the N atoms of the imidazole ring; curves are given in Å).

4B

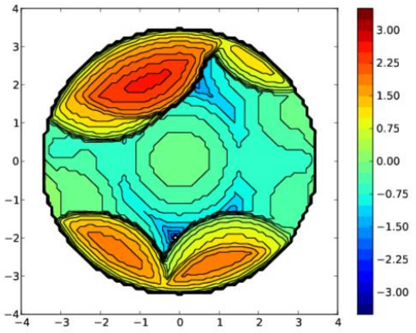

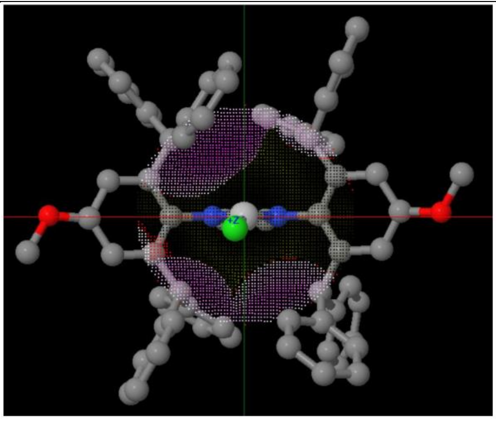

%V Buried	
56.5	
Quadrant	V b
SW	55.6
NW	71.1
NE	46.3
SE	53.2

4D

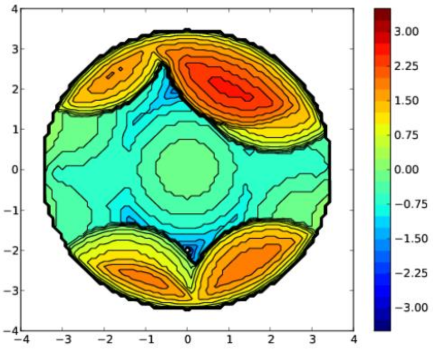

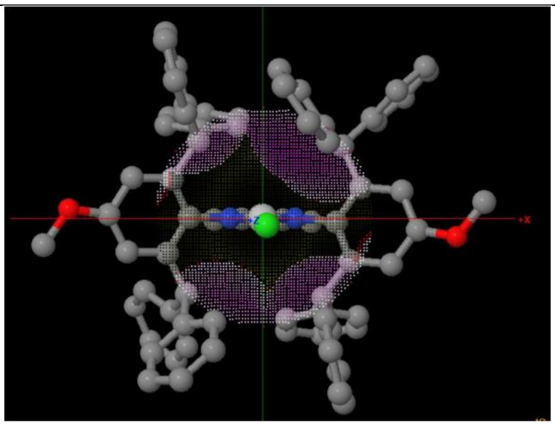

%V Buried	
58.4	
Quadrant	V b
SW	23.4
NW	23.8

NE	31.9
SE	25.8

4F

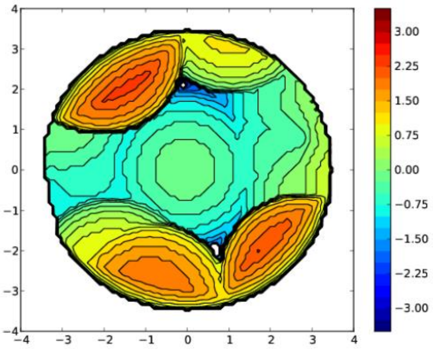

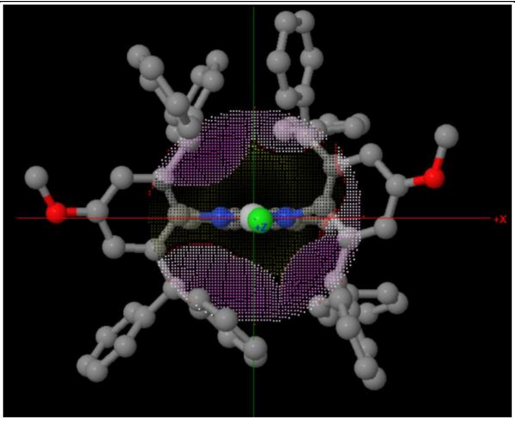

%V Buried	
58.7	
Quadrant	V b
SW	60.7
NW	63.1
NE	48.5
SE	62.3

4H

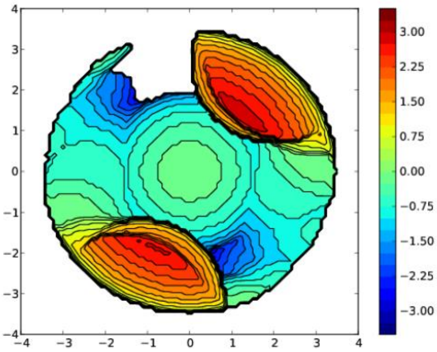

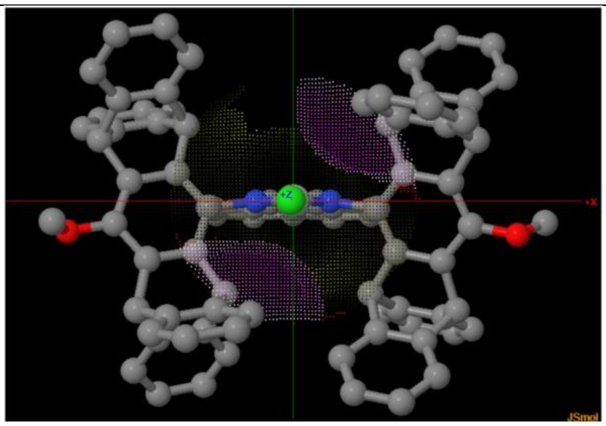

%V Buried	
48.7	
Quadrant	V b
SW	60.7
NW	63.1
NE	48.5
SE	62.3

4A

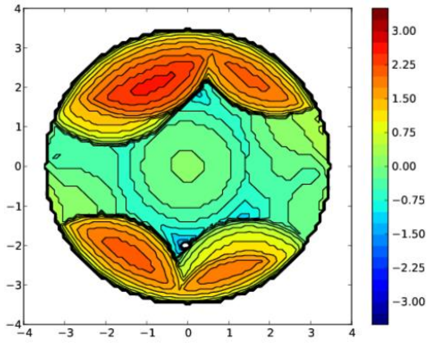

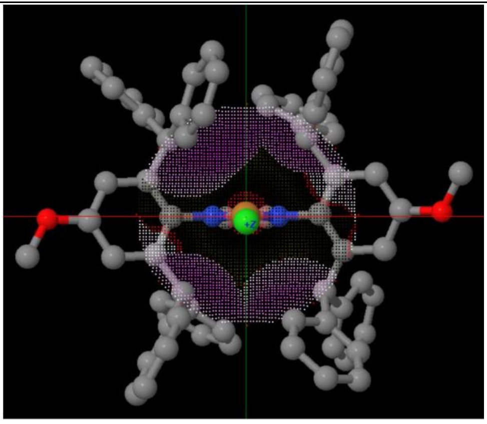

%V Buried	
62.6	
Quadrant	V b
SW	61.6
NW	70.9
NE	62.0
SE	55.7

4C

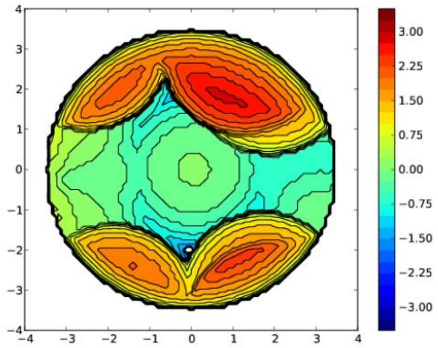

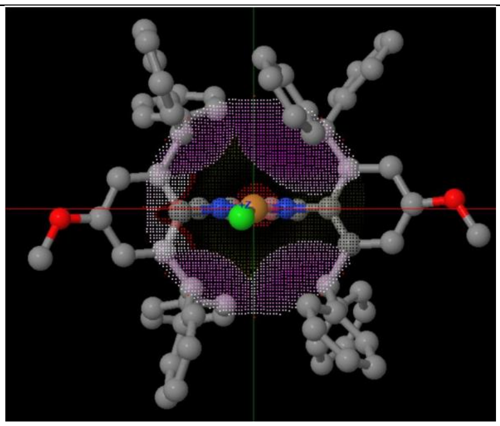

%V Buried	
65.3	
Quadrant	V b
SW	59.9
NW	63.5
NE	76.8
SE	60.8

4E

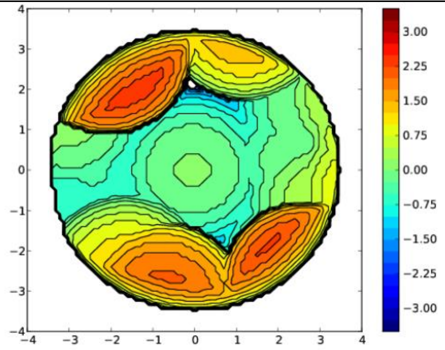

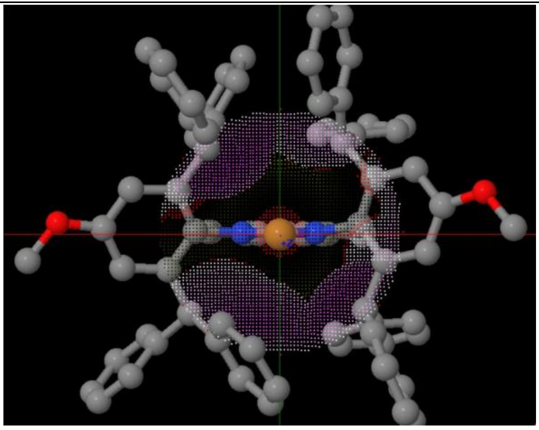

%V Buried	
62.5	
Quadrant	V b
SW	63.5
NW	66.2
NE	54.3
SE	65.8

4G

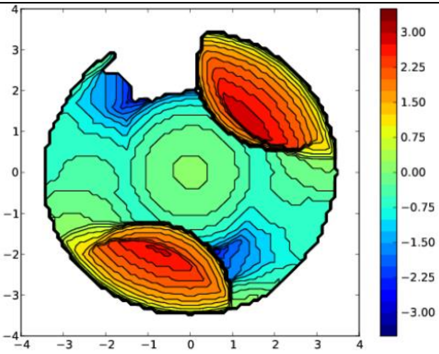

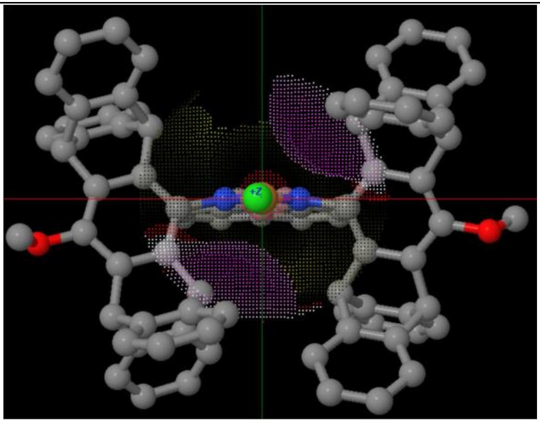

11.

**%V Buried**	
**50.8**	
**Quadrant**	**V b**
**SW**	66.9
**NW**	28.1
**NE**	67.1
**SE**	41.0

Table S4. xyz coordinates and absolute energies (in a.u.) of all computed species.

Ag geometries			
**4B Cat**			
Ag1RX-Clbb SCF Done: -3528.38668775 A.U.			
C	0.252654	7.176281	-1.122036
H	0.989955	7.315708	-0.298541
H	-0.164732	8.161995	-1.401746
H	0.776570	6.739667	-2.002333
Ag	0.123344	0.177511	-2.011181
Cl	0.580503	0.613922	-4.251701
O	-0.852572	6.377584	-0.714664
O	0.803985	-6.163770	-1.359679
N	-0.200406	1.065947	0.905658
C	0.265641	-2.388431	0.278008
C	-0.352121	2.433631	0.483031
C	-0.622116	5.090531	-0.339274
N	0.098215	-1.070465	0.826452
C	0.803708	3.163825	0.128063
C	-0.120371	0.627776	2.230853
H	-0.168888	1.323528	3.072840
C	0.058782	-0.730083	2.180619
H	0.145620	-1.482386	2.969140
C	-2.653289	1.747066	-2.026900
H	-1.805690	2.448180	-2.051846
C	2.793351	-2.093863	0.515439
H	2.419027	-1.076271	0.754367
C	-1.649333	2.987934	0.370788
C	-3.992842	2.755259	1.327158
C	-1.767021	4.328535	-0.019263
H	-2.754891	4.801516	-0.114810
C	-0.058793	0.027211	0.025647
C	0.659624	4.499575	-0.283091
H	1.562428	5.055798	-0.567926
C	-3.347262	-3.085160	-0.800963
C	1.697088	-4.196365	-0.438976
H	2.687906	-4.643912	-0.597119
C	-5.996074	4.016469	2.879898
H	-6.777924	4.504977	3.482754
C	-4.431302	0.610395	-0.833867
H	-5.004255	0.437221	0.091223
C	-3.341597	-1.318934	2.076434
H	-3.699240	-0.548793	1.379191
C	2.172463	2.487873	0.212084
H	2.023674	1.461214	-0.185142
C	-3.031466	1.089563	-3.214915
H	-2.441714	1.261775	-4.128502
C	-2.601937	-2.405014	1.566711

C	-2.264514	-2.461180	0.075667
H	-2.188393	-1.404542	-0.268098
C	-2.876503	2.092572	0.526500
H	-2.551069	1.218101	1.129298
C	-3.989373	2.645929	2.734048
H	-3.193767	2.059063	3.223592
C	3.746844	-1.916378	-0.665877
C	-3.334065	1.503934	-0.817575
C	-0.729982	-4.375951	-0.686695
H	-1.628185	-4.918414	-1.008834
C	4.738834	-2.868912	-0.985738
H	4.888628	-3.740949	-0.329046
C	3.253539	-3.664262	4.008842
H	2.653570	-4.237012	4.734176
C	-4.435711	-3.805626	-0.274924
H	-4.526043	-3.932758	0.815136
C	-0.886448	-3.093652	-0.135955
C	5.550548	-2.700776	-2.120245
H	6.320116	-3.453341	-2.355801
C	-6.009920	4.129193	1.478304
H	-6.804202	4.707205	0.979469
C	-5.411454	-4.351754	-1.131088
H	-6.261490	-4.907937	-0.704327
C	-5.017531	3.499628	0.706995
H	-5.042920	3.567583	-0.392305
C	3.438644	-2.581849	1.817214
C	4.571982	-3.292607	4.322811
H	5.012426	-3.570576	5.293357
C	-3.249538	-2.920501	-2.201380
H	-2.408861	-2.341138	-2.617592
C	1.567221	-2.913977	0.111289
C	3.209280	3.122949	-0.709307
C	-4.214877	-3.471756	-3.055759
H	-4.124863	-3.328248	-4.144256
C	-2.161807	-3.399073	2.465605
H	-1.582200	-4.254923	2.084417
C	-4.811577	-0.046068	-2.013145
H	-5.646609	-0.763735	-1.995160
C	3.110253	2.889083	-2.100218
H	2.301225	2.253033	-2.502296
C	5.067520	4.287917	-2.492935
H	5.795596	4.737596	-3.186929
C	-3.154860	-2.193205	4.339573
H	-3.359786	-2.106716	5.418467
C	2.695377	-3.315485	2.766679
H	1.665378	-3.624202	2.526920
C	4.762426	-2.213720	2.144352
H	5.358402	-1.640369	1.417644
C	3.599563	-0.792479	-1.504241
H	2.825681	-0.042081	-1.277262
C	-2.434874	-3.293899	3.841480
H	-2.077679	-4.076439	4.530118

C	4.034459	3.466926	-2.984371
H	3.944041	3.268726	-4.064276
C	2.619836	2.301529	1.661781
C	5.325431	-2.568048	3.382009
H	6.361383	-2.271919	3.612598
C	-5.303700	-4.188287	-2.522172
H	-6.067523	-4.615405	-3.191390
C	3.347677	1.802519	4.352102
H	3.616997	1.597155	5.400333
C	4.242996	3.946667	-0.223760
H	4.328147	4.124558	0.860028
C	5.167957	4.527281	-1.111799
H	5.975701	5.165637	-0.718942
C	-4.982369	3.269611	3.507321
H	-4.967559	3.169978	4.604559
C	0.560785	-4.932116	-0.834847
C	2.179430	3.154566	2.694308
H	1.525335	4.006140	2.448622
C	-3.615254	-1.206770	3.448407
H	-4.185064	-0.339531	3.818759
C	3.810458	0.958156	3.326070
H	4.433909	0.082673	3.563305
C	-4.105167	0.186898	-3.208321
H	-4.386635	-0.348285	-4.128550
C	4.405636	-0.619858	-2.640244
H	4.254648	0.265598	-3.276163
C	3.459066	1.215855	1.991987
H	3.834493	0.561506	1.188558
C	5.384720	-1.578203	-2.952695
H	6.020326	-1.450985	-3.843255
C	2.543058	2.909362	4.031066
H	2.186331	3.583590	4.826433
C	-0.295499	-6.929583	-1.837515
H	-1.010643	-7.184307	-1.021803
H	0.132582	-7.865013	-2.244777
H	-0.848770	-6.400173	-2.646209

4B a

Ag1RX-ALKI SCF Done: -3261.54451907 A.U.

Ag	0.104467	-0.224546	1.716801
N	-0.037924	-1.013245	-1.232602
C	2.758892	-0.581499	-1.615413
H	1.959408	0.178509	-1.496238
C	2.236632	-1.884634	-1.016058
C	0.840908	-2.102053	-0.887476
C	-0.369661	-0.069603	-0.293776
C	0.324002	-3.264083	-0.283563
C	-1.186278	-3.437414	-0.114128
H	-1.624621	-2.421466	-0.004761
C	3.111359	-2.878675	-0.563482
C	2.613692	-4.072713	0.003771

C	1.223379	-4.260023	0.142488
C	-0.397355	-0.610126	-2.517323
C	-0.957727	0.635450	-2.385446
N	-0.928926	0.946241	-1.020530
C	-1.367563	2.191066	-0.441874
C	-0.403890	3.134562	0.011449
C	-2.755396	2.427826	-0.310041
C	-0.869116	4.336118	0.561919
C	-3.188195	3.644556	0.248034
C	-2.248833	4.603679	0.676448
C	1.104156	2.883986	-0.111898
H	1.260022	1.792823	0.037764
C	-3.767026	1.336994	-0.678616
H	-3.441575	0.898655	-1.647614
C	-3.766813	0.182800	0.330664
C	-3.219815	0.301618	1.623006
C	-4.355362	-1.044578	-0.044664
C	-3.242860	-0.788215	2.514935
H	-2.748322	1.247056	1.932752
C	-4.381332	-2.131382	0.842573
C	-3.822490	-2.006781	2.127749
H	-2.774762	-0.686188	3.506161
H	-3.814466	-2.869185	2.811308
C	-5.165818	1.908719	-0.916643
C	-5.462497	2.533611	-2.146901
C	-6.157108	1.883891	0.085823
C	-6.717046	3.122948	-2.371828
H	-4.691476	2.564699	-2.935096
C	-7.413205	2.476760	-0.134583
C	-7.697735	3.098063	-1.362981
H	-6.932492	3.602069	-3.340351
H	-8.176570	2.448109	0.659377
H	-8.683699	3.557323	-1.537501
C	1.885821	3.562026	1.018682
C	2.495201	4.823579	0.853404
C	1.968500	2.930186	2.276496
C	3.179922	5.433077	1.919889
C	2.651093	3.536200	3.343052
H	1.498317	1.945464	2.438443
C	3.263424	4.790457	3.167809
H	3.653840	6.416843	1.771696
H	2.700735	3.012130	4.310854
H	3.804288	5.267226	4.001073
C	1.664016	3.199438	-1.503213
C	3.053682	3.051766	-1.721719
C	0.857538	3.622182	-2.579178
C	3.617384	3.318213	-2.977155
H	3.698717	2.733712	-0.889453
C	1.422155	3.888745	-3.841940
C	2.804069	3.740481	-4.045363
H	4.700301	3.180571	-3.121228
H	0.774063	4.221778	-4.668583

H	3.246200	3.945379	-5.032983
C	-1.567263	-4.159354	1.179575
C	-0.975144	-3.732891	2.391373
C	-2.571451	-5.145079	1.231471
C	-1.378282	-4.276086	3.619702
H	-0.204949	-2.943034	2.380521
C	-2.977854	-5.692309	2.463723
C	-2.384003	-5.260925	3.661913
H	-0.910400	-3.911787	4.547932
H	-3.769698	-6.458326	2.483454
H	-2.705467	-5.686803	4.625778
C	-1.756199	-4.019403	-1.406594
C	-2.579676	-3.228802	-2.233140
C	-1.424448	-5.323862	-1.833948
C	-3.070643	-3.727835	-3.452680
H	-2.830664	-2.207201	-1.910061
C	-1.916867	-5.828071	-3.048089
C	-2.742363	-5.030849	-3.863355
H	-3.712758	-3.093355	-4.084681
H	-1.650544	-6.849286	-3.364539
H	-3.125171	-5.425501	-4.817949
C	3.930535	0.007268	-0.824791
C	3.721426	0.300683	0.542560
C	5.147751	0.382204	-1.423417
C	4.689727	0.992778	1.282899
C	6.127554	1.064024	-0.676408
H	5.316836	0.171202	-2.490314
C	5.897212	1.383284	0.672620
H	4.484823	1.246535	2.334133
H	7.071768	1.361229	-1.160520
H	6.654400	1.937405	1.249393
C	2.986005	-0.692281	-3.122289
C	2.460515	0.304041	-3.970689
C	3.726585	-1.747075	-3.696483
C	2.677970	0.258638	-5.357427
H	1.874022	1.125909	-3.533814
C	3.945053	-1.796285	-5.084032
C	3.422757	-0.792530	-5.920264
H	2.264060	1.055331	-5.996002
H	4.526139	-2.627106	-5.515391
H	3.594387	-0.832759	-7.007803
O	3.551999	-4.969725	0.413793
C	3.117699	-6.158696	1.061695
H	2.548648	-5.938783	1.993643
H	2.484214	-6.787652	0.394514
H	4.033595	-6.722157	1.322387
O	-2.577741	5.806783	1.220271
C	-3.954310	6.128621	1.375931
H	-3.987435	7.140100	1.823069
H	-4.490053	6.144600	0.399222
H	-4.473039	5.414789	2.055928
C	0.858061	-0.307118	3.584266

C	1.485139	-0.205341	4.652670
C	2.251417	-0.074765	5.870409
H	1.662417	0.141148	6.781935
C	3.644690	0.562579	5.834843
C	3.508626	-0.917521	6.095444
H	3.787662	-1.612942	5.287794
H	3.696141	-1.293025	7.115075
H	4.015823	0.880933	4.847227
H	3.927322	1.225852	6.669191
H	-1.342001	1.337660	-3.130420
H	-0.192541	-1.227904	-3.395768
H	2.776130	0.003172	1.030794
H	4.134074	-2.540959	-3.051784
H	2.442854	5.328693	-0.124094
H	-0.223776	3.761344	-2.424255
H	-4.817590	-3.090692	0.523446
H	-4.806153	-1.141450	-1.046298
H	-5.937075	1.391401	1.045927
H	-0.769960	-5.947959	-1.205077
H	-3.057487	-5.477073	0.301435
H	4.199909	-2.724010	-0.595947
H	0.816855	-5.152753	0.635608
H	-0.160004	5.091365	0.928773
H	-4.266832	3.818976	0.349171

4B a+b

Ag1RX-OCF3ALKIPh--2Npre SCF Done: -4015.07307314 A.U.

Ag	-0.583746	0.026422	-1.033653
N	1.670138	0.219269	1.224068
C	1.358668	-2.462016	2.111429
H	0.626759	-1.632064	2.187489
C	2.306398	-2.122815	0.962537
C	2.516401	-0.766818	0.612468
C	0.442154	0.512252	0.698385
C	3.441440	-0.371945	-0.370392
C	3.610464	1.119916	-0.669079
H	2.596915	1.575039	-0.597082
C	3.044663	-3.099571	0.283753
C	4.002201	-2.727544	-0.686000
C	4.206313	-1.368925	-1.005435
C	1.853760	0.814474	2.471876
C	0.692720	1.490879	2.748829
N	-0.152995	1.290990	1.654582
C	-1.487753	1.813633	1.508382
C	-2.604761	0.956591	1.723534
C	-1.643211	3.145353	1.066493
C	-3.881861	1.501136	1.550491
C	-2.943578	3.660087	0.900395
C	-4.061927	2.839776	1.142202
C	-2.420744	-0.531793	2.051606
H	-1.546818	-0.869702	1.451004

C	-0.428733	3.970143	0.626073
H	0.434427	3.653845	1.250231
C	-0.036455	3.672566	-0.828246
C	-0.946931	3.146649	-1.764960
C	1.281495	3.954197	-1.256032
C	-0.548637	2.896816	-3.092919
H	-1.973074	2.893295	-1.461692
C	1.684842	3.697253	-2.575095
C	0.767153	3.162455	-3.499350
H	-1.279050	2.455952	-3.788328
H	1.093952	2.949659	-4.529474
C	-0.629353	5.462677	0.898010
C	-0.512937	5.941636	2.220724
C	-0.978693	6.370476	-0.122092
C	-0.741344	7.293599	2.520425
H	-0.245918	5.236747	3.026070
C	-1.211993	7.725811	0.175625
C	-1.094689	8.191896	1.495957
H	-0.642199	7.650263	3.558190
H	-1.482830	8.422667	-0.633704
H	-1.273097	9.253918	1.727683
C	-3.595050	-1.382481	1.563507
C	-4.746512	-1.576444	2.357600
C	-3.546861	-1.968408	0.284713
C	-5.831769	-2.323093	1.867842
C	-4.621948	-2.729943	-0.202366
H	-2.663687	-1.829670	-0.354381
C	-5.772433	-2.901457	0.585556
H	-6.726809	-2.461589	2.495524
H	-4.528957	-3.181692	-1.201489
H	-6.621804	-3.491917	0.206239
C	-2.063859	-0.837533	3.507403
C	-1.844845	-2.188422	3.866313
C	-1.952866	0.152672	4.502253
C	-1.522623	-2.536588	5.185051
H	-1.934548	-2.971062	3.096566
C	-1.626359	-0.195753	5.828032
C	-1.413351	-1.540372	6.174306
H	-1.339707	-3.593029	5.435501
H	-1.547824	0.591572	6.595087
H	-1.155308	-1.813108	7.209630
C	4.063235	1.416537	-2.096386
C	3.357239	0.809558	-3.157289
C	5.086462	2.334258	-2.402806
C	3.674328	1.091818	-4.492486
H	2.551389	0.093093	-2.939429
C	5.390749	2.641052	-3.743679
C	4.689389	2.020558	-4.792128
H	3.123243	0.562478	-5.286267
H	6.188403	3.367691	-3.967112
H	4.938430	2.254039	-5.839680
C	4.460732	1.766277	0.423591

C	3.964871	2.857642	1.164381
C	5.746481	1.271725	0.731090
C	4.732400	3.450353	2.182165
H	2.953431	3.233105	0.947476
C	6.518439	1.860835	1.745425
C	6.013920	2.953540	2.475455
H	4.323181	4.300668	2.750695
H	7.519623	1.461195	1.972781
H	6.618083	3.412751	3.273881
C	0.482753	-3.690264	1.866143
C	-0.365858	-3.686894	0.735588
C	0.383213	-4.751967	2.785949
C	-1.308762	-4.706878	0.540075
C	-0.555607	-5.782064	2.587024
H	1.024097	-4.760534	3.680670
C	-1.408144	-5.758310	1.470817
H	-1.969125	-4.653663	-0.337151
H	-0.626269	-6.601832	3.320090
H	-2.154613	-6.555199	1.325441
C	2.113995	-2.460523	3.442108
C	1.623136	-1.692414	4.518034
C	3.288078	-3.219317	3.634871
C	2.281598	-1.686964	5.759081
H	0.712255	-1.091674	4.379557
C	3.948733	-3.216867	4.874908
C	3.447792	-2.449713	5.942867
H	1.872210	-1.083866	6.585115
H	4.863362	-3.816780	5.007125
H	3.967212	-2.446399	6.914442
O	4.673451	-3.757482	-1.278547
C	5.751979	-3.453293	-2.154359
H	5.415428	-2.894832	-3.056767
H	6.541320	-2.857646	-1.641530
H	6.177043	-4.424016	-2.472133
O	-5.354945	3.235392	0.979466
C	-5.605696	4.548254	0.493994
H	-6.705277	4.647963	0.423129
H	-5.214396	5.329440	1.185085
H	-5.160950	4.706546	-0.515037
C	-2.155868	-0.057710	-2.334976
C	-3.336107	0.295028	-2.530787
C	-4.706084	0.713500	-2.675657
H	-4.865309	1.600359	-3.317378
C	-5.643515	0.597266	-1.463584
C	-5.859536	-0.293463	-2.653628
H	-5.584061	-1.353281	-2.556247
H	-6.707473	-0.091662	-3.328409
H	-5.209392	0.135123	-0.563087
H	-6.329837	1.436863	-1.269178
O	-2.225054	-3.339733	-2.201949
C	-1.750907	-2.756062	-3.175220
C	-0.282966	-2.623641	-3.377745

C	0.435737	-1.588711	-4.085763
C	0.495605	-3.647037	-2.787065
N	1.765552	-1.704909	-4.343199
C	1.874747	-3.714993	-2.996769
C	2.443266	-2.733076	-3.830675
H	-0.025380	-4.404238	-2.181756
H	2.495892	-4.504185	-2.551545
H	3.517533	-2.773930	-4.089125
N	-0.142704	-0.422799	-4.479255
H	-0.997391	-0.130497	-3.965961
H	0.514767	0.313583	-4.745822
C	-2.734526	-2.557724	-4.380291
F	-2.562207	-1.458707	-5.139082
F	-2.556367	-3.639418	-5.195683
F	-4.013566	-2.574641	-3.955622
H	0.384121	2.073739	3.620615
H	2.775970	0.684183	3.043958
H	-0.305972	-2.861322	0.004986
H	3.692063	-3.816737	2.803226
H	-4.785081	-1.144116	3.370678
H	-2.140258	1.206125	4.239265
H	2.722957	3.894877	-2.883133
H	2.000680	4.393281	-0.545948
H	-1.062370	6.008382	-1.158924
H	6.138565	0.408292	0.170528
H	5.641291	2.824955	-1.588502
H	2.881740	-4.167933	0.488614
H	4.916991	-1.068141	-1.786530
H	-4.777231	0.881201	1.691760
H	-3.054998	4.695203	0.552046
4B a+b→c			
Ag1RX-OCF3ALKIPh--2Nall SCF Done: -4015.06421814 A.U.			
Ag	0.550408	0.136629	1.030979
N	-1.677936	-0.012961	-1.233462
C	-1.567690	-2.851355	-1.470454
H	-0.837595	-2.079137	-1.786026
C	-2.528670	-2.179776	-0.488418
C	-2.603930	-0.767020	-0.434175
C	-0.437469	0.335093	-0.773769
C	-3.505435	-0.085444	0.407922
C	-3.479357	1.444356	0.423981
H	-2.404230	1.731227	0.366102
C	-3.385691	-2.914914	0.341153
C	-4.343778	-2.257567	1.141935
C	-4.410121	-0.848889	1.165916
C	-1.828499	0.285931	-2.588203
C	-0.637273	0.826741	-2.998802
N	0.192607	0.846411	-1.874915
C	1.546861	1.331770	-1.841348
C	2.623568	0.407967	-1.951597

C	1.759457	2.708523	-1.618892
C	3.921377	0.931680	-1.962657
C	3.078320	3.202457	-1.630314
C	4.156506	2.316353	-1.818469
C	2.364832	-1.105676	-1.948215
H	1.503433	-1.260424	-1.259931
C	0.593710	3.611012	-1.203323
H	-0.327362	3.181908	-1.650164
C	0.378639	3.596829	0.319068
C	1.397399	3.241432	1.226350
C	-0.881196	3.971777	0.840078
C	1.168606	3.270190	2.615170
H	2.377051	2.911157	0.851046
C	-1.119294	3.984923	2.223378
C	-0.088228	3.640944	3.117625
H	1.974163	2.955003	3.296482
H	-0.274312	3.649846	4.203595
C	0.725559	5.023587	-1.771674
C	0.344183	5.265917	-3.108652
C	1.258851	6.087031	-1.015365
C	0.495367	6.538989	-3.680805
H	-0.074003	4.438317	-3.706400
C	1.416594	7.362414	-1.587497
C	1.036251	7.592777	-2.920670
H	0.187580	6.712011	-4.724496
H	1.833054	8.183856	-0.982732
H	1.154226	8.593199	-3.366285
C	3.517551	-1.895392	-1.325462
C	4.648123	-2.272531	-2.082985
C	3.468820	-2.242678	0.038047
C	5.715809	-2.955625	-1.475899
C	4.528818	-2.934488	0.648603
H	2.602973	-1.956340	0.649821
C	5.660011	-3.285379	-0.107593
H	6.594994	-3.238791	-2.076919
H	4.441069	-3.174328	1.719625
H	6.498439	-3.822389	0.364469
C	1.935180	-1.698748	-3.290133
C	1.606734	-3.073861	-3.328818
C	1.860512	-0.950472	-4.480083
C	1.214934	-3.682570	-4.528343
H	1.661920	-3.667925	-2.402896
C	1.462213	-1.560690	-5.686373
C	1.142214	-2.928024	-5.715089
H	0.946511	-4.750329	-4.531077
H	1.412891	-0.962050	-6.610346
H	0.829081	-3.405571	-6.656794
C	-3.966091	2.112770	1.711872
C	-3.632566	1.561905	2.971133
C	-4.621589	3.361408	1.676440
C	-3.927274	2.255677	4.156487
H	-3.145543	0.571669	3.041102

C	-4.915156	4.055758	2.864875
C	-4.562865	3.510436	4.111271
H	-3.654580	1.803654	5.124259
H	-5.420264	5.033521	2.811945
H	-4.789409	4.055674	5.041261
C	-4.147316	1.985032	-0.840839
C	-3.449456	2.840871	-1.715873
C	-5.473446	1.627675	-1.163803
C	-4.055847	3.336839	-2.882811
H	-2.406277	3.105698	-1.486199
C	-6.084851	2.118171	-2.328947
C	-5.377931	2.975955	-3.193275
H	-3.487836	4.002779	-3.551780
H	-7.120029	1.826381	-2.567649
H	-5.857664	3.358360	-4.108152
C	-0.680881	-3.928195	-0.841040
C	0.089778	-3.573546	0.288893
C	-0.474026	-5.189666	-1.432528
C	1.075711	-4.434539	0.792841
C	0.499880	-6.066300	-0.916983
H	-1.053256	-5.476356	-2.323476
C	1.285078	-5.686374	0.184765
H	1.690259	-4.099159	1.643993
H	0.655894	-7.046490	-1.395957
H	2.066682	-6.360744	0.568901
C	-2.292462	-3.257918	-2.754229
C	-1.862790	-2.724905	-3.987201
C	-3.377223	-4.161629	-2.754403
C	-2.494021	-3.084697	-5.190165
H	-1.010524	-2.028763	-4.004428
C	-4.010079	-4.524703	-3.954690
C	-3.571203	-3.986480	-5.178751
H	-2.129533	-2.661558	-6.139777
H	-4.852808	-5.234147	-3.933901
H	-4.068291	-4.271693	-6.119675
O	-5.150627	-3.067065	1.888190
C	-6.132116	-2.462611	2.722670
H	-5.671347	-1.804359	3.494626
H	-6.865410	-1.866543	2.132806
H	-6.663464	-3.292225	3.226049
O	5.467329	2.692723	-1.826683
C	5.778155	4.068667	-1.646087
H	6.880506	4.147244	-1.697433
H	5.330490	4.703004	-2.444713
H	5.433599	4.444475	-0.655343
C	2.166215	-0.133751	2.348753
C	3.266070	0.455998	2.210085
C	4.555533	1.054465	2.054522
H	4.625718	2.128978	2.309154
C	5.509548	0.560717	0.954812
C	5.804644	0.213282	2.379975
H	5.608635	-0.818308	2.710948

H	6.618143	0.732695	2.911701
H	5.126678	-0.242584	0.308864
H	6.103125	1.331208	0.437592
O	2.608989	-2.644142	3.221328
C	1.897712	-1.698115	3.642662
C	0.377219	-1.910479	3.703192
C	-0.670230	-0.929781	3.677654
C	-0.029873	-3.255519	3.757258
N	-1.981411	-1.268644	3.661095
C	-1.386795	-3.606346	3.760213
C	-2.321946	-2.564142	3.700249
H	0.758274	-4.021945	3.781540
H	-1.709801	-4.656955	3.793839
H	-3.401945	-2.786011	3.671126
N	-0.425934	0.424790	3.547353
H	0.478422	0.794901	3.840464
H	-1.230418	1.049062	3.633390
C	2.481279	-0.975585	4.906305
F	1.896707	0.230076	5.213687
F	2.273667	-1.777001	5.984882
F	3.806349	-0.762022	4.807208
H	-0.299952	1.184631	-3.974841
H	-2.756804	0.073471	-3.124720
H	-0.066736	-2.592095	0.770915
H	-3.728996	-4.592396	-1.805035
H	4.683085	-2.036376	-3.159270
H	2.132131	0.117393	-4.465067
H	-2.116015	4.257670	2.602800
H	-1.690731	4.268922	0.154544
H	1.541846	5.911107	0.034686
H	-6.025191	0.949322	-0.493596
H	-4.890991	3.806804	0.706494
H	-3.316932	-4.011024	0.392289
H	-5.123711	-0.329038	1.817312
H	4.791986	0.267278	-2.041518
H	3.237664	4.275659	-1.461043
4B c			
Ag1RX-OCF3ALKIPh--2NpostAFTER SCF Done: -4015.07443545 A.U.			
Ag	-0.209081	-0.087487	-1.209411
N	0.959683	-0.039805	1.709711
C	1.903601	-2.742681	1.348046
H	0.985072	-2.346386	1.829618
C	2.804831	-1.534572	1.082167
C	2.315876	-0.219138	1.265488
C	-0.112659	0.032779	0.864739
C	3.118738	0.924725	1.050506
C	2.494993	2.312213	1.221830
H	1.470160	2.235896	0.787201
C	4.138490	-1.690503	0.680031
C	4.970843	-0.567793	0.502057

C	4.468184	0.732338	0.714839
C	0.567198	0.039891	3.049731
C	-0.796566	0.166899	3.047128
N	-1.183749	0.169796	1.704896
C	-2.524917	0.313626	1.212516
C	-3.281410	-0.856680	0.953958
C	-3.010673	1.602919	0.924489
C	-4.582536	-0.701386	0.464475
C	-4.328722	1.735200	0.444189
C	-5.111771	0.587051	0.214949
C	-2.624973	-2.220937	1.177666
H	-1.590012	-2.123163	0.785034
C	-2.078613	2.808756	1.022316
H	-1.241633	2.500928	1.681028
C	-1.436032	3.178591	-0.321631
C	-1.903569	2.690094	-1.557502
C	-0.319635	4.046007	-0.321245
C	-1.289235	3.079039	-2.762485
H	-2.745744	1.983681	-1.585045
C	0.310397	4.420607	-1.517725
C	-0.183988	3.945853	-2.748050
H	-1.645509	2.659950	-3.715279
H	0.306533	4.228585	-3.692348
C	-2.745022	3.995468	1.715809
C	-2.794188	4.026035	3.126203
C	-3.341368	5.047111	0.991424
C	-3.430310	5.081096	3.799893
H	-2.324927	3.207082	3.697479
C	-3.983317	6.102579	1.663718
C	-4.030031	6.123152	3.068514
H	-3.456192	5.093159	4.901338
H	-4.443042	6.919408	1.084654
H	-4.526982	6.953636	3.594423
C	-3.262812	-3.329141	0.348351
C	-4.380803	-4.059044	0.801855
C	-2.744199	-3.594414	-0.935716
C	-4.983665	-5.021777	-0.026404
C	-3.347722	-4.552673	-1.765795
H	-1.851688	-3.051701	-1.288927
C	-4.472518	-5.266018	-1.314919
H	-5.856395	-5.587364	0.337585
H	-2.925156	-4.740985	-2.764900
H	-4.946373	-6.020247	-1.963266
C	-2.483912	-2.558288	2.661586
C	-1.419668	-3.386827	3.074684
C	-3.368650	-2.053427	3.635651
C	-1.225982	-3.687134	4.431414
H	-0.738115	-3.796677	2.313512
C	-3.180225	-2.355096	4.997422
C	-2.104688	-3.165918	5.400113
H	-0.378502	-4.323970	4.730651
H	-3.878204	-1.950885	5.748287

H	-1.953112	-3.395804	6.466767
C	3.145250	3.453621	0.423491
C	3.673041	3.230239	-0.871156
C	3.098262	4.781167	0.901248
C	4.110182	4.303913	-1.666012
H	3.767221	2.204560	-1.274151
C	3.536306	5.854726	0.104962
C	4.038489	5.623635	-1.187198
H	4.507517	4.096936	-2.672745
H	3.481807	6.881020	0.501989
H	4.377241	6.464849	-1.812393
C	2.306280	2.635498	2.707723
C	1.114242	3.233420	3.165879
C	3.306023	2.324892	3.652035
C	0.913383	3.505650	4.528444
H	0.321134	3.478720	2.444445
C	3.109723	2.589841	5.018708
C	1.911098	3.177928	5.463268
H	-0.030374	3.970504	4.855433
H	3.898672	2.331522	5.743035
H	1.757234	3.381221	6.534881
C	1.400863	-3.467717	0.093174
C	1.483333	-2.905800	-1.198632
C	0.726054	-4.701827	0.250285
C	0.885004	-3.546804	-2.303729
C	0.148658	-5.354250	-0.849133
H	0.671251	-5.167174	1.247410
C	0.223026	-4.772277	-2.126581
H	0.887592	-3.081855	-3.307496
H	-0.378244	-6.310261	-0.701652
H	-0.246979	-5.264031	-2.992775
C	2.545170	-3.656354	2.392784
C	2.293333	-3.421259	3.760564
C	3.415595	-4.707510	2.035520
C	2.900124	-4.211199	4.751889
H	1.600535	-2.612104	4.045332
C	4.027663	-5.496369	3.024068
C	3.773288	-5.250876	4.385711
H	2.686096	-4.016341	5.815162
H	4.703287	-6.314776	2.728181
H	4.249344	-5.873787	5.159520
O	6.248978	-0.828586	0.105038
C	7.123690	0.269885	-0.126306
H	6.733277	0.947186	-0.919978
H	7.298133	0.862783	0.800607
H	8.084515	-0.164937	-0.460494
O	-6.378767	0.612000	-0.288120
C	-6.965351	1.873647	-0.587994
H	-7.983084	1.660086	-0.965140
H	-7.039064	2.520910	0.315208
H	-6.393744	2.415986	-1.375816
C	-0.599982	-0.576633	-3.323668

C	-1.786256	-0.639099	-2.911847
C	-3.214463	-0.772782	-2.746162
H	-3.536501	-1.305322	-1.837186
C	-4.072635	-0.988710	-3.991476
C	-4.166385	0.316498	-3.238488
H	-3.720700	1.209242	-3.705452
H	-5.053559	0.504947	-2.612776
H	-3.539157	-0.988954	-4.955053
H	-4.895313	-1.716528	-3.906529
O	0.248215	-2.003510	-5.028022
C	0.453488	-0.837382	-4.475102
C	1.920966	-0.634008	-3.938275
C	2.374491	0.298414	-2.963874
C	2.861073	-1.563047	-4.401374
N	3.612254	0.266091	-2.427223
C	4.165570	-1.582937	-3.879495
C	4.479671	-0.660731	-2.871614
H	2.494084	-2.293554	-5.141375
H	4.911226	-2.317162	-4.220335
H	5.467672	-0.676849	-2.379088
N	1.502706	1.253356	-2.408032
H	0.866949	1.690351	-3.083682
H	1.983082	1.964475	-1.845466
C	0.142395	0.317922	-5.512376
F	0.317660	1.607097	-5.002753
F	0.960519	0.228124	-6.576821
F	-1.133724	0.273167	-5.956158
H	-1.523506	0.228639	3.861490
H	1.296577	0.033237	3.864688
H	2.031730	-1.961745	-1.352998
H	3.593664	-4.921339	0.969603
H	-4.774949	-3.872659	1.814029
H	-4.200913	-1.401945	3.324143
H	1.194400	5.076814	-1.483080
H	0.062649	4.448664	0.630282
H	-3.284030	5.042478	-0.108880
H	4.238527	1.847684	3.311514
H	2.705004	4.985328	1.908514
H	4.563332	-2.689078	0.507866
H	5.106996	1.611005	0.565919
H	-5.205425	-1.577100	0.231853
H	-4.708948	2.742907	0.229653
4B c’			
Ag1RX-OCF3ALKIPhISOMER1--2min SCF Done: -4015.08891252 A.U.			
Ag	-0.796271	0.742524	-0.759282
N	-0.188070	0.005767	2.102025
C	-2.349400	1.575766	3.248705
H	-1.402991	1.556856	3.831923
C	-2.578801	0.149762	2.733013
C	-1.499500	-0.590263	2.185323

C	0.339027	0.403608	0.900369
C	-1.682383	-1.889796	1.652442
C	-0.516251	-2.710461	1.092972
H	0.226702	-1.980802	0.701937
C	-3.852795	-0.432636	2.733760
C	-4.057089	-1.724171	2.199778
C	-2.972076	-2.448143	1.672778
C	0.763594	0.080089	3.121460
C	1.922693	0.533589	2.543744
N	1.649503	0.700559	1.184083
C	2.635273	0.828796	0.138250
C	2.751868	2.034463	-0.592794
C	3.390520	-0.318114	-0.194916
C	3.641069	2.071513	-1.676025
C	4.284299	-0.251860	-1.280919
C	4.409105	0.940113	-2.023744
C	1.986038	3.279206	-0.149170
H	1.084927	2.934185	0.400514
C	3.258916	-1.600759	0.622701
H	2.235733	-1.610936	1.053691
C	3.310313	-2.863891	-0.238328
C	2.338035	-3.011685	-1.251298
C	4.203463	-3.923909	0.006785
C	2.231206	-4.204838	-1.979149
H	1.635589	-2.188647	-1.453547
C	4.109016	-5.118237	-0.732745
C	3.117665	-5.267029	-1.718299
H	1.431554	-4.307589	-2.728816
H	3.030059	-6.210658	-2.279513
C	4.206354	-1.625078	1.822608
C	3.746580	-2.157845	3.046354
C	5.531856	-1.151596	1.746555
C	4.592643	-2.227351	4.163810
H	2.709036	-2.514604	3.121968
C	6.380783	-1.216178	2.866330
C	5.915385	-1.755613	4.078449
H	4.209955	-2.650743	5.106152
H	7.412495	-0.837142	2.790720
H	6.580374	-1.803167	4.955458
C	1.434073	4.109929	-1.305912
C	1.415871	5.518683	-1.279455
C	0.782797	3.446494	-2.369510
C	0.759454	6.245637	-2.290167
C	0.119177	4.166133	-3.372768
H	0.770922	2.345215	-2.408325
C	0.106174	5.572703	-3.337125
H	0.752473	7.346768	-2.250115
H	-0.405829	3.615480	-4.164743
H	-0.418075	6.141650	-4.121320
C	2.849602	4.023257	0.870141
C	2.508698	3.988685	2.237639
C	4.026153	4.701551	0.484478

C	3.315003	4.624367	3.198746
H	1.598893	3.451385	2.546346
C	4.830981	5.341619	1.440094
C	4.478340	5.305342	2.802384
H	3.029924	4.588009	4.262478
H	5.741912	5.872325	1.120046
H	5.110805	5.806798	3.552021
C	-0.932998	-3.530637	-0.128462
C	-0.974870	-2.897758	-1.387241
C	-1.287660	-4.892477	-0.044727
C	-1.311206	-3.611683	-2.544896
H	-0.733552	-1.823145	-1.468723
C	-1.652922	-5.607483	-1.200123
C	-1.656970	-4.971854	-2.453054
H	-1.316760	-3.095214	-3.516303
H	-1.929825	-6.670922	-1.117595
H	-1.943847	-5.528679	-3.358695
C	0.226970	-3.513568	2.170049
C	1.226520	-4.437326	1.783491
C	-0.008679	-3.325130	3.548224
C	1.969010	-5.141215	2.743430
H	1.423485	-4.608138	0.715658
C	0.736930	-4.029004	4.513049
C	1.729642	-4.940420	4.115190
H	2.750794	-5.841225	2.409914
H	0.532612	-3.865981	5.583483
H	2.315082	-5.490298	4.868765
C	-2.121679	2.556966	2.098194
C	-2.841737	2.460236	0.889297
C	-1.178319	3.594088	2.241253
C	-2.614392	3.369595	-0.161436
C	-0.944459	4.501277	1.193989
H	-0.621429	3.689311	3.188535
C	-1.655208	4.385412	-0.014313
H	-3.137282	3.227997	-1.118101
H	-0.178166	5.284454	1.308080
H	-1.435162	5.065967	-0.851155
C	-3.440222	2.019741	4.227073
C	-3.441942	1.488668	5.535966
C	-4.469431	2.908483	3.858815
C	-4.447522	1.831423	6.452649
H	-2.645407	0.785757	5.833025
C	-5.480272	3.252835	4.775126
C	-5.474380	2.716010	6.073178
H	-4.430844	1.407437	7.469502
H	-6.277120	3.949179	4.468817
H	-6.265292	2.987295	6.790300
O	-5.338405	-2.186483	2.223662
C	-5.578510	-3.535971	1.829231
H	-5.308424	-3.712171	0.764208
H	-5.013254	-4.252352	2.467643
H	-6.663466	-3.706317	1.960801

O	5.227546	1.084168	-3.105548
C	6.035728	-0.020931	-3.497132
H	6.625851	0.321455	-4.367744
H	6.730996	-0.331053	-2.684346
H	5.418377	-0.896528	-3.800968
C	-1.071952	-0.041346	-3.813673
C	0.136127	-0.269618	-3.867052
C	1.563570	-0.403156	-3.747364
H	1.933639	-0.057372	-2.762679
C	2.503655	-0.141305	-4.923000
C	2.361355	-1.540358	-4.378202
H	1.799998	-2.281277	-4.969072
H	3.171889	-1.964321	-3.763460
H	2.034124	0.088600	-5.892438
H	3.416260	0.434419	-4.702252
O	-2.155081	1.281801	-2.225352
C	-2.387207	0.456859	-3.307703
C	-3.347700	-0.719482	-2.959863
C	-3.599847	-1.867895	-3.776091
C	-4.000487	-0.663896	-1.722971
N	-4.402129	-2.880423	-3.372758
C	-4.841965	-1.707860	-1.303375
C	-4.994206	-2.798004	-2.172964
H	-3.818376	0.219898	-1.094243
H	-5.361517	-1.661098	-0.335392
H	-5.635444	-3.655819	-1.893093
N	-2.977002	-2.074367	-4.993329
H	-2.672873	-1.260428	-5.526584
H	-3.384694	-2.841239	-5.532561
C	-3.067581	1.378530	-4.377831
F	-2.271125	2.410617	-4.739945
F	-3.377131	0.709723	-5.537350
F	-4.219401	1.893693	-3.901797
H	2.918859	0.710462	2.957721
H	0.543235	-0.227517	4.147432
H	-3.575117	1.648959	0.758888
H	-4.476560	3.338510	2.845583
H	1.903132	6.056162	-0.451722
H	4.308279	4.731294	-0.579483
H	4.808294	-5.943807	-0.524110
H	4.956997	-3.826729	0.803193
H	5.902758	-0.717595	0.805066
H	-0.795210	-2.626356	3.872883
H	-1.259254	-5.403313	0.930941
H	-4.715581	0.109703	3.145675
H	-3.114653	-3.442941	1.232404
H	3.732847	2.977877	-2.291526
H	4.838741	-1.160437	-1.551803
4D cat			
Ag2 SCF Done: -3606.98753192 A.U.			
Ag	-0.094669	0.052798	-2.057145

Cl	-0.053573	-0.149477	-4.377522
N	0.663208	0.798944	0.845813
C	3.495235	0.215245	0.489778
H	2.632391	-0.453208	0.693037
C	2.899147	1.583037	0.167634
C	1.526136	1.840811	0.355752
C	-0.083668	0.030659	-0.005163
C	0.948243	3.083842	-0.017050
C	-0.556785	3.303851	0.160813
H	-1.058890	2.352337	-0.120798
C	3.718903	2.602350	-0.356092
H	4.781662	2.381894	-0.521069
C	3.170348	3.855536	-0.687832
C	1.784934	4.084238	-0.521588
H	1.373473	5.054688	-0.834170
C	0.441298	0.458036	2.191203
C	-0.470439	-0.577579	2.176856
N	-0.777233	-0.808785	0.823354
C	-1.599743	-1.879660	0.328669
C	-0.964929	-3.065419	-0.122677
C	-2.998727	-1.719064	0.258346
C	-1.758604	-4.100218	-0.629481
C	-3.779537	-2.785616	-0.227290
C	-3.165211	-3.974711	-0.671123
H	-1.296557	-5.020339	-1.016132
H	-4.868499	-2.656397	-0.275176
C	0.552140	-3.206004	-0.009790
H	0.973010	-2.188103	-0.145206
C	-3.632956	-0.366148	0.580066
H	-2.978909	0.119306	1.336657
C	-3.642672	0.563911	-0.641288
C	-3.986340	1.923602	-0.458835
C	-3.316982	0.119572	-1.937471
C	-4.004672	2.811798	-1.543831
H	-4.255472	2.285553	0.546609
C	-3.325960	1.011158	-3.028322
H	-3.040129	-0.931605	-2.107748
C	-3.673743	2.355621	-2.833666
H	-4.246104	3.873778	-1.381540
H	-3.010929	0.647799	-4.018157
H	-3.661822	3.060765	-3.678243
C	-5.007307	-0.509639	1.231469
C	-5.103863	-0.714363	2.624164
C	-6.192635	-0.486181	0.466962
C	-6.352431	-0.900402	3.240119
H	-4.183475	-0.725563	3.231862
C	-7.443442	-0.677854	1.079429
H	-6.127826	-0.301456	-0.617181
C	-7.527806	-0.886863	2.467332
H	-6.409246	-1.052506	4.329891
H	-8.359886	-0.655346	0.468169
H	-8.508475	-1.030139	2.948170

C	1.168525	-4.026961	-1.142387
C	0.960931	-3.586929	-2.470269
C	1.978651	-5.155528	-0.918304
C	1.553103	-4.259873	-3.548085
H	0.338846	-2.696930	-2.667925
C	2.572644	-5.832783	-2.001010
H	2.156544	-5.502776	0.111423
C	2.363486	-5.388250	-3.316598
H	1.382609	-3.889269	-4.571264
H	3.207548	-6.712762	-1.809551
H	2.832636	-5.917205	-4.161494
C	0.941135	-3.629446	1.405223
C	1.966945	-2.937404	2.078978
C	0.281147	-4.675867	2.083253
C	2.326290	-3.263883	3.396189
H	2.479978	-2.112671	1.564677
C	0.639644	-5.014545	3.399857
H	-0.533226	-5.219885	1.579471
C	1.658962	-4.306925	4.063780
H	3.126854	-2.693725	3.894183
H	0.110778	-5.832291	3.915102
H	1.930030	-4.566752	5.099391
C	-1.130086	4.341418	-0.803118
C	-1.880368	5.452887	-0.377258
C	-0.956044	4.128577	-2.190034
C	-2.446090	6.337540	-1.315935
H	-2.039493	5.623268	0.698295
C	-1.510211	5.013325	-3.125140
H	-0.396762	3.244104	-2.538653
C	-2.261589	6.122504	-2.691500
H	-3.036014	7.199408	-0.964888
H	-1.368006	4.824056	-4.200850
H	-2.703337	6.814730	-3.425888
C	-0.865156	3.535788	1.637835
C	-1.787114	2.708529	2.307961
C	-0.202762	4.541381	2.375110
C	-2.050120	2.876388	3.677780
H	-2.283896	1.906255	1.743759
C	-0.465648	4.718458	3.744081
H	0.535507	5.182724	1.867368
C	-1.389272	3.884356	4.401487
H	-2.771742	2.211976	4.179600
H	0.060137	5.508141	4.304227
H	-1.589812	4.018747	5.476268
C	4.196319	-0.373937	-0.737384
C	3.426577	-1.039048	-1.713480
C	5.583359	-0.228548	-0.949080
C	4.020075	-1.545146	-2.879550
H	2.338990	-1.151125	-1.571206
C	6.182022	-0.732000	-2.117617
H	6.201029	0.267569	-0.182800
C	5.402411	-1.390483	-3.085759

H	3.390889	-2.062569	-3.619351
H	7.267202	-0.614087	-2.268970
H	5.873423	-1.787456	-3.999003
C	4.344668	0.187534	1.764274
C	4.935951	-1.034557	2.161399
C	4.511084	1.312231	2.597021
C	5.649375	-1.133893	3.365784
H	4.838544	-1.916605	1.508802
C	5.230528	1.216738	3.803285
H	4.061473	2.273255	2.305380
C	5.796972	-0.006552	4.196217
H	6.096471	-2.098173	3.655974
H	5.344287	2.108595	4.440127
H	6.357949	-0.082100	5.141155
O	3.884640	4.897742	-1.193044
C	5.272021	4.710102	-1.443355
H	5.834499	4.480270	-0.509016
H	5.452843	3.895009	-2.180530
H	5.643849	5.664594	-1.861489
O	-3.841580	-5.047159	-1.164817
C	-5.258238	-4.970262	-1.264849
H	-5.737648	-4.822385	-0.270074
H	-5.581278	-4.148063	-1.943551
H	-5.592078	-5.937107	-1.686571
C	1.109565	1.144558	3.331340
H	1.220419	2.227880	3.122678
H	0.501715	1.036149	4.250260
H	2.120467	0.729188	3.527708
C	-1.051137	-1.378064	3.293651
H	-2.024628	-1.817640	2.999040
H	-0.382413	-2.219460	3.577272
H	-1.204561	-0.742404	4.189004
4D a			
Ag2RX-ALKI SCF Done: -3340.14379179 A.U.			
Ag	0.109127	-0.268554	1.764330
N	-0.511879	-0.979261	-1.141474
C	2.132271	-1.954915	-1.505996
H	1.809582	-0.904470	-1.355567
C	1.053711	-2.836720	-0.878858
C	-0.268732	-2.340181	-0.746288
C	-0.268529	0.032965	-0.252761
C	-1.272129	-3.068265	-0.079912
C	-2.651747	-2.441042	0.121633
H	-2.494601	-1.346172	0.234471
C	1.337398	-4.117007	-0.390540
C	0.330486	-4.886969	0.234924
C	-0.967264	-4.357474	0.395729
C	-0.700891	-0.504509	-2.446708
C	-0.550716	0.866802	-2.373422
N	-0.286382	1.164654	-1.017807

C	-0.071008	2.475135	-0.462650
C	1.231749	2.866505	-0.040192
C	-1.186345	3.330595	-0.296429
C	1.393019	4.153871	0.489155
C	-0.988067	4.617375	0.236828
C	0.301187	5.036900	0.618419
C	2.442696	1.929081	-0.141655
H	2.060558	0.896911	0.020284
C	-2.604915	2.829531	-0.589639
H	-2.544529	2.173286	-1.484936
C	-3.156159	1.939172	0.533133
C	-2.501759	1.756302	1.766162
C	-4.368276	1.248871	0.305924
C	-3.021951	0.871777	2.732302
H	-1.561798	2.289504	1.975487
C	-4.890206	0.369033	1.266269
C	-4.211541	0.169875	2.482452
H	-2.464142	0.709508	3.667675
H	-4.598268	-0.550925	3.218811
C	-3.549794	3.972366	-0.961340
C	-3.553437	4.473688	-2.280258
C	-4.385301	4.583609	-0.003341
C	-4.366213	5.562509	-2.635508
H	-2.905057	4.001454	-3.036910
C	-5.198017	5.675984	-0.354539
C	-5.190628	6.170050	-1.670777
H	-4.359421	5.937085	-3.671648
H	-5.845495	6.141246	0.405941
H	-5.831162	7.022659	-1.946899
C	3.447842	2.172005	0.990073
C	4.584395	2.988900	0.813183
C	3.220884	1.594265	2.256123
C	5.475732	3.217391	1.876826
C	4.111399	1.816797	3.317904
H	2.336925	0.955528	2.424883
C	5.244680	2.629155	3.132834
H	6.359295	3.856845	1.719787
H	3.911708	1.345025	4.292946
H	5.946305	2.802804	3.964404
C	3.101271	1.915956	-1.522884
C	4.219688	1.074513	-1.730020
C	2.640433	2.697048	-2.600310
C	4.846754	1.006802	-2.981465
H	4.601215	0.470858	-0.893571
C	3.268160	2.629028	-3.860033
C	4.372280	1.783101	-4.055904
H	5.702388	0.327484	-3.118266
H	2.891888	3.249459	-4.689498
H	4.860313	1.724802	-5.041460
C	-3.320988	-2.866255	1.429924
C	-2.595660	-2.718767	2.635490
C	-4.657717	-3.301908	1.494054

C	-3.192867	-3.003327	3.871377
H	-1.555606	-2.349795	2.612547
C	-5.260843	-3.583838	2.735433
C	-4.531900	-3.436899	3.927513
H	-2.608589	-2.866188	4.795034
H	-6.310374	-3.918275	2.766739
H	-5.005323	-3.655706	4.898019
C	-3.491223	-2.627436	-1.140137
C	-4.040835	-1.506309	-1.792551
C	-3.705091	-3.905735	-1.699371
C	-4.798249	-1.653110	-2.967803
H	-3.854410	-0.507809	-1.371384
C	-4.460468	-4.057882	-2.873528
C	-5.011788	-2.931281	-3.512520
H	-5.216059	-0.762404	-3.464109
H	-4.615782	-5.062933	-3.297405
H	-5.600980	-3.050837	-4.435596
C	3.469298	-2.020931	-0.766599
C	3.491001	-1.619741	0.589040
C	4.683853	-2.335578	-1.403558
C	4.703956	-1.491447	1.279678
C	5.902128	-2.223596	-0.705916
H	4.681034	-2.636459	-2.462512
C	5.917996	-1.788572	0.630264
H	4.695911	-1.135190	2.321214
H	6.847206	-2.463260	-1.219450
H	6.873840	-1.678512	1.166435
C	2.220353	-2.127270	-3.020147
C	2.269446	-0.979201	-3.837592
C	2.237574	-3.397166	-3.633171
C	2.337095	-1.092618	-5.234789
H	2.236992	0.014652	-3.367520
C	2.301204	-3.514444	-5.033016
C	2.349976	-2.362578	-5.839131
H	2.377856	-0.179360	-5.849684
H	2.307996	-4.513547	-5.497411
H	2.395285	-2.456029	-6.935929
O	0.716225	-6.114297	0.680687
C	-0.222694	-6.900553	1.403088
H	-0.571915	-6.384206	2.326105
H	-1.109829	-7.163815	0.781878
H	0.304985	-7.830752	1.687081
O	0.585655	6.262697	1.137000
C	-0.481269	7.183127	1.328412
H	-0.028882	8.097573	1.756557
H	-0.985088	7.441718	0.368868
H	-1.245272	6.790630	2.037971
C	0.636377	-0.608569	3.681420
C	1.111651	-0.761525	4.819628
C	1.709451	-0.911434	6.126474
H	1.164560	-1.569705	6.829296
C	2.447923	0.262792	6.774049

C	3.233520	-0.924636	6.272340
H	3.816213	-0.815745	5.343113
H	3.672942	-1.615580	7.010689
H	2.482698	1.200561	6.196002
H	2.339914	0.399205	7.862827
H	2.543111	-1.378152	1.102721
H	2.183697	-4.302131	-3.008088
H	4.779666	3.442078	-0.171682
H	1.787337	3.375100	-2.449522
H	-5.818085	-0.185590	1.056060
H	-4.910066	1.412597	-0.640580
H	-4.398644	4.190167	1.025394
H	-3.264173	-4.788952	-1.210760
H	-5.240606	-3.406718	0.565996
H	2.360249	-4.519605	-0.434024
H	-1.744083	-4.907475	0.943285
H	2.379487	4.493696	0.834556
H	-1.858815	5.273646	0.359844
C	-0.585652	1.889707	-3.462661
H	-0.642900	2.913086	-3.042759
H	0.332906	1.837605	-4.086138
H	-1.460960	1.736702	-4.125934
C	-1.012022	-1.396247	-3.600847
H	-0.811195	-0.873829	-4.555174
H	-0.379668	-2.305891	-3.577371
H	-2.074161	-1.720270	-3.580950

4D a+b

Ag2RX-OCF3ALKIPh--2Npre SCF Done: -4093.67239781 A.U.

Ag	0.552441	-0.395325	1.110291
N	-1.587681	0.639475	-1.020692
C	-1.025562	-1.476133	-2.822596
H	-0.307990	-0.712264	-2.459268
C	-2.090635	-1.636889	-1.739334
C	-2.414220	-0.531934	-0.909469
C	-0.364442	0.677053	-0.407684
C	-3.445204	-0.594486	0.044913
C	-3.797980	0.643652	0.867061
H	-2.846236	1.191044	1.048411
C	-2.815069	-2.824226	-1.582363
C	-3.859443	-2.905219	-0.634925
C	-4.173573	-1.793576	0.171864
C	-1.727704	1.660681	-1.976082
C	-0.536023	2.356931	-1.964409
N	0.280297	1.729535	-1.003822
C	1.604778	2.148312	-0.612852
C	2.750184	1.405884	-1.023045
C	1.724721	3.271995	0.238161
C	4.005658	1.850935	-0.590389
C	3.002016	3.687951	0.657368
C	4.144289	2.976418	0.246050

C	2.644552	0.129357	-1.869021
H	1.717454	-0.390455	-1.541205
C	0.475372	3.953081	0.803956
H	-0.303965	3.931353	0.013247
C	-0.106012	3.173513	1.988202
C	0.668911	2.315721	2.791646
C	-1.475689	3.328042	2.292026
C	0.080756	1.616337	3.864243
H	1.734318	2.154805	2.568165
C	-2.069345	2.621974	3.348598
C	-1.288034	1.755993	4.138281
H	0.708228	0.929387	4.451988
H	-1.761732	1.182445	4.949737
C	0.718170	5.429003	1.123553
C	0.711768	6.373379	0.074330
C	1.001548	5.873488	2.431232
C	0.988896	7.727258	0.321328
H	0.488809	6.035519	-0.951368
C	1.283068	7.228790	2.681669
C	1.279159	8.159658	1.628763
H	0.975633	8.450434	-0.509818
H	1.502463	7.559195	3.709616
H	1.495458	9.221623	1.825991
C	3.786593	-0.848777	-1.580924
C	5.009510	-0.786219	-2.284322
C	3.633610	-1.820269	-0.575624
C	6.057627	-1.668751	-1.971587
C	4.670129	-2.717642	-0.272338
H	2.693688	-1.883745	-0.010918
C	5.890339	-2.638222	-0.964630
H	7.008209	-1.604765	-2.525296
H	4.492805	-3.473541	0.506450
H	6.709935	-3.335083	-0.726685
C	2.486035	0.367729	-3.370700
C	2.337152	-0.761398	-4.210617
C	2.498680	1.647668	-3.955618
C	2.185360	-0.609270	-5.595113
H	2.340720	-1.768504	-3.765920
C	2.342188	1.802727	-5.347749
C	2.183865	0.675949	-6.171393
H	2.051272	-1.502644	-6.224505
H	2.354014	2.812957	-5.788054
H	2.057856	0.796111	-7.258874
C	-4.347320	0.296670	2.251779
C	-3.559390	-0.510731	3.100981
C	-5.595876	0.752596	2.718408
C	-4.011301	-0.868381	4.379093
H	-2.582507	-0.877177	2.747152
C	-6.044044	0.412207	4.009547
C	-5.256018	-0.400131	4.841781
H	-3.385443	-1.540297	4.987246
H	-7.021851	0.780112	4.360062

H	-5.613342	-0.675856	5.846912
C	-4.703742	1.585331	0.071414
C	-4.577198	2.980158	0.238124
C	-5.683699	1.103122	-0.820856
C	-5.392196	3.872832	-0.477309
H	-3.820436	3.364589	0.936575
C	-6.504026	1.991509	-1.537202
C	-6.358370	3.380361	-1.373473
H	-5.268571	4.958670	-0.337598
H	-7.257220	1.594750	-2.236495
H	-6.994796	4.076751	-1.942043
C	-0.162678	-2.718832	-3.038466
C	0.612241	-3.188057	-1.953670
C	-0.019297	-3.336798	-4.295463
C	1.534132	-4.230850	-2.128190
C	0.897462	-4.390650	-4.470515
H	-0.605591	-2.970223	-5.151626
C	1.682475	-4.832765	-3.392278
H	2.140303	-4.548755	-1.268316
H	1.005599	-4.858707	-5.462317
H	2.413371	-5.644593	-3.534522
C	-1.627346	-0.890902	-4.101798
C	-0.958698	0.160348	-4.761857
C	-2.835337	-1.371429	-4.648946
C	-1.476247	0.715419	-5.942995
H	-0.027938	0.557343	-4.332506
C	-3.360370	-0.813642	-5.827934
C	-2.682766	0.232346	-6.480001
H	-0.927301	1.530798	-6.440293
H	-4.307509	-1.198242	-6.239061
H	-3.095830	0.669797	-7.402908
O	-4.504089	-4.106279	-0.563082
C	-5.595184	-4.233734	0.341019
H	-5.279575	-4.074505	1.396797
H	-6.413946	-3.517287	0.101814
H	-5.972847	-5.267389	0.228207
O	5.417981	3.282444	0.619582
C	5.617711	4.370279	1.513168
H	6.708107	4.429938	1.690862
H	5.267495	5.334830	1.079438
H	5.099611	4.205798	2.485530
C	2.017209	-0.917943	2.437597
C	3.168615	-0.650102	2.837028
C	4.512756	-0.300366	3.218089
H	4.601126	0.322241	4.128181
C	5.534731	-0.002589	2.110275
C	5.693393	-1.238370	2.950062
H	5.456613	-2.207441	2.487857
H	6.486987	-1.267106	3.714614
H	5.177244	-0.137565	1.077276
H	6.209834	0.855517	2.257848
O	1.978726	-3.909122	0.972440

C	1.543013	-3.763508	2.112864
C	0.088444	-3.677614	2.400158
C	-0.568323	-3.011175	3.501788
C	-0.747743	-4.343928	1.473654
N	-1.887318	-3.212181	3.760100
C	-2.119059	-4.474937	1.707564
C	-2.619796	-3.927668	2.905648
H	-0.276456	-4.786935	0.582475
H	-2.772527	-5.005993	1.002151
H	-3.682385	-4.061242	3.184531
N	0.062319	-2.108938	4.295679
H	0.907511	-1.641617	3.906193
H	-0.553259	-1.563722	4.902327
C	2.550958	-4.109035	3.263221
F	2.413407	-3.439440	4.421946
F	2.366437	-5.433521	3.540009
F	3.825918	-3.958215	2.843673
H	0.511938	-2.722693	-0.958250
H	-3.377522	-2.183865	-4.140453
H	5.135502	-0.044899	-3.090071
H	2.643389	2.531094	-3.315564
H	-3.148111	2.723163	3.546423
H	-2.076648	4.021939	1.681647
H	0.996605	5.147064	3.259072
H	-5.791934	0.017790	-0.970080
H	-6.224892	1.380751	2.069020
H	-2.569051	-3.712857	-2.182420
H	-4.960801	-1.843072	0.935198
H	4.916528	1.305528	-0.870922
H	3.076432	4.558626	1.321678
C	-0.113045	3.521462	-2.798488
H	0.801373	3.991822	-2.386878
H	0.113099	3.203428	-3.839090
H	-0.912915	4.288281	-2.840428
C	-2.953378	1.872338	-2.799375
H	-2.678351	2.217242	-3.814856
H	-3.517428	0.926316	-2.910124
H	-3.633601	2.617434	-2.336120
4D a+b→c			
Ag2RX-OCF3ALKIPh--2N SCF Done: -4093.66320435 A.U.			
Ag	-0.605773	0.095919	-1.080328
N	1.604662	-0.424054	1.134956
C	0.453055	-2.915515	1.810299
H	0.031188	-1.896002	1.916871
C	1.548346	-2.828971	0.747340
C	2.139246	-1.572917	0.465053
C	0.515801	0.242452	0.648889
C	3.179500	-1.413924	-0.472100
C	3.686764	-0.000092	-0.770906
H	2.783330	0.651685	-0.767117

C	2.035490	-3.953001	0.068414
C	3.129946	-3.831945	-0.816551
C	3.701069	-2.569136	-1.081882
C	1.954236	0.001560	2.426857
C	1.046047	0.986703	2.757095
N	0.175102	1.099651	1.656730
C	-0.900371	2.044671	1.529344
C	-2.243258	1.642569	1.780731
C	-0.582795	3.344637	1.076605
C	-3.232038	2.631398	1.704746
C	-1.601758	4.313367	1.008103
C	-2.923096	3.960062	1.341297
C	-2.602016	0.162344	1.995204
H	-1.879779	-0.411456	1.372948
C	0.810079	3.638471	0.512348
H	1.508476	2.922084	0.992392
C	0.890204	3.356635	-0.998437
C	-0.250532	3.204060	-1.812461
C	2.163024	3.232107	-1.601939
C	-0.122012	2.944103	-3.190794
H	-1.255689	3.264407	-1.369880
C	2.295806	2.946207	-2.969353
C	1.147794	2.808498	-3.772043
H	-1.031609	2.803537	-3.795263
H	1.248452	2.583896	-4.845969
C	1.312328	5.030393	0.889394
C	1.908941	5.229047	2.153179
C	1.167070	6.135767	0.026400
C	2.342890	6.503747	2.551346
H	2.030507	4.367505	2.830627
C	1.596613	7.414574	0.424393
C	2.184927	7.602972	1.687169
H	2.810624	6.640583	3.539515
H	1.478431	8.268445	-0.261817
H	2.527551	8.603405	1.995474
C	-3.985112	-0.197943	1.445632
C	-5.160792	0.074989	2.180649
C	-4.104688	-0.812172	0.184632
C	-6.424022	-0.217212	1.639548
C	-5.365554	-1.112501	-0.359775
H	-3.209036	-1.054487	-0.402721
C	-6.529515	-0.805010	0.363766
H	-7.332319	0.008472	2.221283
H	-5.396313	-1.575483	-1.358100
H	-7.521743	-1.030322	-0.059155
C	-2.441172	-0.367282	3.418411
C	-2.637021	-1.752477	3.629952
C	-2.142171	0.452558	4.521222
C	-2.528610	-2.301513	4.914231
H	-2.874166	-2.401818	2.771848
C	-2.025552	-0.099788	5.813068
C	-2.220642	-1.476382	6.013670

H	-2.664813	-3.385366	5.051831
H	-1.791722	0.556051	6.667444
H	-2.128916	-1.908549	7.022528
C	4.285170	0.217527	-2.164656
C	3.682660	-0.377068	-3.297294
C	5.348307	1.122546	-2.367245
C	4.118391	-0.053849	-4.592879
H	2.869106	-1.118105	-3.185524
C	5.783318	1.446159	-3.666259
C	5.165651	0.866020	-4.787086
H	3.628728	-0.533800	-5.456222
H	6.611224	2.161022	-3.798955
H	5.503941	1.121548	-5.803889
C	4.589129	0.502588	0.354682
C	4.321579	1.726776	0.999087
C	5.721337	-0.235843	0.757097
C	5.166469	2.216208	2.009762
H	3.423603	2.293595	0.715071
C	6.569554	0.245265	1.768237
C	6.297635	1.475442	2.396642
H	4.932494	3.176384	2.496612
H	7.448278	-0.345540	2.072164
H	6.962804	1.851227	3.190037
C	-0.766029	-3.747464	1.407048
C	-1.470343	-3.373831	0.240481
C	-1.306668	-4.752532	2.232356
C	-2.709228	-3.951709	-0.073886
C	-2.539030	-5.351895	1.909043
H	-0.777776	-5.044664	3.152555
C	-3.249264	-4.943227	0.767152
H	-3.246383	-3.591740	-0.966438
H	-2.953544	-6.131386	2.568740
H	-4.227285	-5.392248	0.531911
C	1.032317	-3.246551	3.186441
C	0.680909	-2.443693	4.291281
C	1.908031	-4.332477	3.396188
C	1.180300	-2.720473	5.574250
H	0.016480	-1.579872	4.139147
C	2.414245	-4.610373	4.677724
C	2.051485	-3.805531	5.773168
H	0.881454	-2.079600	6.418862
H	3.098320	-5.462148	4.820754
H	2.449126	-4.023509	6.777199
O	3.553016	-4.992198	-1.397590
C	4.650352	-4.937690	-2.301570
H	4.429225	-4.296339	-3.185539
H	5.574789	-4.560507	-1.807129
H	4.821562	-5.975962	-2.643191
O	-3.983803	4.815989	1.291212
C	-3.751472	6.158579	0.883671
H	-4.731563	6.670099	0.926105
H	-3.039075	6.680909	1.562202

H	-3.359095	6.213005	-0.157785
C	-2.235491	0.191151	-2.403744
C	-3.020622	1.171780	-2.392858
C	-3.958978	2.250132	-2.370533
H	-3.624717	3.190957	-2.847559
C	-4.932429	2.405089	-1.189589
C	-5.460915	1.935719	-2.507767
H	-5.715876	0.869710	-2.610767
H	-6.046638	2.624645	-3.137454
H	-4.841339	1.659500	-0.386477
H	-5.130137	3.433515	-0.847357
O	-3.557244	-2.120466	-2.735858
C	-2.614499	-1.558598	-3.351307
C	-1.260360	-2.280110	-3.404571
C	0.048952	-1.721510	-3.596639
C	-1.323292	-3.669843	-3.202356
N	1.172190	-2.479875	-3.545947
C	-0.163471	-4.456907	-3.173497
C	1.062168	-3.799499	-3.343595
H	-2.317871	-4.114765	-3.054472
H	-0.207651	-5.543564	-3.010924
H	2.007198	-4.366931	-3.305532
N	0.273286	-0.372857	-3.748647
H	-0.495571	0.254808	-3.978903
H	1.228339	-0.055753	-3.918552
C	-3.043830	-0.929787	-4.722903
F	-2.129677	-0.075869	-5.285789
F	-3.215891	-1.940469	-5.615650
F	-4.211749	-0.262533	-4.634969
H	-1.055053	-2.596539	-0.425270
H	2.204790	-4.964442	2.545415
H	-5.080928	0.508040	3.191471
H	-2.010809	1.534885	4.367086
H	3.298767	2.820661	-3.406432
H	3.070907	3.366937	-0.993711
H	0.725886	5.983374	-0.971689
H	5.933046	-1.201859	0.271495
H	5.835056	1.595443	-1.500856
H	1.567630	-4.939264	0.203061
H	4.512399	-2.458651	-1.812324
H	-4.287256	2.383116	1.877997
H	-1.344366	5.323082	0.661753
C	0.948193	1.810199	3.998150
H	0.314422	2.702966	3.829761
H	0.498893	1.229544	4.831910
H	1.954080	2.148380	4.320487
C	3.093429	-0.560670	3.209673
H	3.356258	-1.572442	2.845525
H	3.997450	0.076973	3.128336
H	2.818322	-0.653266	4.278019

4D c

Ag2RX-OCF3ALKIPh--2Npost SCF Done: -4093.67320079 A.U.

Ag	0.317585	-0.495132	-1.207676
N	0.354697	0.813580	1.628591
C	1.309838	-1.717322	2.539558
H	0.505906	-1.636452	1.780732
C	2.284019	-0.574360	2.246645
C	1.778889	0.656336	1.754567
C	-0.350816	0.421669	0.524159
C	2.629230	1.727607	1.407634
C	2.038589	2.967912	0.732784
H	1.208931	2.592437	0.091098
C	3.660401	-0.687693	2.470219
C	4.514551	0.412092	2.235795
C	4.001491	1.608631	1.696990
C	-0.483879	1.323175	2.636027
C	-1.765456	1.255422	2.133588
N	-1.651530	0.694956	0.849736
C	-2.735766	0.503556	-0.070511
C	-3.529602	-0.667558	0.010387
C	-2.955087	1.483106	-1.063883
C	-4.564472	-0.828924	-0.919187
C	-4.010440	1.302790	-1.976820
C	-4.807556	0.142324	-1.912881
C	-3.225842	-1.749102	1.047179
H	-2.296233	-1.425777	1.560477
C	-2.025315	2.689255	-1.175219
H	-1.462623	2.728305	-0.220771
C	-0.959873	2.557724	-2.268955
C	-0.984307	1.552656	-3.256245
C	0.116565	3.473483	-2.269627
C	0.031305	1.474756	-4.228980
H	-1.799172	0.814347	-3.262720
C	1.138659	3.391067	-3.226635
C	1.093346	2.393054	-4.219410
H	0.003400	0.666223	-4.976691
H	1.895432	2.323784	-4.971250
C	-2.811762	3.997847	-1.249436
C	-3.339003	4.546505	-0.060061
C	-3.053594	4.661564	-2.468685
C	-4.094550	5.729423	-0.086829
H	-3.146623	4.035273	0.897910
C	-3.813565	5.844858	-2.498962
C	-4.336075	6.382381	-1.309898
H	-4.493839	6.146972	0.851359
H	-3.991537	6.354770	-3.459287
H	-4.925576	7.312481	-1.334148
C	-2.879037	-3.067100	0.347338
C	-3.858700	-4.033934	0.036517
C	-1.555439	-3.280468	-0.086738
C	-3.511685	-5.173948	-0.710721
C	-1.199251	-4.405603	-0.850982

H	-0.790661	-2.521341	0.141127
C	-2.189829	-5.355558	-1.161164
H	-4.284116	-5.922685	-0.949826
H	-0.164328	-4.498944	-1.238173
H	-1.929009	-6.240774	-1.762955
C	-4.274336	-1.884332	2.153492
C	-4.094898	-2.886921	3.134748
C	-5.373427	-1.011445	2.282704
C	-4.977131	-3.001141	4.219845
H	-3.252834	-3.590713	3.037237
C	-6.262034	-1.126038	3.368560
C	-6.065798	-2.116973	4.343715
H	-4.814631	-3.788665	4.972972
H	-7.114564	-0.432688	3.448670
H	-6.760487	-2.206715	5.193786
C	2.971989	3.689079	-0.251569
C	3.872218	2.958877	-1.062849
C	2.857882	5.079883	-0.462033
C	4.626832	3.605577	-2.056156
H	4.009027	1.869619	-0.923344
C	3.610123	5.724689	-1.461161
C	4.498932	4.990774	-2.265453
H	5.319025	3.009722	-2.673152
H	3.498678	6.810944	-1.608125
H	5.089217	5.495341	-3.046822
C	1.407292	3.910950	1.757849
C	0.096821	4.396074	1.574793
C	2.124679	4.329977	2.897330
C	-0.492012	5.269352	2.504564
H	-0.475689	4.083299	0.689410
C	1.544189	5.205260	3.830055
C	0.231256	5.675645	3.640062
H	-1.517709	5.632785	2.332745
H	2.118191	5.518124	4.716744
H	-0.224903	6.358164	4.374453
C	1.910628	-3.108797	2.338668
C	1.884861	-3.673381	1.046952
C	2.484244	-3.851348	3.392143
C	2.393868	-4.958985	0.809499
C	3.015404	-5.131860	3.154261
H	2.498701	-3.435257	4.411724
C	2.964141	-5.691949	1.864753
H	2.327611	-5.347626	-0.218968
H	3.458419	-5.700625	3.987906
H	3.364320	-6.702929	1.685086
C	0.626976	-1.528664	3.894118
C	-0.756559	-1.774047	4.011663
C	1.334451	-1.108868	5.039403
C	-1.426406	-1.593856	5.231740
H	-1.316030	-2.106813	3.124004
C	0.674867	-0.947139	6.270934
C	-0.708532	-1.180847	6.370218

H	-2.511080	-1.779527	5.285849
H	1.243939	-0.620142	7.155893
H	-1.227301	-1.039457	7.331661
O	5.834278	0.209262	2.514349
C	6.753649	1.257740	2.234768
H	6.759282	1.524614	1.153002
H	6.531782	2.173946	2.828939
H	7.753138	0.877686	2.518700
O	-5.813786	-0.146292	-2.787576
C	-6.092849	0.780944	-3.829898
H	-6.931807	0.352359	-4.409798
H	-6.397499	1.774774	-3.429985
H	-5.217633	0.918555	-4.505580
C	0.625197	-2.078948	-2.690372
C	-0.495453	-1.951010	-3.241942
C	-1.742967	-1.927875	-3.943892
H	-1.865816	-1.100069	-4.666923
C	-3.035586	-2.464360	-3.317891
C	-2.349385	-3.280870	-4.367344
H	-1.791761	-4.173702	-4.043976
H	-2.774866	-3.329484	-5.382624
H	-2.974016	-2.800407	-2.273988
H	-3.959984	-1.924541	-3.576074
O	1.679714	-4.170474	-2.207025
C	1.936559	-2.976042	-2.683470
C	3.134482	-2.244631	-1.982532
C	3.345916	-0.842888	-1.861511
C	4.077399	-3.074629	-1.362722
N	4.338297	-0.305370	-1.123810
C	5.141579	-2.527938	-0.628384
C	5.209462	-1.132802	-0.521687
H	3.918223	-4.158827	-1.452495
H	5.881306	-3.167666	-0.124422
H	5.996213	-0.654363	0.085892
N	2.447996	0.083453	-2.430341
H	2.137112	-0.147283	-3.379598
H	2.756579	1.058725	-2.354195
C	2.297857	-3.033644	-4.226422
F	2.489653	-1.775617	-4.782738
F	3.446866	-3.715179	-4.422045
F	1.333633	-3.631145	-4.957011
H	1.470530	-3.120495	0.188138
H	2.410662	-0.887571	4.958599
H	-4.894174	-3.896109	0.387991
H	-5.538103	-0.227527	1.527973
H	1.975999	4.104836	-3.185761
H	0.156361	4.271669	-1.511047
H	-2.624360	4.254361	-3.398063
H	3.146886	3.952630	3.058369
H	2.165559	5.669623	0.157935
H	4.095398	-1.637873	2.812226
H	4.668150	2.439302	1.433402

H	-5.185393	-1.736126	-0.910768
H	-4.181875	2.076412	-2.737404
C	-3.065407	1.631176	2.758469
H	-3.776087	2.018623	2.000065
H	-3.553191	0.759164	3.245928
H	-2.906222	2.411571	3.528271
C	-0.005345	1.810758	3.961015
H	-0.429503	1.197186	4.782045
H	1.096419	1.748986	4.024205
H	-0.294569	2.871020	4.116587
4F cat			
Ag3RX-Clmdo30 SCF Done: -3911.64581900 A.U.			
O	-6.159496	1.841804	-1.455114
C	2.527584	0.102298	3.626459
H	3.407478	-0.236555	3.060562
O	5.286356	-3.056530	-1.804632
C	1.524840	0.744674	5.801636
H	1.632010	0.880560	6.889813
C	2.607958	0.303012	5.040914
H	3.566303	0.096136	5.541651
C	-7.053830	0.840547	-1.927733
H	-6.638115	0.295563	-2.805427
H	-7.976510	1.369076	-2.233222
H	-7.305619	0.101090	-1.133437
C	5.248693	-4.477862	-1.827424
H	5.266854	-4.911188	-0.800767
H	6.156487	-4.803273	-2.369847
H	4.349495	-4.859360	-2.362418
Ag	0.102067	-0.149039	-2.517884
Cl	0.326739	-0.487991	-4.807346
C	0.011359	0.136798	-0.493008
C	2.138238	-2.233313	-0.017197
C	3.296208	-0.182700	-0.768590
N	-1.002431	0.599783	0.319063
N	1.095274	-0.062876	0.342051
C	0.206425	0.823246	3.780094
C	3.323103	1.342961	-0.819515
H	2.266889	1.687380	-0.868736
C	0.949168	-2.866693	0.699125
H	0.094974	-2.168386	0.583824
C	0.779712	0.295022	1.640103
C	-0.972987	1.054723	2.986079
C	-4.922556	1.461746	-1.036202
C	-0.690260	3.529647	-0.909091
C	5.346053	3.538275	-3.266917
H	6.058387	4.378245	-3.231169
C	3.881455	1.859114	0.507849
C	-3.171875	-0.178695	-0.556052
C	-2.763521	2.237757	-0.190363
C	3.964068	1.901652	-2.088262

C	0.264512	1.025274	5.173266
C	2.224510	-0.828943	-0.114216
C	1.185467	-2.950352	2.206507
C	0.447646	-4.146604	0.033686
C	-1.781753	3.362312	0.155147
H	-1.256308	3.059471	1.086550
C	-2.727703	-1.643401	-0.473941
H	-1.628169	-1.653519	-0.639651
C	-2.932071	-2.254168	0.917329
C	-0.282275	-6.475632	0.092146
H	-0.378380	-7.426426	0.640835
C	-2.902076	4.939077	1.803224
H	-2.676736	4.203630	2.593732
C	-4.064702	2.507630	-0.627392
H	-4.437972	3.539913	-0.679656
C	-2.332761	0.883812	-0.147590
C	-0.551807	0.706613	1.631005
C	0.329340	-5.371137	0.716060
H	0.685807	-5.448868	1.754955
C	1.317945	0.357865	2.996194
C	0.157536	-2.545017	3.082010
H	-0.787495	-2.169151	2.664191
C	-2.090291	1.712824	5.035801
H	-3.004687	2.067616	5.536964
C	-4.471564	0.128399	-1.002322
H	-5.114446	-0.698660	-1.330083
C	1.568478	-2.989885	5.011501
H	1.725992	-2.986879	6.101459
C	3.166265	-3.012644	-0.577202
H	3.074760	-4.106321	-0.531373
C	-4.435537	-3.305732	-1.464549
H	-4.905861	-3.401534	-0.473243
C	2.601799	-3.397649	4.148507
H	3.569607	-3.723579	4.562336
C	5.160590	1.471124	0.964491
H	5.781639	0.812939	0.336485
C	-3.344653	-1.489379	2.027320
H	-3.585992	-0.424781	1.893131
C	-2.635757	-3.622761	1.119007
H	-2.314183	-4.237805	0.266067
C	-3.309841	-2.471404	-1.622695
C	-3.230909	-3.085299	-3.992709
H	-2.743517	-2.984682	-4.975254
C	-4.949442	-4.029002	-2.556755
H	-5.825704	-4.681681	-2.413508
C	-2.484317	4.678672	0.481163
C	4.255149	-2.386467	-1.222300
C	-3.593567	6.120607	2.118709
H	-3.908082	6.311099	3.157360
C	4.879421	2.970554	-2.065592
H	5.220467	3.374006	-1.099493
C	4.314056	-0.976872	-1.310355

H	5.152914	-0.519794	-1.854818
C	5.638634	1.907863	2.210812
H	6.638865	1.595856	2.551942
C	3.567780	3.123612	2.581943
H	2.926290	3.752347	3.219198
C	-2.127291	1.505649	3.620255
H	-3.051395	1.701397	3.054615
C	-0.811439	3.028974	-2.220204
H	-1.718079	2.476994	-2.513714
C	0.230207	3.199520	-3.154158
H	0.133870	2.758565	-4.158522
C	3.094428	2.686319	1.333154
H	2.088203	2.969952	0.992604
C	-3.461175	6.811666	-0.208408
H	-3.674787	7.545426	-1.001962
C	-0.788662	-6.362771	-1.214217
H	-1.288601	-7.220595	-1.691107
C	4.842150	2.735156	3.025706
H	5.212709	3.067463	4.008326
C	-2.765251	5.631157	-0.520349
H	-2.428709	5.441631	-1.552028
C	2.411826	-3.377749	2.755473
H	3.233066	-3.676086	2.085132
C	1.523232	4.404013	-1.488054
H	2.446714	4.924238	-1.189239
C	4.904617	3.040610	-4.504694
H	5.268094	3.487857	-5.443649
C	3.992097	1.968314	-4.535450
H	3.621122	1.572053	-5.493702
C	-3.878366	7.060009	1.111421
H	-4.419386	7.987865	1.356176
C	-0.941908	1.484117	5.798881
H	-0.959658	1.657987	6.886870
C	3.525761	1.406743	-3.338482
H	2.787249	0.588971	-3.383707
C	0.484931	4.229703	-0.559856
H	0.576710	4.649338	0.455972
C	0.342218	-2.566260	4.472425
H	-0.471203	-2.221543	5.129298
C	-2.723568	-2.364661	-2.901400
H	-1.849841	-1.706043	-3.053592
C	-2.744993	-4.202356	2.391328
H	-2.488556	-5.265319	2.520693
C	-0.032585	-4.052111	-1.292814
H	0.058949	-3.096175	-1.836803
C	-3.447501	-2.067072	3.306866
H	-3.751738	-1.440567	4.160379
C	-0.657811	-5.144783	-1.909557
H	-1.066639	-5.031967	-2.925650
C	1.401133	3.884090	-2.789647
H	2.229512	3.991563	-3.505867
C	-4.347200	-3.925551	-3.822827

H	-4.747182	-4.497487	-4.675350
C	-3.150204	-3.426941	3.493819
H	-3.219867	-3.879175	4.495490

4F a

Ag3RX-ALKI SCF Done: -3644.79755648 A.U.

Ag	1.332060	0.693939	1.755548
N	0.349545	-0.999480	-0.588938
C	1.797623	0.498420	-2.512751
H	0.918215	0.833244	-1.925486
C	2.487357	-0.587372	-1.691898
C	1.724720	-1.370790	-0.789700
C	0.020259	-0.009275	0.319132
C	2.301832	-2.381609	-0.001754
C	1.416038	-3.192750	0.941908
H	0.605931	-2.516088	1.290038
C	3.856788	-0.849491	-1.804536
C	4.457713	-1.871855	-1.034367
C	3.679282	-2.637465	-0.140802
C	-0.730706	-1.297319	-1.398621
C	-1.769456	-0.453682	-1.010058
N	-1.287717	0.325757	0.037197
C	-2.008764	1.361926	0.726786
C	-1.650825	2.722961	0.525458
C	-3.058449	0.987640	1.597199
C	-2.385298	3.702537	1.206147
C	-3.778682	1.996669	2.261809
C	-3.446313	3.353190	2.067364
C	-0.537027	3.134087	-0.444609
H	0.229710	2.329449	-0.411621
C	-3.347120	-0.494837	1.859597
H	-3.342025	-1.005803	0.872517
C	-2.248869	-1.166425	2.692481
C	-1.322970	-0.439933	3.466024
C	-2.173141	-2.576211	2.696572
C	-0.327608	-1.105395	4.208532
H	-1.359014	0.660530	3.473768
C	-1.183968	-3.242584	3.436518
C	-0.254005	-2.507668	4.194981
H	0.412297	-0.514268	4.769914
H	0.541295	-3.029471	4.748589
C	-4.741856	-0.723346	2.440573
C	-5.849185	-0.839559	1.573788
C	-4.962065	-0.774703	3.832864
C	-7.148896	-0.993597	2.084253
H	-5.684355	-0.811609	0.483720
C	-6.262098	-0.924621	4.346553
C	-7.359996	-1.032752	3.474504
H	-8.001434	-1.089170	1.392807
H	-6.417225	-0.963569	5.436694
H	-8.377970	-1.156051	3.877108

C	0.190665	4.396100	0.026702
C	-0.119927	5.676896	-0.474859
C	1.182691	4.282154	1.023235
C	0.557920	6.814840	-0.001206
C	1.860530	5.415278	1.498405
H	1.437014	3.291687	1.440103
C	1.553054	6.688304	0.983912
H	0.306592	7.807345	-0.408645
H	2.632402	5.291717	2.274790
H	2.086073	7.580201	1.350594
C	-1.005645	3.191901	-1.903502
C	-0.100119	3.626890	-2.899986
C	-2.294655	2.786959	-2.303741
C	-0.476464	3.653473	-4.250496
H	0.910203	3.950251	-2.609377
C	-2.671954	2.804223	-3.659797
C	-1.765482	3.241968	-4.638974
H	0.254821	3.982227	-5.005192
H	-3.679881	2.463007	-3.944805
H	-2.054290	3.249611	-5.701657
C	2.126210	-3.625897	2.223064
C	2.837778	-2.650548	2.960147
C	1.996952	-4.920305	2.761007
C	3.404539	-2.963880	4.203756
H	2.925604	-1.621982	2.571635
C	2.563317	-5.235799	4.011242
C	3.268353	-4.260549	4.736750
H	3.939718	-2.180580	4.763443
H	2.446648	-6.252270	4.420485
H	3.707374	-4.507834	5.716508
C	0.739118	-4.298727	0.130839
C	-0.661536	-4.311764	-0.020227
C	1.498682	-5.280949	-0.542602
C	-1.294046	-5.279087	-0.820007
H	-1.256870	-3.534861	0.481269
C	0.870886	-6.251866	-1.339513
C	-0.529922	-6.252584	-1.483472
H	-2.388292	-5.255032	-0.941920
H	1.479382	-7.010456	-1.857639
H	-1.021671	-7.006124	-2.118779
C	2.628439	1.772177	-2.659569
C	2.971114	2.473463	-1.480522
C	2.952183	2.335759	-3.907218
C	3.587145	3.730939	-1.548477
C	3.585808	3.591341	-3.976916
H	2.674902	1.804087	-4.830427
C	3.892664	4.297457	-2.801252
H	3.802975	4.279064	-0.618328
H	3.825611	4.026499	-4.960484
H	4.367034	5.289936	-2.858167
C	1.218415	-0.065630	-3.810073
C	-0.098825	0.275285	-4.178719

C	1.946636	-0.943129	-4.639217
C	-0.678757	-0.241772	-5.346809
H	-0.690592	0.928865	-3.521922
C	1.368353	-1.465136	-5.809791
C	0.052612	-1.117688	-6.166400
H	-1.717467	0.025198	-5.595722
H	1.947856	-2.156130	-6.442947
H	-0.405974	-1.541527	-7.073690
O	5.798210	-2.031900	-1.209911
C	6.477739	-2.993000	-0.412212
H	6.378333	-2.773399	0.675190
H	6.109683	-4.027309	-0.604281
H	7.545296	-2.933607	-0.696976
O	-4.089202	4.390584	2.669422
C	-5.152373	4.101809	3.568607
H	-5.520710	5.078065	3.936626
H	-5.988628	3.564115	3.065673
H	-4.807803	3.493895	4.436197
C	2.694218	1.386626	3.068493
C	3.548654	1.882107	3.822977
C	4.554825	2.477116	4.672283
H	4.352600	2.417963	5.758654
C	5.299010	3.737446	4.221856
C	6.034648	2.421138	4.284375
H	6.276511	1.916212	3.335443
H	6.769294	2.264950	5.091837
H	5.035821	4.136995	3.229116
H	5.517204	4.503008	4.984876
H	2.725129	2.038112	-0.496147
H	2.970212	-1.234515	-4.356402
H	-0.890936	5.782996	-1.254199
H	-3.017153	2.451558	-1.545773
H	-1.118724	-4.341363	3.402146
H	-2.909814	-3.154411	2.114129
H	-4.100608	-0.701513	4.515525
H	2.595775	-5.275459	-0.444539
H	1.432249	-5.685620	2.206340
H	4.491938	-0.237229	-2.461591
H	4.133279	-3.408602	0.495595
H	-2.139195	4.766914	1.084328
H	-4.592268	1.701006	2.936023
C	-1.149841	-2.131947	-2.520736
C	-0.593312	-3.121638	-3.318343
C	-1.395599	-3.675701	-4.365225
C	-2.706012	-3.262078	-4.608821
H	0.434876	-3.475338	-3.154691
H	-0.951935	-4.455395	-5.003282
H	-3.284239	-3.712447	-5.431528
C	-2.936129	-0.681272	-1.860791
C	-4.226723	-0.177004	-1.993633
C	-2.502967	-1.714470	-2.766564
C	-5.061539	-0.702204	-3.030825

C	-3.308452	-2.245129	-3.793311
C	-4.630537	-1.700642	-3.908549
H	-4.604258	0.611433	-1.324210
H	-6.081131	-0.299207	-3.136766
H	-5.307024	-2.073118	-4.694614
4F a+b			
Ag3RX-OCF3ALKIPh--2Npre SCF Done: -4398.32729812 A.U.			
Ag	1.529330	0.067892	0.739168
N	-1.339744	-0.886854	-0.245869
C	-0.432080	-1.765994	-2.764088
H	-0.362226	-0.737733	-2.353728
C	-0.428133	-2.717225	-1.568024
C	-0.959184	-2.264349	-0.336368
C	-0.428443	0.094290	0.075996
C	-1.069880	-3.083911	0.800216
C	-1.665738	-2.486086	2.078889
H	-1.297091	-1.437447	2.136179
C	0.006161	-4.045438	-1.642839
C	-0.121127	-4.902161	-0.524899
C	-0.659382	-4.426159	0.690196
C	-2.544566	-0.330893	-0.635308
C	-2.389904	1.051277	-0.566649
N	-1.088453	1.287403	-0.140576
C	-0.484466	2.574172	0.084185
C	0.472797	3.078980	-0.841267
C	-0.823898	3.265910	1.267214
C	1.029410	4.334304	-0.570329
C	-0.252772	4.530612	1.501607
C	0.672371	5.064989	0.583630
C	0.916451	2.257037	-2.060034
H	0.947253	1.198956	-1.718141
C	-1.660621	2.573558	2.348598
H	-2.376383	1.899365	1.831573
C	-0.791145	1.671504	3.239251
C	0.613606	1.762848	3.277622
C	-1.423883	0.703246	4.053364
C	1.368553	0.896409	4.093986
H	1.143467	2.492904	2.647983
C	-0.674980	-0.170906	4.855166
C	0.730202	-0.078764	4.873659
H	2.465733	0.979024	4.072775
H	1.314244	-0.779131	5.490972
C	-2.513749	3.557874	3.146442
C	-3.762872	3.967786	2.632672
C	-2.069507	4.103850	4.368484
C	-4.547122	4.909051	3.319146
H	-4.121320	3.538535	1.681891
C	-2.851166	5.050029	5.055060
C	-4.091318	5.456688	4.532643
H	-5.522813	5.212999	2.907186

H	-2.491393	5.466193	6.009762
H	-4.706539	6.192840	5.074035
C	2.343904	2.571688	-2.507294
C	2.631235	3.675254	-3.340491
C	3.403385	1.744957	-2.089489
C	3.955229	3.957451	-3.717297
C	4.726151	2.010264	-2.480706
H	3.206840	0.876450	-1.445929
C	5.007100	3.125855	-3.287238
H	4.166070	4.826320	-4.361386
H	5.516779	1.325925	-2.137841
H	6.043438	3.345370	-3.590102
C	-0.059441	2.280097	-3.238959
C	0.268862	1.529411	-4.393315
C	-1.267424	3.002068	-3.223151
C	-0.583937	1.519587	-5.505644
H	1.203545	0.946109	-4.411182
C	-2.131425	2.983265	-4.335341
C	-1.787553	2.251276	-5.483446
H	-0.314565	0.917513	-6.387233
H	-3.079029	3.543719	-4.294905
H	-2.462521	2.233774	-6.353428
C	-1.156688	-3.136503	3.363214
C	0.238501	-3.270667	3.531525
C	-2.004326	-3.517797	4.420883
C	0.778265	-3.791137	4.715310
H	0.917581	-2.969813	2.719708
C	-1.465184	-4.019284	5.621800
C	-0.074862	-4.160360	5.773050
H	1.871312	-3.911962	4.783756
H	-2.141898	-4.305738	6.442945
H	0.342622	-4.563040	6.709805
C	-3.186908	-2.403126	1.955911
C	-3.848160	-1.166452	2.096471
C	-3.960233	-3.551105	1.680330
C	-5.244334	-1.071161	1.974878
H	-3.249787	-0.261724	2.276990
C	-5.357558	-3.462807	1.563149
C	-6.004742	-2.221600	1.709599
H	-5.734146	-0.089342	2.066721
H	-5.945791	-4.368655	1.345450
H	-7.097855	-2.150035	1.599063
C	0.794337	-1.868429	-3.668968
C	2.060592	-1.584951	-3.108221
C	0.700264	-2.103249	-5.053834
C	3.205559	-1.521650	-3.915967
C	1.849637	-2.049277	-5.865318
H	-0.283577	-2.303439	-5.505037
C	3.101637	-1.752670	-5.301204
H	4.168771	-1.273205	-3.447707
H	1.759619	-2.229766	-6.948727
H	3.997693	-1.694144	-5.939407

C	-1.779768	-1.822506	-3.486831
C	-2.448134	-0.620666	-3.795738
C	-2.390428	-3.043523	-3.841041
C	-3.688130	-0.629646	-4.452365
H	-2.004255	0.336154	-3.488660
C	-3.635942	-3.057610	-4.493252
C	-4.289477	-1.850018	-4.800807
H	-4.191272	0.326446	-4.663391
H	-4.102142	-4.020433	-4.757458
H	-5.272242	-1.862652	-5.297777
O	0.321571	-6.179596	-0.711853
C	0.145724	-7.120957	0.339340
H	0.698991	-6.827366	1.259545
H	-0.929925	-7.252761	0.597728
H	0.550109	-8.081139	-0.032776
O	1.297551	6.265383	0.736175
C	1.021563	7.029259	1.903348
H	1.629586	7.950050	1.822545
H	-0.054478	7.309550	1.972088
H	1.309337	6.484623	2.831584
C	3.360983	0.786773	1.288710
C	4.027565	1.828169	1.454757
C	4.741391	3.070560	1.594982
H	4.856867	3.437331	2.632151
C	4.536757	4.157230	0.527717
C	5.864868	3.463063	0.631726
H	6.120609	2.731077	-0.147413
H	6.721447	4.006158	1.063412
H	3.890291	3.883380	-0.321170
H	4.430590	5.197103	0.876586
O	4.685866	-0.950984	-1.182603
C	4.840722	-1.298653	-0.013049
C	4.084900	-2.432156	0.583886
C	3.713005	-2.632971	1.966562
C	3.687453	-3.434981	-0.331648
N	3.203108	-3.819089	2.393737
C	3.096597	-4.621349	0.111280
C	2.928357	-4.771687	1.501474
H	3.891049	-3.265329	-1.400209
H	2.792427	-5.414830	-0.584869
H	2.535818	-5.717992	1.918843
N	3.788899	-1.650870	2.901198
H	3.804338	-0.669326	2.557760
H	3.289880	-1.843422	3.772382
C	6.161562	-0.799607	0.667189
F	6.127615	-0.609124	1.999575
F	7.095830	-1.766804	0.424639
F	6.598750	0.338972	0.091771
H	2.156781	-1.390276	-2.026161
H	-1.891671	-3.993481	-3.593190
H	1.805906	4.307092	-3.707687
H	-1.534159	3.590305	-2.332539

H	-1.183283	-0.942390	5.453743
H	-2.523709	0.640913	4.064405
H	-1.105831	3.771774	4.786976
H	-3.455013	-4.521947	1.552396
H	-3.094240	-3.407921	4.313377
H	0.459887	-4.436943	-2.565307
H	-0.721517	-5.067332	1.579139
H	1.783737	4.768441	-1.239893
H	-0.520181	5.063104	2.423495
C	-3.894033	-0.669656	-1.077946
C	-4.619385	-1.819798	-1.349800
C	-5.974478	-1.676587	-1.784422
C	-6.585953	-0.433086	-1.945611
H	-4.169680	-2.815993	-1.239919
H	-6.550177	-2.589235	-2.002542
H	-7.632376	-0.370944	-2.285011
C	-3.639444	1.708038	-0.944209
C	-4.122919	3.007896	-1.066237
C	-4.523061	0.611171	-1.248509
C	-5.475549	3.193862	-1.494940
C	-5.855643	0.773678	-1.676995
C	-6.323319	2.124198	-1.795371
H	-3.487914	3.878190	-0.840110
H	-5.856825	4.222693	-1.591601
H	-7.357330	2.316172	-2.124405

4F a+b→c

Ag3RX-OCF3ALKIPh--2N SCF Done: -4398.31947141 A.U.

Ag	1.576062	-0.013774	0.670313
N	-1.318878	-0.863963	-0.230114
C	-0.367723	-1.733719	-2.731481
H	-0.312174	-0.708651	-2.311929
C	-0.399156	-2.694841	-1.543497
C	-0.956891	-2.247676	-0.320882
C	-0.401836	0.111971	0.091435
C	-1.132101	-3.082441	0.797409
C	-1.733181	-2.480463	2.071714
H	-1.318443	-1.449847	2.150132
C	0.006403	-4.031125	-1.625267
C	-0.199436	-4.906721	-0.533880
C	-0.772626	-4.437372	0.668427
C	-2.514216	-0.298379	-0.633412
C	-2.351477	1.082513	-0.561822
N	-1.049130	1.307760	-0.131887
C	-0.440365	2.591147	0.084309
C	0.471066	3.117277	-0.872852
C	-0.745715	3.264834	1.286146
C	0.992530	4.393105	-0.622151
C	-0.201473	4.543221	1.507109
C	0.662169	5.109043	0.548711
C	0.912346	2.294677	-2.093836

H	0.932682	1.235390	-1.754965
C	-1.507795	2.531780	2.393552
H	-2.228142	1.846247	1.898999
C	-0.565276	1.641956	3.219881
C	0.832886	1.823940	3.234415
C	-1.113684	0.599479	4.001726
C	1.658867	0.985741	4.008462
H	1.293070	2.613519	2.620994
C	-0.291934	-0.249375	4.759698
C	1.101787	-0.055658	4.766151
H	2.749148	1.136306	3.985105
H	1.745959	-0.720195	5.363476
C	-2.350476	3.479229	3.244752
C	-3.610640	3.896713	2.763922
C	-1.889997	3.986640	4.476625
C	-4.391161	4.806264	3.495130
H	-3.979394	3.500192	1.802474
C	-2.669509	4.900283	5.209091
C	-3.920956	5.313726	4.720839
H	-5.375176	5.117147	3.109069
H	-2.298064	5.285674	6.172160
H	-4.533821	6.024477	5.297604
C	2.346132	2.602359	-2.525494
C	2.653889	3.730810	-3.318262
C	3.394243	1.754236	-2.122873
C	3.986944	4.023766	-3.652079
C	4.729070	2.036494	-2.461010
H	3.193654	0.845577	-1.537893
C	5.029432	3.181980	-3.216751
H	4.213033	4.910950	-4.265408
H	5.500604	1.333547	-2.109748
H	6.073231	3.415133	-3.481993
C	-0.054626	2.327167	-3.278333
C	0.282121	1.582481	-4.433783
C	-1.266499	3.042073	-3.261044
C	-0.569127	1.570853	-5.547143
H	1.218281	1.001260	-4.449802
C	-2.128589	3.020914	-4.374616
C	-1.777813	2.294019	-5.523902
H	-0.295088	0.973091	-6.430221
H	-3.080096	3.574693	-4.334389
H	-2.451598	2.274028	-6.394743
C	-1.297135	-3.148797	3.375473
C	0.063987	-3.478114	3.556237
C	-2.182149	-3.320990	4.459165
C	0.531378	-3.961555	4.786998
H	0.785733	-3.385588	2.728239
C	-1.712092	-3.793869	5.699585
C	-0.353437	-4.111662	5.871585
H	1.597179	-4.227779	4.878858
H	-2.417735	-3.914045	6.537336
H	0.010976	-4.484007	6.842354

C	-3.246729	-2.325209	1.923539
C	-3.856535	-1.062944	2.069034
C	-4.064942	-3.437628	1.635054
C	-5.246336	-0.906854	1.933395
H	-3.223296	-0.184746	2.265403
C	-5.455740	-3.289179	1.505054
C	-6.051540	-2.022461	1.652586
H	-5.694677	0.094158	2.028881
H	-6.079933	-4.167758	1.276193
H	-7.139428	-1.904328	1.531503
C	0.883613	-1.817634	-3.603816
C	2.134708	-1.558969	-3.000101
C	0.826024	-1.996557	-4.999411
C	3.300402	-1.454406	-3.772438
C	1.996448	-1.905303	-5.776841
H	-0.145443	-2.180844	-5.483557
C	3.232175	-1.625640	-5.168283
H	4.244225	-1.220656	-3.256588
H	1.935837	-2.042175	-6.868746
H	4.143534	-1.535889	-5.780775
C	-1.699013	-1.786375	-3.486039
C	-2.367224	-0.583990	-3.794169
C	-2.299950	-3.005261	-3.864059
C	-3.597769	-0.590214	-4.468646
H	-1.931836	0.371885	-3.472043
C	-3.535930	-3.016940	-4.534103
C	-4.190288	-1.808684	-4.837609
H	-4.100310	0.366680	-4.677330
H	-3.993862	-3.978739	-4.815957
H	-5.165903	-1.819499	-5.348586
O	0.200306	-6.195026	-0.731010
C	-0.101270	-7.160899	0.267956
H	0.407442	-6.938508	1.233752
H	-1.197925	-7.233451	0.451360
H	0.269292	-8.130053	-0.115935
O	1.265878	6.324076	0.683404
C	0.999881	7.086464	1.854662
H	1.574626	8.025972	1.750338
H	-0.082239	7.330619	1.953664
H	1.333198	6.557625	2.776873
C	3.619129	0.281851	1.024666
C	3.941567	1.453741	1.355241
C	4.319854	2.793336	1.671359
H	4.241869	3.082654	2.736026
C	4.076558	3.914429	0.643890
C	5.474614	3.448163	0.885082
H	5.945913	2.816419	0.117343
H	6.164128	4.072839	1.475130
H	3.597098	3.607154	-0.297035
H	3.737652	4.883473	1.043750
O	4.803630	-0.751764	-1.056012
C	4.698736	-1.061811	0.166862

C	4.005308	-2.391429	0.506044
C	3.387962	-2.770777	1.745763
C	3.936439	-3.319813	-0.545578
N	2.766689	-3.966216	1.902229
C	3.308386	-4.563983	-0.376711
C	2.744824	-4.829158	0.878362
H	4.379072	-3.026367	-1.508759
H	3.238238	-5.296549	-1.193647
H	2.231970	-5.788276	1.068619
N	3.360778	-1.956789	2.854714
H	3.585660	-0.966397	2.713514
H	2.626705	-2.169095	3.533538
C	6.033424	-0.838367	0.966017
F	5.920791	-0.897180	2.321060
F	6.913248	-1.806614	0.602193
F	6.598191	0.355970	0.661708
H	2.209870	-1.420198	-1.907797
H	-1.801725	-3.956130	-3.619418
H	1.838073	4.373564	-3.688125
H	-1.539428	3.623934	-2.368123
H	-0.737314	-1.072655	5.338924
H	-2.205388	0.453492	4.023618
H	-0.915673	3.651610	4.867110
H	-3.599569	-4.428276	1.507918
H	-3.246637	-3.066765	4.340784
H	0.486574	-4.418194	-2.535840
H	-0.890448	-5.098436	1.536932
H	1.708766	4.852092	-1.316476
H	-0.439861	5.059442	2.446341
C	-3.860422	-0.626997	-1.093780
C	-4.585461	-1.771121	-1.390234
C	-5.934124	-1.617321	-1.841206
C	-6.539524	-0.369759	-1.993341
H	-4.139097	-2.769918	-1.291334
H	-6.509717	-2.524926	-2.079573
H	-7.581500	-0.299686	-2.344630
C	-3.595479	1.748646	-0.939209
C	-4.073992	3.051746	-1.046512
C	-4.481212	0.658246	-1.260233
C	-5.422048	3.247621	-1.484650
C	-5.808425	0.830977	-1.701383
C	-6.269783	2.184531	-1.808349
H	-3.437879	3.916646	-0.802890
H	-5.799540	4.278739	-1.571281
H	-7.299487	2.384331	-2.146069

4F c

Ag3RX-OCF3ALKIPh--2Npost SCF Done: -4398.32375255 A.U.

Ag	-1.617535	0.000719	-0.746682
N	1.294187	-0.838529	0.146629
C	0.420407	-1.978864	2.575772

H	0.373074	-0.922144	2.244643
C	0.530674	-2.834099	1.314107
C	1.018094	-2.245470	0.123400
C	0.348856	0.135798	-0.102798
C	1.269602	-2.983390	-1.049008
C	1.806478	-2.247360	-2.279044
H	1.305899	-1.251787	-2.284964
C	0.253422	-4.206685	1.299778
C	0.542232	-4.980168	0.153741
C	1.059664	-4.373229	-1.010049
C	2.469929	-0.273841	0.605654
C	2.273726	1.102451	0.641088
N	0.967420	1.324382	0.221873
C	0.323874	2.603053	0.119268
C	-0.585626	3.021345	1.130416
C	0.599444	3.381480	-1.024380
C	-1.136382	4.301599	0.995295
C	0.022348	4.660457	-1.130913
C	-0.838160	5.120584	-0.115199
C	-0.991745	2.078293	2.275627
H	-0.991230	1.055381	1.838783
C	1.371666	2.761216	-2.193536
H	2.112946	2.058781	-1.756815
C	0.448844	1.912983	-3.083613
C	-0.954015	2.051289	-3.060853
C	1.011771	0.951439	-3.952670
C	-1.772554	1.245799	-3.876247
H	-1.415233	2.784500	-2.382915
C	0.198803	0.134829	-4.756004
C	-1.199969	0.280628	-4.719445
H	-2.866949	1.355923	-3.818785
H	-1.837926	-0.364248	-5.344441
C	2.182802	3.796787	-2.967798
C	3.437247	4.202869	-2.462891
C	1.696509	4.397216	-4.146688
C	4.186499	5.193205	-3.117828
H	3.825146	3.733634	-1.542528
C	2.444266	5.392278	-4.802126
C	3.690037	5.793901	-4.289922
H	5.166158	5.495212	-2.714115
H	2.052492	5.851856	-5.723679
H	4.277994	6.568984	-4.806603
C	-2.425260	2.302540	2.757843
C	-2.755044	3.369247	3.624366
C	-3.450525	1.436545	2.334599
C	-4.089267	3.588976	4.006204
C	-4.787655	1.647343	2.714218
H	-3.230160	0.577509	1.687319
C	-5.110219	2.734984	3.542987
H	-4.333364	4.429534	4.675847
H	-5.538902	0.943549	2.321419
H	-6.155676	2.915002	3.841343

C	-0.010669	2.026652	3.448990
C	-0.323894	1.183813	4.541964
C	1.186420	2.764785	3.484235
C	0.536049	1.099107	5.645526
H	-1.250508	0.587789	4.518269
C	2.058630	2.669258	4.585963
C	1.730647	1.845010	5.674424
H	0.280675	0.426399	6.478967
H	2.999313	3.242675	4.584097
H	2.411253	1.766526	6.536593
C	1.435934	-2.855552	-3.633578
C	0.153718	-3.415989	-3.840441
C	2.304397	-2.736768	-4.739097
C	-0.254467	-3.813716	-5.124707
H	-0.535946	-3.567358	-2.988996
C	1.895223	-3.138541	-6.024004
C	0.609832	-3.670124	-6.225593
H	-1.260597	-4.243365	-5.259213
H	2.588023	-3.029495	-6.873828
H	0.287450	-3.979866	-7.232393
C	3.303833	-1.976151	-2.112037
C	3.803004	-0.658457	-2.125325
C	4.213974	-3.036694	-1.921239
C	5.172075	-0.396321	-1.944914
H	3.098505	0.176685	-2.250511
C	5.584590	-2.782029	-1.749050
C	6.068280	-1.460328	-1.755501
H	5.530842	0.644612	-1.933646
H	6.280739	-3.621824	-1.594542
H	7.139348	-1.261395	-1.596766
C	-0.889155	-2.148723	3.343114
C	-2.092937	-1.919799	2.640639
C	-0.942168	-2.359290	4.733590
C	-3.325003	-1.858656	3.306826
C	-2.178917	-2.320006	5.406953
H	-0.008624	-2.524423	5.293731
C	-3.366082	-2.057147	4.700455
H	-4.224875	-1.619715	2.712532
H	-2.208827	-2.480067	6.497091
H	-4.327038	-2.000205	5.236300
C	1.710832	-2.070774	3.394784
C	2.369321	-0.881976	3.770008
C	2.298697	-3.303301	3.749793
C	3.582678	-0.914338	4.474459
H	1.941484	0.086581	3.474602
C	3.515973	-3.341246	4.451967
C	4.165309	-2.146038	4.813011
H	4.079314	0.033801	4.731156
H	3.963280	-4.313642	4.713972
H	5.127001	-2.177589	5.349178
O	0.268237	-6.313185	0.254315
C	0.512002	-7.142802	-0.875397

H	-0.091924	-6.828556	-1.757416
H	1.589028	-7.148100	-1.160671
H	0.212875	-8.165685	-0.578390
O	-1.475365	6.326958	-0.138891
C	-1.252575	7.189567	-1.247702
H	-1.851135	8.100303	-1.057562
H	-0.179616	7.472288	-1.343927
H	-1.588360	6.727812	-2.204526
C	-3.795511	0.214769	-0.772607
C	-3.853429	1.436826	-1.059340
C	-4.060785	2.830471	-1.310460
H	-3.687951	3.211086	-2.279390
C	-4.129266	3.853919	-0.169659
C	-5.401265	3.431911	-0.835504
H	-6.066639	2.740783	-0.294880
H	-5.902749	4.116343	-1.538554
H	-3.928501	3.474046	0.842039
H	-3.708412	4.848643	-0.385914
O	-5.215265	-0.840740	0.846397
C	-4.719174	-1.022721	-0.349751
C	-3.962648	-2.381306	-0.536211
C	-2.941825	-2.678498	-1.483790
C	-4.297719	-3.399509	0.365358
N	-2.261842	-3.842297	-1.499706
C	-3.622400	-4.630248	0.335595
C	-2.598664	-4.790133	-0.607737
H	-5.080724	-3.169594	1.103977
H	-3.865685	-5.437822	1.042110
H	-2.012061	-5.723095	-0.642287
N	-2.497964	-1.707652	-2.403784
H	-3.246340	-1.156129	-2.835990
H	-1.823711	-2.066493	-3.087981
C	-5.854073	-0.976280	-1.454729
F	-5.360789	-1.068231	-2.750472
F	-6.710751	-2.007520	-1.300893
F	-6.569703	0.167696	-1.400341
H	-2.067506	-1.765044	1.547907
H	1.809393	-4.245610	3.460516
H	-1.955077	4.020751	4.013132
H	1.441744	3.424619	2.641781
H	0.658220	-0.626261	-5.404912
H	2.106572	0.842516	-4.007730
H	0.726626	4.072225	-4.556305
H	3.836674	-4.071635	-1.899914
H	3.307714	-2.306525	-4.597667
H	-0.193053	-4.698955	2.176055
H	1.249776	-4.960432	-1.917181
H	-1.854926	4.682790	1.732319
H	0.234251	5.261707	-2.024958
C	3.816628	-0.608059	1.058602
C	4.559623	-1.756617	1.284938
C	5.895472	-1.609860	1.774127

C	6.472414	-0.364532	2.025670
H	4.131802	-2.753554	1.111642
H	6.484440	-2.520745	1.961839
H	7.505584	-0.299925	2.403065
C	3.500417	1.765381	1.078057
C	3.950749	3.066449	1.283754
C	4.406444	0.673534	1.330148
C	5.288954	3.257698	1.754064
C	5.722349	0.840006	1.805484
C	6.153957	2.191496	2.015932
H	3.299797	3.934010	1.094706
H	5.643331	4.287482	1.918912
H	7.173884	2.387194	2.384367
4F a+b→c'			
Ag3RX-OCF3ALKIPhISOMER1--2O SCF Done: -4398.30479454 A.U.			
Ag	-1.216935	-0.128435	-1.269314
N	0.459012	0.918792	1.073834
C	-0.325612	3.685904	1.145157
H	0.714155	3.539608	1.508964
C	-1.179549	2.601844	1.814513
C	-0.761457	1.246272	1.767036
C	0.465872	0.216146	-0.110947
C	-1.514748	0.206581	2.361625
C	-1.031133	-1.246959	2.359906
H	-0.431631	-1.378756	1.432087
C	-2.376257	2.897220	2.472840
C	-3.145014	1.876258	3.072118
C	-2.707339	0.540513	3.025701
C	1.759481	1.164019	1.491469
C	2.607747	0.582263	0.549428
N	1.797951	-0.013101	-0.405281
C	2.173957	-1.009872	-1.373404
C	2.292528	-0.662514	-2.736925
C	2.296526	-2.345087	-0.929706
C	2.506270	-1.686781	-3.669054
C	2.517967	-3.356162	-1.883020
C	2.599557	-3.032127	-3.253594
C	2.249312	0.806381	-3.146588
H	1.625088	1.341931	-2.399263
C	2.206497	-2.670082	0.558764
H	1.539764	-1.910688	1.017733
C	1.505110	-3.997060	0.842620
C	0.162476	-4.133399	0.426786
C	2.094550	-5.033236	1.590022
C	-0.591209	-5.260350	0.781831
H	-0.303729	-3.320054	-0.151769
C	1.346053	-6.175042	1.934176
C	0.000909	-6.287126	1.542271
H	-1.653008	-5.316252	0.495777
H	-0.590419	-7.169162	1.834608

C	3.554037	-2.498116	1.257289
C	3.598804	-1.843449	2.505151
C	4.758694	-2.961332	0.690810
C	4.815628	-1.657740	3.177804
H	2.669997	-1.446402	2.938792
C	5.980400	-2.775454	1.361615
C	6.012553	-2.121164	2.606289
H	4.823656	-1.123331	4.140303
H	6.915377	-3.134509	0.902402
H	6.972150	-1.958790	3.121670
C	1.539235	1.041973	-4.477142
C	2.038073	1.913092	-5.463204
C	0.261203	0.467060	-4.663677
C	1.272016	2.213471	-6.606063
C	-0.507596	0.771347	-5.794926
H	-0.150152	-0.208616	-3.896037
C	-0.004193	1.650252	-6.773507
H	1.674653	2.903465	-7.364797
H	-1.512044	0.331659	-5.900266
H	-0.607541	1.896624	-7.661550
C	3.664135	1.375390	-3.016928
C	3.943043	2.366819	-2.055642
C	4.729379	0.877147	-3.800392
C	5.248424	2.859415	-1.883317
H	3.126999	2.738353	-1.419472
C	6.034173	1.367546	-3.632355
C	6.298807	2.362294	-2.671388
H	5.444899	3.621563	-1.113157
H	6.851913	0.968386	-4.253654
H	7.323658	2.742441	-2.534843
C	-2.181983	-2.246354	2.213181
C	-2.733423	-2.463604	0.933357
C	-2.718840	-2.962501	3.302064
C	-3.749261	-3.405811	0.727959
H	-2.365782	-1.871887	0.077379
C	-3.748557	-3.901268	3.105021
C	-4.258383	-4.135114	1.816804
H	-4.169724	-3.544141	-0.279466
H	-4.152221	-4.454677	3.968109
H	-5.067645	-4.865021	1.660178
C	-0.058096	-1.551346	3.508501
C	0.380329	-2.878903	3.727207
C	0.485390	-0.531294	4.316612
C	1.328409	-3.170921	4.719098
H	-0.025580	-3.692795	3.109853
C	1.442528	-0.820190	5.306865
C	1.866485	-2.142945	5.515317
H	1.660300	-4.212186	4.854173
H	1.861369	0.001066	5.909870
H	2.619986	-2.372110	6.285118
C	-0.269658	3.560703	-0.380159
C	-1.334931	3.019372	-1.130130

C	0.860206	4.063267	-1.059567
C	-1.256909	2.982935	-2.537690
C	0.926685	4.038789	-2.463325
H	1.690440	4.497080	-0.476338
C	-0.135454	3.496833	-3.208169
H	-2.086068	2.537465	-3.104634
H	1.820765	4.424839	-2.978213
H	-0.080080	3.456260	-4.306553
C	-0.717737	5.097927	1.577985
C	-0.059380	5.705006	2.667314
C	-1.753646	5.804525	0.931367
C	-0.429252	6.987037	3.108591
H	0.757895	5.162467	3.172190
C	-2.129048	7.084055	1.373674
C	-1.468890	7.679905	2.463481
H	0.099585	7.448381	3.958122
H	-2.941951	7.621376	0.859669
H	-1.760766	8.685398	2.806156
O	-4.298889	2.283058	3.668138
C	-5.077431	1.327431	4.377199
H	-5.467172	0.528390	3.708417
H	-4.497734	0.853834	5.202558
H	-5.933503	1.881933	4.805641
O	2.727471	-3.952595	-4.250053
C	2.731308	-5.332132	-3.900212
H	2.809250	-5.892674	-4.850880
H	3.599863	-5.594439	-3.253938
H	1.792300	-5.626056	-3.378037
C	-3.162631	-0.343790	-2.245722
C	-2.616474	-1.367828	-2.744775
C	-2.149842	-2.543962	-3.451779
H	-2.546133	-2.632846	-4.479202
C	-1.911992	-3.856226	-2.701431
C	-0.735678	-3.082985	-3.232041
H	-0.106522	-2.536591	-2.510563
H	-0.188374	-3.457561	-4.111331
H	-2.097905	-3.845700	-1.616250
H	-2.213557	-4.786194	-3.209531
O	-3.693629	1.205150	-0.061558
C	-4.400080	0.600070	-0.909671
C	-5.286877	-0.544850	-0.406896
C	-5.938725	-1.595686	-1.139087
C	-5.517170	-0.514430	0.981240
N	-6.759945	-2.494463	-0.533498
C	-6.364072	-1.438898	1.603946
C	-6.967054	-2.406687	0.784204
H	-4.999327	0.276539	1.542122
H	-6.547377	-1.420705	2.688901
H	-7.651831	-3.160458	1.217253
N	-5.744820	-1.834609	-2.476129
H	-5.359498	-1.099485	-3.065852
H	-6.405199	-2.494433	-2.889417

C	-5.098214	1.545755	-1.943080
F	-4.266068	2.521249	-2.367890
F	-5.597239	0.937822	-3.065106
F	-6.151484	2.147599	-1.333329
H	-2.234040	2.611331	-0.630597
H	-2.267493	5.343378	0.073143
H	3.024606	2.381884	-5.326810
H	4.531788	0.092686	-4.547514
H	1.815555	-6.974529	2.529548
H	3.134737	-4.928868	1.934434
H	4.742544	-3.458052	-0.292016
H	0.156271	0.507310	4.168594
H	-2.310971	-2.801562	4.312347
C	4.002383	0.797269	0.927749
C	3.910043	1.532823	2.159964
C	5.257630	0.495935	0.417552
C	2.548302	1.785375	2.553081
C	5.030065	1.962053	2.899674
C	6.408039	0.925233	1.152159
H	5.374830	-0.050786	-0.528948
C	2.310989	2.492243	3.728598
C	4.753900	2.679888	4.109999
C	6.315595	1.630863	2.352293
H	7.402597	0.681980	0.747756
C	3.434787	2.929497	4.498587
H	1.286602	2.715542	4.064884
H	5.588887	3.039035	4.732972
H	7.229594	1.937530	2.885714
H	3.246767	3.486682	5.429829
H	-2.750183	3.928462	2.528233
H	-3.299645	-0.269264	3.471313
H	2.550011	-1.463152	-4.744717
H	2.563858	-4.396307	-1.533331
4F c’			
Ag3RX-OCF3ALKIPhISOMER1--2Opost SCF Done: -4398.34114647 A.U.			
Ag	1.504289	-0.343670	0.780265
N	-0.790425	1.415015	-0.099249
C	-0.239282	3.712295	1.558195
H	-1.317951	3.493205	1.413734
C	0.435426	3.537805	0.190583
C	0.154353	2.383964	-0.587862
C	-0.414032	0.136620	0.270052
C	0.781525	2.150623	-1.834317
C	0.400619	0.963388	-2.724205
H	0.045382	0.156020	-2.046885
C	1.367903	4.457487	-0.304716
C	2.016760	4.235157	-1.540214
C	1.717807	3.089539	-2.299721
C	-2.169544	1.547466	-0.038483
C	-2.678805	0.316735	0.373068

N	-1.596863	-0.537838	0.522373
C	-1.641034	-1.976380	0.585096
C	-1.361871	-2.645186	1.797521
C	-1.870348	-2.677614	-0.620942
C	-1.303067	-4.045704	1.784842
C	-1.822974	-4.082742	-0.603578
C	-1.529522	-4.767139	0.594165
C	-1.201679	-1.845524	3.088586
H	-0.910547	-0.813483	2.798297
C	-2.181238	-1.921902	-1.910552
H	-1.697918	-0.925849	-1.829114
C	-1.535467	-2.544140	-3.149719
C	-0.126026	-2.600226	-3.204250
C	-2.275106	-2.944714	-4.278443
C	0.534511	-2.993766	-4.376474
H	0.458786	-2.285701	-2.326342
C	-1.615726	-3.366571	-5.448451
C	-0.211518	-3.377540	-5.507008
H	1.634461	-2.961310	-4.417252
H	0.303591	-3.676415	-6.433526
C	-3.673182	-1.632480	-2.067675
C	-4.073700	-0.364434	-2.535118
C	-4.663992	-2.587246	-1.763574
C	-5.431502	-0.053034	-2.699075
H	-3.310076	0.399069	-2.742698
C	-6.025942	-2.279070	-1.928365
C	-6.414249	-1.010545	-2.394783
H	-5.715738	0.952953	-3.044499
H	-6.789919	-3.032493	-1.678001
H	-7.481858	-0.763670	-2.504516
C	-0.051968	-2.323248	3.972695
C	-0.140268	-2.350820	5.378520
C	1.199372	-2.581681	3.369993
C	0.998498	-2.622893	6.160346
C	2.340994	-2.830749	4.143699
H	1.298155	-2.555651	2.272052
C	2.242344	-2.854888	5.547397
H	0.912268	-2.636533	7.258804
H	3.307123	-2.971297	3.638866
H	3.136690	-3.045093	6.161738
C	-2.578871	-1.730229	3.743706
C	-3.273617	-0.504917	3.698956
C	-3.219909	-2.846590	4.325404
C	-4.575673	-0.390372	4.216767
H	-2.791425	0.366141	3.230931
C	-4.517106	-2.735304	4.850876
C	-5.202120	-1.506144	4.795468
H	-5.103390	0.574180	4.154211
H	-4.999910	-3.616011	5.303955
H	-6.222247	-1.421595	5.202620
C	1.619277	0.367439	-3.430953
C	2.465634	-0.502086	-2.709424

C	1.948017	0.667701	-4.767916
C	3.588553	-1.087708	-3.309789
H	2.244802	-0.725262	-1.649819
C	3.082356	0.093890	-5.372508
C	3.901040	-0.788844	-4.648731
H	4.233793	-1.758439	-2.721900
H	3.324044	0.340080	-6.419078
H	4.793396	-1.231923	-5.117670
C	-0.800832	1.282812	-3.628666
C	-1.192918	0.373048	-4.638786
C	-1.597010	2.429770	-3.424638
C	-2.343959	0.602770	-5.408157
H	-0.590567	-0.527574	-4.821370
C	-2.754962	2.658946	-4.189625
C	-3.133828	1.745923	-5.187481
H	-2.631155	-0.133897	-6.174577
H	-3.365578	3.554165	-3.991359
H	-4.043428	1.917434	-5.784200
C	0.275412	2.683350	2.567351
C	1.632040	2.301136	2.607093
C	-0.618361	2.099931	3.488308
C	2.090590	1.356758	3.544450
C	-0.166934	1.150027	4.420668
H	-1.680656	2.396795	3.469470
C	1.187677	0.771307	4.447393
H	3.139062	1.028780	3.509840
H	-0.882506	0.678592	5.113136
H	1.530902	-0.004761	5.148368
C	-0.175839	5.148423	2.078762
C	-1.211866	6.049303	1.751602
C	0.906105	5.616784	2.853187
C	-1.166148	7.387223	2.177180
H	-2.070858	5.690770	1.160495
C	0.956686	6.955945	3.277263
C	-0.077693	7.846417	2.939963
H	-1.987704	8.073590	1.916391
H	1.809580	7.303837	3.881594
H	-0.039547	8.894450	3.277102
O	2.931375	5.174943	-1.909407
C	3.532443	5.064040	-3.197616
H	4.120801	4.125322	-3.301111
H	2.768463	5.101604	-4.007096
H	4.212088	5.931106	-3.295976
O	-1.401167	-6.121906	0.685234
C	-1.612124	-6.903480	-0.485397
H	-1.468671	-7.958078	-0.183413
H	-2.643644	-6.777080	-0.886660
H	-0.881230	-6.651957	-1.287582
C	4.225283	-2.289445	0.361800
C	3.748162	-3.368936	0.023300
C	3.085521	-4.577692	-0.389073
H	3.525144	-5.519423	-0.014262

C	2.407687	-4.632641	-1.757086
C	1.560099	-4.544504	-0.514974
H	1.063158	-3.587597	-0.283743
H	1.008774	-5.433186	-0.172038
H	2.503718	-3.733022	-2.383900
H	2.446277	-5.585745	-2.308654
O	3.396346	-0.470055	1.593506
C	4.498201	-0.932216	0.904595
C	4.979936	0.049050	-0.207080
C	5.890582	-0.268372	-1.267362
C	4.502625	1.363465	-0.153941
N	6.252532	0.637984	-2.205801
C	4.896504	2.315137	-1.110266
C	5.768938	1.884536	-2.120626
H	3.808685	1.619153	0.660135
H	4.544750	3.355078	-1.057521
H	6.107078	2.585817	-2.907856
N	6.408825	-1.531581	-1.460487
H	6.426213	-2.186746	-0.679705
H	7.181607	-1.571879	-2.126961
C	5.637934	-1.038643	1.979552
F	5.323103	-1.908601	2.968417
F	6.820655	-1.470347	1.441347
F	5.872742	0.162448	2.550621
H	2.336305	2.731813	1.877808
H	1.712180	4.921501	3.133904
H	-1.101130	-2.135439	5.870694
H	-2.693381	-3.812830	4.365458
H	-2.207334	-3.668170	-6.327763
H	-3.374197	-2.891417	-4.253973
H	-4.368267	-3.575366	-1.377806
H	-1.311551	3.155346	-2.649815
H	1.300929	1.344936	-5.347314
C	-4.133768	0.391123	0.491361
C	-4.438292	1.741538	0.101108
C	-5.173129	-0.450120	0.864409
C	-3.261884	2.495898	-0.243278
C	-5.744944	2.266961	0.057392
C	-6.507675	0.066370	0.833160
H	-4.983947	-1.487004	1.175938
C	-3.410861	3.818182	-0.652434
C	-5.866646	3.630555	-0.369297
C	-6.800431	1.373351	0.443322
H	-7.331128	-0.604298	1.123067
C	-4.730031	4.368594	-0.710838
H	-2.539977	4.433585	-0.926368
H	-6.863563	4.096543	-0.426128
H	-7.844313	1.725455	0.428173
H	-4.847473	5.414553	-1.035127
H	1.620479	5.363533	0.263982
H	2.223512	2.889441	-3.252818
H	-1.041304	-4.601458	2.696736

H	-1.956755	-4.616945	-1.553649

4H cat

Ag4 SCF Done: -4059.15856054 A.U.

Ag	-0.023768	1.152759	-2.077192
Cl	-0.029754	1.918573	-4.268535
N	1.120436	0.125027	0.604776
C	1.777834	-2.063476	-1.384669
H	0.739404	-1.906428	-1.043279
C	2.752522	-1.033340	-0.811278
C	2.451432	0.003822	0.088446
C	0.010539	0.478936	-0.141978
C	3.473374	0.848156	0.545990
C	3.323114	2.032030	1.504563
H	2.280025	2.188828	1.835709
C	4.076599	-1.215802	-1.258187
C	5.109020	-0.366325	-0.814604
C	4.797280	0.657077	0.100906
C	0.748521	-0.311050	1.867307
C	-0.638111	-0.227175	1.928361
N	-1.065206	0.253039	0.699783
C	-2.436875	0.269560	0.281917
C	-3.080521	-0.960532	0.056485
C	-3.136949	1.469772	0.090910
C	-4.438824	-0.988211	-0.313514
C	-4.492403	1.434511	-0.301753
C	-5.159496	0.209317	-0.486717
C	-2.449551	-2.356448	0.135002
H	-1.382569	-2.336807	0.424090
C	-2.595912	2.891851	0.252238
H	-1.536808	2.914044	0.567069
C	-2.827002	3.557586	-1.109505
C	-1.848946	4.115382	-1.938679
C	-4.181160	3.520060	-1.517442
C	-2.227796	4.636645	-3.192248
H	-0.791404	4.121406	-1.632244
C	-4.560605	4.051086	-2.754871
C	-3.572970	4.610578	-3.593529
H	-1.454935	5.034126	-3.866404
H	-5.614943	4.017414	-3.073570
H	-3.858823	5.012996	-4.578124
C	-3.538192	3.582034	1.243267
C	-3.178898	4.196202	2.448272
C	-4.893985	3.549365	0.833493
C	-4.180708	4.782643	3.250944
H	-2.123304	4.220631	2.764092
C	-5.887731	4.130110	1.629639
C	-5.525435	4.749751	2.844966
H	-3.904976	5.268853	4.200152
H	-6.940970	4.105103	1.305385
H	-6.301498	5.211055	3.476167
C	-2.683996	-2.983678	-1.243692

C	-4.050413	-3.006956	-1.612490
C	-1.707026	-3.485344	-2.110497
C	-4.437975	-3.522461	-2.854323
C	-2.096038	-3.998304	-3.365378
H	-0.645100	-3.482510	-1.825048
C	-3.451230	-4.015400	-3.734742
H	-5.501445	-3.533331	-3.142124
H	-1.326631	-4.376325	-4.056326
H	-3.747389	-4.412931	-4.718263
C	-3.315750	-3.145057	1.119920
C	-2.878590	-3.786423	2.284028
C	-4.684254	-3.167771	0.754587
C	-3.819361	-4.454422	3.096052
H	-1.815589	-3.752214	2.570386
C	-5.616761	-3.831277	1.560740
C	-5.177996	-4.475821	2.737827
H	-3.483895	-4.955986	4.017359
H	-6.681187	-3.850175	1.275446
H	-5.906414	-4.999828	3.376832
C	3.901305	3.238374	0.756858
C	3.237320	4.435742	0.468890
C	5.237479	3.038949	0.334449
C	3.916339	5.444389	-0.246761
H	2.194374	4.583947	0.791975
C	5.912024	4.040031	-0.373409
C	5.243687	5.248435	-0.663740
H	3.399596	6.387655	-0.483442
H	6.952245	3.881848	-0.701401
H	5.765283	6.039977	-1.224514
C	4.282305	1.736186	2.659872
C	3.947807	1.634355	4.014937
C	5.614254	1.523205	2.228385
C	4.953699	1.311568	4.949884
H	2.907001	1.786095	4.341520
C	6.612072	1.203775	3.156863
C	6.275493	1.097096	4.523516
H	4.697527	1.223511	6.017415
H	7.647266	1.033025	2.820390
H	7.055663	0.843963	5.258815
C	1.919243	-1.959211	-2.908569
C	3.250512	-2.145625	-3.350952
C	0.900302	-1.667063	-3.821938
C	3.564378	-2.043304	-4.710633
C	1.218680	-1.551136	-5.190670
H	-0.133110	-1.506037	-3.479262
C	2.537891	-1.741955	-5.631844
H	4.602214	-2.187399	-5.053272
H	0.427135	-1.281268	-5.905378
H	2.778027	-1.643227	-6.702286
C	2.348086	-3.424874	-0.983973
C	1.693688	-4.420897	-0.250061
C	3.678271	-3.615647	-1.430664

C	2.372333	-5.625204	0.033364
H	0.661003	-4.265355	0.102439
C	4.351366	-4.809329	-1.147414
C	3.690439	-5.817724	-0.413553
H	1.864641	-6.415898	0.607995
H	5.386970	-4.957047	-1.493729
H	4.213863	-6.760489	-0.188462
O	6.400778	-0.528544	-1.268768
C	6.650738	0.176977	-2.492528
H	7.699892	-0.031968	-2.778752
H	5.968956	-0.163610	-3.305840
H	6.512902	1.275370	-2.361185
O	-6.492463	0.177995	-0.841854
C	-7.373015	0.192474	0.286010
H	-7.226930	1.107171	0.907205
H	-8.407977	0.178740	-0.108152
H	-7.214704	-0.699634	0.936814
C	-1.117952	-0.770803	3.197319
C	-2.341194	-1.001302	3.818194
C	0.089685	-1.173286	3.870185
C	-2.341211	-1.621424	5.108052
H	-3.290514	-0.736769	3.328943
C	0.110418	-1.783744	5.140259
C	-1.166071	-2.001826	5.759527
H	-3.310483	-1.812550	5.593867
H	-1.214992	-2.479429	6.751341
C	1.272084	-0.913813	3.090861
C	2.512642	-1.269909	3.610579
C	2.554930	-1.876821	4.906328
H	3.442841	-1.086108	3.053010
C	1.404253	-2.132606	5.655283
H	3.538051	-2.150096	5.319950
H	1.485065	-2.608948	6.645633
C	-5.095868	2.825771	-0.500142
C	5.782846	1.656771	0.709792
C	4.230103	-2.410767	-2.202146
C	-4.970353	-2.404033	-0.542675
H	-6.150682	2.781671	-0.825961
H	-6.035633	-2.408320	-0.835279
H	5.274790	-2.553197	-2.532413
H	6.819254	1.480985	0.369877

4H a

Ag4RX-ALKI SCF Done: -3792.31620910 A.U.

Ag	-1.056945	1.247742	-0.816034
N	-0.663568	-1.325728	0.656568
C	-2.609924	-0.299827	2.694285
H	-1.573503	-0.533185	3.001105
C	-2.972967	-0.868998	1.320246
C	-2.073862	-1.427544	0.399153
C	0.042946	-0.265093	0.105889

C	-2.517711	-1.775208	-0.888688
C	-1.650270	-2.261714	-2.055533
H	-0.606457	-2.471498	-1.756643
C	-4.293110	-0.574278	0.926291
C	-4.728610	-0.846055	-0.384526
C	-3.844279	-1.491329	-1.270622
C	0.149408	-2.103739	1.459909
C	1.421246	-1.538734	1.396764
N	1.334930	-0.430257	0.564240
C	2.471498	0.341181	0.153106
C	2.644113	1.677081	0.555059
C	3.458969	-0.283956	-0.632284
C	3.823059	2.368579	0.199826
C	4.616713	0.425784	-1.007671
C	4.810261	1.757444	-0.595117
C	1.682975	2.532031	1.385843
H	0.744365	2.006163	1.631275
C	3.411711	-1.701673	-1.219137
H	2.502732	-2.262727	-0.934737
C	3.545108	-1.517686	-2.734599
C	2.653540	-1.976579	-3.710178
C	4.708104	-0.799360	-3.104276
C	2.912823	-1.694340	-5.068459
H	1.757936	-2.549380	-3.420964
C	4.969239	-0.525120	-4.451338
C	4.060827	-0.972144	-5.435372
H	2.207632	-2.040252	-5.840164
H	5.874708	0.034362	-4.737465
H	4.255982	-0.754915	-6.497401
C	4.701313	-2.389359	-0.763806
C	4.799142	-3.593597	-0.057495
C	5.865612	-1.669252	-1.124016
C	6.073856	-4.076937	0.304077
H	3.889290	-4.143882	0.228412
C	7.130574	-2.148090	-0.763271
C	7.230353	-3.358142	-0.043737
H	6.159049	-5.019491	0.867315
H	8.035912	-1.583849	-1.038979
H	8.222307	-3.740903	0.244331
C	1.442309	3.807781	0.563110
C	2.637465	4.489423	0.233617
C	0.205488	4.296467	0.127505
C	2.600146	5.662397	-0.529607
C	0.167605	5.471245	-0.651945
H	-0.729086	3.761167	0.351788
C	1.353055	6.152409	-0.974884
H	3.534497	6.191641	-0.779414
H	-0.805620	5.833825	-1.017667
H	1.313382	7.069605	-1.584095
C	2.487420	2.959536	2.615226
C	2.150209	2.734032	3.954596
C	3.684897	3.637873	2.281294

C	3.014417	3.194575	4.970376
H	1.219639	2.202657	4.211347
C	4.542911	4.093361	3.288492
C	4.201669	3.869468	4.639433
H	2.755176	3.023373	6.027137
H	5.474564	4.620222	3.026240
H	4.871381	4.226184	5.437993
C	-1.752305	-1.149806	-3.109767
C	-0.693779	-0.396353	-3.631171
C	-3.086694	-0.868913	-3.484416
C	-0.974440	0.647276	-4.537746
H	0.342209	-0.597672	-3.319777
C	-3.364313	0.170360	-4.378880
C	-2.299386	0.931196	-4.904509
H	-0.147991	1.255448	-4.936607
H	-4.405789	0.397278	-4.658449
H	-2.512043	1.762192	-5.594842
C	-2.368245	-3.479384	-2.636163
C	-1.833339	-4.761622	-2.806358
C	-3.703003	-3.200876	-3.018143
C	-2.637916	-5.775414	-3.367619
H	-0.794770	-4.973466	-2.503666
C	-4.501483	-4.207670	-3.573299
C	-3.962457	-5.499622	-3.748284
H	-2.225033	-6.787121	-3.507216
H	-5.540423	-3.989444	-3.868655
H	-4.585152	-6.296317	-4.185396
C	-2.868940	1.205384	2.564618
C	-4.191235	1.505150	2.161187
C	-1.936355	2.228567	2.757947
C	-4.569946	2.830379	1.924286
C	-2.320466	3.566308	2.531003
H	-0.911198	1.988107	3.079794
C	-3.625051	3.862120	2.105154
H	-5.593446	3.063012	1.588644
H	-1.586836	4.375174	2.669915
H	-3.912143	4.905766	1.903454
C	-3.655374	-0.849272	3.662824
C	-3.406142	-1.594450	4.821421
C	-4.982016	-0.543682	3.273313
C	-4.494748	-2.030862	5.605393
H	-2.370807	-1.835882	5.112381
C	-6.061284	-0.977812	4.052214
C	-5.812130	-1.722620	5.224207
H	-4.310062	-2.615716	6.520506
H	-7.093645	-0.741919	3.747613
H	-6.656836	-2.066941	5.841825
O	-5.960946	-0.414100	-0.818536
C	-5.902527	0.916181	-1.373750
H	-5.174335	0.976197	-2.211823
H	-6.920667	1.160306	-1.732773
H	-5.574686	1.659828	-0.615089

O	5.942000	2.448830	-0.975652
C	5.750792	3.220032	-2.168722
H	6.709104	3.733306	-2.380061
H	5.481323	2.568295	-3.032336
H	4.944597	3.979343	-2.041295
C	2.350329	-2.289948	2.236656
C	3.697729	-2.237915	2.577154
C	1.530465	-3.330196	2.800312
C	4.209978	-3.227654	3.475810
H	4.365360	-1.470362	2.158465
C	2.013117	-4.305227	3.695814
C	3.409179	-4.230778	4.024248
H	5.279822	-3.191843	3.733120
H	3.845632	-4.970322	4.714696
C	0.160785	-3.255027	2.361772
C	-0.753496	-4.179544	2.858437
C	-0.282729	-5.176192	3.772247
H	-1.814480	-4.150248	2.566342
C	1.050740	-5.252210	4.181464
H	-1.005814	-5.908728	4.163940
H	1.367611	-6.038392	4.885486
C	5.559574	-0.383080	-1.899200
C	-4.110459	-1.750962	-2.755006
C	-5.055475	0.249251	1.967187
C	3.887759	3.786687	0.769230
H	6.466608	0.185479	-2.172229
H	4.831864	4.298493	0.509448
H	-6.088982	0.471641	1.646487
H	-5.157183	-1.532963	-3.033237
C	-2.465080	2.581783	-1.375439
C	-3.434492	3.329163	-1.586840
C	-4.590484	4.159457	-1.829031
H	-5.110944	4.510996	-0.916503
C	-5.504721	3.890546	-3.029823
C	-4.639751	5.127669	-3.011543
H	-3.754226	5.150190	-3.666600
H	-5.123373	6.106031	-2.854054
H	-5.208737	3.065317	-3.697362
H	-6.592545	4.002037	-2.886461
4H a+b→c			
Ag4RX-OCF3ALKIPh--2N SCF Done: -4545.83537746 A.U.			
Ag	-0.187454	-1.452192	-0.651143
C	-0.409880	-3.376287	-1.460827
C	-1.581551	-3.283351	-1.911424
C	-2.905902	-3.090711	-2.427769
H	-2.962940	-2.798709	-3.492213
C	-3.952045	-2.428753	-1.519441
C	-4.101572	-3.877589	-1.877796
H	-3.882918	-4.620912	-1.096531
H	-4.868223	-4.170063	-2.613507

H	-3.626169	-2.179923	-0.497429
H	-4.590782	-1.674911	-2.003955
O	0.603994	-4.873530	0.448165
C	0.922390	-4.562360	-0.736193
C	2.264403	-3.830571	-0.948517
C	2.662923	-2.986968	-2.044536
C	3.203028	-4.009246	0.078074
N	3.880751	-2.388627	-2.091828
C	4.479794	-3.428346	0.004563
C	4.760421	-2.629517	-1.112238
H	2.890150	-4.610845	0.944542
H	5.228384	-3.578656	0.795177
H	5.743673	-2.134337	-1.210375
N	1.847352	-2.703308	-3.106015
H	0.878753	-3.026346	-3.101928
H	2.144588	-1.953579	-3.732977
C	0.697746	-5.732442	-1.760966
F	0.824577	-5.380702	-3.073437
F	1.623107	-6.697399	-1.524569
F	-0.524879	-6.295589	-1.612867
N	0.919160	1.295151	0.227996
C	2.141476	-0.023522	2.632800
H	1.072255	0.257426	2.621928
C	2.839768	0.226026	1.297302
C	2.281180	0.850860	0.171515
C	-0.166332	0.458356	0.094887
C	3.075001	1.111069	-0.952180
C	2.648055	1.855734	-2.217640
H	1.580006	2.142324	-2.210526
C	4.204024	-0.123663	1.307198
C	5.016388	0.154906	0.191879
C	4.437300	0.752796	-0.943570
C	0.513046	2.576552	0.558896
C	-0.875315	2.546388	0.655628
N	-1.266690	1.241767	0.382004
C	-2.629444	0.794887	0.409766
C	-3.089618	-0.096977	1.396209
C	-3.532393	1.306718	-0.536716
C	-4.469682	-0.381810	1.490424
C	-4.901849	0.984264	-0.462668
C	-5.386719	0.149770	0.561998
C	-2.247798	-0.909706	2.384490
H	-1.165810	-0.717156	2.281056
C	-3.166412	2.091470	-1.800774
H	-2.088829	2.328058	-1.867225
C	-3.648123	1.184720	-2.940921
C	-2.851705	0.604035	-3.933334
C	-5.033164	0.896997	-2.870986
C	-3.447374	-0.261162	-4.875914
H	-1.769777	0.809206	-3.967708
C	-5.625902	0.045663	-3.810743
C	-4.824592	-0.534213	-4.818675

H	-2.826690	-0.727082	-5.657258
H	-6.703451	-0.178401	-3.754084
H	-5.281572	-1.210058	-5.558495
C	-4.054121	3.334645	-1.808794
C	-3.622534	4.664942	-1.866951
C	-5.432646	3.028883	-1.712335
C	-4.578687	5.700566	-1.824813
H	-2.547393	4.896333	-1.923573
C	-6.381987	4.057335	-1.671916
C	-5.948127	5.398736	-1.727337
H	-4.247036	6.750056	-1.862079
H	-7.454746	3.818543	-1.593530
H	-6.688956	6.213194	-1.693599
C	-2.622334	-2.377527	2.100520
C	-4.009981	-2.626400	2.244034
C	-1.766386	-3.400748	1.676816
C	-4.536480	-3.897245	1.988244
C	-2.296720	-4.680071	1.400563
H	-0.684613	-3.258696	1.525022
C	-3.667886	-4.929306	1.568132
H	-5.616666	-4.083055	2.106671
H	-1.597082	-5.439329	1.017915
H	-4.074904	-5.931089	1.355932
C	-2.787262	-0.578943	3.773174
C	-2.047198	-0.096314	4.858793
C	-4.177494	-0.814289	3.893600
C	-2.706891	0.158641	6.079329
H	-0.962301	0.072882	4.759582
C	-4.831538	-0.559001	5.104424
C	-4.088788	-0.069040	6.199689
H	-2.134428	0.535342	6.941628
H	-5.913996	-0.742174	5.199448
H	-4.596471	0.132725	7.156216
C	3.018915	0.952165	-3.393987
C	2.157728	0.503630	-4.402294
C	4.382296	0.581396	-3.394966
C	2.672762	-0.315766	-5.430038
H	1.093014	0.787687	-4.389503
C	4.889403	-0.242115	-4.404832
C	4.029429	-0.686958	-5.427547
H	2.006927	-0.672240	-6.231827
H	5.945617	-0.551996	-4.388256
H	4.418581	-1.342730	-6.221690
C	3.591630	3.062533	-2.290455
C	3.220909	4.411131	-2.313184
C	4.959797	2.692012	-2.292694
C	4.225634	5.402601	-2.335690
H	2.156474	4.693916	-2.295025
C	5.955094	3.675454	-2.319091
C	5.582223	5.037529	-2.339227
H	3.940847	6.466487	-2.347436
H	7.018663	3.386346	-2.323074

H	6.361014	5.816312	-2.358356
C	2.365417	-1.499946	2.961724
C	3.732402	-1.856944	2.991055
C	1.374297	-2.449500	3.227760
C	4.109541	-3.161798	3.327131
C	1.750764	-3.774126	3.522734
H	0.311849	-2.166635	3.206760
C	3.110469	-4.122658	3.587319
H	5.175413	-3.437593	3.367194
H	0.973767	-4.534919	3.687732
H	3.399036	-5.158801	3.823230
C	2.959626	0.780566	3.649901
C	2.484173	1.795893	4.486059
C	4.329503	0.417059	3.665459
C	3.383987	2.451128	5.354649
H	1.419754	2.081145	4.461038
C	5.220287	1.066024	4.527025
C	4.740812	2.087753	5.375977
H	3.018416	3.251779	6.016874
H	6.284369	0.778696	4.542268
H	5.436396	2.602207	6.057856
O	6.365613	-0.138064	0.217560
C	7.166975	0.939148	0.713411
H	8.219915	0.596605	0.691721
H	7.062125	1.851329	0.080463
H	6.887523	1.208746	1.758748
O	-6.723828	-0.180652	0.623392
C	-7.040123	-1.392822	-0.075735
H	-8.112320	-1.600188	0.106729
H	-6.865795	-1.285613	-1.171259
H	-6.425833	-2.248604	0.285800
C	-1.383737	3.880326	0.970814
C	-2.618838	4.490055	1.164646
C	-0.192322	4.686682	1.042686
C	-2.647816	5.897519	1.423996
H	-3.556971	3.918888	1.101511
C	-0.200432	6.073651	1.294085
C	-1.489432	6.674668	1.489336
H	-3.627718	6.379118	1.564800
H	-1.561137	7.756209	1.687540
C	1.007265	3.928326	0.803072
C	2.235392	4.581354	0.824287
C	2.247352	5.989135	1.081529
H	3.175253	4.042310	0.633680
C	1.080192	6.722001	1.310224
H	3.218534	6.507853	1.091181
H	1.138470	7.805671	1.501187
C	-5.710678	1.525708	-1.646532
C	5.167777	1.172746	-2.220445
C	4.664809	-0.688549	2.654282
C	-4.805034	-1.368231	2.611429
H	-6.785679	1.284732	-1.564026

H	-5.892214	-1.542284	2.702437
H	5.732353	-0.972828	2.647189
H	6.230408	0.870979	-2.215196
4H c			
Ag4RX-OCF3ALKIPh--2Npost SCF Done: -4545.84763440 A.U.			
N	-0.786334	-1.420326	-0.583848
C	-2.170273	-1.719547	2.062536
H	-1.074608	-1.838154	1.977536
C	-2.827671	-1.290046	0.752021
C	-2.191386	-1.160278	-0.492663
C	0.177297	-0.432272	-0.488741
C	-2.934181	-0.790415	-1.619495
C	-2.402292	-0.607972	-3.040502
H	-1.322744	-0.829704	-3.123888
C	-4.220248	-1.107802	0.858863
C	-4.976264	-0.716962	-0.263500
C	-4.316050	-0.533681	-1.496392
C	-0.215071	-2.682513	-0.550356
C	1.156976	-2.491640	-0.430581
N	1.372804	-1.121951	-0.389183
C	2.630496	-0.570449	0.027513
C	2.798632	-0.181420	1.367317
C	3.729753	-0.555997	-0.840729
C	4.074352	0.186578	1.842146
C	4.997772	-0.161369	-0.367728
C	5.186934	0.202488	0.979104
C	1.705762	-0.066958	2.431993
H	0.706766	-0.333606	2.044742
C	3.714825	-0.883772	-2.335889
H	2.716478	-1.191833	-2.696834
C	4.230564	0.389013	-3.014517
C	3.555745	1.152614	-3.972867
C	5.501792	0.792645	-2.538339
C	4.150404	2.339456	-4.451840
H	2.562929	0.839212	-4.333126
C	6.087980	1.973849	-3.007243
C	5.404042	2.750555	-3.967321
H	3.621768	2.952866	-5.198025
H	7.071481	2.293270	-2.626253
H	5.856132	3.684020	-4.337411
C	4.785163	-1.958872	-2.530075
C	4.592459	-3.238581	-3.063260
C	6.058804	-1.559514	-2.057507
C	5.684349	-4.129327	-3.125787
H	3.594615	-3.548636	-3.411739
C	7.142433	-2.443479	-2.121364
C	6.949842	-3.733792	-2.659377
H	5.540846	-5.140323	-3.538405
H	8.131879	-2.134973	-1.747513
H	7.797676	-4.435155	-2.710448

C	1.783372	1.397337	2.891158
C	3.066145	1.751044	3.373409
C	0.783801	2.369431	2.755580
C	3.350740	3.075922	3.725288
C	1.078564	3.713028	3.071886
H	-0.217887	2.103588	2.378377
C	2.351196	4.057330	3.558726
H	4.353486	3.347885	4.094654
H	0.350281	4.504450	2.827066
H	2.575831	5.111716	3.784159
C	2.163654	-0.941436	3.597436
C	1.460316	-2.007536	4.172109
C	3.445084	-0.582103	4.080484
C	2.038619	-2.714167	5.247190
H	0.465290	-2.289510	3.792497
C	4.019039	-1.285336	5.145750
C	3.307826	-2.354207	5.731288
H	1.490038	-3.550871	5.707676
H	5.016953	-1.005714	5.520231
H	3.752503	-2.910349	6.571738
C	-2.742016	0.837554	-3.404188
C	-1.832005	1.834627	-3.774803
C	-4.120929	1.127761	-3.274223
C	-2.308615	3.136589	-4.027429
H	-0.755858	1.609362	-3.840769
C	-4.592454	2.423225	-3.520304
C	-3.678198	3.427632	-3.899899
H	-1.600825	3.937460	-4.286520
H	-5.662657	2.656250	-3.402751
H	-4.033535	4.455338	-4.068073
C	-3.279589	-1.500356	-3.924296
C	-2.841643	-2.524193	-4.771564
C	-4.660912	-1.208676	-3.807130
C	-3.791181	-3.261353	-5.510847
H	-1.766736	-2.751643	-4.856334
C	-5.602633	-1.939475	-4.540564
C	-5.161188	-2.971006	-5.396677
H	-3.454644	-4.068743	-6.180220
H	-6.676243	-1.706946	-4.450500
H	-5.895725	-3.550000	-5.978480
C	-2.584309	-0.695708	3.123682
C	-3.987783	-0.568521	3.245083
C	-1.732004	0.041414	3.953334
C	-4.539403	0.257924	4.228579
C	-2.288104	0.899467	4.924010
H	-0.640291	-0.048381	3.867593
C	-3.680869	0.994517	5.070932
H	-5.633307	0.342373	4.330150
H	-1.619875	1.492826	5.566575
H	-4.107689	1.662762	5.835108
C	-2.880562	-3.028623	2.425585
C	-2.278646	-4.276532	2.615794

C	-4.287276	-2.881773	2.517238
C	-3.091063	-5.394548	2.902863
H	-1.186386	-4.385243	2.522544
C	-5.091204	-3.990836	2.802532
C	-4.485703	-5.252489	2.995238
H	-2.626110	-6.382398	3.047033
H	-6.185261	-3.878304	2.874804
H	-5.113350	-6.129660	3.219181
O	-6.337941	-0.536517	-0.161610
C	-7.087907	-1.700645	-0.524162
H	-8.159622	-1.442871	-0.417948
H	-6.885050	-2.009888	-1.576368
H	-6.847415	-2.561441	0.143111
O	6.432125	0.577472	1.436778
C	6.658986	1.992790	1.403689
H	7.635895	2.173889	1.892489
H	6.695978	2.374452	0.357022
H	5.861413	2.551024	1.944774
C	1.839218	-3.778191	-0.316176
C	3.145042	-4.239251	-0.182403
C	0.763737	-4.733460	-0.369947
C	3.357951	-5.652469	-0.101393
H	3.997916	-3.545021	-0.146634
C	0.952444	-6.126855	-0.275417
C	2.309742	-6.574887	-0.140636
H	4.392104	-6.017223	-0.002723
H	2.521345	-7.653720	-0.066429
C	-0.526517	-4.109925	-0.511037
C	-1.661069	-4.913681	-0.540821
C	-1.491220	-6.330931	-0.437457
H	-2.668292	-4.479438	-0.625682
C	-0.236014	-6.930880	-0.312508
H	-2.390685	-6.965750	-0.450954
H	-0.153897	-8.027059	-0.236157
C	6.064283	-0.146542	-1.464645
C	-4.950776	-0.079435	-2.813766
C	-4.758208	-1.443287	2.251699
C	4.066858	0.590878	3.318926
H	7.054109	0.159981	-1.081659
H	5.074235	0.856526	3.686254
H	-5.853339	-1.315442	2.320538
H	-6.025537	0.151919	-2.705279
Ag	-0.154266	1.626864	-0.496957
C	0.061904	3.817020	-0.352494
C	1.166791	3.500773	-0.873294
C	2.575074	3.491099	-1.195497
H	2.850259	3.173685	-2.215073
C	3.576177	3.128007	-0.101925
C	3.445546	4.563807	-0.534032
H	2.911998	5.254886	0.139600
H	4.238483	5.011627	-1.154978
H	3.168960	2.840027	0.879868

H	4.445643	2.544991	-0.439608
O	-0.106988	5.670316	1.082108
C	-0.852132	4.895293	0.327620
C	-2.058782	4.224947	1.076243
C	-2.797785	3.071884	0.694634
C	-2.439100	4.843495	2.276259
N	-3.776814	2.530082	1.445918
C	-3.494098	4.323568	3.041960
C	-4.120470	3.154411	2.583517
H	-1.859777	5.734343	2.569628
H	-3.818022	4.803145	3.978495
H	-4.941861	2.693478	3.156788
N	-2.507877	2.359999	-0.495975
H	-2.463058	2.969701	-1.323753
H	-3.208816	1.621325	-0.636108
C	-1.455381	5.729876	-0.870256
F	-2.221682	4.968043	-1.748330
F	-2.272342	6.699296	-0.407777
F	-0.497921	6.311433	-1.618924

4H a+b→c'

Ag4RX-OCF3ALKIPhISOMER1--2O SCF Done: -4545.83891179 A.U.

Ag	0.023495	-1.690229	0.251531
N	-0.674485	1.192772	-0.643860
C	-3.033300	1.000070	-2.601618
H	-2.000275	0.878088	-2.975125
C	-3.102421	1.147263	-1.078815
C	-2.028701	1.117628	-0.178428
C	0.296864	0.239803	-0.412425
C	-2.276064	1.142858	1.205008
C	-1.233361	1.145864	2.324911
H	-0.196647	1.106377	1.946891
C	-4.419812	1.282561	-0.593368
C	-4.676176	1.333499	0.788671
C	-3.597929	1.219495	1.684543
C	-0.105034	2.364598	-1.121997
C	1.266257	2.145037	-1.199508
N	1.484775	0.845629	-0.765732
C	2.779285	0.277806	-0.536173
C	3.301190	-0.717676	-1.376015
C	3.539285	0.752398	0.546486
C	4.608212	-1.202966	-1.156827
C	4.834858	0.249006	0.770774
C	5.379220	-0.736070	-0.075620
C	2.591515	-1.408318	-2.542990
H	1.556763	-1.050611	-2.691357
C	3.095914	1.769718	1.604931
H	2.071326	2.151482	1.440544
C	3.255414	1.055838	2.953099
C	2.259337	0.852166	3.914697
C	4.567486	0.567076	3.166000

C	2.572649	0.143796	5.093718
H	1.239413	1.232878	3.760085
C	4.881471	-0.126989	4.339886
C	3.874346	-0.340259	5.306222
H	1.785237	-0.031797	5.842870
H	5.903548	-0.505681	4.502433
H	4.111445	-0.891550	6.229831
C	4.155314	2.872797	1.571200
C	3.931857	4.233393	1.333679
C	5.466881	2.380756	1.778133
C	5.033000	5.114489	1.303266
H	2.910655	4.604281	1.153163
C	6.558579	3.256250	1.749444
C	6.335775	4.629404	1.509863
H	4.867294	6.185878	1.109662
H	7.579894	2.874153	1.907182
H	7.190680	5.323529	1.483394
C	2.659224	-2.908762	-2.216787
C	3.986058	-3.373183	-2.037244
C	1.573169	-3.775435	-2.041453
C	4.234766	-4.708942	-1.704672
C	1.826516	-5.118596	-1.686010
H	0.531818	-3.427621	-2.147750
C	3.141667	-5.583697	-1.528156
H	5.269605	-5.063103	-1.567531
H	0.973914	-5.790887	-1.519871
H	3.321907	-6.634730	-1.252045
C	3.492920	-1.181819	-3.758549
C	3.123036	-0.589785	-4.971523
C	4.817635	-1.638217	-3.556915
C	4.085743	-0.455917	-5.993976
H	2.090108	-0.237116	-5.122965
C	5.773055	-1.504726	-4.570847
C	5.400793	-0.910039	-5.794952
H	3.803157	0.005618	-6.953374
H	6.803412	-1.861514	-4.411658
H	6.146392	-0.802618	-6.598679
C	-1.581759	-0.056980	3.206573
C	-0.737936	-1.124548	3.532012
C	-2.914378	-0.018350	3.680823
C	-1.238432	-2.172081	4.332570
H	0.299058	-1.151839	3.166460
C	-3.404556	-1.052125	4.483653
C	-2.559574	-2.134507	4.804830
H	-0.587300	-3.028606	4.566163
H	-4.446966	-1.037385	4.836186
H	-2.956977	-2.963623	5.409746
C	-1.525754	2.397664	3.156071
C	-0.637302	3.443459	3.430223
C	-2.853930	2.435310	3.649205
C	-1.074864	4.534242	4.212206
H	0.391902	3.418132	3.036547

C	-3.287501	3.517247	4.423348
C	-2.389460	4.569742	4.705952
H	-0.380924	5.360412	4.433418
H	-4.320008	3.543517	4.808259
H	-2.723679	5.423483	5.316565
C	-3.925267	-0.211775	-2.903155
C	-5.250478	-0.052099	-2.431756
C	-3.512586	-1.422499	-3.468874
C	-6.173881	-1.096768	-2.547436
C	-4.438686	-2.480140	-3.572429
H	-2.466245	-1.567064	-3.774667
C	-5.759177	-2.316266	-3.122445
H	-7.198373	-0.978189	-2.160302
H	-4.110539	-3.451225	-3.971625
H	-6.468668	-3.155587	-3.190199
C	-3.737721	2.233777	-3.166363
C	-3.191000	3.175610	-4.045787
C	-5.055753	2.392895	-2.671855
C	-3.964595	4.290447	-4.431596
H	-2.160036	3.054755	-4.414116
C	-5.823276	3.499533	-3.055475
C	-5.270696	4.452113	-3.938584
H	-3.538436	5.038919	-5.118055
H	-6.848734	3.622124	-2.670405
H	-5.869239	5.325033	-4.244122
O	-5.969531	1.451094	1.257434
C	-6.380424	2.807611	1.451249
H	-7.416649	2.780828	1.841383
H	-5.723200	3.331484	2.184129
H	-6.362000	3.380125	0.494142
O	6.636883	-1.245420	0.170305
C	6.604338	-2.429199	0.979083
H	7.655044	-2.735185	1.148375
H	6.107033	-2.235253	1.957577
H	6.053695	-3.254380	0.471686
C	1.949210	3.371916	-1.605084
C	3.257028	3.798624	-1.811682
C	0.875351	4.317772	-1.765119
C	3.473982	5.162767	-2.186159
H	4.110999	3.118478	-1.675290
C	1.068657	5.663062	-2.138414
C	2.427672	6.072979	-2.350974
H	4.510123	5.501311	-2.340753
H	2.642884	7.114507	-2.639287
C	-0.414925	3.747536	-1.477728
C	-1.545385	4.551791	-1.583586
C	-1.370524	5.916114	-1.979237
H	-2.551775	4.157085	-1.380653
C	-0.115546	6.467283	-2.247128
H	-2.267291	6.547734	-2.074373
H	-0.029153	7.525411	-2.542298
C	5.512833	0.862999	1.996959

C	-3.684206	1.222585	3.211418
C	-5.470849	1.281086	-1.706458
C	5.032171	-2.263236	-2.175348
H	6.536141	0.474365	2.145941
H	6.069137	-2.608698	-2.013503
H	-6.496797	1.394702	-1.312624
H	-4.729127	1.244503	3.568971
C	-0.500686	-3.807518	0.849396
C	0.615508	-3.596765	1.403060
C	1.842185	-3.648117	2.182214
H	1.791590	-4.367604	3.019475
C	2.710426	-2.413002	2.425667
C	3.207451	-3.501355	1.513643
H	3.205047	-3.305410	0.431642
H	4.010405	-4.172064	1.858856
H	2.385382	-1.471341	1.954515
H	3.156050	-2.285466	3.424180
O	-1.823848	-2.665350	-1.254149
C	-2.178152	-3.521793	-0.392653
C	-3.262545	-3.164326	0.610931
C	-3.524829	-3.769357	1.890886
C	-4.141391	-2.149621	0.199043
N	-4.586360	-3.408289	2.655108
C	-5.251303	-1.794179	0.979406
C	-5.424135	-2.470698	2.196728
H	-3.936743	-1.660307	-0.762640
H	-5.967172	-1.029163	0.649462
H	-6.285008	-2.230279	2.849704
N	-2.721311	-4.738620	2.426850
H	-1.771348	-4.806973	2.036572
H	-2.842304	-4.875515	3.431886
C	-2.266382	-4.976737	-0.956352
F	-1.263094	-5.224084	-1.835847
F	-2.254530	-5.967785	-0.038442
F	-3.435805	-5.085783	-1.650229

4H c’

Ag4RX-OCF3ALKIPhISOMER1--2minISOMER SCF Done: -4545.86526685 A.U.

Ag	1.107437	-0.744604	0.183504
N	-0.086546	1.958554	0.551742
C	2.031480	2.291861	2.665729
H	0.979109	2.111143	2.953356
C	2.200546	2.591103	1.176212
C	1.211810	2.453401	0.193805
C	-0.390941	0.617184	0.406211
C	1.543669	2.606636	-1.161322
C	0.625178	2.359359	-2.357749
H	-0.398503	2.067504	-2.059181
C	3.536369	2.832024	0.797491
C	3.883497	2.991701	-0.558501

C	2.865495	2.921712	-1.531746
C	-1.175657	2.668329	1.024256
C	-2.210165	1.747775	1.170593
N	-1.720151	0.510859	0.773651
C	-2.576601	-0.615027	0.527721
C	-2.493806	-1.808702	1.265557
C	-3.562229	-0.475298	-0.471477
C	-3.447723	-2.828173	1.047039
C	-4.475278	-1.519338	-0.712042
C	-4.432296	-2.701119	0.050378
C	-1.472413	-2.178799	2.347215
H	-0.697040	-1.404784	2.491400
C	-3.738342	0.703201	-1.442893
H	-3.019252	1.525024	-1.273121
C	-3.617564	0.096866	-2.844716
C	-2.711444	0.477801	-3.839537
C	-4.536973	-0.955899	-3.073044
C	-2.700307	-0.220444	-5.065886
H	-2.006016	1.305618	-3.667411
C	-4.533988	-1.642208	-4.292573
C	-3.602899	-1.274103	-5.289140
H	-1.971697	0.059553	-5.842221
H	-5.246153	-2.464770	-4.467669
H	-3.586624	-1.816299	-6.247694
C	-5.195297	1.153106	-1.307836
C	-5.645946	2.442926	-1.004885
C	-6.116800	0.100907	-1.524824
C	-7.032319	2.679535	-0.902315
H	-4.924139	3.254986	-0.824010
C	-7.492774	0.335477	-1.420784
C	-7.948499	1.633301	-1.104959
H	-7.394151	3.689268	-0.653056
H	-8.209080	-0.485499	-1.584718
H	-9.029753	1.825721	-1.019818
C	-0.883027	-3.536477	1.922050
C	-1.870989	-4.535446	1.745465
C	0.466555	-3.831050	1.690079
C	-1.515758	-5.829353	1.346079
C	0.822985	-5.130185	1.267201
H	1.255135	-3.066446	1.776038
C	-0.156766	-6.123073	1.102835
H	-2.291955	-6.600876	1.214011
H	1.881045	-5.347964	1.055133
H	0.134965	-7.132635	0.772969
C	-2.304348	-2.444079	3.604090
C	-2.166740	-1.809835	4.843837
C	-3.295460	-3.436198	3.410140
C	-3.027170	-2.172131	5.901649
H	-1.392105	-1.039448	4.988959
C	-4.149819	-3.794617	4.458862
C	-4.011638	-3.156556	5.709996
H	-2.924968	-1.680357	6.881957

H	-4.921237	-4.566572	4.306162
H	-4.680081	-3.433538	6.540560
C	1.328457	1.283532	-3.198881
C	0.816835	0.032080	-3.556652
C	2.631464	1.664124	-3.600561
C	1.604353	-0.827900	-4.350066
H	-0.177862	-0.289382	-3.216348
C	3.409809	0.811313	-4.388591
C	2.886462	-0.441388	-4.768842
H	1.208613	-1.815800	-4.626982
H	4.425792	1.110426	-4.692690
H	3.497404	-1.127647	-5.375159
C	0.671352	3.649595	-3.176604
C	-0.421681	4.436211	-3.557325
C	1.988855	4.020662	-3.541625
C	-0.197717	5.605297	-4.314960
H	-1.444019	4.142498	-3.267481
C	2.209844	5.181208	-4.291710
C	1.108558	5.974581	-4.678399
H	-1.051437	6.229958	-4.622138
H	3.234586	5.469741	-4.575981
H	1.275775	6.889025	-5.269382
C	2.932139	1.074100	2.902272
C	4.276443	1.304980	2.525931
C	2.527579	-0.180154	3.371642
C	5.220727	0.277860	2.626267
C	3.480652	-1.213416	3.473122
H	1.475444	-0.359179	3.646024
C	4.816148	-0.984808	3.105888
H	6.264930	0.451172	2.320509
H	3.166655	-2.208867	3.823215
H	5.550258	-1.802894	3.167578
C	2.662108	3.469017	3.407669
C	2.036217	4.287318	4.355289
C	4.009165	3.698061	3.035849
C	2.766230	5.341141	4.944113
H	0.985714	4.106544	4.635239
C	4.732294	4.744809	3.619992
C	4.104873	5.566740	4.579761
H	2.283814	5.988673	5.693431
H	5.779796	4.923329	3.328443
H	4.668829	6.391187	5.044239
O	5.200986	3.146652	-0.925842
C	5.832848	1.893527	-1.248909
H	5.792692	1.186107	-0.391197
H	5.337577	1.398657	-2.113565
H	6.886621	2.121934	-1.499715
O	-5.336420	-3.714772	-0.189500
C	-4.826639	-4.724424	-1.069662
H	-5.628802	-5.476956	-1.198027
H	-4.556576	-4.293760	-2.061739
H	-3.919684	-5.216001	-0.648588

C	-3.416736	2.415702	1.650341
C	-4.722609	2.065029	1.974733
C	-3.016927	3.793415	1.763207
C	-5.614772	3.097291	2.406308
H	-5.075824	1.027269	1.882571
C	-3.880057	4.823877	2.187190
C	-5.223065	4.433002	2.511803
H	-6.652114	2.820612	2.649274
H	-5.945598	5.195320	2.844771
C	-1.641143	4.004304	1.393644
C	-1.117600	5.291809	1.463075
C	-1.979069	6.351970	1.891462
H	-0.069537	5.499367	1.198595
C	-3.314865	6.141721	2.241997
H	-1.565713	7.371324	1.944185
H	-3.940383	6.989525	2.564567
C	-5.438201	-1.230689	-1.864242
C	3.049740	3.031596	-3.048014
C	4.511660	2.719827	1.972057
C	-3.289624	-4.019482	1.992856
H	-6.154453	-2.056096	-2.024848
H	-4.073293	-4.782820	1.837843
H	5.556991	2.905094	1.666191
H	4.079343	3.322585	-3.323687
C	2.105207	-3.021468	-1.562855
C	1.052023	-3.276104	-2.142923
C	-0.234832	-3.497511	-2.747081
H	-0.219590	-3.807870	-3.807833
C	-1.421116	-2.624753	-2.326293
C	-1.376041	-4.066281	-1.901647
H	-1.148792	-4.289105	-0.848257
H	-2.047122	-4.786879	-2.395490
H	-1.223384	-1.860514	-1.557169
H	-2.122682	-2.316335	-3.115288
O	2.731185	-2.009569	0.468835
C	3.222419	-2.670895	-0.641951
C	3.997751	-3.948596	-0.226136
C	4.350156	-5.026479	-1.104154
C	4.325945	-4.087759	1.126634
N	4.935504	-6.161208	-0.647406
C	4.967213	-5.248231	1.594653
C	5.235439	-6.256602	0.655031
H	4.030762	-3.265994	1.792922
H	5.227667	-5.375333	2.656320
H	5.710802	-7.204522	0.971974
N	4.137033	-4.988940	-2.462117
H	3.467492	-4.314008	-2.836615
H	4.206964	-5.895592	-2.927239
C	4.203660	-1.676975	-1.363645
F	5.270093	-1.395594	-0.575917
F	4.681674	-2.146153	-2.548182
F	3.586366	-0.491438	-1.630763

Cu geometries

4A cat

Cu1RX-Clbb SCF Done: -3578.78595986 A.U.

C	-2.016129	6.760484	-1.619313
H	-1.325126	7.185466	-0.855557
H	-2.723579	7.547615	-1.941878
H	-1.417676	6.426729	-2.497248
Cu	0.024249	0.006150	-1.844608
Cl	0.189352	0.041735	-3.963774
O	-2.809438	5.705687	-1.087775
O	2.716887	-5.602654	-1.218315
N	-0.503057	1.000548	0.856133
C	1.049124	-2.139389	0.349386
C	-1.086864	2.218469	0.364890
C	-2.183280	4.585627	-0.634970
N	0.484871	-0.921959	0.860549
C	-0.223356	3.222721	-0.127599
C	-0.279977	0.670829	2.195435
H	-0.557020	1.347692	3.008302
C	0.332151	-0.557402	2.197941
H	0.667174	-1.204088	3.013629
C	-3.090689	0.540605	-1.864773
H	-2.304998	1.306712	-1.952747
C	3.373717	-1.131602	0.673752
H	2.716914	-0.266331	0.907130
C	-2.494770	2.347315	0.302884
C	-4.542881	1.509171	1.550063
C	-3.027533	3.549764	-0.180494
H	-4.114275	3.699585	-0.244909
C	-0.024598	0.029697	0.015214
C	-0.781497	4.411656	-0.624734
H	-0.102798	5.182018	-1.014174
C	-2.126820	-3.686722	-1.088953
C	2.969369	-3.469078	-0.266938
H	4.054763	-3.612189	-0.368401
C	-6.612210	2.223111	3.348846
H	-7.418444	2.498713	4.046994
C	-4.819534	-0.590256	-0.598559
H	-5.405124	-0.713112	0.326381
C	-2.957312	-2.278972	1.804670
H	-3.498648	-1.686841	1.053468
C	1.286590	2.989850	-0.099354
H	1.428241	1.921887	-0.370694
C	-3.362594	-0.279524	-2.974034
H	-2.746273	-0.162091	-3.878715
C	-1.827535	-3.016452	1.394761
C	-1.340466	-2.893901	-0.049746
H	-1.509390	-1.831213	-0.339195
C	-3.391597	1.149075	0.617590
H	-2.764167	0.426903	1.183809

C	-4.371752	1.360595	2.942800
H	-3.420976	0.952506	3.324987
C	4.217663	-0.697859	-0.526860
C	-3.800889	0.389741	-0.655456
C	0.708261	-4.313078	-0.665592
H	0.017249	-5.075642	-1.048088
C	5.515733	-1.195287	-0.765578
H	5.980083	-1.879318	-0.037412
C	4.386990	-2.564090	4.110691
H	4.055749	-3.359194	4.797841
C	-3.085880	-4.658133	-0.749470
H	-3.288430	-4.872270	0.311672
C	0.167005	-3.129826	-0.138739
C	6.225252	-0.811353	-1.917099
H	7.239603	-1.206091	-2.088561
C	-6.796373	2.369067	1.962119
H	-7.749729	2.758169	1.570283
C	-3.792642	-5.343167	-1.756646
H	-4.545584	-6.097902	-1.477809
C	-5.771328	2.011352	1.070024
H	-5.931920	2.096531	-0.016512
C	4.141599	-1.393770	1.970718
C	5.453515	-1.722923	4.468308
H	5.964738	-1.852278	5.435358
C	-1.884051	-3.406205	-2.453268
H	-1.151307	-2.625799	-2.726209
C	2.451605	-2.278404	0.261557
C	2.021034	3.768047	-1.185389
C	-2.578221	-4.097643	-3.455935
H	-2.381201	-3.861510	-4.513766
C	-1.160356	-3.810525	2.350006
H	-0.278360	-4.396476	2.046606
C	-5.097359	-1.408026	-1.706613
H	-5.880610	-2.178566	-1.632810
C	1.911451	3.316133	-2.520119
H	1.323991	2.407950	-2.748831
C	3.296134	5.171338	-3.280883
H	3.797654	5.716287	-4.096628
C	-2.710659	-3.082534	4.088211
H	-3.045213	-3.100258	5.137712
C	3.739795	-2.401507	2.872459
H	2.910341	-3.072535	2.598965
C	5.215704	-0.553857	2.342366
H	5.547277	0.234971	1.649064
C	3.645270	0.178508	-1.472830
H	2.615070	0.542183	-1.320738
C	-1.599073	-3.843963	3.686572
H	-1.062983	-4.466874	4.420714
C	2.545370	4.012920	-3.559931
H	2.453089	3.643428	-4.593702
C	1.861796	3.134464	1.308603
C	5.866449	-0.716648	3.575860

H	6.703982	-0.051667	3.841661
C	-3.538431	-5.068420	-3.110753
H	-4.090701	-5.606138	-3.898077
C	2.917679	3.268175	3.932605
H	3.322555	3.308279	4.956279
C	2.772389	4.926944	-0.911703
H	2.867253	5.276513	0.128770
C	3.407600	5.625749	-1.955689
H	3.998046	6.528146	-1.729099
C	-5.395993	1.713935	3.837623
H	-5.245396	1.586273	4.921622
C	2.109513	-4.490313	-0.722430
C	1.225779	3.908754	2.300161
H	0.300214	4.451496	2.051375
C	-3.395469	-2.302790	3.137456
H	-4.270492	-1.701084	3.431934
C	3.568374	2.504903	2.945487
H	4.477645	1.938402	3.196488
C	-4.365822	-1.258314	-2.897432
H	-4.566757	-1.917116	-3.755357
C	4.349671	0.566093	-2.622494
H	3.874857	1.253912	-3.338489
C	3.048665	2.445811	1.642932
H	3.566295	1.858768	0.866175
C	5.646959	0.072363	-2.846166
H	6.206572	0.373937	-3.745764
C	1.750704	3.977909	3.603577
H	1.238815	4.585997	4.366817
C	1.904693	-6.633194	-1.768073
H	1.229810	-7.083067	-1.004019
H	2.599406	-7.411685	-2.136236
H	1.287307	-6.264270	-2.618381
4A a			
Cu1RX-ALKI SCF Done: -3311.94679221 A.U.			
Cu	-0.004624	0.275076	1.553237
N	-0.108821	0.941381	-1.255481
C	-2.899048	0.426924	-1.478564
H	-2.063517	-0.295542	-1.377582
C	-2.388435	1.774129	-0.974322
C	-0.994413	2.019758	-0.903633
C	0.304797	0.053766	-0.292654
C	-0.475985	3.201092	-0.339030
C	1.036972	3.376266	-0.186856
H	1.464993	2.365706	-0.003663
C	-3.264914	2.770701	-0.529373
C	-2.767731	3.992056	-0.023145
C	-1.376322	4.199327	0.077799
C	0.203350	0.495344	-2.537484
C	0.820639	-0.720712	-2.381160
N	0.872657	-0.970375	-1.005032

C	1.393271	-2.161418	-0.387366
C	0.494486	-3.121711	0.154086
C	2.794274	-2.316290	-0.282221
C	1.036176	-4.273059	0.740193
C	3.306083	-3.482696	0.314648
C	2.431184	-4.469088	0.812538
C	-1.024597	-2.921503	0.110121
H	-1.198457	-1.825774	0.203651
C	3.732308	-1.180219	-0.701708
H	3.333809	-0.757420	-1.650626
C	3.730622	-0.021685	0.307067
C	3.083471	-0.089444	1.556921
C	4.399172	1.173431	-0.041279
C	3.088088	1.016078	2.430470
H	2.544349	-1.003006	1.852592
C	4.412640	2.272712	0.830393
C	3.754967	2.197552	2.071790
H	2.525828	0.954050	3.374947
H	3.734472	3.071611	2.740130
C	5.139075	-1.690604	-1.016207
C	5.403288	-2.277291	-2.271880
C	6.172625	-1.651006	-0.057304
C	6.667632	-2.813560	-2.565521
H	4.599878	-2.319313	-3.026453
C	7.438320	-2.191316	-0.345939
C	7.690470	-2.774263	-1.600169
H	6.857209	-3.262504	-3.553693
H	8.234693	-2.151795	0.414461
H	8.683865	-3.192175	-1.828195
C	-1.706724	-3.535828	1.336493
C	-2.327323	-4.801505	1.303104
C	-1.679268	-2.825593	2.554501
C	-2.918901	-5.337804	2.461462
C	-2.267778	-3.358537	3.711498
H	-1.199588	-1.833385	2.607545
C	-2.894151	-4.617668	3.668946
H	-3.404517	-6.326018	2.417309
H	-2.235635	-2.774137	4.645113
H	-3.362003	-5.037859	4.573806
C	-1.663420	-3.332149	-1.218915
C	-3.067545	-3.228844	-1.357589
C	-0.913033	-3.790635	-2.320732
C	-3.699110	-3.573313	-2.560764
H	-3.669270	-2.880571	-0.505088
C	-1.546042	-4.135058	-3.530963
C	-2.941272	-4.029842	-3.655270
H	-4.792080	-3.468518	-2.644237
H	-0.940684	-4.494191	-4.378807
H	-3.437085	-4.294508	-4.602366
C	1.417120	4.179639	1.057049
C	0.874750	3.775171	2.299506
C	2.360115	5.224210	1.039038

C	1.263465	4.403228	3.490760
H	0.165541	2.930183	2.342515
C	2.752764	5.855689	2.235452
C	2.205959	5.449938	3.464484
H	0.837456	4.054808	4.445035
H	3.497134	6.667557	2.202673
H	2.516988	5.942235	4.399814
C	1.610461	3.862690	-1.515263
C	2.459216	3.026674	-2.268079
C	1.255688	5.121286	-2.048371
C	2.956965	3.440199	-3.516438
H	2.726045	2.039012	-1.862595
C	1.755627	5.540927	-3.291374
C	2.609494	4.700912	-4.030900
H	3.619488	2.771564	-4.089418
H	1.472350	6.528188	-3.690187
H	2.998494	5.028434	-5.008149
C	-3.983849	-0.160266	-0.572650
C	-3.652506	-0.388078	0.783377
C	-5.234479	-0.591305	-1.051709
C	-4.535693	-1.072849	1.629653
C	-6.129064	-1.264517	-0.197425
H	-5.496469	-0.430566	-2.108651
C	-5.778097	-1.519471	1.139344
H	-4.236163	-1.279046	2.668594
H	-7.101229	-1.606326	-0.587756
H	-6.468343	-2.067805	1.799616
C	-3.230395	0.445177	-2.968810
C	-2.724512	-0.580366	-3.794701
C	-4.040641	1.443068	-3.550169
C	-3.026607	-0.616431	-5.165819
H	-2.087097	-1.361125	-3.352340
C	-4.344297	1.410245	-4.922091
C	-3.839023	0.379594	-5.735556
H	-2.624695	-1.432684	-5.787152
H	-4.978318	2.198177	-5.359349
H	-4.076605	0.355721	-6.811109
O	-3.706518	4.894232	0.375834
C	-3.270425	6.110687	0.968919
H	-2.677746	5.931749	1.894962
H	-2.658663	6.720512	0.264707
H	-4.186120	6.674658	1.229407
O	2.839632	-5.629606	1.394269
C	4.235431	-5.870213	1.521957
H	4.336477	-6.858337	2.009351
H	4.744349	-5.899110	0.531263
H	4.732518	-5.100792	2.156015
C	-0.540556	0.465135	3.305863
C	-1.012004	0.507297	4.456626
C	-1.612021	0.531964	5.769480
H	-0.910777	0.410194	6.616647
C	-3.001950	-0.076848	5.985297

C	-2.826229	1.420082	6.052597
H	-3.202500	2.023814	5.211393
H	-2.879024	1.913711	7.037121
H	-3.496639	-0.499616	5.095985
H	-3.177867	-0.633761	6.920523
H	1.200768	-1.437991	-3.113839
H	-0.066630	1.065044	-3.430591
H	-2.676888	-0.046825	1.176115
H	-4.434489	2.258357	-2.924200
H	-2.358744	-5.367967	0.358974
H	0.179391	-3.895389	-2.227261
H	4.919440	3.203521	0.531544
H	4.921761	1.235469	-1.010124
H	5.977500	-1.186099	0.921810
H	0.575416	5.775567	-1.480227
H	2.810808	5.535434	0.084481
H	-4.351074	2.597388	-0.514653
H	-0.968576	5.109688	0.536528
H	0.378511	-5.038594	1.175828
H	4.394632	-3.594651	0.394582
4A a+b			
Cu1RX-OCF3ALKIPh--2Npre SCF Done: -4065.47051046 A.U.			
Cu	-0.587839	0.266543	-0.926369
N	1.637194	-0.182376	1.129718
C	1.384629	-3.018001	1.180258
H	0.681501	-2.234485	1.526748
C	2.399634	-2.333723	0.262605
C	2.544645	-0.926533	0.304850
C	0.406973	0.235653	0.682419
C	3.518955	-0.244594	-0.450447
C	3.593522	1.279049	-0.346400
H	2.540082	1.634192	-0.267645
C	3.246220	-3.058454	-0.586631
C	4.261786	-2.402062	-1.313243
C	4.408556	-1.002704	-1.231868
C	1.791580	0.027135	2.501301
C	0.620374	0.587713	2.940866
N	-0.203357	0.703694	1.820779
C	-1.529491	1.262630	1.799768
C	-2.658384	0.396050	1.820460
C	-1.659735	2.659633	1.663199
C	-3.925200	0.986381	1.808755
C	-2.950738	3.222894	1.648644
C	-4.081265	2.387315	1.730644
C	-2.473873	-1.123369	1.733590
H	-1.633813	-1.270774	1.016373
C	-0.428946	3.525275	1.370846
H	0.444956	3.019449	1.832539
C	-0.132047	3.614747	-0.134379
C	-1.123887	3.394354	-1.110287

C	1.168394	3.961802	-0.567807
C	-0.832033	3.534186	-2.479822
H	-2.130366	3.073705	-0.811432
C	1.470735	4.079889	-1.934179
C	0.464748	3.873404	-2.897599
H	-1.626545	3.333736	-3.215097
H	0.701167	3.965262	-3.969730
C	-0.526473	4.897765	2.038135
C	-0.247350	5.012393	3.417125
C	-0.932563	6.048017	1.331857
C	-0.373630	6.244523	4.077494
H	0.067493	4.115936	3.977692
C	-1.064507	7.283532	1.991751
C	-0.785839	7.386397	3.365299
H	-0.147272	6.316457	5.153455
H	-1.382172	8.173393	1.424923
H	-0.883967	8.355028	3.880619
C	-3.667770	-1.843777	1.105363
C	-4.795994	-2.205558	1.872995
C	-3.646763	-2.166568	-0.265024
C	-5.886568	-2.859214	1.274029
C	-4.725357	-2.838771	-0.862967
H	-2.790612	-1.856884	-0.879096
C	-5.852384	-3.180845	-0.096344
H	-6.764533	-3.128316	1.883116
H	-4.671000	-3.074163	-1.936972
H	-6.705798	-3.698032	-0.563435
C	-2.052093	-1.810552	3.032961
C	-1.777933	-3.197878	2.988470
C	-1.958022	-1.143266	4.268950
C	-1.425726	-3.897102	4.150228
H	-1.850379	-3.730092	2.026590
C	-1.598174	-1.844123	5.437541
C	-1.336962	-3.223053	5.383506
H	-1.200736	-4.973097	4.088584
H	-1.534767	-1.306784	6.397712
H	-1.055898	-3.771942	6.296010
C	4.146503	2.002971	-1.576743
C	3.787762	1.576434	-2.877286
C	4.902838	3.185860	-1.442521
C	4.155354	2.329611	-4.004787
H	3.229491	0.632084	-3.016698
C	5.272203	3.938101	-2.572745
C	4.893540	3.518363	-3.859474
H	3.858112	1.979815	-5.006732
H	5.856631	4.862934	-2.442469
H	5.177894	4.110147	-4.743976
C	4.288891	1.669538	0.959259
C	3.645803	2.494451	1.903028
C	5.588715	1.202222	1.247651
C	4.283082	2.854940	3.103076
H	2.621265	2.841457	1.701755

C	6.230303	1.557631	2.444906
C	5.579447	2.387490	3.377643
H	3.758813	3.499317	3.826535
H	7.244368	1.181648	2.654699
H	6.081785	2.664609	4.317976
C	0.473577	-4.009443	0.455018
C	-0.315374	-3.512713	-0.606646
C	0.282270	-5.338816	0.877954
C	-1.292814	-4.313310	-1.214749
C	-0.687285	-6.150239	0.257995
H	0.873410	-5.735104	1.717423
C	-1.482233	-5.638750	-0.781395
H	-1.921102	-3.883967	-2.009772
H	-0.831548	-7.186014	0.605403
H	-2.258207	-6.266065	-1.247717
C	2.067911	-3.532666	2.448049
C	1.652855	-3.041234	3.702919
C	3.111181	-4.484023	2.411549
C	2.257328	-3.487055	4.890609
H	0.838239	-2.303331	3.748791
C	3.715812	-4.934605	3.596693
C	3.291521	-4.436645	4.842611
H	1.905283	-3.092081	5.856837
H	4.526302	-5.679304	3.546487
H	3.767569	-4.788977	5.771608
O	5.035233	-3.202312	-2.107185
C	6.066194	-2.596462	-2.877932
H	5.659544	-1.855732	-3.604387
H	6.821052	-2.090098	-2.233968
H	6.559245	-3.415620	-3.434752
O	-5.369200	2.833510	1.705927
C	-5.598530	4.225905	1.543490
H	-6.696647	4.361652	1.533709
H	-5.167290	4.820134	2.381764
H	-5.177998	4.605229	0.583780
C	-2.298899	0.421639	-1.685633
C	-3.480259	0.808021	-1.794121
C	-4.859523	1.201678	-1.900859
H	-5.030873	2.269371	-2.136663
C	-5.930020	0.548015	-1.017853
C	-5.890323	0.229329	-2.485694
H	-5.496476	-0.752480	-2.790861
H	-6.658369	0.653294	-3.153157
H	-5.588121	-0.231859	-0.321243
H	-6.720587	1.207399	-0.625305
O	-2.847397	-2.087360	-3.526055
C	-1.994131	-1.249456	-3.818566
C	-0.527681	-1.569698	-3.743634
C	0.582955	-0.706146	-3.440860
C	-0.215932	-2.923432	-4.004604
N	1.860211	-1.153161	-3.440253
C	1.104704	-3.377971	-3.996554

C	2.109802	-2.437718	-3.715925
H	-1.048081	-3.606952	-4.228167
H	1.352771	-4.428257	-4.205653
H	3.169887	-2.742402	-3.709368
N	0.429835	0.593708	-2.989602
H	-0.386445	1.120710	-3.309352
H	1.294048	1.141584	-2.937466
C	-2.444259	-0.104486	-4.784385
F	-1.776439	1.084977	-4.659057
F	-2.178351	-0.545893	-6.049753
F	-3.752882	0.147401	-4.706214
H	0.294822	0.900140	3.936362
H	2.707244	-0.255913	3.026746
H	-0.181014	-2.466772	-0.938174
H	3.456884	-4.879893	1.444867
H	-4.810844	-1.981941	2.952341
H	-2.185974	-0.066250	4.319458
H	2.495369	4.330928	-2.247549
H	1.959022	4.156563	0.174488
H	-1.138811	5.971299	0.252541
H	6.096460	0.545563	0.523401
H	5.192477	3.533928	-0.439442
H	3.124348	-4.144107	-0.711673
H	5.171530	-0.478368	-1.820963
H	-4.831858	0.367848	1.808344
H	-3.046808	4.311846	1.544789
4A a+b→c			
Cu1RX-OCF3ALKIPhISOMER1--2delAg SCF Done: -4065.46791546 A.U.			
Cu	-0.446588	0.132746	-1.053725
N	1.530188	0.063631	1.215643
C	1.720693	-2.781426	1.313090
H	0.892446	-2.107942	1.610895
C	2.681551	-1.953619	0.456873
C	2.597879	-0.540721	0.470789
C	0.283975	0.294076	0.680906
C	3.510071	0.278528	-0.224890
C	3.330154	1.795766	-0.160140
H	2.230564	1.976541	-0.183823
C	3.695400	-2.544178	-0.308934
C	4.649570	-1.744628	-0.971630
C	4.570262	-0.338664	-0.910443
C	1.563306	0.316559	2.587273
C	0.299849	0.713231	2.938132
N	-0.456907	0.692048	1.764495
C	-1.847645	1.034258	1.667311
C	-2.822873	-0.000796	1.661083
C	-2.192549	2.394018	1.527000
C	-4.167502	0.383325	1.625480
C	-3.555348	2.746790	1.479499
C	-4.540327	1.742431	1.543947

C	-2.393560	-1.472621	1.600959
H	-1.494747	-1.487648	0.942605
C	-1.099708	3.437642	1.270634
H	-0.187582	3.091584	1.801320
C	-0.728959	3.519823	-0.218118
C	-1.600859	3.079212	-1.232534
C	0.512083	4.079486	-0.599338
C	-1.257037	3.210231	-2.590685
H	-2.552471	2.601762	-0.962484
C	0.871960	4.190644	-1.952118
C	-0.020196	3.765954	-2.955256
H	-1.947506	2.825732	-3.357056
H	0.261918	3.852051	-4.016655
C	-1.453037	4.799555	1.868424
C	-1.267245	5.014614	3.250996
C	-2.007681	5.836003	1.090142
C	-1.629259	6.233938	3.844944
H	-0.837139	4.206941	3.867027
C	-2.376112	7.057251	1.683318
C	-2.188674	7.260453	3.061219
H	-1.472942	6.386308	4.924912
H	-2.807410	7.857739	1.061088
H	-2.471998	8.218884	3.524370
C	-3.416201	-2.365918	0.898499
C	-4.552914	-2.864157	1.572420
C	-3.231870	-2.696956	-0.456848
C	-5.499626	-3.645701	0.888125
C	-4.170517	-3.484700	-1.144085
H	-2.359281	-2.309639	-0.998111
C	-5.312802	-3.954317	-0.473775
H	-6.386404	-4.022024	1.423219
H	-3.988841	-3.697814	-2.208743
H	-6.058607	-4.565133	-1.007502
C	-1.961649	-2.080296	2.936358
C	-1.482662	-3.411145	2.932979
C	-2.044450	-1.393416	4.162448
C	-1.101424	-4.037601	4.126687
H	-1.415863	-3.956777	1.978458
C	-1.655834	-2.020543	5.363182
C	-1.188495	-3.344985	5.349830
H	-0.717016	-5.069105	4.099040
H	-1.730737	-1.470539	6.315281
H	-0.883740	-3.836179	6.287253
C	3.862488	2.590899	-1.356727
C	3.738793	2.087307	-2.673569
C	4.348660	3.904403	-1.184702
C	4.063713	2.889563	-3.780843
H	3.398439	1.048672	-2.843609
C	4.677119	4.704746	-2.293743
C	4.528153	4.204559	-3.598828
H	3.950582	2.476420	-4.796244
H	5.047267	5.729983	-2.133256

H	4.779160	4.832906	-4.468036
C	3.831015	2.315804	1.189180
C	2.983546	3.052620	2.039712
C	5.151568	2.057115	1.613243
C	3.439221	3.530614	3.280418
H	1.943411	3.233342	1.730945
C	5.612218	2.528785	2.853018
C	4.757391	3.269651	3.691335
H	2.756723	4.103102	3.928554
H	6.645043	2.313790	3.170410
H	5.118697	3.637904	4.664591
C	1.009472	-3.894807	0.541630
C	0.275591	-3.529693	-0.608637
C	0.927834	-5.222922	1.003369
C	-0.545473	-4.455552	-1.268630
C	0.117049	-6.160071	0.334501
H	1.475115	-5.520485	1.910658
C	-0.627752	-5.777715	-0.794012
H	-1.140583	-4.119209	-2.133029
H	0.056634	-7.193261	0.713158
H	-1.281999	-6.504994	-1.300289
C	2.388741	-3.193528	2.625973
C	1.848353	-2.740671	3.847113
C	3.538833	-4.012310	2.664217
C	2.436592	-3.092629	5.073977
H	0.945541	-2.111589	3.836169
C	4.128057	-4.369022	3.888285
C	3.580466	-3.907520	5.099834
H	1.986132	-2.731919	6.012339
H	5.022630	-5.012204	3.895941
H	4.044311	-4.186008	6.059577
O	5.598726	-2.421543	-1.684007
C	6.564687	-1.670465	-2.409471
H	6.089527	-1.016360	-3.176143
H	7.190304	-1.039940	-1.736963
H	7.214347	-2.408840	-2.916357
O	-5.883072	1.980321	1.489501
C	-6.326728	3.324447	1.357775
H	-7.432333	3.286525	1.338530
H	-5.999738	3.956105	2.215124
H	-5.963365	3.789874	0.412764
C	-1.967393	-0.253360	-2.185523
C	-3.135888	0.200583	-2.088741
C	-4.495076	0.641298	-2.050307
H	-4.649432	1.733535	-2.141705
C	-5.567420	-0.113912	-1.252118
C	-5.560834	-0.233122	-2.743242
H	-5.183910	-1.169036	-3.184674
H	-6.317031	0.299889	-3.341878
H	-5.228890	-0.985435	-0.674294
H	-6.322619	0.512615	-0.751633
O	-2.197924	-2.536941	-3.571727

C	-1.506403	-1.492224	-3.705756
C	0.025446	-1.644825	-3.677147
C	1.011840	-0.674590	-3.323167
C	0.507310	-2.919952	-4.025219
N	2.330013	-0.951470	-3.264305
C	1.877855	-3.208675	-3.985383
C	2.748212	-2.186340	-3.581732
H	-0.234572	-3.682190	-4.305990
H	2.261703	-4.206363	-4.244589
H	3.832956	-2.371086	-3.500080
N	0.660141	0.615504	-2.884467
H	-0.092932	1.062115	-3.418036
H	1.468883	1.237299	-2.771443
C	-1.974051	-0.558868	-4.876233
F	-1.404370	0.701184	-4.877120
F	-1.603287	-1.128779	-6.053200
F	-3.305868	-0.387753	-4.910270
H	-0.132367	0.992253	3.902319
H	2.474911	0.180394	3.174690
H	0.322388	-2.489194	-0.977663
H	3.978475	-4.381008	1.725499
H	-4.688027	-2.644304	2.644327
H	-2.434677	-0.363049	4.178494
H	1.854437	4.606317	-2.223585
H	1.207941	4.445020	0.172503
H	-2.142824	5.682864	0.007757
H	5.819861	1.469419	0.963923
H	4.455450	4.313694	-0.168629
H	3.756263	-3.636501	-0.417549
H	5.289463	0.291653	-1.447984
H	-4.965063	-0.370516	1.617118
H	-3.819633	3.806741	1.369201

4A c

Cu1RX-OCF3ALKIPhISOMER1--2Npost SCF Done: -4065.48063416 A.U.

Cu	0.106502	0.100173	1.271903
N	-0.826958	-0.255953	-1.553772
C	-1.104250	-3.113827	-1.248403
H	-0.304714	-2.510216	-1.728259
C	-2.284001	-2.160018	-1.027576
C	-2.124073	-0.764026	-1.201246
C	0.153977	0.089021	-0.653975
C	-3.210020	0.137747	-1.120227
C	-2.947576	1.631825	-1.325074
H	-1.956254	1.833444	-0.854445
C	-3.563447	-2.638582	-0.715009
C	-4.662574	-1.760623	-0.639951
C	-4.492411	-0.383191	-0.886913
C	-0.393997	-0.126635	-2.876441
C	0.900030	0.310870	-2.818113
N	1.208491	0.434954	-1.461899

C	2.468088	0.902653	-0.960500
C	3.499152	-0.038706	-0.717513
C	2.633347	2.279967	-0.720966
C	4.733424	0.441126	-0.266366
C	3.882878	2.739319	-0.261117
C	4.931902	1.823879	-0.037998
C	3.198896	-1.524454	-0.931562
H	2.181864	-1.685696	-0.510164
C	1.444791	3.225604	-0.895305
H	0.757956	2.727035	-1.609459
C	0.635088	3.423748	0.391567
C	1.133092	3.096055	1.667442
C	-0.670071	3.960432	0.293829
C	0.353211	3.312788	2.818790
H	2.127545	2.638419	1.771049
C	-1.459133	4.162079	1.435720
C	-0.941665	3.846298	2.707461
H	0.744516	3.015477	3.802294
H	-1.554125	3.992098	3.610019
C	1.848666	4.542637	-1.555035
C	1.971425	4.597357	-2.960092
C	2.135271	5.699163	-0.801869
C	2.377241	5.779290	-3.600631
H	1.746025	3.694773	-3.553435
C	2.547586	6.882545	-1.440368
C	2.669925	6.926661	-2.840168
H	2.463410	5.807377	-4.698709
H	2.766668	7.779446	-0.839125
H	2.986097	7.856029	-3.339757
C	4.120305	-2.461855	-0.157828
C	5.391012	-2.832643	-0.646927
C	3.692240	-2.966548	1.085736
C	6.225384	-3.676490	0.105719
C	4.520974	-3.818066	1.834510
H	2.696235	-2.698114	1.469690
C	5.792458	-4.171001	1.350291
H	7.215870	-3.957941	-0.286329
H	4.160377	-4.209528	2.798589
H	6.443750	-4.838795	1.936515
C	3.118893	-1.886396	-2.416200
C	2.282461	-2.947953	-2.819021
C	3.854295	-1.192069	-3.398282
C	2.157011	-3.290725	-4.173912
H	1.726399	-3.509163	-2.052341
C	3.734420	-1.535235	-4.757845
C	2.878512	-2.578932	-5.150896
H	1.485679	-4.113905	-4.465288
H	4.312225	-0.980100	-5.514401
H	2.778433	-2.842551	-6.215813
C	-3.902364	2.596715	-0.601775
C	-4.458577	2.277152	0.660773
C	-4.117689	3.895380	-1.112465

C	-5.180882	3.235165	1.392792
H	-4.342182	1.266003	1.088666
C	-4.841167	4.852729	-0.380071
C	-5.371821	4.530212	0.880856
H	-5.593565	2.959913	2.376499
H	-4.987849	5.860259	-0.800854
H	-5.934527	5.281224	1.457457
C	-2.763563	1.948274	-2.813052
C	-1.715803	2.790627	-3.239706
C	-3.614020	1.388363	-3.787958
C	-1.511630	3.063560	-4.601674
H	-1.036875	3.229336	-2.493135
C	-3.410944	1.652220	-5.153679
C	-2.357298	2.488347	-5.566498
H	-0.682636	3.722738	-4.904998
H	-4.079705	1.197898	-5.902104
H	-2.196959	2.690678	-6.637322
C	-0.459436	-3.670131	0.026017
C	-0.645881	-3.062479	1.283338
C	0.445882	-4.754177	-0.069111
C	0.057641	-3.507340	2.420503
C	1.133495	-5.218112	1.062896
H	0.602647	-5.251178	-1.039618
C	0.940633	-4.593427	2.308121
H	-0.043103	-2.985145	3.391989
H	1.838137	-6.059075	0.964955
H	1.492693	-4.939949	3.196253
C	-1.488064	-4.169963	-2.285775
C	-1.297918	-3.886451	-3.654249
C	-2.072271	-5.402796	-1.925844
C	-1.683579	-4.807106	-4.643203
H	-0.830700	-2.930264	-3.941756
C	-2.462499	-6.324601	-2.911897
C	-2.270197	-6.030656	-4.273988
H	-1.521113	-4.569171	-5.706792
H	-2.914851	-7.283818	-2.613397
H	-2.571824	-6.756651	-5.045582
O	-5.856547	-2.329031	-0.306543
C	-6.991663	-1.483634	-0.160424
H	-6.828948	-0.708716	0.623257
H	-7.259837	-0.978915	-1.116818
H	-7.828877	-2.139322	0.144889
O	6.167876	2.171270	0.413757
C	6.425994	3.539445	0.703791
H	7.474032	3.589846	1.054551
H	6.313125	4.183696	-0.198042
H	5.756222	3.924637	1.506458
C	0.475029	-0.203125	3.191212
C	1.616814	-0.128374	2.643840
C	3.061002	-0.229416	2.789594
H	3.670353	-0.229856	1.876438
C	3.537396	-1.069351	3.974515

C	3.675794	0.429953	4.027232
H	2.989055	0.980707	4.689307
H	4.675473	0.877939	3.900921
H	2.727680	-1.521915	4.576468
H	4.437332	-1.685363	3.816961
O	0.147570	-1.636788	5.014435
C	-0.432502	-0.644262	4.369542
C	-1.859740	-0.960218	3.816108
C	-2.478107	-0.326183	2.710049
C	-2.541468	-2.038348	4.392789
N	-3.650843	-0.720712	2.186272
C	-3.774950	-2.455116	3.864455
C	-4.279125	-1.769918	2.748645
H	-2.032608	-2.552565	5.224831
H	-4.323415	-3.309532	4.289672
H	-5.218572	-2.086739	2.262478
N	-1.817866	0.725728	2.001200
H	-1.527635	1.498753	2.620278
H	-2.442931	1.107364	1.283482
C	-0.579782	0.612335	5.312079
F	-1.297911	1.653394	4.735360
F	-1.235510	0.288751	6.441316
F	0.620866	1.138553	5.662996
H	1.634821	0.518911	-3.600010
H	-1.058047	-0.332055	-3.720718
H	-1.357689	-2.227591	1.378787
H	-2.200292	-5.647704	-0.859564
H	5.721236	-2.466254	-1.632344
H	4.510621	-0.360216	-3.095597
H	-2.481415	4.557767	1.328636
H	-1.075016	4.239943	-0.691946
H	2.018404	5.671016	0.293362
H	-4.430414	0.720209	-3.471773
H	-3.703361	4.168167	-2.094559
H	-3.739429	-3.709613	-0.544102
H	-5.342401	0.307670	-0.835938
H	5.561433	-0.249248	-0.049888
H	4.012182	3.814762	-0.081487

4A c′

Cu1RX-OCF3ALKIPhISOMER1--2Opost SCF Done: -4065.49295303 A.U.

Cu	-0.414486	-0.133289	-0.986254
N	-1.111885	0.471045	1.772122
C	-4.008810	0.713298	1.548575
H	-3.493297	1.432318	2.222550
C	-3.319649	-0.643731	1.733499
C	-1.909599	-0.723895	1.869094
C	-0.352194	0.707912	0.655404
C	-1.241779	-1.969278	1.982508
C	0.281814	-2.050686	2.131606
H	0.694905	-1.233638	1.497883

C	-4.052934	-1.837645	1.703223
C	-3.404513	-3.087591	1.795494
C	-2.007272	-3.146938	1.953254
C	-0.862447	1.428548	2.760240
C	0.073832	2.292278	2.246165
N	0.381156	1.824220	0.969865
C	1.468200	2.208242	0.107045
C	1.188555	2.883577	-1.107292
C	2.753983	1.721684	0.413227
C	2.238071	3.079710	-2.011498
C	3.797197	1.935523	-0.509307
C	3.538878	2.605970	-1.723201
C	-0.222362	3.425331	-1.347706
H	-0.936608	2.648943	-0.992757
C	2.991136	0.977008	1.724059
H	2.027267	0.503732	2.006902
C	3.946270	-0.207540	1.579162
C	3.594175	-1.231197	0.671612
C	5.080772	-0.380255	2.394071
C	4.336308	-2.418713	0.606558
H	2.702814	-1.106270	0.034489
C	5.838326	-1.564603	2.317642
C	5.462570	-2.590489	1.433412
H	4.010416	-3.220595	-0.071937
H	6.039710	-3.527746	1.392157
C	3.326449	1.925833	2.873592
C	2.716005	1.726406	4.130269
C	4.241858	2.988061	2.725462
C	3.023078	2.558875	5.218518
H	1.987419	0.910659	4.250539
C	4.549998	3.823968	3.813200
C	3.943511	3.611176	5.064182
H	2.536078	2.382066	6.190836
H	5.264567	4.652000	3.680287
H	4.183251	4.268902	5.914821
C	-0.581501	3.625122	-2.819376
C	-1.081042	4.842259	-3.320645
C	-0.519360	2.505870	-3.682614
C	-1.510987	4.944716	-4.657454
C	-0.946264	2.608604	-5.014854
H	-0.183935	1.525090	-3.303656
C	-1.445556	3.829363	-5.508531
H	-1.905714	5.903805	-5.029936
H	-0.903699	1.717943	-5.658769
H	-1.787559	3.907156	-6.553065
C	-0.410594	4.643947	-0.444599
C	-1.416279	4.651338	0.541399
C	0.450791	5.758645	-0.542264
C	-1.573428	5.752355	1.401531
H	-2.078163	3.777023	0.630027
C	0.293187	6.863040	0.310350
C	-0.721210	6.863957	1.286694

H	-2.365889	5.738350	2.167146
H	0.971008	7.726761	0.218114
H	-0.841511	7.728154	1.959310
C	0.848940	-3.337023	1.532265
C	1.078008	-3.401186	0.143087
C	1.137018	-4.470715	2.320311
C	1.623652	-4.552202	-0.445105
H	0.836808	-2.529974	-0.490346
C	1.663693	-5.634582	1.732366
C	1.916724	-5.674293	0.350405
H	1.826195	-4.563661	-1.526352
H	1.885961	-6.511243	2.362031
H	2.337902	-6.581505	-0.111092
C	0.783842	-1.764171	3.553599
C	2.165681	-1.892538	3.831277
C	-0.068794	-1.342414	4.594013
C	2.677324	-1.596104	5.102873
H	2.846938	-2.235574	3.039238
C	0.443370	-1.040434	5.870498
C	1.818333	-1.164930	6.130864
H	3.759692	-1.689618	5.282074
H	-0.242974	-0.713746	6.668322
H	2.219822	-0.927224	7.128555
C	-3.850010	1.276420	0.131544
C	-3.401337	0.498048	-0.952271
C	-4.182671	2.628168	-0.104546
C	-3.261520	1.055511	-2.238214
C	-4.053023	3.188010	-1.385899
H	-4.551600	3.246196	0.731102
C	-3.586636	2.403690	-2.456939
H	-2.840757	0.432626	-3.041998
H	-4.292367	4.251139	-1.544939
H	-3.445763	2.851107	-3.452660
C	-5.470428	0.675021	1.999816
C	-5.782629	0.790859	3.370836
C	-6.520575	0.471407	1.081060
C	-7.111670	0.706429	3.816465
H	-4.967777	0.944020	4.098431
C	-7.851640	0.381279	1.524305
C	-8.152028	0.499046	2.892477
H	-7.337575	0.804337	4.890413
H	-8.660119	0.220987	0.793275
H	-9.195573	0.433140	3.239031
O	-4.205748	-4.185242	1.700952
C	-3.612253	-5.472079	1.858281
H	-2.842243	-5.670432	1.079534
H	-3.146085	-5.586718	2.863233
H	-4.434158	-6.204757	1.752534
O	4.475666	2.827639	-2.687130
C	5.799232	2.363365	-2.467041
H	6.379480	2.636645	-3.368558
H	6.265991	2.841804	-1.575245

H	5.834884	1.257398	-2.336533
C	1.336433	-1.753435	-3.153315
C	2.525026	-1.518572	-2.953900
C	3.914971	-1.225393	-2.724757
H	4.084142	-0.394127	-2.014523
C	4.932399	-1.365345	-3.858496
C	4.958785	-2.340075	-2.709400
H	4.591547	-3.364245	-2.882538
H	5.758771	-2.257195	-1.956633
H	4.550579	-1.708763	-4.833013
H	5.722007	-0.599050	-3.922604
O	-0.728028	-0.714994	-2.692643
C	-0.141455	-1.803666	-3.310874
C	-0.733083	-3.155298	-2.810390
C	-0.193627	-4.453836	-3.079406
C	-1.876314	-3.099927	-2.004141
N	-0.733818	-5.582257	-2.563473
C	-2.443688	-4.268039	-1.468566
C	-1.818404	-5.484062	-1.782636
H	-2.300204	-2.107577	-1.795621
H	-3.341671	-4.226529	-0.834744
H	-2.214685	-6.438772	-1.387002
N	0.965808	-4.652983	-3.805385
H	1.250135	-3.941989	-4.478267
H	1.117030	-5.626822	-4.076605
C	-0.493592	-1.613662	-4.829835
F	0.032618	-0.465550	-5.318918
F	-0.012414	-2.628039	-5.615627
F	-1.829057	-1.568503	-5.012411
H	0.559647	3.173251	2.673007
H	-1.347831	1.394426	3.739125
H	-3.149403	-0.560778	-0.788971
H	-6.287706	0.387877	0.007903
H	-1.158239	5.716339	-2.656545
H	1.255974	5.751007	-1.294364
H	6.718602	-1.690918	2.968280
H	5.353702	0.404678	3.115542
H	4.710095	3.169527	1.745372
H	-1.150653	-1.259170	4.407316
H	0.963994	-4.433649	3.407540
H	-5.145831	-1.821319	1.588836
H	-1.483450	-4.108846	2.011393
H	2.059125	3.576900	-2.975720
H	4.790969	1.530971	-0.273456
4C cat			
Cu2RX SCF Done: -3657.38549032 A.U.			
O	0.579218	-6.111801	-1.943447
O	-0.705411	6.340666	-0.308516
C	1.827422	-6.576842	-2.442619
H	2.255603	-5.881458	-3.200047
H	1.625413	-7.553444	-2.921726

H	2.572607	-6.721031	-1.626688
C	0.437055	7.166239	-0.493558
H	1.061835	7.224146	0.427600
H	0.054594	8.176782	-0.732150
H	1.073359	6.810441	-1.335727
Cu	-0.112433	0.437732	-1.829264
Cl	0.069255	1.253935	-3.796810
C	-0.093309	-0.012291	-0.023770
C	0.846096	3.060902	0.481835
C	-1.611101	2.871450	0.357110
N	0.083077	-1.186910	0.656766
N	-0.241347	0.922236	0.968543
C	-2.829800	1.946870	0.303986
H	-2.461394	0.973963	-0.092099
C	2.197129	2.348798	0.580541
H	2.013688	1.311520	0.226673
C	-0.181805	0.355234	2.248974
C	0.539379	-4.903478	-1.318291
C	-2.885093	-1.915875	-1.131439
C	-6.141063	2.955151	-1.418306
H	-7.203609	3.093082	-1.160952
C	-3.364579	1.669163	1.704038
C	1.531973	-2.853297	-0.430555
C	-0.901963	-3.241797	-0.253375
C	-3.863555	2.405474	-0.723527
C	-0.341805	2.317569	0.647710
C	2.660029	2.225025	2.027261
C	3.224448	2.909186	-0.404947
C	-2.286234	-2.676181	0.062203
H	-2.133176	-1.914444	0.856829
C	2.759051	-1.981854	-0.166433
H	2.371222	-0.980170	0.117747
C	3.597035	-2.452820	1.025747
C	5.379222	3.909467	-0.977033
H	6.347529	4.324803	-0.654104
C	-3.296340	-3.870618	2.060144
H	-2.691385	-3.209698	2.703681
C	-0.738558	-4.475049	-0.892472
H	-1.602188	-5.122048	-1.100701
C	0.249525	-2.450467	-0.002861
C	0.029762	-0.995111	2.052139
C	4.464080	3.446463	-0.010985
H	4.723247	3.494841	1.058018
C	3.009595	0.960854	2.540342
H	2.946053	0.078889	1.888111
C	1.665486	-4.084895	-1.100417
H	2.662615	-4.380305	-1.452513
C	3.459091	1.919625	4.728658
H	3.764980	1.803421	5.780554
C	0.751026	4.427795	0.164109
H	1.680067	4.990461	0.000505
C	4.670868	-2.561079	-1.795440

H	5.016542	-3.341772	-1.098269
C	3.111418	3.189482	4.230224
H	3.143415	4.069022	4.893037
C	-3.655604	2.720385	2.600149
H	-3.506350	3.762000	2.273978
C	3.241961	-3.558886	1.823969
H	2.361310	-4.162598	1.556425
C	4.740483	-1.702816	1.387365
H	5.037605	-0.842584	0.766505
C	3.565253	-1.757385	-1.447425
C	3.828025	-0.529258	-3.548240
H	3.477190	0.271625	-4.217238
C	5.345838	-2.352658	-3.011854
H	6.211221	-2.984110	-3.270458
C	-3.230065	-3.714253	0.659582
C	-0.515086	5.025112	-0.013410
C	-4.126336	-4.846066	2.638444
H	-4.168887	-4.950774	3.734438
C	-5.222843	2.591914	-0.413856
H	-5.572490	2.440459	0.619059
C	-1.686116	4.236140	0.054940
H	-2.648324	4.703228	-0.200186
C	-4.111049	2.447850	3.901596
H	-4.329628	3.280185	4.589494
C	-3.991883	0.063067	3.444858
H	-4.118286	-0.984245	3.763171
C	-2.178804	-1.709181	-2.333604
H	-1.171442	-2.133455	-2.462996
C	-2.747478	-0.963139	-3.384636
H	-2.153892	-0.781637	-4.292666
C	-3.543867	0.342475	2.143440
H	-3.314842	-0.480821	1.452432
C	-4.856895	-5.525376	0.421601
H	-5.474465	-6.167122	-0.226915
C	5.065047	3.835025	-2.343675
H	5.784637	4.191790	-3.097900
C	-4.275719	1.117426	4.330803
H	-4.623678	0.904716	5.354100
C	-4.029539	-4.545694	-0.152890
H	-4.014288	-4.403777	-1.245316
C	2.712764	3.340513	2.890591
H	2.420142	4.331975	2.509941
C	-4.738402	-0.597117	-2.043764
H	-5.728531	-0.133460	-1.913055
C	-5.705428	3.141650	-2.740381
H	-6.423032	3.428532	-3.525654
C	-4.345324	2.955694	-3.055898
H	-3.991401	3.083118	-4.091227
C	-4.906122	-5.681108	1.818682
H	-5.559547	-6.445387	2.268400
C	-3.433583	2.582980	-2.058990
H	-2.376667	2.401697	-2.323553

C	-4.172642	-1.344753	-1.000644
H	-4.743418	-1.499230	-0.071104
C	3.408470	0.802140	3.876963
H	3.678021	-0.201540	4.243021
C	3.160064	-0.739440	-2.333395
H	2.297015	-0.101607	-2.077157
C	5.490340	-2.033463	2.525673
H	6.368953	-1.425868	2.794233
C	2.913247	2.839700	-1.783410
H	1.944753	2.421312	-2.111078
C	3.995684	-3.895767	2.964739
H	3.698419	-4.763116	3.575812
C	3.826478	3.296265	-2.743461
H	3.564882	3.224781	-3.811139
C	-4.027611	-0.409464	-3.243169
H	-4.465595	0.192644	-4.052879
C	4.925114	-1.339207	-3.892571
H	5.457151	-1.178121	-4.843761
C	5.118021	-3.131925	3.323863
H	5.705508	-3.393157	4.218305
C	-0.330158	1.158854	3.494277
H	-0.249716	0.507712	4.384549
H	0.460135	1.934788	3.563244
H	-1.314492	1.670329	3.520130
C	0.223965	-2.108991	3.024325
H	-0.281965	-3.034686	2.680673
H	1.301176	-2.352827	3.149171
H	-0.185661	-1.833104	4.014886

4C a

Cu2RX-ALKI SCF Done: -3390.54632641 A.U.

Cu	0.128219	-0.312323	1.635896
N	-0.107200	-1.040274	-1.135894
C	2.711527	-1.185272	-1.416444
H	2.071485	-0.292793	-1.263026
C	1.921409	-2.377055	-0.879814
C	0.512006	-2.282546	-0.768178
C	-0.220523	-0.046238	-0.200233
C	-0.253903	-3.284667	-0.142539
C	-1.749744	-3.046127	0.072697
H	-1.862506	-1.966313	0.317827
C	2.547573	-3.546928	-0.433283
C	1.788050	-4.597707	0.129198
C	0.391922	-4.458103	0.286389
C	-0.401604	-0.590711	-2.429196
C	-0.697051	0.752851	-2.301518
N	-0.579532	1.056411	-0.927075
C	-0.801411	2.337276	-0.313191
C	0.303798	3.085081	0.184745
C	-2.131022	2.795961	-0.155437
C	0.046476	4.331776	0.769186

C	-2.353548	4.055562	0.430842
C	-1.267672	4.832000	0.879680
C	1.741919	2.556012	0.110057
H	1.676772	1.450512	0.223946
C	-3.316998	1.889406	-0.507781
H	-3.013516	1.283573	-1.390368
C	-3.617030	0.863192	0.598563
C	-2.820539	0.730976	1.752923
C	-4.684592	-0.045594	0.413859
C	-3.046951	-0.311798	2.672532
H	-1.989225	1.430115	1.932539
C	-4.922391	-1.077095	1.335833
C	-4.093655	-1.223299	2.463127
H	-2.361274	-0.424159	3.527129
H	-4.247795	-2.062278	3.158911
C	-4.541401	2.694241	-0.939869
C	-4.663257	3.120789	-2.278906
C	-5.536467	3.078967	-0.016137
C	-5.747088	3.915799	-2.687299
H	-3.894045	2.823534	-3.010458
C	-6.620666	3.877321	-0.420048
C	-6.729627	4.299453	-1.756894
H	-5.827330	4.234542	-3.738920
H	-7.389038	4.166807	0.314663
H	-7.581941	4.920502	-2.075170
C	2.583579	3.034727	1.297245
C	3.433385	4.156704	1.205082
C	2.480983	2.360410	2.531705
C	4.168536	4.592302	2.322058
C	3.216266	2.792555	3.646593
H	1.822898	1.479500	2.629779
C	4.064287	3.910544	3.547147
H	4.829284	5.469516	2.231638
H	3.124271	2.244390	4.597692
H	4.643670	4.248509	4.421244
C	2.422266	2.797909	-1.238157
C	3.748249	2.337964	-1.418253
C	1.787916	3.455491	-2.309663
C	4.411491	2.518863	-2.639490
H	4.262814	1.837560	-0.584641
C	2.452491	3.636850	-3.538823
C	3.765819	3.168584	-3.708761
H	5.436107	2.133633	-2.758066
H	1.938219	4.155449	-4.364151
H	4.284913	3.304788	-4.670470
C	-2.305723	-3.765694	1.299668
C	-1.690974	-3.513866	2.549136
C	-3.448658	-4.584681	1.256330
C	-2.207183	-4.076882	3.724499
H	-0.816982	-2.840469	2.604344
C	-3.970793	-5.146087	2.438144
C	-3.352204	-4.896717	3.674454

H	-1.719923	-3.856151	4.687504
H	-4.870901	-5.779883	2.388290
H	-3.762985	-5.334402	4.598418
C	-2.498615	-3.274355	-1.236372
C	-3.315177	-2.257527	-1.768909
C	-2.354276	-4.475720	-1.962976
C	-3.983922	-2.434873	-2.992232
H	-3.413865	-1.314553	-1.211933
C	-3.021459	-4.658784	-3.185716
C	-3.839801	-3.638042	-3.705541
H	-4.613777	-1.624210	-3.392617
H	-2.896266	-5.601742	-3.741626
H	-4.358008	-3.779556	-4.667309
C	3.955260	-0.877118	-0.581482
C	3.766351	-0.555766	0.782812
C	5.245691	-0.779856	-1.133772
C	4.838057	-0.105878	1.566317
C	6.325682	-0.341619	-0.343221
H	5.400631	-1.015239	-2.197998
C	6.123927	0.008468	1.002753
H	4.657216	0.177325	2.614553
H	7.329593	-0.259649	-0.790206
H	6.965361	0.372330	1.613395
C	2.934129	-1.255664	-2.924168
C	2.649536	-0.121222	-3.712660
C	3.394056	-2.425887	-3.563219
C	2.824090	-0.150117	-5.105070
H	2.273514	0.790888	-3.225684
C	3.567051	-2.459175	-4.958127
C	3.281802	-1.321407	-5.734647
H	2.598196	0.751232	-5.696900
H	3.922734	-3.382831	-5.442203
H	3.414021	-1.349855	-6.828036
O	2.495558	-5.691453	0.526932
C	1.803988	-6.744393	1.186201
H	1.315259	-6.395788	2.124409
H	1.030896	-7.208423	0.531112
H	2.565844	-7.506596	1.437389
O	-1.389391	6.062015	1.450153
C	-2.692560	6.601792	1.627717
H	-2.556330	7.591625	2.103026
H	-3.224103	6.734867	0.657486
H	-3.318647	5.963852	2.292852
C	0.615734	-0.607803	3.387122
C	1.035333	-0.747915	4.550273
C	1.571880	-0.853665	5.886485
H	1.235177	-1.730674	6.470898
C	1.845985	0.410989	6.705138
C	3.004992	-0.393038	6.168414
H	3.568398	0.005087	5.308831
H	3.605822	-0.988243	6.875677
H	1.601149	1.370892	6.222074

H	1.638651	0.375691	7.787428
H	2.759236	-0.636114	1.231764
H	3.603152	-3.325661	-2.964087
H	3.528586	4.688036	0.244879
H	0.765323	3.839924	-2.177585
H	-5.741902	-1.790821	1.157609
H	-5.330570	0.054329	-0.473827
H	-5.458850	2.736315	1.027904
H	-1.699410	-5.268709	-1.567653
H	-3.948341	-4.771308	0.293281
H	3.643357	-3.641224	-0.455797
H	-0.205912	-5.226199	0.794498
H	0.869088	4.938094	1.173734
H	-3.386734	4.408131	0.541816
C	-1.023311	1.762954	-3.353359
H	-1.457924	2.677316	-2.903807
H	-0.109908	2.068459	-3.908014
H	-1.750059	1.354840	-4.084411
C	-0.391720	-1.486661	-3.621223
H	-0.360473	-0.890224	-4.552445
H	0.499971	-2.144663	-3.614591
H	-1.293465	-2.134810	-3.637284

4C a+b

Cu2RX-OCF3ALKIPh--2Npre SCF Done: -4144.06836740 A.U.

Cu	0.588279	0.325833	0.961694
N	-1.597120	-0.517685	-1.002750
C	-0.763313	-3.221457	-1.086450
H	-0.247174	-2.300874	-1.424092
C	-1.872641	-2.776831	-0.132013
C	-2.293996	-1.424914	-0.142108
C	-0.444509	0.117107	-0.612038
C	-3.341156	-0.952114	0.674775
C	-3.674720	0.542025	0.647798
H	-2.695374	1.065865	0.553048
C	-2.527425	-3.664997	0.731254
C	-3.620676	-3.227912	1.509628
C	-4.033846	-1.880412	1.472040
C	-1.862773	-0.349456	-2.372569
C	-0.843742	0.442461	-2.860106
N	0.008701	0.695061	-1.771072
C	1.180838	1.528255	-1.784385
C	2.476197	0.941241	-1.869154
C	1.002785	2.918797	-1.615881
C	3.574189	1.806541	-1.905497
C	2.129920	3.762088	-1.658128
C	3.413868	3.205903	-1.813488
C	2.658617	-0.579813	-1.763995
H	1.889484	-0.910802	-1.029172
C	-0.371637	3.471274	-1.221482
H	-1.123978	2.738531	-1.578780

C	-0.547405	3.548797	0.305958
C	0.541537	3.504578	1.197323
C	-1.848120	3.672258	0.848018
C	0.340722	3.580441	2.588679
H	1.561270	3.364073	0.817547
C	-2.056647	3.721487	2.235540
C	-0.956800	3.681318	3.114248
H	1.214961	3.511497	3.254031
H	-1.118419	3.715523	4.203438
C	-0.701288	4.787418	-1.921601
C	-1.264005	4.763925	-3.215888
C	-0.431199	6.035461	-1.323181
C	-1.540941	5.957608	-3.902058
H	-1.483757	3.792049	-3.688187
C	-0.702558	7.232280	-2.009653
C	-1.257650	7.197766	-3.300791
H	-1.983231	5.920897	-4.910593
H	-0.486728	8.199358	-1.527777
H	-1.476567	8.135454	-3.835774
C	4.001551	-0.994817	-1.156094
C	5.173893	-1.062623	-1.940719
C	4.074586	-1.349401	0.204145
C	6.396380	-1.448990	-1.365361
C	5.290497	-1.755352	0.778837
H	3.174442	-1.275788	0.828095
C	6.457344	-1.797961	-0.002648
H	7.305198	-1.488017	-1.987229
H	5.305766	-2.020267	1.847047
H	7.415769	-2.103879	0.446569
C	2.410955	-1.376589	-3.042795
C	2.418509	-2.788882	-2.953102
C	2.244626	-0.780074	-4.305721
C	2.258225	-3.580446	-4.097549
H	2.558182	-3.265884	-1.969894
C	2.073208	-1.575045	-5.457393
C	2.082908	-2.976143	-5.357932
H	2.250522	-4.677201	-4.000203
H	1.945861	-1.091355	-6.439632
H	1.951564	-3.597941	-6.257494
C	-4.285929	1.112662	1.932804
C	-3.793749	0.710649	3.197024
C	-5.245891	2.145539	1.886194
C	-4.227446	1.347278	4.371885
H	-3.072780	-0.124702	3.271770
C	-5.684127	2.778242	3.064131
C	-5.170723	2.389213	4.312959
H	-3.819969	1.020938	5.342526
H	-6.429321	3.587293	3.000421
H	-5.508179	2.889934	5.234269
C	-4.478452	0.893832	-0.603682
C	-4.031302	1.894298	-1.489077
C	-5.693215	0.236091	-0.889376

C	-4.778539	2.245092	-2.626189
H	-3.068504	2.387265	-1.295727
C	-6.445610	0.580609	-2.024528
C	-5.992460	1.588963	-2.896436
H	-4.402893	3.029592	-3.302277
H	-7.390594	0.054846	-2.234557
H	-6.581918	1.856397	-3.787607
C	0.356460	-4.015532	-0.411778
C	1.080186	-3.378045	0.620670
C	0.787663	-5.277103	-0.864578
C	2.228992	-3.968703	1.166008
C	1.931384	-5.880325	-0.306921
H	0.245471	-5.776303	-1.682128
C	2.660441	-5.224376	0.699328
H	2.797010	-3.427185	1.937842
H	2.263072	-6.863439	-0.678145
H	3.571021	-5.684012	1.114932
C	-1.331182	-3.845371	-2.362130
C	-0.922053	-3.337052	-3.612229
C	-2.254829	-4.912182	-2.340815
C	-1.410107	-3.883015	-4.810313
H	-0.219140	-2.491212	-3.642891
C	-2.749397	-5.458913	-3.537365
C	-2.327963	-4.946746	-4.778098
H	-1.065357	-3.469381	-5.771265
H	-3.470157	-6.291416	-3.499592
H	-2.716587	-5.375546	-5.715587
O	-4.190550	-4.175209	2.314234
C	-5.265338	-3.785525	3.161089
H	-4.957339	-2.994163	3.882623
H	-6.140614	-3.418018	2.578075
H	-5.562164	-4.691682	3.722330
O	4.568386	3.931632	-1.838606
C	4.487308	5.333825	-1.627456
H	5.525582	5.714914	-1.661551
H	3.889217	5.842279	-2.418476
H	4.044485	5.575510	-0.633807
C	2.247976	0.825384	1.698249
C	3.311581	1.473819	1.772641
C	4.559689	2.185578	1.830036
H	4.482495	3.257722	2.094334
C	5.703356	1.827259	0.871994
C	5.825085	1.478672	2.328291
H	5.696910	0.424754	2.619699
H	6.505476	2.062283	2.969850
H	5.514159	1.003533	0.167872
H	6.288774	2.664986	0.460501
O	3.409151	-1.503034	3.473079
C	2.409464	-0.863759	3.802696
C	1.040983	-1.483983	3.769212
C	-0.236230	-0.870808	3.515897
C	1.030219	-2.875501	4.017628

N	-1.389067	-1.580159	3.544369
C	-0.163765	-3.599954	4.040966
C	-1.353118	-2.891178	3.804146
H	1.995375	-3.369175	4.203608
H	-0.177124	-4.681098	4.239320
H	-2.325287	-3.412807	3.819084
N	-0.377746	0.435679	3.086074
H	0.335126	1.117111	3.357323
H	-1.335348	0.799551	3.075654
C	2.643998	0.337023	4.776217
F	1.721802	1.347866	4.715511
F	2.549707	-0.173119	6.040105
F	3.858498	0.877291	4.644054
H	0.755855	-2.383041	0.977320
H	-2.597646	-5.318521	-1.377611
H	5.122279	-0.822811	-3.015525
H	2.266539	0.317762	-4.388650
H	-3.082023	3.786209	2.631154
H	-2.717300	3.739112	0.175725
H	-0.018194	6.062366	-0.301998
H	-6.046093	-0.559280	-0.213586
H	-5.646880	2.473647	0.915451
H	-2.190144	-4.706937	0.832304
H	-4.849819	-1.520817	2.111694
H	4.597460	1.412662	-1.949011
H	1.983424	4.842943	-1.532262
C	-0.634313	0.980023	-4.237353
H	0.102949	1.806771	-4.226563
H	-0.251219	0.196165	-4.924609
H	-1.587432	1.364882	-4.654257
C	-3.030858	-0.957055	-3.075150
H	-2.770757	-1.173042	-4.128985
H	-3.318241	-1.916162	-2.602283
H	-3.915845	-0.289145	-3.060161

4C a+b→c

Cu2RX-OCF3ALKIPhISOMER1--2N SCF Done: -4144.06595470 A.U.

Cu	0.458979	0.236728	1.090912
N	-1.496516	-0.485360	-1.085555
C	-0.702538	-3.199327	-1.190768
H	-0.154400	-2.277442	-1.469746
C	-1.861523	-2.762003	-0.294216
C	-2.258976	-1.403077	-0.293341
C	-0.362362	0.126796	-0.610672
C	-3.355117	-0.933777	0.458942
C	-3.660973	0.566290	0.447422
H	-2.667301	1.070833	0.429952
C	-2.585014	-3.660151	0.501030
C	-3.722607	-3.224939	1.213997
C	-4.116982	-1.871436	1.177829
C	-1.686980	-0.267045	-2.460141

C	-0.637935	0.531546	-2.864117
N	0.156209	0.738938	-1.721908
C	1.329934	1.561137	-1.656282
C	2.620814	0.965465	-1.717659
C	1.153068	2.949558	-1.475257
C	3.723125	1.827505	-1.738938
C	2.283750	3.788920	-1.491460
C	3.565992	3.227462	-1.643720
C	2.780048	-0.560021	-1.632050
H	1.961670	-0.897450	-0.956459
C	-0.230893	3.494150	-1.107928
H	-0.972041	2.778938	-1.519340
C	-0.458621	3.511938	0.413586
C	0.600334	3.438674	1.338255
C	-1.776934	3.612893	0.915859
C	0.356567	3.473750	2.724148
H	1.629612	3.320062	0.975404
C	-2.029096	3.624371	2.296620
C	-0.957787	3.564078	3.208537
H	1.203126	3.378373	3.421039
H	-1.153507	3.568965	4.292529
C	-0.533908	4.837640	-1.765759
C	-1.065820	4.864872	-3.072881
C	-0.271057	6.060923	-1.115385
C	-1.320908	6.084682	-3.720595
H	-1.279871	3.912352	-3.585820
C	-0.520651	7.283775	-1.763288
C	-1.046040	7.300055	-3.067146
H	-1.739944	6.087709	-4.739604
H	-0.311679	8.231168	-1.241011
H	-1.248415	8.258145	-3.571542
C	4.077377	-0.996927	-0.947201
C	5.307750	-1.020141	-1.641350
C	4.052787	-1.397825	0.401577
C	6.491069	-1.392034	-0.980915
C	5.231635	-1.778885	1.064906
H	3.107055	-1.383899	0.958337
C	6.456477	-1.766844	0.376632
H	7.445693	-1.396255	-1.531344
H	5.160560	-2.059870	2.126751
H	7.386630	-2.054796	0.892525
C	2.605429	-1.324488	-2.942308
C	2.590760	-2.738067	-2.882250
C	2.522259	-0.698476	-4.199077
C	2.491347	-3.502737	-4.051564
H	2.660819	-3.237590	-1.902679
C	2.410780	-1.466646	-5.375988
C	2.398530	-2.869506	-5.306499
H	2.465473	-4.601058	-3.979153
H	2.347472	-0.960780	-6.353228
H	2.314302	-3.470439	-6.225644
C	-4.349953	1.127594	1.698483

C	-4.006387	0.656740	2.988129
C	-5.235133	2.222878	1.601121
C	-4.509846	1.286220	4.139555
H	-3.342715	-0.220998	3.103168
C	-5.743387	2.847412	2.754423
C	-5.376670	2.388238	4.031121
H	-4.216696	0.905449	5.131336
H	-6.427025	3.705147	2.650336
H	-5.768722	2.881804	4.934480
C	-4.373883	0.965029	-0.845287
C	-3.862881	1.988581	-1.667769
C	-5.573488	0.330506	-1.228723
C	-4.534836	2.384705	-2.836742
H	-2.909754	2.465861	-1.399986
C	-6.250506	0.719524	-2.396289
C	-5.735549	1.751458	-3.203973
H	-4.111239	3.187871	-3.460669
H	-7.185128	0.211137	-2.681807
H	-6.265964	2.055341	-4.120172
C	0.365117	-4.027724	-0.473816
C	1.007871	-3.441470	0.638935
C	0.836694	-5.262930	-0.957788
C	2.119907	-4.051266	1.237015
C	1.942326	-5.888141	-0.349151
H	0.358283	-5.723093	-1.836151
C	2.592291	-5.280106	0.738473
H	2.624809	-3.537051	2.071058
H	2.307012	-6.850416	-0.743590
H	3.474037	-5.757170	1.194913
C	-1.202535	-3.777187	-2.515060
C	-0.715628	-3.240344	-3.724654
C	-2.135825	-4.833997	-2.578602
C	-1.137909	-3.747052	-4.964502
H	-0.002602	-2.403089	-3.691075
C	-2.565342	-5.341140	-3.816868
C	-2.067196	-4.799971	-5.016218
H	-0.732402	-3.311448	-5.891484
H	-3.295164	-6.166123	-3.844525
H	-2.404424	-5.198171	-5.986530
O	-4.359735	-4.181782	1.952797
C	-5.484392	-3.796529	2.733720
H	-5.216821	-3.024695	3.491472
H	-6.314441	-3.406087	2.101319
H	-5.828607	-4.710429	3.253834
O	4.723799	3.950391	-1.658695
C	4.642033	5.359260	-1.491202
H	5.680525	5.738716	-1.533178
H	4.046558	5.841100	-2.300167
H	4.195364	5.633000	-0.507597
C	2.068736	0.412258	2.159897
C	2.996023	1.250837	2.028580
C	4.099763	2.155774	1.956522

H	3.852158	3.226790	2.088177
C	5.331053	1.863045	1.086993
C	5.443742	1.712475	2.570717
H	5.454386	0.692643	2.985898
H	5.984485	2.469067	3.161852
H	5.300644	0.943090	0.487121
H	5.783636	2.732554	0.584467
O	3.129345	-1.662441	3.483391
C	2.136640	-0.907429	3.668672
C	0.745815	-1.564407	3.710355
C	-0.522381	-0.976951	3.417369
C	0.734744	-2.928077	4.054660
N	-1.672816	-1.680063	3.411580
C	-0.461122	-3.658674	4.070809
C	-1.640470	-2.984189	3.724979
H	1.702197	-3.398011	4.285685
H	-0.477168	-4.727987	4.328124
H	-2.604694	-3.519662	3.688418
N	-0.642224	0.356762	2.984760
H	-0.043361	1.030461	3.474128
H	-1.614730	0.680412	2.930491
C	2.326430	0.119197	4.838444
F	1.367058	1.112201	4.900292
F	2.235798	-0.550895	6.017644
F	3.523741	0.729080	4.810360
H	0.648749	-2.470681	1.025462
H	-2.537021	-5.264702	-1.649080
H	5.333032	-0.756557	-2.711599
H	2.559839	0.400705	-4.256805
H	-3.067145	3.675219	2.659847
H	-2.625212	3.692267	0.218456
H	0.118166	6.048380	-0.084560
H	-5.974708	-0.482150	-0.602456
H	-5.520946	2.605296	0.609815
H	-2.270132	-4.709528	0.596979
H	-4.973317	-1.516765	1.764998
H	4.745168	1.430158	-1.780728
H	2.139980	4.869425	-1.358954
C	-0.356230	1.113323	-4.209877
H	0.362822	1.952882	-4.135235
H	0.079176	0.356379	-4.896303
H	-1.292040	1.492194	-4.669634
C	-2.823285	-0.829441	-3.247367
H	-3.255609	-1.710657	-2.735602
H	-3.632929	-0.084061	-3.388161
H	-2.473924	-1.161581	-4.243929

4C c

Cu2RX-OCF3ALKIPh--2NpostDiss SCF Done: -4144.08303911 A.U.

Cu	0.342564	-0.387647	-1.189021
N	0.411637	0.422608	1.652625

C	1.006365	-2.325313	2.093719
H	0.249504	-1.968659	1.366241
C	2.123607	-1.282955	2.074481
C	1.796948	0.067024	1.798372
C	-0.293424	0.307141	0.478536
C	2.784895	1.072312	1.727003
C	2.386796	2.470479	1.251835
H	1.555571	2.304362	0.526695
C	3.457859	-1.607481	2.342093
C	4.444744	-0.599409	2.369640
C	4.106396	0.735771	2.071622
C	-0.396128	0.846639	2.721817
C	-1.653908	1.034482	2.194449
N	-1.564485	0.684961	0.835664
C	-2.648620	0.787997	-0.096083
C	-3.650468	-0.213983	-0.130149
C	-2.686019	1.905559	-0.959385
C	-4.693406	-0.074621	-1.058021
C	-3.739748	2.018001	-1.884113
C	-4.740426	1.025446	-1.941902
C	-3.539905	-1.457829	0.751099
H	-2.617047	-1.323450	1.351920
C	-1.588489	2.966558	-0.886206
H	-1.083568	2.809827	0.087992
C	-0.487528	2.813349	-1.937945
C	-0.607977	1.976989	-3.063533
C	0.718144	3.528996	-1.753679
C	0.450484	1.855086	-3.983803
H	-1.521513	1.386077	-3.214779
C	1.779557	3.404841	-2.661954
C	1.646348	2.564690	-3.785964
H	0.355663	1.158911	-4.829514
H	2.480240	2.448879	-4.495565
C	-2.181681	4.373639	-0.825236
C	-2.685232	4.849850	0.404952
C	-2.275423	5.199238	-1.963413
C	-3.275508	6.120328	0.496702
H	-2.611564	4.208016	1.299204
C	-2.871394	6.470313	-1.875405
C	-3.372487	6.934771	-0.646876
H	-3.659441	6.478963	1.465189
H	-2.937016	7.105862	-2.772992
H	-3.832407	7.933250	-0.577997
C	-3.296250	-2.696881	-0.120088
C	-4.363488	-3.445278	-0.661269
C	-1.977635	-3.053762	-0.461350
C	-4.106423	-4.515657	-1.534421
C	-1.712376	-4.103941	-1.356926
H	-1.140454	-2.457928	-0.066887
C	-2.783272	-4.838856	-1.891582
H	-4.947491	-5.095810	-1.947345
H	-0.677077	-4.300931	-1.680640

H	-2.585254	-5.658225	-2.600315
C	-4.666001	-1.632555	1.772885
C	-4.685997	-2.808101	2.558779
C	-5.640482	-0.645222	2.022496
C	-5.641924	-2.984421	3.570699
H	-3.942265	-3.596943	2.362920
C	-6.603400	-0.821475	3.034233
C	-6.606948	-1.988790	3.814352
H	-5.635759	-3.908421	4.170740
H	-7.356317	-0.036567	3.210368
H	-7.360853	-2.127311	4.605367
C	3.454827	3.224886	0.442556
C	4.375970	2.540040	-0.386380
C	3.439495	4.636217	0.397532
C	5.238619	3.249803	-1.239870
H	4.438864	1.436240	-0.374821
C	4.300904	5.344360	-0.458693
C	5.204610	4.654199	-1.285454
H	5.939998	2.689832	-1.879031
H	4.262475	6.445193	-0.478130
H	5.878726	5.207725	-1.958092
C	1.795445	3.304628	2.389343
C	0.550473	3.946841	2.233785
C	2.481697	3.455580	3.611561
C	-0.006405	4.711743	3.272335
H	0.003045	3.844666	1.284842
C	1.933038	4.221724	4.653193
C	0.683676	4.849296	4.489782
H	-0.980533	5.203678	3.121726
H	2.480509	4.323617	5.603661
H	0.251412	5.445298	5.308989
C	1.452962	-3.683318	1.561159
C	1.483855	-3.862559	0.163541
C	1.846359	-4.749447	2.393858
C	1.886417	-5.079234	-0.404975
C	2.262692	-5.970031	1.830011
H	1.810953	-4.629772	3.488423
C	2.279982	-6.138162	0.433237
H	1.869847	-5.152240	-1.505467
H	2.564670	-6.799193	2.490401
H	2.594922	-7.100257	-0.002297
C	0.299072	-2.357920	3.447627
C	-1.106955	-2.466679	3.488177
C	1.000583	-2.256266	4.666295
C	-1.802313	-2.444784	4.707226
H	-1.660219	-2.569639	2.541926
C	0.312244	-2.256919	5.892789
C	-1.091282	-2.338792	5.917635
H	-2.902106	-2.508917	4.705521
H	0.877038	-2.175716	6.835304
H	-1.631318	-2.320515	6.877600
O	5.713987	-1.018567	2.641090

C	6.767283	-0.064554	2.587005
H	6.851329	0.398881	1.577304
H	6.636471	0.742932	3.343684
H	7.698896	-0.617942	2.810623
O	-5.777500	1.033282	-2.823802
C	-5.854322	2.090981	-3.772270
H	-6.750788	1.890162	-4.388558
H	-5.967063	3.083680	-3.279725
H	-4.958008	2.117077	-4.433768
C	0.494667	-1.639443	-2.719521
C	-0.726986	-1.322430	-2.714272
C	-2.113810	-1.277976	-3.131246
H	-2.834341	-0.972697	-2.361688
C	-2.600395	-2.294270	-4.154138
C	-2.436898	-0.850900	-4.564845
H	-1.584057	-0.602339	-5.216528
H	-3.337321	-0.229638	-4.705593
H	-1.849264	-3.017268	-4.509473
H	-3.611874	-2.701644	-4.001073
O	1.387385	-3.743256	-3.290483
C	1.705850	-2.469035	-3.220362
C	2.979654	-2.157228	-2.360631
C	3.245374	-0.968306	-1.636163
C	3.909971	-3.195682	-2.225623
N	4.285226	-0.823005	-0.797405
C	5.016329	-3.050728	-1.373776
C	5.143438	-1.849541	-0.660709
H	3.695494	-4.124492	-2.777128
H	5.748555	-3.861349	-1.239886
H	5.959633	-1.705604	0.068897
N	2.354590	0.147913	-1.698753
H	2.187013	0.454008	-2.670110
H	2.740489	0.942531	-1.174091
C	1.988995	-1.859185	-4.656421
F	2.336112	-0.511135	-4.612187
F	3.013570	-2.493218	-5.259733
F	0.912258	-1.940644	-5.473462
H	1.180181	-3.040162	-0.507201
H	2.096856	-2.151784	4.652117
H	-5.400584	-3.197369	-0.382604
H	-5.647483	0.277221	1.422385
H	2.715642	3.955334	-2.480583
H	0.828552	4.200818	-0.887278
H	-1.863003	4.844290	-2.921520
H	3.450783	2.950727	3.749321
H	2.734962	5.191586	1.035138
H	3.757589	-2.652657	2.506919
H	4.879107	1.512881	2.019604
H	-5.475730	-0.842589	-1.138362
H	-3.763671	2.892765	-2.547298
C	-2.911234	1.488357	2.853511
H	-3.524776	2.103463	2.163498

H	-3.543013	0.633245	3.177956
H	-2.670615	2.094342	3.749080
C	0.080875	1.007510	4.125393
H	-0.436815	0.292049	4.797482
H	1.166733	0.812316	4.194389
H	-0.102278	2.040578	4.487627
4C c′			
Cu2RX-OCF3ALKIPhISOMER1--2Opost SCF Done: -4144.09217892 A.U.			
Cu	0.070122	0.452007	0.965204
N	-1.455062	-0.142526	-1.457676
C	-4.063514	0.961050	-0.728435
H	-3.949114	-0.059929	-1.152032
C	-2.944263	1.824820	-1.325220
C	-1.693784	1.257449	-1.691031
C	-0.649359	-0.534519	-0.422239
C	-0.617213	2.064725	-2.144652
C	0.752640	1.466842	-2.490939
H	0.927854	0.657772	-1.746517
C	-3.103637	3.213238	-1.434982
C	-2.043728	4.031176	-1.876248
C	-0.813980	3.452589	-2.242059
C	-1.929795	-1.252999	-2.183405
C	-1.405581	-2.375444	-1.568122
N	-0.618234	-1.900671	-0.511845
C	0.346210	-2.614126	0.279746
C	0.054001	-2.905282	1.635210
C	1.630385	-2.806375	-0.267403
C	1.068517	-3.449965	2.428906
C	2.639798	-3.353063	0.549469
C	2.358968	-3.675935	1.894444
C	-1.351707	-2.608959	2.158134
H	-1.636125	-1.616826	1.739620
C	1.900570	-2.384528	-1.709983
H	1.198879	-1.553381	-1.930275
C	3.278866	-1.755694	-1.904936
C	3.573334	-0.579053	-1.180859
C	4.209157	-2.224978	-2.851449
C	4.755140	0.134527	-1.424470
H	2.848524	-0.205461	-0.436590
C	5.404133	-1.518589	-3.085630
C	5.675397	-0.332617	-2.381712
H	4.941666	1.069720	-0.877333
H	6.598602	0.233866	-2.581938
C	1.556546	-3.473964	-2.721178
C	0.874630	-3.120764	-3.904329
C	1.901079	-4.824838	-2.513407
C	0.552740	-4.092679	-4.864724
H	0.578047	-2.073169	-4.061827
C	1.572784	-5.802130	-3.469706
C	0.898643	-5.439257	-4.649623

H	0.022045	-3.792136	-5.782261
H	1.842093	-6.855225	-3.289780
H	0.639753	-6.205707	-5.397366
C	-1.435125	-2.420075	3.672758
C	-2.272162	-3.203381	4.489691
C	-0.707182	-1.357039	4.256848
C	-2.385955	-2.931941	5.866623
C	-0.819559	-1.088739	5.629253
H	-0.075198	-0.701959	3.631996
C	-1.662033	-1.873755	6.440100
H	-3.050649	-3.551106	6.490526
H	-0.253767	-0.247344	6.056126
H	-1.754809	-1.657190	7.516538
C	-2.337145	-3.622188	1.580524
C	-3.553878	-3.183096	1.023079
C	-2.054557	-5.004591	1.586878
C	-4.478402	-4.101706	0.497760
H	-3.771734	-2.105356	1.006555
C	-2.975816	-5.926983	1.062248
C	-4.193320	-5.479290	0.516894
H	-5.424386	-3.736896	0.066043
H	-2.739947	-7.003146	1.075160
H	-4.914012	-6.202135	0.102519
C	1.890825	2.464031	-2.272146
C	2.451527	2.604932	-0.987667
C	2.392965	3.261432	-3.322597
C	3.517413	3.489446	-0.760071
H	2.058577	2.002523	-0.151073
C	3.442733	4.168047	-3.093898
C	4.013951	4.276539	-1.813855
H	3.962581	3.553038	0.243937
H	3.823590	4.783473	-3.924955
H	4.844316	4.977506	-1.633631
C	0.819944	0.792913	-3.865163
C	2.051643	0.241311	-4.293417
C	-0.292527	0.688108	-4.721934
C	2.159573	-0.405602	-5.532431
H	2.936802	0.329043	-3.646471
C	-0.188268	0.032858	-5.964614
C	1.037233	-0.517412	-6.375054
H	3.126935	-0.838117	-5.831539
H	-1.072836	-0.036221	-6.618263
H	1.119744	-1.029878	-7.346471
C	-3.930216	0.773298	0.790165
C	-2.853976	1.299491	1.528172
C	-4.883252	-0.018393	1.470915
C	-2.692992	1.006424	2.895976
C	-4.737472	-0.302091	2.838978
H	-5.744510	-0.423289	0.914162
C	-3.630471	0.193458	3.552465
H	-1.796840	1.382983	3.412299
H	-5.478366	-0.941064	3.344925

H	-3.484411	-0.072971	4.610779
C	-5.445716	1.454261	-1.157954
C	-5.985557	1.040192	-2.393524
C	-6.185123	2.360098	-0.368252
C	-7.228690	1.521954	-2.835973
H	-5.421218	0.327726	-3.016806
C	-7.427700	2.847249	-0.809191
C	-7.953615	2.430928	-2.044793
H	-7.635590	1.183006	-3.802161
H	-7.990552	3.554835	-0.179684
H	-8.929505	2.809308	-2.388129
O	-2.286286	5.372122	-1.890549
C	-1.270114	6.238883	-2.389457
H	-0.338077	6.175743	-1.784250
H	-1.025646	6.013367	-3.452353
H	-1.679526	7.264213	-2.320509
O	3.274430	-4.187092	2.764449
C	4.606255	-4.382112	2.313621
H	5.171457	-4.784292	3.175606
H	4.660595	-5.111233	1.472385
H	5.076943	-3.426151	1.987952
C	2.773319	1.403631	2.530505
C	3.698630	0.639705	2.269332
C	4.762997	-0.278244	1.960264
H	4.427888	-1.172837	1.402016
C	5.925244	-0.483281	2.933037
C	6.164759	0.226715	1.625233
H	6.315076	1.318018	1.642949
H	6.700799	-0.300495	0.819982
H	5.912762	0.110226	3.860923
H	6.302187	-1.511935	3.054755
O	0.419827	1.302243	2.545793
C	1.501737	2.104803	2.854695
C	1.410691	3.505920	2.181245
C	2.469848	4.466327	2.108968
C	0.197306	3.855985	1.576924
N	2.329677	5.650402	1.468923
C	0.045851	5.083329	0.910333
C	1.157159	5.939231	0.887610
H	-0.625680	3.130295	1.632471
H	-0.906205	5.357982	0.432640
H	1.102194	6.919066	0.375867
N	3.738602	4.228235	2.603405
H	3.852411	3.546227	3.352617
H	4.322112	5.065947	2.654012
C	1.414901	2.281096	4.413962
F	1.509119	1.090994	5.052908
F	2.416241	3.072686	4.910637
F	0.244035	2.849769	4.770030
H	-2.117296	1.941895	1.022941
H	-5.781855	2.675350	0.607087
H	-2.857419	-4.022262	4.044246

H	-1.097693	-5.356477	2.004632
H	6.119149	-1.891383	-3.836634
H	3.981513	-3.131950	-3.432215
H	2.417062	-5.115209	-1.584583
H	-1.250896	1.136014	-4.420109
H	1.966243	3.156750	-4.332896
H	-4.051854	3.693630	-1.155799
H	0.030489	4.075779	-2.560263
H	0.891509	-3.667568	3.492003
H	3.646314	-3.473450	0.125797
C	-1.615550	-3.824187	-1.852804
H	-1.636496	-4.012685	-2.943947
H	-0.794810	-4.428844	-1.421972
H	-2.566416	-4.181594	-1.403974
C	-2.799992	-1.157730	-3.393571
H	-3.140933	-0.116335	-3.552008
H	-2.246459	-1.470011	-4.304939
H	-3.695335	-1.806079	-3.295946
4E cat			
Cu3RX-Cl SCF Done: -3962.04539304 A.U.			
Cu	-0.324948	-0.079059	-2.393458
N	-0.901420	-0.593664	0.350097
C	-2.773415	1.507861	0.564491
H	-1.674061	1.580042	0.433948
C	-3.191282	0.159828	-0.015158
C	-2.245003	-0.890805	-0.057129
C	0.000902	-0.083013	-0.566548
C	-2.532824	-2.145411	-0.623487
C	-1.423075	-3.197329	-0.663495
H	-0.484593	-2.652900	-0.912915
C	-4.472857	-0.084601	-0.521536
C	-4.800242	-1.350317	-1.061601
C	-3.830754	-2.376868	-1.112741
C	-0.390812	-0.521979	1.630014
C	0.864656	0.074627	1.531250
N	1.086935	0.331326	0.181294
C	2.236542	0.974907	-0.392354
C	2.106242	2.290041	-0.917743
C	3.461083	0.268531	-0.440134
C	3.245542	2.897494	-1.459251
C	4.586865	0.910762	-0.988043
C	4.484630	2.223704	-1.491565
C	0.774142	3.047342	-0.868743
H	-0.027676	2.283732	-0.986963
C	3.534975	-1.190359	0.028776
H	2.919474	-1.260666	0.952660
C	2.899273	-2.177105	-0.964646
C	2.343811	-1.784503	-2.197938
C	2.824417	-3.540778	-0.599201
C	1.687078	-2.717824	-3.024506
H	2.395163	-0.731112	-2.515530

C	2.182341	-4.475730	-1.425997
C	1.600592	-4.065157	-2.639393
H	1.208025	-2.363505	-3.950741
H	1.059008	-4.788460	-3.267979
C	4.951035	-1.593019	0.435459
C	5.392749	-1.370250	1.756116
C	5.855928	-2.139889	-0.499301
C	6.711490	-1.674776	2.134129
H	4.688708	-0.955229	2.496154
C	7.177128	-2.440414	-0.125572
C	7.610032	-2.206707	1.192131
H	7.037839	-1.500354	3.171940
H	7.871327	-2.867015	-0.867268
H	8.643672	-2.448018	1.486959
C	0.609391	3.979122	-2.070958
C	0.896603	5.357794	-2.002013
C	0.190115	3.434288	-3.302691
C	0.751166	6.176336	-3.136480
C	0.043667	4.248789	-4.436150
H	-0.031083	2.356179	-3.382641
C	0.320289	5.625978	-4.356236
H	0.974383	7.252962	-3.063999
H	-0.293111	3.797995	-5.383114
H	0.201344	6.269039	-5.242945
C	0.508561	3.717737	0.483689
C	-0.651126	4.512738	0.641044
C	1.350729	3.533994	1.598818
C	-0.952141	5.106443	1.875259
H	-1.322760	4.663924	-0.216966
C	1.045649	4.121129	2.841051
C	-0.104513	4.914673	2.982769
H	-1.870686	5.705600	1.973367
H	1.710533	3.943401	3.701200
H	-0.350333	5.369180	3.955249
C	-1.579877	-4.207835	-1.795241
C	-1.670917	-3.709126	-3.116327
C	-1.537293	-5.599231	-1.592974
C	-1.721401	-4.587629	-4.207761
H	-1.668103	-2.619221	-3.293190
C	-1.582737	-6.481809	-2.689809
C	-1.675190	-5.980442	-3.998902
H	-1.778503	-4.178127	-5.228825
H	-1.540346	-7.569157	-2.516138
H	-1.706539	-6.671593	-4.856374
C	-1.235913	-3.766637	0.742375
C	0.013984	-3.666915	1.385654
C	-2.316819	-4.334313	1.451290
C	0.188002	-4.126632	2.701494
H	0.852997	-3.206526	0.843420
C	-2.145171	-4.802455	2.764973
C	-0.891439	-4.697307	3.395964
H	1.168403	-4.019124	3.191728

H	-2.999290	-5.242102	3.304721
H	-0.761657	-5.046390	4.432355
C	-3.294997	2.698260	-0.235389
C	-2.892752	2.802064	-1.587080
C	-4.035852	3.745607	0.340935
C	-3.192105	3.948381	-2.336236
C	-4.349671	4.891715	-0.414748
H	-4.339227	3.677144	1.397132
C	-3.919190	5.001861	-1.748064
H	-2.832388	4.027574	-3.373631
H	-4.921762	5.710988	0.049757
H	-4.145873	5.908726	-2.330713
C	-2.988430	1.564293	2.075600
C	-1.940203	2.030755	2.895257
C	-4.179717	1.122694	2.686262
C	-2.072638	2.060103	4.291335
H	-0.992451	2.339628	2.431244
C	-4.315933	1.148701	4.085747
C	-3.261764	1.614675	4.892742
H	-1.227073	2.406088	4.905766
H	-5.249767	0.792242	4.549524
H	-3.363198	1.616166	5.989499
O	-6.071685	-1.479561	-1.529978
C	-6.442899	-2.695839	-2.166770
H	-5.810163	-2.905992	-3.058906
H	-6.382531	-3.564661	-1.471536
H	-7.493190	-2.567981	-2.490561
O	5.522552	2.917025	-2.033184
C	6.792464	2.283118	-2.121093
H	7.474138	3.018529	-2.588633
H	7.189689	2.005849	-1.117736
H	6.755933	1.368245	-2.755675
H	-2.312359	1.980501	-2.044906
H	-4.999796	0.735513	2.061452
H	1.223687	5.797031	-1.046384
H	2.259095	2.922528	1.496563
H	2.111576	-5.528075	-1.109136
H	3.269778	-3.867169	0.355085
H	5.511235	-2.335729	-1.527237
H	-3.304725	-4.396437	0.967004
H	-1.447700	-5.996637	-0.570276
H	-5.227960	0.714854	-0.549412
H	-4.050108	-3.348889	-1.573718
H	3.189668	3.911199	-1.880496
H	5.537907	0.364362	-1.020079
C	-0.703822	-0.819818	3.023307
C	-1.756227	-1.378432	3.733487
C	-1.643856	-1.454780	5.157557
C	-0.524998	-0.990351	5.850201
H	-2.655006	-1.749267	3.221278
H	-2.480772	-1.892323	5.723361
H	-0.485232	-1.063680	6.948890

C	1.449910	0.213534	2.863030
C	2.614697	0.726420	3.427201
C	0.450875	-0.348682	3.737026
C	2.765789	0.671370	4.849532
C	0.578683	-0.408959	5.138508
C	1.790698	0.125807	5.688931
H	3.406213	1.168914	2.802869
H	3.687994	1.078270	5.293665
H	1.949468	0.107507	6.779197
Cl	-0.792147	-0.164994	-4.467056
4E a			
Cu3RX-ALKI SCF Done: -3695.19946106 A.U.			
Cu	-1.137606	0.128369	-1.864692
N	-0.248394	-0.993149	0.619695
C	-2.343589	0.240773	2.063836
H	-1.473886	0.723233	1.573638
C	-2.581950	-1.073980	1.329115
C	-1.502831	-1.691210	0.653377
C	-0.009378	-0.055677	-0.370519
C	-1.662864	-2.863753	-0.104779
C	-0.452049	-3.423826	-0.851261
H	0.125213	-2.547374	-1.222775
C	-3.837066	-1.690493	1.274295
C	-4.013550	-2.895060	0.555189
C	-2.927829	-3.478143	-0.134129
C	0.720119	-0.928754	1.600724
C	1.589179	0.099912	1.241015
N	1.125945	0.619565	0.035753
C	1.691784	1.719154	-0.695575
C	0.992981	2.956399	-0.754919
C	2.922291	1.524355	-1.364997
C	1.581173	4.009296	-1.466977
C	3.487102	2.604734	-2.067174
C	2.824182	3.847883	-2.113814
C	-0.346671	3.164165	-0.037830
H	-0.882512	2.188941	-0.082468
C	3.575617	0.137014	-1.394422
H	3.480334	-0.288018	-0.370887
C	2.844492	-0.847160	-2.321852
C	1.747980	-0.482729	-3.127539
C	3.275113	-2.193169	-2.343520
C	1.075592	-1.443461	-3.908135
H	1.387885	0.557693	-3.135036
C	2.612808	-3.151009	-3.127058
C	1.504247	-2.780279	-3.909914
H	0.188144	-1.137167	-4.483723
H	0.961160	-3.537382	-4.495850
C	5.074026	0.212833	-1.681253
C	5.983269	0.404043	-0.620110
C	5.573306	0.152939	-2.999678

C	7.359353	0.542463	-0.868865
H	5.603070	0.441682	0.414296
C	6.948539	0.296484	-3.252019
C	7.846345	0.493617	-2.187447
H	8.056226	0.684267	-0.027276
H	7.322052	0.247553	-4.287452
H	8.925014	0.599849	-2.384278
C	-1.246693	4.146009	-0.793169
C	-1.348298	5.505824	-0.433775
C	-1.972458	3.684469	-1.911266
C	-2.168768	6.380815	-1.168737
C	-2.792310	4.555240	-2.645687
H	-1.908943	2.625714	-2.216151
C	-2.896540	5.908560	-2.275059
H	-2.240584	7.439313	-0.870807
H	-3.351983	4.162227	-3.509573
H	-3.542713	6.594189	-2.846518
C	-0.188929	3.484482	1.452398
C	-1.342762	3.765743	2.221574
C	1.061476	3.472897	2.102925
C	-1.243691	4.027155	3.595675
H	-2.327832	3.782651	1.732477
C	1.162590	3.729356	3.483214
C	0.010459	4.012445	4.234613
H	-2.160629	4.225985	4.171983
H	2.150841	3.696989	3.968930
H	0.085204	4.206074	5.316124
C	-0.830626	-4.185909	-2.116935
C	-1.613789	-3.508229	-3.081759
C	-0.358574	-5.478286	-2.407752
C	-1.916835	-4.115995	-4.308060
H	-1.961146	-2.478406	-2.880895
C	-0.658992	-6.087310	-3.642215
C	-1.437994	-5.410108	-4.595113
H	-2.517104	-3.565666	-5.049981
H	-0.275226	-7.097277	-3.858960
H	-1.669399	-5.885734	-5.561637
C	0.445164	-4.141831	0.156058
C	1.764535	-3.695922	0.371377
C	-0.048086	-5.198262	0.952710
C	2.576628	-4.287587	1.353071
H	2.145597	-2.859042	-0.231970
C	0.762969	-5.797731	1.930865
C	2.078605	-5.341236	2.137069
H	3.596279	-3.906050	1.519713
H	0.361664	-6.620318	2.544452
H	2.708250	-5.798092	2.916650
C	-3.463825	1.255931	1.852190
C	-3.715913	1.680675	0.526552
C	-4.151312	1.873271	2.912139
C	-4.607427	2.731891	0.271836
C	-5.060075	2.918019	2.655356

H	-3.945598	1.557609	3.946785
C	-5.280835	3.358253	1.338972
H	-4.752393	3.079322	-0.762781
H	-5.585321	3.402033	3.494507
H	-5.973153	4.191995	1.141963
C	-1.894822	0.007790	3.504990
C	-0.760383	0.694506	3.983642
C	-2.538319	-0.911737	4.358603
C	-0.273171	0.471540	5.280011
H	-0.229963	1.386715	3.314097
C	-2.052316	-1.139362	5.658484
C	-0.915462	-0.451798	6.121842
H	0.629309	1.005670	5.615408
H	-2.560179	-1.868091	6.310625
H	-0.524499	-0.646400	7.133020
O	-5.275355	-3.407305	0.563008
C	-5.540403	-4.571162	-0.209292
H	-5.328378	-4.408205	-1.290606
H	-4.949172	-5.447758	0.143052
H	-6.617426	-4.790232	-0.080942
O	3.300998	4.943554	-2.765781
C	4.538151	4.837604	-3.458576
H	4.727406	5.830477	-3.908705
H	5.378516	4.583507	-2.772584
H	4.496216	4.074445	-4.269092
C	-2.386119	0.344082	-3.198956
C	-3.291537	0.613726	-4.008931
C	-4.373721	0.968798	-4.895756
H	-4.122000	0.986252	-5.973069
C	-5.408865	2.009299	-4.453570
C	-5.812313	0.560152	-4.569061
H	-5.963981	-0.010619	-3.638994
H	-6.460010	0.258277	-5.408792
H	-5.280797	2.431150	-3.443511
H	-5.771749	2.724085	-5.210646
H	-3.182118	1.199188	-0.313791
H	-3.414904	-1.470326	3.994798
H	-0.788982	5.881363	0.437528
H	1.972480	3.262588	1.523732
H	2.947545	-4.199957	-3.107804
H	4.141788	-2.490490	-1.730114
H	4.870569	-0.014796	-3.831403
H	-1.085566	-5.540894	0.810424
H	0.266430	-6.007532	-1.671850
H	-4.713020	-1.222658	1.747503
H	-3.055519	-4.382477	-0.743603
H	1.075219	4.982301	-1.541175
H	4.442985	2.452578	-2.583712
C	1.135586	-1.523739	2.866491
C	0.700625	-2.538079	3.707301
C	1.433659	-2.777482	4.912519
C	2.559013	-2.033352	5.269258

H	-0.181927	-3.145253	3.460856
H	1.086044	-3.578239	5.583390
H	3.088212	-2.248406	6.211498
C	2.625030	0.250078	2.260519
C	3.725168	1.069799	2.496761
C	2.300749	-0.765392	3.230758
C	4.482309	0.866915	3.694578
C	3.032694	-0.980947	4.414655
C	4.159605	-0.119553	4.630280
H	4.011914	1.860149	1.785812
H	5.351476	1.517525	3.880457
H	4.770704	-0.238412	5.539502
4E a+b			
Cu3RX-OCF3ALKIPh--2Npre SCF Done: -4448.72415180 A.U.			
Cu	1.430717	-0.461534	0.752261
N	-1.506049	-0.434062	-0.036997
C	-1.143886	-1.601098	-2.591041
H	-0.706599	-0.657083	-2.209437
C	-1.484277	-2.464815	-1.380469
C	-1.717909	-1.852239	-0.125101
C	-0.253101	0.129988	0.153684
C	-2.160074	-2.582461	0.993549
C	-2.434116	-1.849949	2.304627
H	-1.719806	-0.996387	2.347552
C	-1.650509	-3.850361	-1.486444
C	-2.106037	-4.600163	-0.381513
C	-2.390241	-3.963244	0.841313
C	-2.416956	0.526860	-0.443930
C	-1.730701	1.736179	-0.519024
N	-0.413944	1.472063	-0.166765
C	0.670850	2.419371	-0.088399
C	1.719484	2.389172	-1.052887
C	0.689865	3.318771	1.001370
C	2.770268	3.297651	-0.897561
C	1.770744	4.211038	1.132234
C	2.815539	4.193501	0.189311
C	1.721147	1.385827	-2.213790
H	1.343179	0.428094	-1.790980
C	-0.389385	3.237539	2.086586
H	-1.353479	3.027823	1.575234
C	-0.153158	2.063598	3.044366
C	1.086164	1.402786	3.144970
C	-1.218879	1.628097	3.861415
C	1.242008	0.307037	4.017685
H	1.940075	1.716611	2.526306
C	-1.071975	0.523067	4.712657
C	0.159415	-0.157534	4.781809
H	2.227144	-0.182655	4.070438
H	0.260365	-1.042496	5.430584
C	-0.584088	4.564373	2.818880

C	-1.426550	5.550306	2.262565
C	0.093433	4.851841	4.022152
C	-1.582379	6.798546	2.887094
H	-1.968477	5.329602	1.327886
C	-0.057404	6.102193	4.647365
C	-0.894616	7.080020	4.081833
H	-2.248841	7.554506	2.441680
H	0.478481	6.311092	5.587210
H	-1.017392	8.057432	4.574988
C	3.125148	1.076824	-2.739612
C	3.729467	1.896747	-3.718369
C	3.844678	-0.029597	-2.247222
C	5.025443	1.620934	-4.185683
C	5.134146	-0.318887	-2.725306
H	3.408938	-0.656177	-1.455842
C	5.730391	0.507401	-3.692575
H	5.483723	2.275395	-4.944553
H	5.664989	-1.188601	-2.311329
H	6.745389	0.287499	-4.060437
C	0.775525	1.760521	-3.359643
C	0.721134	0.914672	-4.493036
C	-0.030691	2.914657	-3.345826
C	-0.113236	1.220187	-5.576733
H	1.348671	0.010593	-4.522452
C	-0.874918	3.220580	-4.430789
C	-0.914350	2.378131	-5.553388
H	-0.147492	0.537435	-6.440029
H	-1.507913	4.121260	-4.387156
H	-1.577241	2.611524	-6.401216
C	-2.149726	-2.651043	3.578945
C	-1.092766	-3.589901	3.630701
C	-2.847802	-2.364691	4.771012
C	-0.719278	-4.186019	4.848188
H	-0.563950	-3.880794	2.705171
C	-2.476370	-2.962520	5.987815
C	-1.402122	-3.868178	6.035455
H	0.113800	-4.907313	4.862129
H	-3.030865	-2.714668	6.907141
H	-1.107389	-4.332492	6.989832
C	-3.835384	-1.233764	2.250392
C	-4.001519	0.163467	2.307892
C	-4.981792	-2.044167	2.104669
C	-5.278165	0.745298	2.227713
H	-3.108732	0.799262	2.390546
C	-6.259722	-1.467590	2.021970
C	-6.412385	-0.069244	2.082558
H	-5.380699	1.841418	2.251533
H	-7.144023	-2.114177	1.903931
H	-7.413439	0.382759	2.003742
C	-0.028221	-2.184551	-3.452579
C	1.241427	-2.340502	-2.852873
C	-0.176043	-2.458090	-4.824783

C	2.349942	-2.727188	-3.618584
C	0.933693	-2.861261	-5.591354
H	-1.158611	-2.319507	-5.301380
C	2.200022	-2.985492	-4.994077
H	3.338545	-2.800840	-3.142550
H	0.808903	-3.062617	-6.667479
H	3.073087	-3.276269	-5.599330
C	-2.417703	-1.182489	-3.329809
C	-2.603125	0.176911	-3.651649
C	-3.439061	-2.097107	-3.661630
C	-3.773014	0.618778	-4.287337
H	-1.833132	0.906767	-3.365425
C	-4.614554	-1.658623	-4.296077
C	-4.787532	-0.297711	-4.607675
H	-3.892869	1.691088	-4.505254
H	-5.405772	-2.385833	-4.539730
H	-5.716972	0.047614	-5.087309
O	-2.203575	-5.948493	-0.580583
C	-2.597101	-6.766727	0.514996
H	-1.891271	-6.675377	1.371950
H	-3.624910	-6.521070	0.867698
H	-2.584953	-7.808928	0.143781
O	3.916866	4.994114	0.247609
C	4.063541	5.861848	1.362969
H	5.026934	6.387983	1.223434
H	3.244304	6.615412	1.417089
H	4.093379	5.298276	2.323627
C	3.245939	-0.031369	0.977698
C	4.266748	0.679234	1.079816
C	5.472453	1.462934	1.101823
H	5.650914	2.039936	2.028677
C	6.002694	2.105064	-0.185841
C	6.730400	0.929882	0.404250
H	6.622515	-0.051781	-0.083312
H	7.690798	1.092751	0.920313
H	5.420464	1.931932	-1.103472
H	6.434530	3.114470	-0.093830
O	5.247708	-2.217407	-0.161253
C	4.445495	-2.442171	0.745044
C	3.215189	-3.270069	0.501077
C	1.963909	-3.291424	1.214066
C	3.319366	-4.156888	-0.595326
N	0.980322	-4.172806	0.918556
C	2.286107	-5.040099	-0.916737
C	1.141210	-5.023271	-0.101301
H	4.252721	-4.133029	-1.177943
H	2.365040	-5.730676	-1.768299
H	0.309284	-5.724735	-0.286183
N	1.609117	-2.329691	2.142943
H	2.360459	-1.891218	2.678487
H	0.758194	-2.534530	2.676203
C	5.025794	-2.329903	2.195750

F	4.150694	-1.974835	3.192371
F	5.474088	-3.575661	2.529373
F	6.060295	-1.488522	2.254838
H	1.361484	-2.124584	-1.775650
H	-3.325675	-3.161043	-3.403363
H	3.169405	2.754900	-4.123734
H	0.006972	3.589135	-2.477867
H	-1.923030	0.181096	5.319888
H	-2.176174	2.173772	3.831316
H	0.739065	4.080794	4.471918
H	-4.867413	-3.138408	2.049590
H	-3.684870	-1.649978	4.745675
H	-1.411951	-4.372845	-2.424594
H	-2.729638	-4.539089	1.711399
H	3.610537	3.304469	-1.603017
H	1.783760	4.899379	1.986627
C	-3.805056	0.703199	-0.859644
C	-4.925984	-0.095272	-1.035159
C	-6.127824	0.521326	-1.504939
C	-6.212525	1.884260	-1.791985
H	-4.897162	-1.171920	-0.815693
H	-7.016784	-0.112451	-1.646380
H	-7.158793	2.315232	-2.156556
C	-2.639533	2.790888	-0.966443
C	-2.578718	4.155205	-1.235845
C	-3.892160	2.105826	-1.160529
C	-3.762243	4.812881	-1.700092
C	-5.065306	2.731973	-1.625550
C	-4.969944	4.137648	-1.895243
H	-1.644996	4.723407	-1.104302
H	-3.711196	5.892128	-1.914312
H	-5.854109	4.683936	-2.261492

4E a+b→c

Cu3RX-OCF3ALKIPh--2N SCF Done: -4448.72059629 A.U.

Cu	1.374011	-0.990057	0.233395
N	-1.509020	-0.099546	0.007798
C	-2.577813	-1.558330	-2.245556
H	-2.572168	-0.450876	-2.139711
C	-2.674911	-2.104334	-0.816433
C	-2.158819	-1.354858	0.272275
C	-0.136879	0.111460	0.002260
C	-2.351486	-1.755085	1.614939
C	-1.919710	-0.826046	2.754607
H	-0.943023	-0.381898	2.452000
C	-3.305078	-3.322970	-0.535479
C	-3.466577	-3.762912	0.793569
C	-3.035041	-2.957874	1.863586
C	-2.185625	1.093640	-0.199482
C	-1.228458	2.091313	-0.338105
N	0.004832	1.474723	-0.214150

C	1.288046	2.106079	-0.345613
C	1.912052	2.122087	-1.618992
C	1.899701	2.662363	0.792266
C	3.152701	2.755495	-1.737624
C	3.143585	3.308326	0.646431
C	3.768247	3.355342	-0.615226
C	1.202789	1.470105	-2.809916
H	0.745697	0.531609	-2.422160
C	1.257484	2.473526	2.163547
H	0.194199	2.231233	1.970219
C	1.820388	1.270989	2.928408
C	3.058370	0.680740	2.613518
C	1.067705	0.727372	3.994176
C	3.544938	-0.414531	3.349717
H	3.635510	1.061113	1.761055
C	1.540271	-0.374807	4.723088
C	2.788111	-0.946084	4.406704
H	4.500197	-0.877617	3.060226
H	3.157336	-1.816485	4.971611
C	1.249030	3.767532	2.973224
C	0.240934	4.723497	2.721058
C	2.239032	4.057460	3.933391
C	0.227061	5.947930	3.407742
H	-0.537093	4.501163	1.970971
C	2.229144	5.285721	4.618952
C	1.224768	6.234189	4.358444
H	-0.569029	6.681357	3.202051
H	3.008028	5.499389	5.368481
H	1.214276	7.193078	4.900563
C	2.177918	1.016686	-3.894909
C	2.216945	1.564223	-5.190303
C	3.069329	-0.028814	-3.568580
C	3.138949	1.081263	-6.139842
C	3.994081	-0.504413	-4.509344
H	3.022864	-0.484235	-2.566153
C	4.032660	0.050724	-5.802375
H	3.154593	1.516061	-7.152301
H	4.668911	-1.328793	-4.228712
H	4.752029	-0.325156	-6.547484
C	0.057317	2.366523	-3.280833
C	-1.233663	1.827218	-3.444423
C	0.244165	3.745465	-3.514504
C	-2.320887	2.639463	-3.804607
H	-1.378229	0.753206	-3.266267
C	-0.835678	4.559046	-3.900469
C	-2.124134	4.011850	-4.035552
H	-3.325173	2.195890	-3.894175
H	-0.674001	5.635115	-4.073062
H	-2.974887	4.657130	-4.304296
C	-1.645042	-1.504877	4.102797
C	-0.994700	-2.760506	4.167185
C	-1.927791	-0.836582	5.313274

C	-0.628914	-3.317408	5.404586
H	-0.783612	-3.323026	3.238369
C	-1.562224	-1.395463	6.551201
C	-0.907464	-2.638277	6.604001
H	-0.118941	-4.294057	5.425442
H	-1.789484	-0.850027	7.481048
H	-0.618711	-3.074890	7.573164
C	-2.920068	0.331111	2.863766
C	-2.489396	1.670187	2.804409
C	-4.300008	0.078551	3.015312
C	-3.404478	2.735065	2.865996
H	-1.419286	1.884611	2.673474
C	-5.218698	1.137883	3.094628
C	-4.775771	2.470782	3.011628
H	-3.039245	3.770598	2.785478
H	-6.293328	0.920885	3.202777
H	-5.499838	3.299378	3.043592
C	-1.275934	-1.899624	-2.981756
C	-0.157694	-2.466868	-2.340453
C	-1.168094	-1.547999	-4.348238
C	1.043043	-2.678757	-3.045012
C	0.021752	-1.768883	-5.056547
H	-2.033956	-1.100438	-4.861770
C	1.127229	-2.341199	-4.405013
H	1.928244	-3.090224	-2.533296
H	0.089597	-1.482285	-6.117762
H	2.067849	-2.502766	-4.951280
C	-3.827778	-1.879337	-3.062714
C	-4.885668	-0.949064	-3.121923
C	-3.961575	-3.100864	-3.757763
C	-6.060199	-1.237827	-3.838527
H	-4.782372	0.017761	-2.602440
C	-5.135916	-3.394946	-4.469892
C	-6.191847	-2.465878	-4.509752
H	-6.873281	-0.495199	-3.877013
H	-5.224249	-4.353695	-5.005539
H	-7.110206	-2.694684	-5.073510
O	-4.051307	-4.986923	0.947066
C	-4.216516	-5.495657	2.265395
H	-3.241603	-5.590729	2.796172
H	-4.896232	-4.857127	2.874967
H	-4.669625	-6.499107	2.156148
O	4.979886	3.938843	-0.847712
C	5.679703	4.502680	0.253612
H	6.627173	4.904596	-0.152504
H	5.108007	5.331833	0.729662
H	5.912291	3.737012	1.029304
C	3.197482	-1.505892	-0.174441
C	4.186308	-0.727842	-0.212963
C	5.380395	0.057554	-0.246605
H	5.657358	0.547637	0.706723
C	5.844112	0.780783	-1.518054

C	6.565186	-0.458580	-1.087956
H	6.379372	-1.395234	-1.636494
H	7.563192	-0.377115	-0.627959
H	5.179787	0.720836	-2.392173
H	6.309373	1.767237	-1.366841
O	3.830364	-3.672606	-1.538766
C	3.421082	-3.440982	-0.367722
C	2.062448	-4.051317	0.042688
C	1.179428	-3.618904	1.077717
C	1.618991	-5.129939	-0.742167
N	-0.031870	-4.173591	1.292255
C	0.366697	-5.720209	-0.515128
C	-0.426584	-5.194274	0.515110
H	2.280037	-5.461189	-1.557941
H	0.006743	-6.557176	-1.131746
H	-1.433307	-5.599327	0.720123
N	1.452328	-2.478906	1.854502
H	2.426703	-2.371848	2.157255
H	0.798347	-2.354625	2.635071
C	4.519606	-3.672596	0.728708
F	4.195724	-3.210749	1.992633
F	4.716586	-5.008466	0.868556
F	5.700133	-3.117205	0.404940
H	-0.220257	-2.749556	-1.277844
H	-3.123041	-3.815533	-3.754457
H	1.514551	2.365219	-5.467527
H	1.244188	4.185968	-3.372946
H	0.927466	-0.790729	5.537004
H	0.097309	1.174829	4.263522
H	3.014078	3.305353	4.152859
H	-4.655073	-0.963440	3.056632
H	-2.432536	0.140984	5.287879
H	-3.705778	-3.953184	-1.340751
H	-3.192664	-3.270321	2.902820
H	3.676397	2.778413	-2.703885
H	3.608159	3.753689	1.536234
C	-3.524028	1.663375	-0.297236
C	-4.836127	1.212367	-0.223896
C	-5.891536	2.170441	-0.360791
C	-5.650862	3.532240	-0.551312
H	-5.064983	0.150250	-0.053610
H	-6.931618	1.812925	-0.303837
H	-6.494942	4.234111	-0.646921
C	-1.878856	3.385915	-0.512206
C	-1.495482	4.712791	-0.664919
C	-3.282442	3.068808	-0.491082
C	-2.519721	5.705171	-0.794429
C	-4.304926	4.027982	-0.616185
C	-3.879594	5.389562	-0.776596
H	-0.434095	4.999199	-0.697020
H	-2.215807	6.756346	-0.919537
H	-4.632857	6.187203	-0.879967

4E a+b→c'			
Cu3RX-OCF3ALKIPhISOMER1--2O SCF Done: -4448.70691074 A.U.			
Cu	1.096213	-1.051421	0.961359
N	-0.149263	1.271456	-0.482034
C	0.267403	3.895833	0.773133
H	-0.661234	3.889275	0.162648
C	1.337887	3.200233	-0.088102
C	1.091885	1.954751	-0.731050
C	-0.250209	0.055075	0.178375
C	2.023266	1.374996	-1.625998
C	1.719483	0.074814	-2.381862
H	1.118627	-0.561162	-1.695965
C	2.563576	3.830644	-0.339536
C	3.509613	3.266589	-1.218814
C	3.228624	2.054304	-1.872527
C	-1.400507	1.696224	-0.912195
C	-2.318867	0.733858	-0.504513
N	-1.600896	-0.259349	0.131077
C	-2.101366	-1.562508	0.460967
C	-2.461535	-1.852766	1.794667
C	-2.170405	-2.524351	-0.565018
C	-2.888135	-3.149160	2.100612
C	-2.596394	-3.827168	-0.235652
C	-2.947926	-4.139783	1.095377
C	-2.438759	-0.723808	2.820552
H	-1.637077	-0.021154	2.508631
C	-1.779441	-2.142811	-1.990272
H	-1.119346	-1.255185	-1.915332
C	-0.897721	-3.198301	-2.653702
C	0.369162	-3.442548	-2.076848
C	-1.245026	-3.859799	-3.844947
C	1.278136	-4.318499	-2.685030
H	0.656507	-2.907211	-1.155550
C	-0.336160	-4.746903	-4.453979
C	0.926643	-4.975050	-3.880521
H	2.272818	-4.464106	-2.236969
H	1.642208	-5.655601	-4.368441
C	-2.989148	-1.674027	-2.797712
C	-2.902601	-0.460572	-3.509123
C	-4.206527	-2.385692	-2.823084
C	-3.998619	0.033823	-4.231776
H	-1.974089	0.126107	-3.460462
C	-5.307894	-1.893349	-3.545033
C	-5.207876	-0.680650	-4.250631
H	-3.906883	0.996956	-4.757008
H	-6.254723	-2.456912	-3.547452
H	-6.076753	-0.286790	-4.801079
C	-2.019004	-1.193355	4.208880
C	-2.810869	-1.045183	5.361342
C	-0.724958	-1.749068	4.335190

C	-2.320516	-1.448974	6.619195
C	-0.233789	-2.146173	5.585846
H	-0.090579	-1.852174	3.438022
C	-1.033421	-1.998902	6.736406
H	-2.950930	-1.323862	7.514230
H	0.785309	-2.557335	5.662245
H	-0.649284	-2.306701	7.721920
C	-3.753622	0.047309	2.702659
C	-3.730909	1.407649	2.338030
C	-5.005257	-0.586590	2.867093
C	-4.923136	2.127345	2.155719
H	-2.763686	1.903692	2.178021
C	-6.200342	0.130407	2.687357
C	-6.163113	1.491622	2.330052
H	-4.877028	3.185236	1.853184
H	-7.168144	-0.379391	2.820272
H	-7.100206	2.049835	2.176636
C	2.975514	-0.740090	-2.693648
C	3.431901	-1.706432	-1.776461
C	3.707571	-0.539509	-3.884437
C	4.582084	-2.465291	-2.043267
H	2.884770	-1.870795	-0.834481
C	4.871804	-1.282412	-4.144532
C	5.312056	-2.250219	-3.223723
H	4.918177	-3.214383	-1.310907
H	5.433173	-1.110198	-5.077025
H	6.223548	-2.835283	-3.423758
C	0.848328	0.295251	-3.626526
C	0.539745	-0.812437	-4.451684
C	0.328161	1.556224	-3.978547
C	-0.266751	-0.658887	-5.588442
H	0.943083	-1.804181	-4.197498
C	-0.486884	1.711970	-5.115717
C	-0.784412	0.605769	-5.927878
H	-0.504790	-1.540965	-6.203240
H	-0.894997	2.706621	-5.356040
H	-1.428214	0.722541	-6.813610
C	-0.113799	3.217649	2.095969
C	0.549471	2.094656	2.625463
C	-1.176367	3.791815	2.833411
C	0.131934	1.544831	3.854214
C	-1.576852	3.253455	4.065342
H	-1.689839	4.681043	2.432137
C	-0.929463	2.113363	4.575172
H	0.645017	0.657850	4.246159
H	-2.414936	3.709729	4.615389
H	-1.254987	1.658558	5.523500
C	0.591485	5.373078	1.003984
C	0.096032	6.356056	0.123984
C	1.409413	5.776607	2.081193
C	0.414863	7.713055	0.307255
H	-0.553389	6.051056	-0.713063

C	1.735110	7.130297	2.263598
C	1.239284	8.103694	1.376526
H	0.013529	8.469056	-0.386617
H	2.377715	7.428661	3.107283
H	1.490599	9.166193	1.523132
O	4.665932	3.970442	-1.376296
C	5.612623	3.509532	-2.331338
H	5.997676	2.495648	-2.081950
H	5.182121	3.481482	-3.358647
H	6.453022	4.228649	-2.308549
O	-3.300084	-5.387372	1.520648
C	-3.270053	-6.455680	0.582366
H	-3.559395	-7.366661	1.139316
H	-3.990978	-6.296599	-0.251841
H	-2.251539	-6.598405	0.153927
C	2.514184	-2.366547	1.674502
C	1.438762	-3.018108	1.488967
C	0.463854	-4.098457	1.460240
H	-0.338871	-3.979997	0.718375
C	0.073609	-4.822789	2.745598
C	0.924109	-5.530540	1.719690
H	1.992911	-5.679971	1.940972
H	0.467155	-6.322322	1.103501
H	0.553879	-4.481516	3.675562
H	-0.982931	-5.114993	2.850448
O	2.925773	0.385592	1.144454
C	3.762130	-0.388321	1.669326
C	5.016648	-0.745535	0.946932
C	5.752671	-1.982842	1.034657
C	5.492517	0.218356	0.033846
N	6.910846	-2.178723	0.349389
C	6.681461	0.006427	-0.670307
C	7.358644	-1.208611	-0.450146
H	4.903896	1.139115	-0.087776
H	7.085872	0.755808	-1.366393
H	8.315817	-1.411544	-0.967015
N	5.300023	-3.038631	1.760321
H	4.287168	-3.020837	1.999889
H	5.778348	-3.925285	1.590445
C	3.804038	-0.374726	3.229591
F	2.565436	-0.398688	3.780339
F	4.526978	-1.358724	3.799517
F	4.376389	0.809197	3.587921
H	1.399083	1.638445	2.092637
H	1.787916	5.015043	2.781444
H	-3.812149	-0.594870	5.280479
H	-5.042382	-1.656622	3.125808
H	-0.614447	-5.251941	-5.393011
H	-2.219547	-3.653237	-4.313774
H	-4.303534	-3.324258	-2.255948
H	0.568987	2.431239	-3.357946
H	3.347980	0.195840	-4.622334

C	-3.663065	1.087571	-0.949721
C	-3.466932	2.318240	-1.665080
C	-4.941912	0.557285	-0.854834
C	-2.088386	2.736033	-1.679163
C	-4.507605	3.023279	-2.301432
C	-6.013401	1.262502	-1.487084
H	-5.134514	-0.374794	-0.304430
C	-1.755113	3.901347	-2.364490
C	-4.131730	4.220340	-2.996194
C	-5.820083	2.451937	-2.190606
H	-7.027877	0.841265	-1.414728
C	-2.796576	4.631856	-3.019531
H	-0.715473	4.260695	-2.409925
H	-4.901286	4.814640	-3.514671
H	-6.674711	2.956695	-2.668965
H	-2.528456	5.553053	-3.560510
H	2.809060	4.793156	0.127916
H	3.953773	1.599223	-2.558294
H	-3.134529	-3.426848	3.135907
H	-2.586847	-4.592880	-1.023337
4E c′			
Cu3RX-OCF3ALKIPhISOMER1--2Opost SCF Done: -4448.73582007 A.U.			
Cu	-1.347277	-0.469988	-0.792681
N	0.579149	1.294952	0.527137
C	0.622701	3.902577	-0.785114
H	1.581740	3.657767	-0.280557
C	-0.500206	3.485545	0.176865
C	-0.471391	2.227954	0.839586
C	0.325405	0.101832	-0.132836
C	-1.478009	1.843493	1.760223
C	-1.383458	0.532671	2.553818
H	-1.008770	-0.239394	1.846188
C	-1.582679	4.336491	0.429919
C	-2.619358	3.953390	1.305202
C	-2.553448	2.720686	1.980153
C	1.932075	1.405865	0.822579
C	2.549559	0.256604	0.332330
N	1.555824	-0.529631	-0.220313
C	1.657567	-1.914223	-0.583336
C	1.744015	-2.274563	-1.945967
C	1.565764	-2.874946	0.442704
C	1.723013	-3.634542	-2.277278
C	1.564590	-4.239747	0.089898
C	1.626160	-4.617715	-1.268291
C	1.925642	-1.176094	-2.990065
H	1.400875	-0.273296	-2.609799
C	1.440850	-2.424486	1.897307
H	0.985082	-1.413973	1.881014
C	0.435477	-3.274240	2.670815
C	-0.918891	-3.190420	2.276503

C	0.776644	-4.083590	3.769012
C	-1.915485	-3.893147	2.966298
H	-1.189357	-2.536568	1.430444
C	-0.222169	-4.796552	4.461173
C	-1.567208	-4.702956	4.064976
H	-2.967109	-3.784236	2.660008
H	-2.347212	-5.250050	4.617776
C	2.807930	-2.243404	2.553114
C	3.077448	-1.051226	3.254418
C	3.827187	-3.212519	2.449203
C	4.330211	-0.828807	3.844972
H	2.306471	-0.269000	3.302996
C	5.084394	-2.993129	3.038961
C	5.339929	-1.799531	3.738263
H	4.517841	0.122115	4.366949
H	5.873665	-3.755851	2.941977
H	6.331084	-1.620112	4.183731
C	1.232449	-1.498014	-4.310129
C	1.889050	-1.536507	-5.552934
C	-0.167644	-1.690772	-4.272353
C	1.160225	-1.765493	-6.737007
C	-0.895747	-1.910959	-5.448670
H	-0.692528	-1.638273	-3.303782
C	-0.231242	-1.951385	-6.690303
H	1.687817	-1.788635	-7.704109
H	-1.989433	-2.026183	-5.394117
H	-0.799886	-2.119458	-7.618713
C	3.413095	-0.824380	-3.044597
C	3.839916	0.472545	-2.697498
C	4.390897	-1.795485	-3.356066
C	5.205317	0.802657	-2.677502
H	3.089163	1.226850	-2.422222
C	5.756921	-1.468126	-3.341014
C	6.169627	-0.165542	-3.001636
H	5.514004	1.817975	-2.383043
H	6.505571	-2.237658	-3.589118
H	7.241334	0.087937	-2.977964
C	-2.741637	0.022411	3.032842
C	-3.490485	-0.854620	2.224495
C	-3.276370	0.416441	4.278808
C	-4.728829	-1.353258	2.658243
H	-3.099029	-1.151526	1.239559
C	-4.526853	-0.061367	4.706095
C	-5.253422	-0.954765	3.898956
H	-5.294416	-2.041794	2.013788
H	-4.929989	0.257574	5.680843
H	-6.230511	-1.338125	4.233137
C	-0.358122	0.581517	3.697355
C	-0.216960	-0.551523	4.534101
C	0.457544	1.701596	3.949017
C	0.707714	-0.557329	5.588079
H	-0.845712	-1.436609	4.355128

C	1.393050	1.695731	5.001416
C	1.517885	0.568767	5.829442
H	0.805669	-1.456773	6.215611
H	2.029789	2.579853	5.163869
H	2.251946	0.559380	6.650255
C	0.636450	3.137055	-2.113471
C	-0.418033	2.301163	-2.526910
C	1.741473	3.319081	-2.975514
C	-0.350584	1.642301	-3.769185
C	1.800001	2.671131	-4.220066
H	2.562246	3.986548	-2.663955
C	0.754020	1.818661	-4.617686
H	-1.174604	0.981417	-4.065452
H	2.677104	2.815309	-4.870547
H	0.802086	1.280529	-5.576978
C	0.653718	5.413808	-1.018585
C	1.415713	6.246155	-0.173605
C	-0.097318	6.007220	-2.055547
C	1.423183	7.640370	-0.351321
H	2.017347	5.791399	0.630403
C	-0.096444	7.400693	-2.232916
C	0.663056	8.222771	-1.380748
H	2.029630	8.274634	0.315058
H	-0.690614	7.847738	-3.045914
H	0.667441	9.315159	-1.523144
O	-3.639725	4.845241	1.439760
C	-4.706223	4.523783	2.323008
H	-5.217407	3.580536	2.027489
H	-4.356766	4.421837	3.376209
H	-5.426257	5.361345	2.259588
O	1.542221	-5.907607	-1.707324
C	1.347618	-6.937953	-0.746428
H	1.287283	-7.885207	-1.314716
H	2.195180	-7.000852	-0.026338
H	0.401791	-6.795368	-0.174772
C	-3.061162	-1.416048	-1.410619
C	-2.209605	-2.317127	-1.186589
C	-1.685326	-3.667125	-1.121660
H	-0.769443	-3.786322	-0.526834
C	-1.808813	-4.580276	-2.339775
C	-2.663776	-4.838913	-1.124778
H	-3.733085	-4.574467	-1.163340
H	-2.418992	-5.691769	-0.470916
H	-2.280374	-4.153717	-3.239289
H	-0.947894	-5.239311	-2.537356
O	-2.875317	0.857962	-1.156849
C	-3.792941	-0.104717	-1.517067
C	-5.071363	-0.085310	-0.645190
C	-5.988436	-1.171315	-0.497762
C	-5.324339	1.083920	0.082748
N	-7.066006	-1.098402	0.316164
C	-6.455543	1.165590	0.911156

C	-7.285640	0.037954	0.993914
H	-4.589087	1.899604	-0.004157
H	-6.684494	2.071206	1.492641
H	-8.176773	0.045449	1.648715
N	-5.799590	-2.407236	-1.105980
H	-5.369391	-2.403397	-2.032096
H	-6.630032	-3.001572	-1.032644
C	-4.205505	0.109686	-3.014806
F	-3.141153	-0.017222	-3.851784
F	-5.130383	-0.812040	-3.446381
F	-4.736425	1.328961	-3.197801
H	-1.307421	2.138574	-1.895438
H	-0.682940	5.362402	-2.729821
H	2.975490	-1.366186	-5.601489
H	4.076945	-2.822473	-3.601118
H	0.055288	-5.420972	5.325715
H	1.825654	-4.138006	4.099216
H	3.642796	-4.139125	1.883351
H	0.357375	2.594593	3.315242
H	-2.695285	1.093253	4.925515
C	3.979419	0.282548	0.621968
C	4.157988	1.518999	1.332464
C	5.079977	-0.532062	0.396542
C	2.925607	2.248113	1.489495
C	5.398608	1.954800	1.837893
C	6.350276	-0.104573	0.896614
H	4.985127	-1.478144	-0.155778
C	2.953105	3.455393	2.182065
C	5.393156	3.202842	2.544804
C	6.519975	1.091102	1.595838
H	7.223221	-0.752206	0.722053
C	4.202699	3.916746	2.704655
H	2.038403	4.048747	2.333498
H	6.332985	3.596608	2.964242
H	7.516477	1.376979	1.969014
H	4.219708	4.872431	3.251962
H	-1.653557	5.317264	-0.059302
H	-3.344364	2.411002	2.673972
H	1.736285	-3.954615	-3.329340
H	1.440668	-4.982254	0.889772

4G cat

Cu4 SCF Done: -4109.55330816 A.U.

Cu	-0.018719	1.162596	-2.055682
Cl	0.035527	1.775097	-4.084977
N	1.133001	0.321272	0.480761
C	1.650671	-1.989577	-1.372847
H	0.624800	-1.733741	-1.055078
C	2.690397	-0.987315	-0.874442
C	2.450372	0.123285	-0.047162
C	0.009340	0.629157	-0.278306
C	3.523487	0.929809	0.358400

C	3.450845	2.174549	1.245748
H	2.418241	2.421020	1.554023
C	3.999463	-1.290660	-1.297295
C	5.083787	-0.485019	-0.898237
C	4.833790	0.619588	-0.060737
C	0.771806	-0.084895	1.756048
C	-0.615049	-0.020244	1.822537
N	-1.059419	0.407964	0.581373
C	-2.436585	0.365121	0.186575
C	-3.055890	-0.889983	0.043245
C	-3.178727	1.534718	-0.028208
C	-4.432337	-0.967815	-0.245106
C	-4.551710	1.450713	-0.344919
C	-5.194978	0.202025	-0.430632
C	-2.381361	-2.264010	0.134137
H	-1.300706	-2.201317	0.357095
C	-2.657265	2.970692	0.012619
H	-1.581825	3.027953	0.260665
C	-2.974441	3.531606	-1.378247
C	-2.046886	4.014654	-2.305285
C	-4.349385	3.456528	-1.703256
C	-2.502100	4.443989	-3.568359
H	-0.971694	4.019599	-2.069398
C	-4.803726	3.894100	-2.951887
C	-3.869375	4.393647	-3.884900
H	-1.771279	4.789739	-4.314634
H	-5.874126	3.832749	-3.206442
H	-4.215401	4.727213	-4.876087
C	-3.555169	3.712843	1.005431
C	-3.139998	4.413060	2.143614
C	-4.931021	3.631090	0.677596
C	-4.105607	5.037347	2.961765
H	-2.068879	4.473521	2.395670
C	-5.888980	4.249487	1.489502
C	-5.470357	4.955769	2.637476
H	-3.786041	5.591143	3.858784
H	-6.958329	4.186493	1.229378
H	-6.218251	5.446449	3.280435
C	-2.681860	-2.951316	-1.203778
C	-4.067739	-3.026260	-1.483502
C	-1.747678	-3.464420	-2.110238
C	-4.517993	-3.603985	-2.675611
C	-2.200679	-4.040768	-3.315240
H	-0.670776	-3.423935	-1.895015
C	-3.575178	-4.109347	-3.596219
H	-5.596852	-3.654540	-2.893567
H	-1.465230	-4.428461	-4.037112
H	-3.921457	-4.557251	-4.541025
C	-3.159050	-3.042028	1.196716
C	-2.628190	-3.637382	2.345999
C	-4.546821	-3.112006	0.921687
C	-3.494613	-4.305356	3.236574

H	-1.550325	-3.562914	2.561459
C	-5.405693	-3.774005	1.806967
C	-4.872740	-4.371247	2.970180
H	-3.085924	-4.768546	4.148210
H	-6.485551	-3.830063	1.593530
H	-5.543059	-4.894160	3.670748
C	4.122278	3.292563	0.439761
C	3.551454	4.517714	0.078380
C	5.444785	2.973562	0.047458
C	4.311377	5.434234	-0.678632
H	2.518581	4.758493	0.376851
C	6.199430	3.883404	-0.701500
C	5.625622	5.120385	-1.064039
H	3.868329	6.398866	-0.971936
H	7.228775	3.632147	-1.004851
H	6.210787	5.841469	-1.656297
C	4.377191	1.878121	2.427885
C	4.025868	1.880200	3.782430
C	5.692625	1.541478	2.025063
C	4.996431	1.533985	4.745903
H	2.997398	2.129810	4.086976
C	6.655358	1.198171	2.981598
C	6.300481	1.193590	4.347782
H	4.726003	1.525030	5.813460
H	7.676922	0.929919	2.667744
H	7.052624	0.921706	5.105294
C	1.801188	-2.003619	-2.897564
C	3.113733	-2.323583	-3.318511
C	0.807960	-1.688624	-3.830232
C	3.430240	-2.350591	-4.681239
C	1.129225	-1.706990	-5.202696
H	-0.199559	-1.396715	-3.497066
C	2.426212	-2.042054	-5.625058
H	4.452561	-2.600060	-5.009403
H	0.359975	-1.429732	-5.939060
H	2.668247	-2.049490	-6.699719
C	2.126271	-3.350409	-0.864058
C	1.408202	-4.236235	-0.052179
C	3.440920	-3.665157	-1.286876
C	2.005031	-5.455763	0.331419
H	0.389100	-3.984978	0.282965
C	4.033189	-4.873693	-0.903563
C	3.306662	-5.772150	-0.092844
H	1.445877	-6.160760	0.966523
H	5.056691	-5.117408	-1.231048
H	3.765836	-6.726336	0.210665
O	6.367406	-0.773114	-1.312131
C	6.697058	-0.168758	-2.569972
H	7.733590	-0.472885	-2.814099
H	6.010273	-0.510173	-3.378882
H	6.640757	0.943191	-2.512368
O	-6.543813	0.118871	-0.710501

C	-7.366647	0.198462	0.457152
H	-7.211620	1.158308	1.003625
H	-8.419454	0.136295	0.118803
H	-7.155806	-0.641188	1.160625
C	-1.081341	-0.545249	3.103910
C	-2.298578	-0.777649	3.735609
C	0.133893	-0.927280	3.774943
C	-2.285469	-1.382001	5.032741
H	-3.252997	-0.530033	3.247219
C	0.167435	-1.526516	5.049777
C	-1.103413	-1.747592	5.679862
H	-3.250138	-1.577364	5.525818
H	-1.142227	-2.216942	6.676071
C	1.308571	-0.668245	2.983501
C	2.554577	-1.018212	3.494683
C	2.609980	-1.614291	4.795014
H	3.479361	-0.840078	2.926431
C	1.466982	-1.866563	5.556748
H	3.597701	-1.882169	5.201186
H	1.557935	-2.334913	6.550053
C	-5.193692	2.817952	-0.592175
C	5.883565	1.578970	0.503731
C	4.073357	-2.563202	-2.146868
C	-4.936181	-2.405477	-0.381310
H	-6.264297	2.737360	-0.853345
H	-6.017221	-2.449759	-0.604475
H	5.106480	-2.799769	-2.458859
H	6.907661	1.310413	0.188343

4G a

Cu4RX-ALKI SCF Done: -3842.71523171 A.U.

Cu	0.998885	1.156769	0.673601
N	0.739518	-1.252779	-0.742655
C	2.840474	-0.214068	-2.610737
H	1.821127	-0.419385	-2.986849
C	3.101241	-0.820938	-1.231013
C	2.125595	-1.363636	-0.382818
C	0.014992	-0.183376	-0.227353
C	2.460057	-1.706925	0.938740
C	1.477273	-2.113696	2.041449
H	0.452050	-2.280716	1.662717
C	4.395249	-0.561072	-0.739341
C	4.729478	-0.854930	0.597048
C	3.761613	-1.465006	1.419839
C	-0.052758	-2.059001	-1.537578
C	-1.329937	-1.500564	-1.513188
N	-1.270492	-0.373514	-0.704411
C	-2.416341	0.380054	-0.293781
C	-2.574808	1.731059	-0.645444
C	-3.407680	-0.262291	0.473191
C	-3.749633	2.420015	-0.273192

C	-4.560410	0.444316	0.869629
C	-4.744143	1.789821	0.497925
C	-1.584612	2.611839	-1.411747
H	-0.646296	2.084996	-1.660203
C	-3.352243	-1.685339	1.047585
H	-2.446725	-2.243686	0.747308
C	-3.462622	-1.509090	2.566355
C	-2.553569	-1.966853	3.526168
C	-4.622696	-0.795877	2.956042
C	-2.793884	-1.691032	4.889223
H	-1.655546	-2.528485	3.222746
C	-4.864764	-0.528499	4.307855
C	-3.939789	-0.976474	5.276279
H	-2.073553	-2.033912	5.647984
H	-5.767875	0.026449	4.609959
H	-4.119735	-0.764369	6.342041
C	-4.646877	-2.373849	0.609604
C	-4.753242	-3.579465	-0.093338
C	-5.806985	-1.656761	0.988522
C	-6.032376	-4.065857	-0.434382
H	-3.846815	-4.128709	-0.391638
C	-7.076487	-2.138834	0.648282
C	-7.184951	-3.349262	-0.069148
H	-6.124235	-5.009623	-0.994594
H	-7.978653	-1.577113	0.939152
H	-8.180462	-3.734550	-0.341157
C	-1.343807	3.834591	-0.513878
C	-2.534729	4.517500	-0.171822
C	-0.108397	4.272041	-0.024967
C	-2.493723	5.642396	0.660240
C	-0.067637	5.398344	0.822918
H	0.818962	3.726332	-0.255488
C	-1.247910	6.082016	1.159600
H	-3.423683	6.174123	0.920942
H	0.903289	5.721449	1.229785
H	-1.205914	6.961843	1.821575
C	-2.354113	3.120182	-2.631692
C	-1.986685	2.969823	-3.973499
C	-3.549916	3.795616	-2.285593
C	-2.821023	3.500108	-4.980096
H	-1.055288	2.443224	-4.237559
C	-4.378283	4.320000	-3.284134
C	-4.008264	4.169502	-4.637613
H	-2.538910	3.387765	-6.038920
H	-5.308920	4.844186	-3.013080
H	-4.654846	4.580235	-5.429245
C	1.550488	-0.964559	3.057703
C	0.498037	-0.123548	3.439944
C	2.859703	-0.744829	3.544378
C	0.756323	0.936297	4.334304
H	-0.512975	-0.277227	3.034417
C	3.114323	0.307475	4.430177

C	2.053688	1.149629	4.824837
H	-0.062807	1.611813	4.624554
H	4.136713	0.481274	4.803154
H	2.251246	1.991079	5.507412
C	2.078049	-3.341205	2.720795
C	1.460952	-4.585939	2.892156
C	3.391536	-3.117551	3.199788
C	2.161771	-5.618075	3.550500
H	0.438740	-4.754452	2.515189
C	4.087205	-4.143067	3.851035
C	3.465904	-5.397629	4.025886
H	1.683683	-6.600445	3.691497
H	5.110287	-3.968504	4.221286
H	4.007823	-6.208503	4.538090
C	3.115010	1.282199	-2.417646
C	4.416793	1.544414	-1.930193
C	2.209174	2.326908	-2.624623
C	4.806876	2.855704	-1.640786
C	2.603872	3.649891	-2.338035
H	1.196217	2.114694	-3.001397
C	3.891653	3.909969	-1.843293
H	5.815982	3.059829	-1.247456
H	1.891366	4.475953	-2.485456
H	4.187088	4.942762	-1.601146
C	3.936766	-0.756926	-3.524691
C	3.751426	-1.462717	-4.719265
C	5.240188	-0.491695	-3.039481
C	4.880873	-1.900628	-5.442169
H	2.733330	-1.672293	-5.085799
C	6.360344	-0.926775	-3.757883
C	6.175733	-1.632430	-4.965454
H	4.745900	-2.454825	-6.384628
H	7.374423	-0.721494	-3.378716
H	7.052942	-1.977263	-5.535670
O	5.945862	-0.472878	1.116621
C	5.915442	0.859981	1.663075
H	5.116385	0.969044	2.427540
H	6.908445	1.043686	2.116752
H	5.710090	1.617793	0.875667
O	-5.866556	2.479954	0.908146
C	-5.659850	3.209469	2.124671
H	-6.612094	3.723500	2.360275
H	-5.389438	2.527390	2.964243
H	-4.847966	3.965779	2.016968
C	-2.240859	-2.287260	-2.339889
C	-3.583088	-2.255939	-2.702838
C	-1.407941	-3.345168	-2.849387
C	-4.077259	-3.283137	-3.569046
H	-4.260969	-1.476926	-2.323744
C	-1.873482	-4.359910	-3.708799
C	-3.264412	-4.304696	-4.062711
H	-5.143247	-3.262960	-3.843571

H	-3.687320	-5.074735	-4.727766
C	-0.046223	-3.246700	-2.390908
C	0.876178	-4.194005	-2.825723
C	0.422804	-5.232197	-3.701364
H	1.930642	-4.153125	-2.512315
C	-0.902108	-5.327016	-4.133413
H	1.152875	-5.982350	-4.043701
H	-1.205799	-6.144594	-4.806807
C	-5.492171	-0.374043	1.764642
C	3.894336	-1.699291	2.928178
C	5.242356	0.265804	-1.711153
C	-3.784170	3.865805	-0.772276
H	-6.395966	0.191262	2.054752
H	-4.725651	4.378484	-0.504653
H	6.257434	0.459015	-1.320175
H	4.924249	-1.524138	3.287659
C	2.321169	2.299722	1.262697
C	3.242969	3.057268	1.610302
C	4.315662	3.922361	2.036730
H	4.997277	4.259105	1.231202
C	4.975016	3.724642	3.406374
C	4.100483	4.931286	3.166676
H	3.099390	4.942162	3.626527
H	4.579636	5.919922	3.072601
H	4.569478	2.912327	4.030361
H	6.066042	3.867541	3.480515

4G a+b

Cu4RX-OCF3ALKIPh--2Npre SCF Done: -4596.24892194 A.U.

N	-0.642605	-1.752620	-0.552040
C	-2.600505	-2.165649	1.750433
H	-1.576780	-2.576850	1.823046
C	-2.876572	-1.449859	0.419772
C	-1.984933	-1.265754	-0.654291
C	0.455809	-0.927459	-0.374670
C	-2.409901	-0.581053	-1.804085
C	-1.590048	-0.319132	-3.070369
H	-0.567582	-0.734857	-3.014472
C	-4.185829	-0.935526	0.333280
C	-4.613275	-0.229401	-0.805318
C	-3.716832	-0.057524	-1.873669
C	-0.276122	-3.085808	-0.437765
C	1.089584	-3.113629	-0.157621
N	1.509873	-1.797193	-0.124763
C	2.760646	-1.245546	0.317799
C	2.824559	-0.701891	1.612892
C	3.801068	-1.009863	-0.590002
C	3.917453	0.101225	1.990599
C	4.878011	-0.180787	-0.217317
C	4.928006	0.410743	1.059071
C	1.700062	-0.710114	2.651353

H	0.828162	-1.313765	2.339101
C	3.807175	-1.381162	-2.074060
H	2.954542	-2.024961	-2.359670
C	3.808577	-0.032066	-2.804943
C	2.852809	0.421293	-3.719030
C	4.892493	0.797048	-2.430124
C	2.977262	1.718553	-4.260692
H	2.007396	-0.226024	-4.003134
C	5.012514	2.085387	-2.960992
C	4.045128	2.546186	-3.878824
H	2.222365	2.087779	-4.972268
H	5.850685	2.733530	-2.658366
H	4.126773	3.562619	-4.294713
C	5.166873	-2.028081	-2.337101
C	5.395394	-3.303127	-2.867845
C	6.252579	-1.197531	-1.967362
C	6.722545	-3.750920	-3.036587
H	4.545686	-3.946485	-3.148529
C	7.568956	-1.643028	-2.134640
C	7.801072	-2.925931	-2.673615
H	6.912859	-4.751836	-3.455460
H	8.412555	-0.996469	-1.844098
H	8.834588	-3.282498	-2.808356
C	1.347360	0.770576	2.861153
C	2.454373	1.545846	3.276173
C	0.103204	1.373027	2.644039
C	2.313293	2.919277	3.501834
C	-0.040716	2.752684	2.889988
H	-0.749476	0.783341	2.278194
C	1.053449	3.524289	3.307525
H	3.179604	3.515826	3.831386
H	-1.016944	3.230375	2.739614
H	0.918911	4.605786	3.462874
C	2.333045	-1.195734	3.953204
C	1.911880	-2.279859	4.732083
C	3.450332	-0.418563	4.344530
C	2.610595	-2.588732	5.918258
H	1.045550	-2.884640	4.417354
C	4.144705	-0.727745	5.519838
C	3.719169	-1.817977	6.308188
H	2.284911	-3.438082	6.539527
H	5.014330	-0.123355	5.824155
H	4.260452	-2.065134	7.235212
C	-1.596757	1.201237	-3.248608
C	-0.472128	2.034109	-3.285945
C	-2.902202	1.745812	-3.309287
C	-0.654673	3.426074	-3.402162
H	0.537179	1.617038	-3.163894
C	-3.081552	3.128780	-3.432829
C	-1.948235	3.967149	-3.482991
H	0.225408	4.085335	-3.374741
H	-4.096226	3.556064	-3.457584

H	-2.082605	5.057570	-3.535598
C	-2.422175	-0.889113	-4.223216
C	-2.010484	-1.851002	-5.151993
C	-3.728485	-0.343484	-4.287041
C	-2.910346	-2.272718	-6.154224
H	-0.993774	-2.272879	-5.098698
C	-4.620832	-0.762409	-5.280352
C	-4.205883	-1.732664	-6.217718
H	-2.593463	-3.028704	-6.890008
H	-5.635069	-0.333594	-5.329512
H	-4.901975	-2.065540	-7.003896
C	-2.913663	-1.153297	2.856321
C	-4.221586	-0.621037	2.759837
C	-2.063544	-0.764542	3.897367
C	-4.669623	0.320111	3.691998
C	-2.510521	0.195915	4.828645
H	-1.048753	-1.184358	3.981050
C	-3.802178	0.736323	4.724121
H	-5.686714	0.735669	3.614888
H	-1.834409	0.527924	5.631264
H	-4.141160	1.492127	5.449656
C	-3.683073	-3.245451	1.869644
C	-3.478624	-4.615895	2.064453
C	-4.995991	-2.721856	1.772122
C	-4.595493	-5.475266	2.140414
H	-2.456972	-5.018026	2.144041
C	-6.103163	-3.574489	1.846947
C	-5.897600	-4.959582	2.027815
H	-4.441153	-6.555932	2.287043
H	-7.123724	-3.165029	1.771563
H	-6.764254	-5.636870	2.088006
O	-5.884460	0.305661	-0.856275
C	-6.855855	-0.580124	-1.419857
H	-7.826254	-0.046442	-1.409716
H	-6.597662	-0.854320	-2.469385
H	-6.946493	-1.520676	-0.827099
O	5.887999	1.353384	1.351627
C	5.428218	2.685094	1.042680
H	6.231960	3.383502	1.345152
H	5.212313	2.798744	-0.043422
H	4.490033	2.926120	1.587123
C	1.540369	-4.497107	-0.014689
C	2.732454	-5.166490	0.245327
C	0.343986	-5.269519	-0.227578
C	2.710592	-6.597198	0.297306
H	3.673624	-4.618904	0.406801
C	0.302291	-6.677133	-0.180024
C	1.544855	-7.339826	0.096264
H	3.653713	-7.126668	0.504707
H	1.575836	-8.440087	0.146492
C	-0.805436	-4.445682	-0.498304
C	-2.032014	-5.059699	-0.727645

C	-2.096034	-6.489238	-0.679024
H	-2.940180	-4.470678	-0.923142
C	-0.978309	-7.282941	-0.415463
H	-3.070617	-6.972285	-0.849872
H	-1.074439	-8.380252	-0.387669
C	5.809726	0.146032	-1.385641
C	-3.999982	0.682876	-3.182769
C	-5.019458	-1.200616	1.586063
C	3.750125	0.728496	3.377630
H	6.652714	0.789988	-1.077314
H	4.628684	1.330478	3.671811
H	-6.038629	-0.781207	1.508925
H	-5.018123	1.110134	-3.208977
Cu	0.820623	0.928525	-0.409467
C	1.540353	2.633998	-0.402587
C	2.131908	3.726783	-0.333624
C	2.853066	4.977331	-0.304506
H	3.438759	5.180294	-1.221870
C	3.485449	5.514337	0.986367
C	2.304011	6.227205	0.386095
H	1.330477	6.153342	0.894323
H	2.468496	7.190540	-0.123651
H	3.305926	4.925243	1.898001
H	4.485930	5.972912	0.917521
O	-0.982499	5.978325	2.102297
C	-1.559374	5.446278	1.159033
C	-2.569068	4.380148	1.283151
C	-2.650940	3.247635	0.394798
C	-3.522928	4.456303	2.321953
N	-3.755205	2.458681	0.359023
C	-4.598368	3.563080	2.346282
C	-4.692918	2.627864	1.289173
H	-3.425920	5.266394	3.062339
H	-5.378936	3.621143	3.118360
H	-5.582530	1.979585	1.182834
N	-1.621010	2.890826	-0.396633
H	-0.652074	3.232295	-0.302782
H	-1.757977	2.099848	-1.031159
C	-1.330946	6.054785	-0.280091
F	-0.341946	5.434185	-0.967339
F	-2.462525	5.977482	-1.027435
F	-0.994032	7.353829	-0.168274

4G a+b→c

Cu4RX-OCF3ALKIPh--2N SCF Done: -4596.23202169 A.U.

Cu	0.379218	-1.577000	0.020954
C	1.431455	-2.443707	-1.328738
C	2.628247	-2.812120	-1.368456
C	3.979425	-3.285190	-1.382971
H	4.188102	-4.094003	-0.660830
C	5.144740	-2.323724	-1.643644

C	4.785009	-3.342062	-2.685980
H	4.271000	-2.988779	-3.592277
H	5.431531	-4.223993	-2.823787
H	4.871611	-1.278110	-1.844829
H	6.051278	-2.454498	-1.031194
O	-0.018155	-2.451888	-3.761302
C	-0.082625	-3.163233	-2.740638
C	-1.380896	-3.169377	-1.925646
C	-1.573312	-3.423073	-0.533814
C	-2.548353	-2.912013	-2.670299
N	-2.781457	-3.412902	0.058473
C	-3.810778	-2.915397	-2.062092
C	-3.867063	-3.175509	-0.685351
H	-2.437062	-2.706005	-3.743046
H	-4.717767	-2.713574	-2.649524
H	-4.826567	-3.193232	-0.139564
N	-0.502931	-3.572605	0.372551
H	0.279034	-4.150356	0.040787
H	-0.868504	-3.850111	1.292629
C	0.559789	-4.583710	-2.872297
F	0.711648	-5.263073	-1.688870
F	-0.286903	-5.342906	-3.626675
F	1.747790	-4.567158	-3.496668
N	1.037345	1.095554	0.875829
C	2.686962	-0.639331	2.812535
H	1.609119	-0.474120	2.992357
C	3.201595	0.108399	1.583384
C	2.434212	0.854073	0.678750
C	0.043023	0.143039	0.660390
C	3.040512	1.359039	-0.481091
C	2.306026	1.936428	-1.689905
H	1.210734	1.982951	-1.550177
C	4.577339	-0.087838	1.344197
C	5.211314	0.510342	0.236477
C	4.430869	1.241066	-0.680130
C	0.489022	2.360892	1.024022
C	-0.889832	2.214071	0.935708
N	-1.138703	0.867204	0.714044
C	-2.454574	0.359687	0.469630
C	-3.170670	-0.269771	1.495204
C	-3.097823	0.656459	-0.746639
C	-4.527479	-0.603674	1.309531
C	-4.481244	0.423503	-0.885080
C	-5.208042	-0.221455	0.137875
C	-2.645465	-0.603803	2.892689
H	-1.588392	-0.312213	3.031280
C	-2.472379	1.349202	-1.961777
H	-1.378271	1.475552	-1.865120
C	-2.872528	0.535609	-3.199054
C	-2.011456	0.030634	-4.177127
C	-4.272428	0.359549	-3.317637
C	-2.552914	-0.665545	-5.277584

H	-0.923662	0.145364	-4.084298
C	-4.810566	-0.339548	-4.403223
C	-3.940036	-0.855493	-5.388340
H	-1.871865	-1.086239	-6.032502
H	-5.901198	-0.474095	-4.491531
H	-4.352717	-1.408956	-6.246650
C	-3.226990	2.675109	-2.095632
C	-2.668137	3.956299	-2.128835
C	-4.628775	2.494999	-2.183618
C	-3.519814	5.075145	-2.235765
H	-1.577700	4.087567	-2.045921
C	-5.474247	3.605904	-2.281564
C	-4.912226	4.901426	-2.304760
H	-3.088797	6.088087	-2.247998
H	-6.565889	3.468584	-2.343464
H	-5.572204	5.780108	-2.381396
C	-2.878083	-2.102425	3.069181
C	-4.222395	-2.481283	2.848644
C	-1.913250	-3.054892	3.411028
C	-4.590128	-3.828933	2.920997
C	-2.285501	-4.413236	3.488981
H	-0.877825	-2.738416	3.617199
C	-3.612428	-4.797271	3.228850
H	-5.634056	-4.129637	2.735720
H	-1.534500	-5.173968	3.756249
H	-3.894862	-5.860552	3.279020
C	-3.603054	0.092015	3.865503
C	-3.258540	1.023459	4.850745
C	-4.954090	-0.289580	3.677858
C	-4.273164	1.576452	5.660714
H	-2.206853	1.323079	4.986581
C	-5.960158	0.259076	4.480698
C	-5.613327	1.196000	5.477521
H	-4.011548	2.311574	6.438174
H	-7.011026	-0.036186	4.330512
H	-6.399910	1.632779	6.113095
C	2.719273	0.998292	-2.831681
C	1.885776	0.106768	-3.511680
C	4.115131	0.988813	-3.062601
C	2.432153	-0.727710	-4.508326
H	0.827402	-0.000921	-3.231688
C	4.659336	0.182087	-4.068148
C	3.802402	-0.661064	-4.809167
H	1.775819	-1.468151	-4.987571
H	5.746341	0.175584	-4.247618
H	4.224302	-1.307557	-5.595159
C	2.934652	3.302986	-1.947547
C	2.267013	4.532429	-1.986911
C	4.340462	3.242141	-2.109617
C	3.008882	5.713926	-2.192150
H	1.176528	4.575114	-1.835647
C	5.076276	4.415657	-2.315982

C	4.403696	5.655859	-2.355514
H	2.490437	6.685170	-2.216318
H	6.169872	4.367918	-2.444787
H	4.977010	6.582883	-2.515544
C	3.018016	-2.106188	2.523827
C	4.395219	-2.326323	2.286612
C	2.102741	-3.159286	2.443460
C	4.860958	-3.614623	2.002845
C	2.568507	-4.454998	2.141148
H	1.030411	-2.961555	2.590540
C	3.939842	-4.682285	1.933758
H	5.932765	-3.788282	1.816008
H	1.856022	-5.291965	2.067523
H	4.298039	-5.697632	1.701760
C	3.579188	-0.212533	3.980384
C	3.157141	0.342956	5.193385
C	4.957217	-0.428507	3.733032
C	4.118778	0.688761	6.166537
H	2.084207	0.507708	5.383470
C	5.911214	-0.085684	4.698125
C	5.485898	0.476830	5.920538
H	3.794193	1.126905	7.123605
H	6.982378	-0.257929	4.503343
H	6.230331	0.748836	6.685535
O	6.559875	0.319612	0.009809
C	7.393455	1.241001	0.717291
H	8.443183	0.980541	0.478402
H	7.194114	2.291772	0.402011
H	7.238920	1.172696	1.819876
O	-6.571582	-0.406564	0.044550
C	-7.013232	-1.427668	-0.852363
H	-8.102350	-1.284742	-0.993813
H	-6.505568	-1.367917	-1.841104
H	-6.834924	-2.443485	-0.431687
C	-1.546268	3.513148	1.048409
C	-2.844101	4.011200	1.026789
C	-0.448178	4.435400	1.173574
C	-3.028229	5.426853	1.125065
H	-3.710905	3.344718	0.903283
C	-0.607891	5.832808	1.250927
C	-1.959182	6.318969	1.229113
H	-4.055855	5.819928	1.093162
H	-2.148109	7.402995	1.287808
C	0.831926	3.775045	1.166106
C	1.985193	4.549836	1.228830
C	1.844672	5.972002	1.308830
H	2.984673	4.091500	1.188314
C	0.600205	6.605432	1.322632
H	2.759205	6.583926	1.344658
H	0.540818	7.704365	1.378690
C	-5.051259	1.017071	-2.175939
C	4.891463	1.817789	-2.028388

C	5.227221	-1.037454	2.355138
C	-5.138327	-1.292481	2.531089
H	-6.145749	0.894422	-2.259318
H	-6.193766	-1.575420	2.367732
H	6.298954	-1.205770	2.145719
H	5.987001	1.755270	-2.158297

4G c

Cu4RX-OCF3ALKIPh--2Npost SCF Done: -4596.25473818 A.U.

N	-0.869998	-1.274282	-0.636943
C	-2.213427	-1.527479	2.040619
H	-1.120656	-1.647377	1.934002
C	-2.893561	-1.138822	0.730653
C	-2.276583	-1.037612	-0.526563
C	0.083175	-0.264701	-0.579372
C	-3.041127	-0.694418	-1.647884
C	-2.528444	-0.529196	-3.077159
H	-1.455461	-0.774776	-3.175896
C	-4.283856	-0.949623	0.857864
C	-5.056966	-0.570972	-0.257059
C	-4.416658	-0.415435	-1.504268
C	-0.284266	-2.528207	-0.585604
C	1.086439	-2.319220	-0.493514
N	1.290473	-0.946988	-0.496632
C	2.557405	-0.412524	-0.082423
C	2.725173	-0.042612	1.262195
C	3.676142	-0.477025	-0.923920
C	4.011761	0.207201	1.780334
C	4.960098	-0.201109	-0.408770
C	5.145608	0.117228	0.950631
C	1.620737	0.080563	2.309311
H	0.615513	-0.084608	1.882557
C	3.684098	-0.821028	-2.417431
H	2.673414	-1.030763	-2.813135
C	4.355448	0.380405	-3.091260
C	3.793063	1.206825	-4.070970
C	5.640934	0.659627	-2.567127
C	4.515805	2.331470	-4.520995
H	2.786586	0.993617	-4.464826
C	6.357806	1.775373	-3.014154
C	5.787082	2.615398	-3.994118
H	4.074306	2.996032	-5.279806
H	7.353341	1.997705	-2.596945
H	6.340888	3.500468	-4.344594
C	4.644633	-2.002529	-2.559582
C	4.341340	-3.262877	-3.086599
C	5.932000	-1.730956	-2.035900
C	5.333144	-4.265610	-3.086928
H	3.331933	-3.472430	-3.474237
C	6.916487	-2.726430	-2.037537
C	6.610660	-3.999178	-2.565445

H	5.099981	-5.263461	-3.490228
H	7.916011	-2.517431	-1.623735
H	7.379647	-4.787883	-2.566493
C	1.781770	1.495599	2.876000
C	3.067792	1.726115	3.420158
C	0.840821	2.528573	2.807978
C	3.403850	2.985537	3.929404
C	1.185591	3.806060	3.296040
H	-0.143524	2.365631	2.340159
C	2.452195	4.025728	3.864097
H	4.407308	3.165002	4.349354
H	0.487757	4.639984	3.140817
H	2.716207	5.030210	4.230526
C	1.998440	-0.907504	3.414548
C	1.225739	-1.972724	3.892908
C	3.290662	-0.668693	3.942563
C	1.741963	-2.799053	4.912926
H	0.222064	-2.164663	3.484898
C	3.803526	-1.489227	4.953754
C	3.021171	-2.557752	5.441042
H	1.134868	-3.634641	5.295327
H	4.810245	-1.301444	5.360624
H	3.418142	-3.206156	6.238157
C	-2.841323	0.924167	-3.431824
C	-1.913493	1.899643	-3.814858
C	-4.211357	1.244336	-3.279663
C	-2.365077	3.210917	-4.064908
H	-0.843479	1.649725	-3.890560
C	-4.655934	2.550739	-3.516943
C	-3.724909	3.533175	-3.913779
H	-1.643292	3.994363	-4.336985
H	-5.718561	2.808693	-3.382958
H	-4.060749	4.567790	-4.081289
C	-3.438236	-1.399528	-3.949256
C	-3.034865	-2.427484	-4.808723
C	-4.810920	-1.078514	-3.811116
C	-4.010744	-3.139924	-5.537698
H	-1.966473	-2.677167	-4.911117
C	-5.779038	-1.785247	-4.533911
C	-5.372475	-2.821295	-5.401547
H	-3.701450	-3.950356	-6.216457
H	-6.845889	-1.529808	-4.427157
H	-6.127672	-3.381387	-5.975336
C	-2.607233	-0.471339	3.078415
C	-4.008157	-0.337848	3.220383
C	-1.740315	0.289809	3.869869
C	-4.542353	0.520634	4.185923
C	-2.278560	1.179302	4.822030
H	-0.650837	0.193672	3.771658
C	-3.668457	1.281159	4.990589
H	-5.634310	0.609793	4.303463
H	-1.597325	1.791406	5.432601

H	-4.081095	1.972871	5.741666
C	-2.915459	-2.823494	2.459651
C	-2.308963	-4.063600	2.684091
C	-4.320164	-2.672889	2.572995
C	-3.115280	-5.169615	3.028587
H	-1.218954	-4.177043	2.572135
C	-5.117932	-3.770548	2.914118
C	-4.508012	-5.024062	3.142269
H	-2.647037	-6.151505	3.200138
H	-6.210452	-3.655353	3.003337
H	-5.130703	-5.892131	3.411049
O	-6.413293	-0.367240	-0.131409
C	-7.190284	-1.516569	-0.485004
H	-8.255436	-1.236969	-0.368664
H	-7.004031	-1.831602	-1.538510
H	-6.960595	-2.380593	0.181715
O	6.414721	0.311665	1.454589
C	6.819080	1.684036	1.513993
H	7.818610	1.705651	1.990149
H	6.888956	2.130805	0.494724
H	6.110068	2.298179	2.115175
C	1.781337	-3.591131	-0.306828
C	3.089788	-4.031385	-0.132419
C	0.714896	-4.558057	-0.304220
C	3.314732	-5.435283	0.032403
H	3.937133	-3.329575	-0.124978
C	0.915291	-5.941464	-0.125266
C	2.276064	-6.368739	0.039570
H	4.351752	-5.782521	0.159188
H	2.496997	-7.439307	0.178287
C	-0.582107	-3.954284	-0.468968
C	-1.709964	-4.767855	-0.441067
C	-1.527317	-6.175316	-0.257988
H	-2.722077	-4.347700	-0.537930
C	-0.266099	-6.756260	-0.107539
H	-2.421445	-6.816998	-0.226678
H	-0.173779	-7.845135	0.034168
C	6.062291	-0.315305	-1.463360
C	-5.061391	0.053600	-2.810932
C	-4.796498	-1.243793	2.269385
C	3.999325	0.517588	3.280879
H	7.063261	-0.106617	-1.045068
H	5.013193	0.689557	3.684761
H	-5.890294	-1.113494	2.353720
H	-6.129150	0.308380	-2.686361
Cu	-0.219808	1.630381	-0.509692
C	0.234629	3.540740	-0.315928
C	1.290788	2.977904	-0.742170
C	2.723475	2.957780	-0.974322
H	3.109270	2.227929	-1.701476
C	3.643146	3.298286	0.196775
C	3.429789	4.313060	-0.892246

H	2.777410	5.170453	-0.654455
H	4.235678	4.496749	-1.621520
H	3.163603	3.472969	1.172045
H	4.593127	2.745908	0.229299
O	0.269871	5.496422	0.976234
C	-0.543714	4.752756	0.259500
C	-1.814983	4.235647	1.014496
C	-2.576741	3.082110	0.703678
C	-2.217754	4.971351	2.137909
N	-3.615534	2.645429	1.435941
C	-3.330145	4.558988	2.886951
C	-3.987958	3.380862	2.497298
H	-1.612653	5.858903	2.386728
H	-3.678177	5.128399	3.762735
H	-4.855805	3.005176	3.063873
N	-2.258130	2.254610	-0.427784
H	-2.300563	2.807350	-1.300090
H	-2.971822	1.514466	-0.482286
C	-1.066590	5.561859	-0.990745
F	-1.955745	4.839851	-1.781698
F	-1.732782	6.668550	-0.599337
F	-0.063268	5.945046	-1.807564
4G c′			
Cu4RX-OCF3ALKIPhISOMER1--2min SCF Done: -4596.27117168 A.U.			
Cu	0.475084	-1.471225	0.527496
N	0.727798	1.325380	0.809069
C	3.189183	1.106388	2.629308
H	2.171263	0.974531	3.040220
C	3.188495	1.349428	1.122119
C	2.066580	1.343894	0.289437
C	-0.146499	0.257212	0.639551
C	2.239498	1.446779	-1.099783
C	1.132278	1.348122	-2.144950
H	0.132968	1.213500	-1.695710
C	4.478466	1.508938	0.571332
C	4.658525	1.674384	-0.816767
C	3.524810	1.601651	-1.653411
C	0.039253	2.471953	1.163853
C	-1.308641	2.135160	1.211677
N	-1.405857	0.790537	0.877472
C	-2.671242	0.180155	0.583941
C	-3.219804	-0.844766	1.373215
C	-3.415810	0.702110	-0.494196
C	-4.550053	-1.263109	1.149186
C	-4.724911	0.244437	-0.736108
C	-5.304499	-0.741396	0.083536
C	-2.528564	-1.646010	2.478601
H	-1.466086	-1.369692	2.612029
C	-2.927463	1.690564	-1.564642
H	-1.893378	2.038812	-1.394160

C	-3.094963	0.951790	-2.900823
C	-2.093896	0.677121	-3.838713
C	-4.421026	0.504601	-3.119817
C	-2.412124	-0.077779	-4.987306
H	-1.065167	1.029911	-3.689160
C	-4.739944	-0.237111	-4.262852
C	-3.723594	-0.534842	-5.197168
H	-1.618031	-0.314398	-5.712029
H	-5.771853	-0.588309	-4.425070
H	-3.962900	-1.126679	-6.094773
C	-3.939086	2.835111	-1.585942
C	-3.659440	4.195627	-1.416650
C	-5.266210	2.389260	-1.794369
C	-4.721521	5.122521	-1.446852
H	-2.626229	4.531085	-1.232714
C	-6.320468	3.309918	-1.820178
C	-6.041852	4.682333	-1.643403
H	-4.512499	6.193883	-1.302541
H	-7.354851	2.964290	-1.977152
H	-6.866703	5.412219	-1.661850
C	-2.706903	-3.125537	2.084007
C	-4.065488	-3.498583	1.939745
C	-1.689976	-4.061898	1.859742
C	-4.420030	-4.813569	1.619950
C	-2.049655	-5.387386	1.526881
H	-0.624877	-3.776248	1.894665
C	-3.397971	-5.766200	1.423172
H	-5.481514	-5.093222	1.520270
H	-1.253947	-6.119791	1.334497
H	-3.659241	-6.805090	1.166431
C	-3.382090	-1.422554	3.728914
C	-2.945767	-0.914175	4.957561
C	-4.739582	-1.777158	3.539691
C	-3.874719	-0.761625	6.008301
H	-1.888049	-0.639249	5.098316
C	-5.661265	-1.625285	4.581834
C	-5.222283	-1.114188	5.821555
H	-3.540224	-0.364885	6.979944
H	-6.717064	-1.903477	4.432716
H	-5.941188	-0.992136	6.647214
C	1.551025	0.149476	-3.002009
C	0.845951	-1.050578	-3.145539
C	2.831071	0.314350	-3.583349
C	1.416559	-2.085952	-3.913446
H	-0.121338	-1.196110	-2.640035
C	3.392604	-0.713857	-4.346209
C	2.673723	-1.915255	-4.514416
H	0.905905	-3.056899	-3.986635
H	4.398707	-0.600873	-4.780952
H	3.131945	-2.749462	-5.065573
C	1.246169	2.600528	-3.010864
C	0.240927	3.543611	-3.259342

C	2.533525	2.757410	-3.578061
C	0.523028	4.649892	-4.087661
H	-0.761363	3.424876	-2.818607
C	2.813403	3.856420	-4.398541
C	1.799766	4.804104	-4.653813
H	-0.263818	5.393437	-4.290198
H	3.816911	3.978544	-4.837102
H	2.012721	5.670754	-5.299566
C	4.065451	-0.141348	2.787767
C	5.369236	0.031295	2.261811
C	3.651346	-1.386077	3.274937
C	6.272125	-1.037647	2.257138
C	4.556503	-2.467514	3.251424
H	2.619167	-1.530561	3.630063
C	5.858749	-2.291404	2.755755
H	7.283748	-0.907444	1.839652
H	4.225855	-3.459705	3.592576
H	6.554873	-3.144134	2.729804
C	3.938210	2.292433	3.233533
C	3.440457	3.194232	4.181598
C	5.236693	2.458939	2.693728
C	4.252815	4.267537	4.603696
H	2.422949	3.067767	4.584931
C	6.043568	3.522437	3.116023
C	5.545951	4.427995	4.076803
H	3.869755	4.982471	5.348976
H	7.053053	3.652980	2.694046
H	6.174884	5.268100	4.411751
O	5.907490	1.944131	-1.332629
C	6.628637	0.835407	-1.877498
H	5.999435	0.225836	-2.563413
H	7.487673	1.255667	-2.436044
H	7.012620	0.164887	-1.074515
O	-6.568943	-1.220520	-0.188346
C	-6.536159	-2.391550	-1.018158
H	-7.585803	-2.707551	-1.174794
H	-6.059857	-2.173823	-2.002344
H	-5.963942	-3.217658	-0.536414
C	-2.109085	3.316008	1.529423
C	-3.453322	3.632769	1.692873
C	-1.128570	4.362286	1.661417
C	-3.800118	4.989515	1.987378
H	-4.239825	2.872603	1.574987
C	-1.449032	5.701636	1.959629
C	-2.844503	5.998615	2.120093
H	-4.865922	5.240675	2.099856
H	-3.158850	7.030212	2.346184
C	0.215933	3.892214	1.452211
C	1.271911	4.790734	1.570232
C	0.965969	6.154351	1.880219
H	2.316437	4.469324	1.437846
C	-0.342079	6.609343	2.064923

H	1.800103	6.867026	1.974362
H	-0.529058	7.670192	2.296999
C	-5.369009	0.870334	-1.973601
C	3.501805	1.638629	-3.186809
C	5.583560	1.414558	1.630232
C	-5.026527	-2.326596	2.138934
H	-6.404653	0.517391	-2.126209
H	-6.088213	-2.592206	1.986818
H	6.591041	1.552267	1.197986
H	4.506143	1.773903	-3.626002
C	0.499096	-4.147905	-1.022167
C	-0.520372	-3.982190	-1.689494
C	-1.726284	-3.660517	-2.406113
H	-1.685623	-3.798246	-3.501940
C	-2.544049	-2.454501	-1.920689
C	-3.097654	-3.840925	-1.751586
H	-3.114207	-4.272414	-0.740019
H	-3.921417	-4.161222	-2.410362
H	-2.166876	-1.939084	-1.021782
H	-2.966170	-1.793946	-2.692280
O	1.282021	-3.076832	0.918328
C	1.652812	-3.942295	-0.102280
C	2.909082	-3.448411	-0.877607
C	3.406200	-4.006589	-2.100908
C	3.647095	-2.409363	-0.302483
N	4.564993	-3.589207	-2.664501
C	4.830969	-1.954152	-0.904016
C	5.247811	-2.596646	-2.077037
H	3.284988	-1.970601	0.638690
H	5.412670	-1.152094	-0.435069
H	6.189779	-2.299358	-2.577178
N	2.724232	-4.959437	-2.825489
H	1.996295	-5.507057	-2.367916
H	3.282124	-5.427450	-3.541263
C	2.038320	-5.279145	0.620718
F	1.028893	-5.743562	1.397463
F	2.344510	-6.277301	-0.262021
F	3.113299	-5.106168	1.419629

4G d

Cu4RX-OCF3ALKIPh--2NpostH+ SCF Done: -4596.71649212 A.U.

N	0.792022	1.333918	-0.574286
C	2.214281	1.680678	2.054647
H	1.117414	1.799564	1.982482
C	2.858375	1.277947	0.729271
C	2.207155	1.136866	-0.507558
C	-0.127791	0.301467	-0.481655
C	2.936147	0.765006	-1.643461
C	2.381288	0.573158	-3.050534
H	1.298621	0.784967	-3.117853
C	4.254559	1.095311	0.816435

C	4.998546	0.703618	-0.317274
C	4.321298	0.516920	-1.541675
C	0.171331	2.568983	-0.531626
C	-1.192267	2.320755	-0.406273
N	-1.353683	0.943266	-0.385875
C	-2.602617	0.359482	0.012744
C	-2.743687	-0.106316	1.330049
C	-3.725721	0.422095	-0.826460
C	-4.009431	-0.494493	1.814195
C	-4.990462	0.034930	-0.334625
C	-5.150256	-0.408671	0.993281
C	-1.642013	-0.197907	2.386059
H	-0.651729	0.100300	1.996980
C	-3.755482	0.847175	-2.298792
H	-2.761165	1.145048	-2.679737
C	-4.348066	-0.356245	-3.041781
C	-3.747963	-1.069897	-4.085927
C	-5.613212	-0.747293	-2.538613
C	-4.410862	-2.194504	-4.622510
H	-2.765973	-0.760112	-4.478423
C	-6.269619	-1.863935	-3.069321
C	-5.660716	-2.590183	-4.115601
H	-3.946071	-2.763016	-5.442951
H	-7.253239	-2.167731	-2.676421
H	-6.172144	-3.468257	-4.539887
C	-4.793562	1.967610	-2.389886
C	-4.578074	3.263070	-2.873372
C	-6.059318	1.586422	-1.885527
C	-5.639263	4.191153	-2.844666
H	-3.586632	3.556414	-3.252481
C	-7.112846	2.508112	-1.856669
C	-6.895886	3.816520	-2.338489
H	-5.478646	5.214922	-3.216611
H	-8.097781	2.214596	-1.460189
H	-7.719589	4.547163	-2.318971
C	-1.665854	-1.653678	2.858323
C	-2.936672	-2.057286	3.333387
C	-0.599829	-2.559148	2.823400
C	-3.147083	-3.374368	3.758405
C	-0.811319	-3.884879	3.253142
H	0.390524	-2.238823	2.460726
C	-2.074985	-4.290961	3.712646
H	-4.137590	-3.688083	4.125349
H	0.016529	-4.605582	3.210983
H	-2.230817	-5.330258	4.041271
C	-2.126065	0.660850	3.557930
C	-1.464510	1.758139	4.122590
C	-3.388685	0.252076	4.049582
C	-2.064310	2.441731	5.200875
H	-0.488625	2.086730	3.731385
C	-3.984538	0.931230	5.117914
C	-3.312533	2.028834	5.696474

H	-1.549844	3.303272	5.654184
H	-4.968402	0.614319	5.498814
H	-3.773330	2.567584	6.539087
C	2.732022	-0.864950	-3.432954
C	1.838214	-1.848645	-3.875651
C	4.119706	-1.134584	-3.342761
C	2.344037	-3.102736	-4.274516
H	0.758515	-1.634083	-3.925015
C	4.617132	-2.387094	-3.725498
C	3.722456	-3.366379	-4.205130
H	1.652792	-3.884160	-4.624547
H	5.696926	-2.596976	-3.666345
H	4.107660	-4.350486	-4.513567
C	3.234629	1.478064	-3.949726
C	2.768642	2.494720	-4.790319
C	4.620444	1.204806	-3.853711
C	3.697779	3.242746	-5.544212
H	1.689927	2.707950	-4.860735
C	5.542364	1.945882	-4.601353
C	5.073099	2.970418	-5.450808
H	3.341055	4.043973	-6.210037
H	6.620087	1.727574	-4.529600
H	5.790350	3.557747	-6.045059
C	2.640997	0.630539	3.086027
C	4.046149	0.498375	3.185853
C	1.799357	-0.122248	3.913568
C	4.610484	-0.353229	4.140844
C	2.366990	-0.997485	4.862191
H	0.707132	-0.017841	3.854562
C	3.762310	-1.098144	4.986869
H	5.705384	-0.437286	4.228971
H	1.706676	-1.588210	5.515633
H	4.198209	-1.764975	5.747512
C	2.928590	2.981727	2.439874
C	2.327235	4.223803	2.667549
C	4.335126	2.833210	2.514671
C	3.142926	5.332999	2.976573
H	1.234136	4.335119	2.590070
C	5.142958	3.933764	2.821555
C	4.538568	5.188783	3.053099
H	2.680226	6.316212	3.154168
H	6.237354	3.820522	2.882539
H	5.167663	6.059438	3.295866
O	6.355565	0.511432	-0.228387
C	7.121096	1.673278	-0.583933
H	8.188657	1.396612	-0.492459
H	6.909324	1.996658	-1.629206
H	6.898789	2.525394	0.099045
O	-6.395580	-0.737151	1.478173
C	-6.735226	-2.123014	1.357036
H	-7.725582	-2.255313	1.833093
H	-6.799615	-2.435356	0.288563

H	-5.989868	-2.774386	1.868918
C	-1.917915	3.574650	-0.217609
C	-3.233050	3.980957	-0.009141
C	-0.878282	4.569158	-0.254563
C	-3.492985	5.379125	0.144797
H	-4.060186	3.257002	0.032293
C	-1.113546	5.949638	-0.087349
C	-2.480313	6.340763	0.109374
H	-4.534830	5.699796	0.297340
H	-2.727699	7.406280	0.240218
C	0.431690	4.003298	-0.441537
C	1.537217	4.848667	-0.450815
C	1.318193	6.251859	-0.283563
H	2.559051	4.457808	-0.567036
C	0.043439	6.797431	-0.110341
H	2.192734	6.920345	-0.284080
H	-0.076820	7.885010	0.017853
C	-6.097132	0.137913	-1.384154
C	4.939983	0.073186	-2.867893
C	4.806123	1.402141	2.209740
C	-3.978438	-0.936118	3.278569
H	-7.083575	-0.155555	-0.983646
H	-4.974159	-1.236099	3.650122
H	5.901870	1.274298	2.264729
H	6.018931	-0.145983	-2.779038
Cu	0.315633	-1.574350	-0.361743
C	-0.025510	-3.492126	-0.337732
C	-1.101154	-2.973903	-0.756461
C	-2.469833	-2.844821	-1.187644
H	-2.692494	-1.984806	-1.836091
C	-3.581618	-3.282184	-0.233808
C	-3.266080	-4.130566	-1.432606
H	-2.729816	-5.079326	-1.272072
H	-3.954929	-4.106171	-2.292346
H	-3.276808	-3.624720	0.768653
H	-4.484468	-2.657325	-0.264771
O	0.006057	-5.645234	0.575700
C	0.851776	-4.632751	0.043605
C	1.972536	-4.221351	1.017794
C	2.659874	-2.980412	0.951560
C	2.379051	-5.112790	2.025394
N	3.619488	-2.622833	1.812091
C	3.400909	-4.754838	2.915335
C	3.985216	-3.486701	2.771274
H	1.873355	-6.084503	2.122875
H	3.731189	-5.441228	3.708718
H	4.782487	-3.146586	3.451357
N	2.348440	-1.972979	-0.029207
H	2.647719	-2.261614	-0.978099
H	2.906676	-1.141409	0.222065
C	1.526702	-5.211842	-1.249962
F	2.521794	-4.406206	-1.688436

F	2.081026	-6.422715	-0.958480
F	0.650683	-5.392437	-2.251339
H	0.442848	-6.510127	0.434037
4G d→f			
Cu4RX-OCF3ALKIPh--2NpostH--int1+ SCF Done: -4596.69395702 A.U.			
N	-0.284664	1.744153	-0.136021
C	-2.222862	2.210065	-2.403664
H	-1.140884	2.393755	-2.538872
C	-2.595573	1.864720	-0.963995
C	-1.698782	1.672714	0.093044
C	0.471118	0.591022	-0.207453
C	-2.180284	1.300727	1.358097
C	-1.346182	1.066049	2.613307
H	-0.260316	1.178494	2.438138
C	-3.974641	1.648169	-0.769413
C	-4.468989	1.274814	0.496156
C	-3.563605	1.128800	1.564703
C	0.521908	2.864146	-0.222056
C	1.835773	2.401086	-0.329673
N	1.778038	1.016035	-0.324406
C	2.896799	0.120977	-0.362645
C	3.115735	-0.701009	-1.481868
C	3.750028	0.030168	0.750291
C	4.192948	-1.611935	-1.483569
C	4.806499	-0.903839	0.748319
C	5.027313	-1.744298	-0.358292
C	2.266596	-0.784950	-2.751069
H	1.399709	-0.099115	-2.737789
C	3.626029	0.791871	2.075232
H	2.796247	1.522538	2.083976
C	3.474538	-0.294665	3.145683
C	2.431156	-0.413867	4.068755
C	4.527214	-1.242567	3.126103
C	2.412328	-1.514998	4.952875
H	1.631482	0.343146	4.105138
C	4.511039	-2.333712	4.002646
C	3.439778	-2.472328	4.913260
H	1.584667	-1.619538	5.671574
H	5.330428	-3.070480	3.986540
H	3.421152	-3.326651	5.608060
C	4.993006	1.440450	2.307815
C	5.243823	2.804037	2.500533
C	6.054036	0.504494	2.301914
C	6.573831	3.236257	2.683907
H	4.413319	3.527207	2.499139
C	7.373808	0.933982	2.485366
C	7.629694	2.308290	2.676262
H	6.783609	4.306683	2.835053
H	8.201159	0.206747	2.478038
H	8.665036	2.654188	2.821296

C	1.850117	-2.257153	-2.864742
C	2.951249	-3.147648	-2.887728
C	0.543132	-2.746129	-2.947990
C	2.747306	-4.526273	-3.010010
C	0.334958	-4.137852	-3.070879
H	-0.311148	-2.052292	-2.926439
C	1.427706	-5.019625	-3.107642
H	3.607535	-5.214073	-3.040564
H	-0.689138	-4.527851	-3.142150
H	1.255972	-6.102223	-3.215432
C	3.231808	-0.523628	-3.910135
C	3.105573	0.466667	-4.890606
C	4.333896	-1.410719	-3.919285
C	4.092079	0.568188	-5.893577
H	2.245418	1.155594	-4.878858
C	5.313479	-1.309578	-4.913805
C	5.186871	-0.313081	-5.904342
H	4.002509	1.341655	-6.672248
H	6.171796	-2.000028	-4.921049
H	5.952303	-0.227101	-6.691290
C	-1.721790	-0.328367	3.123666
C	-0.842912	-1.381833	3.395816
C	-3.106871	-0.466754	3.376328
C	-1.345941	-2.558379	3.987810
H	0.226704	-1.290776	3.157174
C	-3.604081	-1.636033	3.960935
C	-2.714012	-2.682710	4.277702
H	-0.655053	-3.376636	4.246726
H	-4.677976	-1.732100	4.179418
H	-3.097073	-3.603891	4.741096
C	-1.899389	2.058862	3.643675
C	-1.173757	3.037090	4.331675
C	-3.291859	1.912178	3.856961
C	-1.846438	3.875503	5.246122
H	-0.091005	3.150298	4.159838
C	-3.958567	2.742973	4.763782
C	-3.227386	3.729393	5.460006
H	-1.284501	4.647327	5.794884
H	-5.040931	2.625287	4.933734
H	-3.743860	4.387035	6.176519
C	-2.736120	1.028684	-3.229175
C	-4.122478	0.797183	-3.053448
C	-1.977386	0.213817	-4.080477
C	-4.752925	-0.245312	-3.743254
C	-2.618061	-0.826945	-4.783921
H	-0.898142	0.396799	-4.209389
C	-3.995114	-1.051552	-4.617407
H	-5.828763	-0.433231	-3.601946
H	-2.031554	-1.473624	-5.454464
H	-4.484257	-1.875234	-5.159589
C	-3.086125	3.421629	-2.765236
C	-2.624641	4.671499	-3.193906

C	-4.471236	3.184590	-2.591570
C	-3.560744	5.692768	-3.461845
H	-1.544686	4.853059	-3.315611
C	-5.399060	4.197689	-2.859492
C	-4.936543	5.456674	-3.298328
H	-3.209923	6.679090	-3.803597
H	-6.477572	4.012603	-2.729409
H	-5.659969	6.258489	-3.513641
O	-5.807122	1.002825	0.678742
C	-6.608101	2.161835	0.950069
H	-7.654376	1.815502	1.052157
H	-6.288456	2.659673	1.894224
H	-6.544411	2.905397	0.122574
O	6.018749	-2.698278	-0.325878
C	5.547207	-3.971774	0.135923
H	4.716857	-4.354865	-0.502281
H	6.403273	-4.671831	0.089860
H	5.176639	-3.907760	1.185777
C	2.759868	3.529363	-0.404799
C	4.135177	3.726278	-0.488450
C	1.892425	4.676749	-0.359139
C	4.625674	5.069588	-0.532272
H	4.837516	2.879707	-0.508076
C	2.356404	6.007102	-0.416674
C	3.778511	6.180087	-0.503197
H	5.713844	5.225186	-0.591592
H	4.201221	7.196476	-0.545939
C	0.500972	4.323610	-0.256756
C	-0.453547	5.337224	-0.238521
C	-0.001702	6.692914	-0.305656
H	-1.529777	5.112898	-0.182437
C	1.352020	7.030925	-0.385717
H	-0.754480	7.496170	-0.291618
H	1.652898	8.089730	-0.430345
C	5.585606	-0.935509	2.061310
C	-3.910581	0.786342	3.015256
C	-4.776472	1.776078	-2.067845
C	4.296913	-2.427886	-2.771674
H	6.413947	-1.665643	2.036819
H	5.165912	-3.109804	-2.767071
H	-5.853384	1.579246	-1.922630
H	-4.997188	0.662788	3.170824
Cu	-0.340679	-1.102287	-0.137873
C	-1.491337	-2.635009	-0.003239
C	-0.742875	-3.411408	0.696949
C	0.469867	-3.871386	1.326465
H	0.349551	-4.098062	2.398102
C	1.835473	-3.267827	0.944590
C	1.524931	-4.686768	0.571892
H	1.328478	-4.886803	-0.493205
H	2.001002	-5.507613	1.131778
H	1.866410	-2.524724	0.129529

H	2.503750	-3.040706	1.792088
O	-3.450731	-1.374892	-0.729060
C	-2.951926	-2.659478	-0.417346
C	-3.818970	-3.240065	0.695738
C	-3.431580	-4.374939	1.456217
C	-5.053012	-2.642657	1.010848
N	-4.165765	-4.875643	2.461363
C	-5.845338	-3.195814	2.026113
C	-5.351233	-4.307777	2.729552
H	-5.372499	-1.742220	0.466820
H	-6.822643	-2.759772	2.281046
H	-5.930446	-4.756072	3.556119
N	-2.182595	-5.013142	1.237657
H	-2.088360	-5.431388	0.304017
H	-2.013558	-5.713187	1.972889
C	-3.143197	-3.526693	-1.709741
F	-2.645769	-4.795459	-1.571989
F	-4.440297	-3.630913	-2.032593
F	-2.497811	-2.962649	-2.758644
H	-2.871671	-0.958563	-1.403533

4G f

Cu4RX-OCF3ALKIPh--2NpostHint1+ SCF Done: -4596.70680225 A.U.

N	0.351252	1.668883	0.416055
C	2.471046	1.966384	2.567889
H	1.404482	2.141996	2.799509
C	2.724873	1.729841	1.080017
C	1.746610	1.592053	0.090784
C	-0.436324	0.530913	0.438306
C	2.129202	1.280551	-1.224955
C	1.201580	1.117866	-2.425752
H	0.132341	1.203550	-2.160172
C	4.083661	1.521444	0.765223
C	4.475687	1.212577	-0.550549
C	3.490933	1.126929	-1.552329
C	-0.429334	2.806732	0.532588
C	-1.755080	2.376527	0.615226
N	-1.733634	0.991151	0.549843
C	-2.866248	0.116006	0.462819
C	-3.131948	-0.811755	1.484272
C	-3.678064	0.143204	-0.686171
C	-4.219526	-1.700785	1.362266
C	-4.734418	-0.781500	-0.816588
C	-5.006923	-1.718775	0.196714
C	-2.357455	-0.998275	2.790866
H	-1.479321	-0.332110	2.871360
C	-3.522935	1.049281	-1.914369
H	-2.694959	1.775675	-1.819416
C	-3.352947	0.107763	-3.111042
C	-2.320721	0.135863	-4.053565
C	-4.401420	-0.838870	-3.223537

C	-2.305719	-0.821690	-5.091354
H	-1.523299	0.893369	-3.988428
C	-4.385028	-1.791737	-4.248147
C	-3.322490	-1.786244	-5.179627
H	-1.485229	-0.813147	-5.825309
H	-5.201009	-2.527383	-4.333559
H	-3.304837	-2.530141	-5.991644
C	-4.885850	1.721992	-2.108653
C	-5.134092	3.097988	-2.172663
C	-5.944875	0.791160	-2.231531
C	-6.459593	3.547734	-2.347731
H	-4.305522	3.817074	-2.078733
C	-7.259841	1.238129	-2.405010
C	-7.513803	2.625050	-2.460344
H	-6.666530	4.628230	-2.397691
H	-8.085145	0.514261	-2.496639
H	-8.545688	2.984718	-2.596523
C	-1.975299	-2.482978	2.842267
C	-3.092660	-3.347795	2.746808
C	-0.684725	-3.007882	2.971309
C	-2.925959	-4.736052	2.798688
C	-0.516348	-4.409664	3.027363
H	0.187040	-2.336193	3.017840
C	-1.626584	-5.265916	2.946575
H	-3.800812	-5.402675	2.733096
H	0.493259	-4.830884	3.130387
H	-1.483445	-6.356760	2.997022
C	-3.390360	-0.783766	3.902122
C	-3.314237	0.148783	4.942334
C	-4.507214	-1.645554	3.788571
C	-4.364612	0.216885	5.881585
H	-2.443697	0.819417	5.025075
C	-5.549371	-1.578353	4.719967
C	-5.473035	-0.639972	5.770780
H	-4.314366	0.945626	6.705703
H	-6.418375	-2.249552	4.630771
H	-6.288618	-0.580029	6.508272
C	1.542352	-0.228636	-3.069275
C	0.644770	-1.256230	-3.364992
C	2.904874	-0.340573	-3.435668
C	1.095298	-2.382058	-4.084448
H	-0.404672	-1.180095	-3.055796
C	3.356101	-1.461224	-4.140946
C	2.440445	-2.481577	-4.477376
H	0.372766	-3.168196	-4.356316
H	4.410930	-1.534805	-4.446691
H	2.787994	-3.355746	-5.049457
C	1.667101	2.184930	-3.423966
C	0.885434	3.205336	-3.975195
C	3.038797	2.066546	-3.757337
C	1.479968	4.115566	-4.875286
H	-0.178888	3.298301	-3.704200

C	3.627667	2.968497	-4.649964
C	2.839492	3.997444	-5.209552
H	0.873986	4.922145	-5.316563
H	4.693661	2.875097	-4.913292
H	3.295129	4.711592	-5.913151
C	3.032328	0.715026	3.246894
C	4.395444	0.485894	2.935837
C	2.323700	-0.187903	4.045649
C	5.047814	-0.654387	3.425611
C	2.983258	-1.329020	4.547730
H	1.259930	-0.012013	4.272322
C	4.333449	-1.561730	4.239933
H	6.107044	-0.836321	3.182523
H	2.430034	-2.048558	5.170467
H	4.837330	-2.461544	4.624097
C	3.372578	3.139453	2.957667
C	2.959910	4.354400	3.517442
C	4.737796	2.910519	2.661759
C	3.925202	5.345541	3.793551
H	1.894609	4.532495	3.735029
C	5.695682	3.892983	2.938896
C	5.282438	5.115354	3.509597
H	3.612207	6.303845	4.236665
H	6.759180	3.712674	2.713100
H	6.029190	5.893262	3.732826
O	5.796539	0.934509	-0.848378
C	6.600930	2.099038	-1.087759
H	7.632312	1.747111	-1.281938
H	6.229880	2.666862	-1.971336
H	6.603106	2.781993	-0.207630
O	-6.015653	-2.643100	0.039284
C	-5.543442	-3.898188	-0.466781
H	-6.419638	-4.570956	-0.537688
H	-5.084008	-3.777594	-1.475714
H	-4.780711	-4.349777	0.209591
C	-2.647268	3.528404	0.727670
C	-4.015765	3.761713	0.824263
C	-1.748914	4.653080	0.717795
C	-4.468870	5.116617	0.909553
H	-4.742009	2.935505	0.822142
C	-2.175281	5.993439	0.813227
C	-3.592123	6.203749	0.907428
H	-5.552204	5.300017	0.978049
H	-3.986345	7.229926	0.979342
C	-0.367654	4.263366	0.609179
C	0.614490	5.250300	0.619964
C	0.200688	6.616210	0.721499
H	1.684220	4.996937	0.559957
C	-1.142983	6.989814	0.809762
H	0.975686	7.398260	0.729025
H	-1.414553	8.054980	0.882709
C	-5.477315	-0.667447	-2.145399

C	3.727352	0.890528	-3.046553
C	4.986967	1.551098	2.002760
C	-4.413492	-2.594484	2.586913
H	-6.304136	-1.395492	-2.225075
H	-5.292514	-3.256203	2.488173
H	6.048670	1.364613	1.760898
H	4.799512	0.792327	-3.294330
Cu	0.286416	-1.195275	0.271508
C	1.298619	-2.723619	-0.207536
C	0.910896	-3.678322	-1.076071
C	-0.450642	-4.028040	-1.577290
H	-0.450920	-4.702403	-2.453077
C	-1.619645	-3.049866	-1.544113
C	-1.623420	-4.222090	-0.607288
H	-1.397294	-4.013240	0.450719
H	-2.312361	-5.063317	-0.785235
H	-1.433537	-2.053345	-1.101232
H	-2.290123	-3.030403	-2.419736
O	3.000964	-1.348009	0.752256
C	2.753774	-2.644991	0.241349
C	3.686822	-3.005691	-0.905988
C	3.330746	-4.041786	-1.774431
C	4.929837	-2.396683	-1.152832
N	4.065389	-4.552675	-2.750917
C	5.735188	-2.902382	-2.184700
C	5.277928	-3.998332	-2.941649
H	5.251474	-1.529400	-0.556758
H	6.713798	-2.449624	-2.403013
H	5.895108	-4.432477	-3.745984
N	1.971623	-4.629951	-1.646806
H	2.025731	-5.479080	-1.049004
H	1.708863	-4.949581	-2.599784
C	3.031768	-3.667225	1.390226
F	2.789183	-4.955986	0.995799
F	4.336360	-3.606911	1.767878
F	2.285437	-3.427748	2.478310
H	3.755358	-1.341807	1.383228

4G f→g

Cu4RX-OCF3ALKIPh--2NpostHint1--2+Fll
SCF Done: -4673.10266777 A.U.

N	0.904452	-1.417718	-0.546241
C	3.358606	-1.139753	-2.363795
H	2.359738	-1.320558	-2.801904
C	3.333689	-1.061541	-0.836109
C	2.206846	-1.157979	-0.008936
C	-0.142577	-0.517162	-0.428586
C	2.353403	-1.024897	1.383443
C	1.254502	-1.210537	2.426928
H	0.252833	-1.336987	1.980194
C	4.605967	-0.839396	-0.266492
C	4.768044	-0.734893	1.129101

C	3.626829	-0.830773	1.950419
C	0.452848	-2.641254	-1.014385
C	-0.921131	-2.511900	-1.211289
N	-1.260259	-1.216510	-0.850755
C	-2.599326	-0.713472	-0.749931
C	-3.068883	0.290316	-1.614048
C	-3.437323	-1.207771	0.266369
C	-4.372773	0.805180	-1.450418
C	-4.736787	-0.684815	0.420784
C	-5.209459	0.339271	-0.420880
C	-2.290335	0.995217	-2.726547
H	-1.258644	0.614123	-2.836142
C	-3.074740	-2.235068	1.347619
H	-2.052011	-2.641384	1.239225
C	-3.290568	-1.513147	2.683449
C	-2.354755	-1.354672	3.710966
C	-4.595865	-0.977802	2.818442
C	-2.707893	-0.617465	4.862377
H	-1.351520	-1.799765	3.629879
C	-4.946338	-0.244502	3.957643
C	-3.990094	-0.056754	4.979998
H	-1.968181	-0.483548	5.666954
H	-5.961738	0.171684	4.057826
H	-4.259837	0.515764	5.881409
C	-4.155502	-3.316357	1.269346
C	-3.949546	-4.685909	1.067008
C	-5.463944	-2.795848	1.410126
C	-5.066908	-5.543838	0.999818
H	-2.929400	-5.083155	0.948098
C	-6.572339	-3.648327	1.345480
C	-6.367504	-5.028871	1.137787
H	-4.916218	-6.622493	0.837127
H	-7.591432	-3.244013	1.452563
H	-7.234280	-5.706024	1.084475
C	-2.333459	2.487251	-2.369724
C	-3.648698	2.989374	-2.218416
C	-1.231525	3.326819	-2.190855
C	-3.859927	4.333922	-1.891834
C	-1.441791	4.683323	-1.863131
H	-0.214569	2.925832	-2.291884
C	-2.745235	5.184318	-1.714746
H	-4.884828	4.723308	-1.781067
H	-0.576773	5.349623	-1.726169
H	-2.900439	6.245286	-1.462750
C	-3.141322	0.836445	-3.990361
C	-2.735018	0.269555	-5.203591
C	-4.456695	1.334537	-3.834646
C	-3.654034	0.201421	-6.271504
H	-1.709697	-0.116683	-5.321015
C	-5.369110	1.266339	-4.893572
C	-4.960444	0.696144	-6.117415
H	-3.344614	-0.241506	-7.231109

H	-6.393357	1.652764	-4.770152
H	-5.671323	0.638874	-6.956498
C	1.320583	-0.010911	3.370727
C	0.253687	0.817777	3.727525
C	2.596738	0.158789	3.964131
C	0.437529	1.769707	4.757362
H	-0.723448	0.700760	3.237925
C	2.793459	1.132848	4.950615
C	1.697845	1.918376	5.374687
H	-0.427418	2.351475	5.118984
H	3.779802	1.251903	5.425954
H	1.830134	2.636559	6.199923
C	1.711400	-2.414747	3.261617
C	1.005738	-3.606065	3.460287
C	2.993690	-2.232880	3.835489
C	1.585019	-4.623879	4.248299
H	0.014424	-3.751061	3.001137
C	3.567177	-3.240518	4.618025
C	2.854085	-4.441770	4.822883
H	1.038421	-5.565790	4.410686
H	4.562862	-3.097499	5.067866
H	3.297948	-5.241069	5.436559
C	3.988628	0.178329	-2.825053
C	5.260559	0.410555	-2.250544
C	3.423648	1.109100	-3.702429
C	5.964212	1.583293	-2.545016
C	4.135186	2.288974	-4.004113
H	2.430833	0.925741	-4.143231
C	5.393892	2.525260	-3.427118
H	6.952951	1.765918	-2.094612
H	3.696577	3.029676	-4.690658
H	5.942652	3.449752	-3.666081
C	4.369740	-2.240351	-2.701521
C	4.129893	-3.382058	-3.474729
C	5.645251	-2.012668	-2.130582
C	5.174184	-4.310067	-3.672406
H	3.134900	-3.556811	-3.913419
C	6.681290	-2.933491	-2.327442
C	6.439170	-4.088468	-3.101659
H	4.994964	-5.211435	-4.279110
H	7.675348	-2.753683	-1.886946
H	7.249610	-4.816297	-3.263511
O	6.013993	-0.557682	1.688419
C	6.733791	-1.784297	1.880099
H	7.714069	-1.518888	2.320321
H	6.188795	-2.467375	2.572149
H	6.894450	-2.319808	0.916127
O	-6.452125	0.895679	-0.215885
C	-6.413092	2.032667	0.657401
H	-7.452038	2.401351	0.756661
H	-6.016951	1.756921	1.662425
H	-5.766471	2.840981	0.244243

C	-1.489959	-3.791040	-1.629457
C	-2.746819	-4.305357	-1.932949
C	-0.350445	-4.669608	-1.667274
C	-2.847454	-5.690122	-2.279124
H	-3.649195	-3.677201	-1.893984
C	-0.429055	-6.035433	-2.007926
C	-1.736924	-6.536346	-2.322697
H	-3.843091	-6.097666	-2.512324
H	-1.862562	-7.596557	-2.594491
C	0.874209	-4.011011	-1.295497
C	2.055492	-4.746792	-1.273603
C	1.997877	-6.131142	-1.630133
H	3.015467	-4.284578	-1.000152
C	0.805447	-6.767010	-1.985239
H	2.934013	-6.710567	-1.620983
H	0.808584	-7.837091	-2.247230
C	-5.489745	-1.275965	1.610766
C	3.618458	-0.872433	3.481185
C	5.712974	-0.717165	-1.314360
C	-4.718418	1.916570	-2.440826
H	-6.510623	-0.864735	1.703981
H	-5.751507	2.285367	-2.311662
H	6.703453	-0.529138	-0.862990
H	4.624820	-0.721513	3.910801
Cu	0.070593	1.241239	0.210652
C	0.390091	3.045690	0.739035
C	-0.572711	3.731328	1.442758
C	-1.786588	3.135171	2.089963
H	-1.656478	2.982138	3.178038
C	-2.677322	2.131420	1.402677
C	-3.196681	3.529693	1.667325
H	-3.301034	4.184511	0.786938
H	-3.950365	3.678663	2.457323
H	-2.449630	1.891662	0.352058
H	-3.063133	1.285035	1.993079
O	2.637185	3.640908	1.628969
C	1.629688	3.817979	0.388326
C	1.411792	5.292662	0.180547
C	0.369305	5.914103	0.926413
C	2.138397	6.141666	-0.688963
N	0.074638	7.223180	0.895005
C	1.825263	7.504368	-0.749905
C	0.795665	8.000355	0.077550
H	2.939422	5.740133	-1.323032
H	2.373636	8.179428	-1.423119
H	0.534880	9.073409	0.067195
N	-0.450680	5.118408	1.727052
H	-1.302561	5.605983	2.036133
H	0.644712	4.749045	3.207253
C	2.512053	3.162860	-0.714720
F	1.965150	3.352459	-1.934448
F	3.760642	3.731704	-0.732442

F	2.698820	1.848451	-0.521714
H	3.444789	4.172101	1.443008
H	2.103039	4.020836	2.640281
O	1.476560	4.375430	3.624080
H	1.215939	3.506550	4.052092

4G f→g +H_2O

Cu4RX-OCF3ALKIPh--2NpostHint3+H2O+
SCF Done: -4596.75530073 A.U.

N	0.806375	1.362785	0.507033
C	3.221167	1.282466	2.400936
H	2.196198	1.351558	2.809633
C	3.246980	1.201942	0.873275
C	2.142760	1.220772	0.007951
C	-0.163903	0.378282	0.415490
C	2.346834	1.172670	-1.383375
C	1.265130	1.160732	-2.464033
H	0.243047	1.187824	-2.046274
C	4.553783	1.131839	0.340034
C	4.771103	1.104840	-1.052179
C	3.652809	1.119397	-1.909009
C	0.246485	2.573150	0.883098
C	-1.120657	2.353446	1.038812
N	-1.345701	1.017977	0.748507
C	-2.640486	0.417546	0.615958
C	-3.106363	-0.521123	1.548239
C	-3.446344	0.778568	-0.478242
C	-4.380757	-1.105099	1.384105
C	-4.720351	0.197009	-0.632147
C	-5.197429	-0.752965	0.292337
C	-2.367500	-1.026612	2.788297
H	-1.364190	-0.577267	2.903293
C	-3.086589	1.750668	-1.608688
H	-2.081832	2.198711	-1.496109
C	-3.240052	0.948766	-2.905044
C	-2.263269	0.752610	-3.887365
C	-4.524448	0.367298	-3.044982
C	-2.557630	-0.060663	-5.002500
H	-1.273503	1.224236	-3.797987
C	-4.820564	-0.432820	-4.154987
C	-3.825080	-0.653260	-5.133029
H	-1.785892	-0.227944	-5.769611
H	-5.820867	-0.882069	-4.263792
H	-4.049874	-1.282355	-6.008592
C	-4.205297	2.796161	-1.616857
C	-4.050508	4.180836	-1.487560
C	-5.491324	2.222421	-1.757319
C	-5.197619	5.001357	-1.494115
H	-3.047516	4.619112	-1.366532
C	-6.629179	3.037430	-1.765423
C	-6.476125	4.433932	-1.631512
H	-5.087617	6.091916	-1.388809

H	-7.631119	2.592308	-1.872387
H	-7.366458	5.081973	-1.636046
C	-2.317172	-2.547484	2.623486
C	-3.597004	-3.133260	2.464716
C	-1.161794	-3.333255	2.568194
C	-3.717762	-4.511778	2.253514
C	-1.282177	-4.721172	2.343174
H	-0.173131	-2.867394	2.686074
C	-2.550816	-5.305570	2.189466
H	-4.712635	-4.970012	2.132775
H	-0.372265	-5.337867	2.291141
H	-2.639203	-6.390538	2.021894
C	-3.302536	-0.745922	3.968032
C	-2.998916	0.000878	5.111765
C	-4.581592	-1.329575	3.808634
C	-3.984930	0.162834	6.107298
H	-2.001712	0.454160	5.231619
C	-5.560113	-1.169640	4.796414
C	-5.254900	-0.418548	5.950758
H	-3.756803	0.747796	7.011984
H	-6.556035	-1.623688	4.671411
H	-6.018511	-0.287301	6.733254
C	1.529480	-0.109362	-3.278715
C	0.636942	-1.169769	-3.477747
C	2.841929	-0.165320	-3.808601
C	1.059416	-2.294903	-4.216626
H	-0.377682	-1.121424	-3.064283
C	3.259949	-1.282831	-4.540642
C	2.360965	-2.351400	-4.743314
H	0.359487	-3.128939	-4.380847
H	4.284961	-1.332142	-4.939632
H	2.685887	-3.234858	-5.314593
C	1.585175	2.342793	-3.381046
C	0.734440	3.409252	-3.693013
C	2.895479	2.285179	-3.914638
C	1.196326	4.426120	-4.555218
H	-0.280877	3.456303	-3.266801
C	3.353075	3.294017	-4.769706
C	2.494501	4.367567	-5.089902
H	0.534945	5.269193	-4.808562
H	4.371338	3.248291	-5.188316
H	2.846271	5.164641	-5.763327
C	3.984390	0.048132	2.890632
C	5.287150	-0.045628	2.347099
C	3.509463	-0.928417	3.772513
C	6.109201	-1.132415	2.667598
C	4.337781	-2.021339	4.098151
H	2.495173	-0.849322	4.194624
C	5.625525	-2.124663	3.545217
H	7.121233	-1.208144	2.239259
H	3.969186	-2.800271	4.783524
H	6.265393	-2.983568	3.801309

C	4.089674	2.496595	2.750997
C	3.694661	3.617531	3.489952
C	5.399747	2.408525	2.220851
C	4.618266	4.663925	3.697665
H	2.671667	3.685114	3.892467
C	6.315975	3.446326	2.427875
C	5.918216	4.578852	3.170666
H	4.316598	5.550438	4.277067
H	7.336554	3.376471	2.018060
H	6.633896	5.398446	3.340375
O	6.045306	1.055484	-1.563292
C	6.621942	2.349826	-1.790145
H	7.639780	2.183708	-2.191722
H	6.024269	2.935826	-2.526195
H	6.687799	2.936263	-0.844554
O	-6.447981	-1.308398	0.134098
C	-6.449420	-2.547144	-0.580358
H	-7.504681	-2.868995	-0.670640
H	-6.011639	-2.428896	-1.599496
H	-5.873830	-3.332634	-0.036577
C	-1.795908	3.607295	1.364672
C	-3.096836	4.042506	1.599259
C	-0.724329	4.567672	1.391077
C	-3.309015	5.432118	1.864424
H	-3.949931	3.348590	1.568255
C	-0.913111	5.939763	1.655650
C	-2.264244	6.358857	1.897383
H	-4.338982	5.777217	2.042502
H	-2.475743	7.419730	2.105973
C	0.558541	3.982409	1.102282
C	1.686167	4.798893	1.092491
C	1.515430	6.191552	1.371218
H	2.688428	4.394194	0.887557
C	0.266417	6.756697	1.641438
H	2.407896	6.836147	1.370141
H	0.182860	7.836231	1.844698
C	-5.459221	0.692929	-1.875527
C	3.684318	1.059233	-3.437950
C	5.636806	1.122934	1.417945
C	-4.730281	-2.101817	2.492280
H	-6.463262	0.243068	-1.973311
H	-5.734874	-2.542137	2.361693
H	6.652404	1.048311	0.990192
H	4.714608	1.011196	-3.832659
Cu	0.082790	-1.445477	-0.023229
C	0.415156	-3.276866	-0.492427
C	-0.345449	-3.959091	-1.502080
C	-1.681929	-3.566077	-1.995684
H	-2.029368	-4.160983	-2.858113
C	-2.315113	-2.186012	-1.875337
C	-2.770302	-3.252029	-0.942994
H	-2.412723	-3.191790	0.096455

H	-3.746383	-3.740070	-1.079704
H	-1.704857	-1.374809	-1.437773
H	-2.953489	-1.877138	-2.717162
O	3.173191	-1.937697	-1.121129
C	1.533865	-3.966442	0.007501
C	1.968250	-5.244882	-0.497021
C	1.237519	-5.796633	-1.593200
C	3.071160	-6.033348	-0.047845
N	1.515700	-6.943522	-2.238910
C	3.364708	-7.226897	-0.699114
C	2.565226	-7.638487	-1.798789
H	3.684416	-5.698889	0.798249
H	4.210616	-7.850630	-0.373892
H	2.792987	-8.577125	-2.333714
N	0.113387	-5.125901	-2.021945
H	-0.405975	-5.607794	-2.769409
H	2.488484	-1.590038	-1.728106
C	2.287737	-3.436594	1.233268
F	1.981292	-4.229758	2.308042
F	3.629050	-3.454275	1.109339
F	1.935555	-2.169874	1.576660
H	3.260584	-1.221886	-0.461381
4G g			
Cu4RX-OCF3ALKIPh--2NpostHint3--PROD+ SCF Done: -4596.68778360 A.U.			
N	0.644915	-1.366383	0.625846
C	2.004305	-2.236464	-1.940153
H	0.924962	-2.427239	-1.790571
C	2.665035	-1.534873	-0.747202
C	2.057874	-1.177831	0.468153
C	-0.277055	-0.359399	0.384133
C	2.830499	-0.616662	1.498784
C	2.407385	-0.418009	2.959016
H	1.344085	-0.662379	3.137402
C	4.041866	-1.296305	-0.940697
C	4.815380	-0.681246	0.059396
C	4.194042	-0.346382	1.277807
C	0.007871	-2.578982	0.824110
C	-1.361393	-2.335496	0.716993
N	-1.508540	-0.984937	0.450501
C	-2.731386	-0.371971	0.019577
C	-2.915942	-0.109848	-1.348570
C	-3.759586	-0.090669	0.928856
C	-4.124771	0.451696	-1.805808
C	-4.967159	0.471879	0.467271
C	-5.160675	0.753283	-0.899878
C	-1.911493	-0.372172	-2.472125
H	-0.959878	-0.796403	-2.105154
C	-3.730266	-0.309225	2.443051
H	-2.776783	-0.742713	2.797955
C	-4.030216	1.063710	3.052448

C	-3.224161	1.771175	3.951061
C	-5.247473	1.619057	2.588905
C	-3.632765	3.050015	4.385646
H	-2.279754	1.331291	4.311081
C	-5.653609	2.887509	3.019883
C	-4.837798	3.604972	3.921403
H	-3.006612	3.614908	5.093932
H	-6.601187	3.319103	2.659476
H	-5.151352	4.602745	4.265872
C	-4.941244	-1.196603	2.745240
C	-4.921459	-2.443099	3.381842
C	-6.157859	-0.642823	2.280283
C	-6.133019	-3.145033	3.552513
H	-3.970154	-2.873430	3.732801
C	-7.360232	-1.338940	2.451870
C	-7.342174	-2.596756	3.091302
H	-6.129149	-4.127225	4.050447
H	-8.307396	-0.910190	2.087825
H	-8.283729	-3.150909	3.229322
C	-1.719683	0.984648	-3.156744
C	-2.934944	1.545936	-3.618256
C	-0.518686	1.694867	-3.267564
C	-2.944683	2.818879	-4.199845
C	-0.530730	2.982009	-3.844607
H	0.422116	1.257206	-2.904477
C	-1.733601	3.538000	-4.309714
H	-3.889702	3.259749	-4.556052
H	0.410450	3.548223	-3.918676
H	-1.736524	4.543885	-4.757720
C	-2.635932	-1.275185	-3.469979
C	-2.215230	-2.534736	-3.912410
C	-3.848150	-0.714206	-3.937033
C	-3.010622	-3.236017	-4.842687
H	-1.276908	-2.974225	-3.536509
C	-4.638479	-1.411398	-4.857919
C	-4.211481	-2.677265	-5.312295
H	-2.688231	-4.226290	-5.200371
H	-5.583329	-0.976504	-5.220779
H	-4.826743	-3.231239	-6.038348
C	2.769019	1.008678	3.356818
C	1.952411	1.911690	4.049304
C	4.116781	1.341505	3.070256
C	2.459474	3.195915	4.381385
H	0.931358	1.618492	4.344362
C	4.609870	2.619059	3.370735
C	3.774939	3.552828	4.015406
H	1.848791	3.892526	4.980313
H	5.651572	2.880283	3.127441
H	4.163039	4.550034	4.273925
C	3.375893	-1.299116	3.768556
C	3.028077	-2.332505	4.644329
C	4.735181	-0.969768	3.546466

C	4.052598	-3.045577	5.303576
H	1.969875	-2.588284	4.813360
C	5.750366	-1.672619	4.203774
C	5.401323	-2.717819	5.086235
H	3.790822	-3.863187	5.993055
H	6.807559	-1.412047	4.034882
H	6.192947	-3.277919	5.607784
C	2.295080	-1.378526	-3.175847
C	3.672931	-1.107725	-3.346409
C	1.362654	-0.898936	-4.101551
C	4.112500	-0.305019	-4.404150
C	1.803982	-0.093323	-5.172002
H	0.294931	-1.142237	-4.004191
C	3.166866	0.213319	-5.314041
H	5.185340	-0.087002	-4.526499
H	1.068827	0.300625	-5.890337
H	3.503177	0.849592	-6.147481
C	2.816284	-3.525368	-2.117148
C	2.322225	-4.833500	-2.070415
C	4.196719	-3.280666	-2.319396
C	3.219119	-5.911041	-2.230724
H	1.249758	-5.017005	-1.899741
C	5.084746	-4.350233	-2.481163
C	4.588431	-5.671279	-2.436038
H	2.840717	-6.944597	-2.195776
H	6.158114	-4.161669	-2.645111
H	5.280413	-6.518127	-2.565309
O	6.159534	-0.452220	-0.121090
C	6.982425	-1.580106	0.214878
H	8.030452	-1.285320	0.016064
H	6.870590	-1.856411	1.288728
H	6.725420	-2.472034	-0.401431
O	-6.349088	1.292831	-1.336171
C	-6.343892	2.722933	-1.411041
H	-7.359739	3.035207	-1.720107
H	-6.100212	3.178841	-0.423363
H	-5.604767	3.089445	-2.161017
C	-2.105002	-3.586626	0.831419
C	-3.435039	-3.996205	0.802291
C	-1.072986	-4.574725	1.003514
C	-3.715655	-5.391659	0.946403
H	-4.256817	-3.275274	0.679225
C	-1.330058	-5.955488	1.131198
C	-2.709905	-6.348831	1.103085
H	-4.767613	-5.715661	0.928430
H	-2.974076	-7.413817	1.201259
C	0.250409	-4.008303	1.001274
C	1.347861	-4.856920	1.114861
C	1.106937	-6.261310	1.240904
H	2.377877	-4.470951	1.092676
C	-0.180358	-6.805111	1.254723
H	1.974322	-6.934128	1.323709

H	-0.316681	-7.893963	1.352139
C	-5.976886	0.715059	1.590048
C	4.918269	0.162766	2.520879
C	4.551989	-1.787253	-2.293324
C	-4.150523	0.659233	-3.324111
H	-6.924713	1.141400	1.216489
H	-5.107043	1.086157	-3.674303
H	5.628133	-1.585945	-2.438240
H	5.974751	0.412741	2.318392
Cu	0.081136	1.449613	-0.085041
C	0.551507	3.279666	-0.443352
C	-0.283485	4.434751	-0.117885
C	-1.753142	4.348564	0.109158
H	-2.174632	5.346299	0.309107
C	-2.472473	3.184974	0.804898
C	-2.630646	3.374141	-0.668638
H	-2.101805	2.672133	-1.330528
H	-3.577475	3.759723	-1.073856
H	-1.878079	2.319893	1.149966
H	-3.294564	3.456052	1.485568
O	0.459574	3.618354	2.342425
C	1.851099	3.601437	-0.866965
C	2.360826	4.948166	-0.834157
C	1.525497	5.925458	-0.177146
C	3.653357	5.377240	-1.262275
N	1.965688	7.168984	0.159524
C	4.063228	6.669642	-0.962449
C	3.190302	7.515383	-0.219755
H	4.319600	4.695275	-1.808344
H	5.052021	7.037296	-1.276648
H	3.525780	8.529813	0.066157
N	0.222741	5.648994	0.137668
H	0.432491	4.537527	1.935108
H	0.473188	2.985158	1.504567
C	2.827474	2.517603	-1.306042
F	3.290413	2.745543	-2.563153
F	3.908111	2.449243	-0.478938
F	2.279467	1.270849	-1.325826
H	1.300121	3.447921	2.905036

4G g→h +2H_2O

Cu4RX-OCF3ALKIPh--2NpostHint3--PROD+2H2O SCF Done: -4673.10544074 A.U.

N	-0.893090	-1.426392	-0.255265
C	-2.356701	-1.070734	2.361089
H	-1.293604	-1.375988	2.349987
C	-2.925727	-0.818061	0.960347
C	-2.270061	-1.025394	-0.264389
C	0.170616	-0.544732	-0.333226
C	-2.969321	-0.874944	-1.470050
C	-2.453552	-1.188844	-2.875814

H	-1.398158	-1.518362	-2.884613
C	-4.283623	-0.441568	0.972200
C	-4.991314	-0.256034	-0.230682
C	-4.319196	-0.472649	-1.448904
C	-0.448235	-2.708046	0.012185
C	0.941329	-2.642612	0.102888
N	1.293021	-1.319383	-0.105254
C	2.595129	-0.787321	0.170333
C	2.817744	-0.135697	1.395356
C	3.655560	-0.974984	-0.724446
C	4.110981	0.310244	1.732662
C	4.943739	-0.508121	-0.391601
C	5.187076	0.128090	0.841581
C	1.765976	0.213338	2.448975
H	0.749625	-0.112185	2.164283
C	3.585051	-1.640977	-2.099274
H	2.570385	-2.000030	-2.352773
C	4.113147	-0.592195	-3.082652
C	3.445984	-0.095120	-4.207402
C	5.406665	-0.123205	-2.746842
C	4.073755	0.886833	-5.002816
H	2.441531	-0.467633	-4.465608
C	6.026468	0.856522	-3.531145
C	5.352638	1.362511	-4.664276
H	3.558825	1.280593	-5.893081
H	7.033577	1.220515	-3.271262
H	5.838150	2.126929	-5.291032
C	4.625798	-2.763429	-2.056770
C	4.390777	-4.125083	-2.279169
C	5.920626	-2.299634	-1.723255
C	5.464267	-5.033369	-2.166826
H	3.378341	-4.481171	-2.527037
C	6.985804	-3.201016	-1.612891
C	6.751196	-4.574371	-1.837056
H	5.290538	-6.107087	-2.338682
H	7.993141	-2.841392	-1.349417
H	7.583886	-5.289928	-1.751892
C	1.862104	1.734015	2.627145
C	3.165191	2.165712	2.976014
C	0.828647	2.662674	2.455548
C	3.438508	3.528499	3.139692
C	1.100097	4.032961	2.650573
H	-0.182076	2.330375	2.174928
C	2.395521	4.465194	2.975884
H	4.454950	3.863342	3.402194
H	0.287356	4.766511	2.550479
H	2.596589	5.539645	3.106010
C	2.263185	-0.408016	3.753542
C	1.567548	-1.314018	4.562283
C	3.564606	0.023644	4.104447
C	2.179147	-1.788970	5.741390
H	0.556114	-1.650433	4.281606

C	4.171405	-0.449481	5.273326
C	3.470072	-1.359067	6.093309
H	1.640968	-2.499326	6.388081
H	5.184736	-0.115770	5.547789
H	3.940148	-1.734383	7.015675
C	-2.694883	0.063250	-3.718344
C	-1.752692	0.728865	-4.508852
C	-4.039595	0.505878	-3.678897
C	-2.152474	1.868916	-5.246821
H	-0.713433	0.366846	-4.558103
C	-4.429991	1.648217	-4.387344
C	-3.478971	2.334308	-5.171029
H	-1.430220	2.373921	-5.909095
H	-5.473864	1.996848	-4.345332
H	-3.782843	3.222331	-5.746934
C	-3.423611	-2.247429	-3.421524
C	-3.090362	-3.526443	-3.879618
C	-4.772002	-1.814579	-3.413816
C	-4.116713	-4.381485	-4.335028
H	-2.040636	-3.861189	-3.881937
C	-5.789177	-2.661185	-3.868074
C	-5.454488	-3.952010	-4.330360
H	-3.865606	-5.390779	-4.696728
H	-6.837512	-2.321666	-3.866436
H	-6.248125	-4.625111	-4.690434
C	-2.606685	0.201283	3.173610
C	-3.962619	0.605250	3.159955
C	-1.656641	0.934274	3.892691
C	-4.358743	1.773483	3.820375
C	-2.053272	2.114668	4.555132
H	-0.611411	0.596813	3.950365
C	-3.392671	2.536567	4.510437
H	-5.414309	2.088514	3.803852
H	-1.303834	2.702733	5.107210
H	-3.695817	3.458075	5.032034
C	-3.275867	-2.146627	2.952646
C	-2.890852	-3.413480	3.404083
C	-4.636452	-1.753479	2.973050
C	-3.878684	-4.298145	3.887047
H	-1.833197	-3.719207	3.366913
C	-5.614641	-2.630597	3.454678
C	-5.228588	-3.908869	3.913940
H	-3.587448	-5.298940	4.242161
H	-6.673401	-2.325545	3.475270
H	-5.992590	-4.604135	4.295622
O	-6.324029	0.087119	-0.216594
C	-7.201813	-1.048658	-0.189708
H	-8.236938	-0.657328	-0.170475
H	-7.063841	-1.689791	-1.090978
H	-7.025460	-1.676330	0.714148
O	6.453541	0.556717	1.169263
C	6.734047	1.902148	0.769291

H	7.779404	2.117033	1.062703
H	6.628936	2.027548	-0.333933
H	6.055489	2.627814	1.275797
C	1.485133	-3.954380	0.443424
C	2.737466	-4.525011	0.653032
C	0.312439	-4.781174	0.559765
C	2.799951	-5.916151	0.980989
H	3.661832	-3.935325	0.562473
C	0.354040	-6.149342	0.898897
C	1.657776	-6.711210	1.107886
H	3.790395	-6.369291	1.140969
H	1.755133	-7.776401	1.371594
C	-0.908125	-4.061267	0.308806
C	-2.123487	-4.729433	0.423147
C	-2.101656	-6.115017	0.777340
H	-3.080756	-4.212393	0.259516
C	-0.913169	-6.814104	1.005275
H	-3.062177	-6.643780	0.875421
H	-0.945999	-7.881889	1.274418
C	5.975477	-0.784504	-1.486128
C	-4.944491	-0.400375	-2.841100
C	-4.866766	-0.354215	2.382196
C	4.165254	1.008852	3.092981
H	6.984600	-0.426594	-1.214675
H	5.187475	1.332509	3.358582
H	-5.926733	-0.043658	2.379750
H	-5.994145	-0.057664	-2.813127
Cu	0.097533	1.315394	-0.641139
C	-0.037292	3.276741	-0.742658
C	1.093425	4.193441	-0.786613
C	2.433191	3.797175	-1.300539
H	3.042334	4.678600	-1.557559
C	2.777483	2.486665	-1.997017
C	3.193077	2.679698	-0.574794
H	2.628992	2.146055	0.205161
H	4.248203	2.861715	-0.326953
H	1.974484	1.759952	-2.209023
H	3.543809	2.528754	-2.786995
O	-0.078873	2.973399	-3.341178
C	-1.232726	3.796739	-0.217141
C	-1.273656	5.089925	0.392095
C	-0.131281	5.945174	0.150957
C	-2.345315	5.613246	1.175341
N	-0.102643	7.248336	0.544704
C	-2.264682	6.924788	1.619267
C	-1.131285	7.707043	1.248155
H	-3.205308	4.980342	1.437272
H	-3.058741	7.365392	2.240788
H	-1.075700	8.765641	1.563711
N	0.988279	5.490001	-0.485617
H	0.162948	3.973874	-3.442548
H	-0.116581	2.811269	-2.094117

C	-2.529785	3.003757	-0.327217
F	-2.835590	2.381160	0.841208
F	-3.579749	3.791379	-0.656401
F	-2.454056	2.044580	-1.295248
H	-0.986287	2.808470	-3.702933
O	0.648606	5.469794	-3.427970
H	1.512849	5.577177	-3.871560
H	0.817079	5.787546	-2.503548
4G g→h +3H_2O			
Cu4RX-OCF3ALKIPh--2NpostHint3--PROD+3H2O SCF Done: -4749.50807827 A.U.			
N	-1.122432	-1.308460	-0.220782
C	-2.558538	-0.810704	2.375526
H	-1.554380	-1.274865	2.365051
C	-3.056742	-0.422772	0.979604
C	-2.425556	-0.709732	-0.241007
C	0.060949	-0.594728	-0.180965
C	-3.094595	-0.476060	-1.450170
C	-2.637546	-0.901319	-2.847095
H	-1.637863	-1.374133	-2.849592
C	-4.345439	0.146325	0.986486
C	-5.013206	0.428688	-0.220950
C	-4.377117	0.107658	-1.436034
C	-0.883665	-2.660430	-0.062999
C	0.494280	-2.815809	0.080073
N	1.045681	-1.546849	0.010378
C	2.412456	-1.233358	0.302900
C	2.732898	-0.643931	1.537796
C	3.430405	-1.542372	-0.609892
C	4.076771	-0.367760	1.860147
C	4.772721	-1.259326	-0.284342
C	5.110569	-0.677404	0.953924
C	1.746105	-0.172275	2.607614
H	0.694369	-0.375587	2.338869
C	3.257627	-2.152638	-2.002120
H	2.202091	-2.366145	-2.252616
C	3.912911	-1.158483	-2.965902
C	3.309102	-0.549125	-4.071264
C	5.261737	-0.878319	-2.636603
C	4.058441	0.354472	-4.854754
H	2.259610	-0.770983	-4.322767
C	6.003184	0.023300	-3.409218
C	5.394704	0.641422	-4.523950
H	3.593990	0.832985	-5.731423
H	7.053424	0.239221	-3.155106
H	5.975655	1.343581	-5.142364
C	4.140552	-3.404477	-2.009809
C	3.726403	-4.713297	-2.282462
C	5.488544	-3.128287	-1.679099
C	4.671733	-5.758467	-2.219938

H	2.674107	-4.922788	-2.530943
C	6.426278	-4.165600	-1.617035
C	6.010433	-5.486272	-1.889342
H	4.356808	-6.792089	-2.432205
H	7.474876	-3.952042	-1.355736
H	6.742166	-6.307879	-1.842972
C	2.029612	1.326035	2.771610
C	3.381499	1.595556	3.098690
C	1.120071	2.374621	2.586063
C	3.826406	2.916152	3.227008
C	1.566378	3.704078	2.741062
H	0.070222	2.167161	2.327136
C	2.909512	3.974580	3.046529
H	4.880384	3.125964	3.470676
H	0.852178	4.532194	2.624277
H	3.248767	5.017104	3.146335
C	2.180998	-0.840063	3.911412
C	1.386884	-1.640678	4.740546
C	3.529753	-0.570746	4.242996
C	1.948482	-2.173827	5.919570
H	0.338150	-1.851124	4.474569
C	4.087300	-1.101893	5.411739
C	3.287653	-1.905733	6.251466
H	1.333440	-2.802631	6.582008
H	5.137397	-0.893679	5.671591
H	3.718048	-2.325816	7.173911
C	-2.714794	0.340776	-3.733227
C	-1.695365	0.845846	-4.547882
C	-3.983881	0.965819	-3.701809
C	-1.933779	2.014388	-5.307918
H	-0.717427	0.341677	-4.588410
C	-4.218140	2.128661	-4.445990
C	-3.183780	2.657822	-5.245410
H	-1.143485	2.411568	-5.965358
H	-5.204583	2.617734	-4.414197
H	-3.362885	3.566887	-5.840410
C	-3.749591	-1.840075	-3.338368
C	-3.601999	-3.176757	-3.723511
C	-5.025729	-1.225416	-3.344295
C	-4.742114	-3.905182	-4.125755
H	-2.609088	-3.654132	-3.708966
C	-6.155023	-1.946727	-3.746866
C	-6.007394	-3.294625	-4.139770
H	-4.636904	-4.957960	-4.431026
H	-7.146971	-1.466785	-3.756756
H	-6.890738	-3.869468	-4.458946
C	-2.631555	0.446592	3.241152
C	-3.910269	1.051703	3.226170
C	-1.597684	0.992466	4.009662
C	-4.140598	2.232790	3.940863
C	-1.827232	2.184619	4.727496
H	-0.616141	0.498494	4.063862

C	-3.087061	2.805373	4.685005
H	-5.136071	2.704677	3.925426
H	-1.011575	2.624047	5.322391
H	-3.260379	3.734698	5.250210
C	-3.643098	-1.761806	2.898761
C	-3.465521	-3.096151	3.278654
C	-4.928603	-1.166995	2.924122
C	-4.585848	-3.843196	3.701798
H	-2.466662	-3.559285	3.234718
C	-6.037714	-1.907564	3.347498
C	-5.860242	-3.252607	3.739387
H	-4.457749	-4.894817	4.002133
H	-7.037876	-1.445482	3.373337
H	-6.728324	-3.841441	4.074699
O	-6.283073	0.959758	-0.213615
C	-7.320310	-0.032087	-0.191107
H	-8.285180	0.510130	-0.187108
H	-7.270862	-0.693767	-1.086464
H	-7.251782	-0.672528	0.718193
O	6.426370	-0.426729	1.272012
C	6.886090	0.877118	0.902857
H	7.954592	0.935844	1.185390
H	6.786604	1.049120	-0.194395
H	6.323136	1.676723	1.438804
C	0.816792	-4.221884	0.312467
C	1.955615	-4.994504	0.521798
C	-0.470910	-4.866056	0.303346
C	1.791602	-6.402018	0.719601
H	2.960946	-4.548099	0.527762
C	-0.653004	-6.248799	0.511227
C	0.538685	-7.020579	0.720459
H	2.692965	-7.013677	0.878742
H	0.461439	-8.107137	0.885095
C	-1.555583	-3.947272	0.081771
C	-2.862114	-4.426668	0.082256
C	-3.066285	-5.825944	0.297379
H	-3.719644	-3.754335	-0.070113
C	-2.009267	-6.717331	0.502174
H	-4.098466	-6.208640	0.301953
H	-2.215668	-7.787417	0.663397
C	5.747548	-1.642002	-1.398683
C	-4.996654	0.221891	-2.828941
C	-4.932329	0.275716	2.392731
C	4.233251	0.325057	3.216546
H	6.797616	-1.429189	-1.130545
H	5.290183	0.523130	3.468673
H	-5.932803	0.743764	2.390076
H	-5.989467	0.705502	-2.810773
Cu	0.285060	1.261385	-0.418132
C	0.427659	3.234271	-0.642615
C	1.686182	3.960138	-0.684561
C	2.964331	3.354528	-1.144123

H	3.702707	4.119813	-1.429387
C	3.130073	1.982972	-1.780983
C	3.532004	2.169975	-0.353344
H	2.875462	1.750812	0.423689
H	4.593991	2.211221	-0.075793
H	2.241102	1.368491	-1.998443
H	3.919527	1.885322	-2.542908
O	0.343622	2.516288	-3.165440
C	-0.684144	3.930871	-0.139356
C	-0.545819	5.234827	0.432515
C	0.720327	5.896213	0.211001
C	-1.541594	5.948161	1.166000
N	0.942123	7.194377	0.556615
C	-1.270435	7.247648	1.568874
C	-0.023124	7.835244	1.205870
H	-2.498962	5.470875	1.417339
H	-2.004347	7.827630	2.148357
H	0.183020	8.886919	1.478404
N	1.774910	5.251675	-0.363144
H	0.950334	3.309747	-3.509365
H	0.284624	2.640996	-1.862679
C	-2.068138	3.294113	-0.213454
F	-2.391001	2.692556	0.962867
F	-3.037744	4.194730	-0.491479
F	-2.135254	2.346198	-1.191307
H	-0.559325	2.589923	-3.552357
O	1.938697	4.299733	-3.969751
H	2.769945	3.797411	-4.073991
H	2.236481	5.114707	-3.428877
O	2.980987	6.240591	-2.542656
H	2.676752	6.094723	-1.590466
H	2.826672	7.187161	-2.720103

4G h

Cu4RX-OCF3ALKIPh--2NpostHPROD+ SCF Done: -4520.36252748 A.U.

N	-0.922485	1.134048	-0.558714
C	-2.958159	-0.038817	-2.500413
H	-1.914850	0.140282	-2.821178
C	-3.205803	0.337859	-1.039329
C	-2.272519	0.877125	-0.145683
C	0.151197	0.307759	-0.282086
C	-2.669581	1.243134	1.150036
C	-1.793425	1.892440	2.221903
H	-0.747788	2.044324	1.894406
C	-4.538043	0.135855	-0.624492
C	-4.950940	0.496207	0.673519
C	-4.004287	1.054616	1.555897
C	-0.498291	2.314563	-1.135140
C	0.891635	2.244546	-1.222963
N	1.265049	1.019511	-0.700205

C	2.611825	0.537217	-0.628253
C	2.999106	-0.542918	-1.433667
C	3.537385	1.125769	0.248403
C	4.285081	-1.102330	-1.296146
C	4.832228	0.576276	0.362801
C	5.211880	-0.554809	-0.388141
C	2.138392	-1.247254	-2.478518
H	1.135954	-0.796798	-2.594359
C	3.275669	2.266049	1.238064
H	2.262465	2.699542	1.146796
C	3.535431	1.642123	2.612244
C	2.626058	1.563454	3.671357
C	4.828452	1.076184	2.729039
C	2.993923	0.878800	4.849797
H	1.637959	2.042877	3.586617
C	5.197761	0.404065	3.900119
C	4.269540	0.298656	4.959878
H	2.279863	0.808382	5.685431
H	6.202048	-0.039541	3.992409
H	4.553951	-0.230094	5.883016
C	4.385731	3.292495	1.005784
C	4.214106	4.647986	0.699426
C	5.681939	2.738005	1.122283
C	5.353365	5.454742	0.499647
H	3.202643	5.074043	0.608201
C	6.812682	3.539177	0.922544
C	6.642077	4.903857	0.608854
H	5.230498	6.522180	0.258473
H	7.822272	3.107147	1.008698
H	7.526206	5.541001	0.451098
C	2.079428	-2.703501	-2.024300
C	3.360107	-3.287473	-1.871570
C	0.919146	-3.453732	-1.799250
C	3.478749	-4.626545	-1.479692
C	1.044702	-4.814821	-1.455122
H	-0.076630	-2.997327	-1.921125
C	2.311536	-5.392742	-1.277495
H	4.473490	-5.081559	-1.348727
H	0.138143	-5.423760	-1.333626
H	2.391633	-6.444365	-0.965308
C	2.956212	-1.235610	-3.773803
C	2.545921	-0.726438	-5.010754
C	4.236976	-1.817807	-3.623436
C	3.428030	-0.799630	-6.108960
H	1.546477	-0.276145	-5.124023
C	5.113205	-1.889168	-4.712168
C	4.701047	-1.376290	-5.960069
H	3.116109	-0.403091	-7.087754
H	6.111480	-2.339691	-4.594286
H	5.383285	-1.429162	-6.822794
C	-1.906130	0.981219	3.444236
C	-0.849249	0.353382	4.112784

C	-3.242748	0.779029	3.862210
C	-1.127636	-0.487313	5.212128
H	0.186669	0.510427	3.776899
C	-3.519880	-0.057084	4.949615
C	-2.455594	-0.692592	5.624309
H	-0.300583	-0.985427	5.742539
H	-4.560614	-0.222981	5.269795
H	-2.668832	-1.351110	6.480645
C	-2.507411	3.199897	2.581257
C	-1.971675	4.489675	2.501684
C	-3.843593	2.999219	3.004491
C	-2.779445	5.591351	2.854813
H	-0.933617	4.641205	2.164399
C	-4.644085	4.091987	3.355777
C	-4.104193	5.393719	3.279618
H	-2.367876	6.611073	2.796991
H	-5.682598	3.935725	3.689312
H	-4.726566	6.259177	3.555710
C	-3.376083	-1.505682	-2.609493
C	-4.705989	-1.733859	-2.184554
C	-2.566481	-2.569990	-3.020596
C	-5.223498	-3.034162	-2.162490
C	-3.082450	-3.882522	-2.986731
H	-1.542065	-2.380577	-3.378964
C	-4.402405	-4.111170	-2.559281
H	-6.257148	-3.214286	-1.827070
H	-2.450177	-4.725844	-3.306353
H	-4.804580	-5.136576	-2.543579
C	-3.978994	0.784977	-3.292506
C	-3.694868	1.720681	-4.293326
C	-5.314043	0.549158	-2.883579
C	-4.759179	2.420216	-4.901032
H	-2.653101	1.910495	-4.597631
C	-6.369513	1.240470	-3.489488
C	-6.085703	2.178867	-4.504881
H	-4.547163	3.157036	-5.691434
H	-7.409081	1.053552	-3.175336
H	-6.910679	2.725433	-4.987928
O	-6.253820	0.309705	1.071696
C	-7.101612	1.436224	0.803435
H	-8.113764	1.172615	1.165231
H	-6.743591	2.348067	1.335167
H	-7.144390	1.660508	-0.287527
O	6.449860	-1.125787	-0.213844
C	6.465369	-2.166385	0.775080
H	7.502145	-2.550205	0.822600
H	6.163870	-1.780189	1.775831
H	5.773266	-2.996244	0.503966
C	1.417688	3.471850	-1.815697
C	2.660924	4.003143	-2.147551
C	0.234410	4.250882	-2.072093
C	2.704913	5.308687	-2.730648

H	3.592852	3.449148	-1.961157
C	0.257957	5.533514	-2.658142
C	1.553102	6.057653	-2.984767
H	3.688445	5.732024	-2.985999
H	1.636261	7.057302	-3.440198
C	-0.976485	3.580952	-1.678524
C	-2.200449	4.207425	-1.896503
C	-2.195495	5.506333	-2.494820
H	-3.149885	3.720937	-1.626600
C	-1.016075	6.160322	-2.862346
H	-3.161171	6.005271	-2.669258
H	-1.062103	7.162758	-3.316862
C	5.669557	1.243015	1.455471
C	-4.261824	1.522898	2.991142
C	-5.418674	-0.455587	-1.729106
C	4.503933	-2.317207	-2.197270
H	6.680123	0.804281	1.532955
H	5.508844	-2.758542	-2.074435
H	-6.454240	-0.634835	-1.389208
H	-5.306547	1.353678	3.307367
Cu	-0.018908	-1.369943	0.591421
C	-0.492923	-2.557337	2.230373
C	0.842729	-3.121438	2.346647
C	1.945143	-2.251743	2.823948
H	1.633659	-1.326637	3.334882
C	3.149659	-2.117713	1.870327
C	3.295853	-2.850000	3.168609
H	3.348990	-3.949623	3.133266
H	3.850876	-2.352370	3.979188
H	3.092816	-2.700024	0.934552
H	3.570772	-1.107669	1.775224
C	-1.483966	-3.320234	1.584531
C	-1.176025	-4.631185	1.111117
C	0.135144	-5.153407	1.450304
C	-2.083189	-5.479214	0.407988
N	0.460121	-6.461861	1.242445
C	-1.707978	-6.793655	0.176204
C	-0.437269	-7.240369	0.652446
H	-3.053856	-5.094787	0.061906
H	-2.372138	-7.490487	-0.356600
H	-0.150966	-8.299743	0.514771
N	1.107767	-4.374719	1.996784
C	-2.919298	-2.814467	1.533419
F	-3.310642	-2.622852	0.247029
F	-3.770730	-3.703976	2.092683
F	-3.059744	-1.642427	2.186488
H	-0.783044	-1.678382	2.836882

1. References

1. Agilent Technologies, *CrysAlisPro*, Version 1.171.40.45a.
2. Sheldrick, G. M., Crystal structure refinement withSHELXL. *Acta Crystallographica Section C Structural Chemistry* **2015,** *C71*, 3-8.
3. Farrugia, L., WinGX suite for small-molecule single-crystal crystallography. *J. Appl. Crystallogr.* **1999,** *32*, 837-838.
4. Barbour, L. J., X-Seed — A Software Tool for Supramolecular Crystallography. *J. Supramol. Chem.* **2001,** *1*, 189-191.
5. Frisch, M. J.; Trucks, G. W.; Schlegel, H. B.; Scuseria, G. E.; Robb, M. A.; Cheeseman, J. R.; Scalmani, G.; Barone, V.; Petersson, G. A.; Nakatsuji, H.; Li, X.; Caricato, M.; Marenich, A. V.; Bloino, J.; Janesko, B. G.; Gomperts, R.; Mennucci, B.; Hratchian, H. P.; Ortiz, J. V.; Izmaylov, A. F.; Sonnenberg, J. L.; Williams-Young, D.; Ding, F.; Lipparini, F.; Egidi, F.; Goings, J.; Peng, B.; Petrone, A.; Henderson, T.; Ranasinghe, D.; Zakrzewski, V. G.; Gao, J.; Rega, N.; Zheng, G.; Liang, W.; Hada, M.; Ehara, M.; Toyota, K.; Fukuda, R.; Hasegawa, J.; Ishida, M.; Nakajima, T.; Honda, Y.; Kitao, O.; Nakai, H.; Vreven, T.; Throssell, K.; Montgomery, J. A. J.; Peralta, J. E.; Ogliaro, F.; Bearpark, M. J.; Heyd, J. J.; Brothers, E. N.; Kudin, K. N.; Staroverov, V. N.; Keith, T. A.; Kobayashi, R.; Normand, J.; Raghavachari, K.; Rendell, A. P.; Burant, J. C.; Iyengar, S. S.; Tomasi, J.; Cossi, M.; Millam, J. M.; Klene, M.; Adamo, C.; Cammi, R.; Ochterski, J. W.; Martin, R. L.; Morokuma, K.; Farkas, O.; Foresman, J. B.; Fox, D. J., Gaussian 16, Revision C.01, Gaussian, Inc., Wallingford CT, 2016., Gaussian 16, Revision C.01, Gaussian, Inc., Wallingford CT, 2016.
6. (a) Becke, A. D., Density-functional exchange-energy approximation with correct asymptotic behavior. *Phys. Rev. A* **1988,** *38*, 3098-3100. (b) Perdew, J. P., Density-functional approximation for the correlation energy of the inhomogeneous electron gas. *Phys. Rev. B* **1986,** *33*, 8822-8824.
7. Grimme, S.; Antony, J.; Ehrlich, S.; Krieg, H., A consistent and accurate ab initio parametrization of density functional dispersion correction (DFT-D) for the 94 elements H-Pu. *J. Chem. Phys.* **2010,** *132*, 154104.
8. (a) Weigend, F.; Ahlrichs, R., Balanced basis sets of split valence, triple zeta valence and quadruple zeta valence quality for H to Rn: Design and assessment of accuracy. *Phys. Chem. Chem. Phys.* **2005,** *7*, 3297-3305. (b) Weigend, F., Accurate Coulomb-fitting basis sets for H to Rn. *Phys. Chem. Chem. Phys.* **2006,** *8*, 1057-1065.
9. (a) Häussermann, U.; Dolg, M.; Stoll, H.; Preuss, H.; Schwerdtfeger, P.; Pitzer, R. M., Accuracy of energy-adjusted quasirelativistic ab initio pseudopotentials. *Mol. Phys.* **1993,** *78*, 1211-1224. (b) Küchle, W.; Dolg, M.; Stoll, H.; Preuss, H., Energy-adjusted pseudopotentials for the actinides. Parameter sets and test calculations for thorium and thorium monoxide. *J. Chem. Phys.* **1994,** *100*, 7535-7542. (c) Leininger, T.; Nicklass, A.; Stoll, H.; Dolg, M.; Schwerdtfeger, P., The accuracy of the pseudopotential approximation. II. A comparison of various core sizes for indium pseudopotentials in calculations for spectroscopic constants of InH, InF, and InCl. *J. Chem. Phys.* **1996,** *105*, 1052-1059.
10. Marenich, A. V.; Cramer, C. J.; Truhlar, D. G., Universal Solvation Model Based on Solute Electron Density and on a Continuum Model of the Solvent Defined by the Bulk Dielectric Constant and Atomic Surface Tensions. *J. Phys. Chem. B* **2009,** *113*, 6378-6396.
11. (a) Stephens, P. J.; Devlin, F. J.; Chabalowski, C. F.; Frisch, M. J., Ab Initio Calculation of Vibrational Absorption and Circular Dichroism Spectra Using Density Functional Force Fields. *J. Phys. Chem.* **1994,** *98*, 11623-11627. (b) Becke, A. D., Density-functional thermochemistry. III. The role of exact exchange. *J. Chem. Phys.* **1993,** *98*, 5648-5652. (c) Lee, C.; Yang, W.; Parr, R. G., Development of the Colle-Salvetti correlation-energy formula into a functional of the electron density. *Phys. Rev. B* **1988,** *37*, 785-789.
12. Poater, A.; Cosenza, B.; Correa, A.; Giudice, S.; Ragone, F.; Scarano, V.; Cavallo, L., SambVca: A Web Application for the Calculation of the Buried Volume of N-Heterocyclic Carbene Ligands. *Eur. J. Inorg. Chem.* **2009**, 1759-1766.
13. Falivene, L.; Poater, A.; Cazin, C. S. J.; Slugovc, C.; Cavallo, L., Energetics of the ruthenium–halide bond in olefin metathesis (pre)catalysts. *Dalton Trans.* **2013,** *42*, 7312-7317.
