# Supplementary material for: NHC–BIAN–Cu(I)-Catalyzed Friedländer-Type Annulation of 2-Amino-3-(per)fluoroacetylpyridines with Alkynes on Water
Source: J Org Chem. 2022 Apr 8;87(9):6115–36. doi: 10.1021/acs.joc.2c00380 (PMC9087358; doi:10.1021/acs.joc.2c00380)
Supplement: Supplementary file 2 — jo2c00380_si_002.pdf [file jo2c00380_si_002.pdf]

Electronic Supplementary Information

for

NHC-BIAN-Cu(I)-Catalyzed Friedländer-Type Annulation of 2-Amino-3-(per)fluoroacetylpyridines with Alkynes on Water

Magdalena Dolna,[a] Michał Nowacki,[a] Oksana Danyluk,[b] Artur Brotons-Rufes,[c]

Albert Poater,*[c] and Michał Michalak*[a]

*e-mail: albert.poater@udg.edu, michal.michalak@icho.edu.pl

[a] Institute of Organic Chemistry, Polish Academy of Sciences, 01-224 Warsaw, Kasprzaka 44/52, Poland

[b] Institute of Physical Chemistry, Polish Academy of Sciences, 01-224 Warsaw, Kasprzaka 44/52, Poland

[c] Institut de Química Computacional i Catàlisi and Departament de Química, Universitat de Girona, Campus Montilivi, 17071 Girona, Catalonia, Spain

Tables of contents

1. Copies of NMR spectra

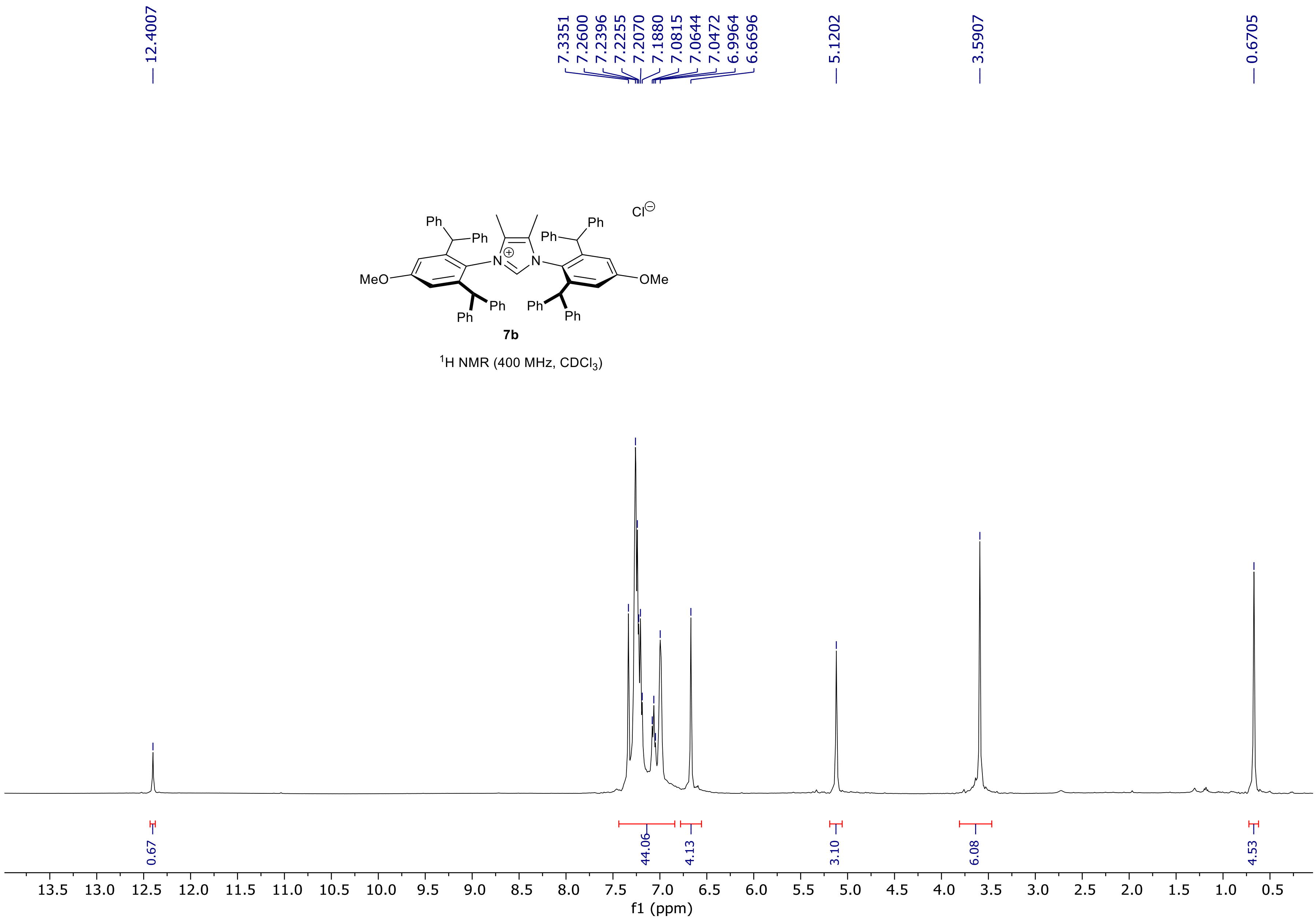
12.4007
7.3351
7.2600
7.2396
7.2255
7.2070
7.1880
7.0815
7.0644
7.0472
6.9964
6.6696
5.1202
3.5907
0.6705
Ph
Ph
Ph
Ph
Cl⊖
MeO
N
N
OMe
Ph
Ph
Ph
Ph
7b
^{1}H NMR (400 MHz, CDCl$_3$)
0.67
44.06
4.13
3.10
6.08
4.53
13.5
13.0
12.5
12.0
11.5
11.0
10.5
10.0
9.5
9.0
8.5
8.0
7.5
7.0
6.5
6.0
5.5
5.0
4.5
4.0
3.5
3.0
2.5
2.0
1.5
1.0
0.5
f1 (ppm)

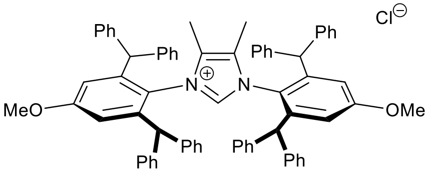

7b

^{13}C NMR (100 MHz, $CDCl_3$)

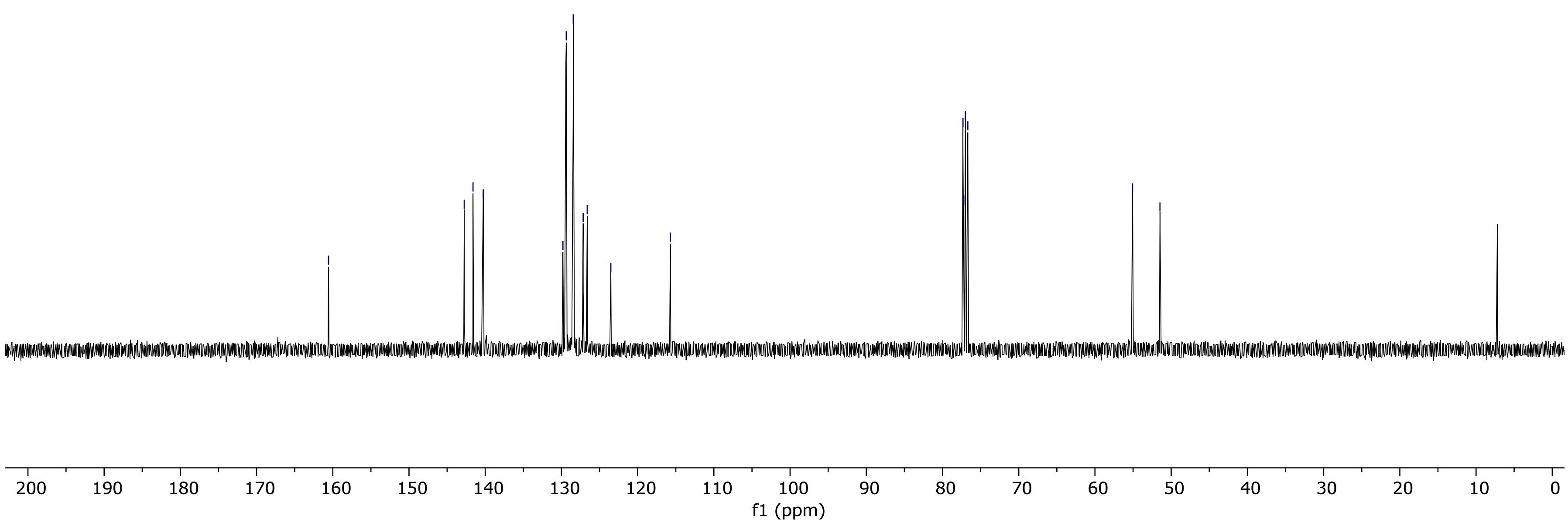

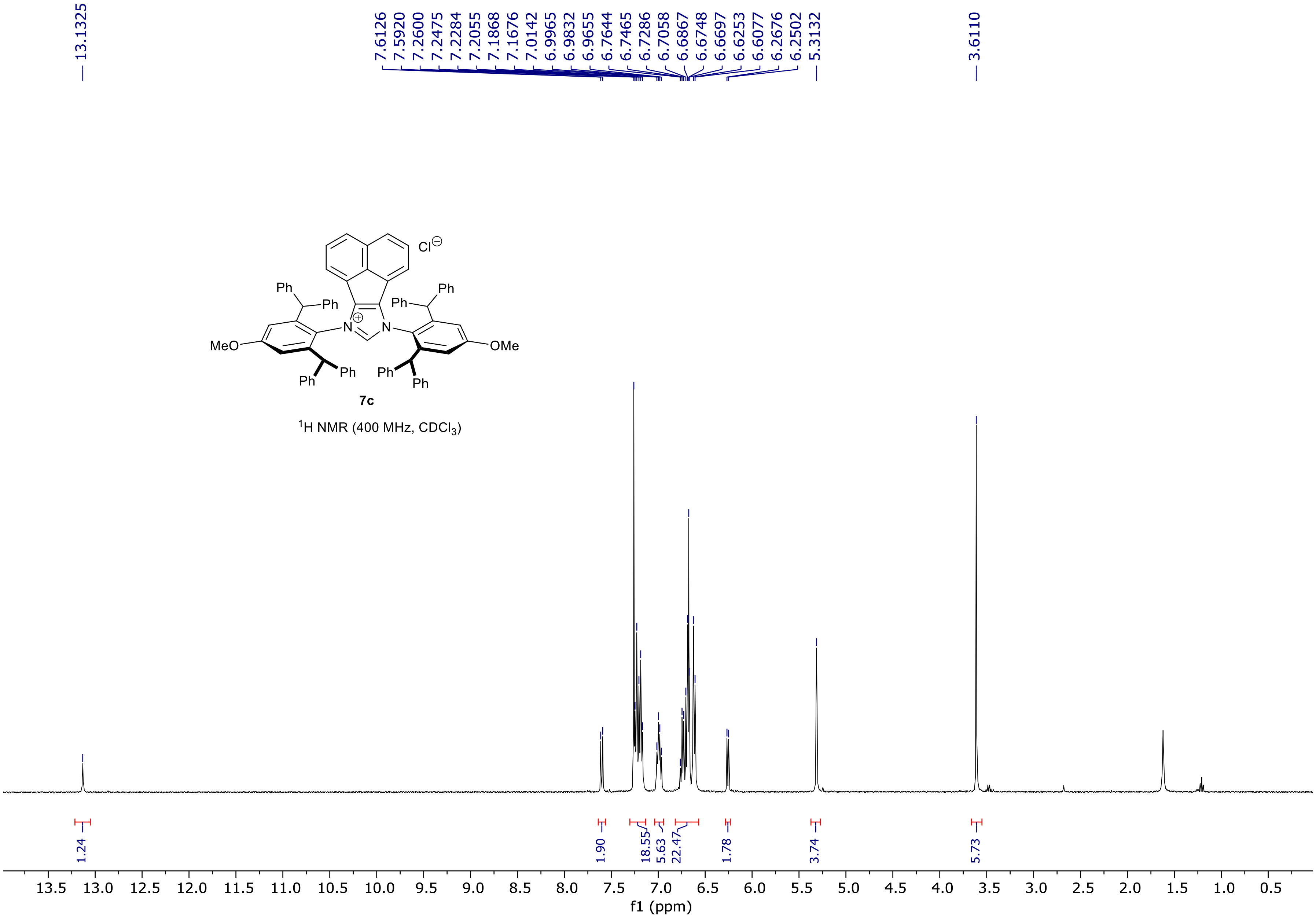
13.1325
7.6126
7.5920
7.2600
7.2475
7.2284
7.2055
7.1868
7.1676
7.0142
6.9965
6.9832
6.9655
6.7644
6.7465
6.7286
6.7058
6.6867
6.6748
6.6697
6.6253
6.6077
6.2676
6.2502
5.3132
3.6110
Cl
Ph
Ph
Ph
Ph
MeO
OMe
N
N
Ph
Ph
Ph
Ph
7c
1H NMR (400 MHz, CDCl3)
1.24
1.90
18.55
5.63
22.47
1.78
3.74
5.73
13.5 13.0 12.5 12.0 11.5 11.0 10.5 10.0 9.5 9.0 8.5 8.0 7.5 7.0 6.5 6.0 5.5 5.0 4.5 4.0 3.5 3.0 2.5 2.0 1.5 1.0 0.5
f1 (ppm)

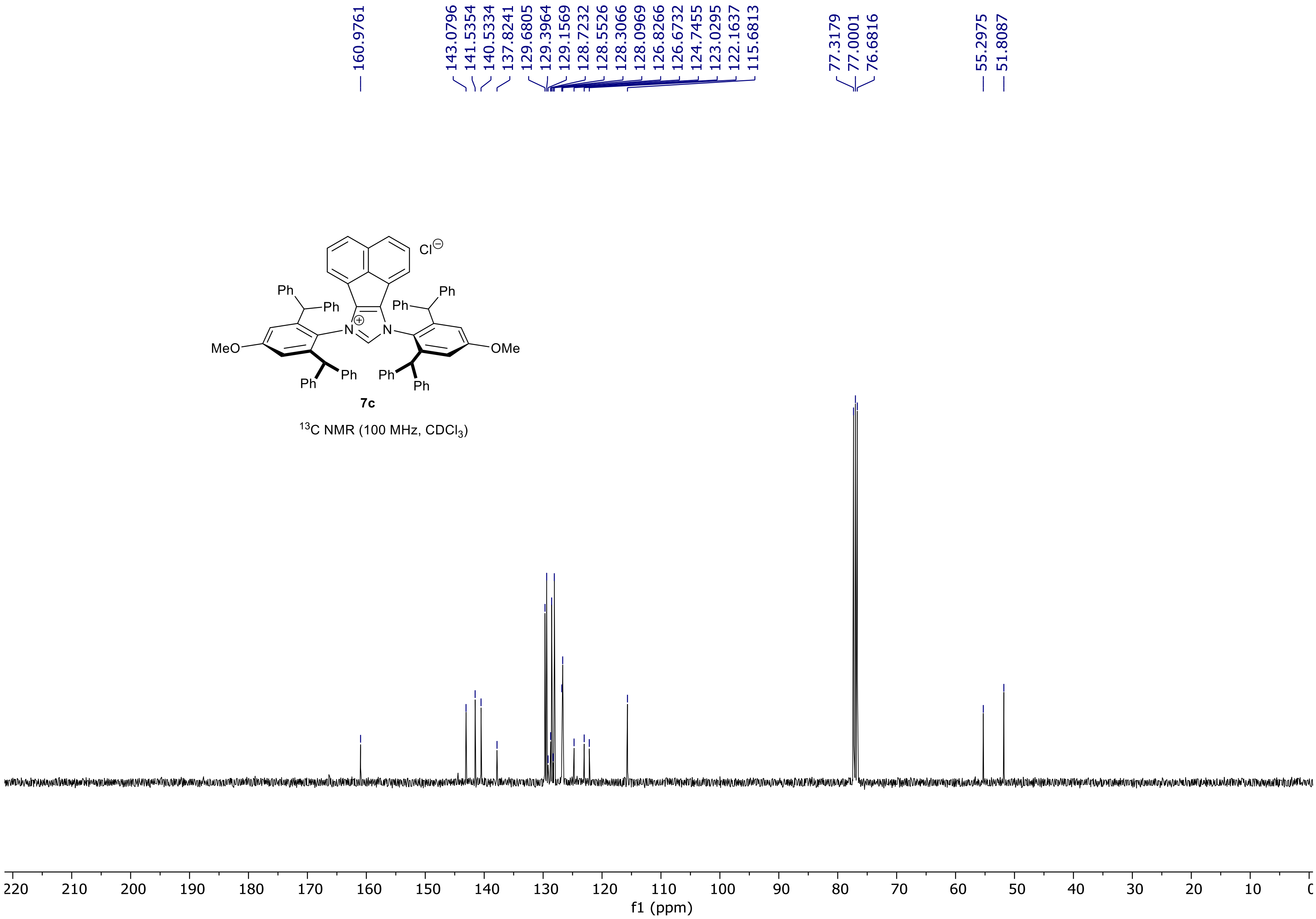
160.9761
143.0796
141.5354
140.5334
137.8241
129.6805
129.3964
129.1569
128.7232
128.5526
128.3066
128.0969
126.8266
126.6732
124.7455
123.0295
122.1637
115.6813
77.3179
77.0001
76.6816
55.2975
51.8087
Cl⊖
Ph
Ph
MeO
OMe
N
N
⊕
7c
^{13}C NMR (100 MHz, $CDCl_3$)
220 210 200 190 180 170 160 150 140 130 120 110 100 90 80 70 60 50 40 30 20 10
f1 (ppm)

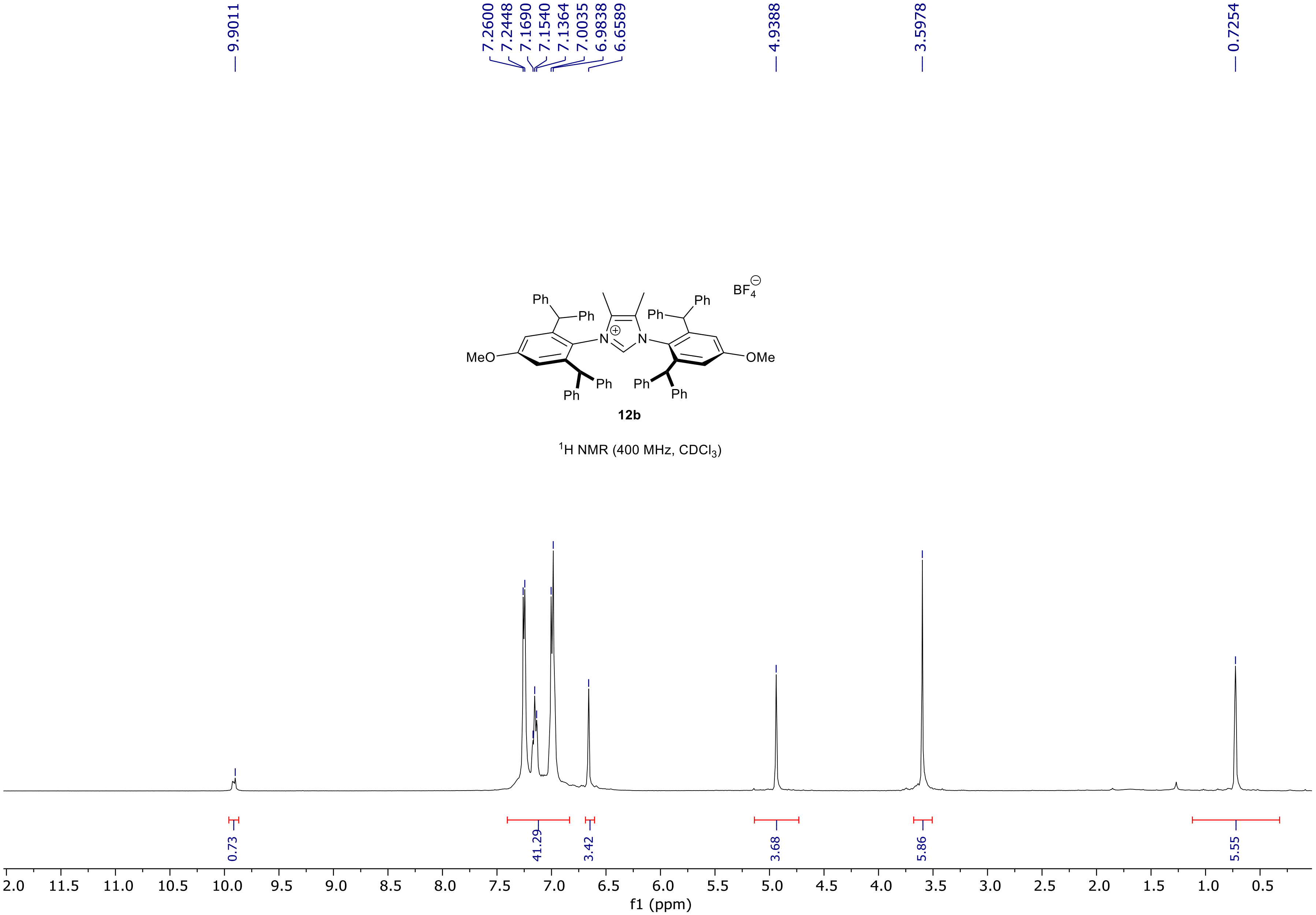
9.9011
7.2600
7.2448
7.1690
7.1540
7.1364
7.0035
6.9838
6.6589
4.9388
3.5978
0.7254
Ph
Ph
Ph
Ph
Ph
Ph
Ph
Ph
MeO
OMe
N
N
$BF_4^{\ominus}$
12b
^{1}H NMR (400 MHz, $CDCl_3$)
0.73
41.29
3.42
3.68
5.86
5.55
2.0
11.5
11.0
10.5
10.0
9.5
9.0
8.5
8.0
7.5
7.0
6.5
6.0
5.5
5.0
4.5
4.0
3.5
3.0
2.5
2.0
1.5
1.0
0.5
f1 (ppm)

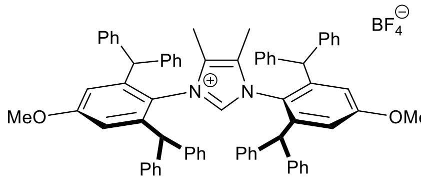

12b

^{13}C NMR (100 MHz, $CDCl_3$)

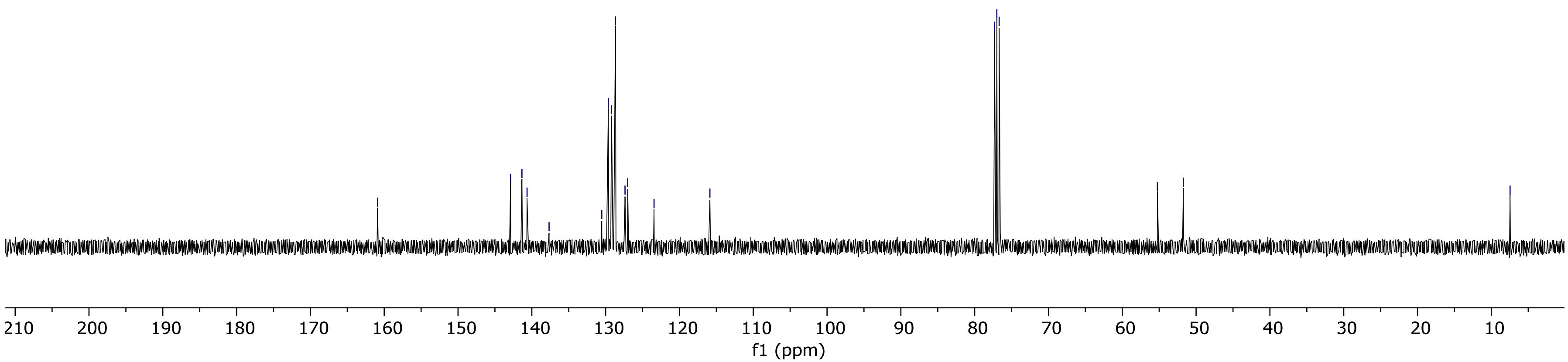

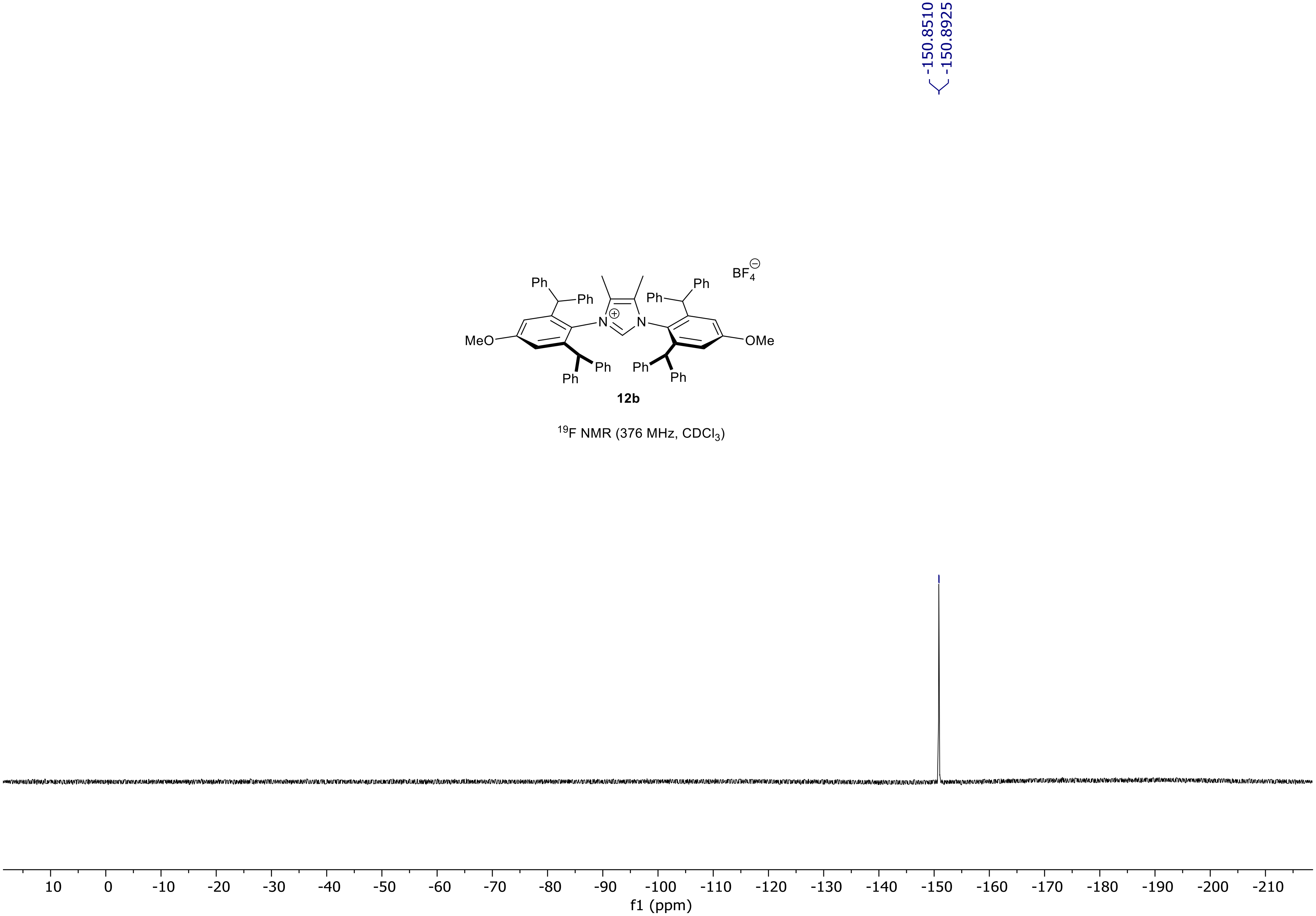
-150.8510
-150.8925
BF4
MeO
OMe
Ph
12b
19F NMR (376 MHz, CDCl3)
10 0 -10 -20 -30 -40 -50 -60 -70 -80 -90 -100 -110 -120 -130 -140 -150 -160 -170 -180 -190 -200 -210
f1 (ppm)

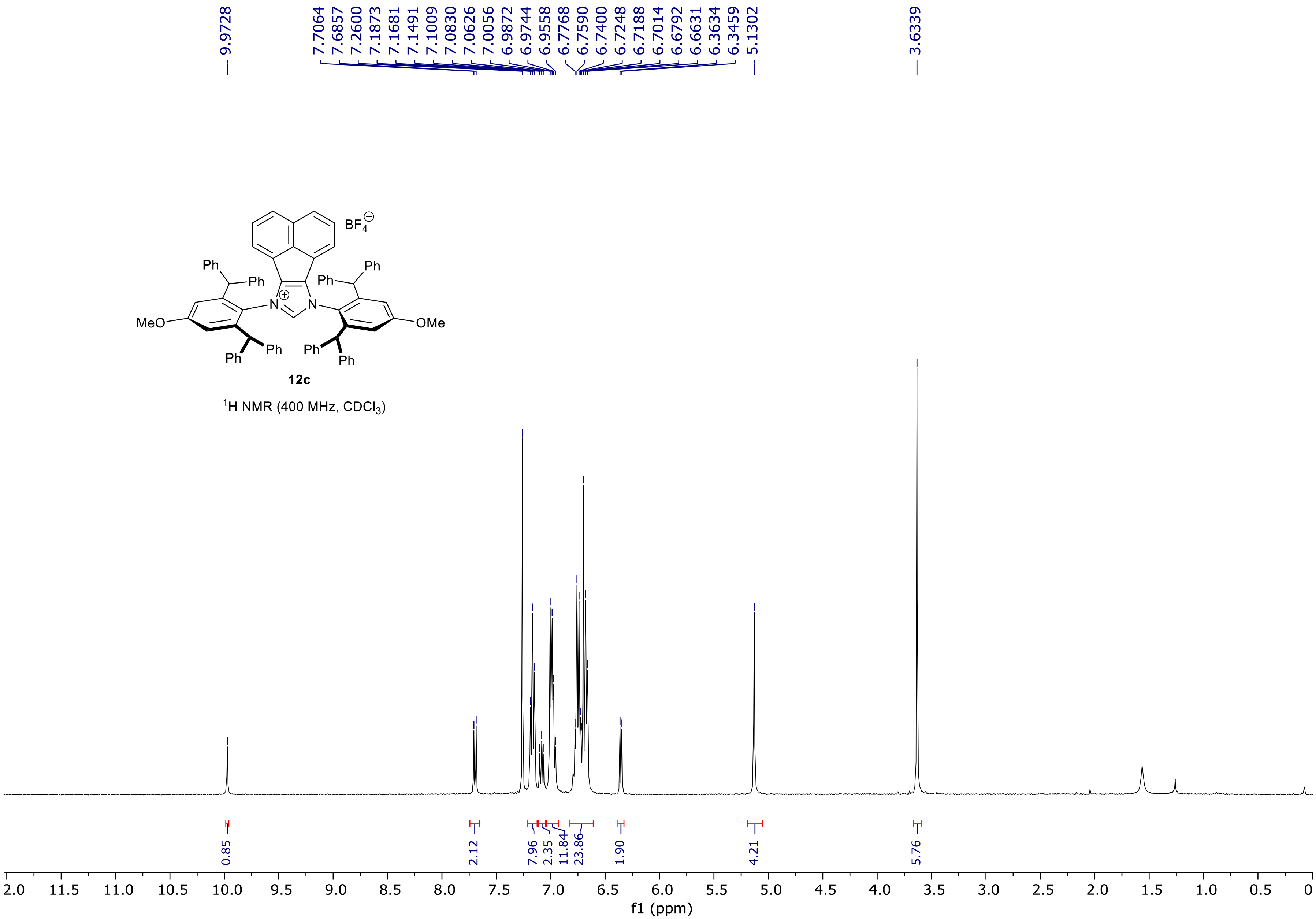
9.9728
7.7064
7.6857
7.2600
7.1873
7.1681
7.1491
7.1009
7.0830
7.0626
7.0056
6.9872
6.9744
6.9558
6.7768
6.7590
6.7400
6.7248
6.7188
6.7014
6.6792
6.6631
6.3634
6.3459
5.1302
3.6339
BF4
Ph
Ph
Ph
Ph
N
N
MeO
OMe
Ph
Ph
Ph
Ph
12c
1H NMR (400 MHz, CDCl3)
0.85
2.12
7.96
2.35
11.84
23.86
1.90
4.21
5.76
f1 (ppm)

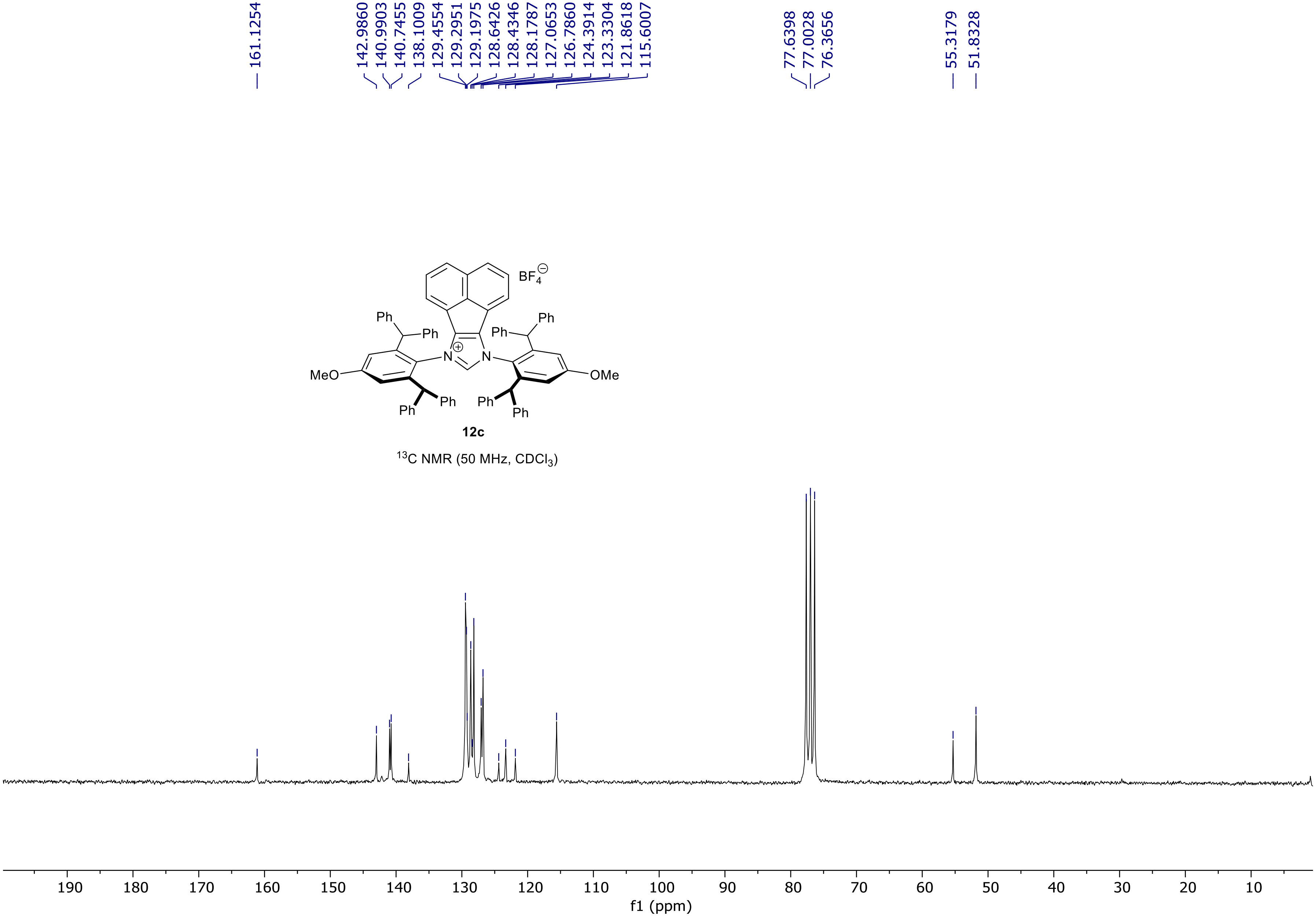

12c

^{13}C NMR (50 MHz, $CDCl_3$)

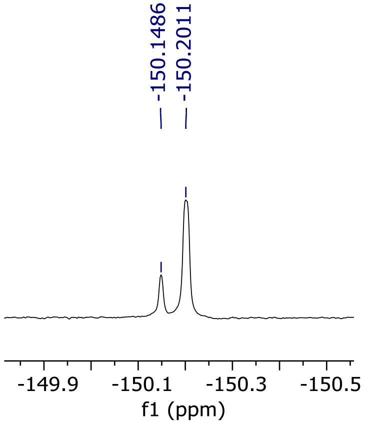

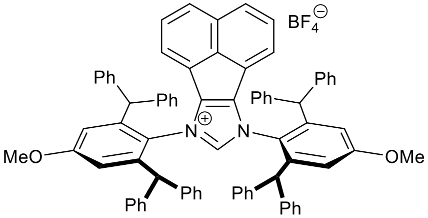

12c

^{19}F NMR (376 MHz, $CDCl_3$)

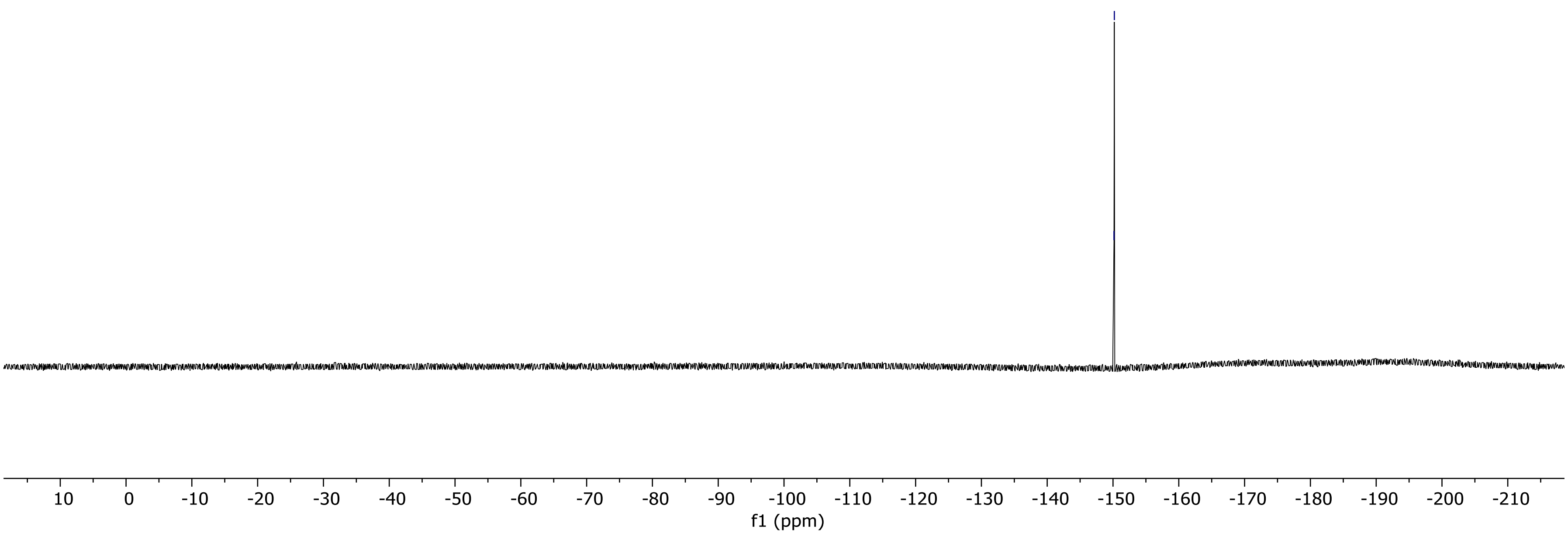

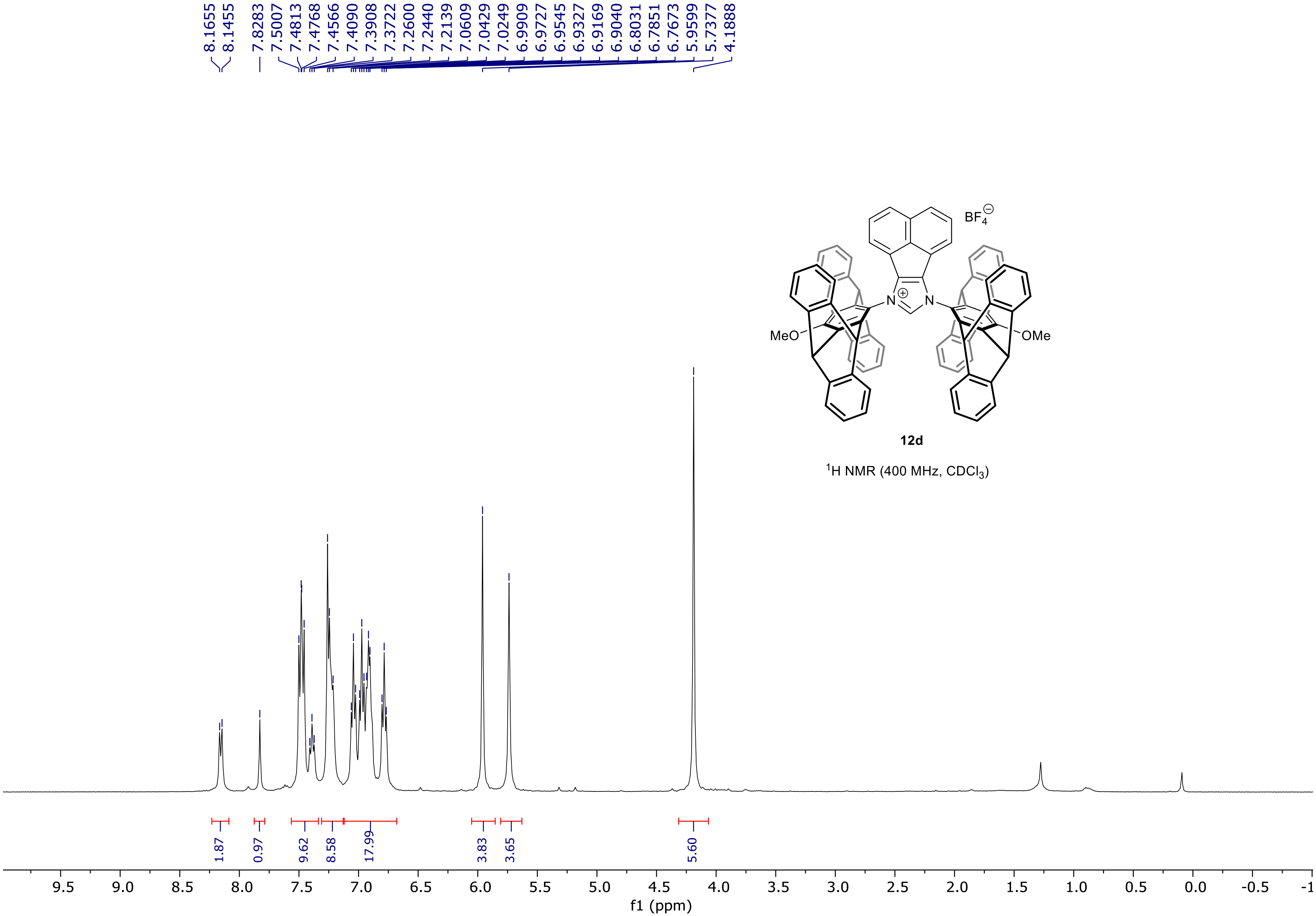
8.1655
8.1455
7.8283
7.5007
7.4813
7.4768
7.4566
7.4090
7.3908
7.3722
7.2600
7.2440
7.2139
7.0609
7.0429
7.0249
6.9909
6.9727
6.9545
6.9327
6.9169
6.9040
6.8031
6.7851
6.7673
5.9599
5.7377
4.1888
BF4
MeO
OMe
N
N
12d
1H NMR (400 MHz, CDCl3)
1.87
0.97
9.62
8.58
17.99
3.83
3.65
5.60
9.5
9.0
8.5
8.0
7.5
7.0
6.5
6.0
5.5
5.0
4.5
4.0
3.5
3.0
2.5
2.0
1.5
1.0
0.5
0.0
-0.5
-1
f1 (ppm)

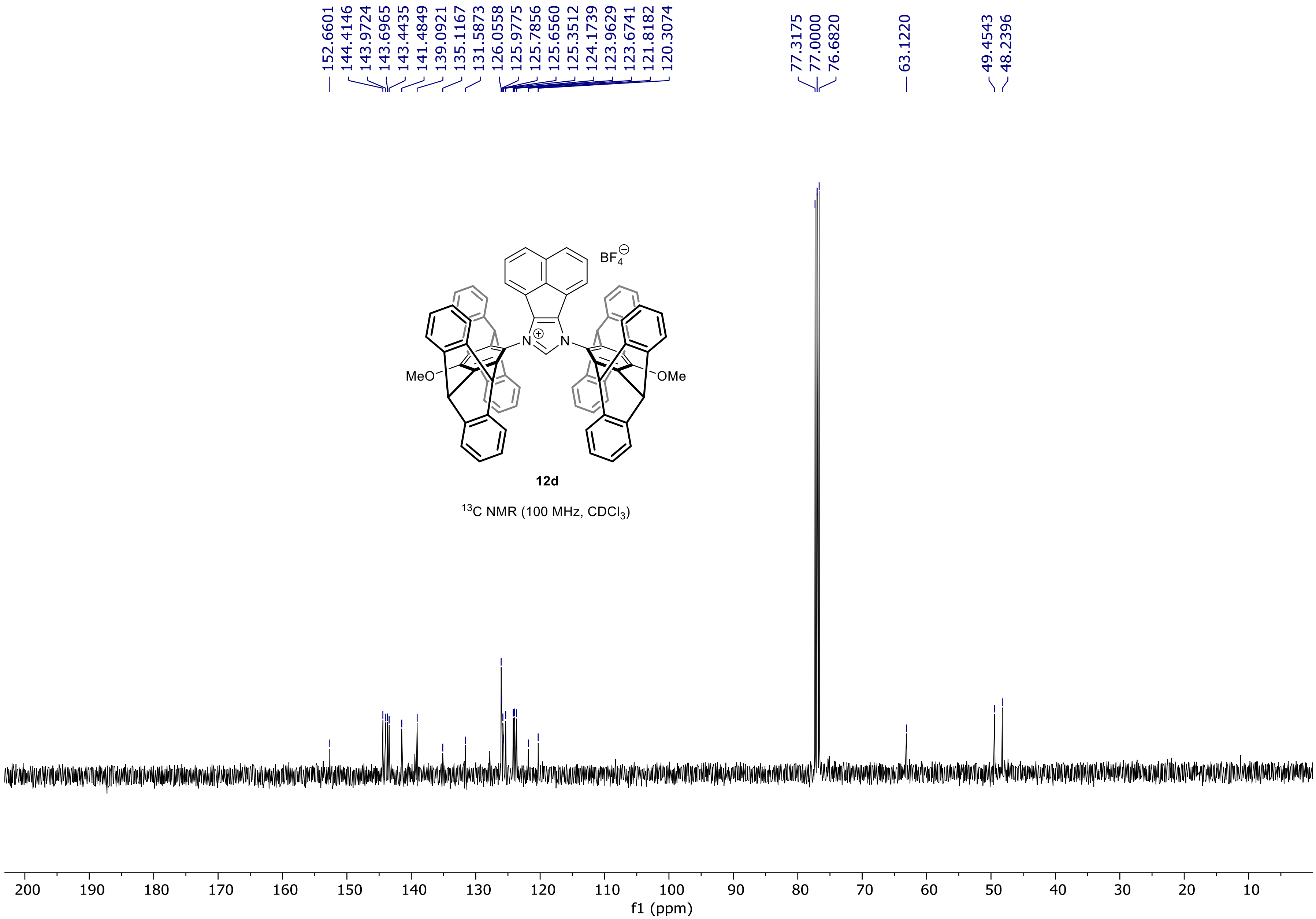
152.6601
144.4146
143.9724
143.6965
143.4435
141.4849
139.0921
135.1167
131.5873
126.0558
125.9775
125.7856
125.6560
125.3512
124.1739
123.9629
123.6741
121.8182
120.3074
77.3175
77.0000
76.6820
63.1220
49.4543
48.2396
BF4
N
N
MeO
OMe
12d
13C NMR (100 MHz, CDCl3)
200
190
180
170
160
150
140
130
120
110
100
90
80
70
60
50
40
30
20
10
f1 (ppm)

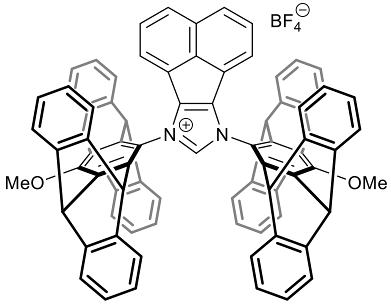

12d

^{19}F NMR (376 MHz, $CDCl_3$)

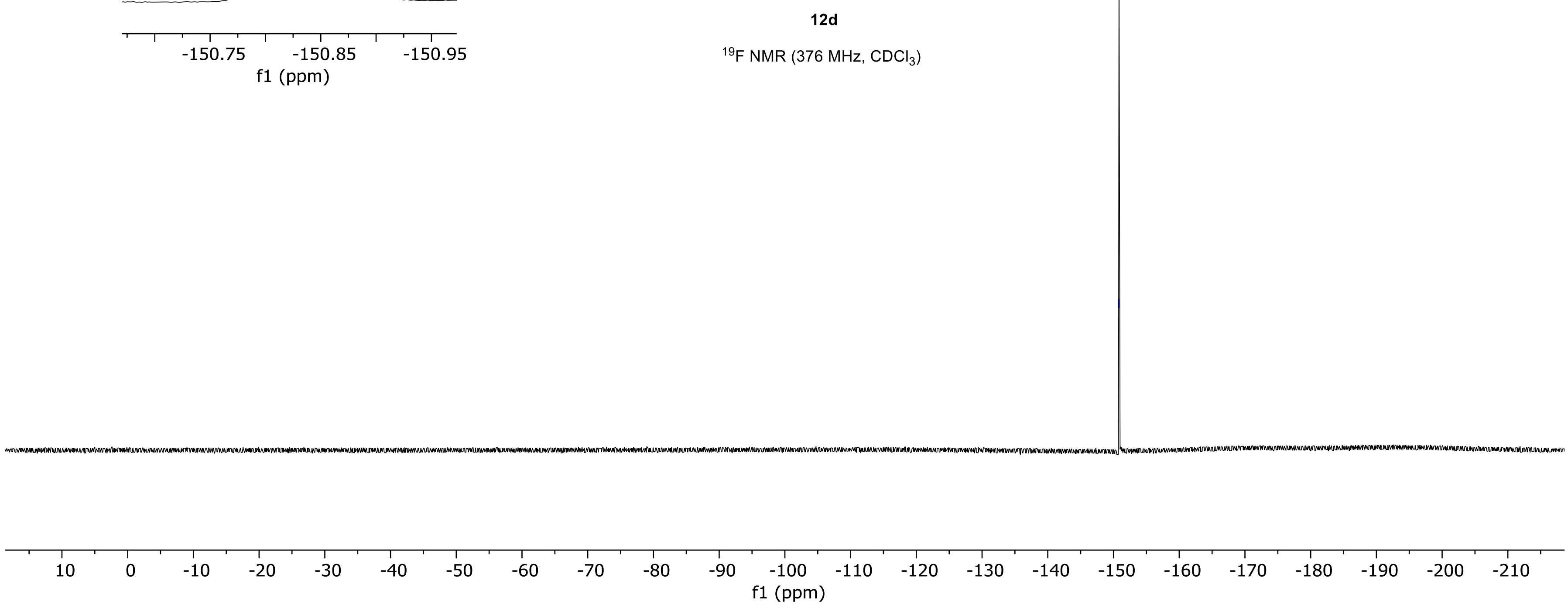

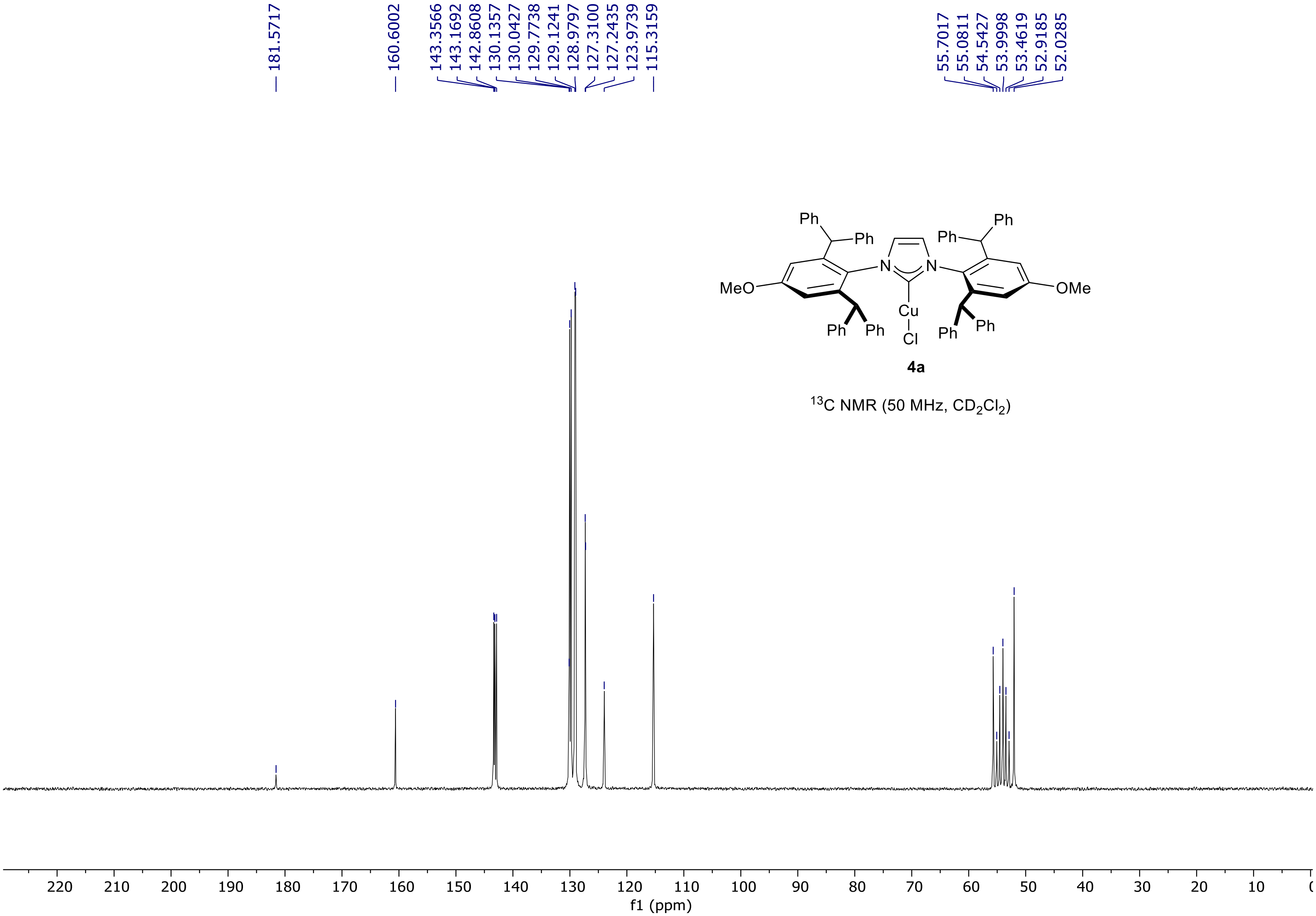
181.5717
160.6002
143.3566
143.1692
142.8608
130.1357
130.0427
129.7738
129.1241
128.9797
127.3100
127.2435
123.9739
115.3159
55.7017
55.0811
54.5427
53.9998
53.4619
52.9185
52.0285
Ph
Ph
MeO
N
N
Cu
Cl
OMe
4a
13C NMR (50 MHz, CD2Cl2)
220 210 200 190 180 170 160 150 140 130 120 110 100 90 80 70 60 50 40 30 20 10
f1 (ppm)

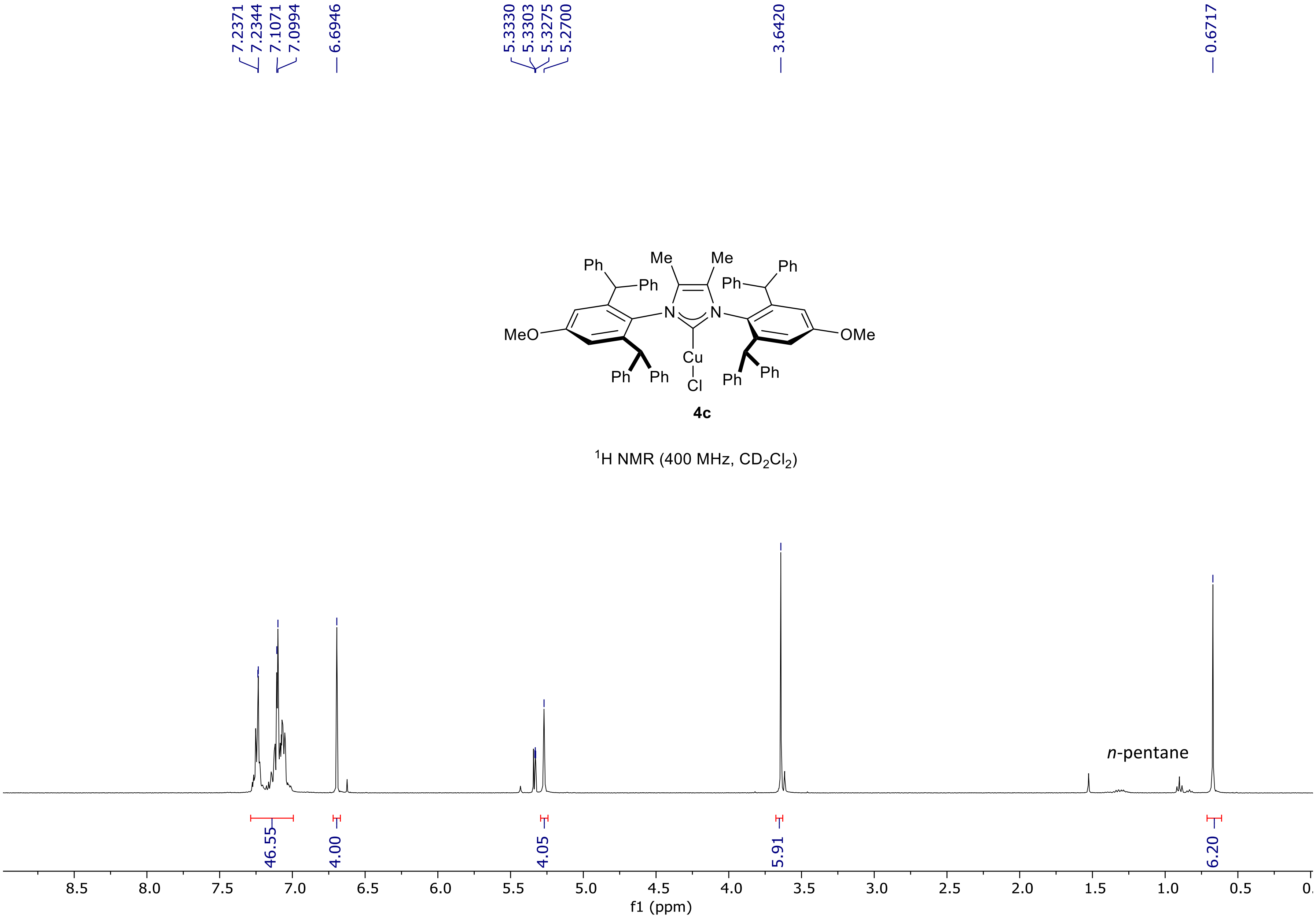

7.2371
7.2344
7.1071
7.0994
6.6946
5.3330
5.3303
5.3275
5.2700
3.6420
0.6717
Me
Me
Ph
Ph
Ph
Ph
MeO
OMe
N
N
Cu
Cl
Ph
Ph
Ph
Ph
4c
1H NMR (400 MHz, CD2Cl2)
n-pentane
46.55
4.00
4.05
5.91
6.20
8.5
8.0
7.5
7.0
6.5
6.0
5.5
5.0
4.5
4.0
3.5
3.0
2.5
2.0
1.5
1.0
0.5
f1 (ppm)

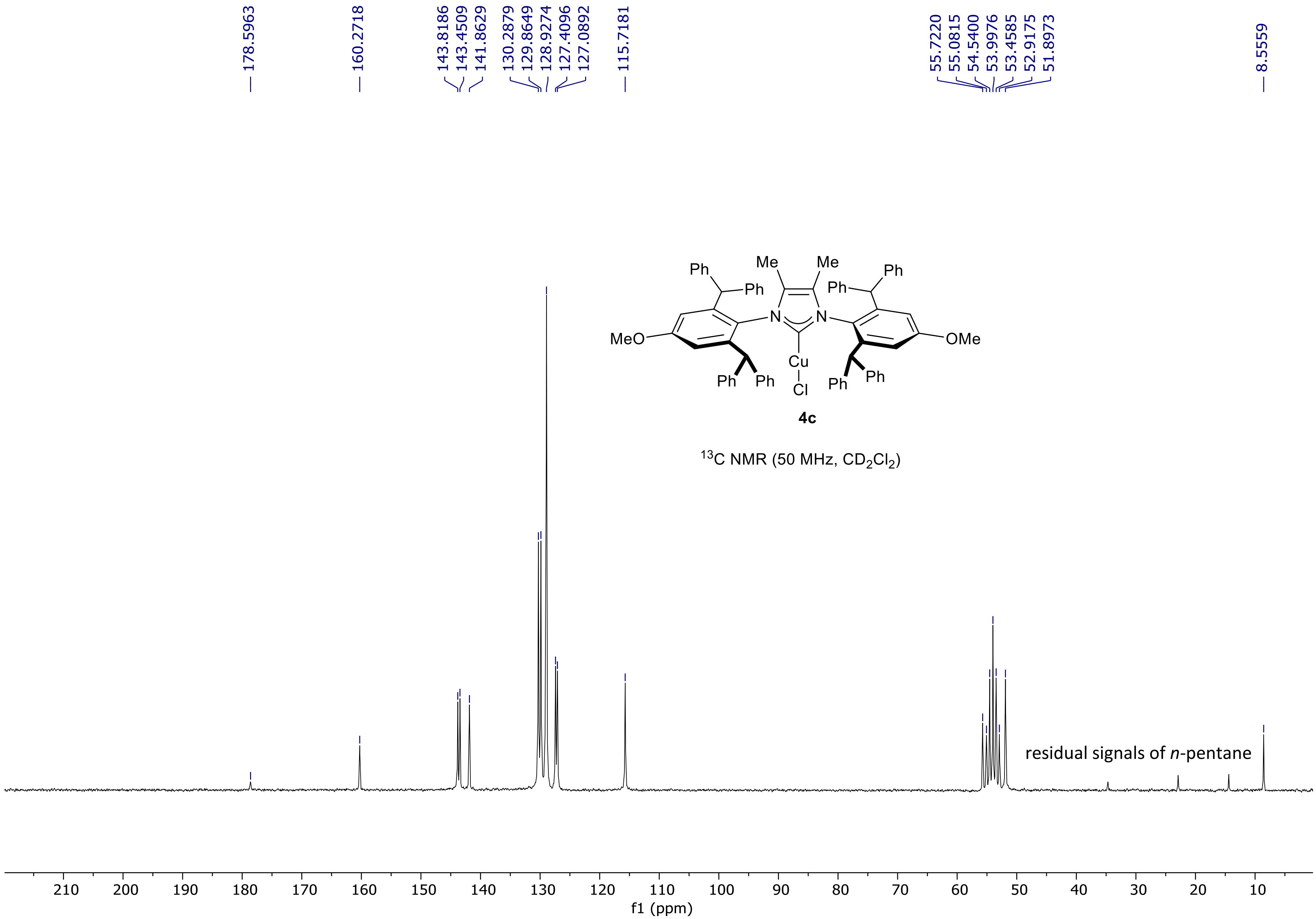

178.5963
160.2718
143.8186
143.4509
141.8629
130.2879
129.8649
128.9274
127.4096
127.0892
115.7181
55.7220
55.0815
54.5400
53.9976
53.4585
52.9175
51.8973
8.5559
Me
Me
Ph
Ph
Ph
Ph
MeO
N
N
OMe
Cu
Cl
Ph
Ph
Ph
Ph
4c
^{13}C NMR (50 MHz, CD_2Cl_2)
residual signals of *n*-pentane
210
200
190
180
170
160
150
140
130
120
110
100
90
80
70
60
50
40
30
20
10
f1 (ppm)

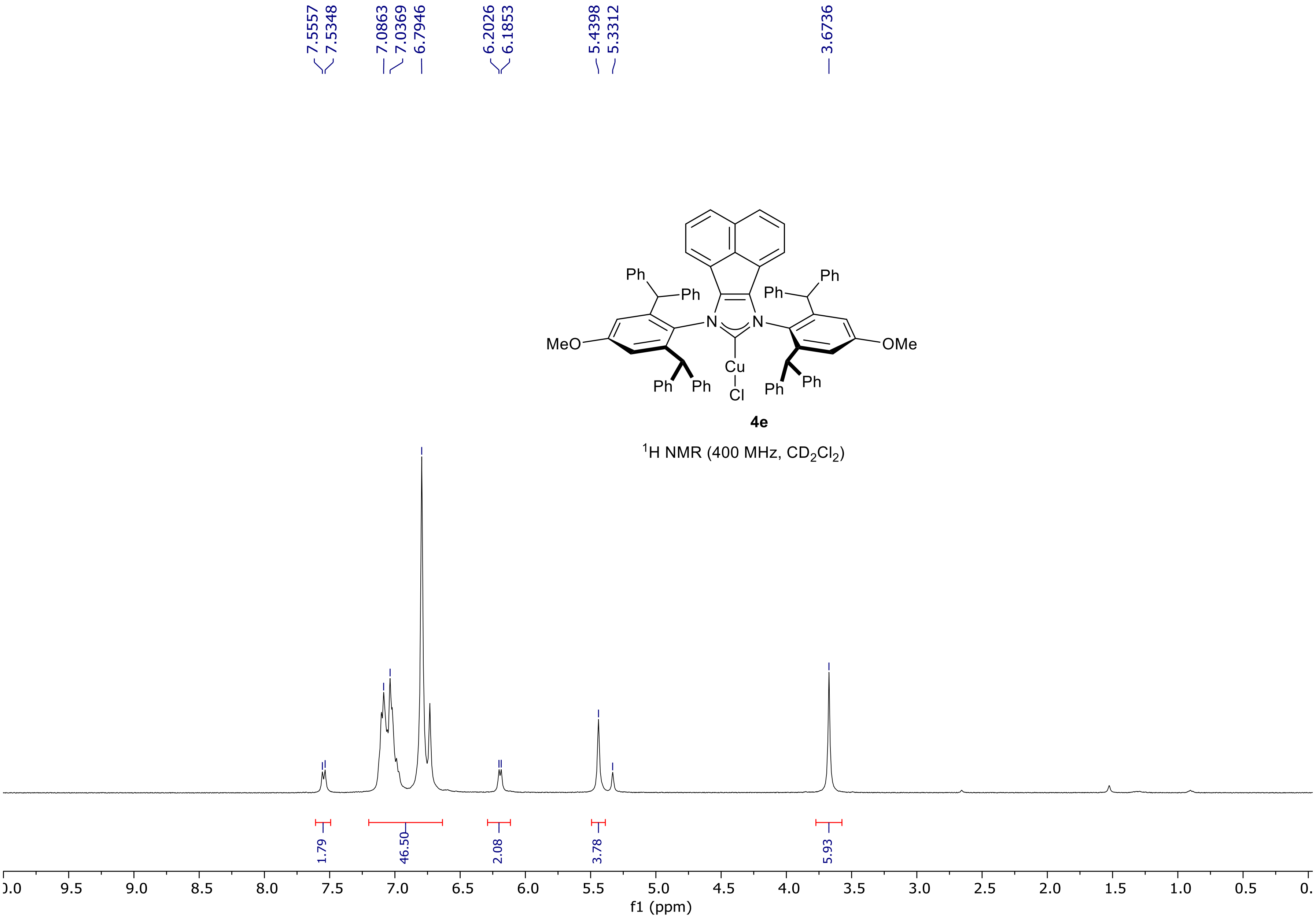

4e

^{1}H NMR (400 MHz, CD_2Cl_2)

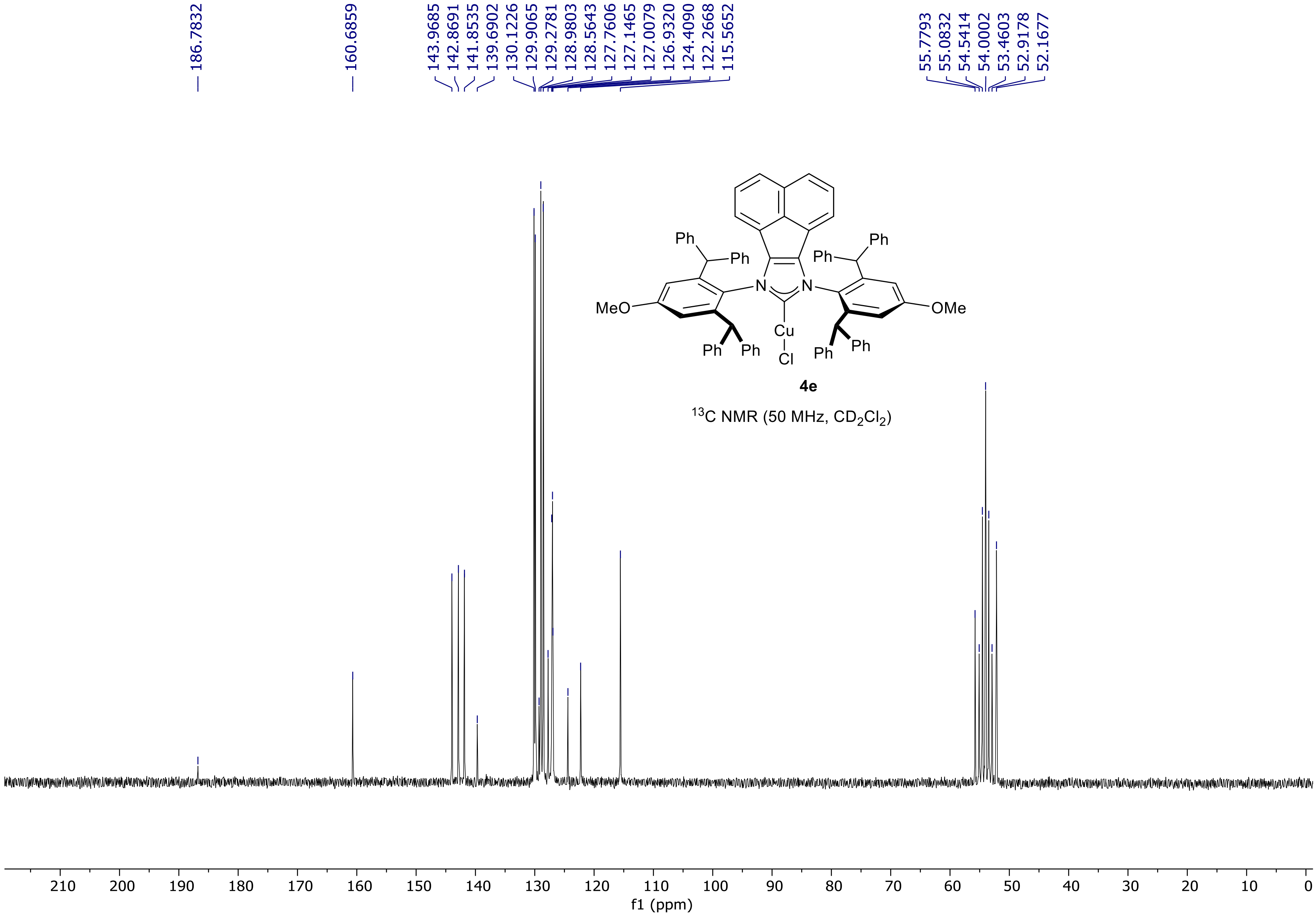

4e

^{13}C NMR (50 MHz, CD_2Cl_2)

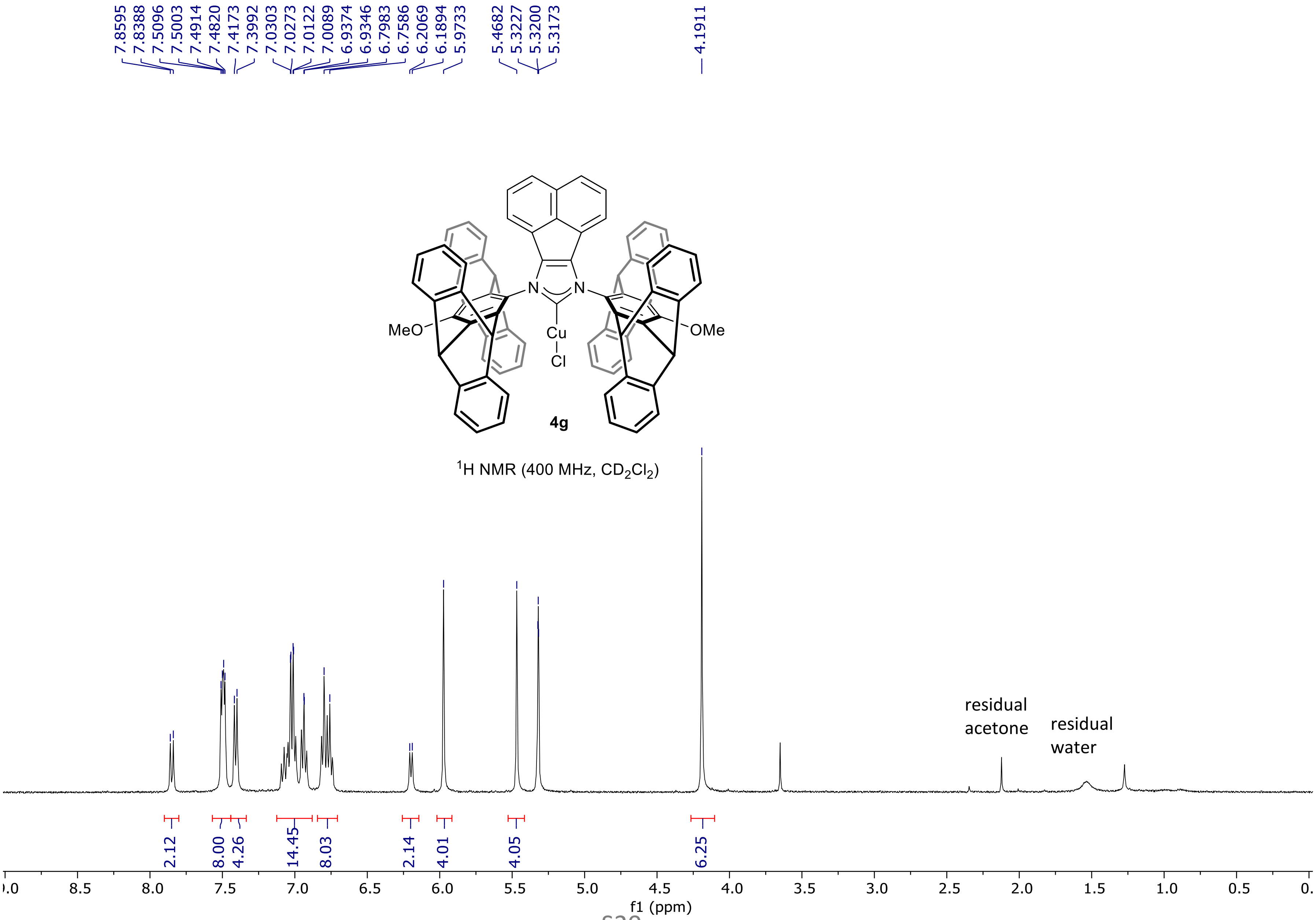
7.8595
7.8388
7.5096
7.5003
7.4914
7.4820
7.4173
7.3992
7.0303
7.0273
7.0122
7.0089
6.9374
6.9346
6.7983
6.7586
6.2069
6.1894
5.9733
5.4682
5.3227
5.3200
5.3173
4.1911
MeO
OMe
N
N
Cu
Cl
4g
1H NMR (400 MHz, CD2Cl2)
residual acetone
residual water
2.12
8.00
4.26
14.45
8.03
2.14
4.01
4.05
6.25
8.5
8.0
7.5
7.0
6.5
6.0
5.5
5.0
4.5
4.0
3.5
3.0
2.5
2.0
1.5
1.0
0.5
f1 (ppm)

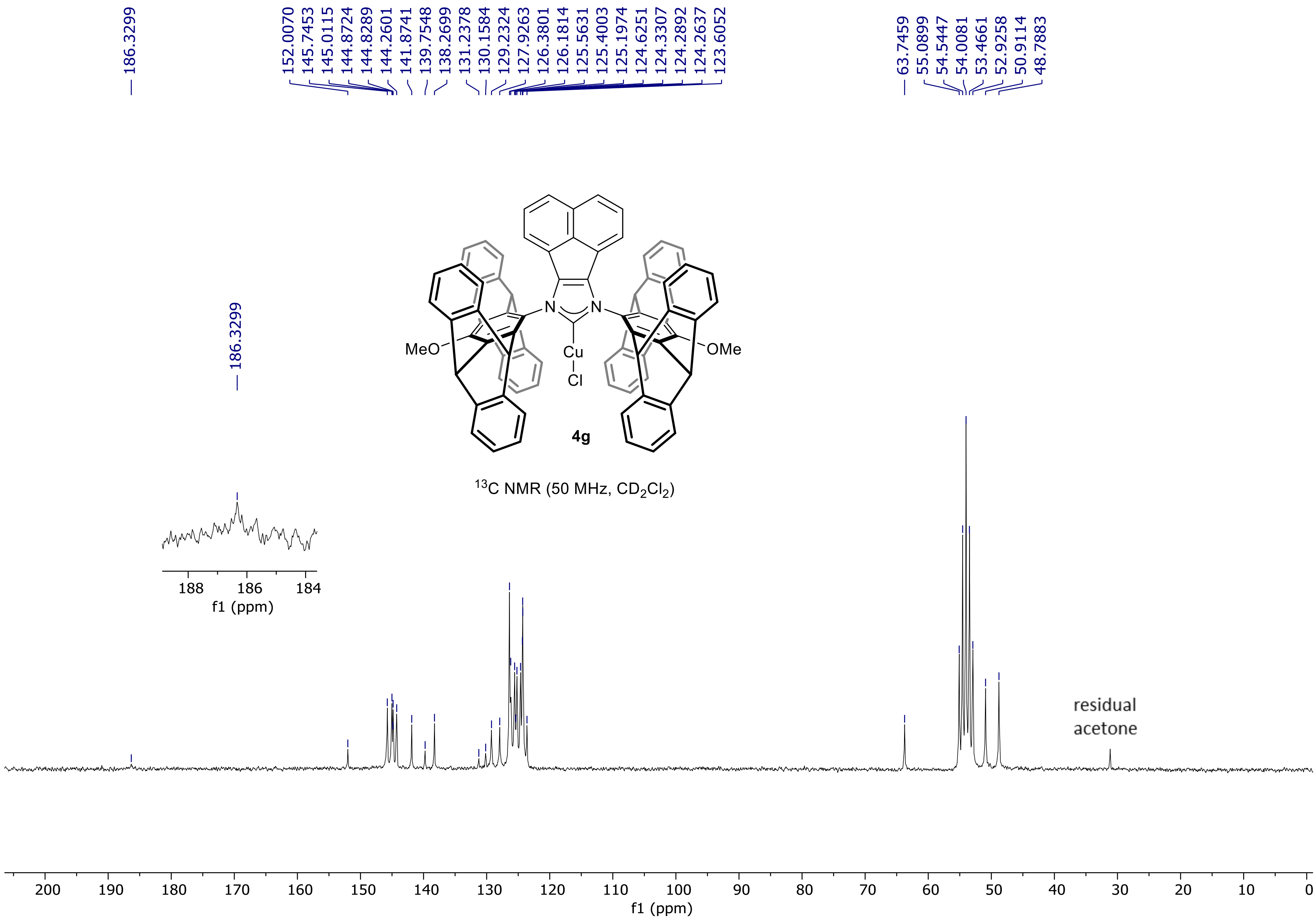
186.3299
152.0070
145.7453
145.0115
144.8724
144.8289
144.2601
141.8741
139.7548
138.2699
131.2378
130.1584
129.2324
127.9263
126.3801
126.1814
125.5631
125.4003
125.1974
124.6251
124.3307
124.2892
124.2637
123.6052
63.7459
55.0899
54.5447
54.0081
53.4661
52.9258
50.9114
48.7883
MeO
OMe
N
N
Cu
Cl
4g
^{13}C NMR (50 MHz, CD_2Cl_2)
188
186
184
f1 (ppm)
residual acetone
200
190
180
170
160
150
140
130
120
110
100
90
80
70
60
50
40
30
20
10
0
f1 (ppm)

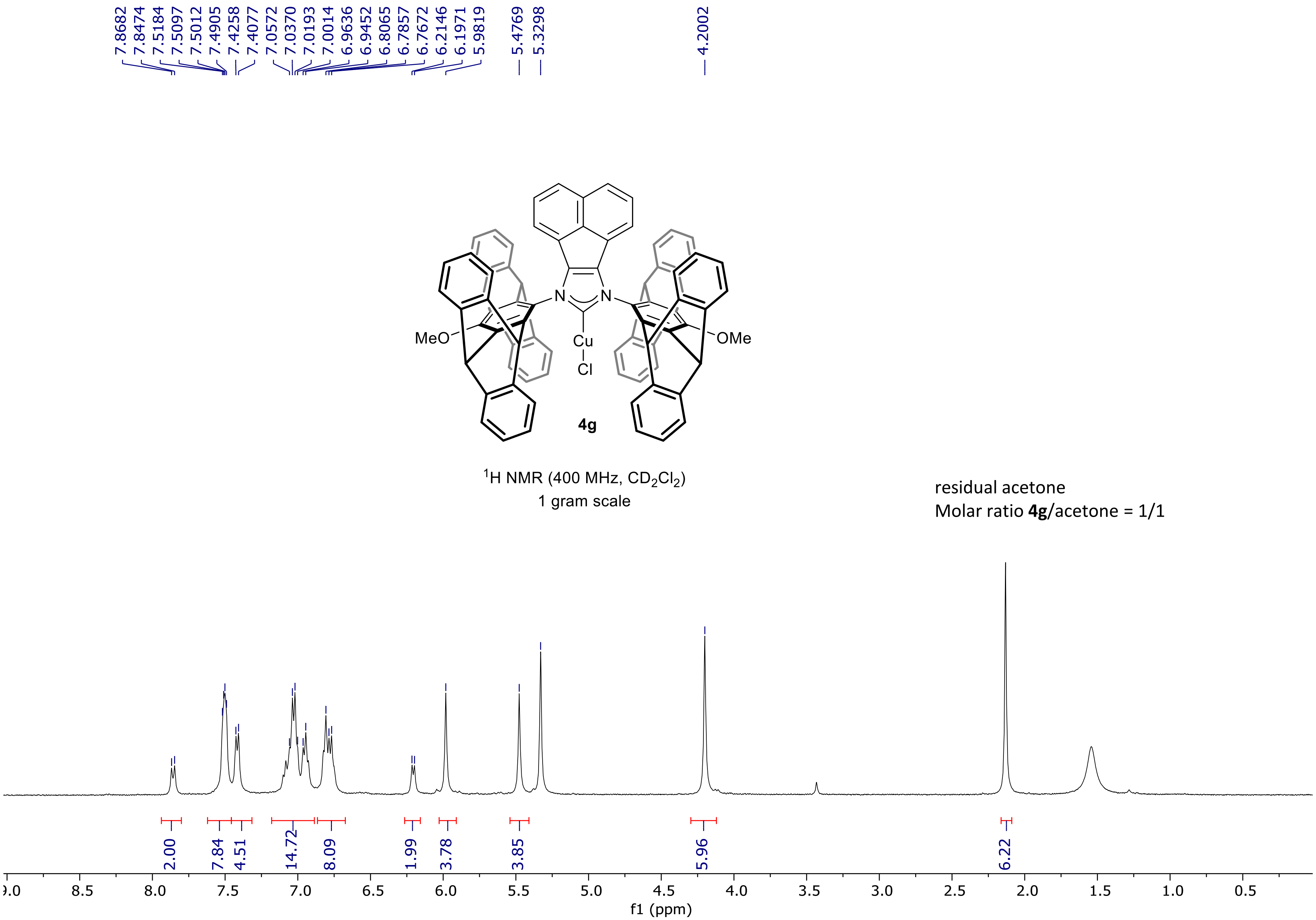
7.8682
7.8474
7.5184
7.5097
7.5012
7.4905
7.4258
7.4077
7.0572
7.0370
7.0193
7.0014
6.9636
6.9452
6.8065
6.7857
6.7672
6.2146
6.1971
5.9819
5.4769
5.3298
4.2002
MeO
OMe
N
N
Cu
Cl
4g
^{1}H NMR (400 MHz, CD_2Cl_2)
1 gram scale
residual acetone
Molar ratio **4g**/acetone = 1/1
2.00
7.84
4.51
14.72
8.09
1.99
3.78
3.85
5.96
6.22
f1 (ppm)

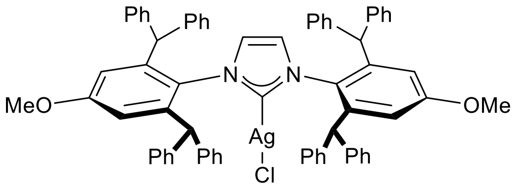

4b

^{1}H NMR (400 MHz, CD_2Cl_2)

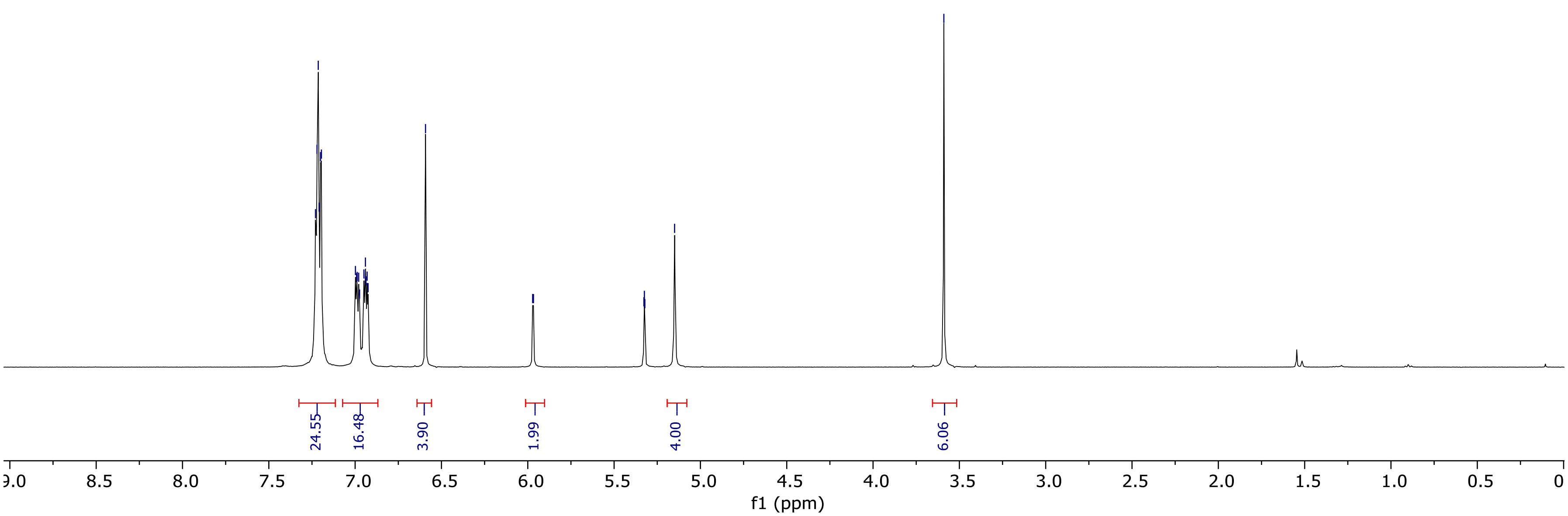

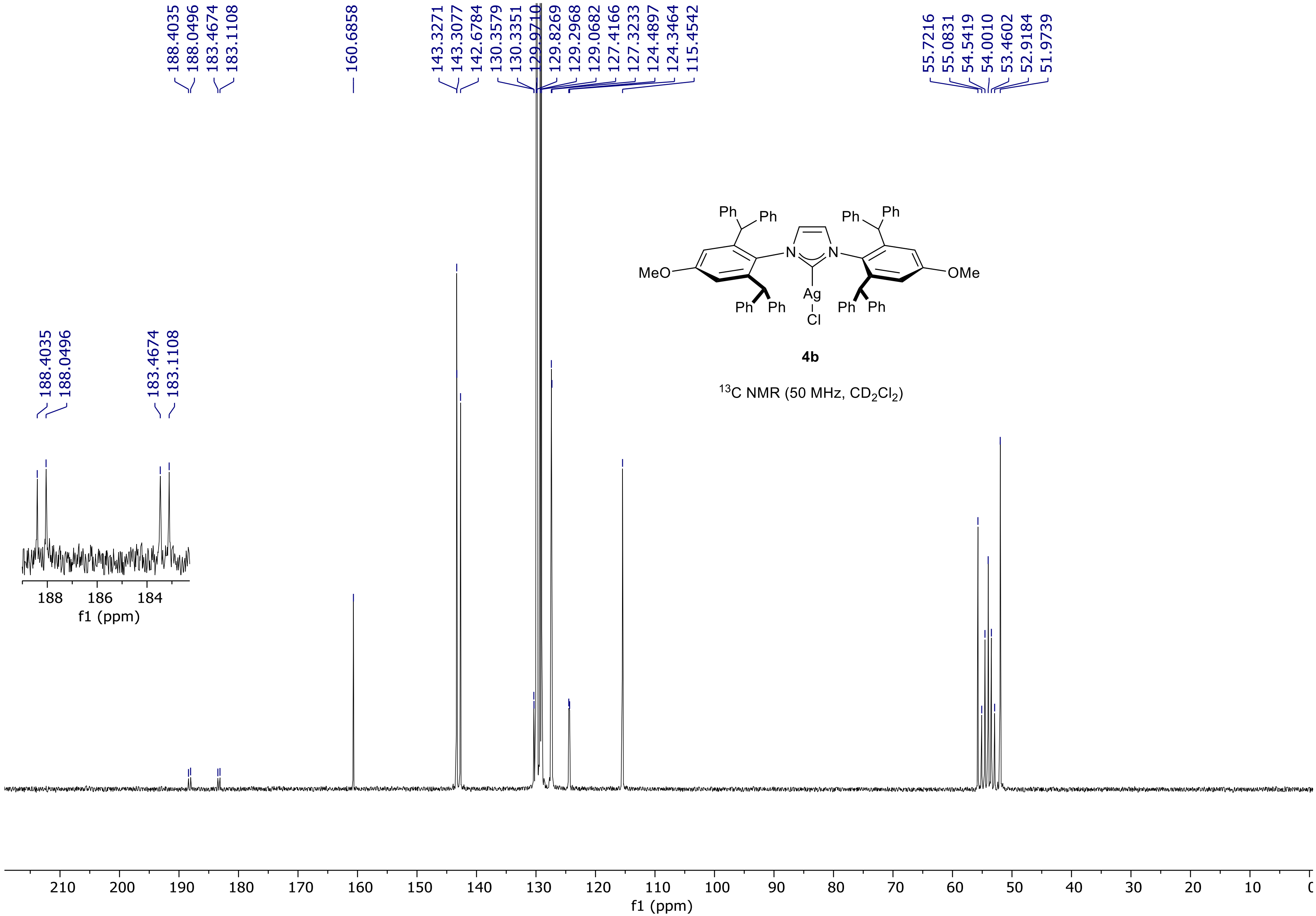
188.4035
188.0496
183.4674
183.1108
160.6858
143.3271
143.3077
142.6784
130.3579
130.3351
129.9710
129.8269
129.2968
129.0682
127.4166
127.3233
124.4897
124.3464
115.4542
55.7216
55.0831
54.5419
54.0010
53.4602
52.9184
51.9739
Ph
Ph
MeO
N
N
Ag
Cl
OMe
4b
^{13}C NMR (50 MHz, CD_2Cl_2)
188 186 184
f1 (ppm)
210 200 190 180 170 160 150 140 130 120 110 100 90 80 70 60 50 40 30 20 10
f1 (ppm)

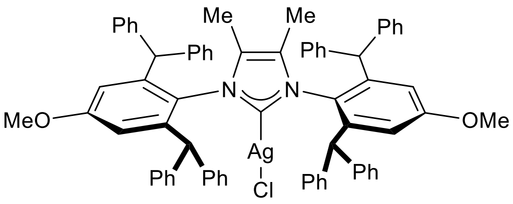

4d

^{1}H NMR (400 MHz, CD_2Cl_2)

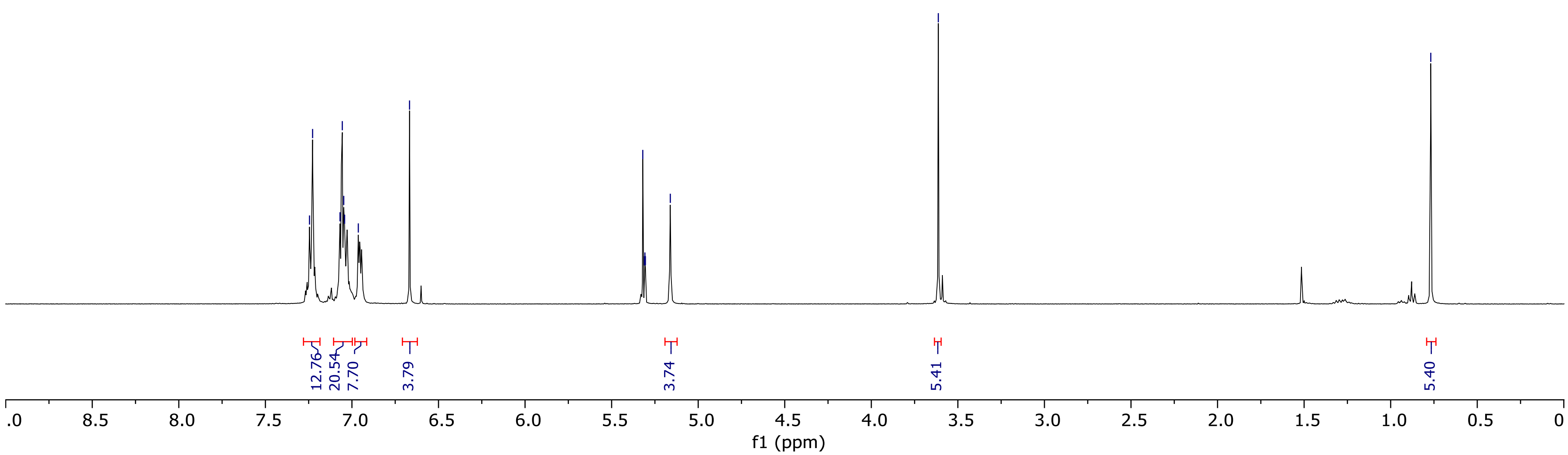

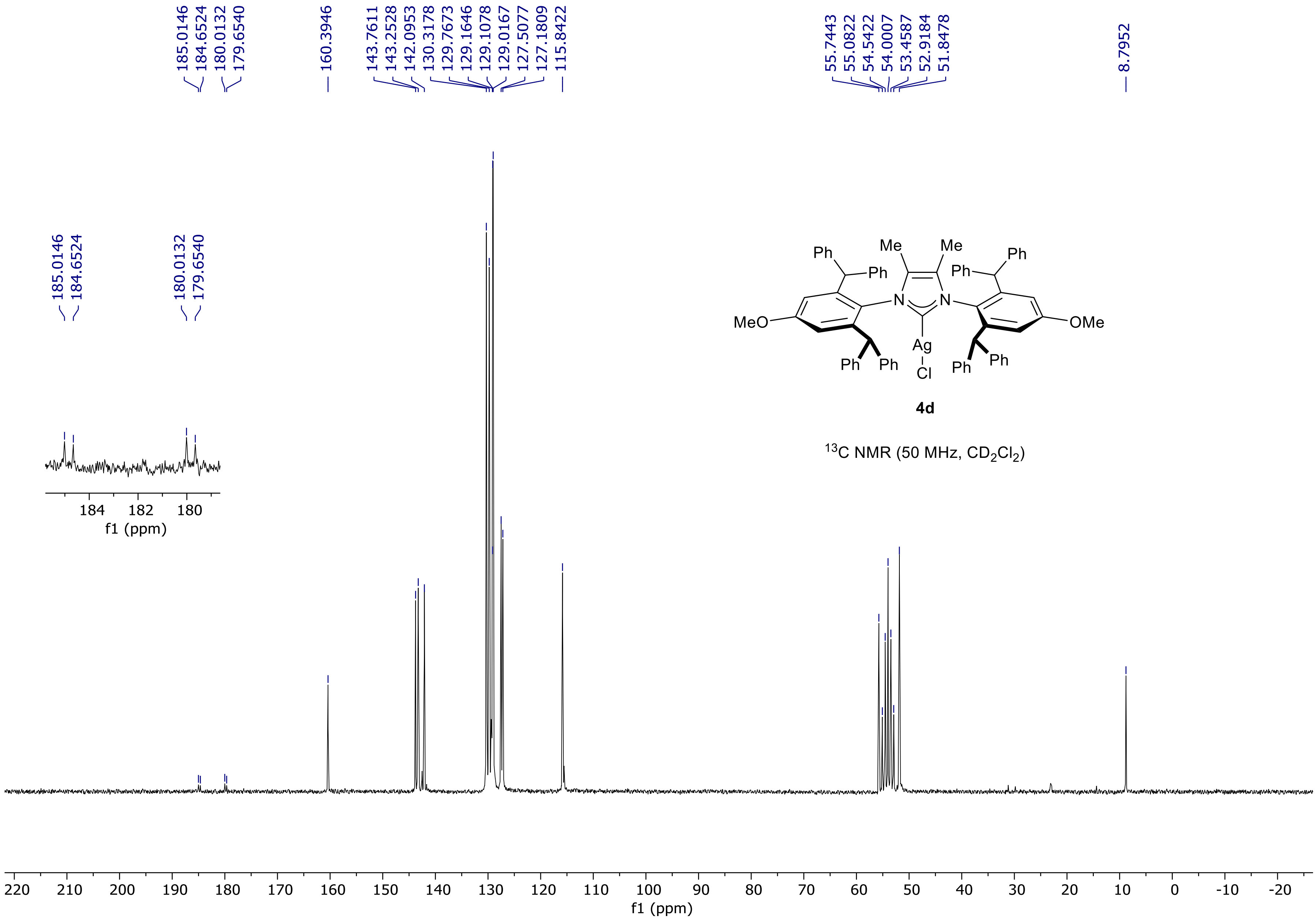
185.0146
184.6524
180.0132
179.6540
160.3946
143.7611
143.2528
142.0953
130.3178
129.7673
129.1646
129.1078
129.0167
127.5077
127.1809
115.8422
55.7443
55.0822
54.5422
54.0007
53.4587
52.9184
51.8478
8.7952
185.0146
184.6524
180.0132
179.6540
184
182
180
f1 (ppm)
Me
Me
Ph
Ph
Ph
Ph
MeO
N
N
OMe
Ag
Cl
Ph
Ph
Ph
Ph
4d
^{13}C NMR (50 MHz, CD_2Cl_2)
220 210 200 190 180 170 160 150 140 130 120 110 100 90 80 70 60 50 40 30 20 10 0 -10 -20
f1 (ppm)

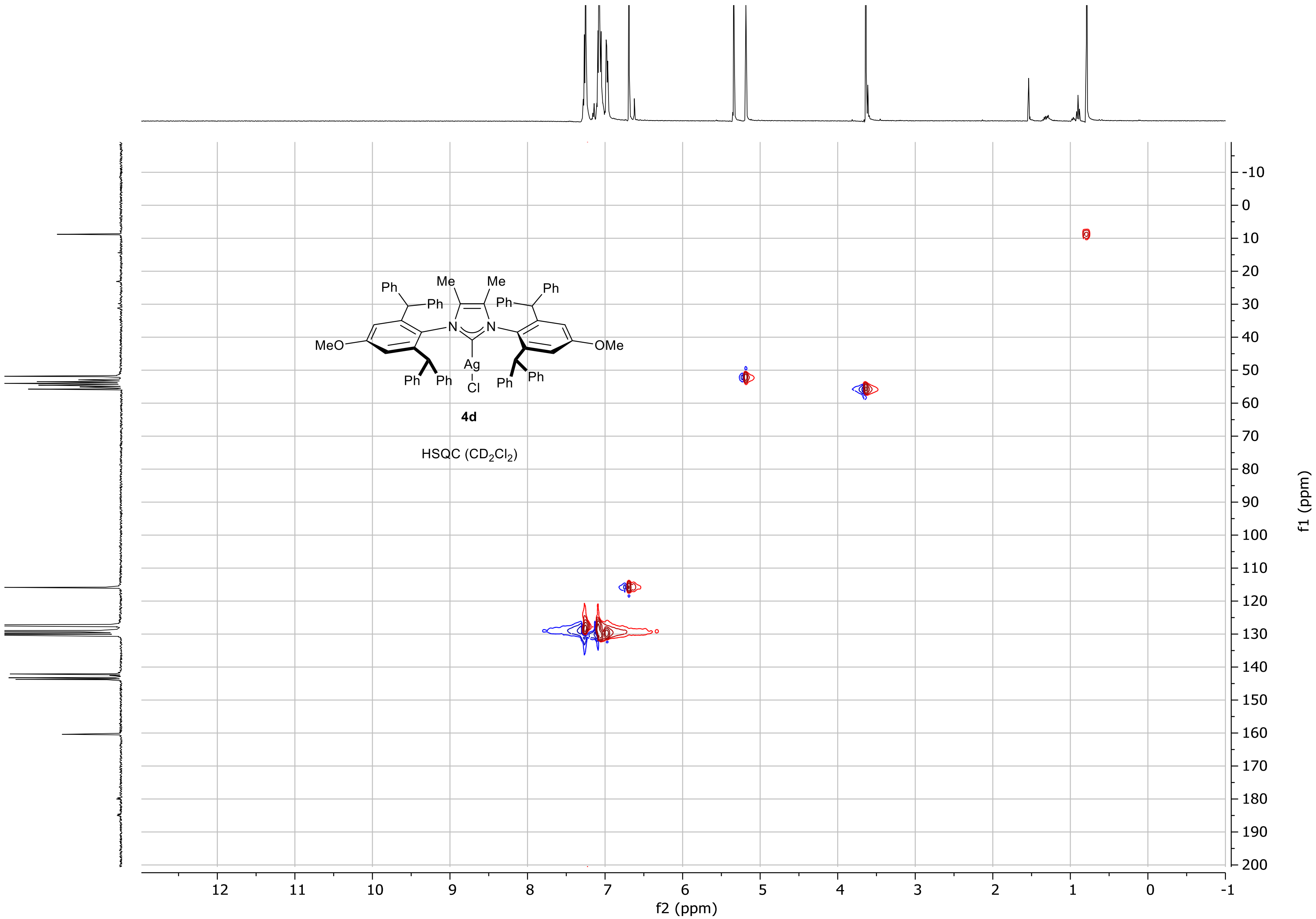
Ph
Ph
Me
Me
Ph
Ph
MeO
N
N
OMe
Ag
Cl
Ph
Ph
Ph
Ph
4d
HSQC (CD_2Cl_2)
f2 (ppm)
f1 (ppm)

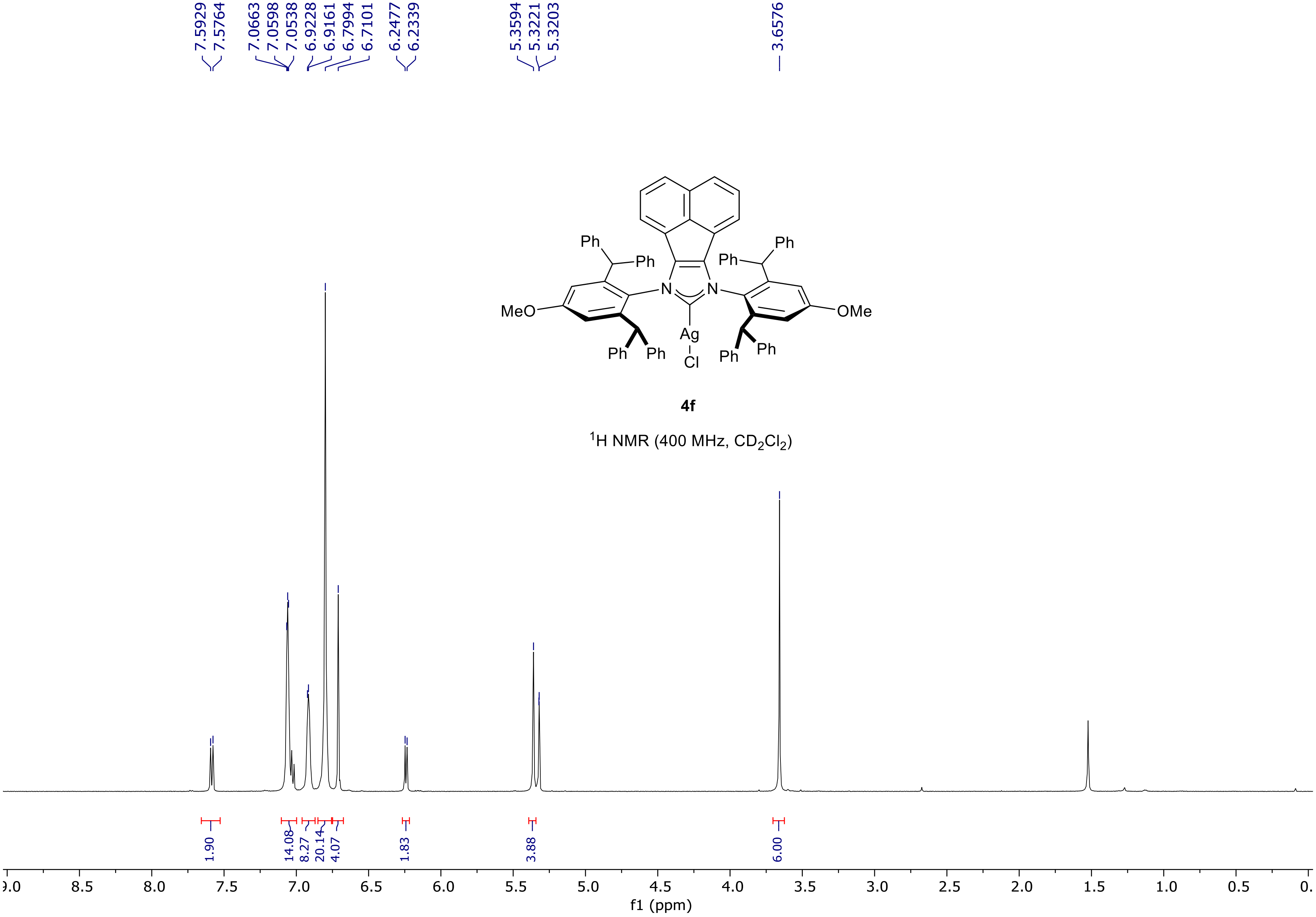
7.5929
7.5764
7.0663
7.0598
7.0538
6.9228
6.9161
6.7994
6.7101
6.2477
6.2339
5.3594
5.3221
5.3203
3.6576
Ph
Ph
Ph
Ph
MeO
OMe
N
N
Ag
Cl
Ph
Ph
Ph
Ph
4f
¹H NMR (400 MHz, CD₂Cl₂)
1.90
14.08
8.27
20.14
4.07
1.83
3.88
6.00
f1 (ppm)

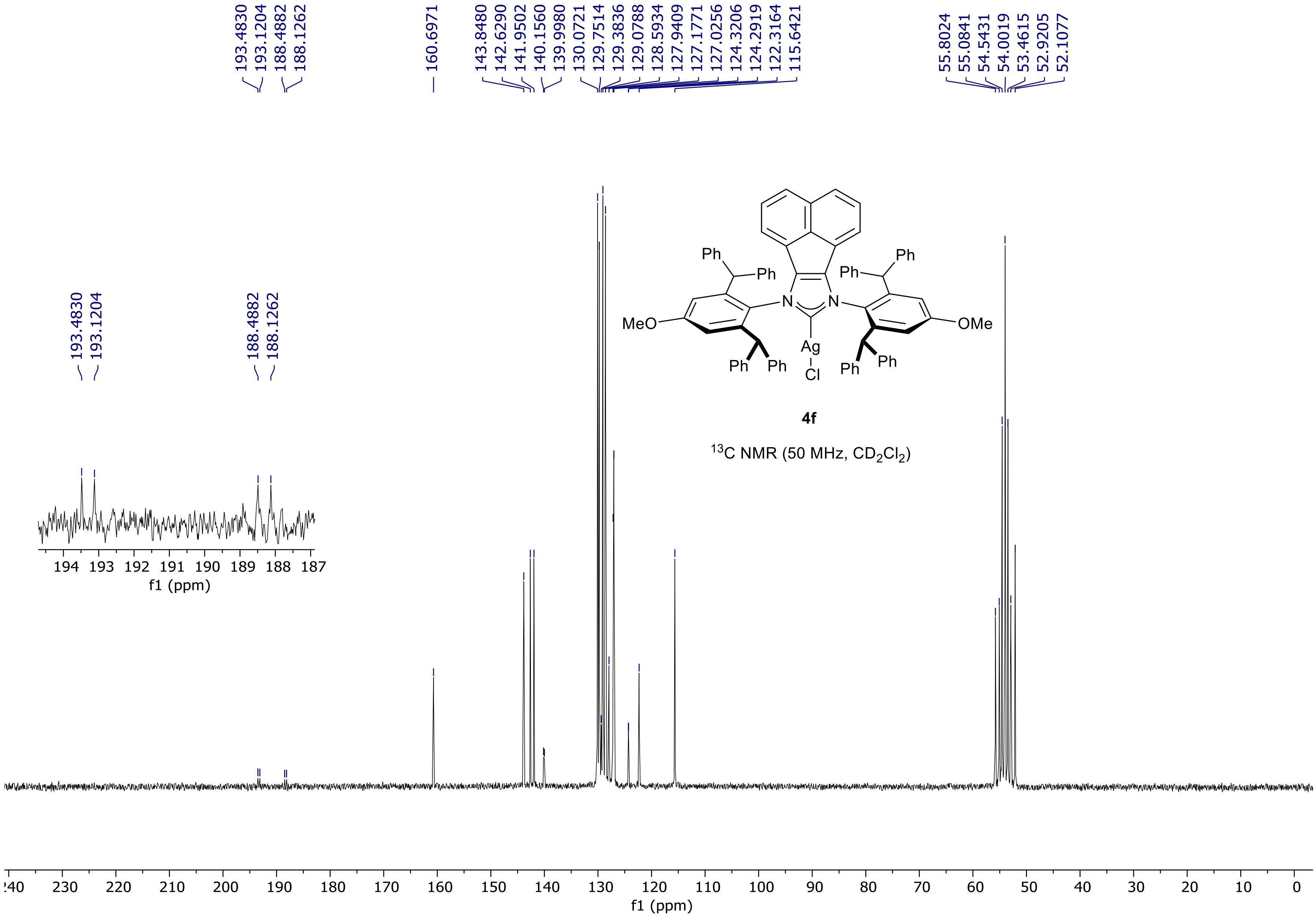
193.4830
193.1204
188.4882
188.1262
160.6971
143.8480
142.6290
141.9502
140.1560
139.9980
130.0721
129.7514
129.3836
129.0788
128.5934
127.9409
127.1771
127.0256
124.3206
124.2919
122.3164
115.6421
55.8024
55.0841
54.5431
54.0019
53.4615
52.9205
52.1077
194 193 192 191 190 189 188 187
f1 (ppm)
Ph
Ph
Ph
Ph
MeO
OMe
N
N
Ag
Cl
Ph
Ph
Ph
Ph
4f
13C NMR (50 MHz, CD2Cl2)
240 230 220 210 200 190 180 170 160 150 140 130 120 110 100 90 80 70 60 50 40 30 20 10 0
f1 (ppm)

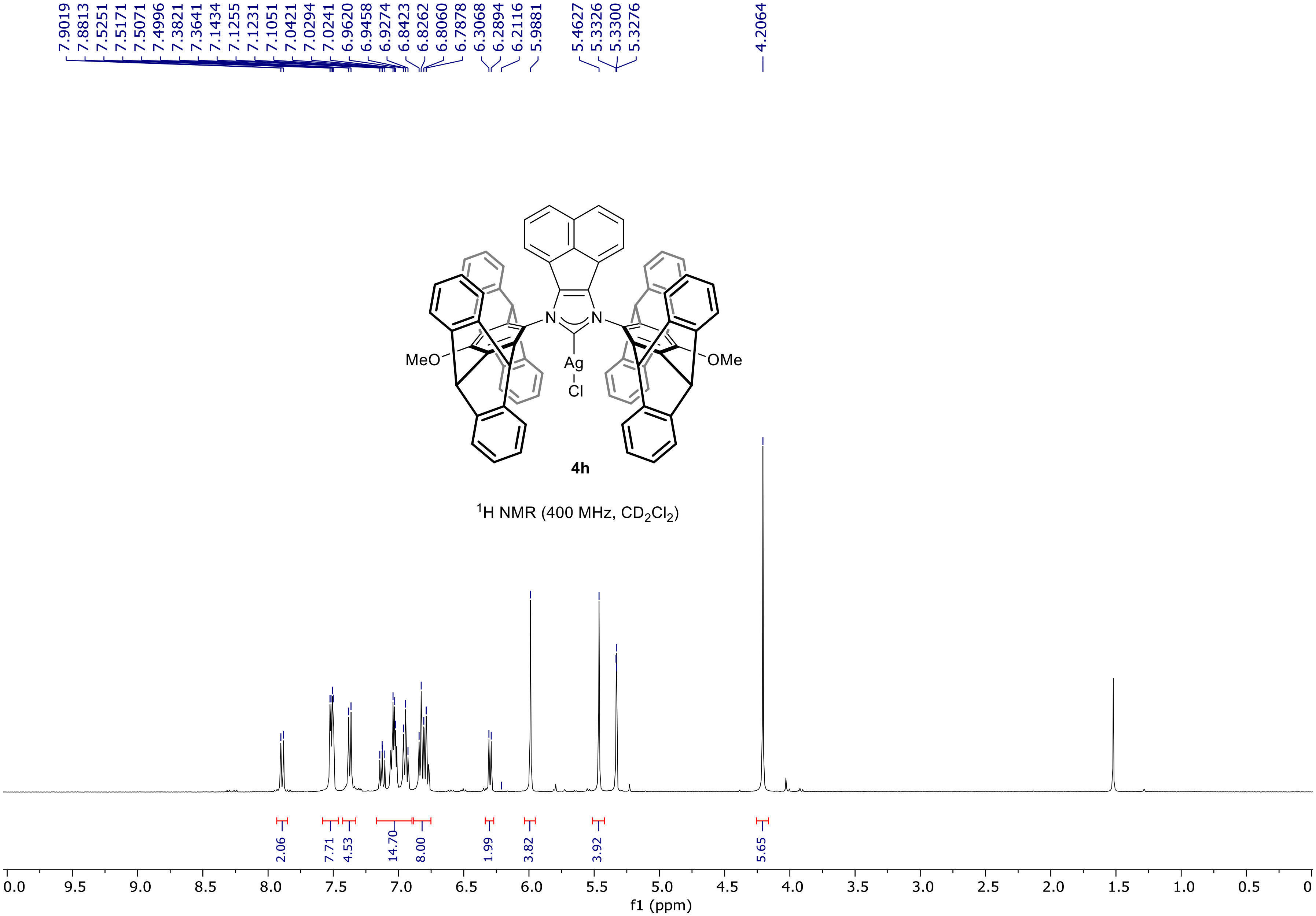

7.9019
7.8813
7.5251
7.5171
7.5071
7.4996
7.3821
7.3641
7.1434
7.1255
7.1231
7.1051
7.0421
7.0294
7.0241
6.9620
6.9458
6.9274
6.8423
6.8262
6.8060
6.7878
6.3068
6.2894
6.2116
5.9881
5.4627
5.3326
5.3300
5.3276
4.2064
MeO
OMe
N
N
Ag
Cl
4h
^{1}H NMR (400 MHz, CD_2Cl_2)
2.06
7.71
4.53
14.70
8.00
1.99
3.82
3.92
5.65
0.0 9.5 9.0 8.5 8.0 7.5 7.0 6.5 6.0 5.5 5.0 4.5 4.0 3.5 3.0 2.5 2.0 1.5 1.0 0.5 0
f1 (ppm)

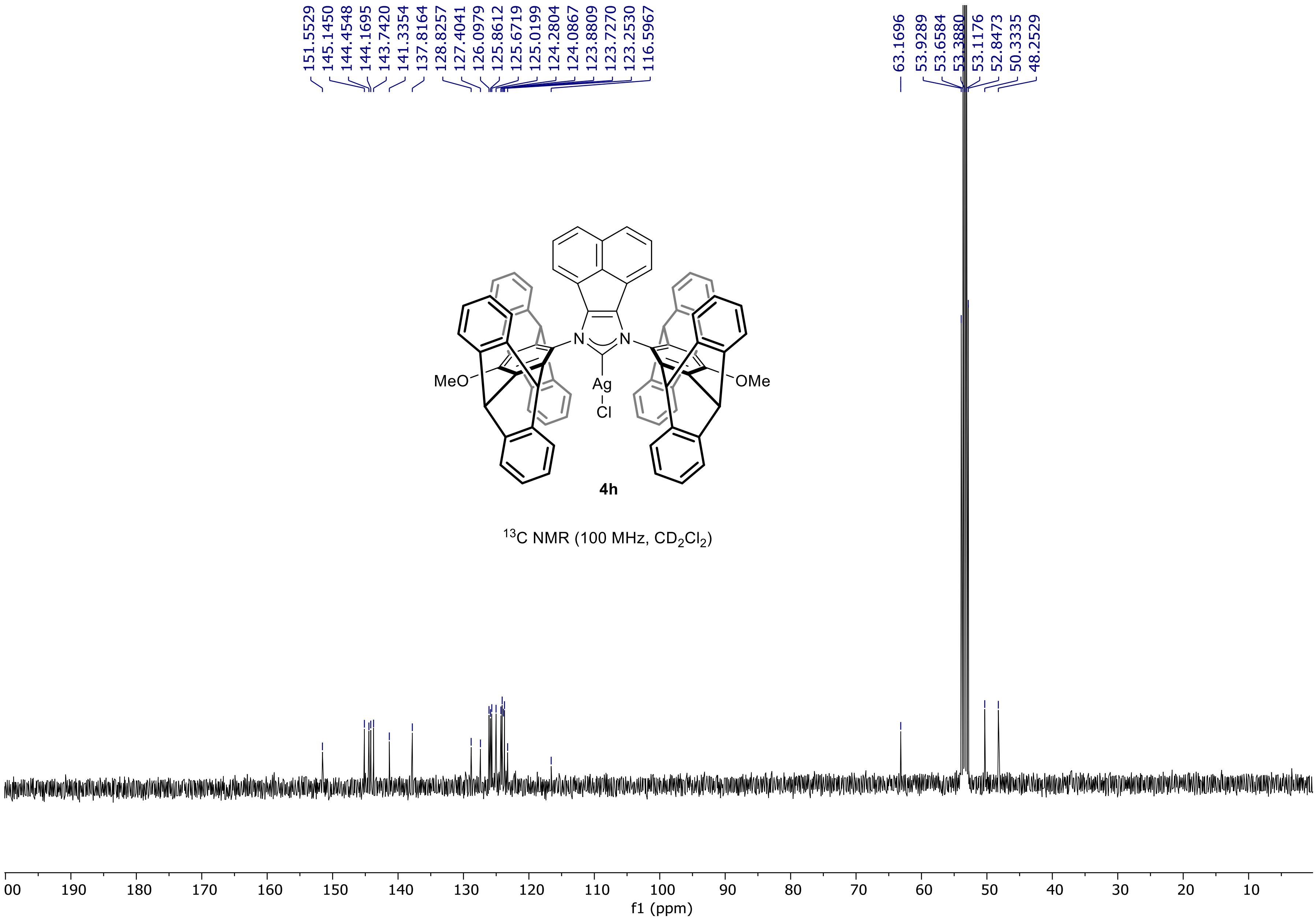

4h

^{13}C NMR (100 MHz, CD_2Cl_2)

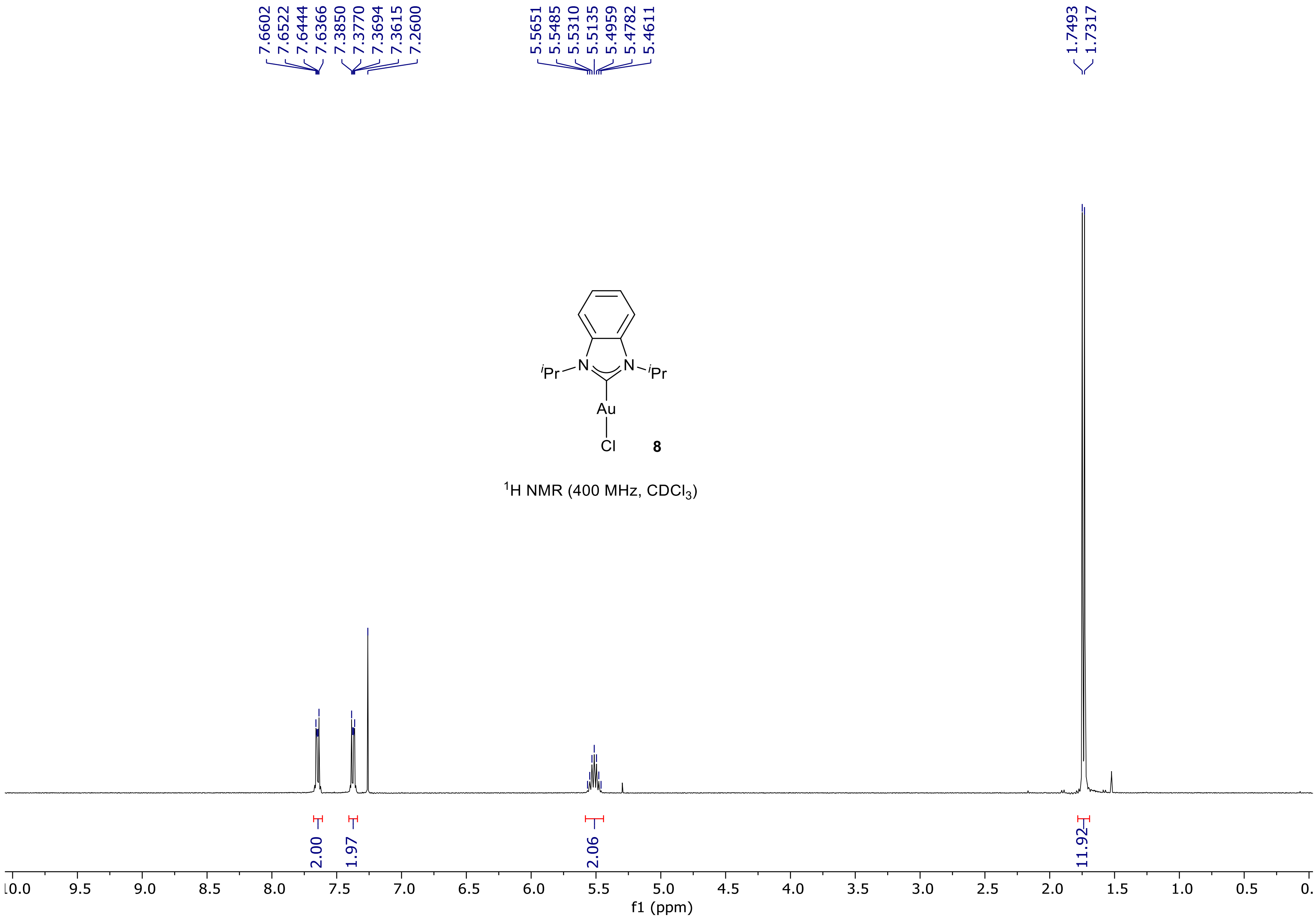
7.6602
7.6522
7.6444
7.6366
7.3850
7.3770
7.3694
7.3615
7.2600
5.5651
5.5485
5.5310
5.5135
5.4959
5.4782
5.4611
1.7493
1.7317
^{i}Pr
N
N
^{i}Pr
Au
Cl
8
^{1}H NMR (400 MHz, $CDCl_3$)
2.00
1.97
2.06
11.92
10.0
9.5
9.0
8.5
8.0
7.5
7.0
6.5
6.0
5.5
5.0
4.5
4.0
3.5
3.0
2.5
2.0
1.5
1.0
0.5
0.
f1 (ppm)

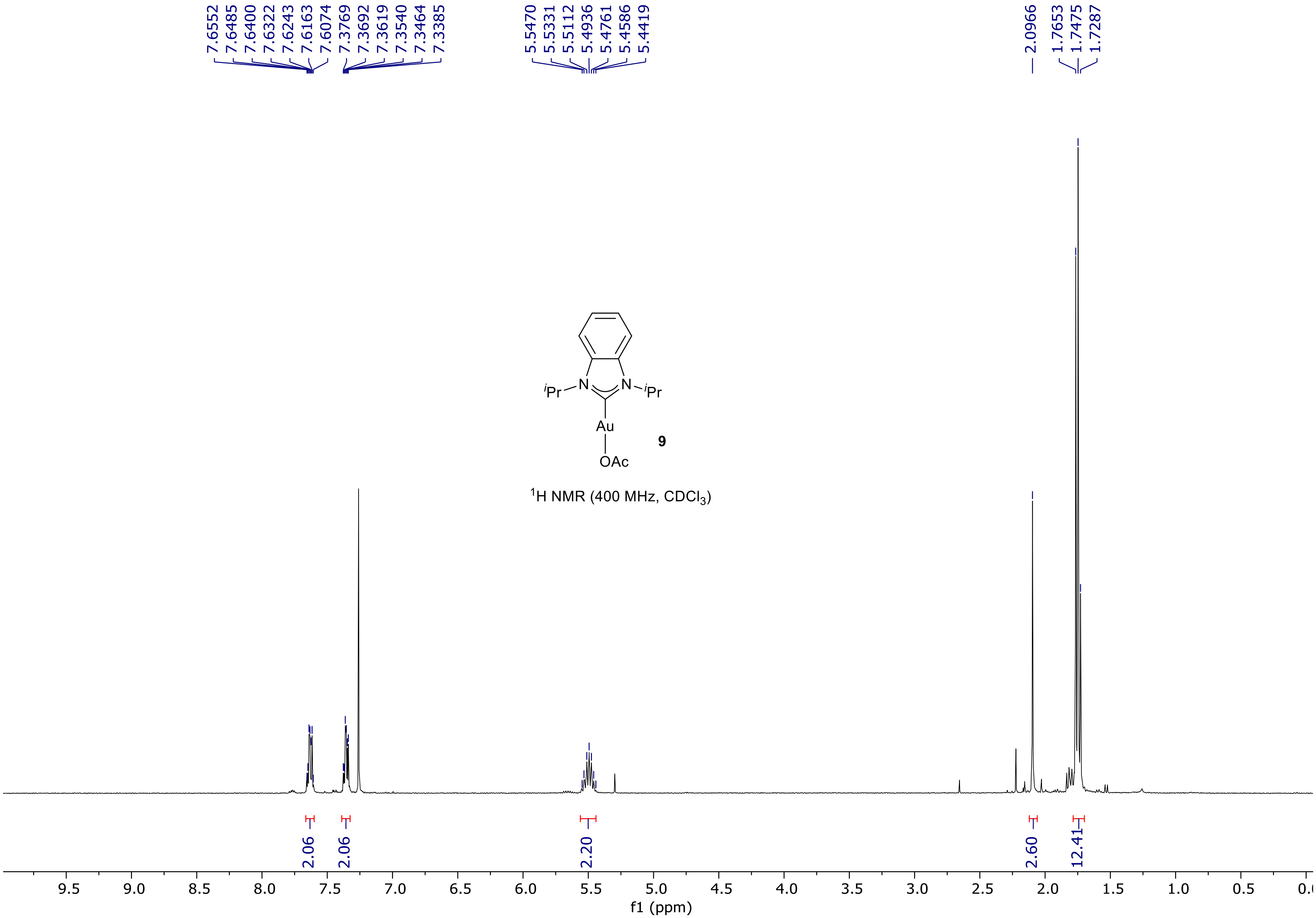
7.6552
7.6485
7.6400
7.6322
7.6243
7.6163
7.6074
7.3769
7.3692
7.3619
7.3540
7.3464
7.3385
5.5470
5.5331
5.5112
5.4936
5.4761
5.4586
5.4419
2.0966
1.7653
1.7475
1.7287
ⁱPr
N
N
ⁱPr
Au
OAc
9
1H NMR (400 MHz, CDCl3)
2.06
2.06
2.20
2.60
12.41
f1 (ppm)

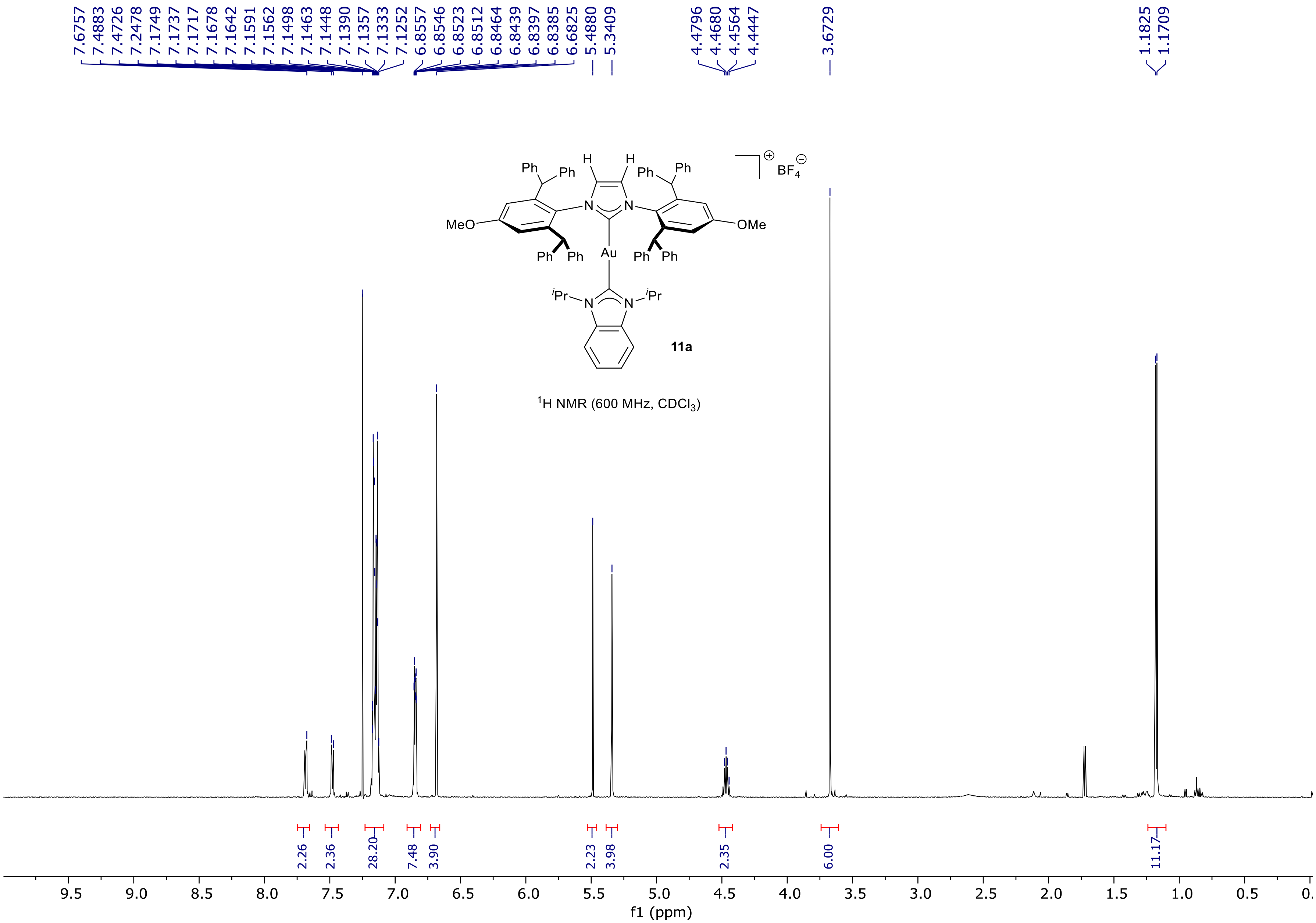
7.6757
7.4883
7.4726
7.2478
7.1749
7.1737
7.1717
7.1678
7.1642
7.1591
7.1562
7.1498
7.1463
7.1448
7.1390
7.1357
7.1333
7.1252
6.8557
6.8546
6.8523
6.8512
6.8464
6.8439
6.8397
6.8385
6.6825
5.4880
5.3409
4.4796
4.4680
4.4564
4.4447
3.6729
1.1825
1.1709
Ph
Ph
H
H
Ph
Ph
MeO
N
N
OMe
Ph
Ph
Au
Ph
Ph
BF4
iPr
N
N
iPr
11a
1H NMR (600 MHz, CDCl3)
2.26
2.36
28.20
7.48
3.90
2.23
3.98
2.35
6.00
11.17
9.5
9.0
8.5
8.0
7.5
7.0
6.5
6.0
5.5
5.0
4.5
4.0
3.5
3.0
2.5
2.0
1.5
1.0
0.5
0.
f1 (ppm)

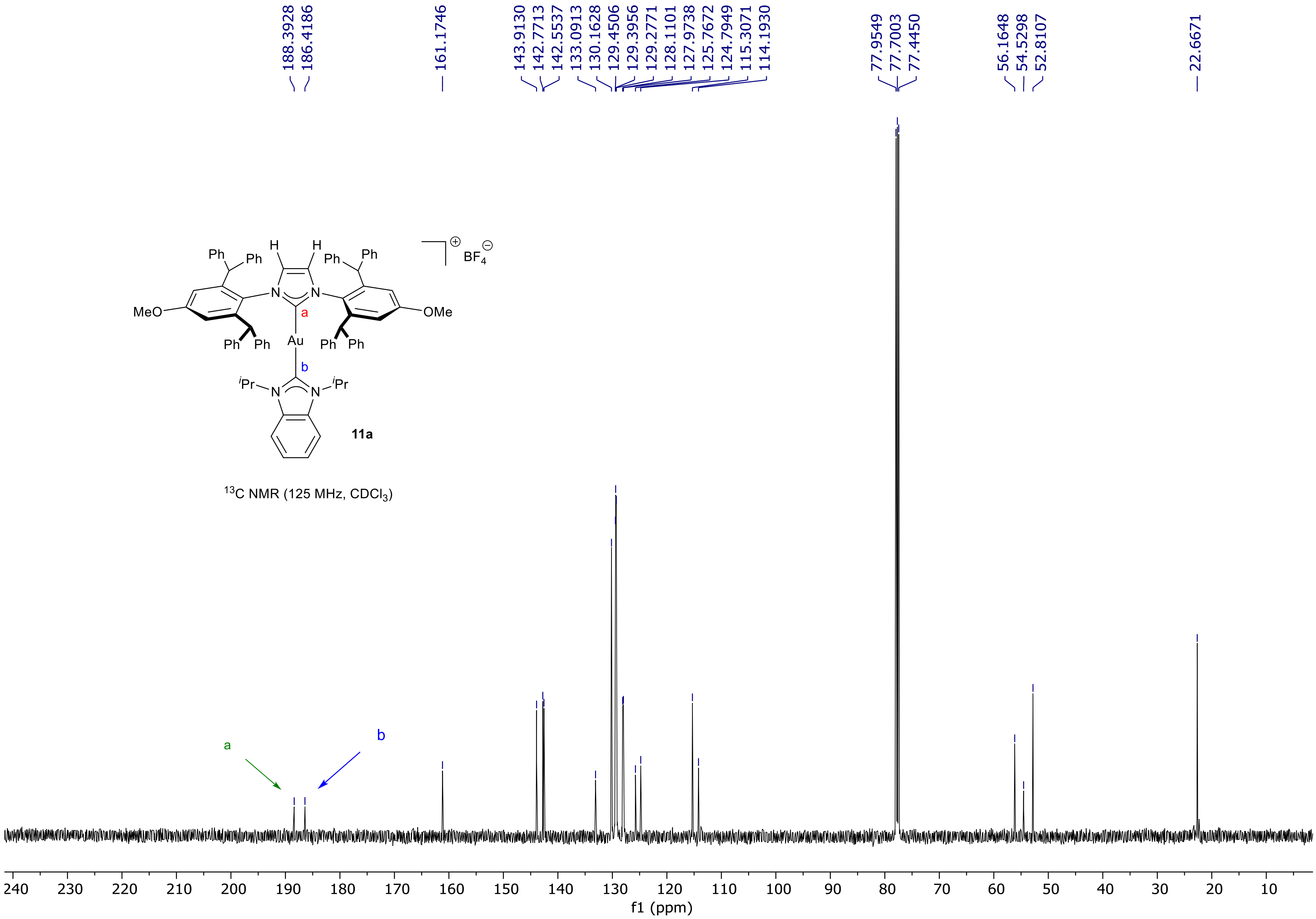

188.3928
186.4186
161.1746
143.9130
142.7713
142.5537
133.0913
130.1628
129.4506
129.3956
129.2771
128.1101
127.9738
125.7672
124.7949
115.3071
114.1930
77.9549
77.7003
77.4450
56.1648
54.5298
52.8107
22.6671
11a
^{13}C NMR (125 MHz, CDCl$_3$)
a
b
f1 (ppm)

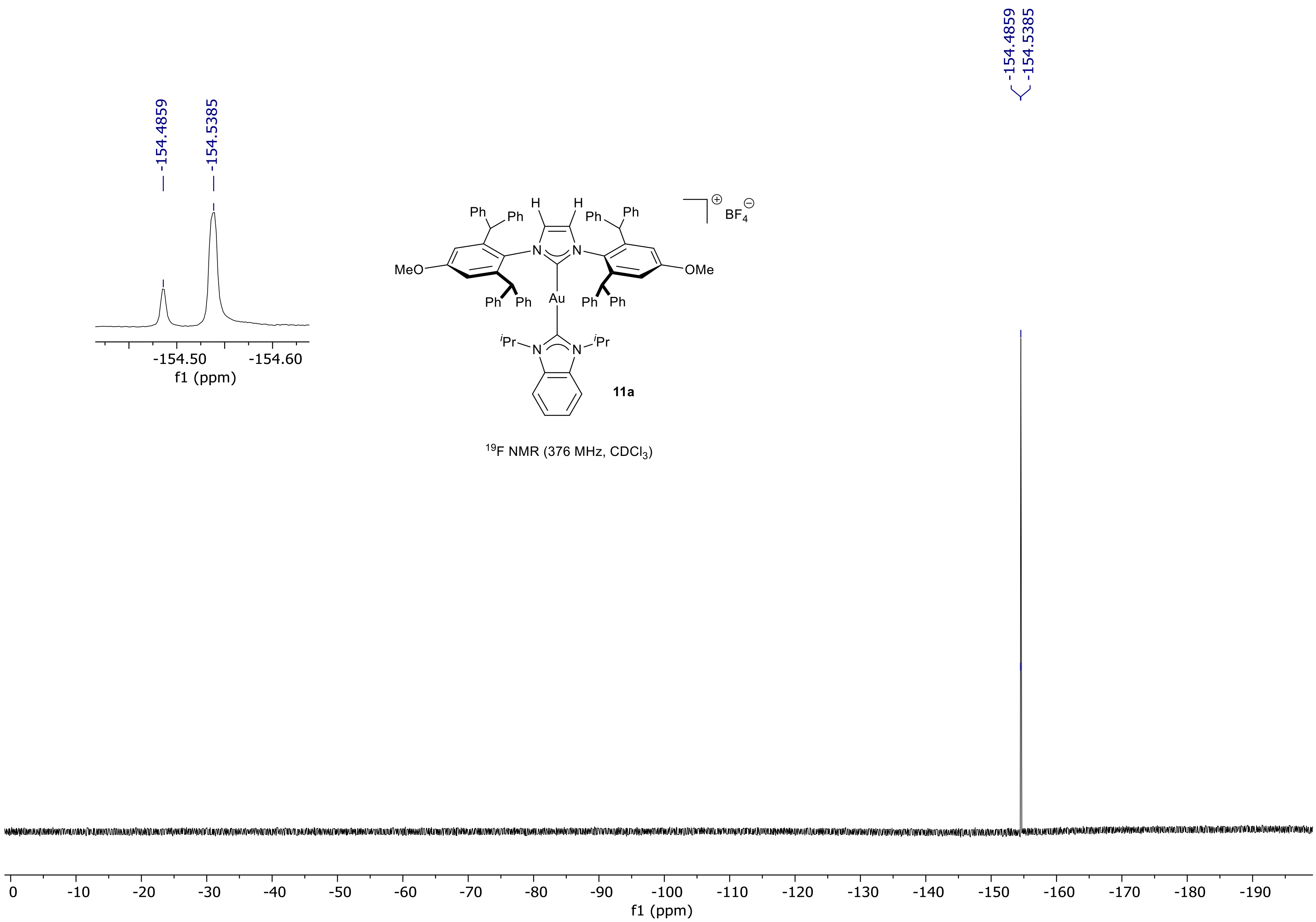

^{19}F NMR (376 MHz, $CDCl_3$)

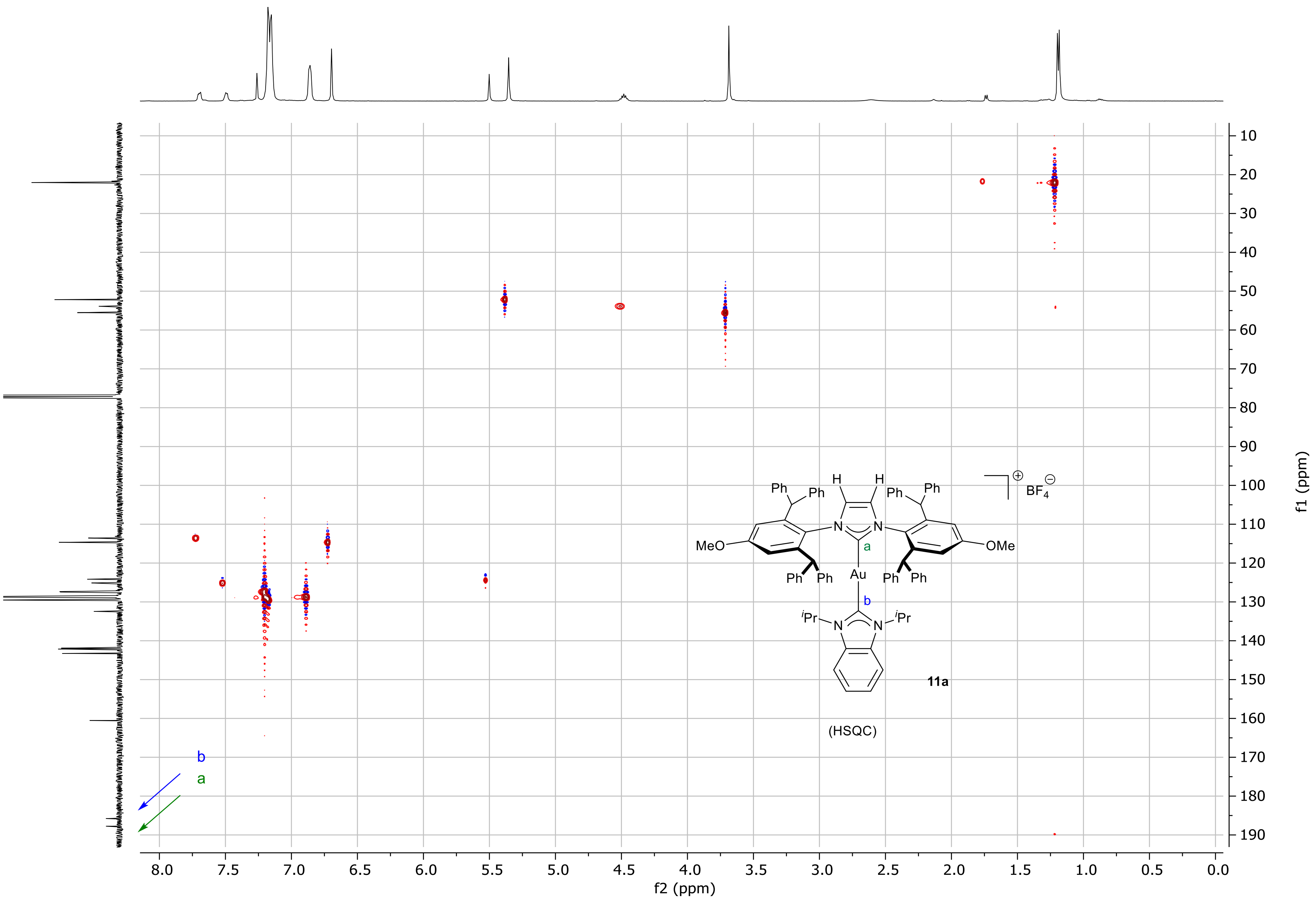

Ph
Ph
H
H
Ph
Ph
BF4
MeO
N
N
a
OMe
Ph
Ph
Au
Ph
Ph
b
iPr
N
N
iPr
11a
(HSQC)
b
a
f2 (ppm)
f1 (ppm)

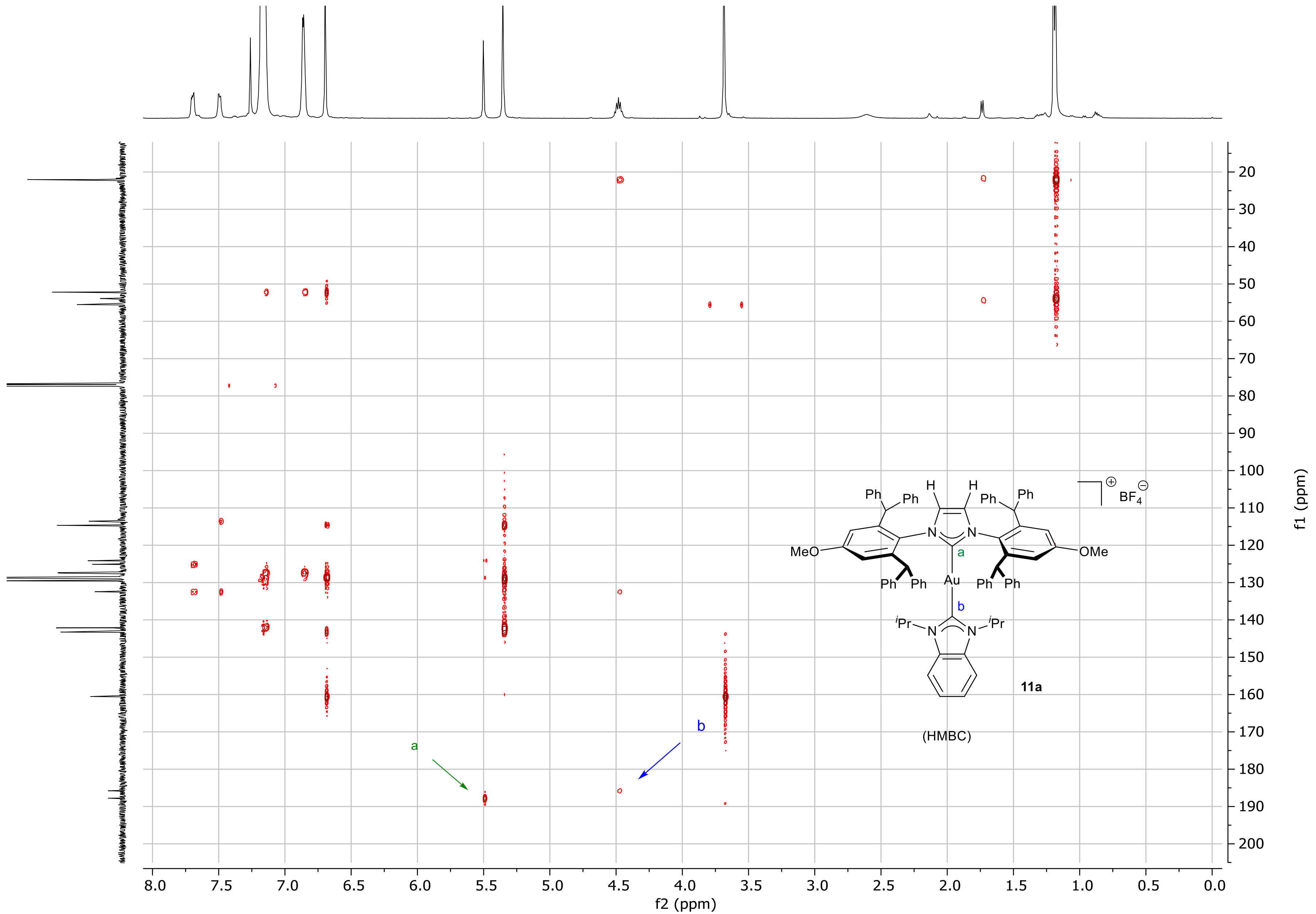

f2 (ppm)
f1 (ppm)
a
b
MeO
OMe
Ph
H
N
Au
^{i}Pr
BF_4
11a
(HMBC)

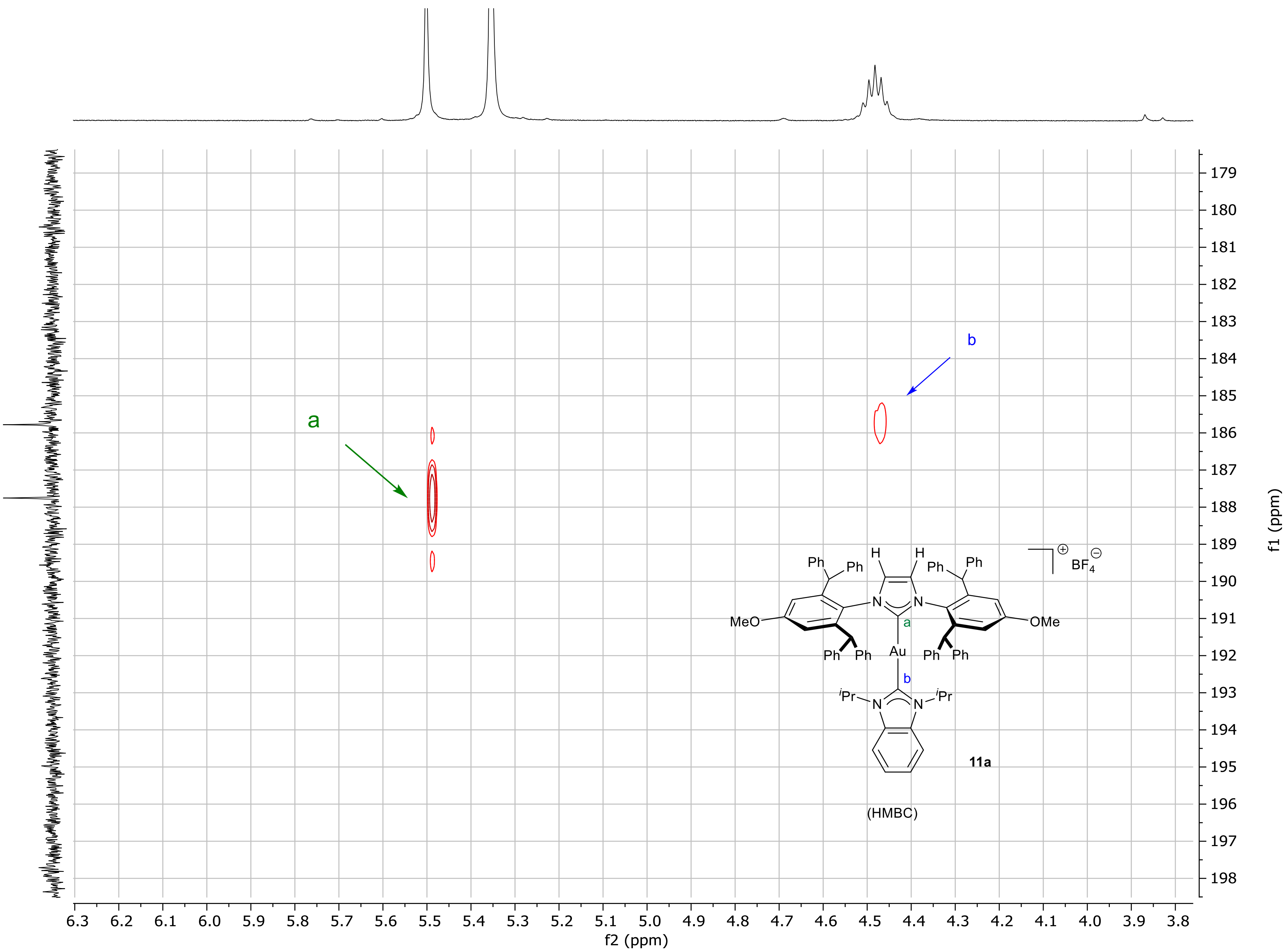
a
b
11a
(HMBC)
f2 (ppm)
f1 (ppm)
MeO
OMe
Au
BF4

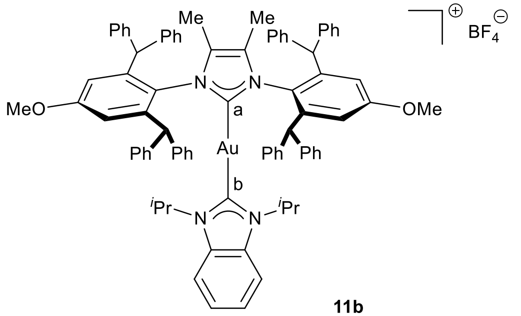

11b

^{1}H NMR (600 MHz, $CDCl_3$)

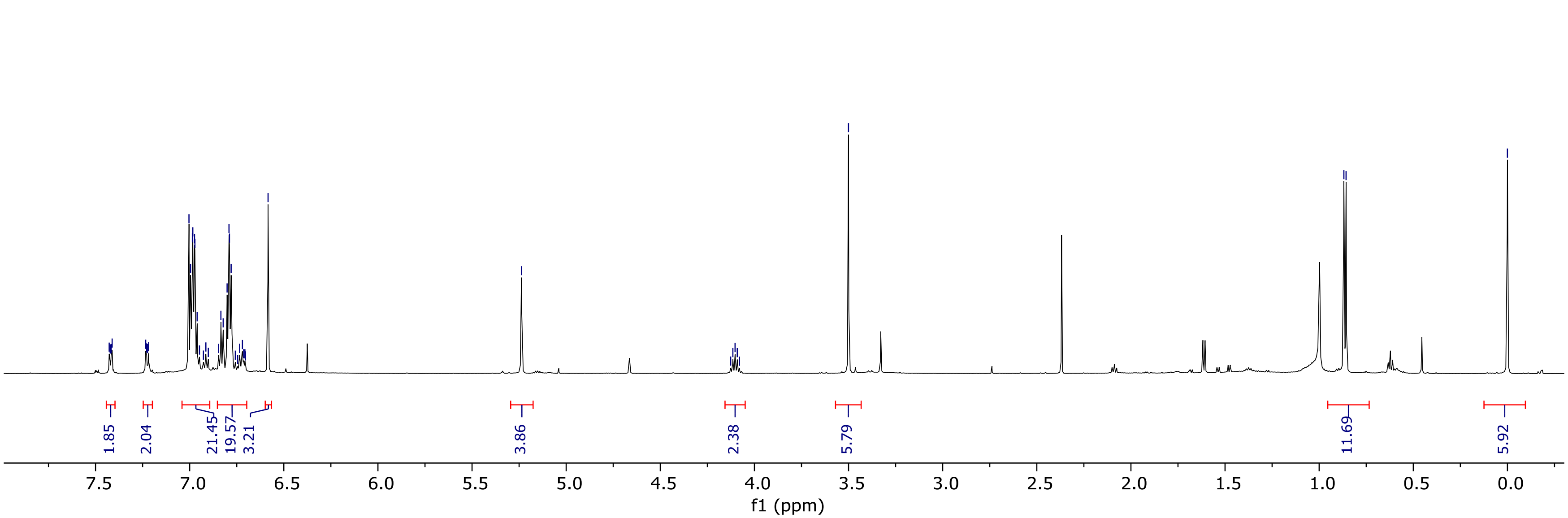

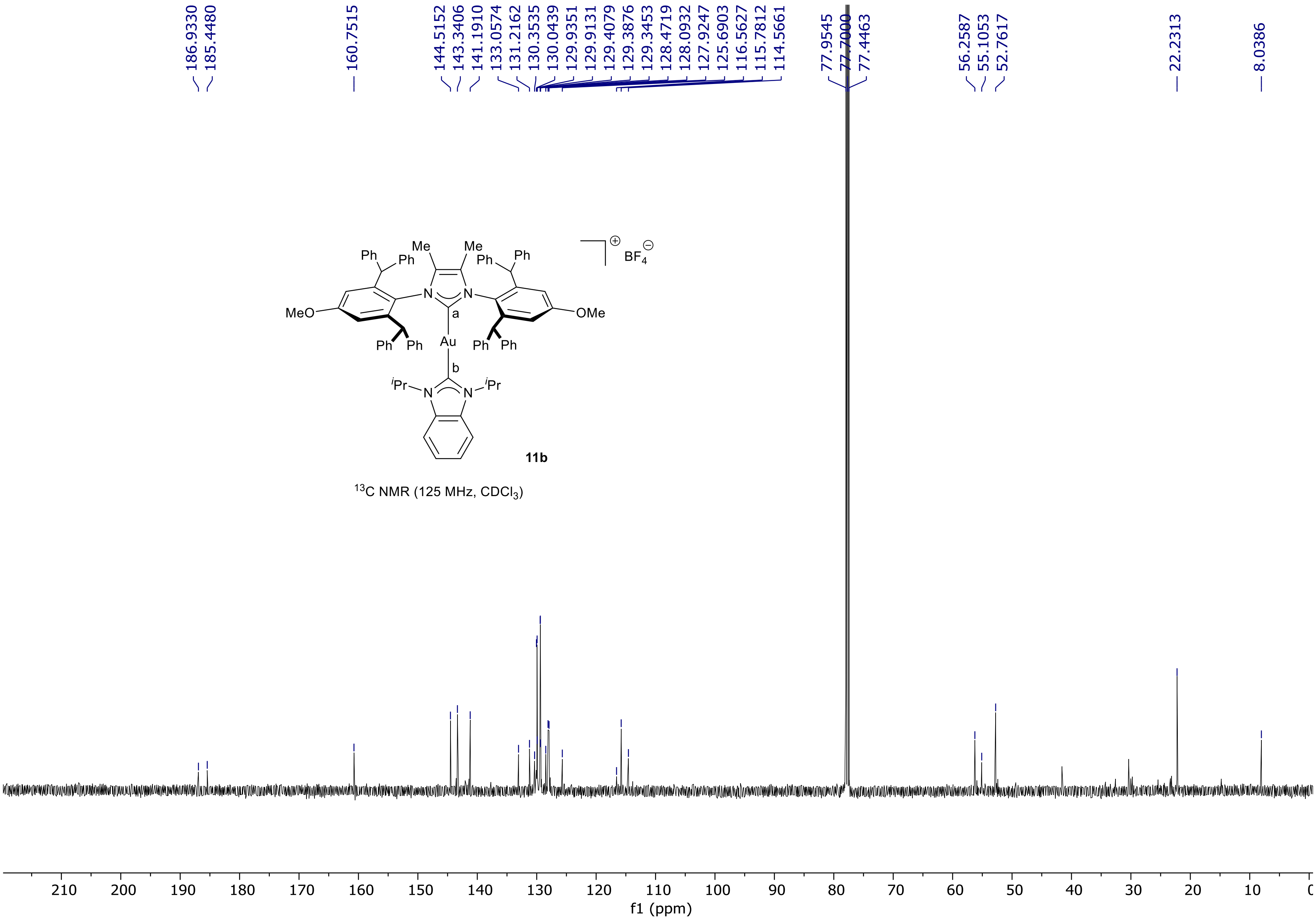
186.9330
185.4480
160.7515
144.5152
143.3406
141.1910
133.0574
131.2162
130.3535
130.0439
129.9351
129.9131
129.4079
129.3876
129.3453
128.4719
128.0932
127.9247
125.6903
116.5627
115.7812
114.5661
77.9545
77.7000
77.4463
56.2587
55.1053
52.7617
22.2313
8.0386
Me
Me
Ph
Ph
Ph
Ph
MeO
OMe
N
N
a
Au
b
Ph
Ph
Ph
Ph
BF4
iPr
iPr
N
N
11b
13C NMR (125 MHz, CDCl3)
210 200 190 180 170 160 150 140 130 120 110 100 90 80 70 60 50 40 30 20 10
f1 (ppm)

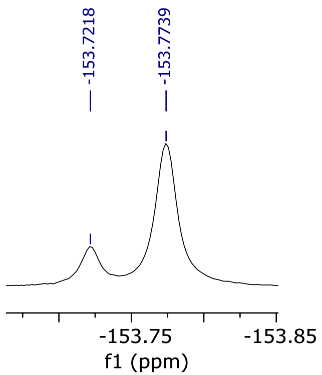

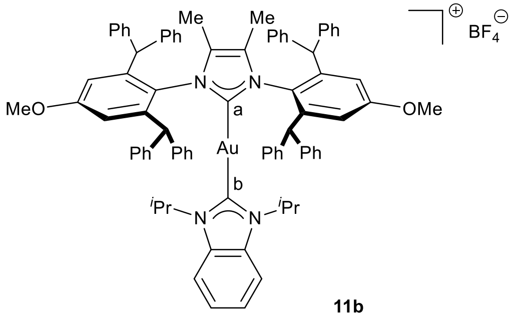

11b

^{19}F NMR (376 MHz, $CDCl_3$)

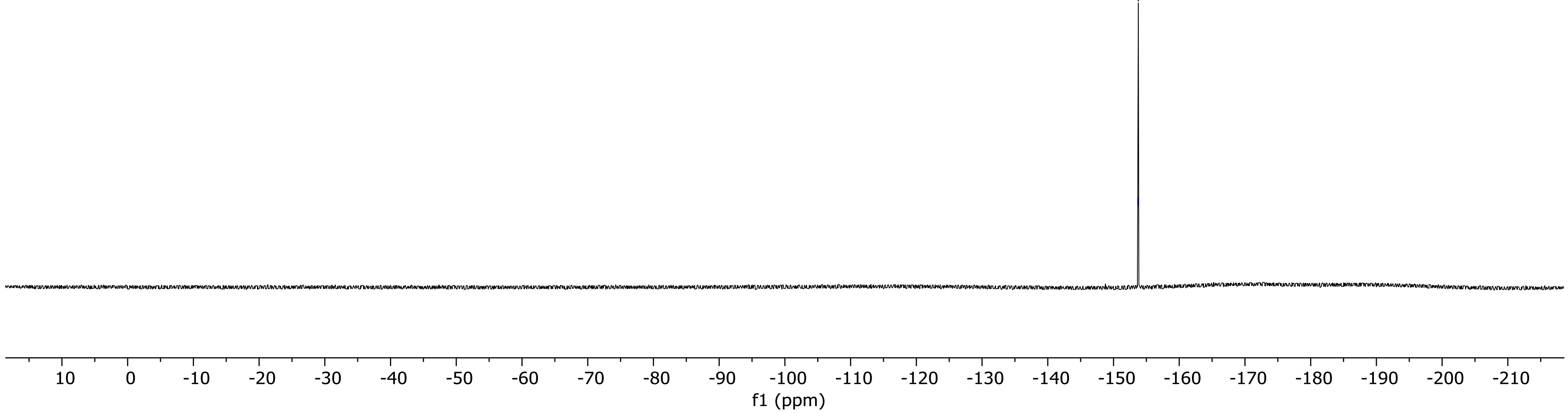

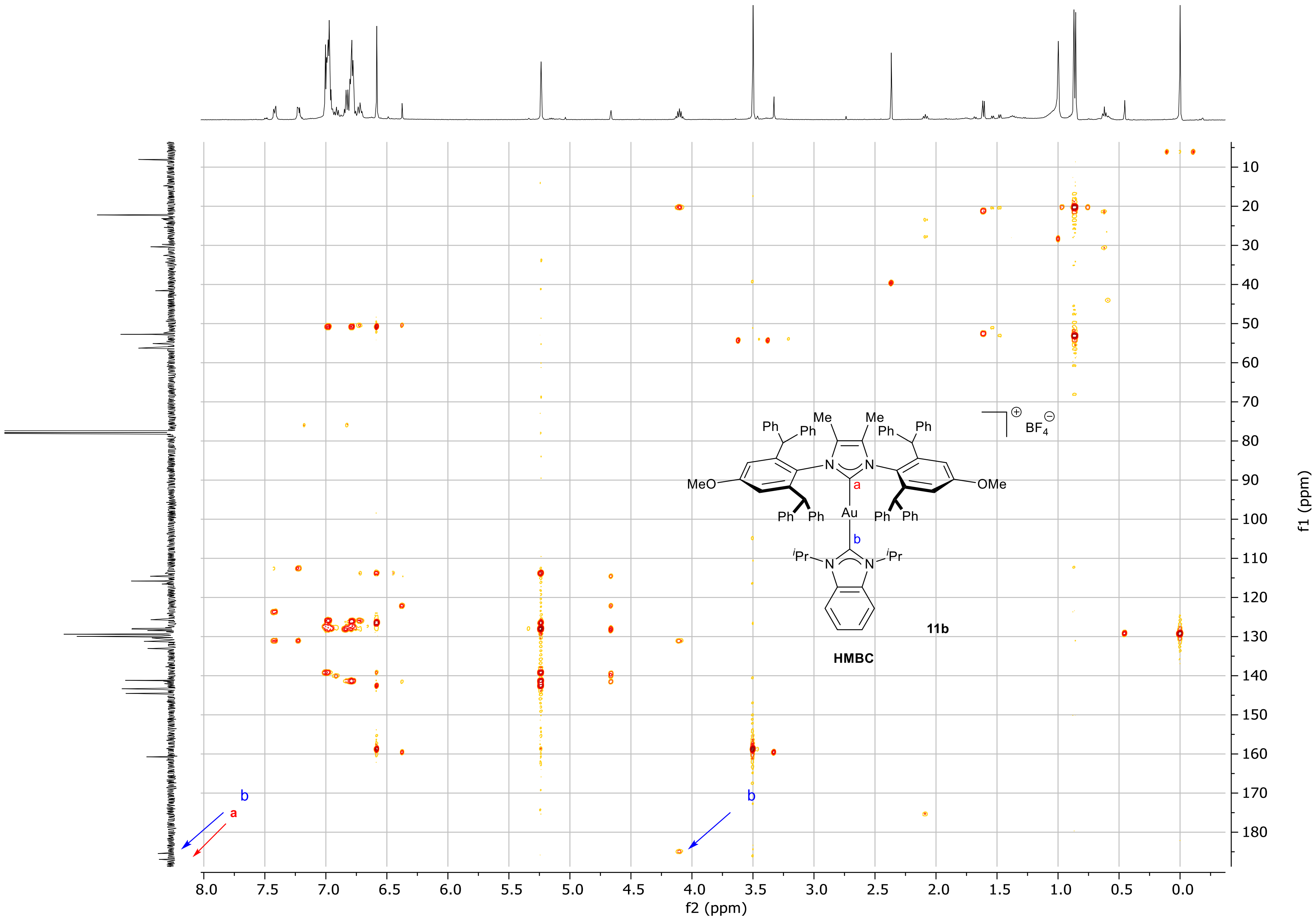

Me
Me
Ph
Ph
Ph
Ph
MeO
OMe
N
N
a
Au
b
Ph
Ph
Ph
Ph
iPr
iPr
BF4
11b
HMBC
b
a
b
f2 (ppm)
f1 (ppm)

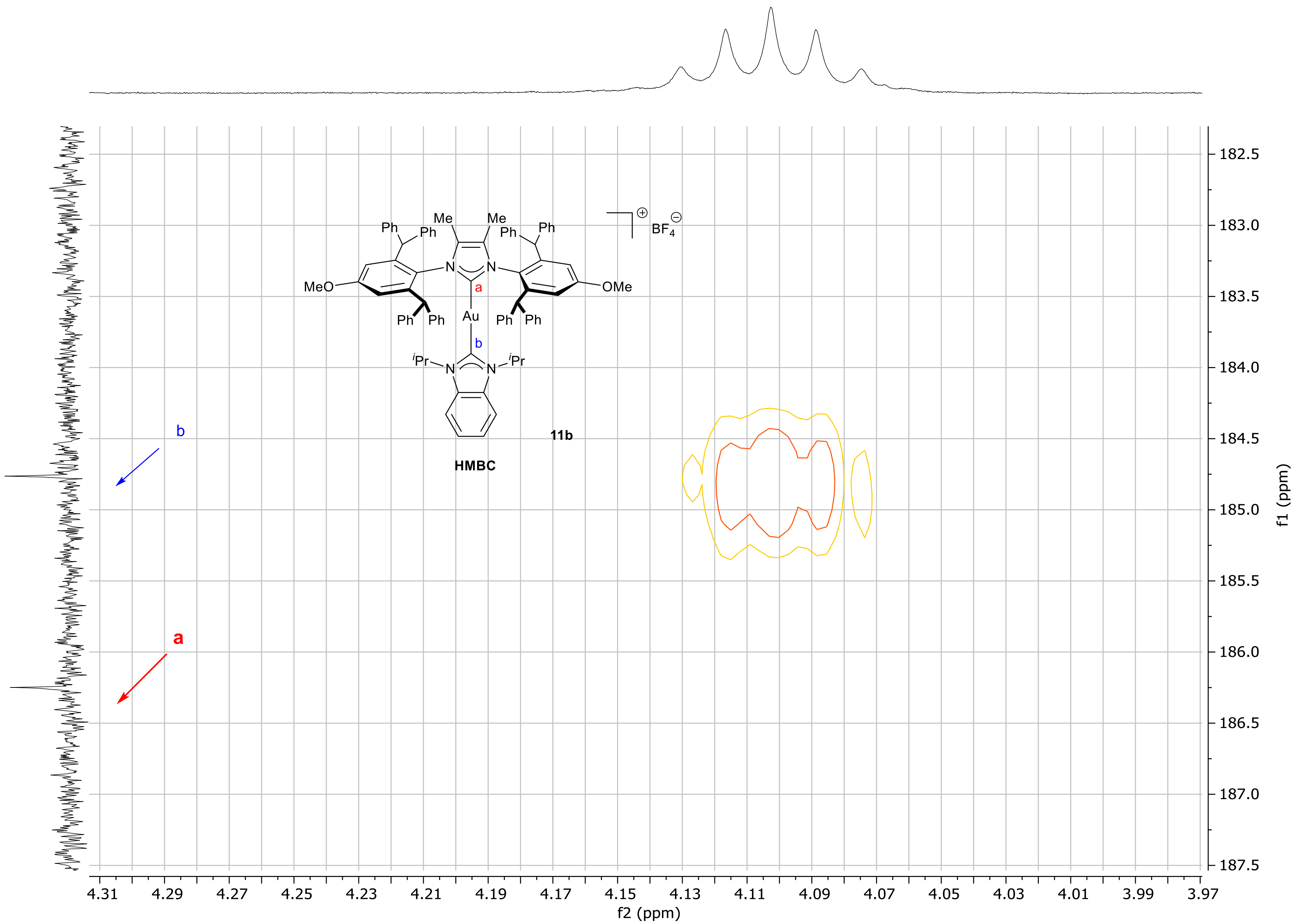
Me
Me
Ph
Ph
Ph
Ph
BF4
MeO
OMe
a
Au
Ph
Ph
Ph
Ph
b
iPr
iPr
N
N
N
N
11b
HMBC
b
a
f1 (ppm)
f2 (ppm)

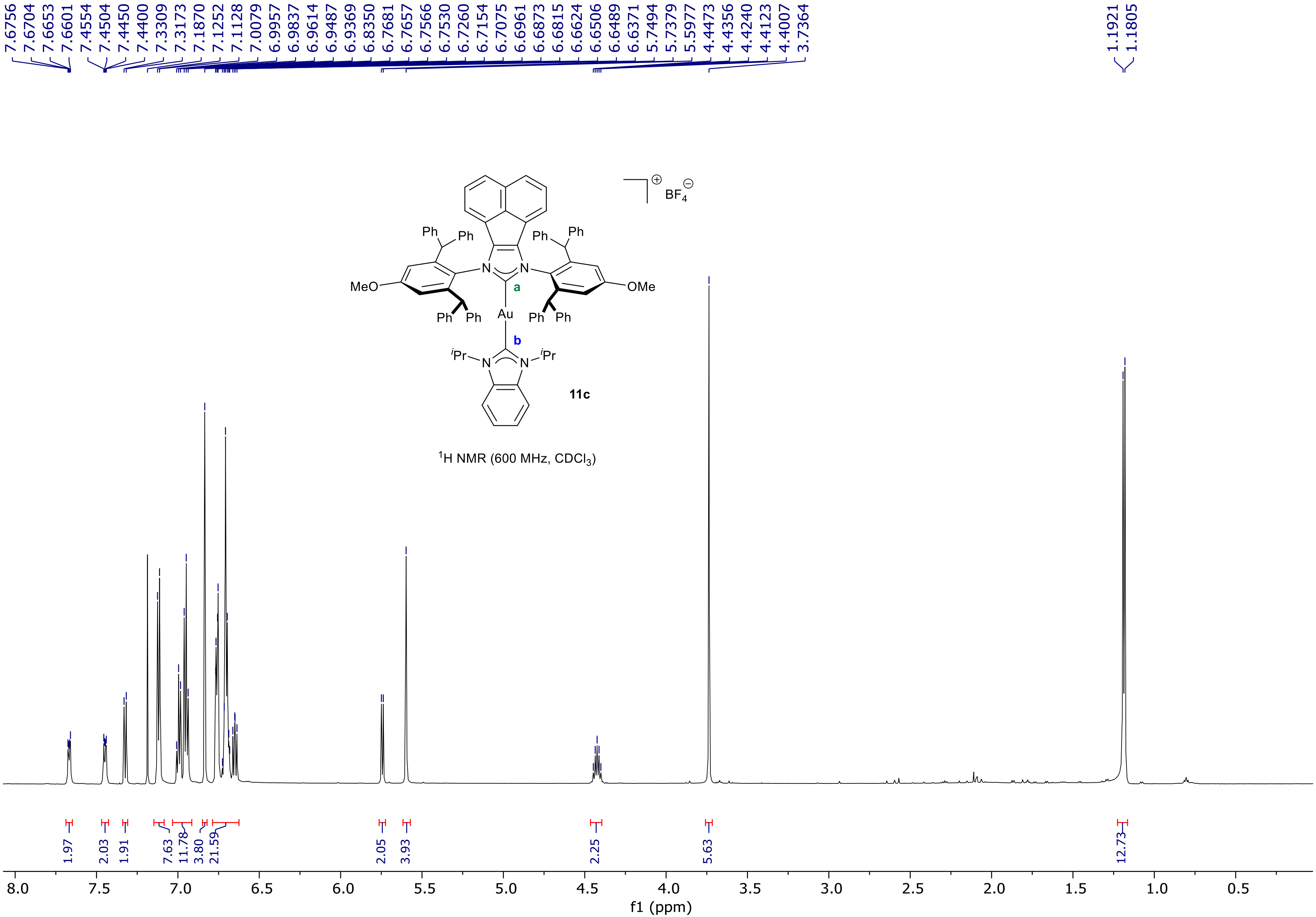
7.6756
7.6704
7.6653
7.6601
7.4554
7.4504
7.4450
7.4400
7.3309
7.3173
7.1870
7.1252
7.1128
7.0079
6.9957
6.9837
6.9614
6.9487
6.9369
6.8350
6.7681
6.7657
6.7566
6.7530
6.7260
6.7154
6.7075
6.6961
6.6873
6.6815
6.6624
6.6506
6.6489
6.6371
5.7494
5.7379
5.5977
4.4473
4.4356
4.4240
4.4123
4.4007
3.7364
1.1921
1.1805
BF4
Ph
MeO
OMe
a
Au
b
iPr
11c
1H NMR (600 MHz, CDCl3)
1.97
2.03
1.91
7.63
11.78
3.80
21.59
2.05
3.93
2.25
5.63
12.73
8.0
7.5
7.0
6.5
6.0
5.5
5.0
4.5
4.0
3.5
3.0
2.5
2.0
1.5
1.0
0.5
f1 (ppm)

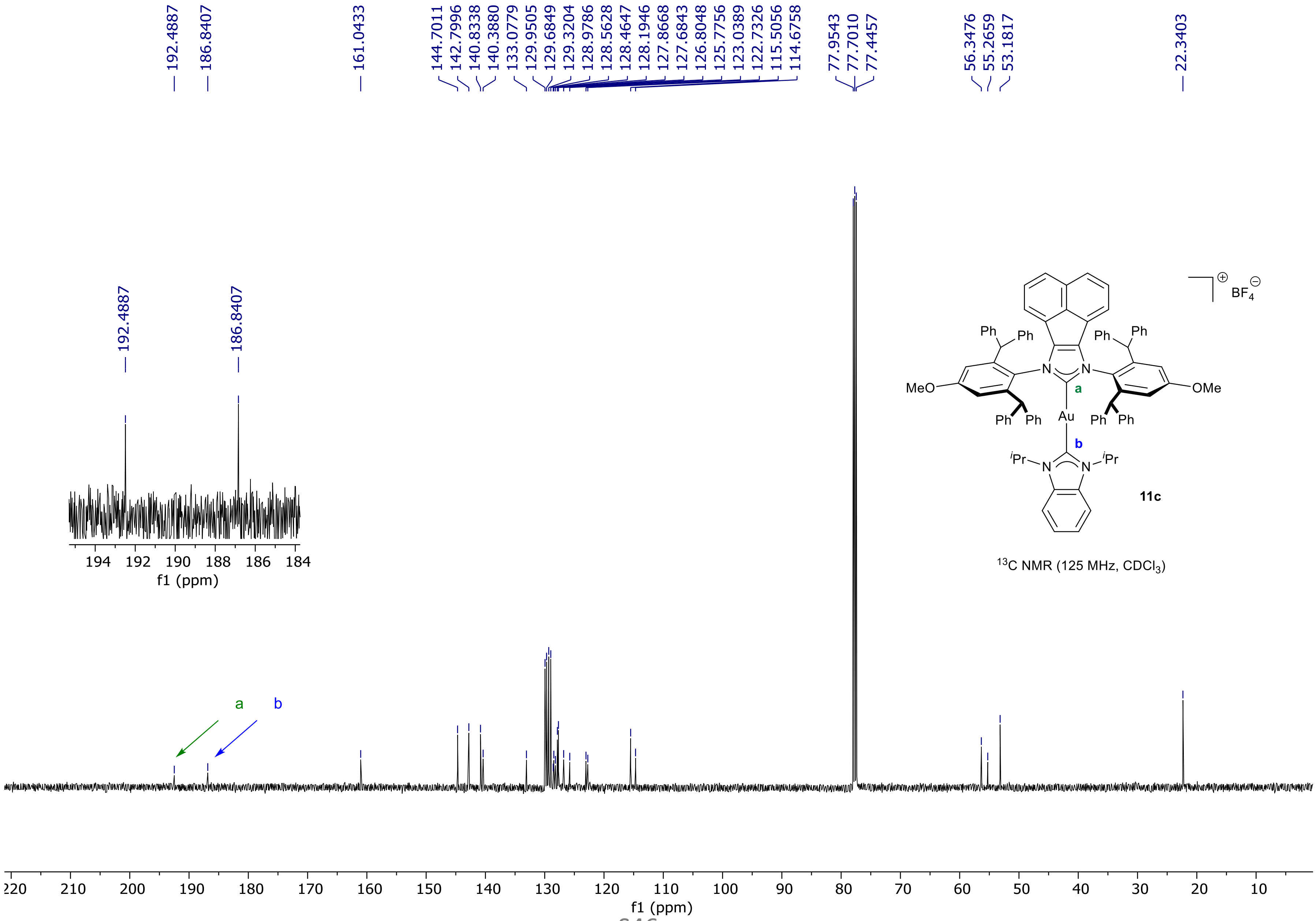
192.4887
186.8407
161.0433
144.7011
142.7996
140.8338
140.3880
133.0779
129.9505
129.6849
129.3204
128.9786
128.5628
128.4647
128.1946
127.8668
127.6843
126.8048
125.7756
123.0389
122.7326
115.5056
114.6758
77.9543
77.7010
77.4457
56.3476
55.2659
53.1817
22.3403
a
b
11c
^{13}C NMR (125 MHz, $CDCl_3$)
f1 (ppm)

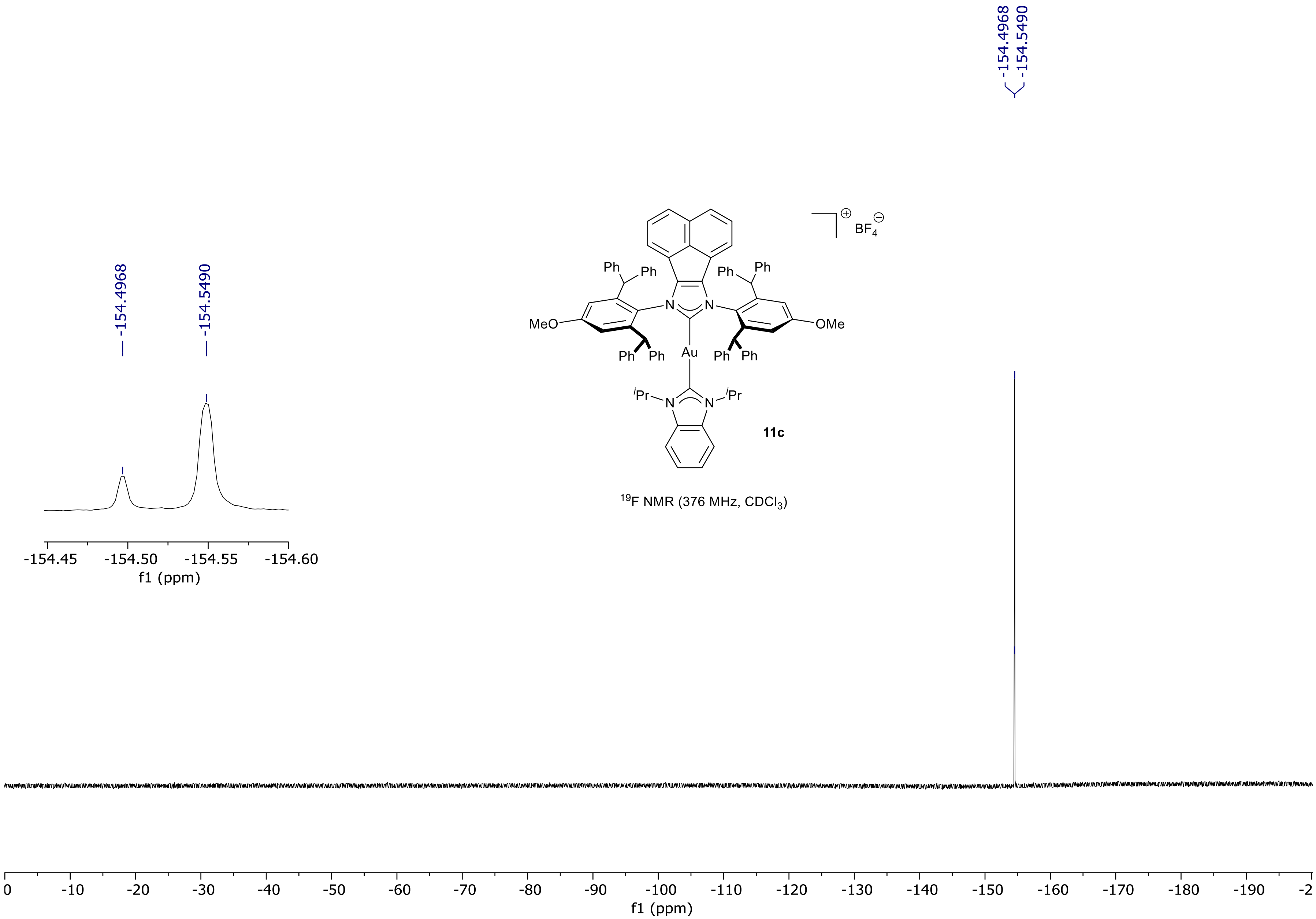

11c

^{19}F NMR (376 MHz, $CDCl_3$)

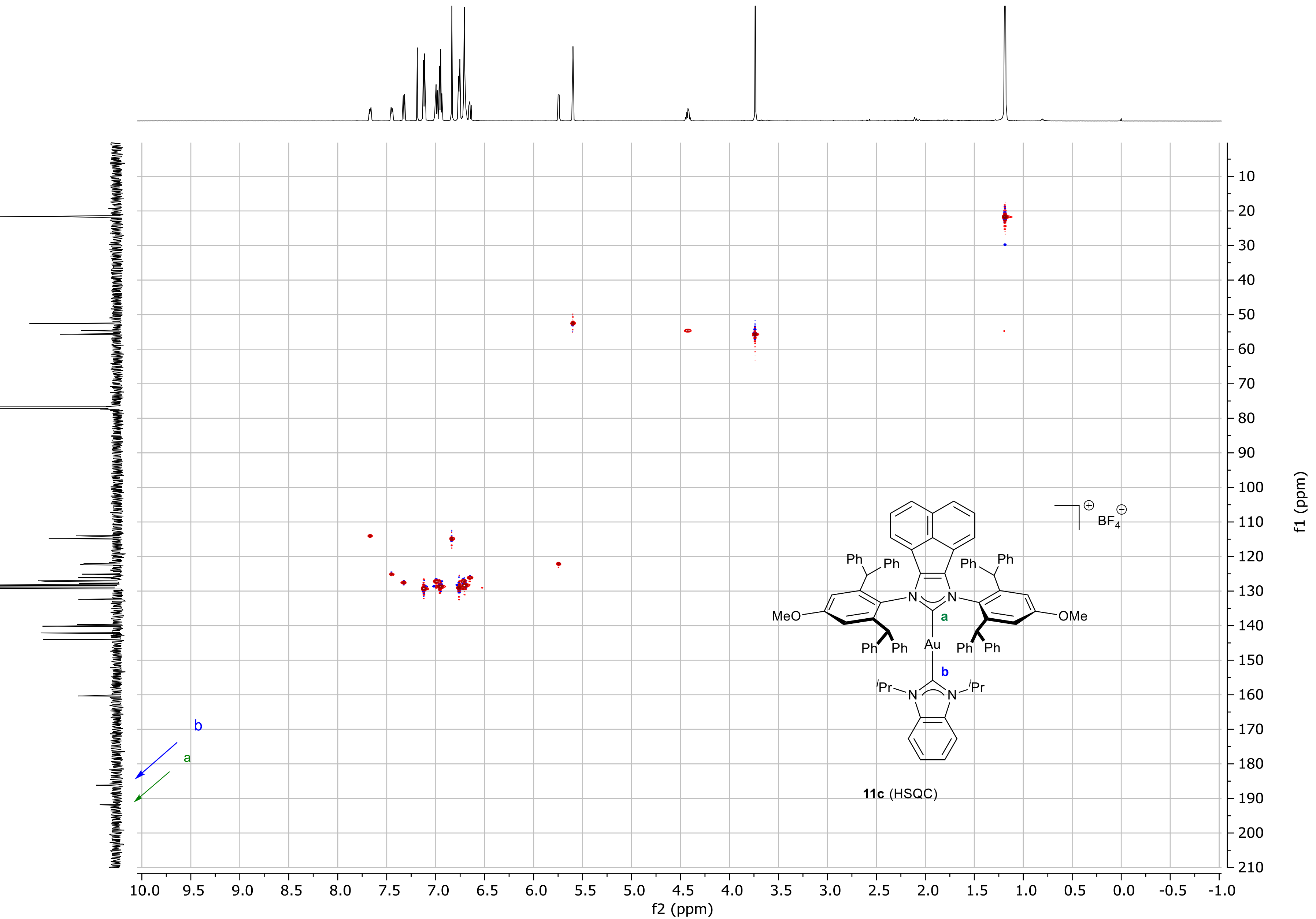

11c (HSQC)

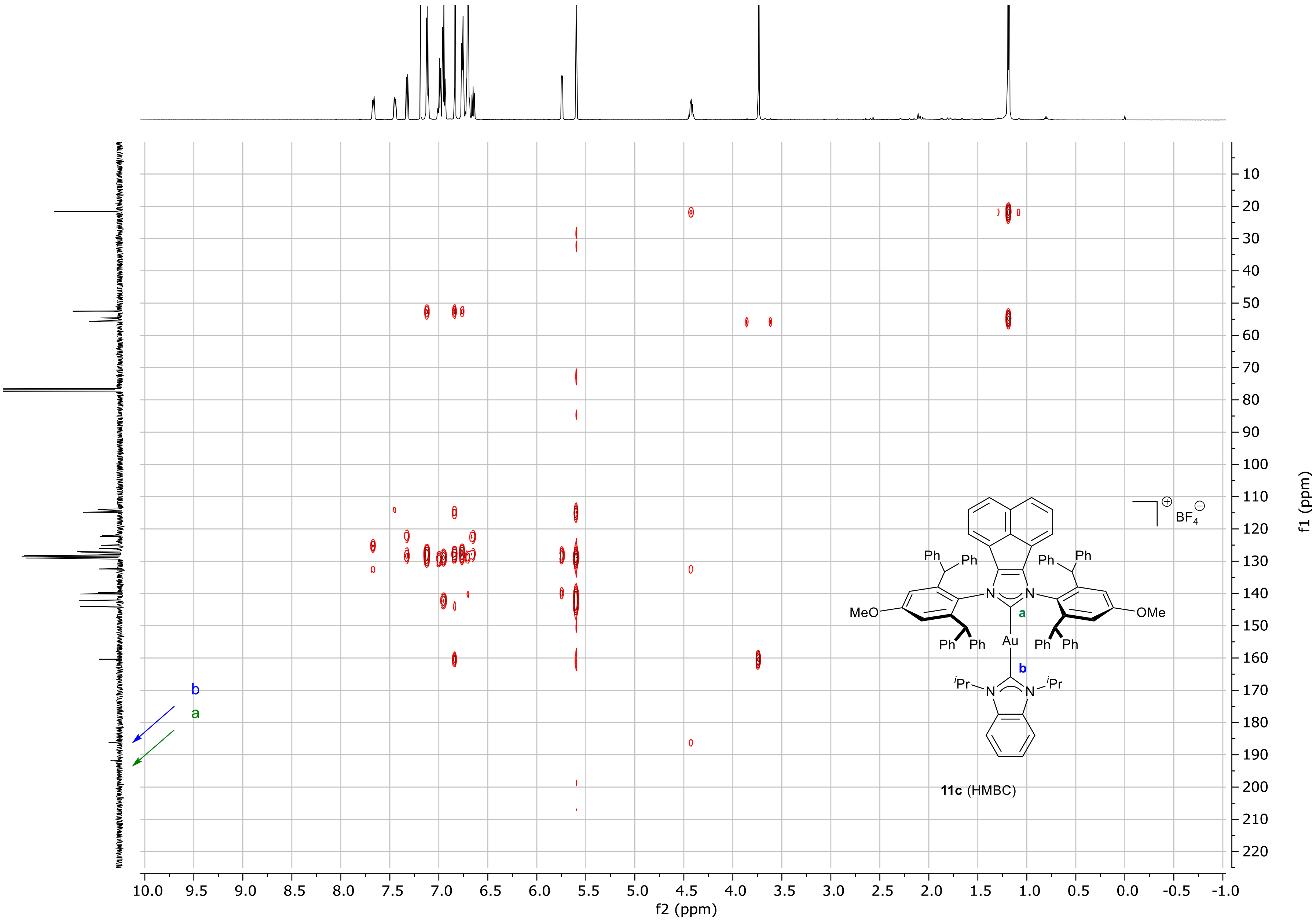

b
a
MeO
OMe
Ph
Au
iPr
BF4
11c (HMBC)
f2 (ppm)
f1 (ppm)

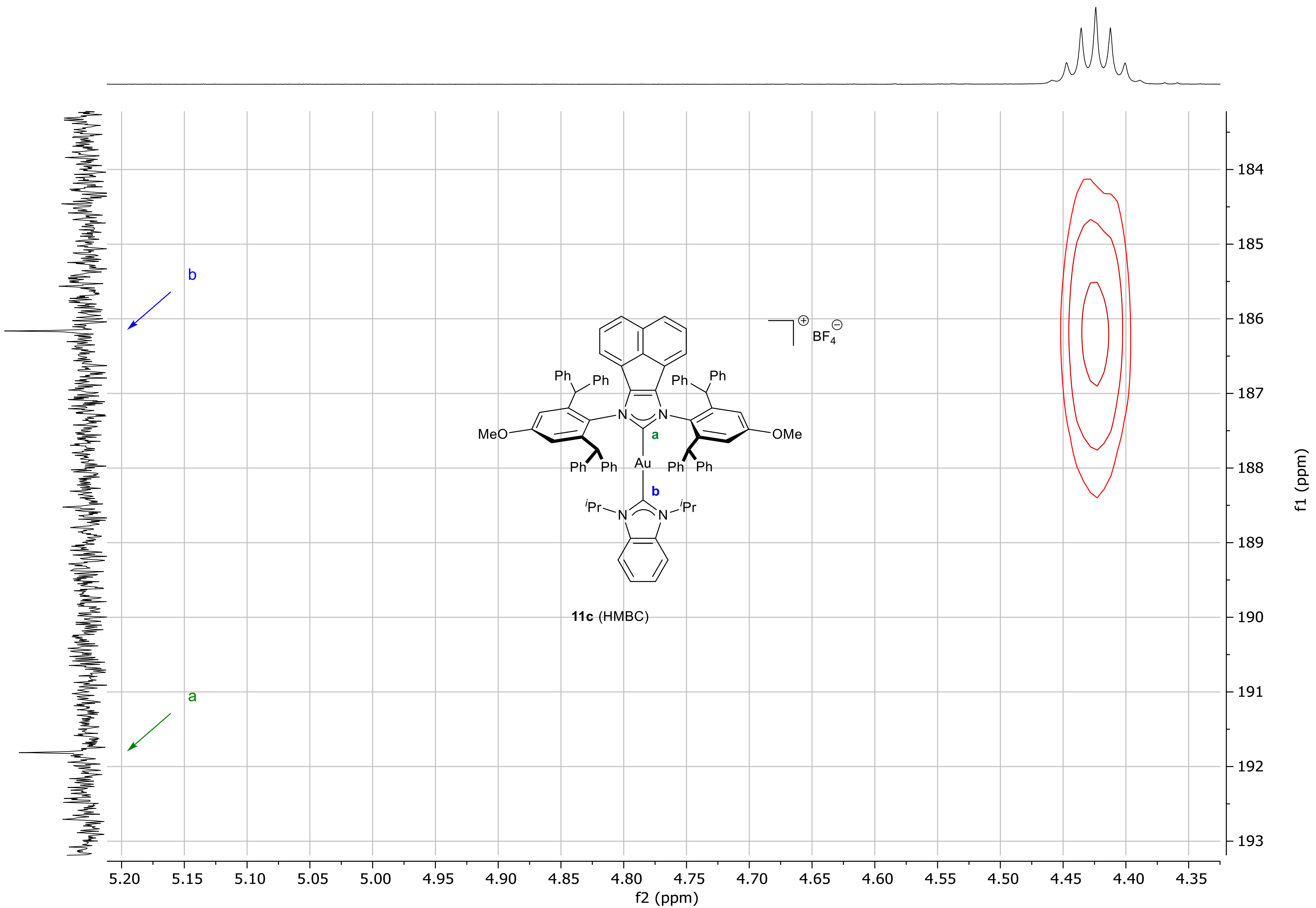
b
a
Ph
Ph
MeO
OMe
N
Au
BF4
iPr
11c (HMBC)
f2 (ppm)
f1 (ppm)
5.20
5.15
5.10
5.05
5.00
4.95
4.90
4.85
4.80
4.75
4.70
4.65
4.60
4.55
4.50
4.45
4.40
4.35
184
185
186
187
188
189
190
191
192
193

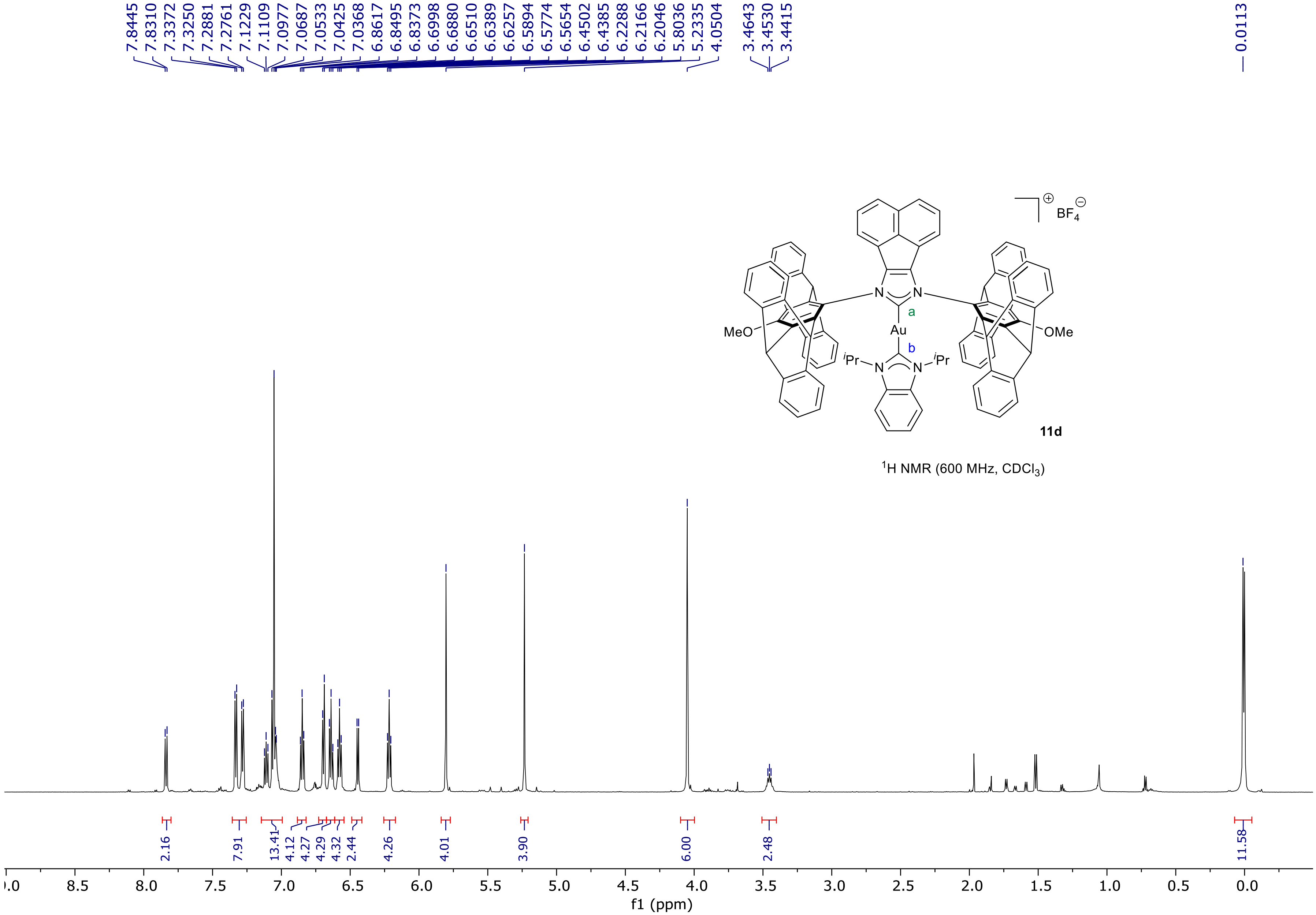
7.8445
7.8310
7.3372
7.3250
7.2881
7.2761
7.1229
7.1109
7.0977
7.0687
7.0533
7.0425
7.0368
6.8617
6.8495
6.8373
6.6998
6.6880
6.6510
6.6389
6.6257
6.5894
6.5774
6.5654
6.4502
6.4385
6.2288
6.2166
6.2046
5.8036
5.2335
4.0504
3.4643
3.4530
3.4415
0.0113
BF4
MeO
OMe
N
Au
a
b
iPr
11d
1H NMR (600 MHz, CDCl3)
2.16
7.91
13.41
4.12
4.27
4.29
4.32
2.44
4.26
4.01
3.90
6.00
2.48
11.58
8.5
8.0
7.5
7.0
6.5
6.0
5.5
5.0
4.5
4.0
3.5
3.0
2.5
2.0
1.5
1.0
0.5
0.0
f1 (ppm)

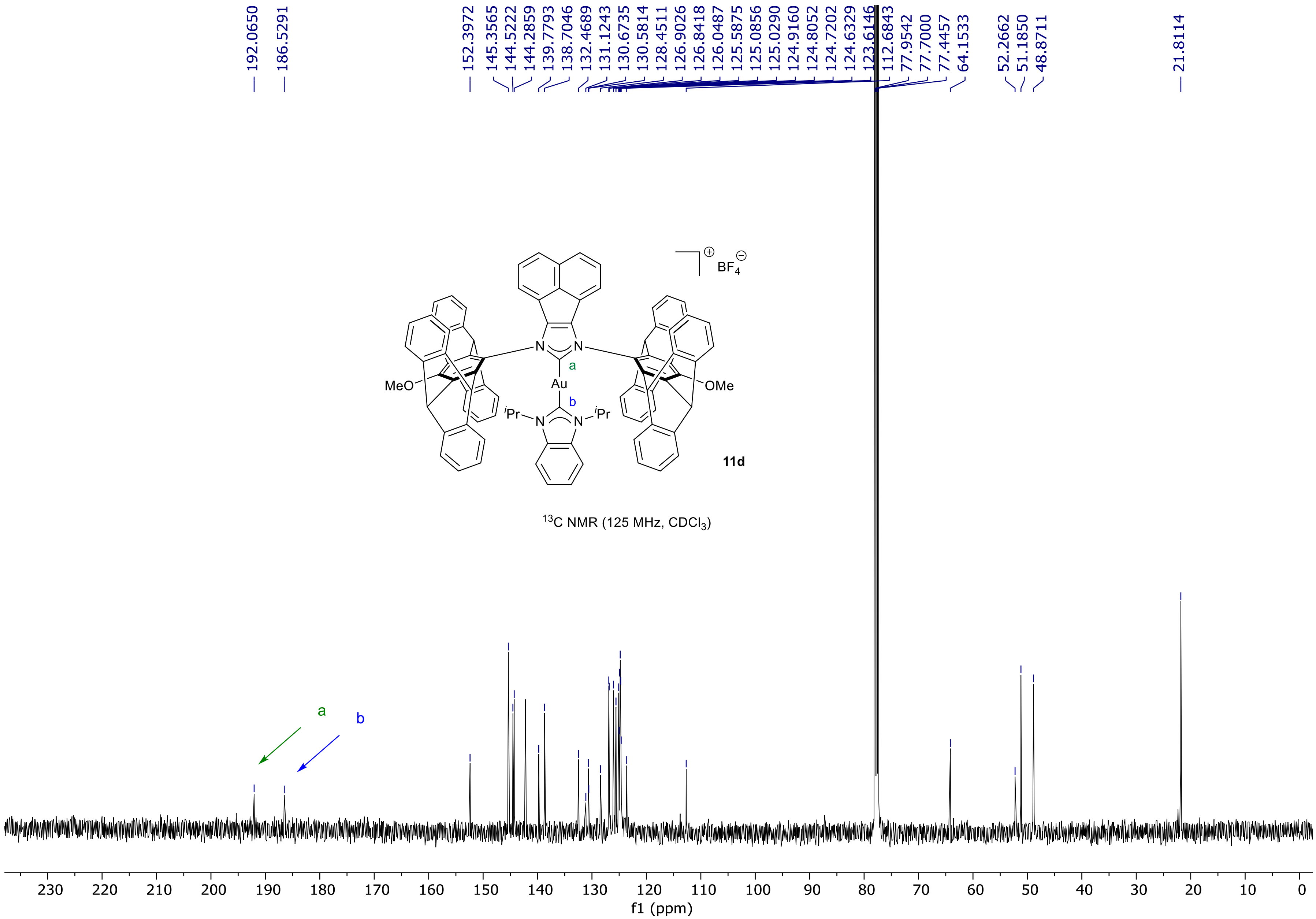
192.0650
186.5291
152.3972
145.3565
144.5222
144.2859
139.7793
138.7046
132.4689
131.1243
130.6735
130.5814
128.4511
126.9026
126.8418
126.0487
125.5875
125.0856
125.0290
124.9160
124.8052
124.7202
124.6329
123.6146
112.6843
77.9542
77.7000
77.4457
64.1533
52.2662
51.1850
48.8711
21.8114
BF4
MeO
OMe
Au
iPr
a
b
11d
13C NMR (125 MHz, CDCl3)
f1 (ppm)

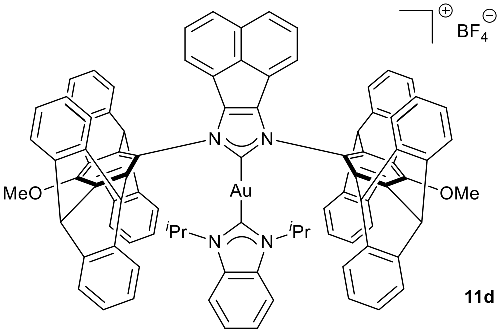

11d

^{19}F NMR (376 MHz, CDCl$_3$)

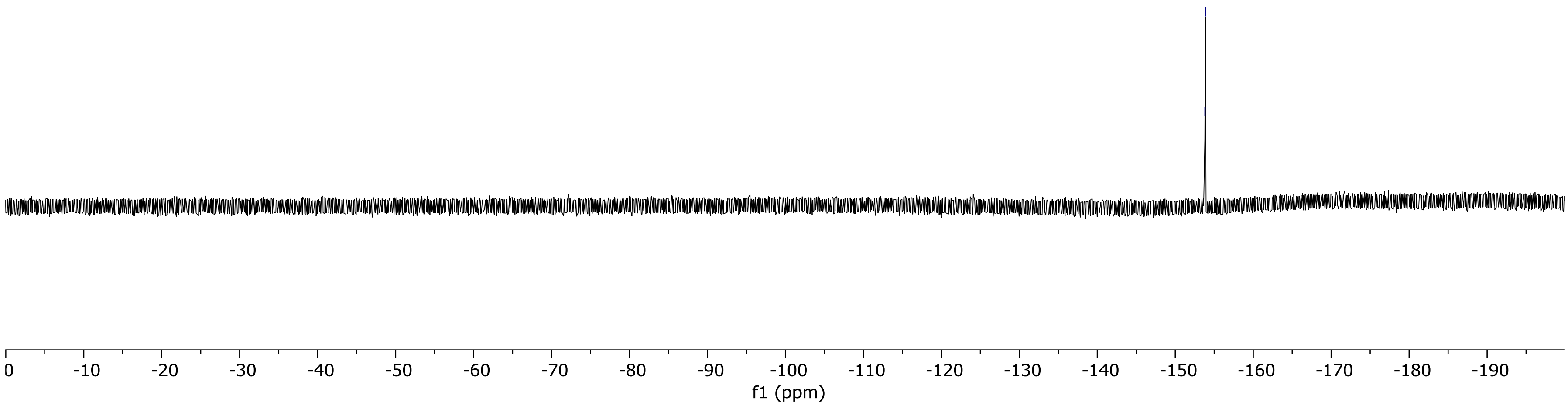

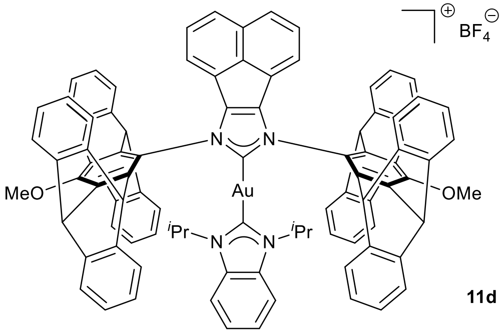

11d

^{19}F NMR (376 MHz, $CDCl_3$)

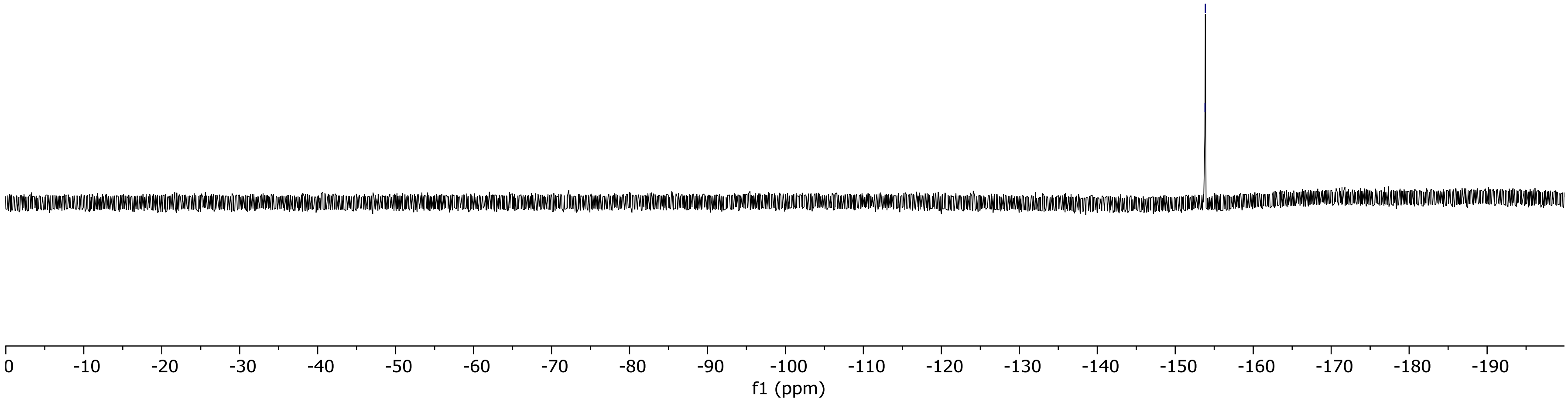

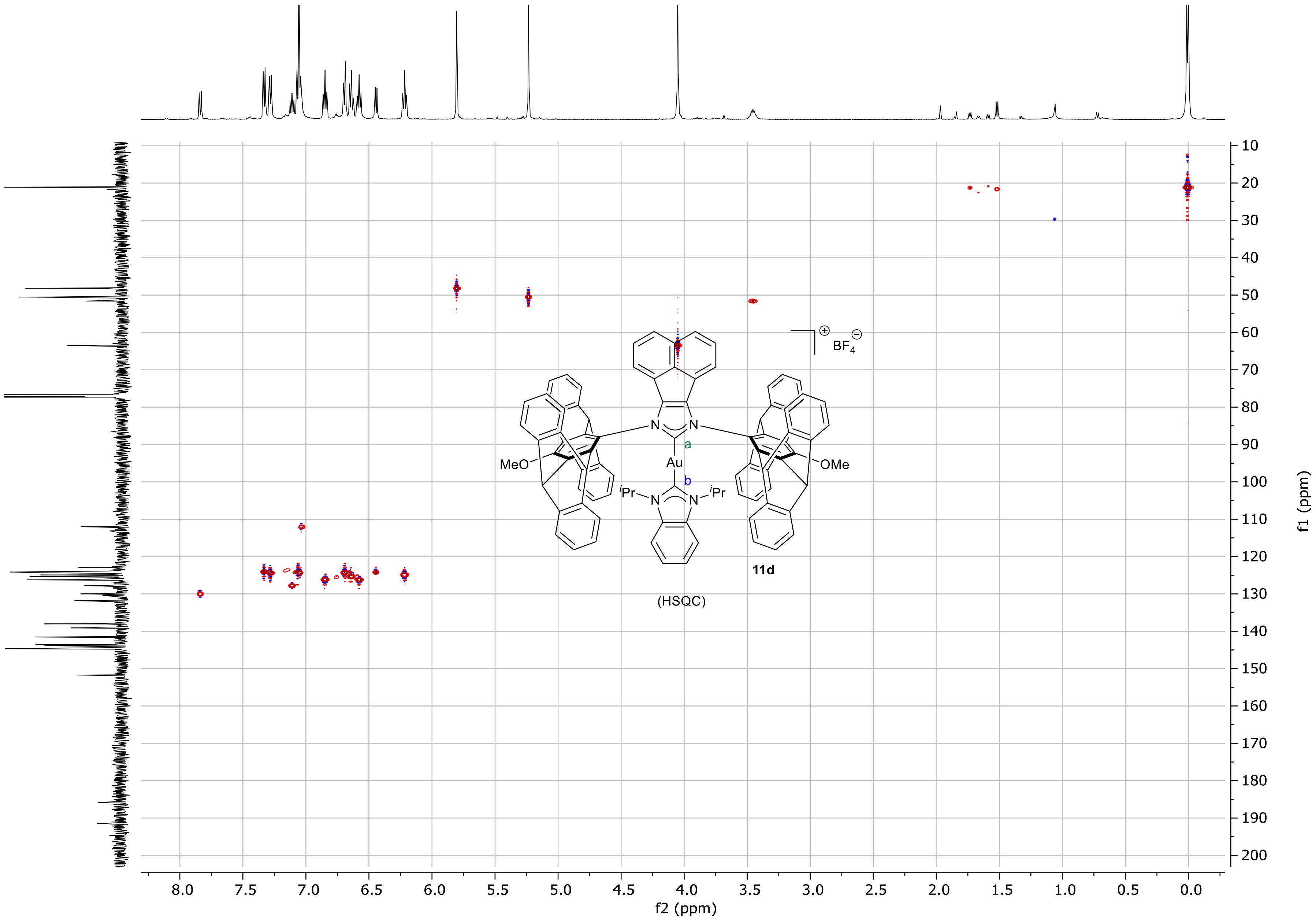

BF4
MeO
OMe
Au
a
b
iPr
iPr
N
11d
(HSQC)
f2 (ppm)
f1 (ppm)

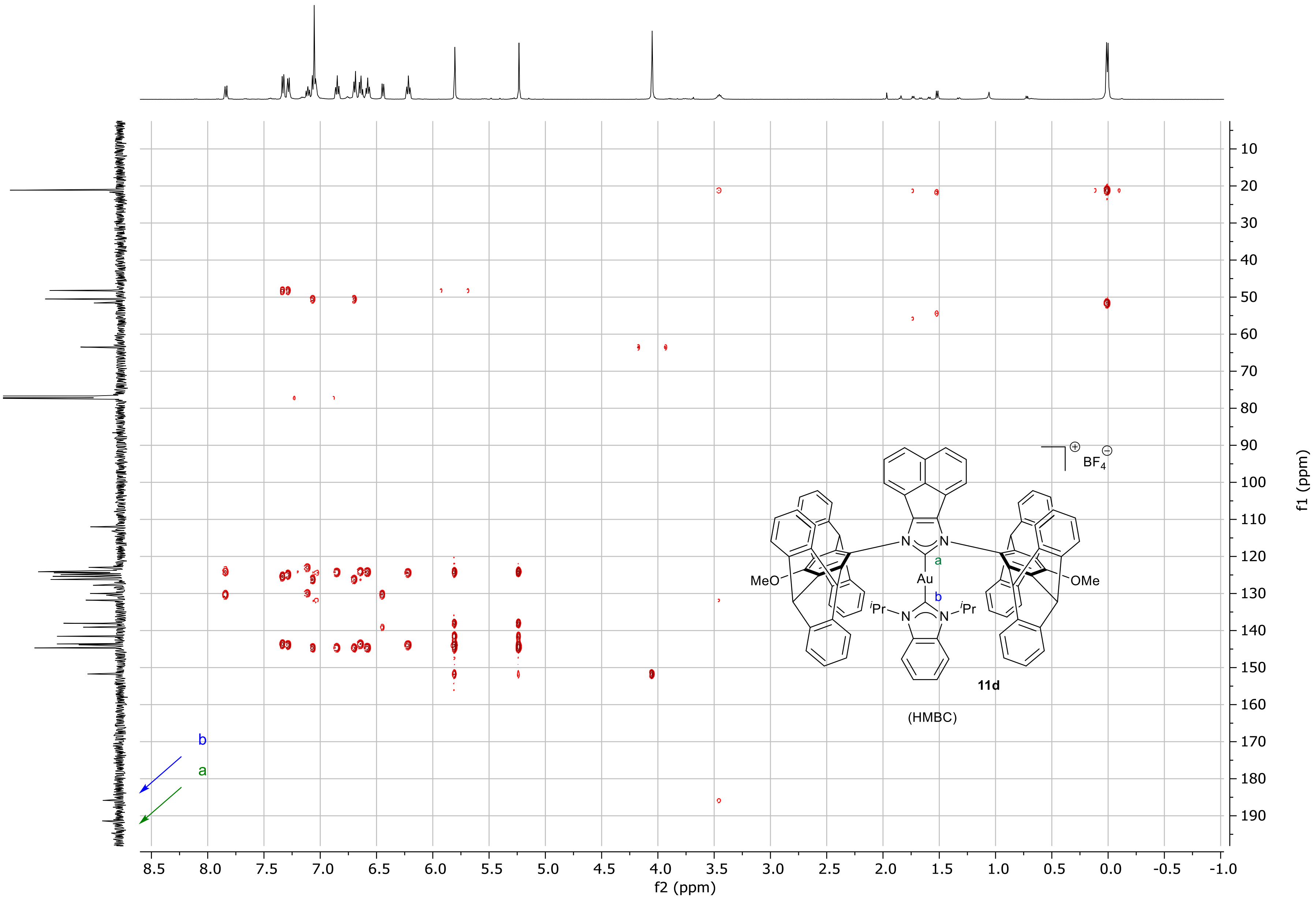
f2 (ppm)
f1 (ppm)
MeO
OMe
N
Au
iPr
BF4
11d
(HMBC)
a
b

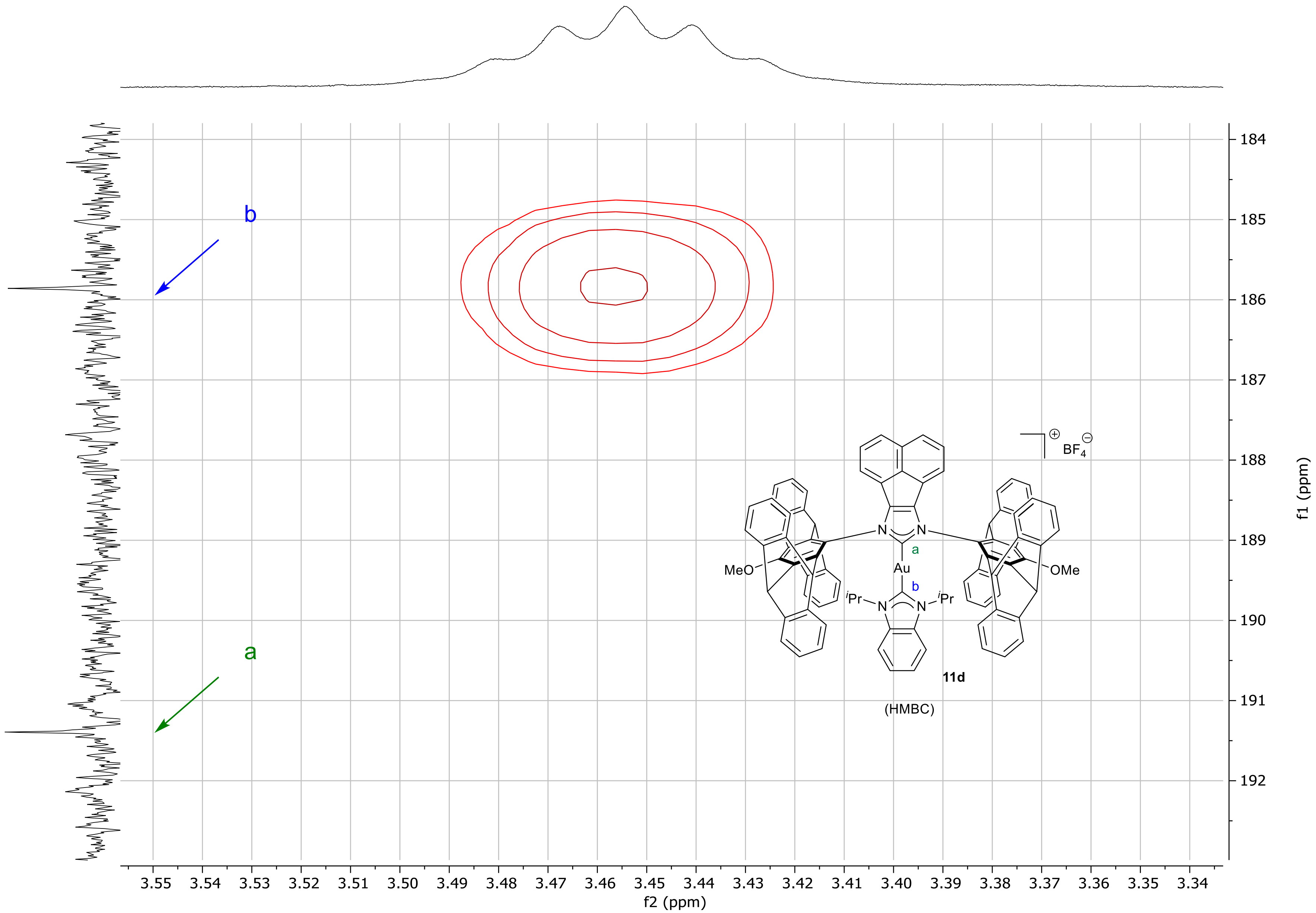
b
a
BF4
MeO
OMe
Au
N
iPr
a
b
11d
(HMBC)
f1 (ppm)
f2 (ppm)

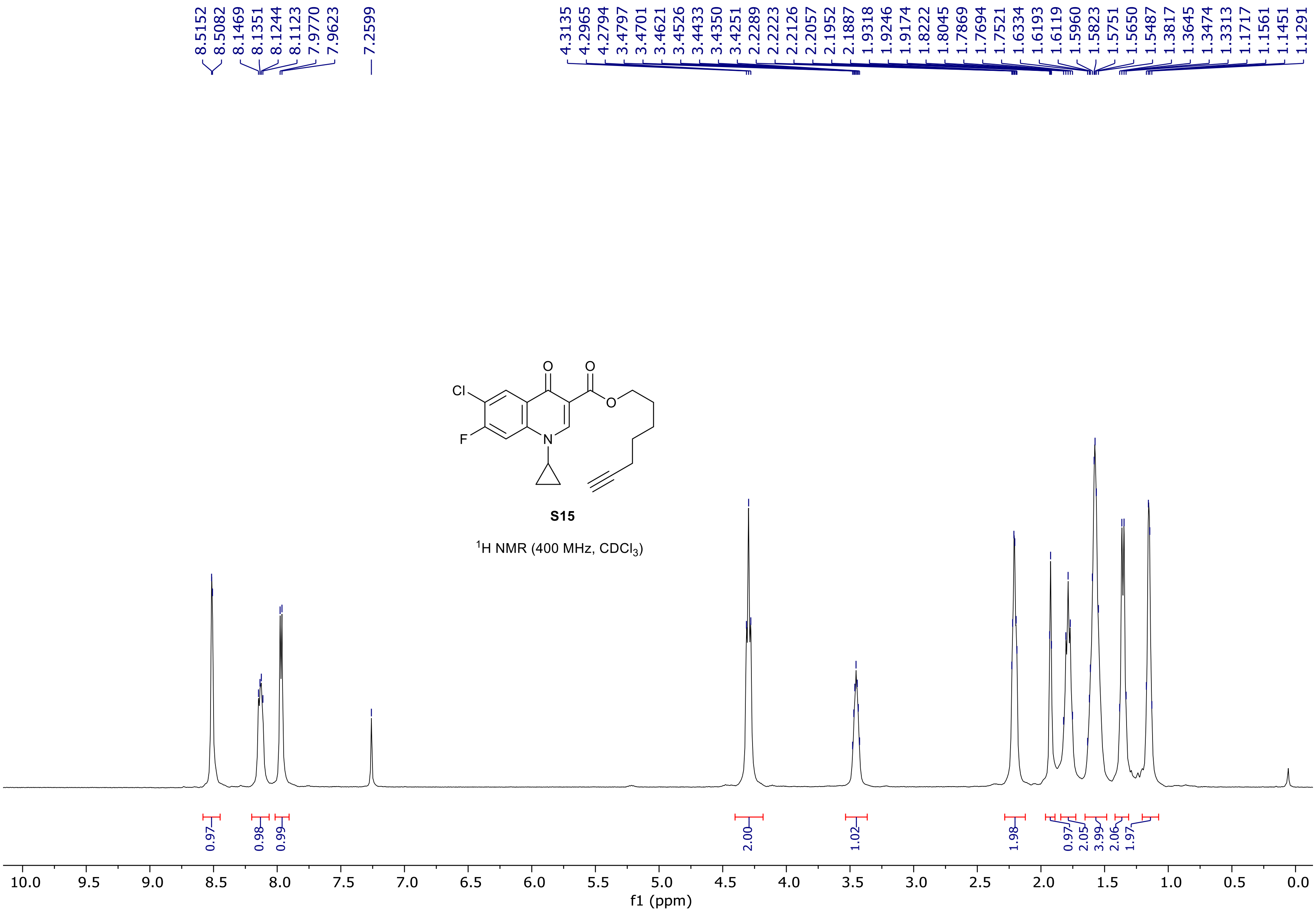
8.5152
8.5082
8.1469
8.1351
8.1244
8.1123
7.9770
7.9623
7.2599
4.3135
4.2965
4.2794
3.4797
3.4701
3.4621
3.4526
3.4433
3.4350
3.4251
2.2289
2.2223
2.2126
2.2057
2.1952
2.1887
1.9318
1.9246
1.9174
1.8222
1.8045
1.7869
1.7694
1.7521
1.6334
1.6193
1.6119
1.5960
1.5823
1.5751
1.5650
1.5487
1.3817
1.3645
1.3474
1.3313
1.1717
1.1561
1.1451
1.1291
Cl
F
O
O
O
N
S15
^{1}H NMR (400 MHz, $CDCl_3$)
0.97
0.98
0.99
2.00
1.02
1.98
0.97
2.05
3.99
2.06
1.97
10.0
9.5
9.0
8.5
8.0
7.5
7.0
6.5
6.0
5.5
5.0
4.5
4.0
3.5
3.0
2.5
2.0
1.5
1.0
0.5
0.0
f1 (ppm)

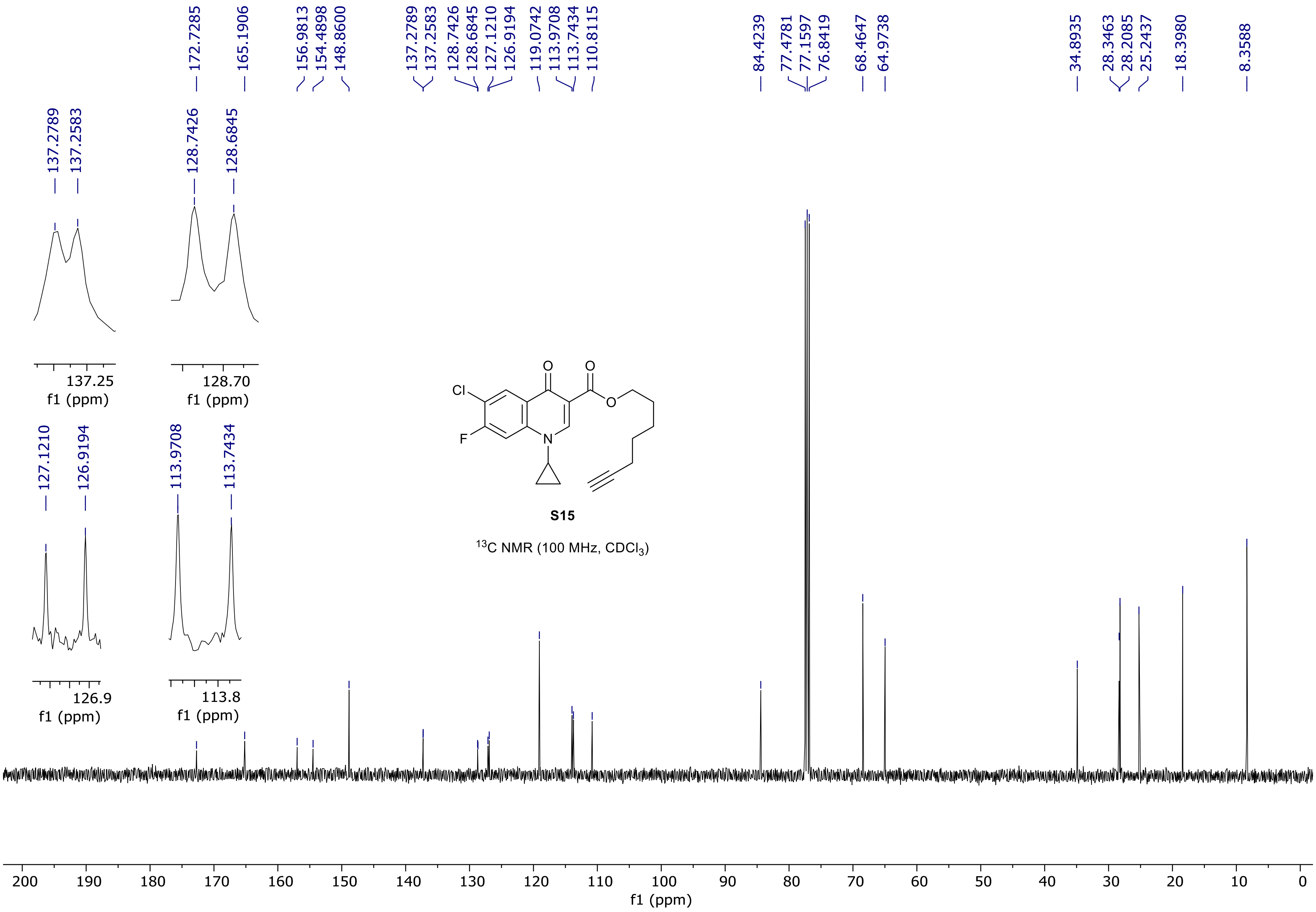

S15

^{13}C NMR (100 MHz, $CDCl_3$)

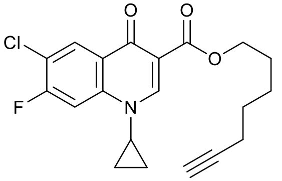

S15

^{19}F NMR (376 MHz, $CDCl_3$)

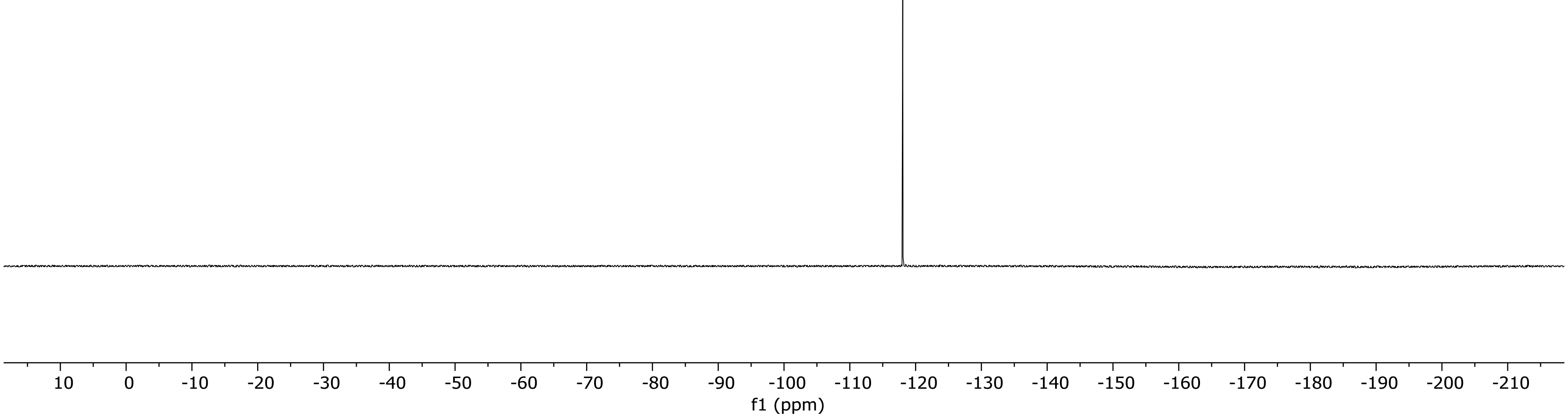

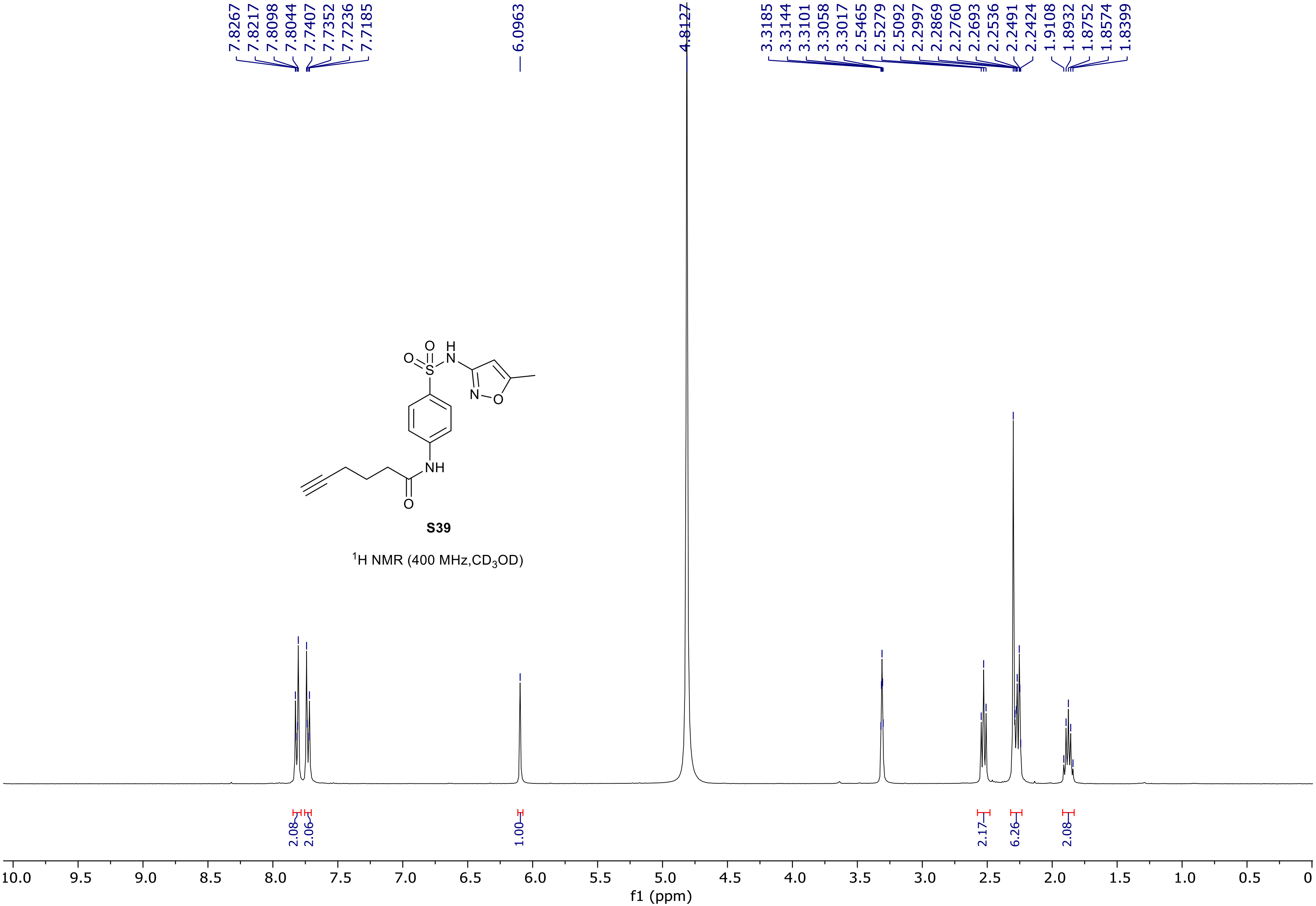

S39

^{1}H NMR (400 MHz,CD$_3$OD)

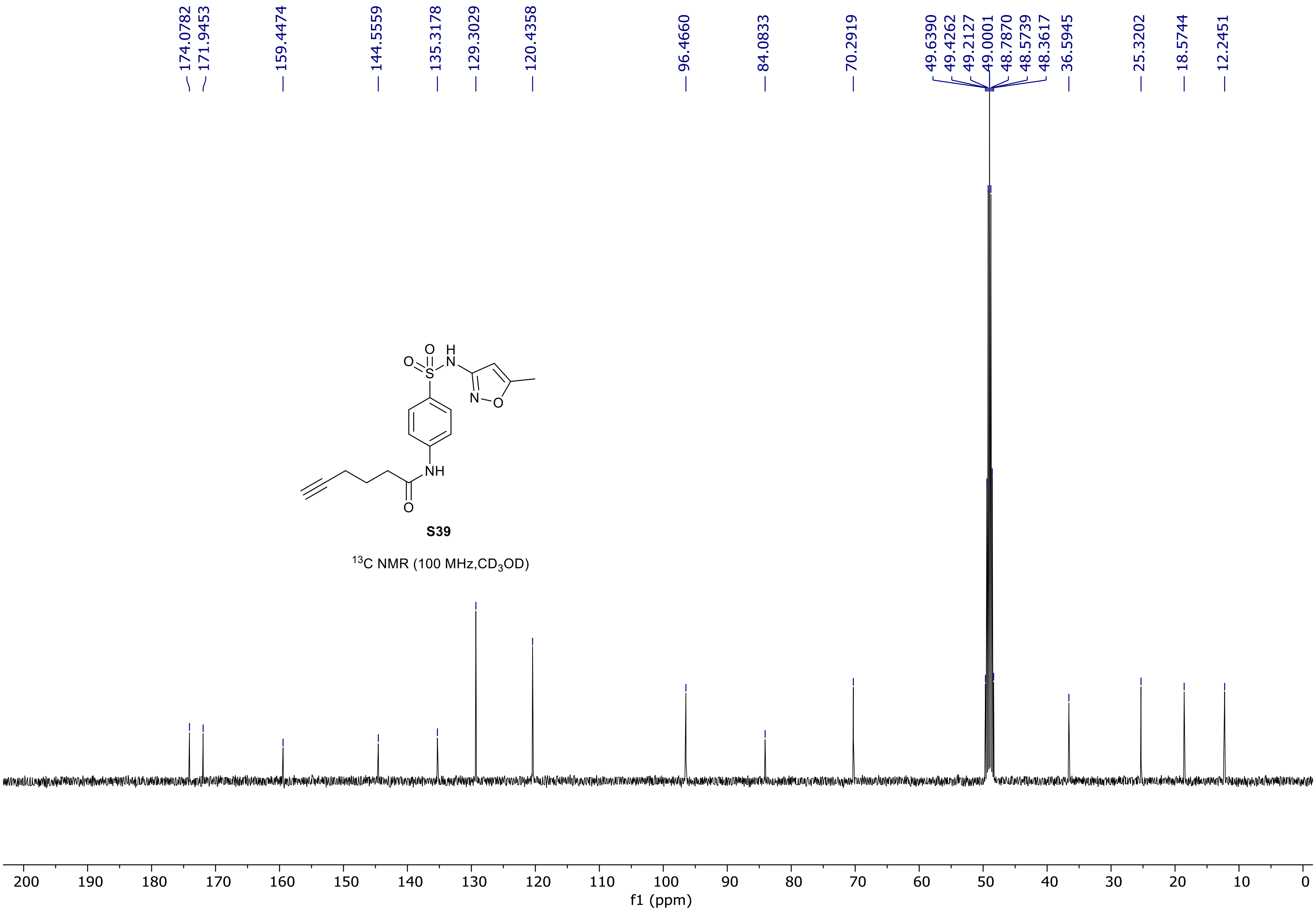
174.0782
171.9453
159.4474
144.5559
135.3178
129.3029
120.4358
96.4660
84.0833
70.2919
49.6390
49.4262
49.2127
49.0001
48.7870
48.5739
48.3617
36.5945
25.3202
18.5744
12.2451
S39
^{13}C NMR (100 MHz,CD_3OD)
200 190 180 170 160 150 140 130 120 110 100 90 80 70 60 50 40 30 20 10 0
f1 (ppm)

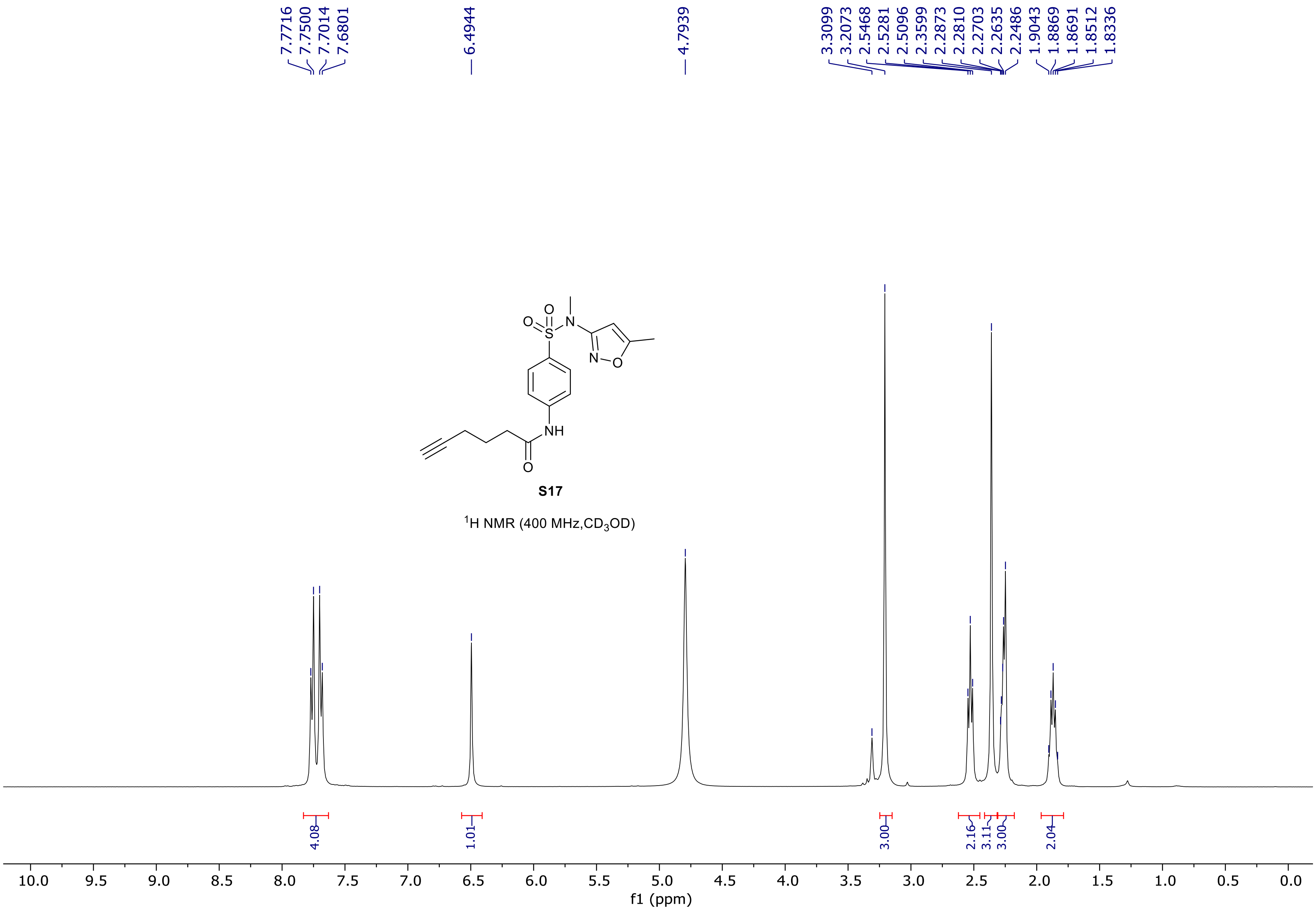
7.7716
7.7500
7.7014
7.6801
6.4944
4.7939
3.3099
3.2073
2.5468
2.5281
2.5096
2.3599
2.2873
2.2810
2.2703
2.2635
2.2486
1.9043
1.8869
1.8691
1.8512
1.8336
S17
^{1}H NMR (400 MHz,CD$_3$OD)
4.08
1.01
3.00
2.16
3.11
3.00
2.04
10.0 9.5 9.0 8.5 8.0 7.5 7.0 6.5 6.0 5.5 5.0 4.5 4.0 3.5 3.0 2.5 2.0 1.5 1.0 0.5 0.0
f1 (ppm)

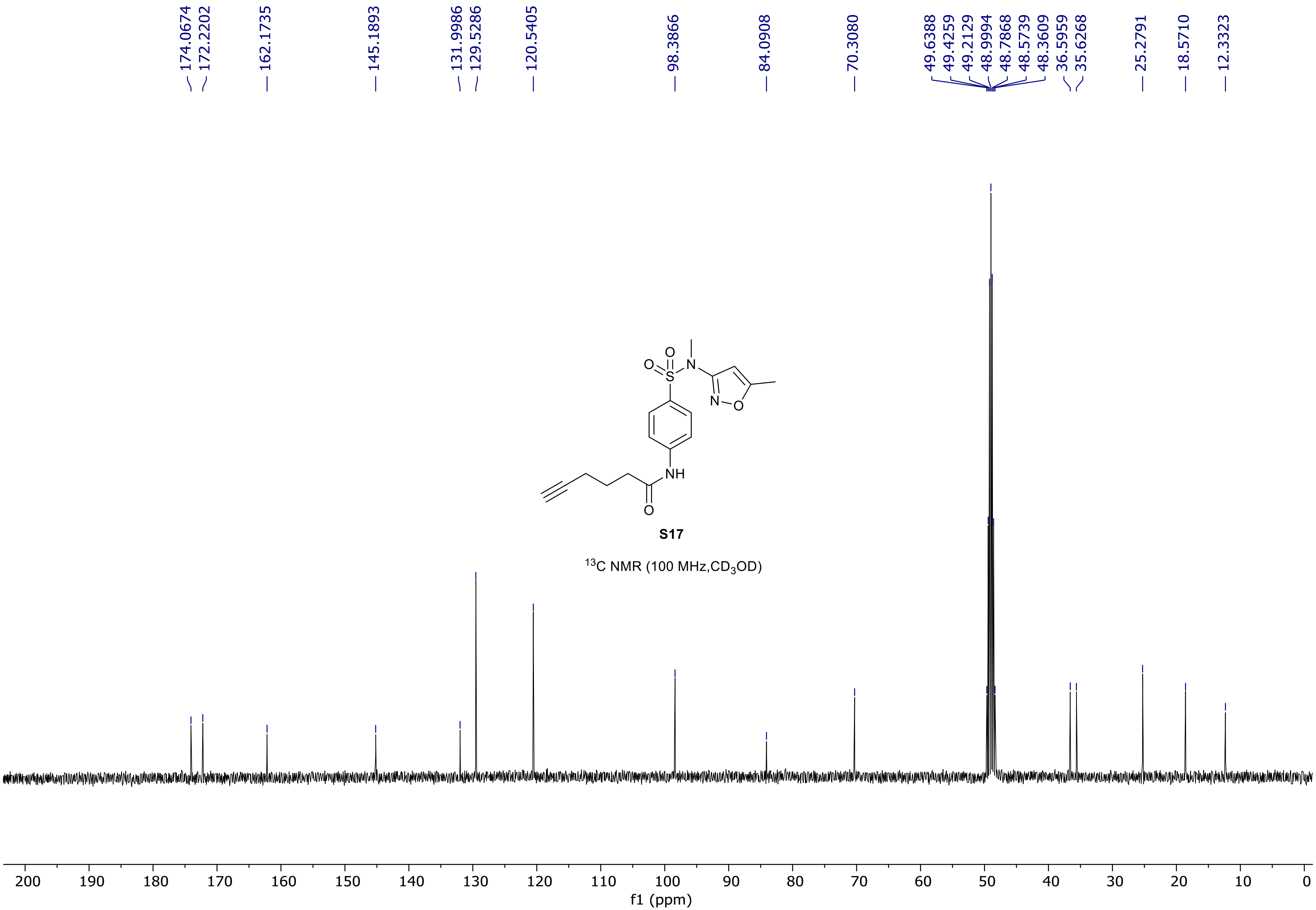
174.0674
172.2202
162.1735
145.1893
131.9986
129.5286
120.5405
98.3866
84.0908
70.3080
49.6388
49.4259
49.2129
48.9994
48.7868
48.5739
48.3609
36.5959
35.6268
25.2791
18.5710
12.3323
S17
^{13}C NMR (100 MHz,CD$_3$OD)
200 190 180 170 160 150 140 130 120 110 100 90 80 70 60 50 40 30 20 10 0
f1 (ppm)

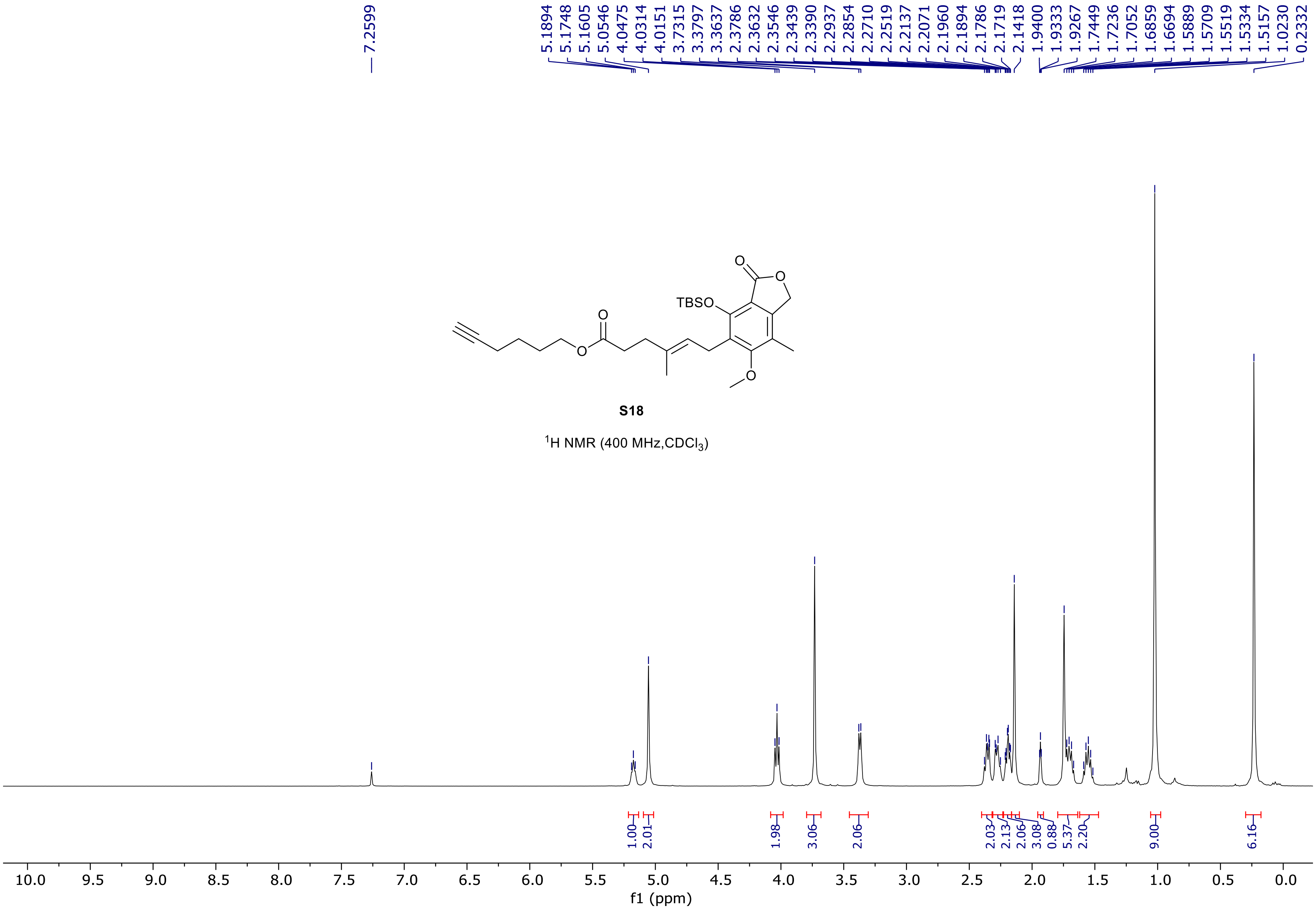
7.2599
5.1894
5.1748
5.1605
5.0546
4.0475
4.0314
4.0151
3.7315
3.3797
3.3637
2.3786
2.3632
2.3546
2.3439
2.3390
2.2937
2.2854
2.2710
2.2519
2.2137
2.2071
2.1960
2.1894
2.1786
2.1719
2.1418
1.9400
1.9333
1.9267
1.7449
1.7236
1.7052
1.6859
1.6694
1.5889
1.5709
1.5519
1.5334
1.5157
1.0230
0.2332
TBSO
O
S18
^{1}H NMR (400 MHz,CDCl$_3$)
1.00
2.01
1.98
3.06
2.06
2.03
2.13
2.06
3.08
0.88
5.37
2.20
9.00
6.16
10.0 9.5 9.0 8.5 8.0 7.5 7.0 6.5 6.0 5.5 5.0 4.5 4.0 3.5 3.0 2.5 2.0 1.5 1.0 0.5 0.0
f1 (ppm)

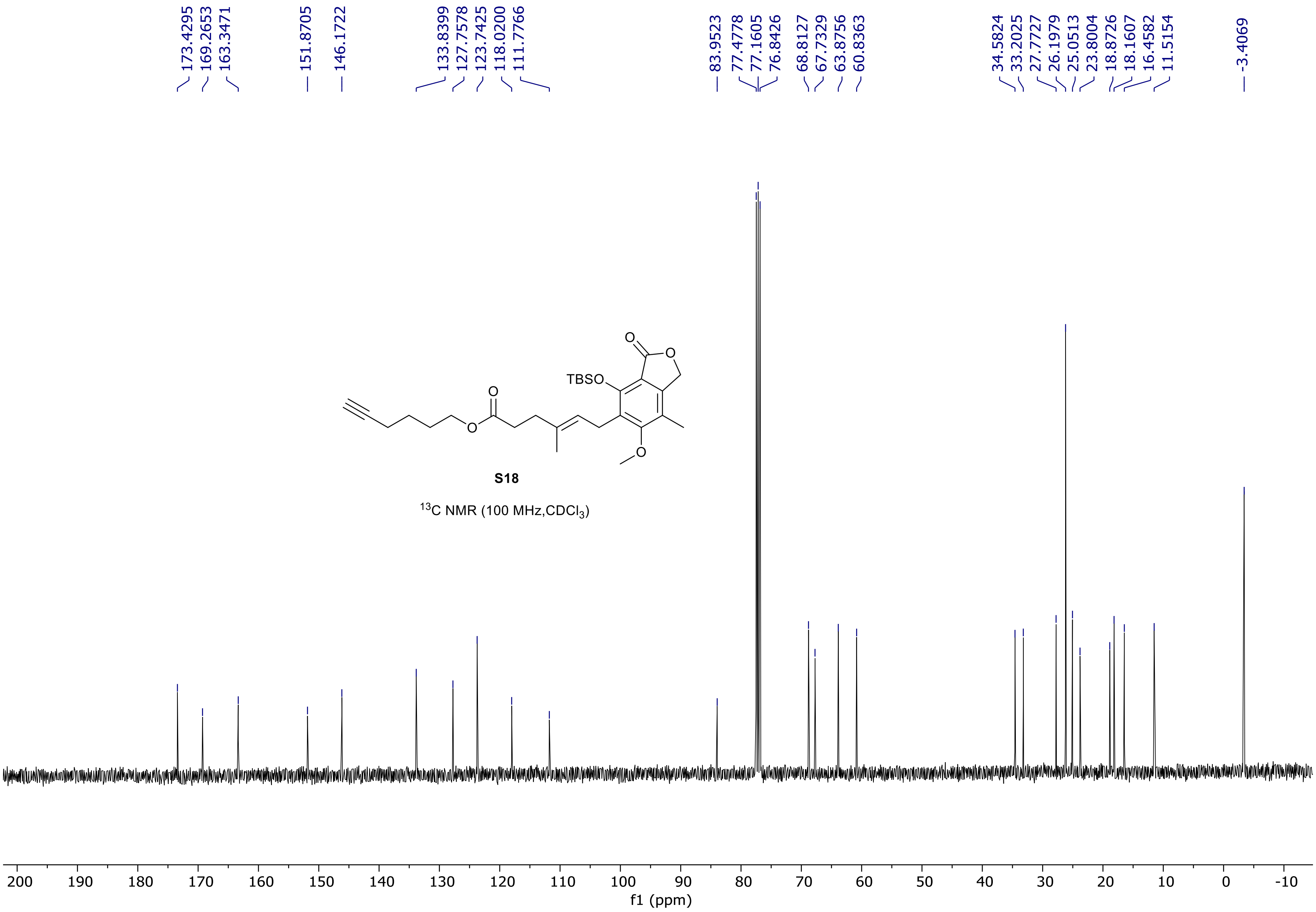
173.4295
169.2653
163.3471
151.8705
146.1722
133.8399
127.7578
123.7425
118.0200
111.7766
83.9523
77.4778
77.1605
76.8426
68.8127
67.7329
63.8756
60.8363
34.5824
33.2025
27.7727
26.1979
25.0513
23.8004
18.8726
18.1607
16.4582
11.5154
-3.4069
TBSO
O
S18
^{13}C NMR (100 MHz,CDCl$_3$)
200 190 180 170 160 150 140 130 120 110 100 90 80 70 60 50 40 30 20 10 0 -10
f1 (ppm)

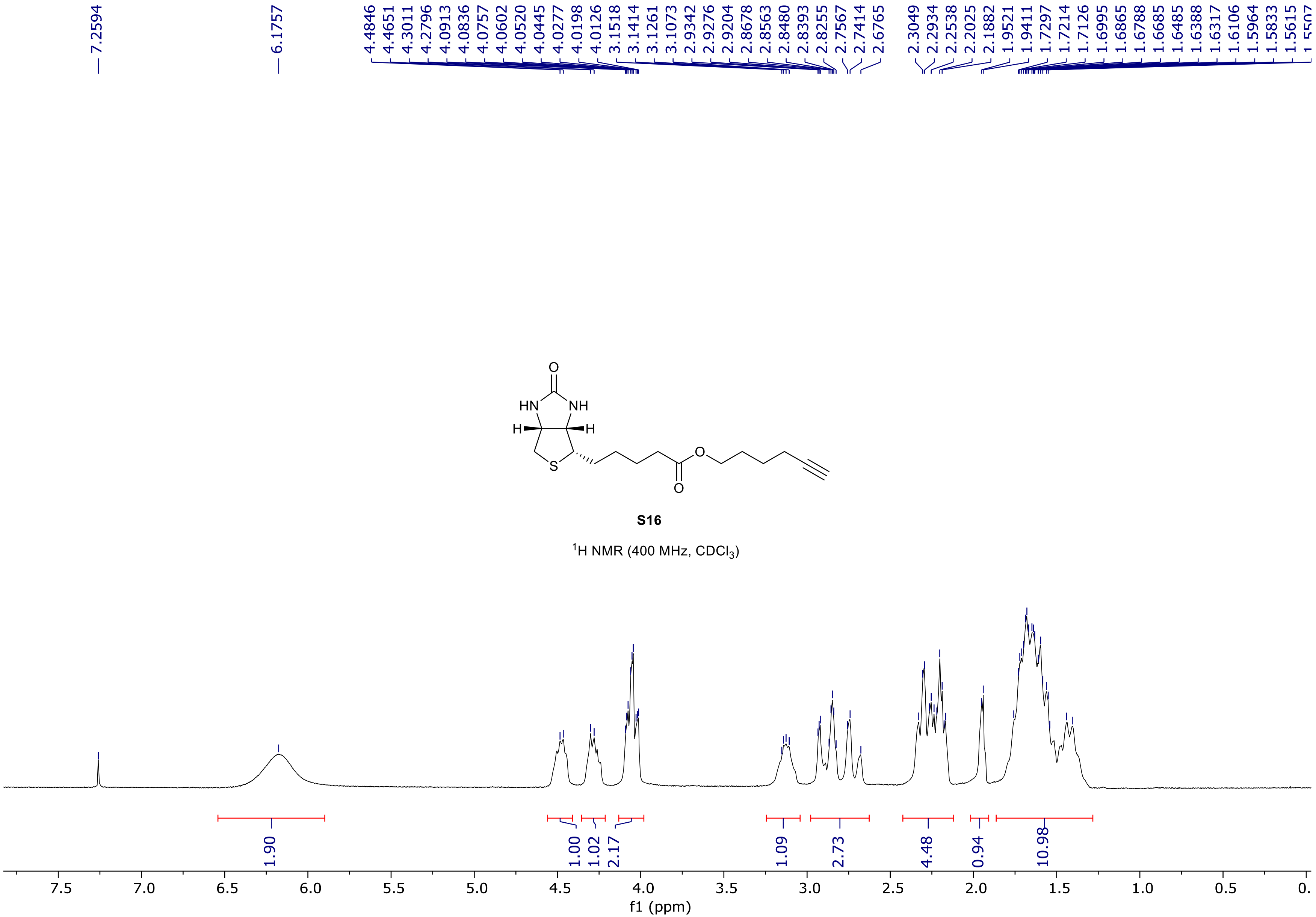

S16

^{1}H NMR (400 MHz, $CDCl_3$)

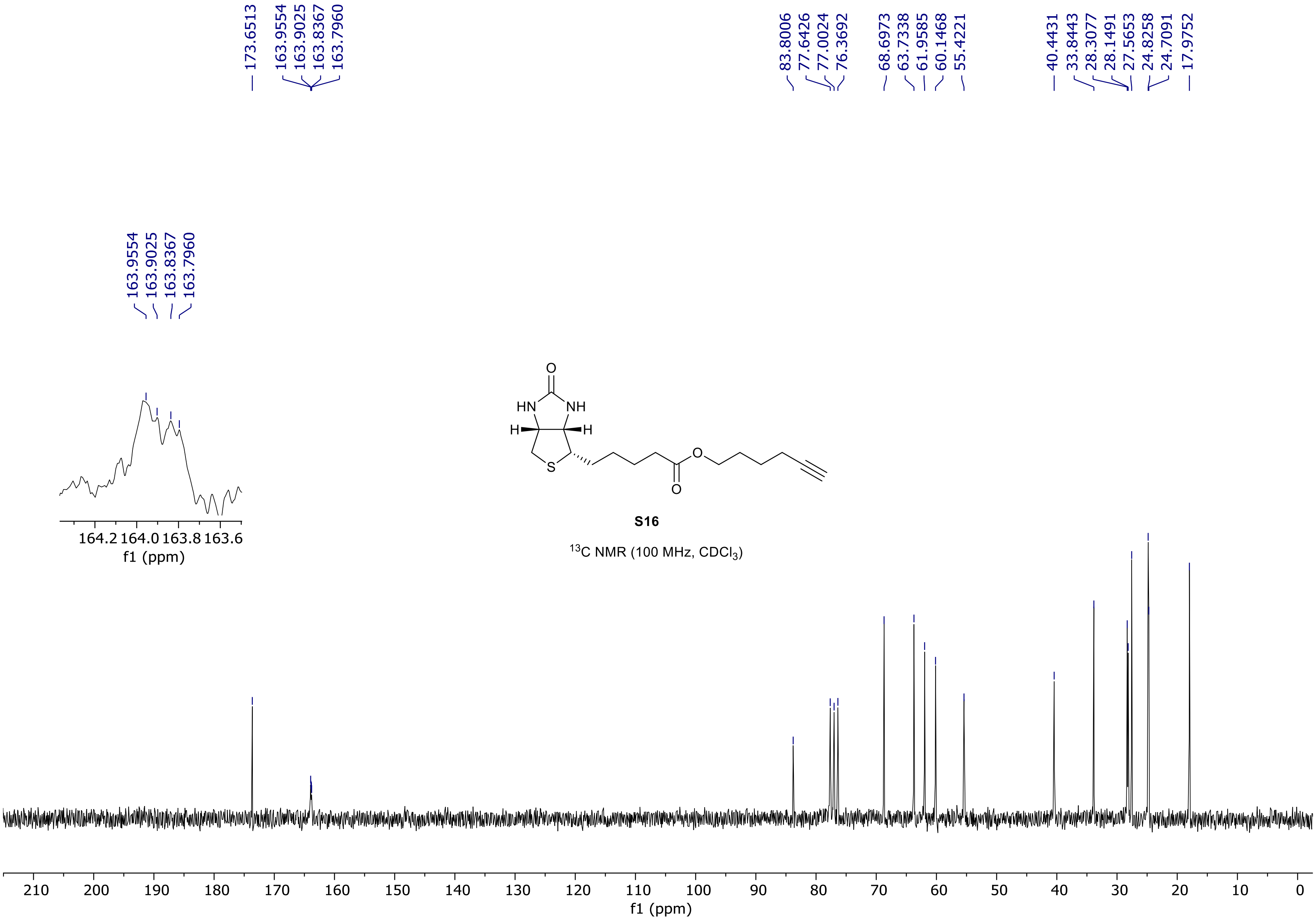

S16

^{13}C NMR (100 MHz, $CDCl_3$)

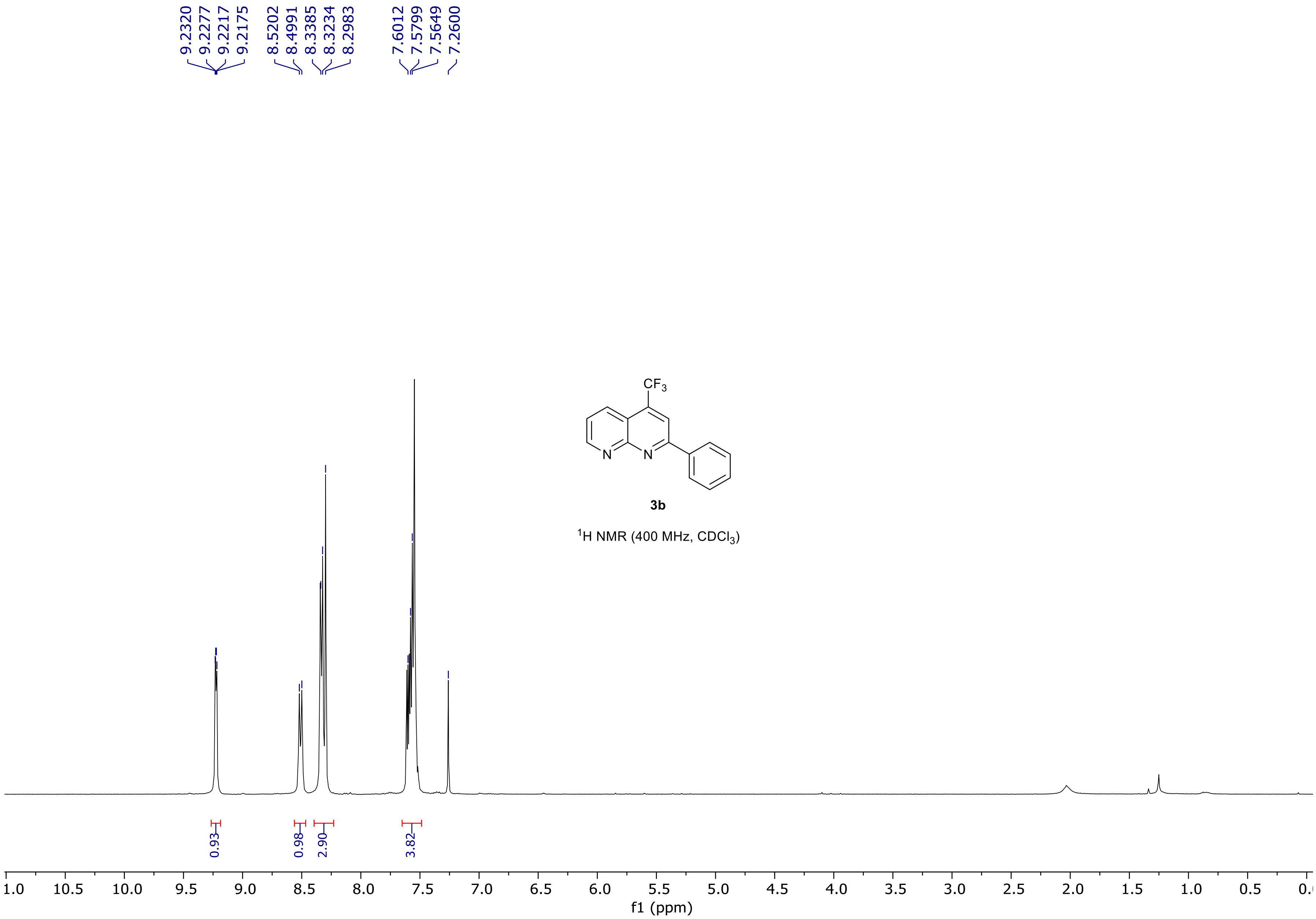
9.2320
9.2277
9.2217
9.2175
8.5202
8.4991
8.3385
8.3234
8.2983
7.6012
7.5799
7.5649
7.2600
CF3
N
N
3b
1H NMR (400 MHz, CDCl3)
0.93
0.98
2.90
3.82
10.5
10.0
9.5
9.0
8.5
8.0
7.5
7.0
6.5
6.0
5.5
5.0
4.5
4.0
3.5
3.0
2.5
2.0
1.5
1.0
0.5
f1 (ppm)

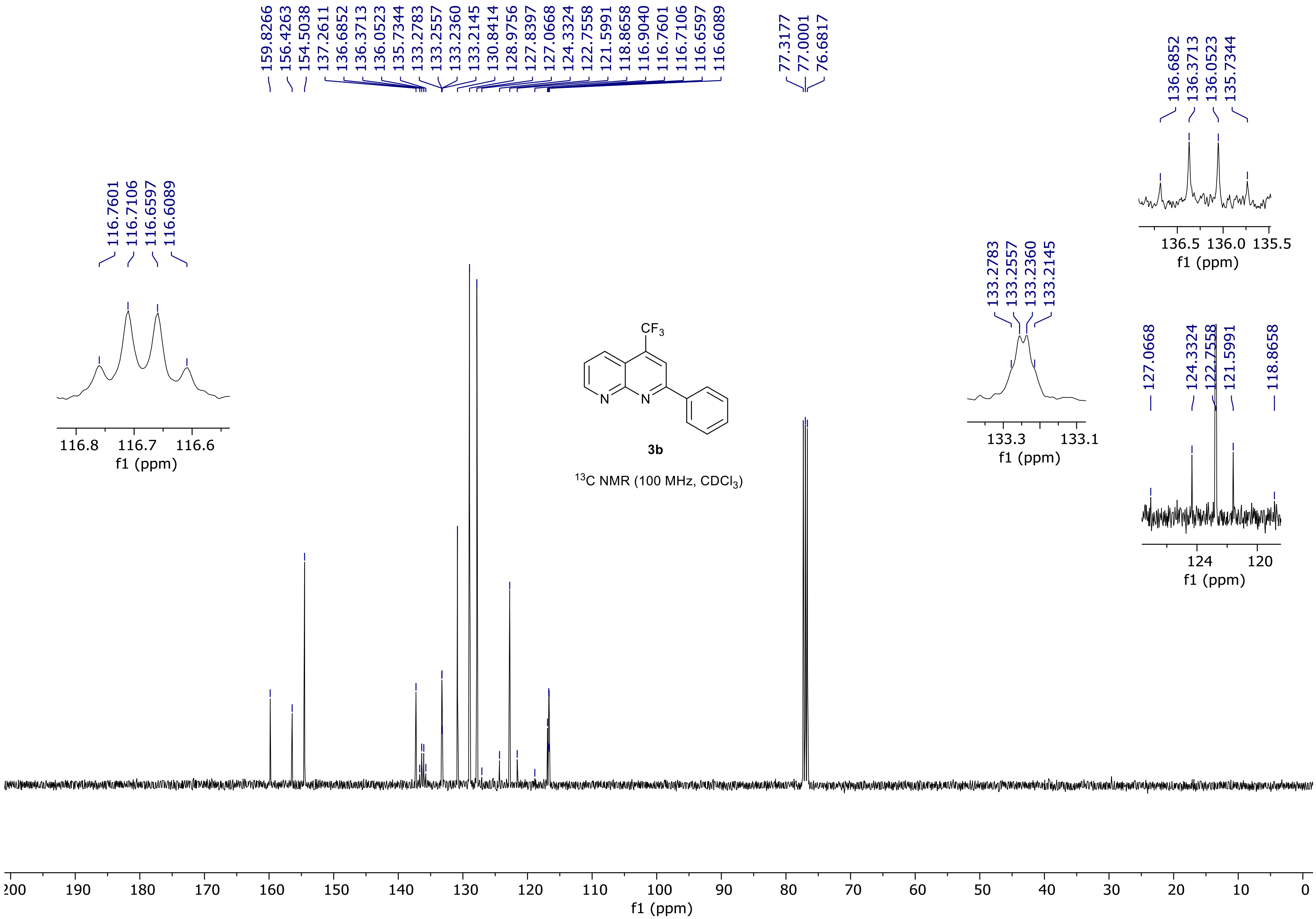
159.8266
156.4263
154.5038
137.2611
136.6852
136.3713
136.0523
135.7344
133.2783
133.2557
133.2360
133.2145
130.8414
128.9756
127.8397
127.0668
124.3324
122.7558
121.5991
118.8658
116.9040
116.7601
116.7106
116.6597
116.6089
77.3177
77.0001
76.6817
CF_3
N
N
3b
^{13}C NMR (100 MHz, $CDCl_3$)
f1 (ppm)

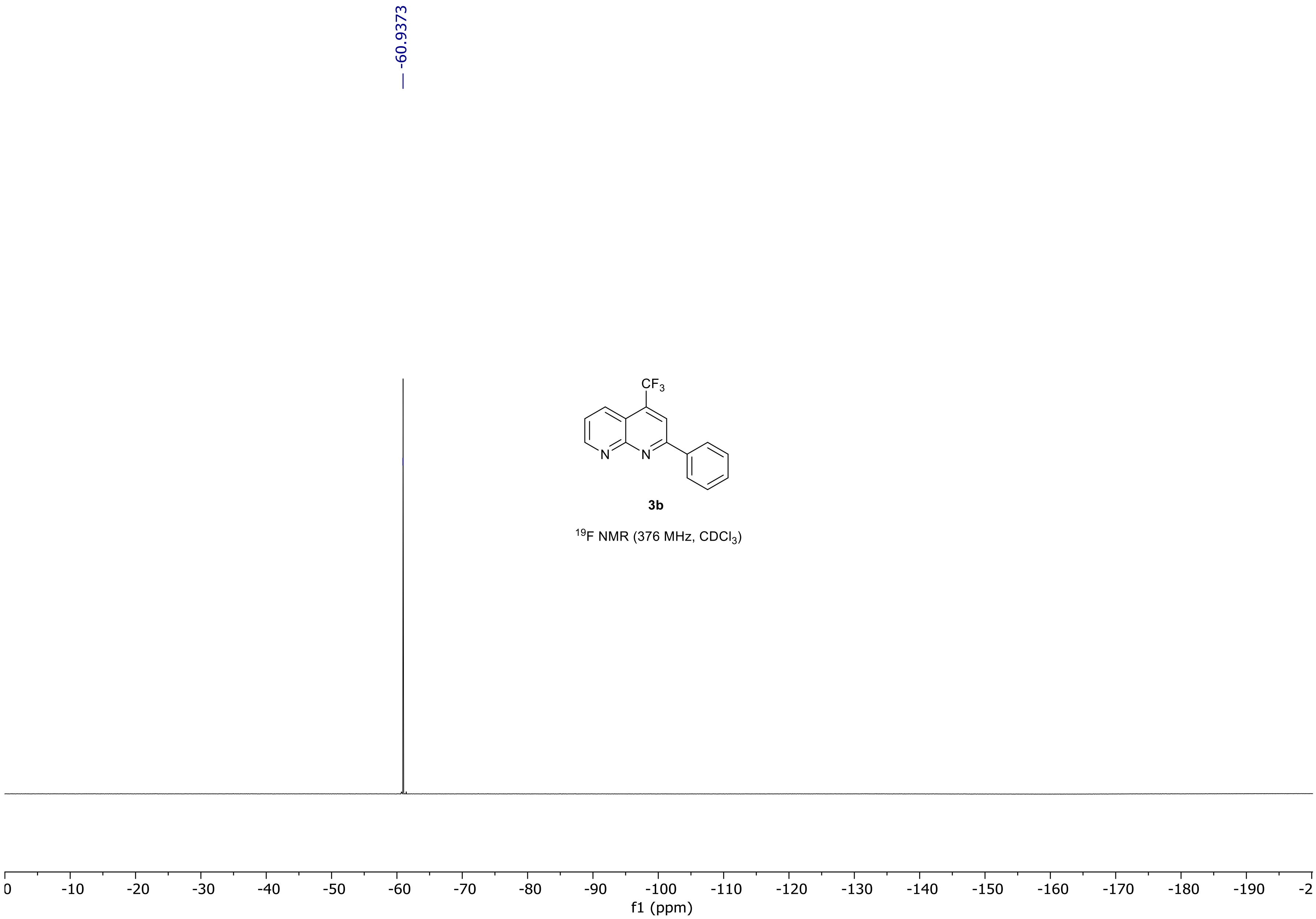
-60.9373
CF3
N
N
3b
19F NMR (376 MHz, CDCl3)
0
-10
-20
-30
-40
-50
-60
-70
-80
-90
-100
-110
-120
-130
-140
-150
-160
-170
-180
-190
-2
f1 (ppm)

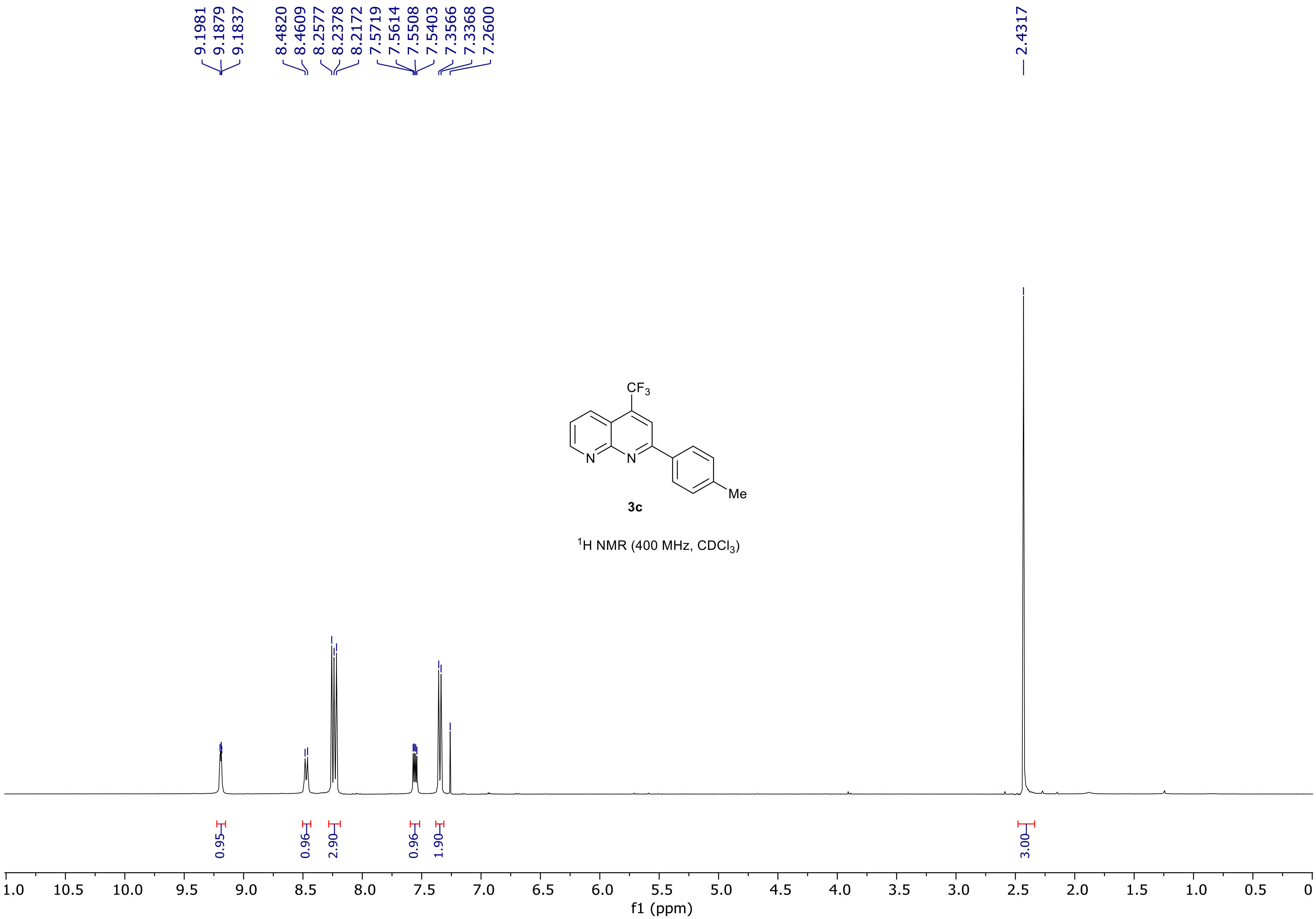
9.1981
9.1879
9.1837
8.4820
8.4609
8.2577
8.2378
8.2172
7.5719
7.5614
7.5508
7.5403
7.3566
7.3368
7.2600
2.4317
CF3
N
N
Me
3c
1H NMR (400 MHz, CDCl3)
0.95
0.96
2.90
0.96
1.90
3.00
1.0
10.5
10.0
9.5
9.0
8.5
8.0
7.5
7.0
6.5
6.0
5.5
5.0
4.5
4.0
3.5
3.0
2.5
2.0
1.5
1.0
0.5
0
f1 (ppm)

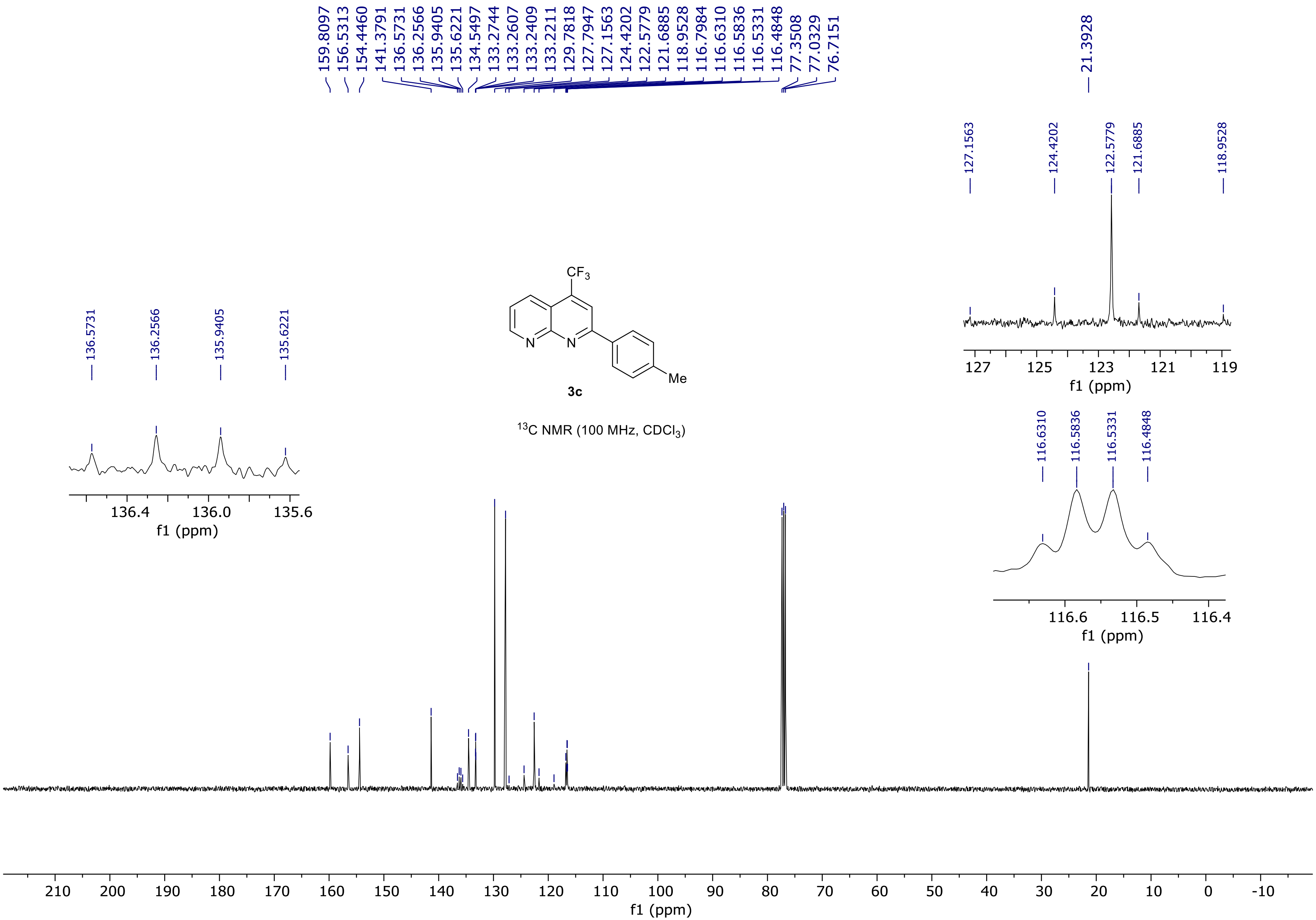

159.8097
156.5313
154.4460
141.3791
136.5731
136.2566
135.9405
135.6221
134.5497
133.2744
133.2607
133.2409
133.2211
129.7818
127.7947
127.1563
124.4202
122.5779
121.6885
118.9528
116.7984
116.6310
116.5836
116.5331
116.4848
77.3508
77.0329
76.7151
21.3928
CF3
N
N
Me
3c
13C NMR (100 MHz, CDCl3)
f1 (ppm)

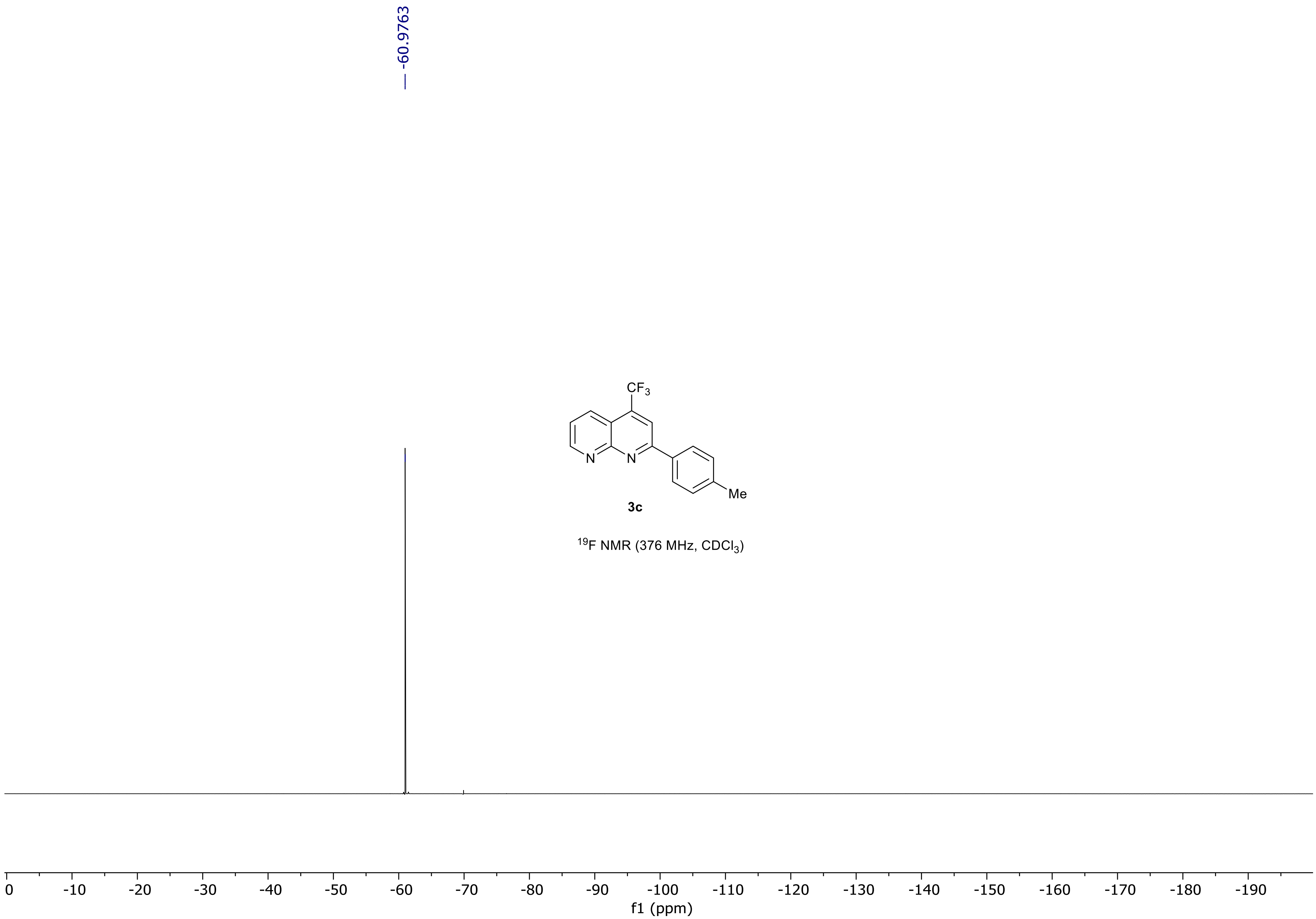
-60.9763
CF3
N
N
Me
3c
19F NMR (376 MHz, CDCl3)
0
-10
-20
-30
-40
-50
-60
-70
-80
-90
-100
-110
-120
-130
-140
-150
-160
-170
-180
-190
f1 (ppm)

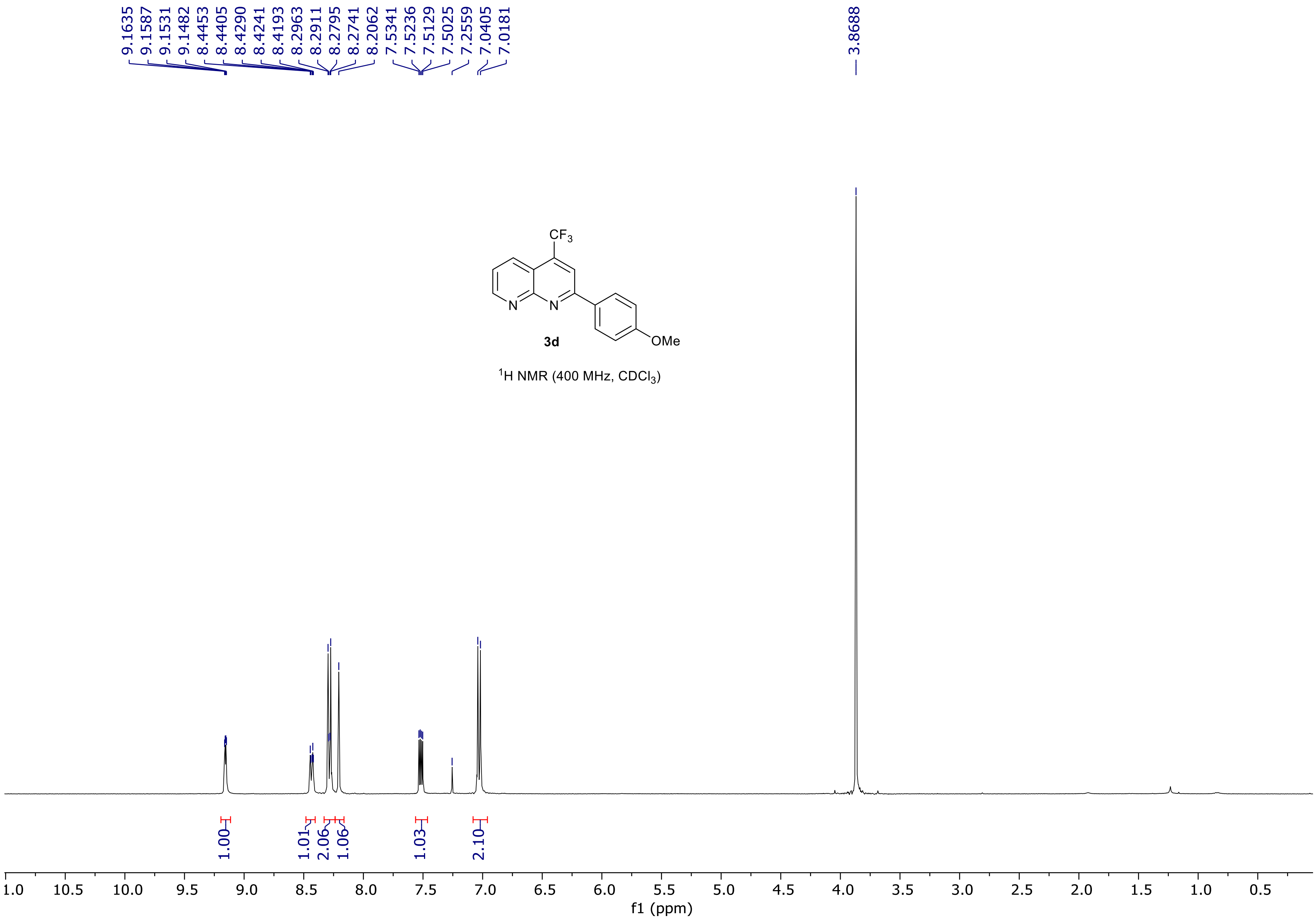
9.1635
9.1587
9.1531
9.1482
8.4453
8.4405
8.4290
8.4241
8.4193
8.2963
8.2911
8.2795
8.2741
8.2062
7.5341
7.5236
7.5129
7.5025
7.2559
7.0405
7.0181
3.8688
CF3
N
N
OMe
3d
1H NMR (400 MHz, CDCl3)
1.00
1.01
2.06
1.06
1.03
2.10
f1 (ppm)

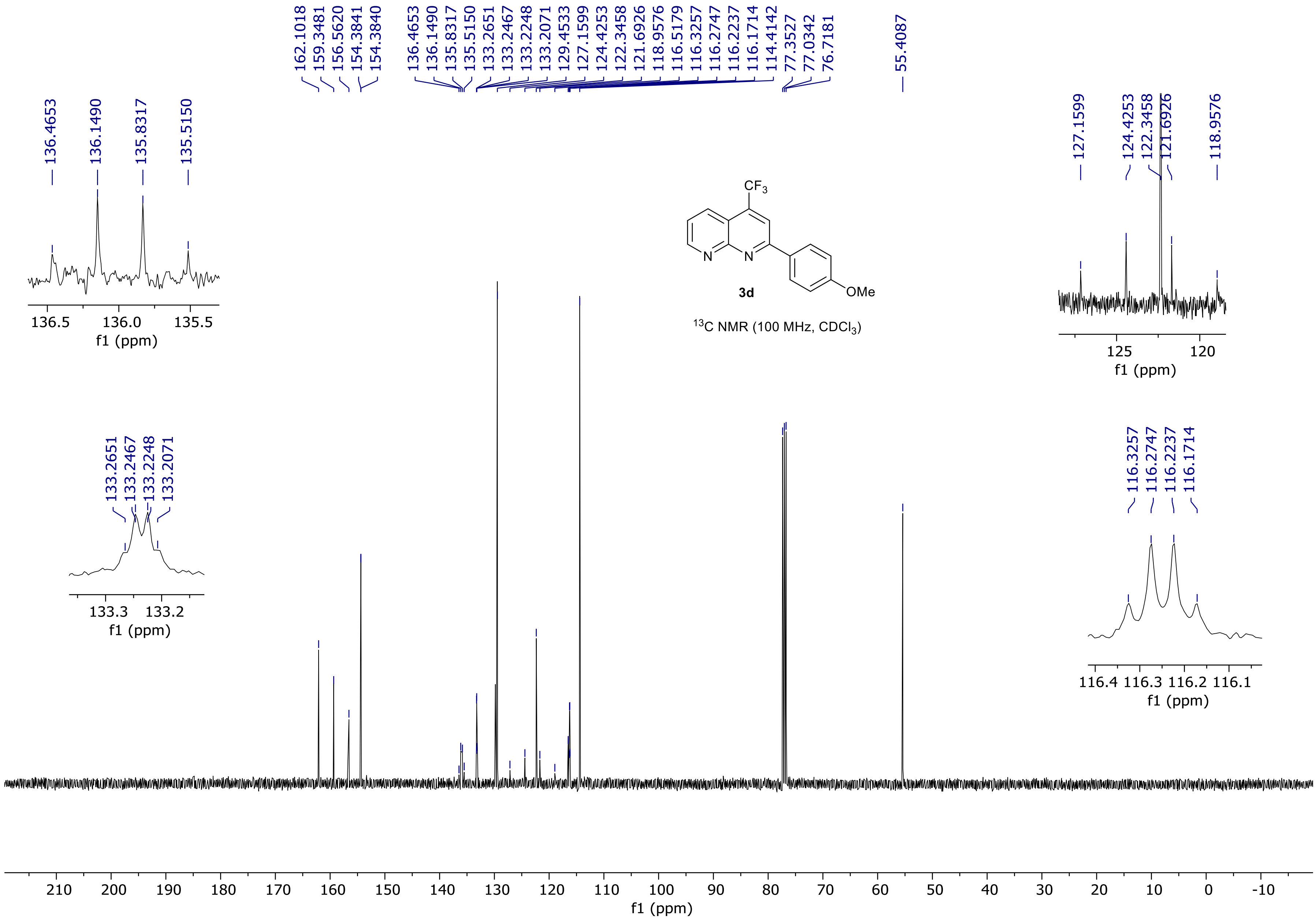
162.1018
159.3481
156.5620
154.3841
154.3840
136.4653
136.1490
135.8317
135.5150
133.2651
133.2467
133.2248
133.2071
129.4533
127.1599
124.4253
122.3458
121.6926
118.9576
116.5179
116.3257
116.2747
116.2237
116.1714
114.4142
77.3527
77.0342
76.7181
55.4087
CF3
N
N
OMe
3d
13C NMR (100 MHz, CDCl3)
f1 (ppm)

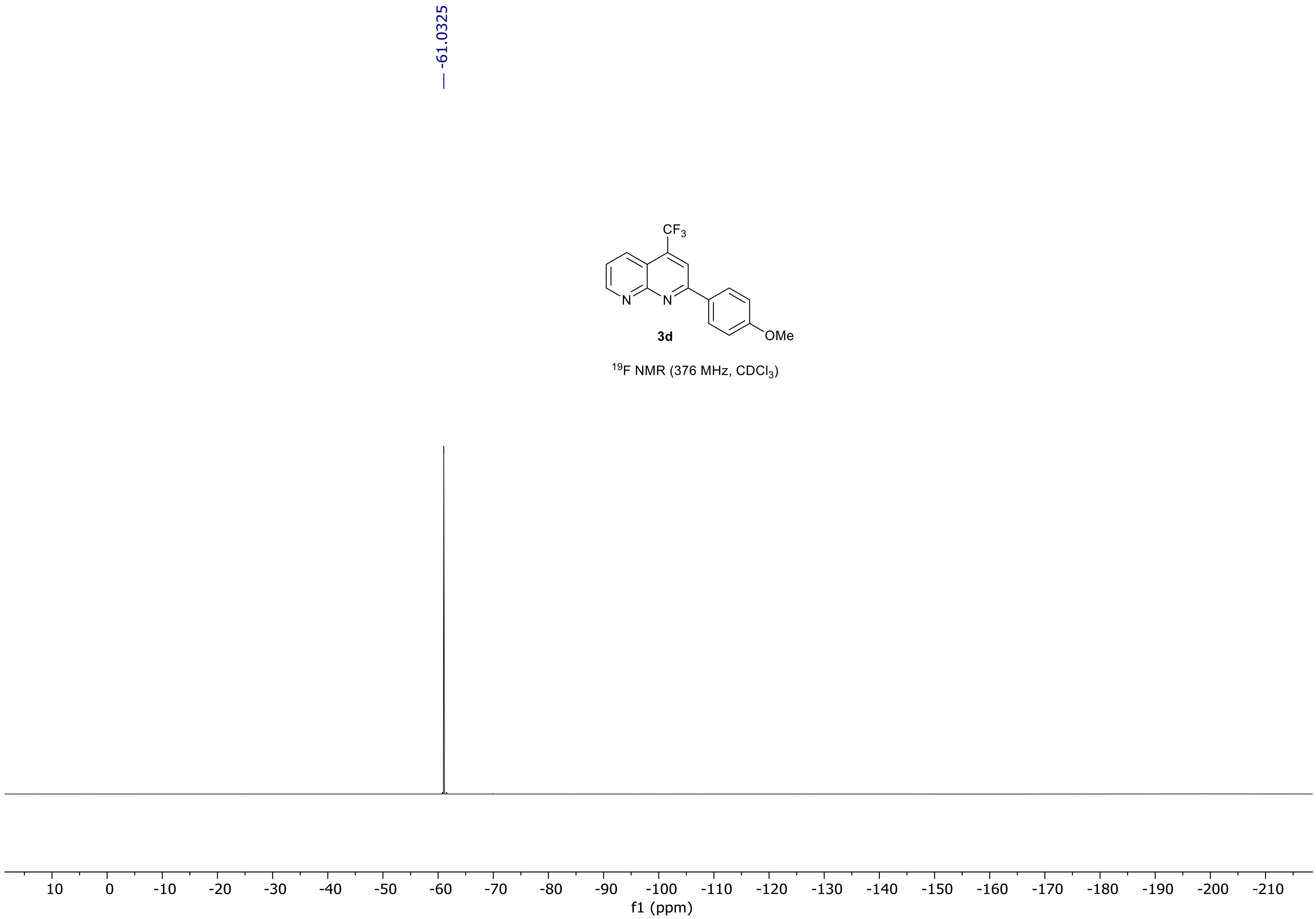
-61.0325
CF3
N
N
OMe
3d
19F NMR (376 MHz, CDCl3)
10 0 -10 -20 -30 -40 -50 -60 -70 -80 -90 -100 -110 -120 -130 -140 -150 -160 -170 -180 -190 -200 -210
f1 (ppm)

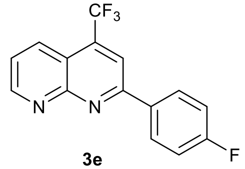

3e

^1H NMR (400 MHz, $CDCl_3$)

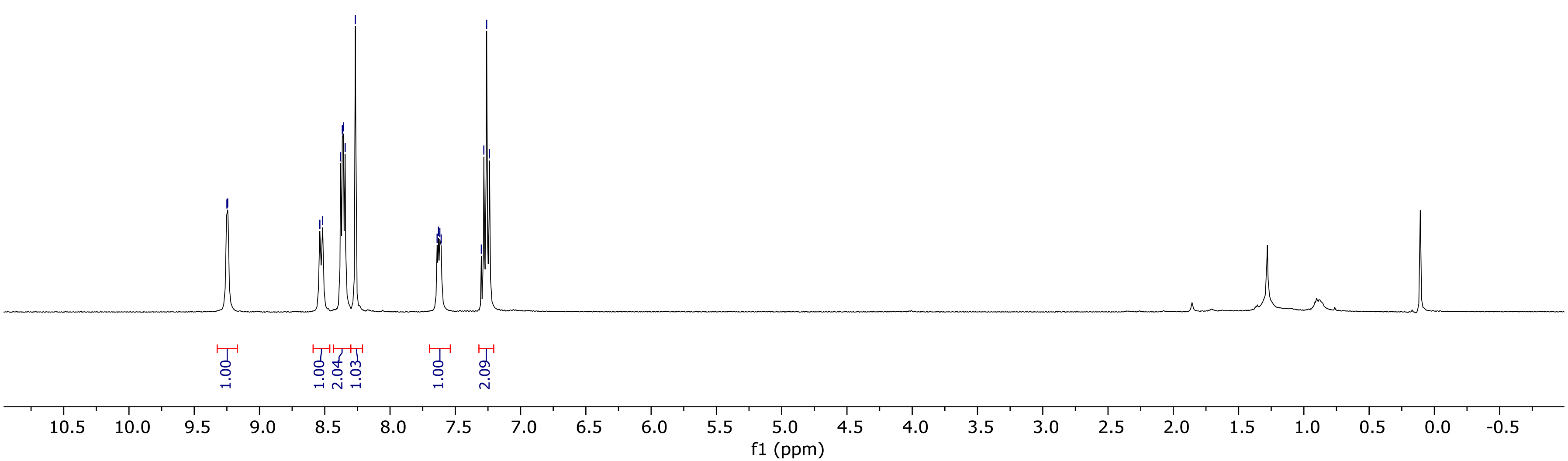

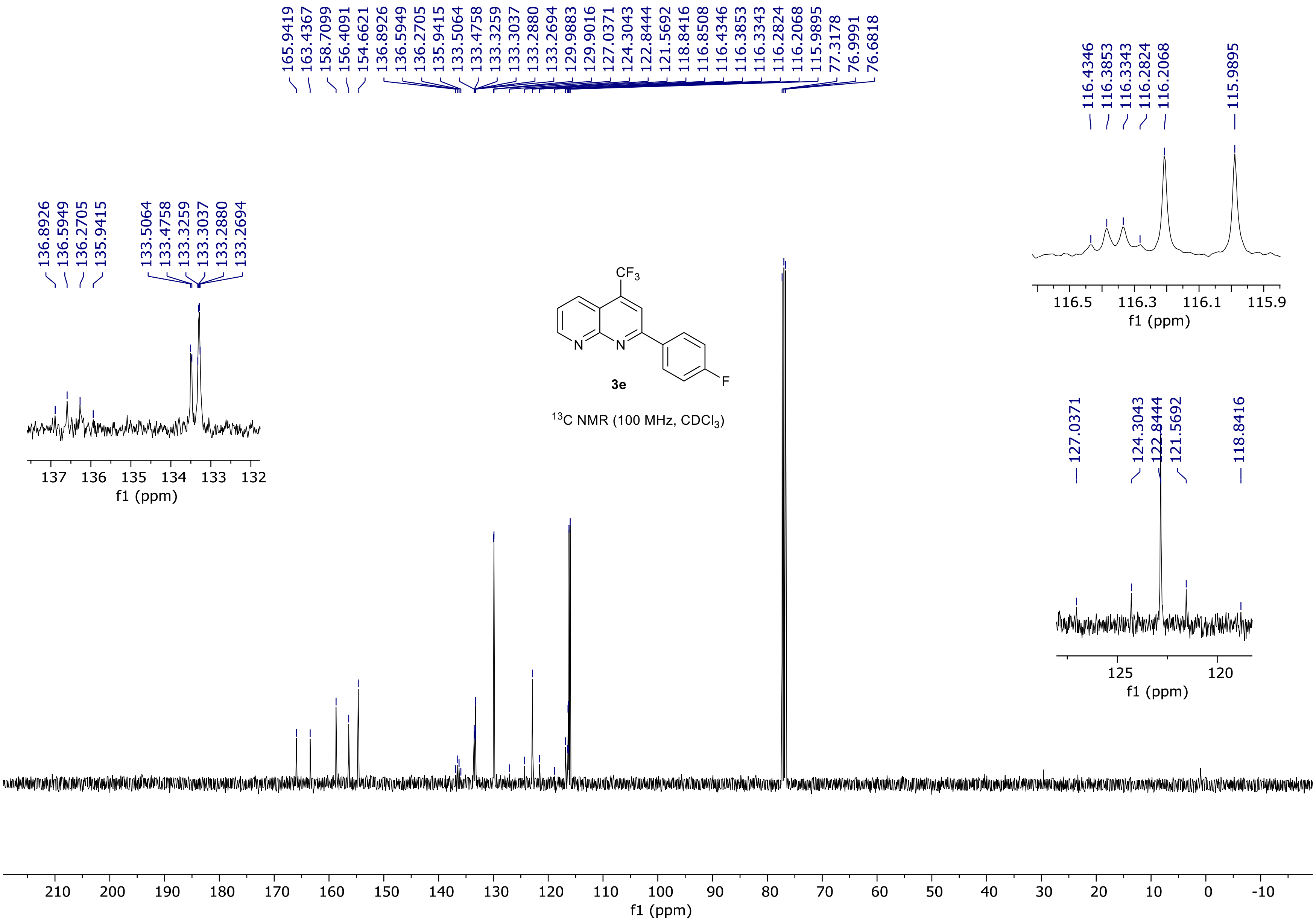
165.9419
163.4367
158.7099
156.4091
154.6621
136.8926
136.5949
136.2705
135.9415
133.5064
133.4758
133.3259
133.3037
133.2880
133.2694
129.9883
129.9016
127.0371
124.3043
122.8444
121.5692
118.8416
116.8508
116.4346
116.3853
116.3343
116.2824
116.2068
115.9895
77.3178
76.9991
76.6818
CF3
N
N
F
3e
13C NMR (100 MHz, CDCl3)
f1 (ppm)

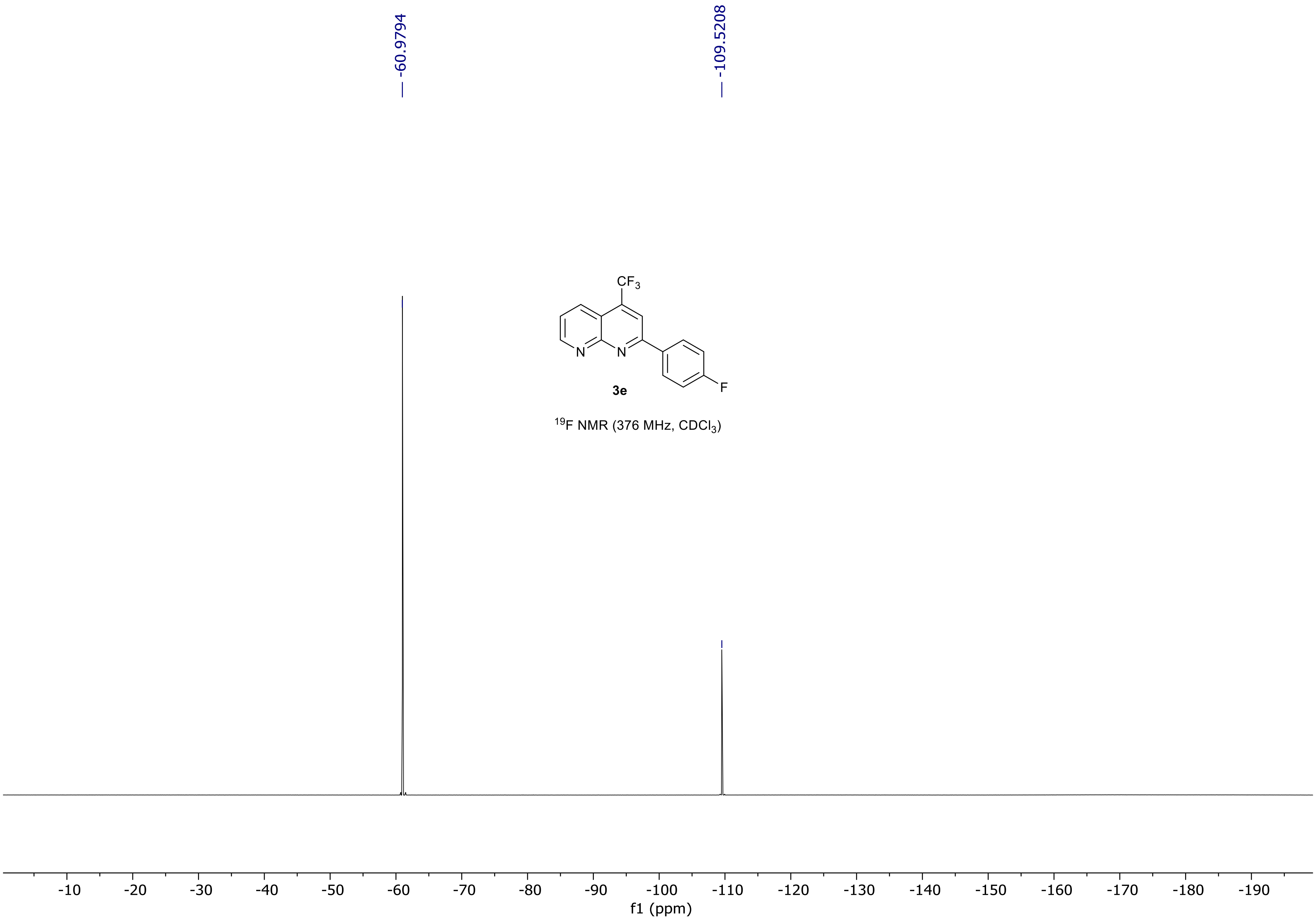
-60.9794
-109.5208
CF3
N
N
F
3e
19F NMR (376 MHz, CDCl3)
-10 -20 -30 -40 -50 -60 -70 -80 -90 -100 -110 -120 -130 -140 -150 -160 -170 -180 -190
f1 (ppm)

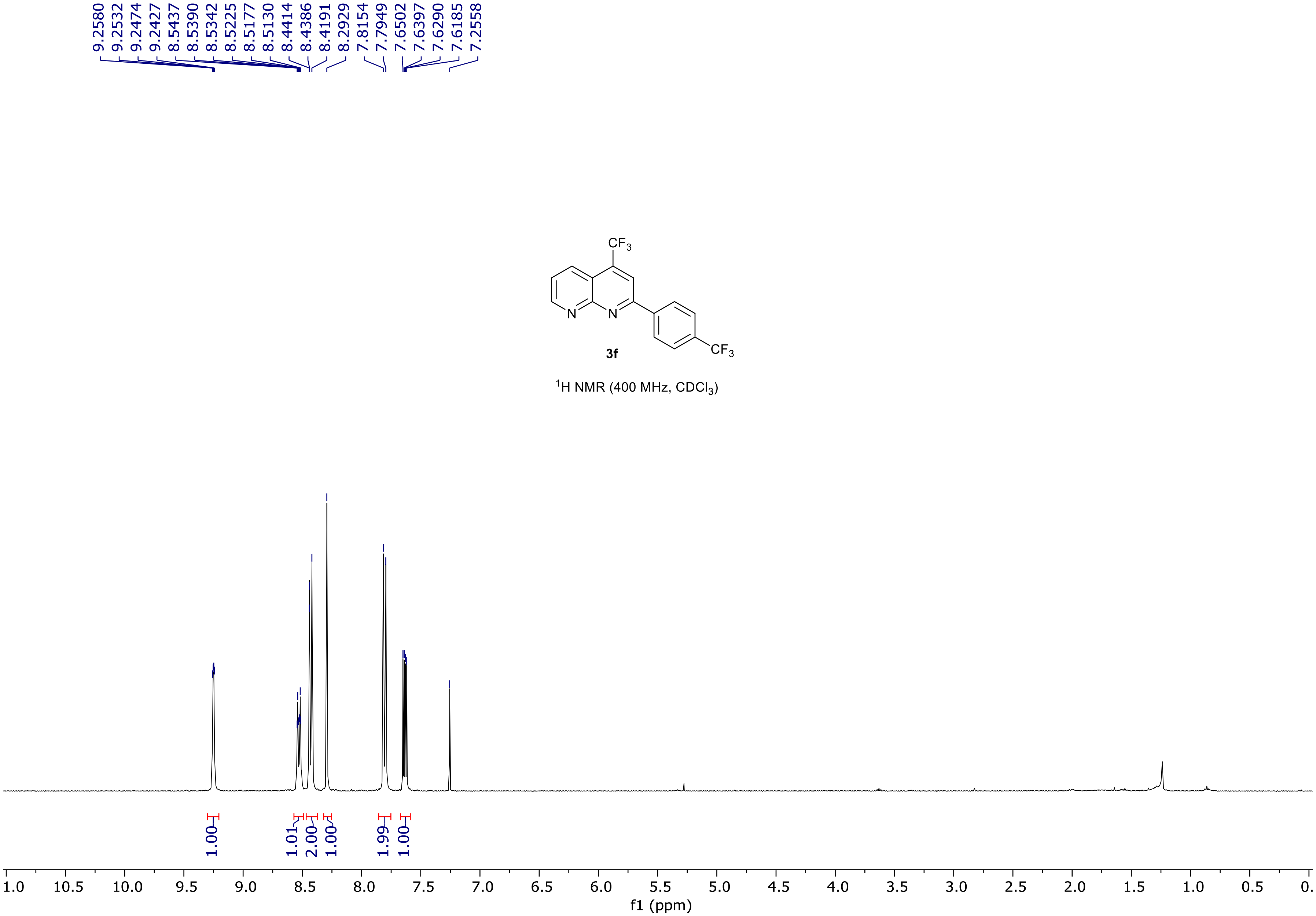

3f

^{1}H NMR (400 MHz, $CDCl_3$)

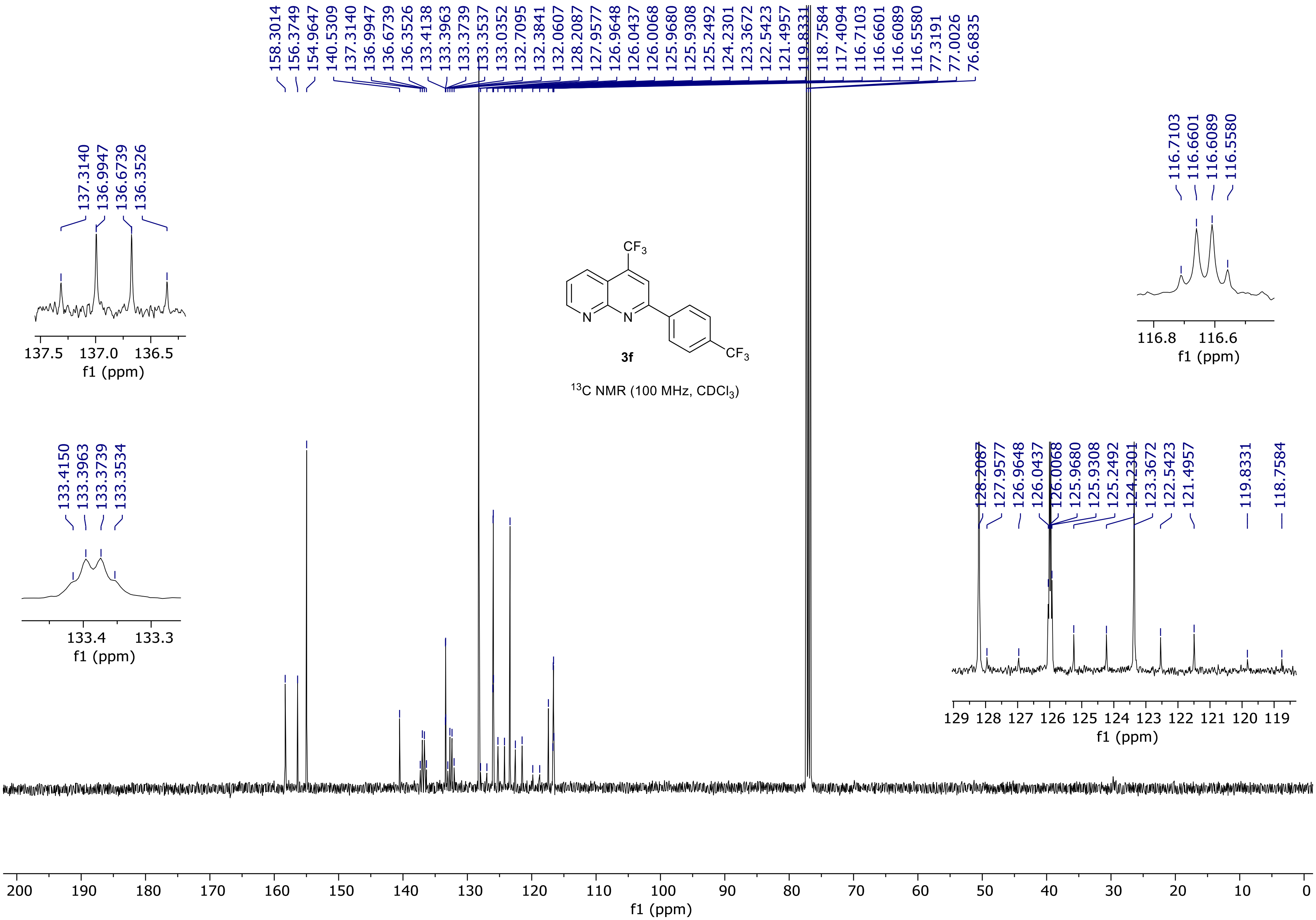
158.3014
156.3749
154.9647
140.5309
137.3140
136.9947
136.6739
136.3526
133.4138
133.3963
133.3739
133.3537
133.0352
132.7095
132.3841
132.0607
128.2087
127.9577
126.9648
126.0437
126.0068
125.9680
125.9308
125.2492
124.2301
123.3672
122.5423
121.4957
119.8331
118.7584
117.4094
116.7103
116.6601
116.6089
116.5580
77.3191
77.0026
76.6835
CF3
N
N
CF3
3f
13C NMR (100 MHz, CDCl3)
137.3140
136.9947
136.6739
136.3526
137.5 137.0 136.5
f1 (ppm)
116.7103
116.6601
116.6089
116.5580
116.8 116.6
f1 (ppm)
133.4150
133.3963
133.3739
133.3534
133.4 133.3
f1 (ppm)
128.2087
127.9577
126.9648
126.0437
126.0068
125.9680
125.9308
125.2492
124.2301
123.3672
122.5423
121.4957
119.8331
118.7584
129 128 127 126 125 124 123 122 121 120 119
f1 (ppm)
200 190 180 170 160 150 140 130 120 110 100 90 80 70 60 50 40 30 20 10 0
f1 (ppm)

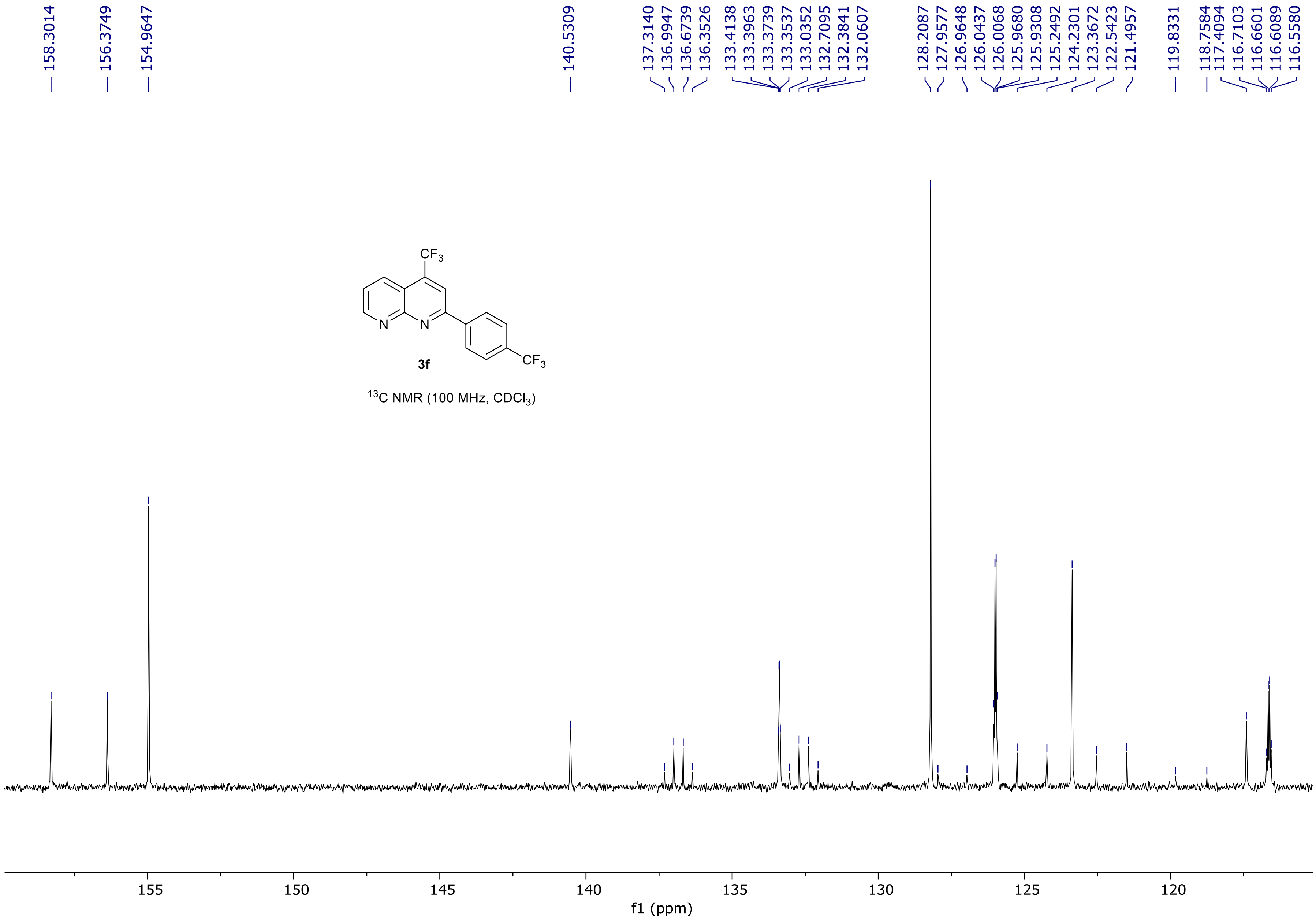
158.3014
156.3749
154.9647
140.5309
137.3140
136.9947
136.6739
136.3526
133.4138
133.3963
133.3739
133.3537
133.0352
132.7095
132.3841
132.0607
128.2087
127.9577
126.9648
126.0437
126.0068
125.9680
125.9308
125.2492
124.2301
123.3672
122.5423
121.4957
119.8331
118.7584
117.4094
116.7103
116.6601
116.6089
116.5580
CF3
N
N
CF3
3f
13C NMR (100 MHz, CDCl3)
155
150
145
140
135
130
125
120
f1 (ppm)

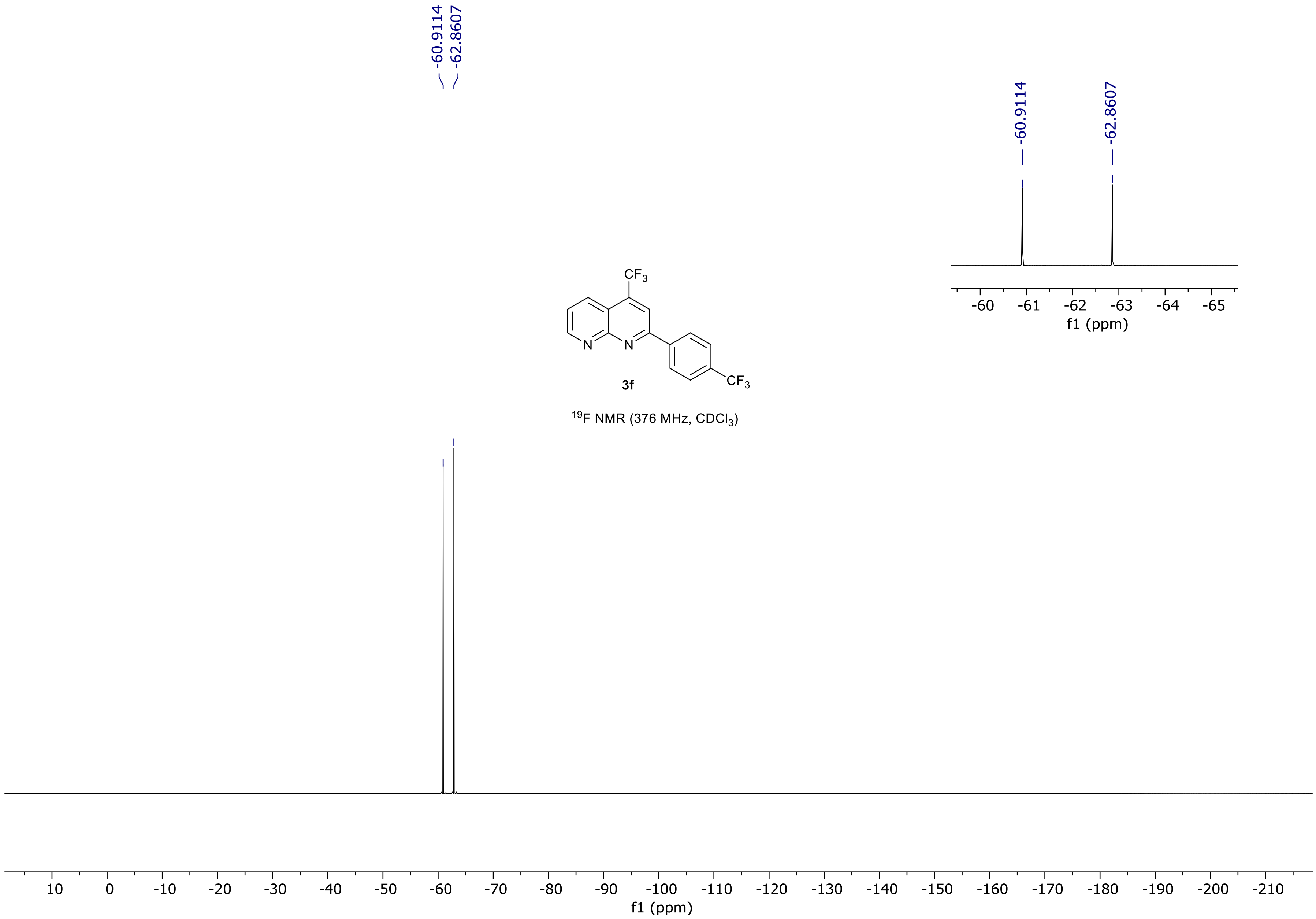
-60.9114
-62.8607
CF3
N
N
CF3
3f
19F NMR (376 MHz, CDCl3)
-60 -61 -62 -63 -64 -65
f1 (ppm)
10 0 -10 -20 -30 -40 -50 -60 -70 -80 -90 -100 -110 -120 -130 -140 -150 -160 -170 -180 -190 -200 -210
f1 (ppm)

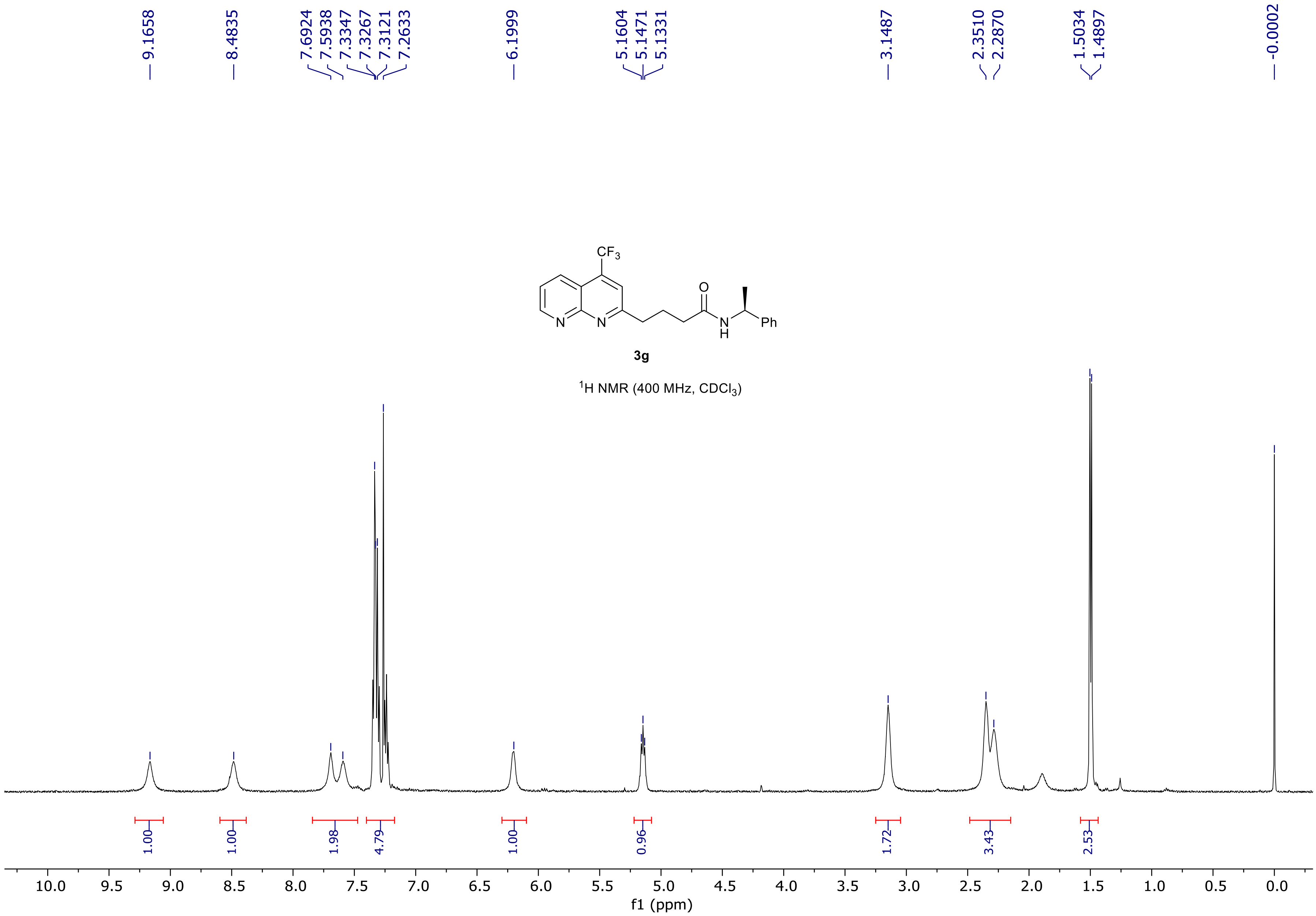

3g

^1H NMR (400 MHz, $CDCl_3$)

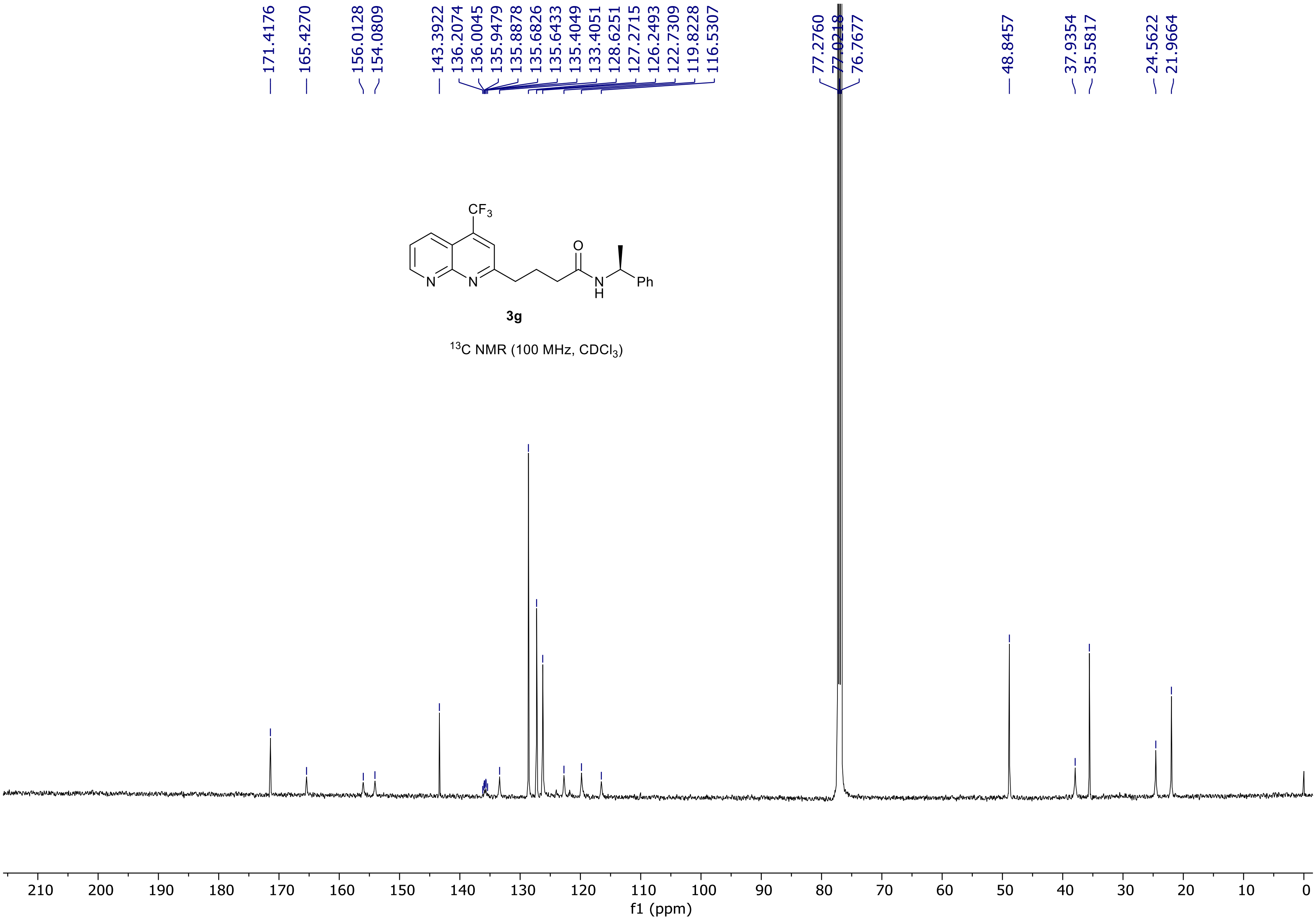
171.4176
165.4270
156.0128
154.0809
143.3922
136.2074
136.0045
135.9479
135.8878
135.6826
135.6433
135.4049
133.4051
128.6251
127.2715
126.2493
122.7309
119.8228
116.5307
77.2760
77.0218
76.7677
48.8457
37.9354
35.5817
24.5622
21.9664
CF3
O
N
N
N
H
Ph
3g
13C NMR (100 MHz, CDCl3)
f1 (ppm)

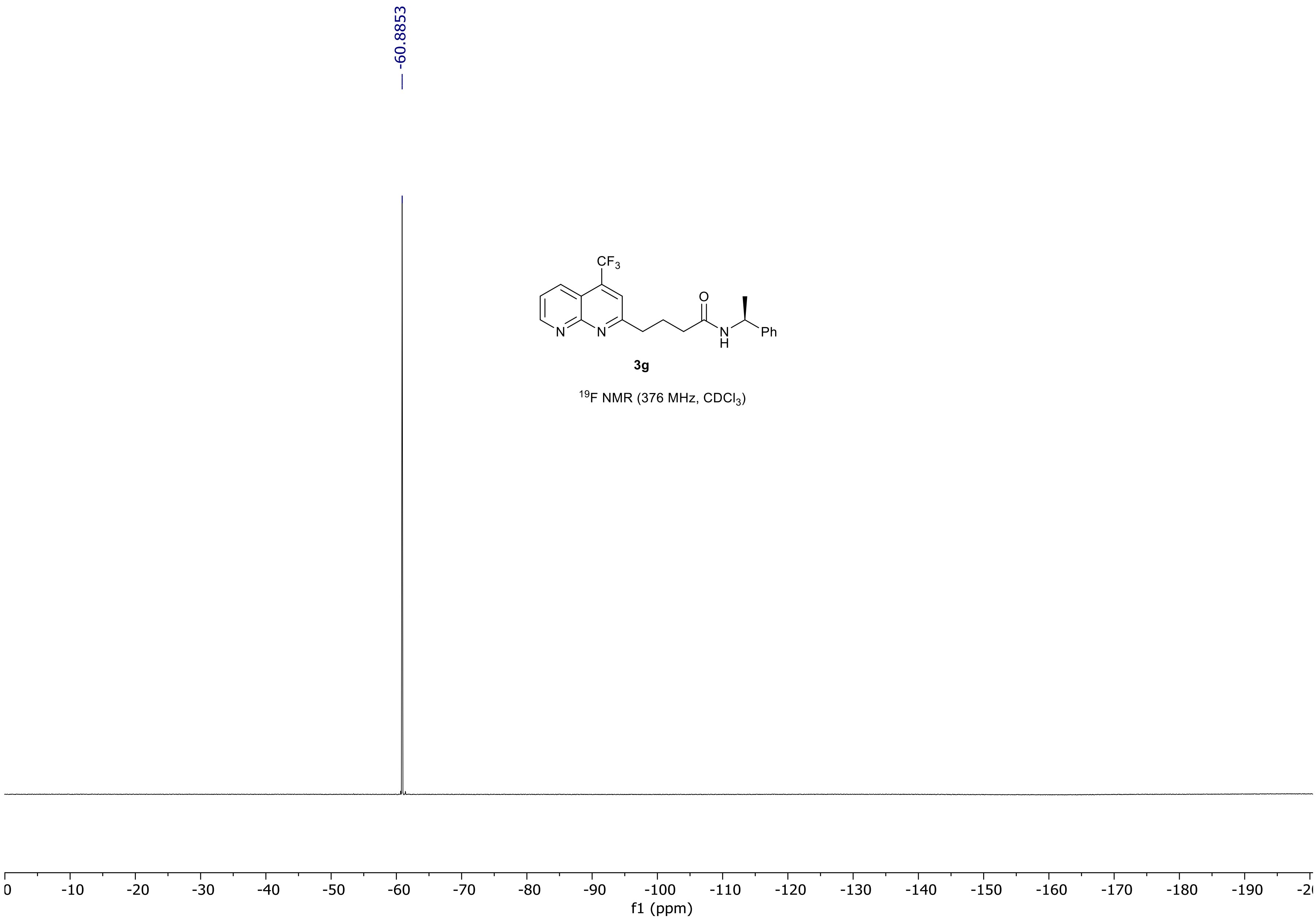
-60.8853
CF3
O
N
N
N
H
Ph
3g
19F NMR (376 MHz, CDCl3)
0 -10 -20 -30 -40 -50 -60 -70 -80 -90 -100 -110 -120 -130 -140 -150 -160 -170 -180 -190
f1 (ppm)

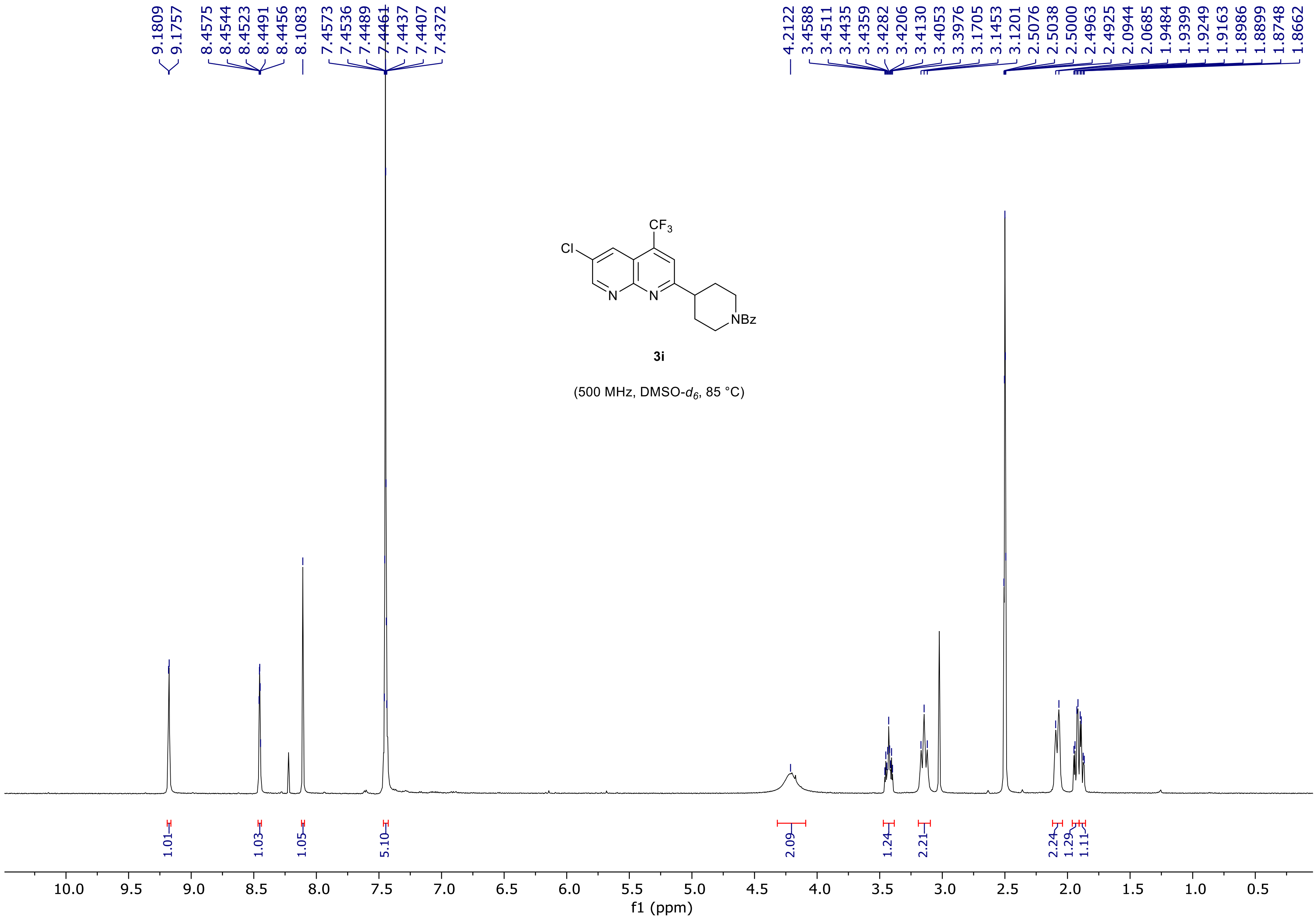
9.1809
9.1757
8.4575
8.4544
8.4523
8.4491
8.4456
8.1083
7.4573
7.4536
7.4489
7.4461
7.4437
7.4407
7.4372
4.2122
3.4588
3.4511
3.4435
3.4359
3.4282
3.4206
3.4130
3.4053
3.3976
3.1705
3.1453
3.1201
2.5076
2.5038
2.5000
2.4963
2.4925
2.0944
2.0685
1.9484
1.9399
1.9249
1.9163
1.8986
1.8899
1.8748
1.8662
CF3
Cl
N
N
NBz
3i
(500 MHz, DMSO-d6, 85 °C)
1.01
1.03
1.05
5.10
2.09
1.24
2.21
2.24
1.29
1.11
10.0
9.5
9.0
8.5
8.0
7.5
7.0
6.5
6.0
5.5
5.0
4.5
4.0
3.5
3.0
2.5
2.0
1.5
1.0
0.5
f1 (ppm)

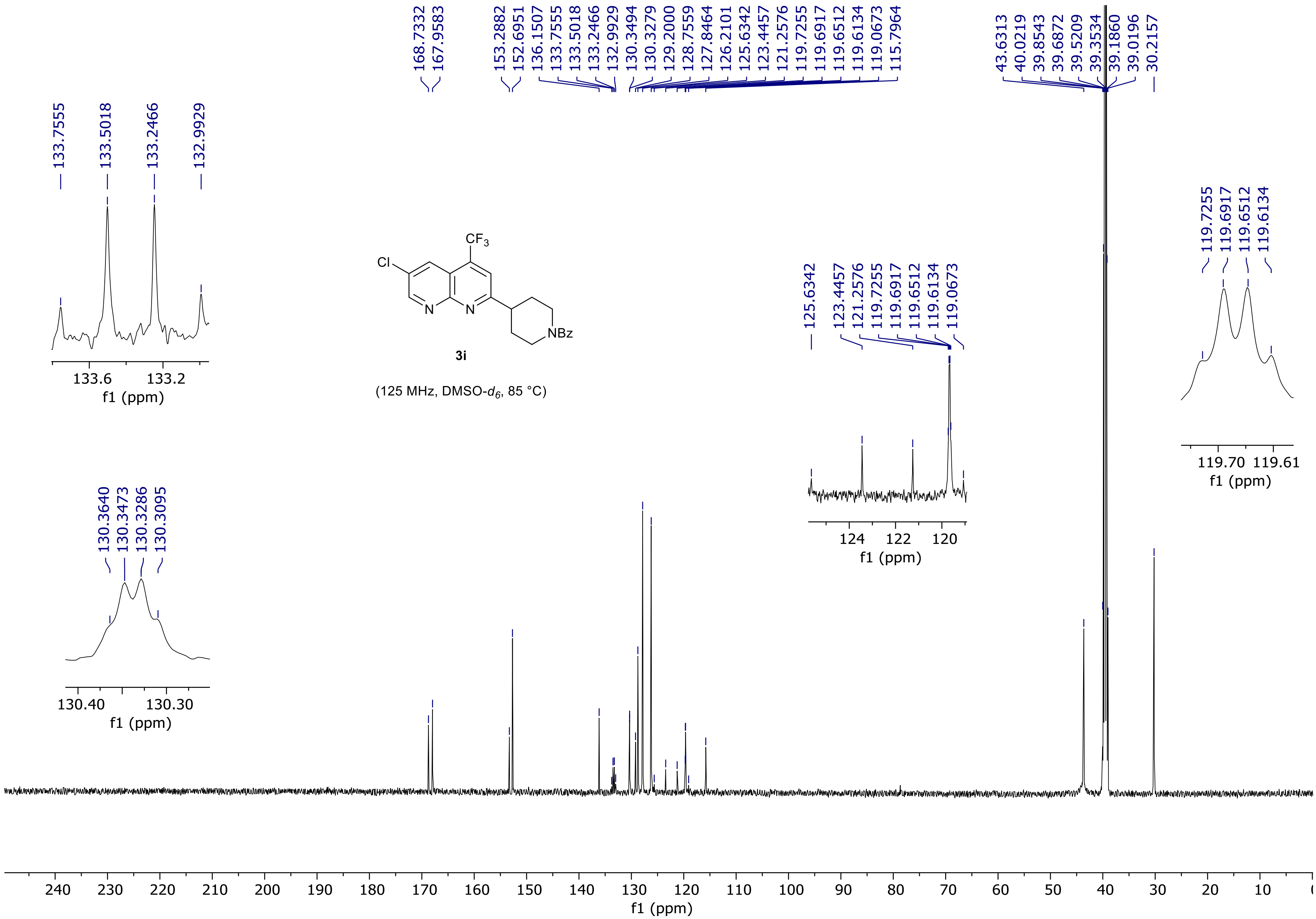
168.7332
167.9583
153.2882
152.6951
136.1507
133.7555
133.5018
133.2466
132.9929
130.3494
130.3279
129.2000
128.7559
127.8464
126.2101
125.6342
123.4457
121.2576
119.7255
119.6917
119.6512
119.6134
119.0673
115.7964
43.6313
40.0219
39.8543
39.6872
39.5209
39.3534
39.1860
39.0196
30.2157
130.3640
130.3473
130.3286
130.3095
CF3
Cl
N
N
NBz
3i
(125 MHz, DMSO-d6, 85 °C)
f1 (ppm)

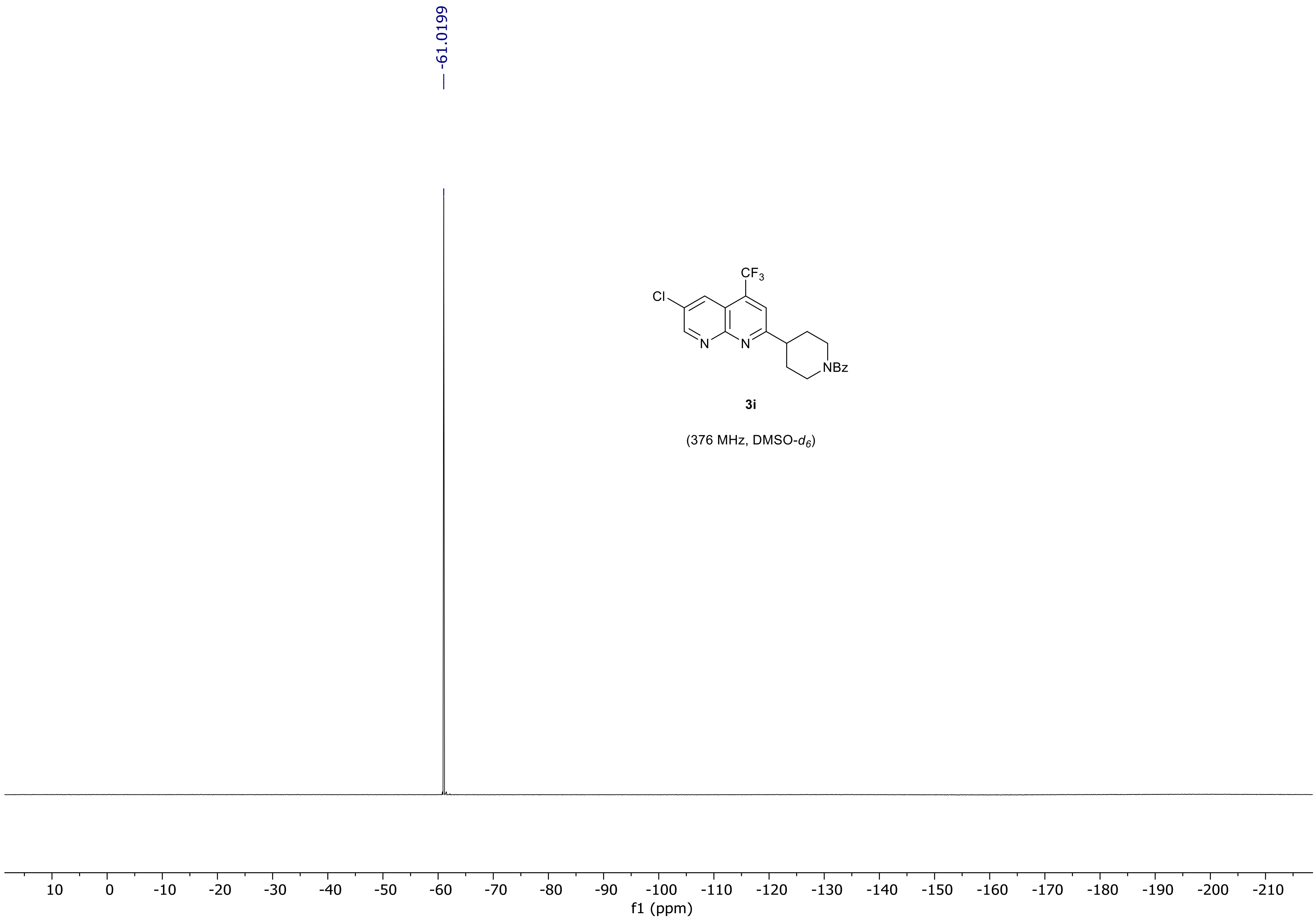

-61.0199
CF3
Cl
N
N
NBz
3i
(376 MHz, DMSO-d6)
10 0 -10 -20 -30 -40 -50 -60 -70 -80 -90 -100 -110 -120 -130 -140 -150 -160 -170 -180 -190 -200 -210
f1 (ppm)

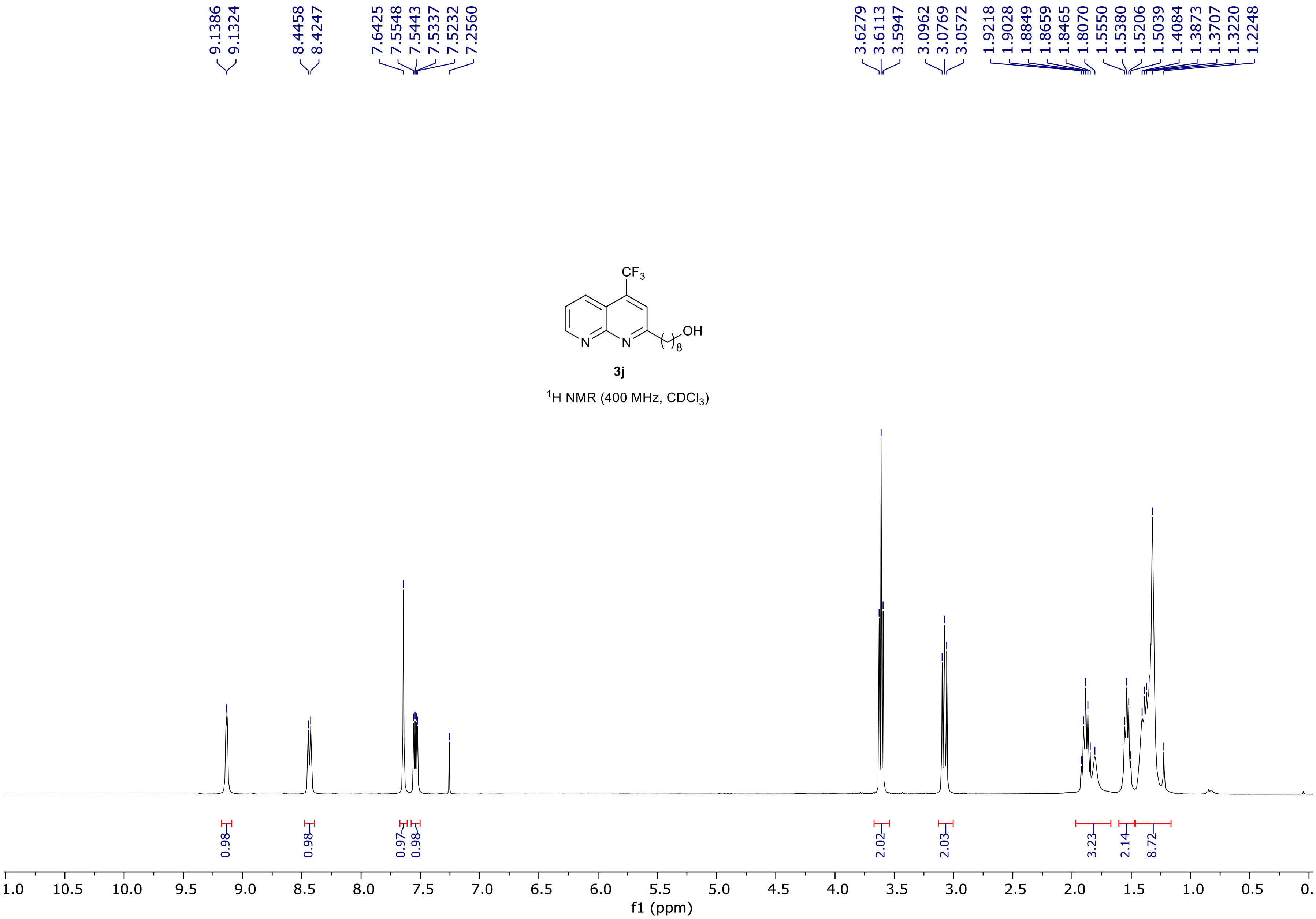
9.1386
9.1324
8.4458
8.4247
7.6425
7.5548
7.5443
7.5337
7.5232
7.2560
3.6279
3.6113
3.5947
3.0962
3.0769
3.0572
1.9218
1.9028
1.8849
1.8659
1.8465
1.8070
1.5550
1.5380
1.5206
1.5039
1.4084
1.3873
1.3707
1.3220
1.2248
CF3
N
N
OH
8
3j
1H NMR (400 MHz, CDCl3)
0.98
0.98
0.97
0.98
2.02
2.03
3.23
2.14
8.72
f1 (ppm)

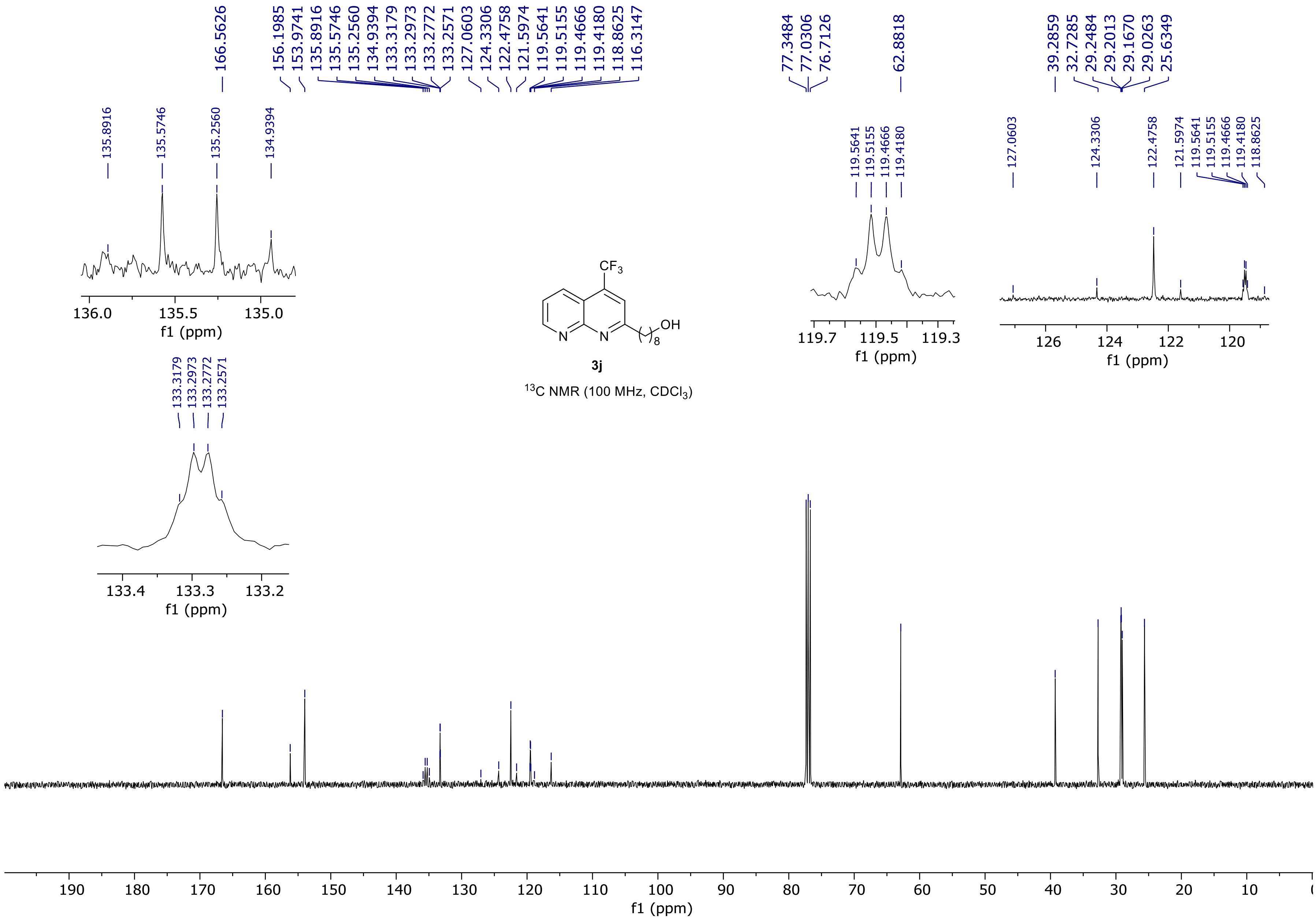

3j

^{13}C NMR (100 MHz, $CDCl_3$)

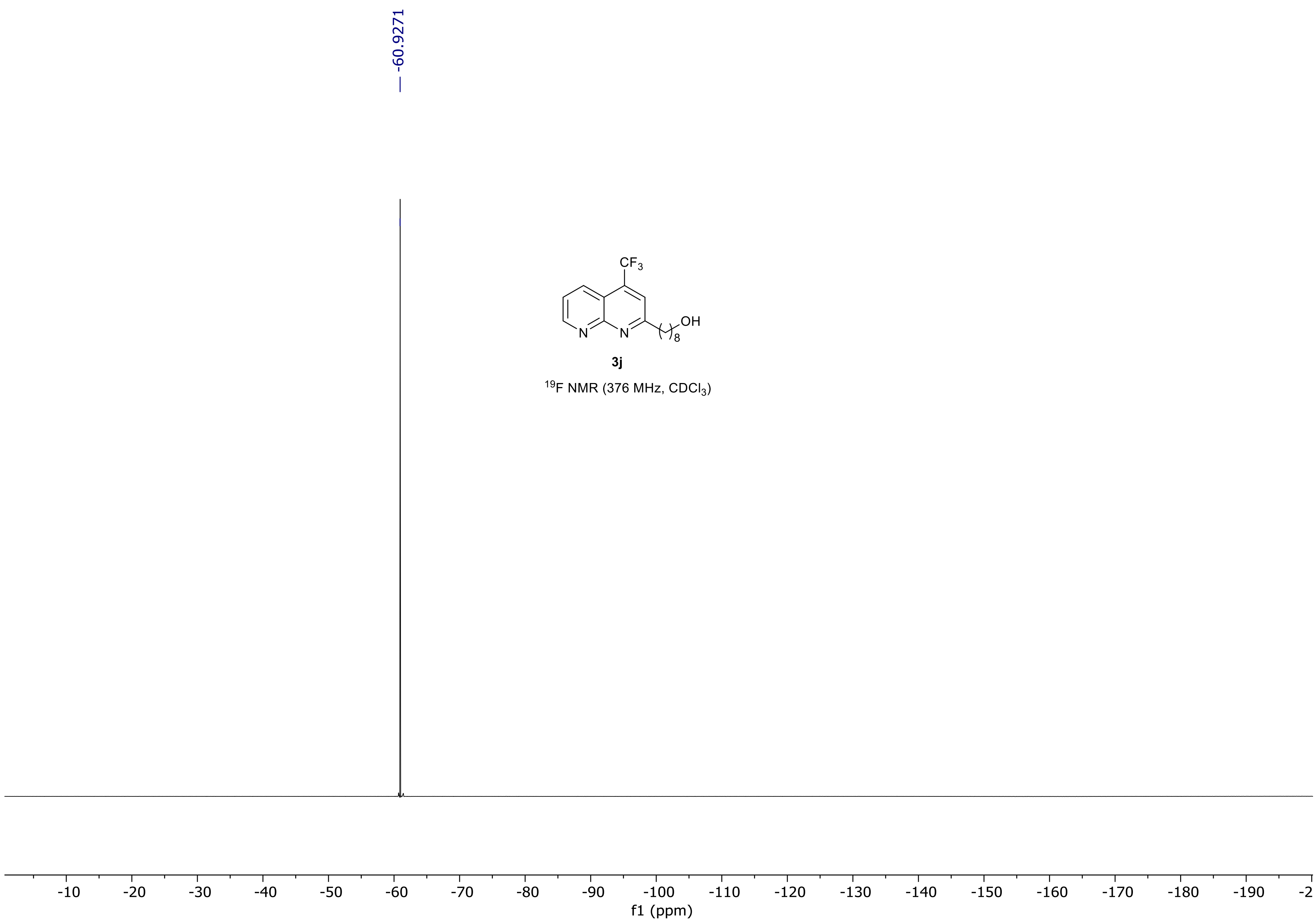
-60.9271
CF3
N
N
OH
8
3j
19F NMR (376 MHz, CDCl3)
-10 -20 -30 -40 -50 -60 -70 -80 -90 -100 -110 -120 -130 -140 -150 -160 -170 -180 -190 -2
f1 (ppm)

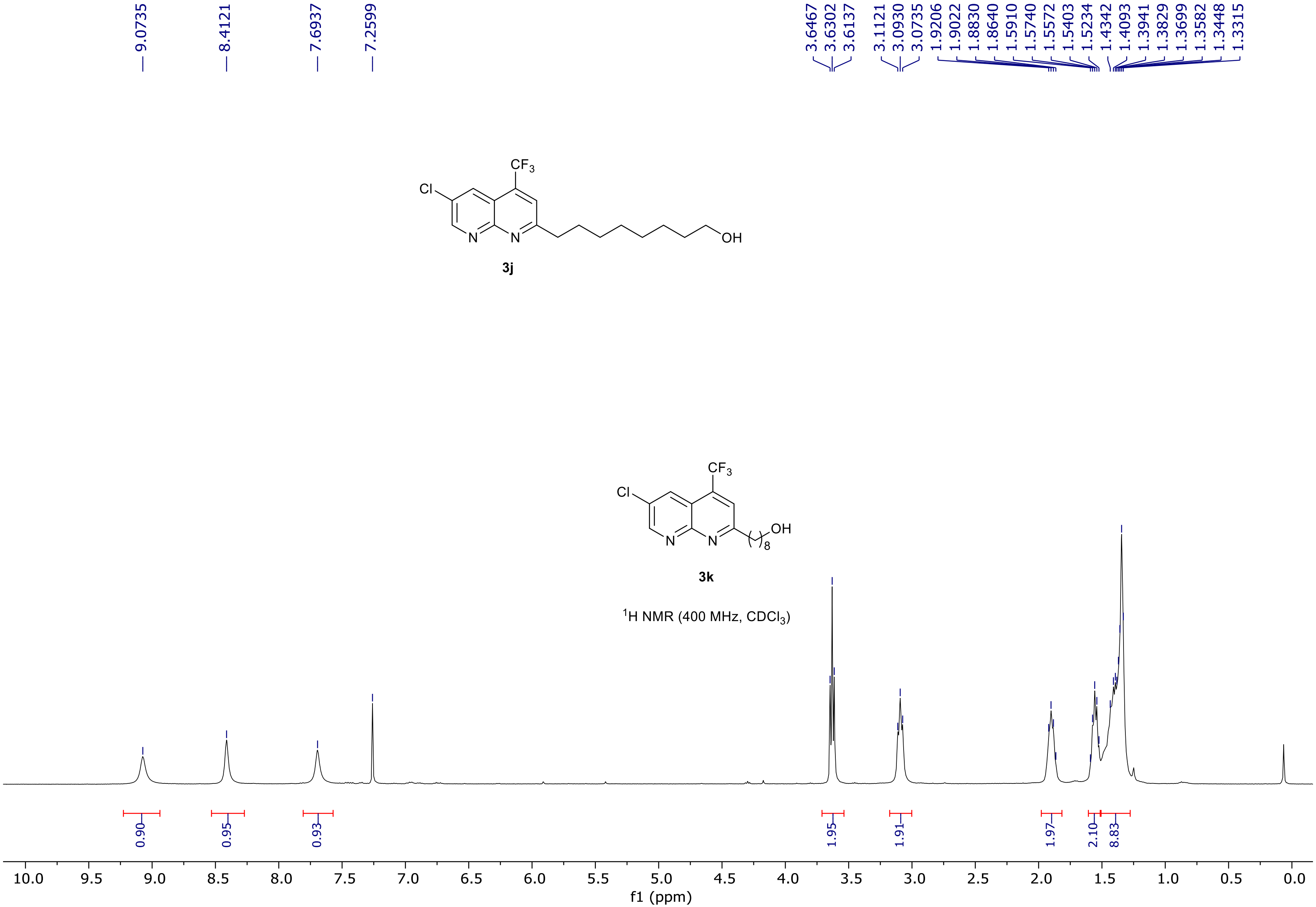
9.0735
8.4121
7.6937
7.2599
3.6467
3.6302
3.6137
3.1121
3.0930
3.0735
1.9206
1.9022
1.8830
1.8640
1.5910
1.5740
1.5572
1.5403
1.5234
1.4342
1.4093
1.3941
1.3829
1.3699
1.3582
1.3448
1.3315
CF3
Cl
N
N
OH
3j
CF3
Cl
N
N
OH
8
3k
1H NMR (400 MHz, CDCl3)
0.90
0.95
0.93
1.95
1.91
1.97
2.10
8.83
10.0
9.5
9.0
8.5
8.0
7.5
7.0
6.5
6.0
5.5
5.0
4.5
4.0
3.5
3.0
2.5
2.0
1.5
1.0
0.5
0.0
f1 (ppm)

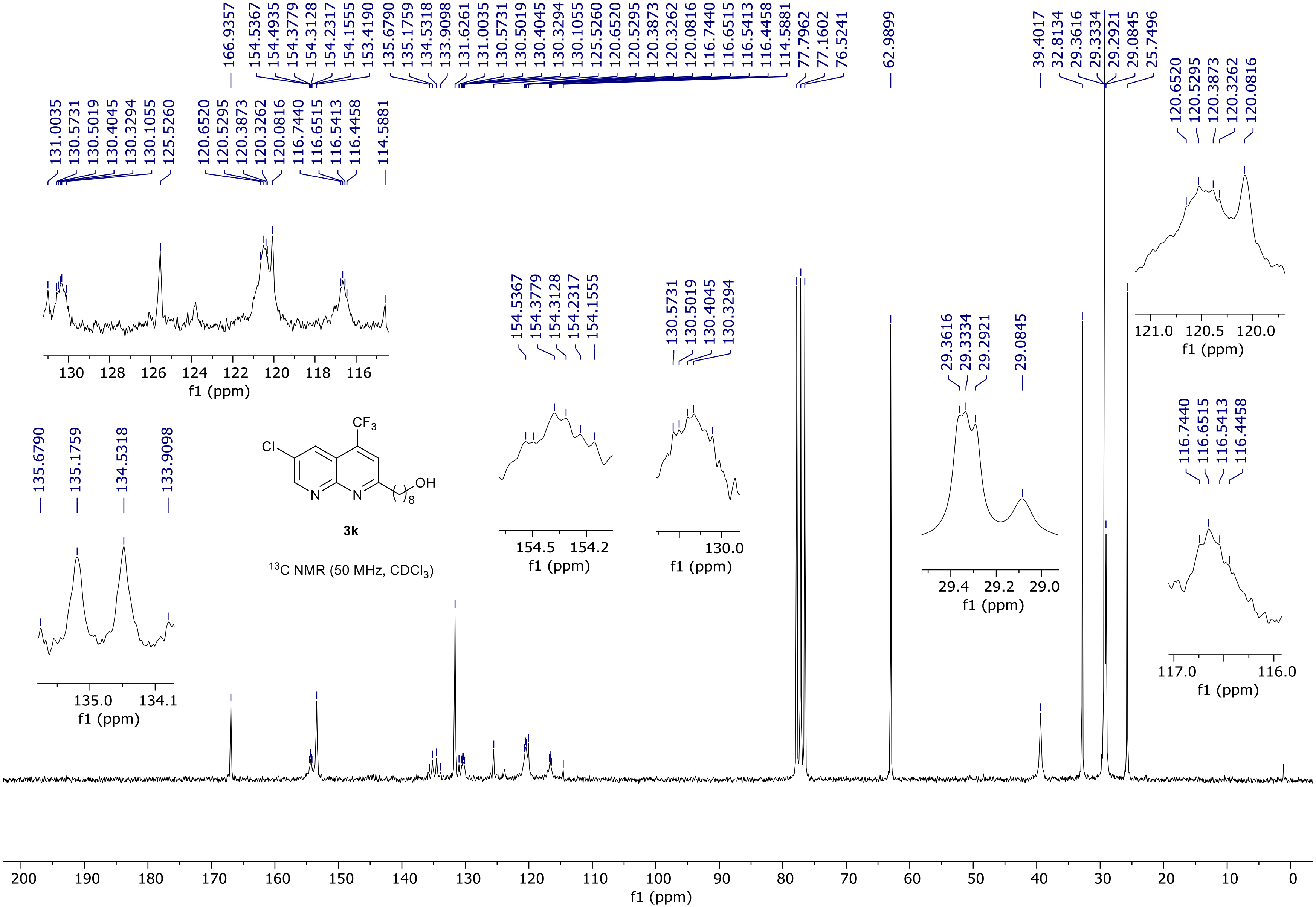
166.9357
154.5367
154.4935
154.3779
154.3128
154.2317
154.1555
153.4190
135.6790
135.1759
134.5318
133.9098
131.6261
131.0035
130.5731
130.5019
130.4045
130.3294
130.1055
125.5260
120.6520
120.5295
120.3873
120.3262
120.0816
116.7440
116.6515
116.5413
116.4458
114.5881
77.7962
77.1602
76.5241
62.9899
39.4017
32.8134
29.3616
29.3334
29.2921
29.0845
25.7496
Cl
CF3
N
N
OH
8
3k
13C NMR (50 MHz, CDCl3)
f1 (ppm)

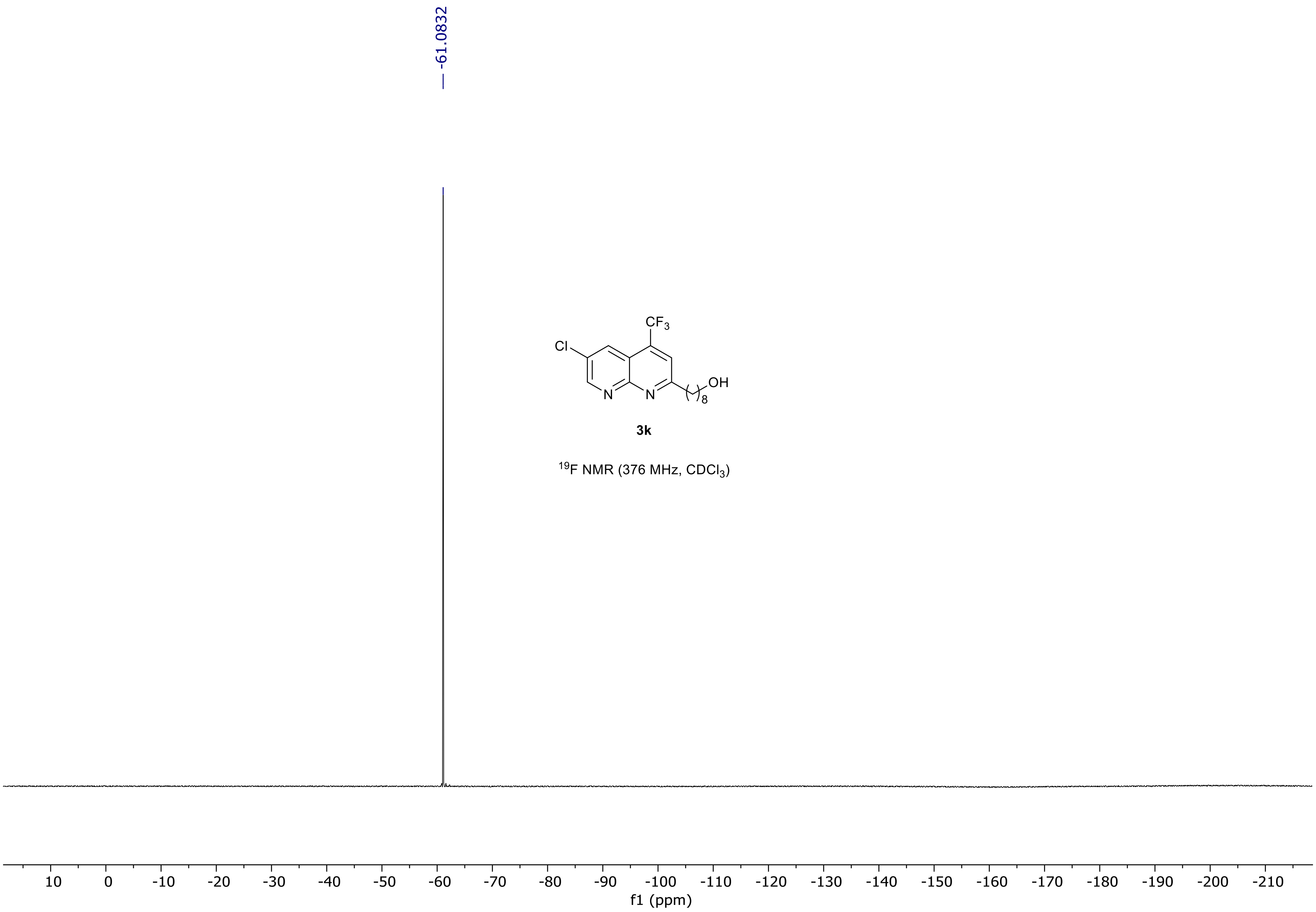

3k

^{19}F NMR (376 MHz, $CDCl_3$)

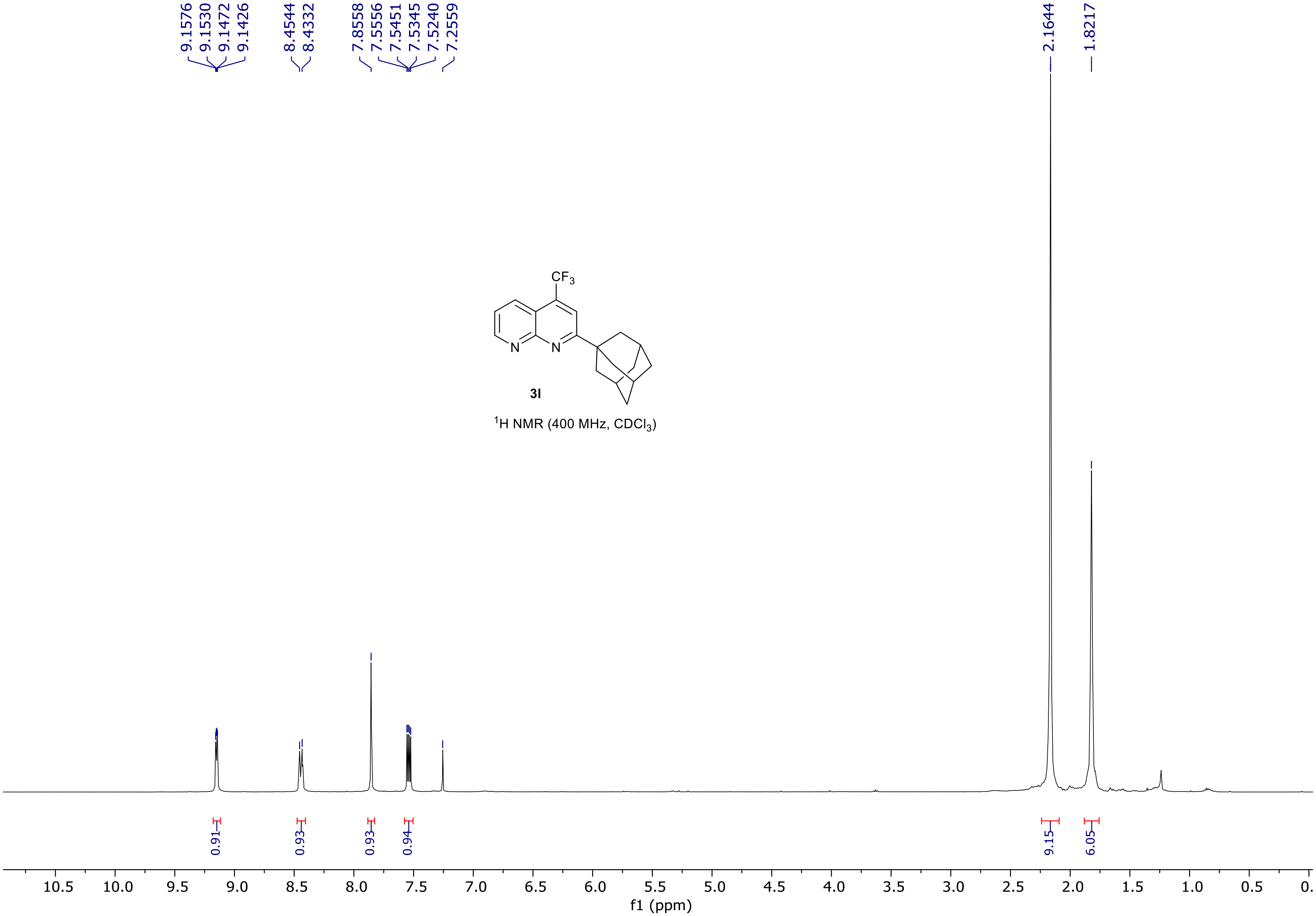
9.1576
9.1530
9.1472
9.1426
8.4544
8.4332
7.8558
7.5556
7.5451
7.5345
7.5240
7.2559
2.1644
1.8217
CF3
N
N
3l
1H NMR (400 MHz, CDCl3)
0.91
0.93
0.93
0.94
9.15
6.05
10.5 10.0 9.5 9.0 8.5 8.0 7.5 7.0 6.5 6.0 5.5 5.0 4.5 4.0 3.5 3.0 2.5 2.0 1.5 1.0 0.5 0.
f1 (ppm)

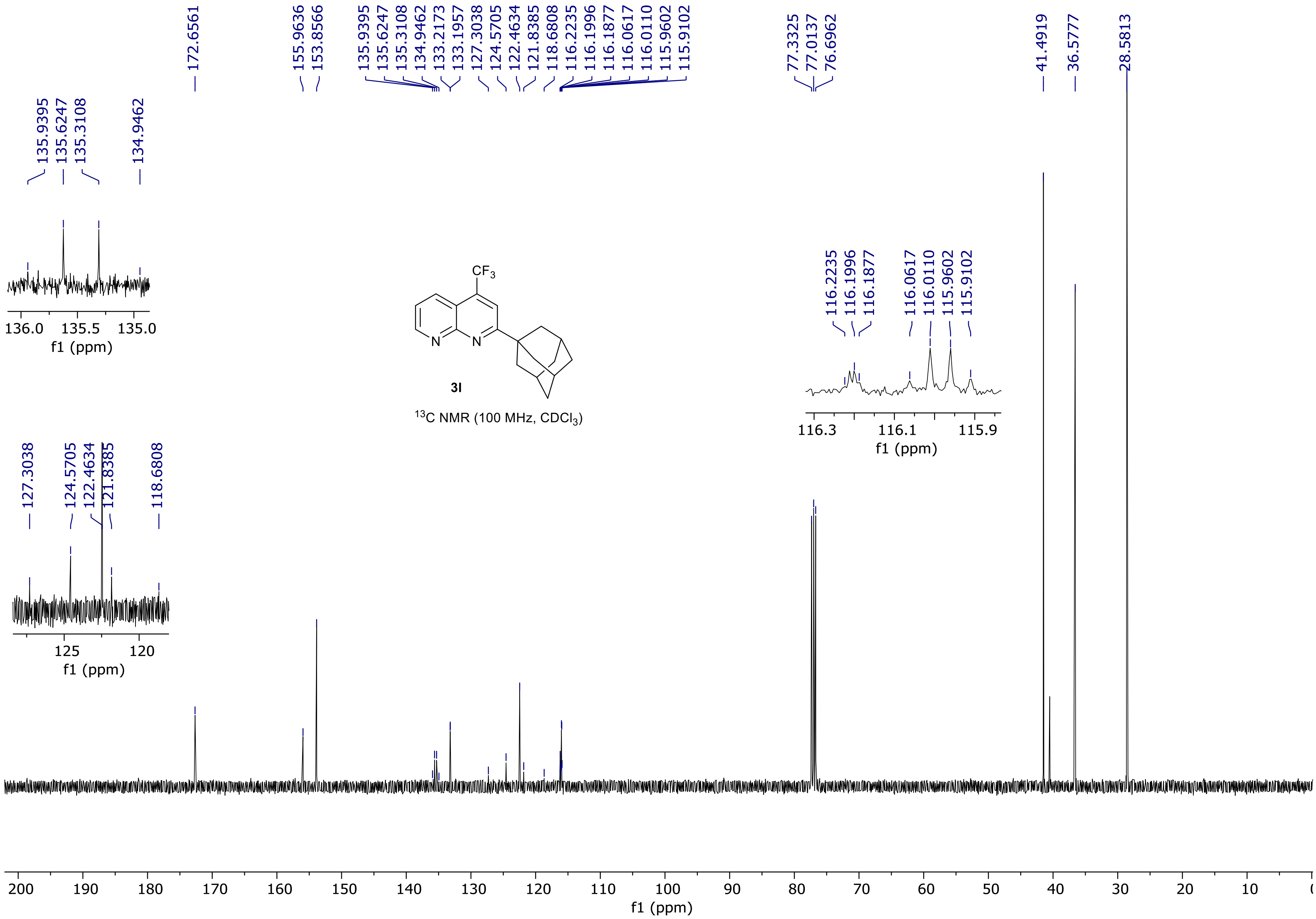
172.6561
155.9636
153.8566
135.9395
135.6247
135.3108
134.9462
133.2173
133.1957
127.3038
124.5705
122.4634
121.8385
118.6808
116.2235
116.1996
116.1877
116.0617
116.0110
115.9602
115.9102
77.3325
77.0137
76.6962
41.4919
36.5777
28.5813
CF3
N
N
3l
13C NMR (100 MHz, CDCl3)
f1 (ppm)

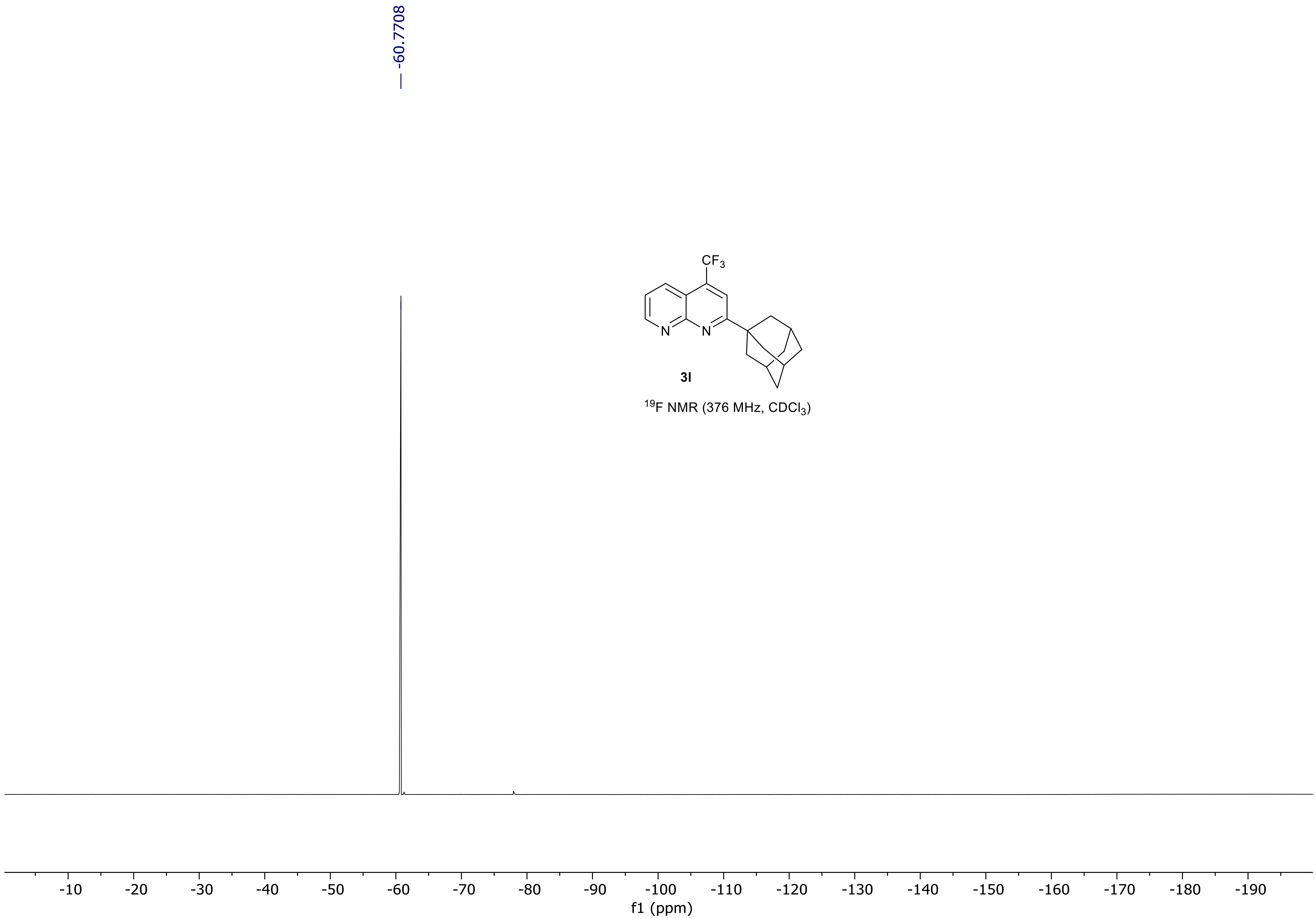
-60.7708
CF_3
N
N
3l
^{19}F NMR (376 MHz, $CDCl_3$)
-10
-20
-30
-40
-50
-60
-70
-80
-90
-100
-110
-120
-130
-140
-150
-160
-170
-180
-190
f1 (ppm)

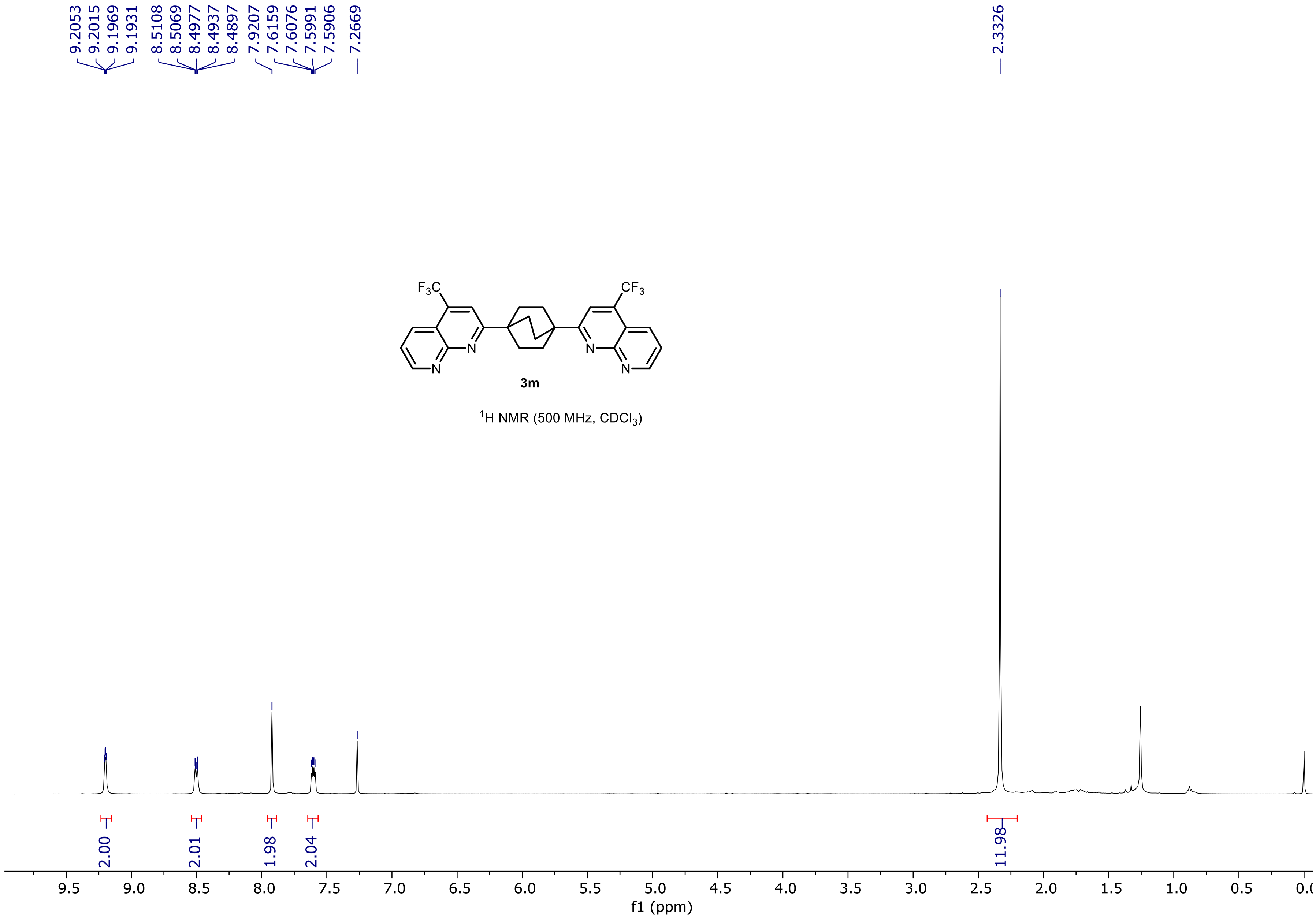

3m

^{1}H NMR (500 MHz, $CDCl_3$)

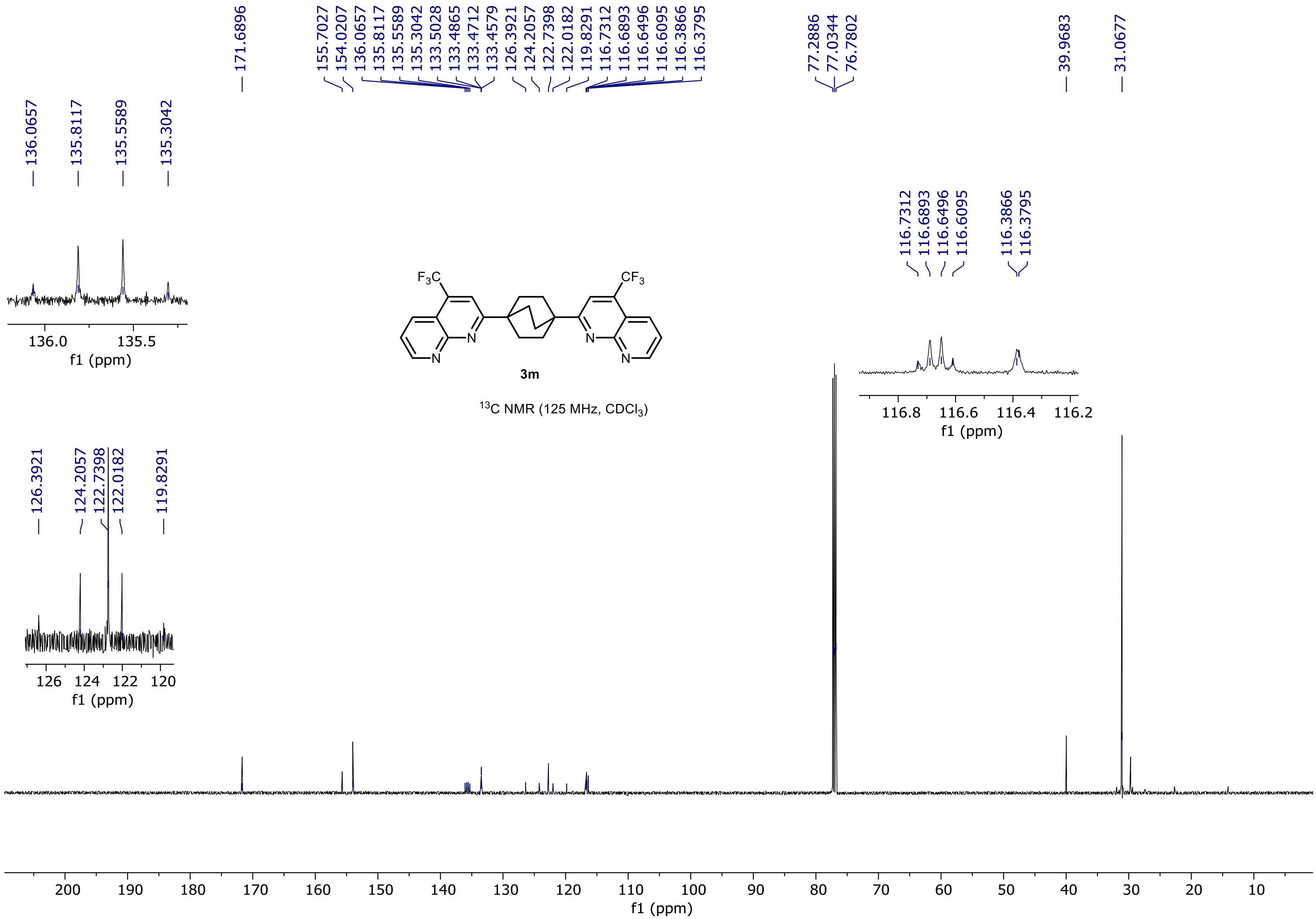
171.6896
155.7027
154.0207
136.0657
135.8117
135.5589
135.3042
133.5028
133.4865
133.4712
133.4579
126.3921
124.2057
122.7398
122.0182
119.8291
116.7312
116.6893
116.6496
116.6095
116.3866
116.3795
77.2886
77.0344
76.7802
39.9683
31.0677
F3C
CF3
N
3m
13C NMR (125 MHz, CDCl3)
f1 (ppm)

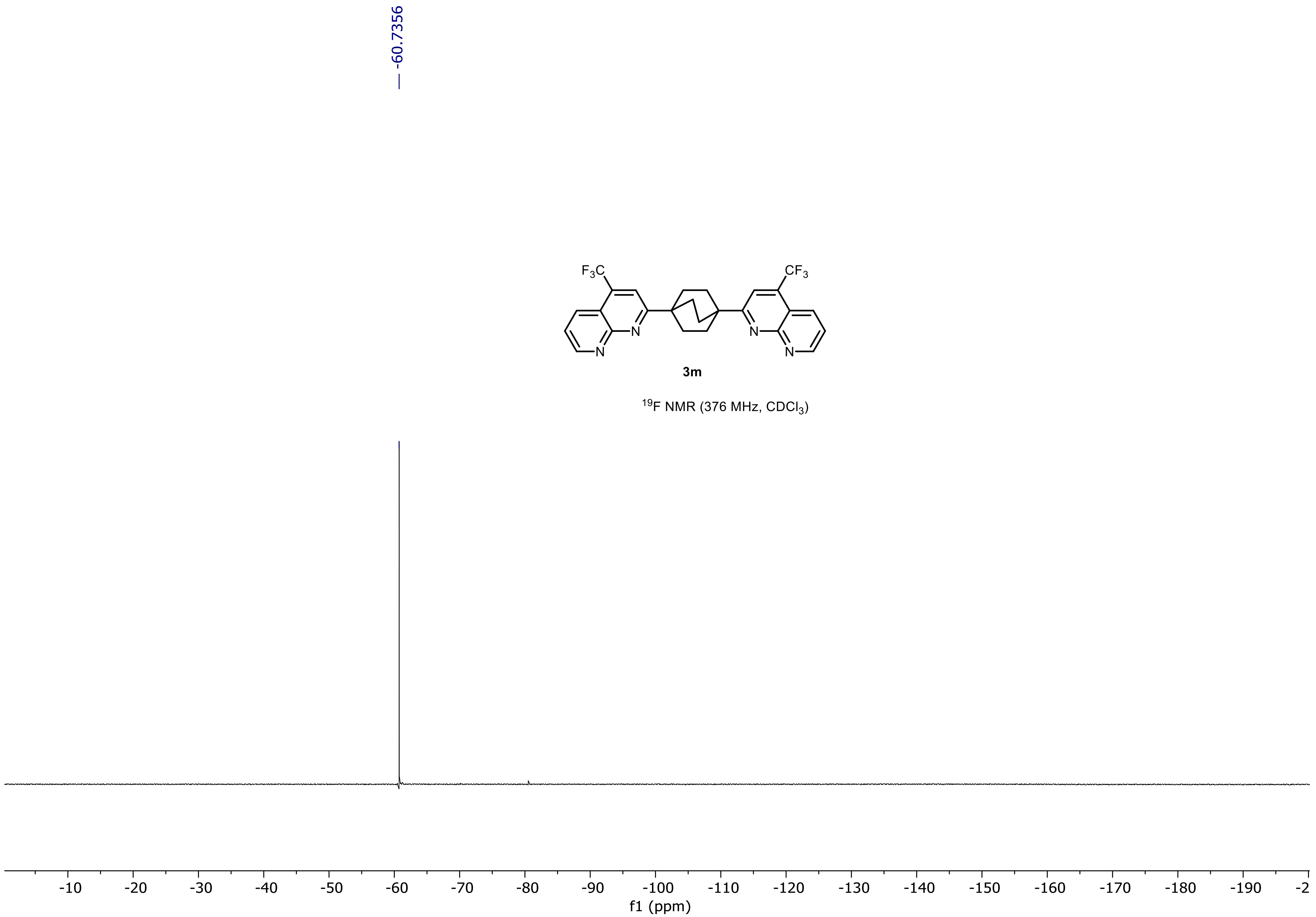

-60.7356
F3C
CF3
N
N
N
N
3m
19F NMR (376 MHz, CDCl3)
-10 -20 -30 -40 -50 -60 -70 -80 -90 -100 -110 -120 -130 -140 -150 -160 -170 -180 -190 -2
f1 (ppm)

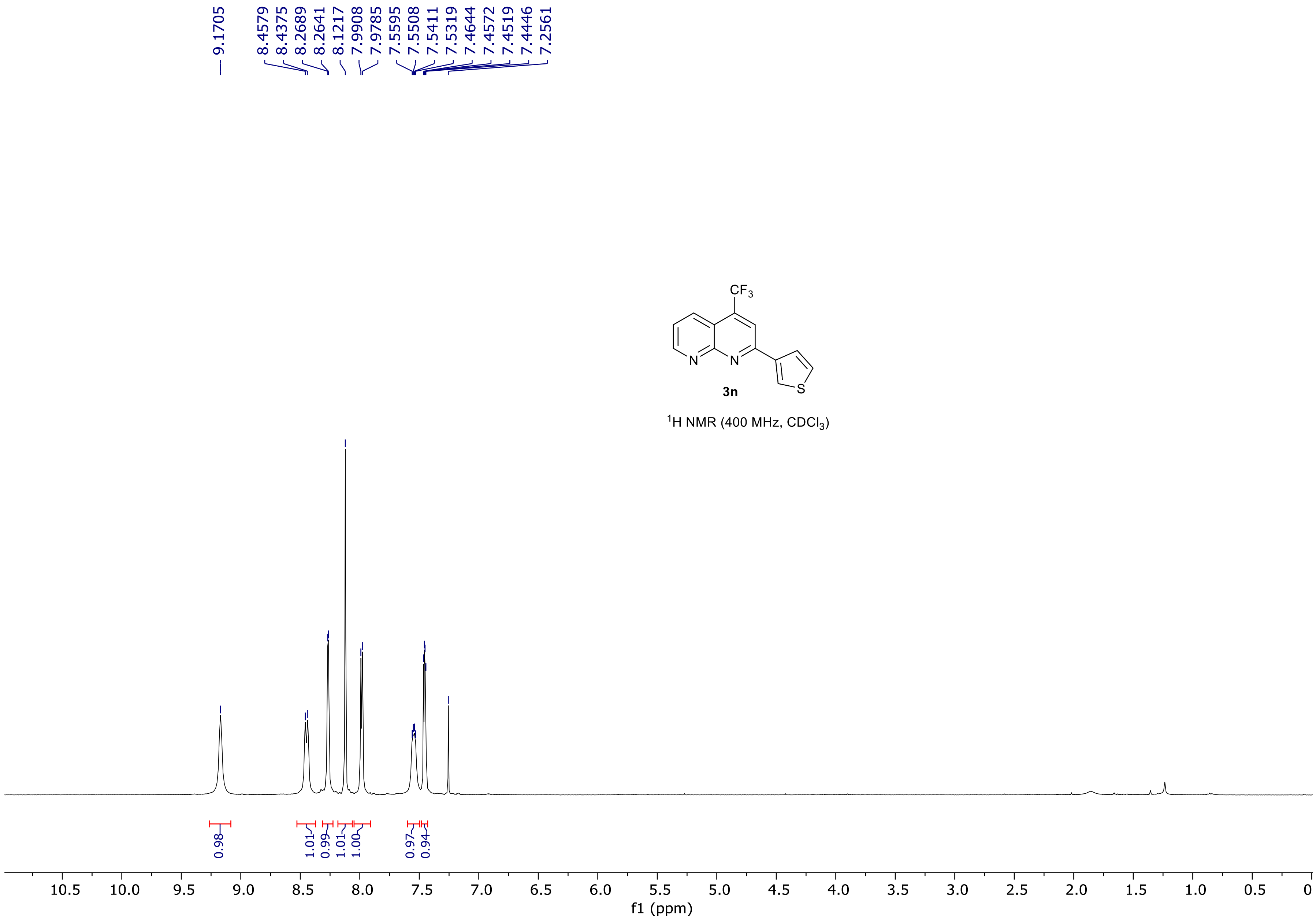
9.1705
8.4579
8.4375
8.2689
8.2641
8.1217
7.9908
7.9785
7.5595
7.5508
7.5411
7.5319
7.4644
7.4572
7.4519
7.4446
7.2561
CF3
N
N
S
3n
1H NMR (400 MHz, CDCl3)
0.98
1.01
0.99
1.01
1.00
0.97
0.94
10.5
10.0
9.5
9.0
8.5
8.0
7.5
7.0
6.5
6.0
5.5
5.0
4.5
4.0
3.5
3.0
2.5
2.0
1.5
1.0
0.5
0
f1 (ppm)

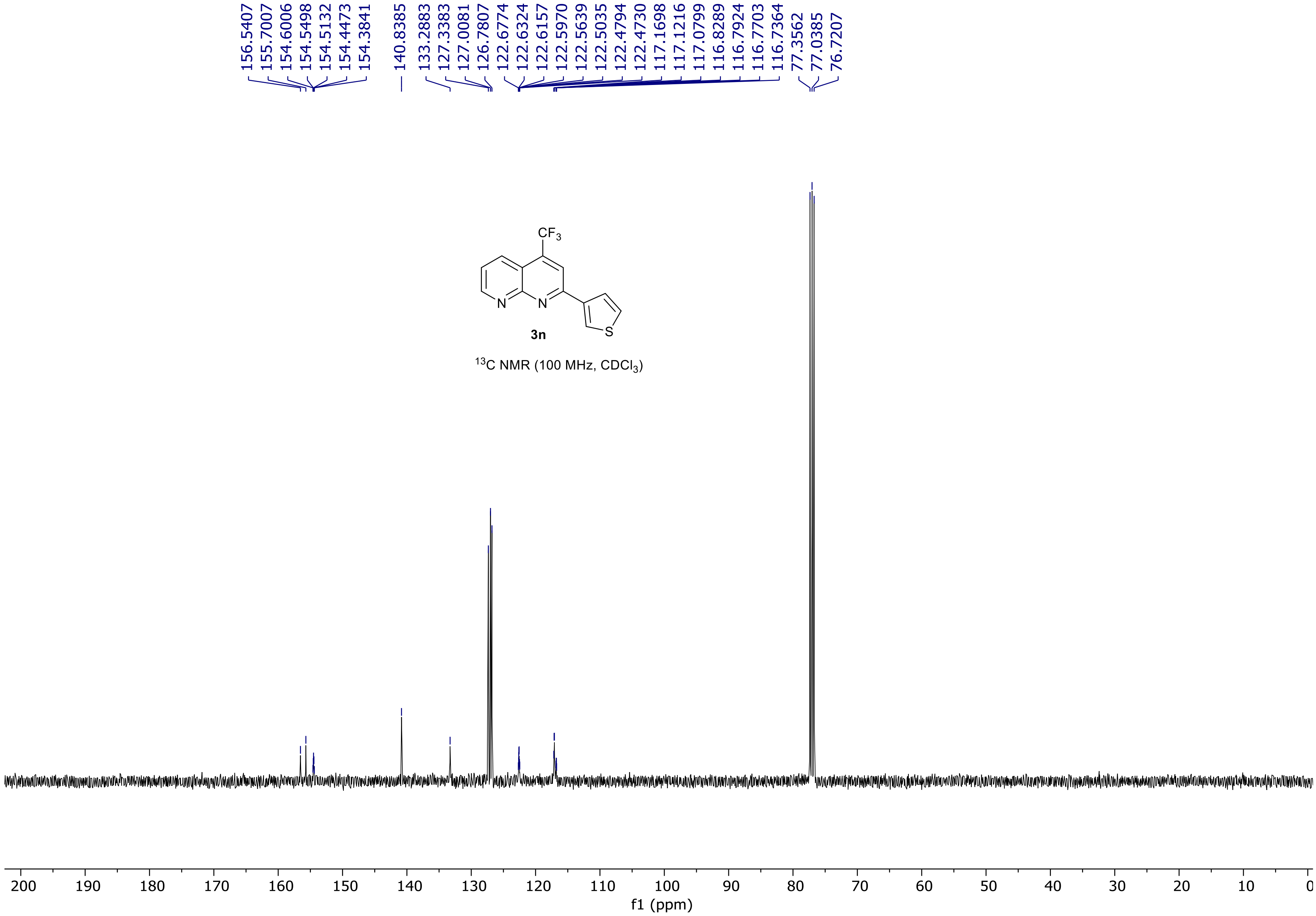
156.5407
155.7007
154.6006
154.5498
154.5132
154.4473
154.3841
140.8385
133.2883
127.3383
127.0081
126.7807
122.6774
122.6324
122.6157
122.5970
122.5639
122.5035
122.4794
122.4730
117.1698
117.1216
117.0799
116.8289
116.7924
116.7703
116.7364
77.3562
77.0385
76.7207
CF3
N
N
S
3n
13C NMR (100 MHz, CDCl3)
200 190 180 170 160 150 140 130 120 110 100 90 80 70 60 50 40 30 20 10 0
f1 (ppm)

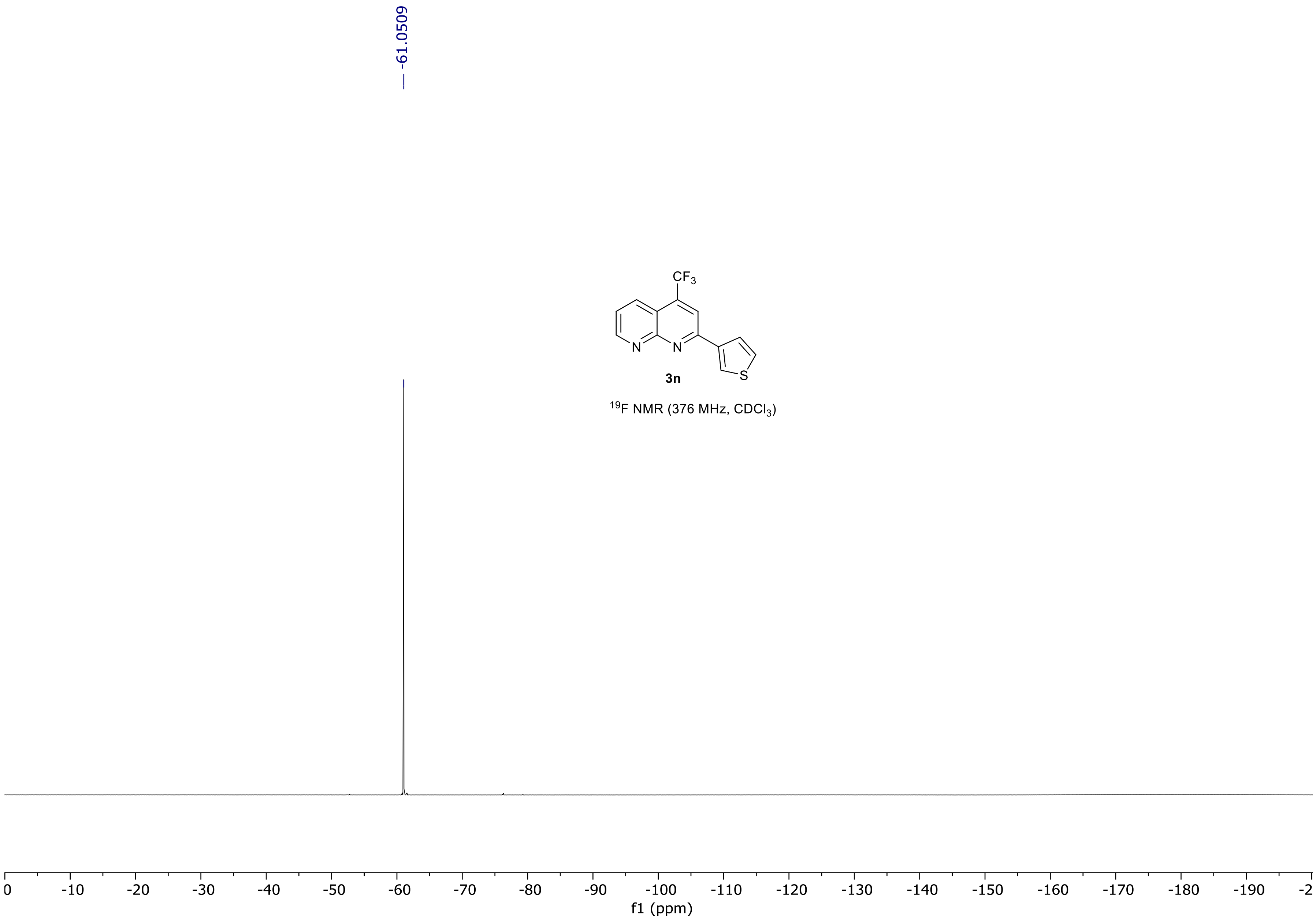
-61.0509
CF3
N
N
S
3n
19F NMR (376 MHz, CDCl3)
0 -10 -20 -30 -40 -50 -60 -70 -80 -90 -100 -110 -120 -130 -140 -150 -160 -170 -180 -190 -2
f1 (ppm)

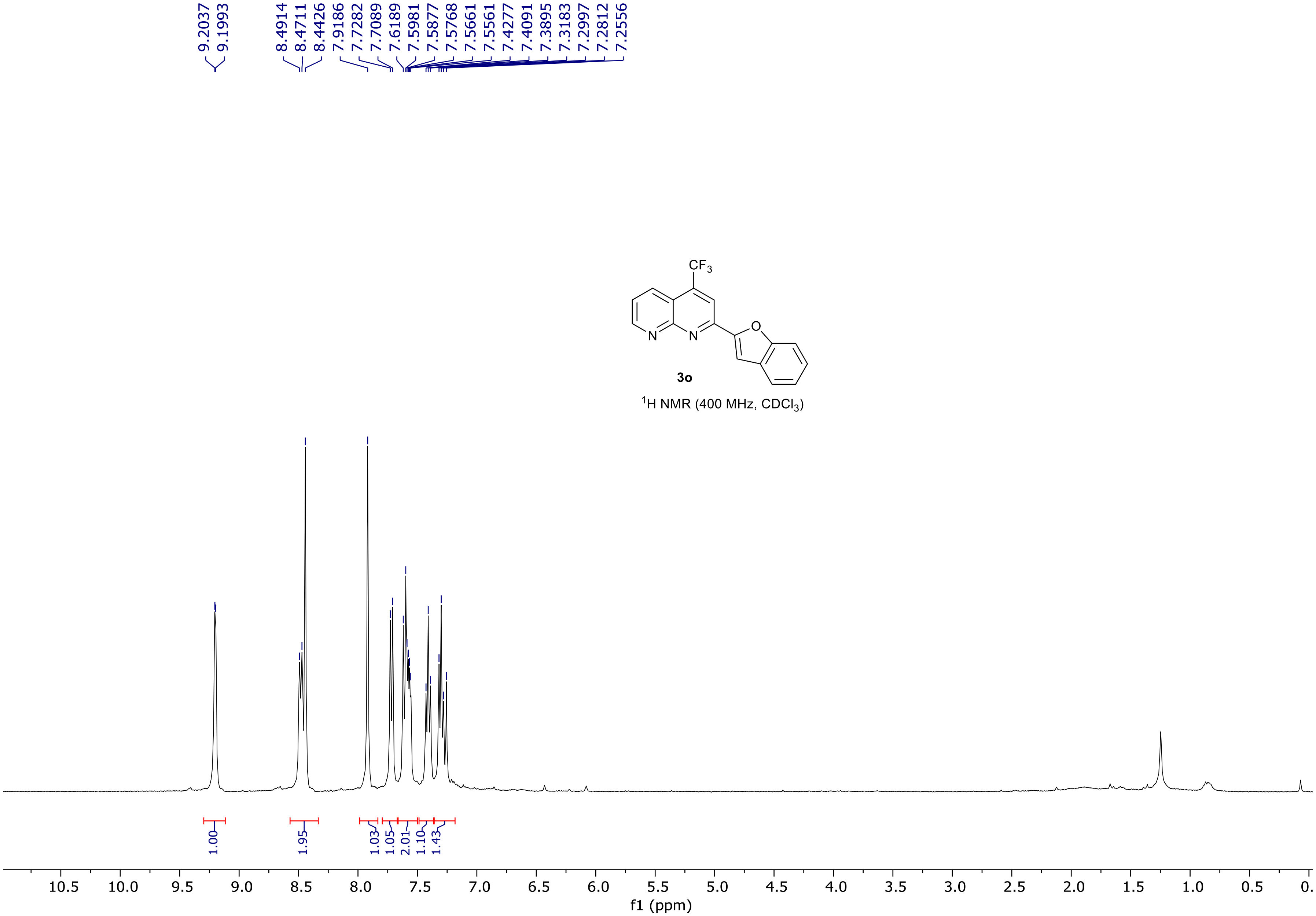
9.2037
9.1993
8.4914
8.4711
8.4426
7.9186
7.7282
7.7089
7.6189
7.5981
7.5877
7.5768
7.5661
7.5561
7.4277
7.4091
7.3895
7.3183
7.2997
7.2812
7.2556
CF3
N
N
O
3o
1H NMR (400 MHz, CDCl3)
1.00
1.95
1.03
1.05
2.01
1.10
1.43
10.5
10.0
9.5
9.0
8.5
8.0
7.5
7.0
6.5
6.0
5.5
5.0
4.5
4.0
3.5
3.0
2.5
2.0
1.5
1.0
0.5
0.
f1 (ppm)

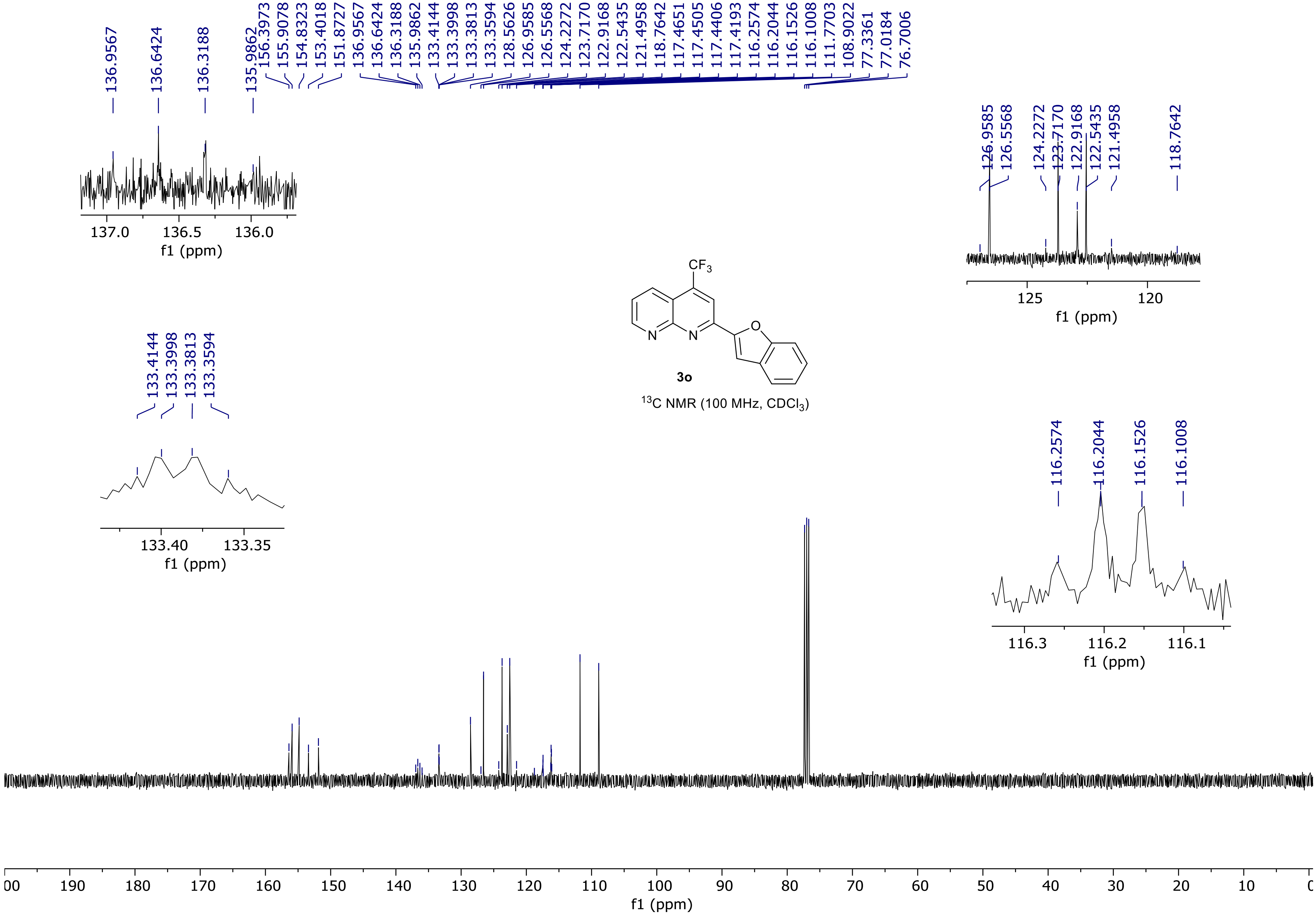
156.3973
155.9078
154.8323
153.4018
151.8727
136.9567
136.6424
136.3188
135.9862
133.4144
133.3998
133.3813
133.3594
128.5626
126.9585
126.5568
124.2272
123.7170
122.9168
122.5435
121.4958
118.7642
117.4651
117.4505
117.4406
117.4193
116.2574
116.2044
116.1526
116.1008
111.7703
108.9022
77.3361
77.0184
76.7006
CF3
N
N
O
3o
13C NMR (100 MHz, CDCl3)
f1 (ppm)

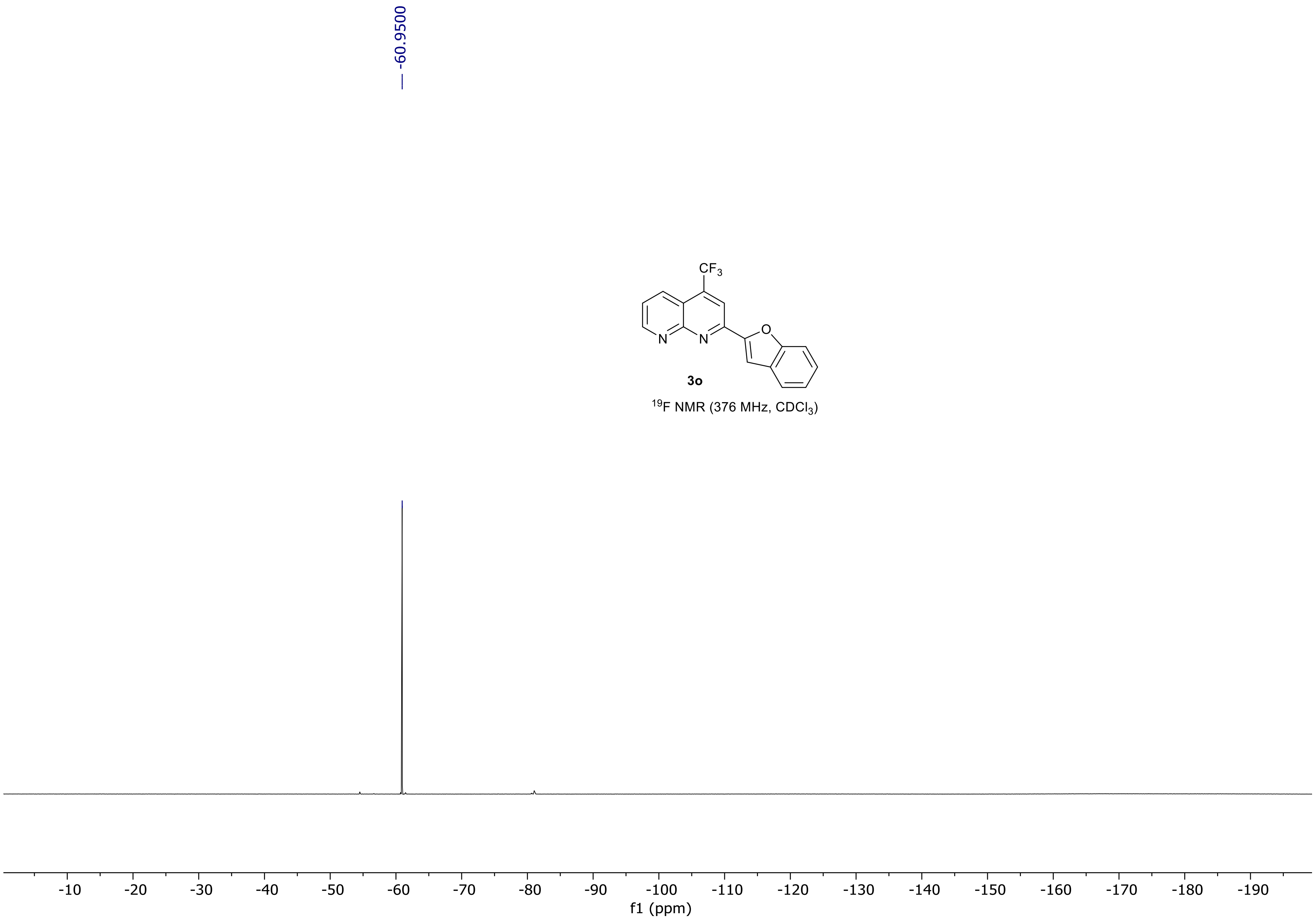

3o

^{19}F NMR (376 MHz, $CDCl_3$)

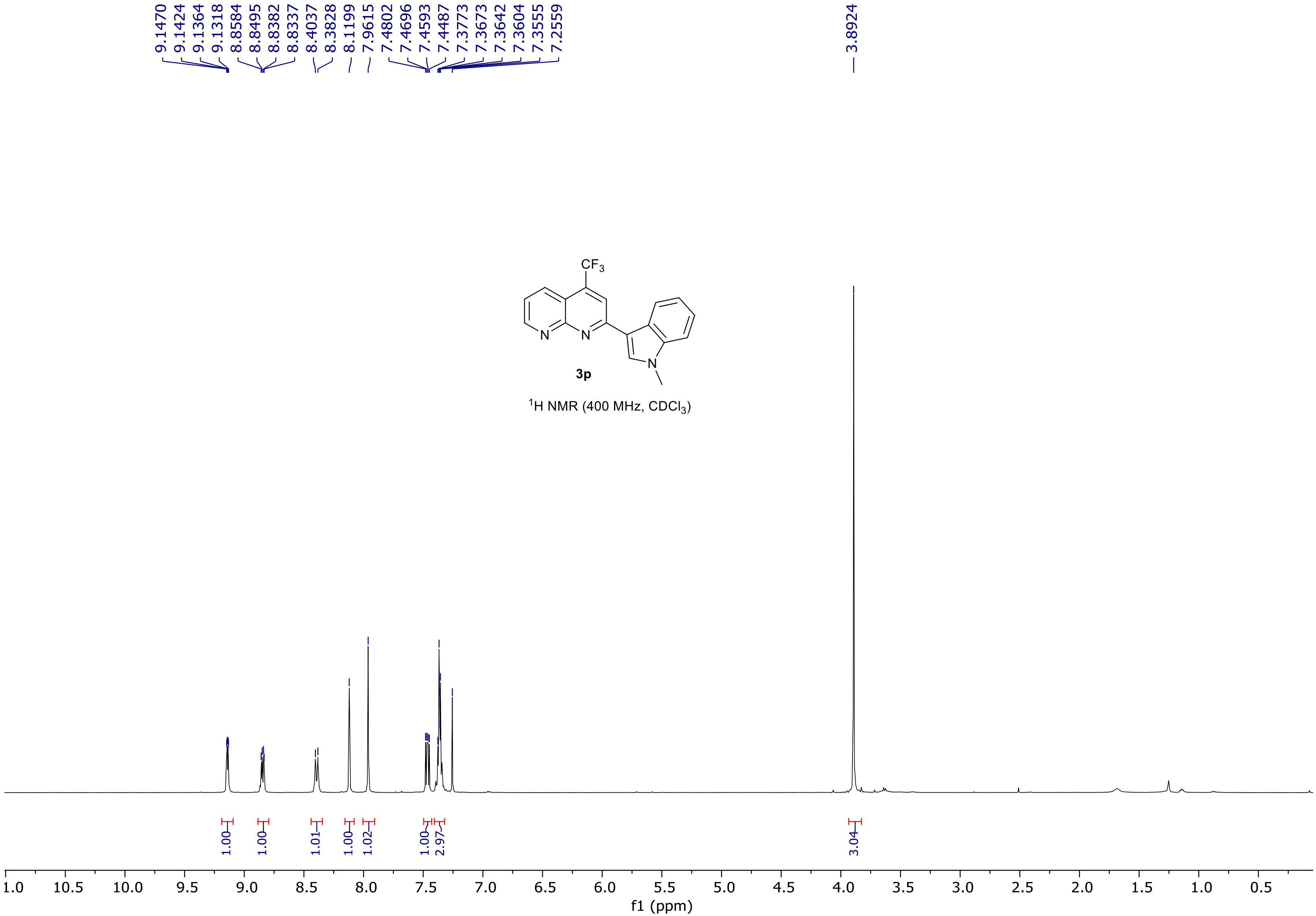
9.1470
9.1424
9.1364
9.1318
8.8584
8.8495
8.8382
8.8337
8.4037
8.3828
8.1199
7.9615
7.4802
7.4696
7.4593
7.4487
7.3773
7.3673
7.3642
7.3604
7.3555
7.2559
3.8924
CF3
N
N
N
3p
1H NMR (400 MHz, CDCl3)
1.00
1.00
1.01
1.00
1.02
1.00
2.97
3.04
f1 (ppm)

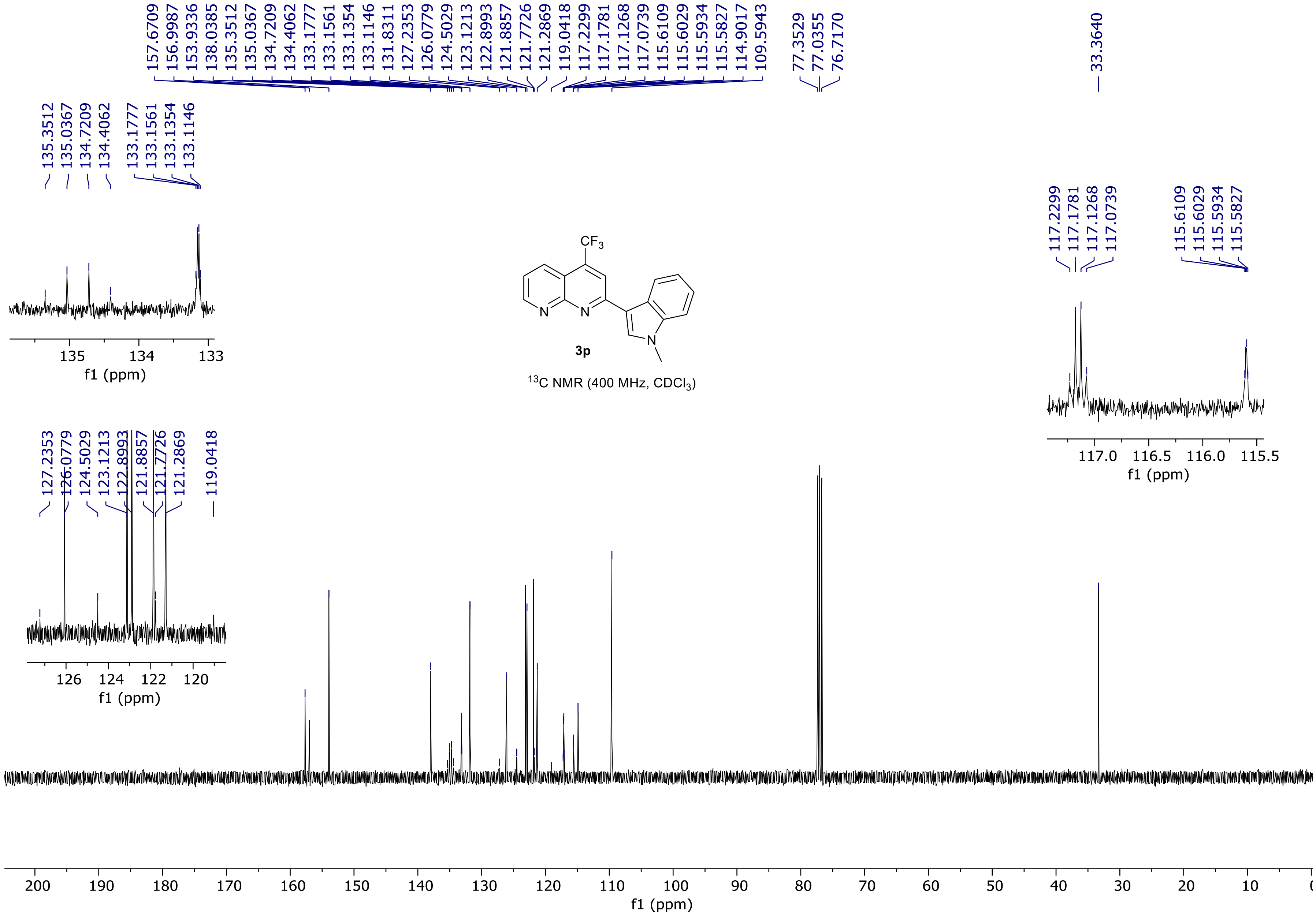
157.6709
156.9987
153.9336
138.0385
135.3512
135.0367
134.7209
134.4062
133.1777
133.1561
133.1354
133.1146
131.8311
127.2353
126.0779
124.5029
123.1213
122.8993
121.8857
121.7726
121.2869
119.0418
117.2299
117.1781
117.1268
117.0739
115.6109
115.6029
115.5934
115.5827
114.9017
109.5943
77.3529
77.0355
76.7170
33.3640
CF3
N
N
N
3p
13C NMR (400 MHz, CDCl3)
f1 (ppm)

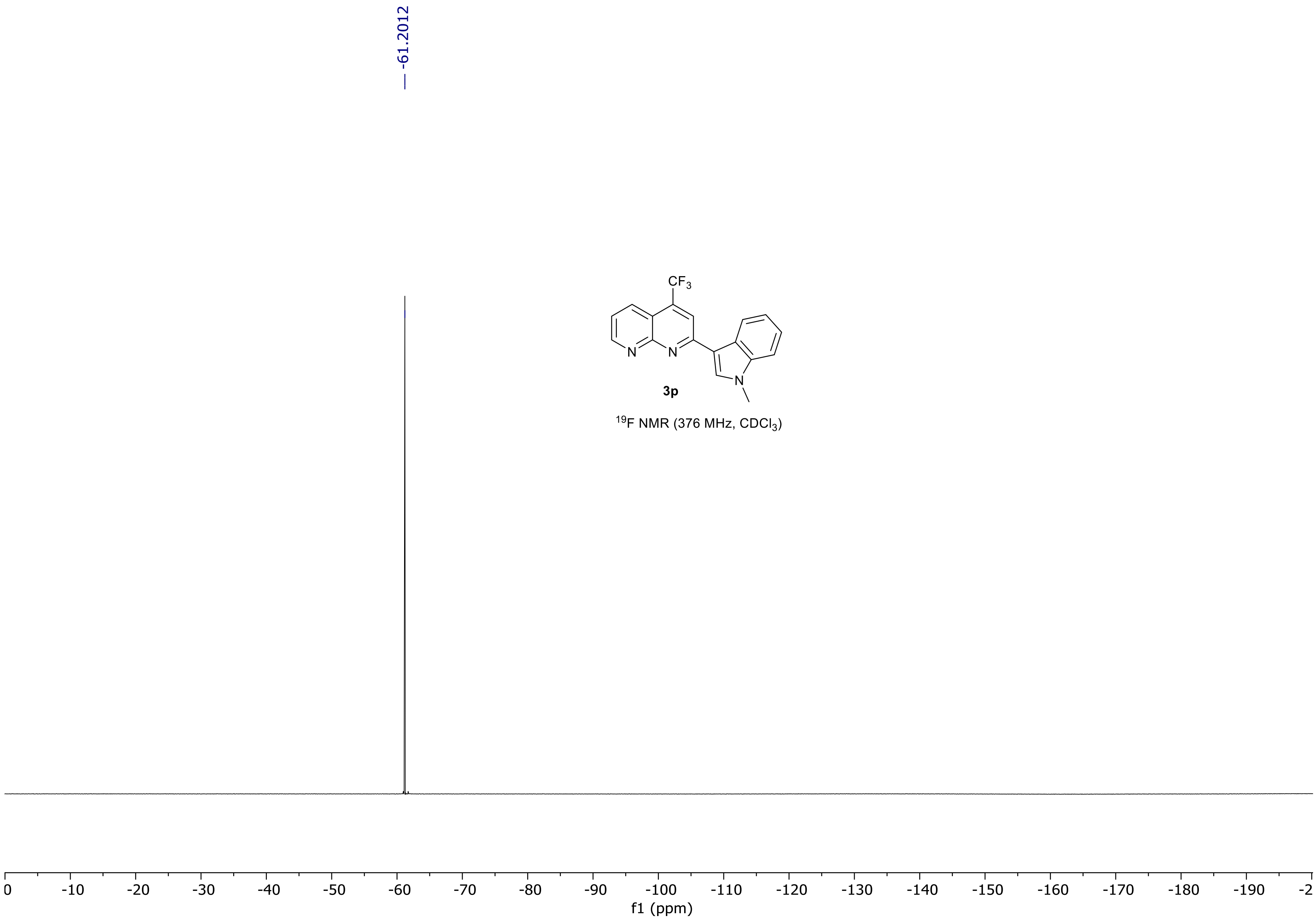
-61.2012
CF3
N
N
N
3p
19F NMR (376 MHz, CDCl3)
0 -10 -20 -30 -40 -50 -60 -70 -80 -90 -100 -110 -120 -130 -140 -150 -160 -170 -180 -190 -2
f1 (ppm)

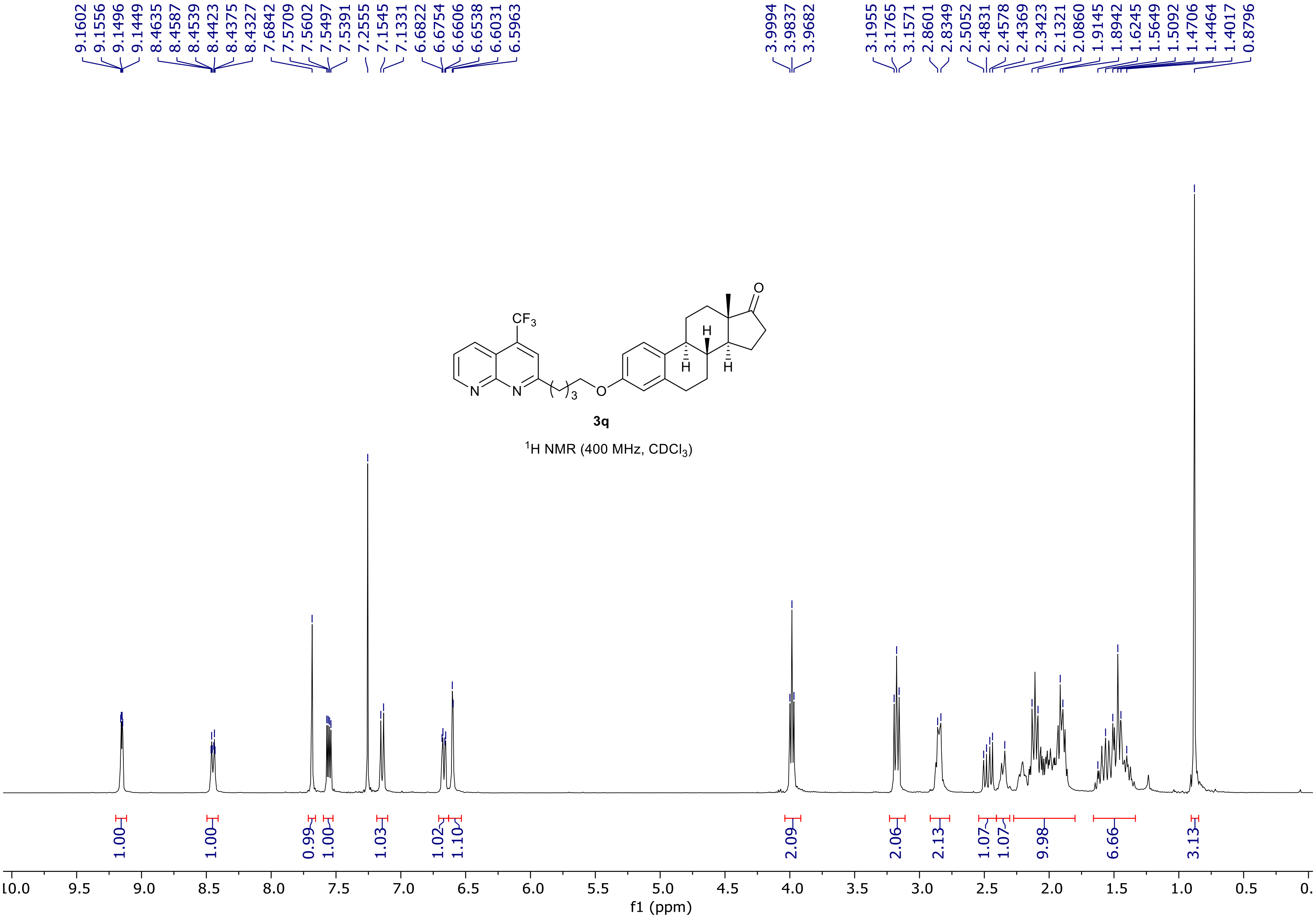
9.1602
9.1556
9.1496
9.1449
8.4635
8.4587
8.4539
8.4423
8.4375
8.4327
7.6842
7.5709
7.5602
7.5497
7.5391
7.2555
7.1545
7.1331
6.6822
6.6754
6.6606
6.6538
6.6031
6.5963
3.9994
3.9837
3.9682
3.1955
3.1765
3.1571
2.8601
2.8349
2.5052
2.4831
2.4578
2.4369
2.3423
2.1321
2.0860
1.9145
1.8942
1.6245
1.5649
1.5092
1.4706
1.4464
1.4017
0.8796
CF3
N
N
O
H
H
H
O
3q
1H NMR (400 MHz, CDCl3)
1.00
1.00
0.99
1.00
1.03
1.02
1.10
2.09
2.06
2.13
1.07
1.07
9.98
6.66
3.13
10.0
9.5
9.0
8.5
8.0
7.5
7.0
6.5
6.0
5.5
5.0
4.5
4.0
3.5
3.0
2.5
2.0
1.5
1.0
0.5
f1 (ppm)

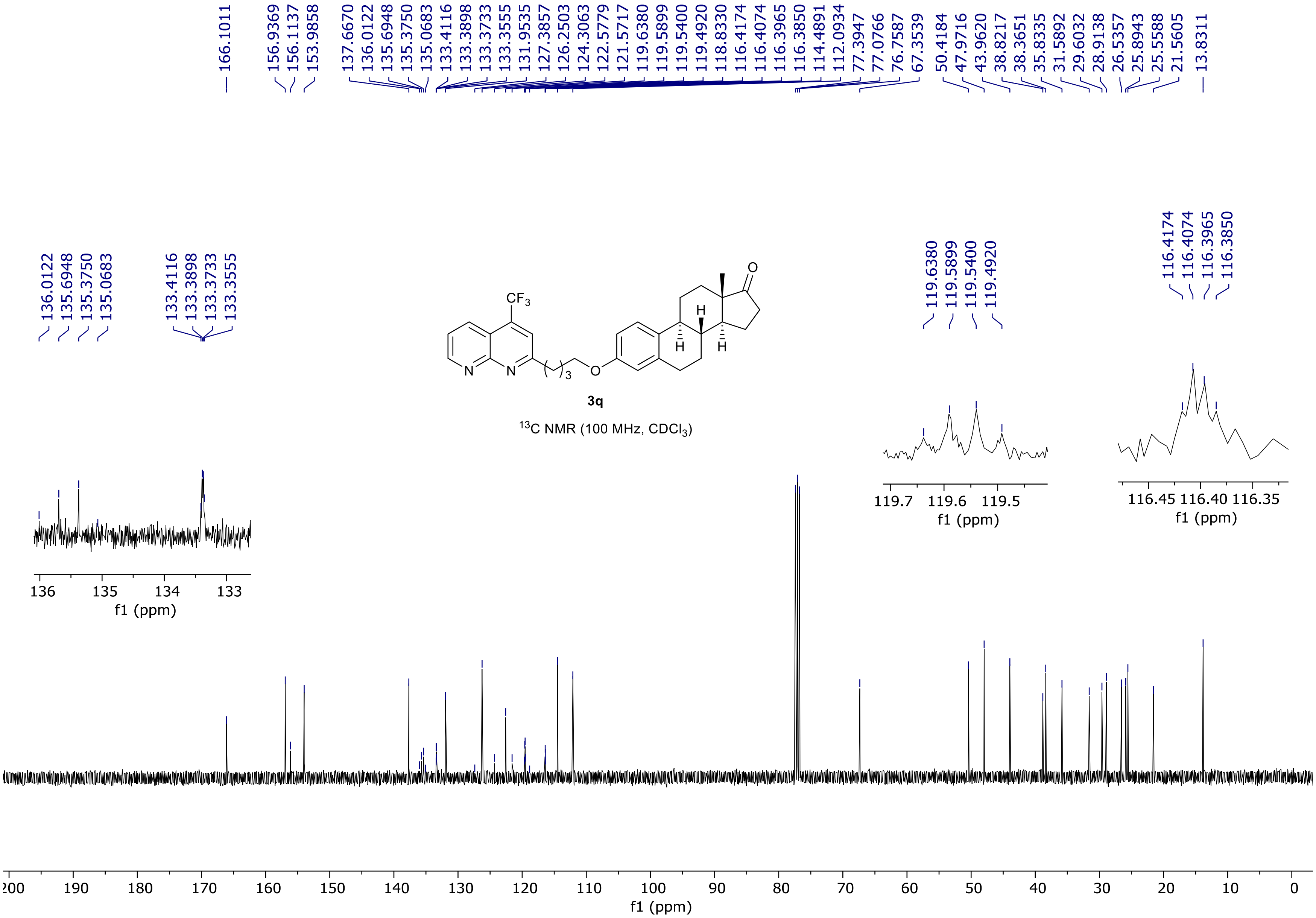
166.1011
156.9369
156.1137
153.9858
137.6670
136.0122
135.6948
135.3750
135.0683
133.4116
133.3898
133.3733
133.3555
131.9535
127.3857
126.2503
124.3063
122.5779
121.5717
119.6380
119.5899
119.5400
119.4920
118.8330
116.4174
116.4074
116.3965
116.3850
114.4891
112.0934
77.3947
77.0766
76.7587
67.3539
50.4184
47.9716
43.9620
38.8217
38.3651
35.8335
31.5892
29.6032
28.9138
26.5357
25.8943
25.5588
21.5605
13.8311
CF3
N
O
H
3q
^{13}C NMR (100 MHz, CDCl$_3$)
f1 (ppm)

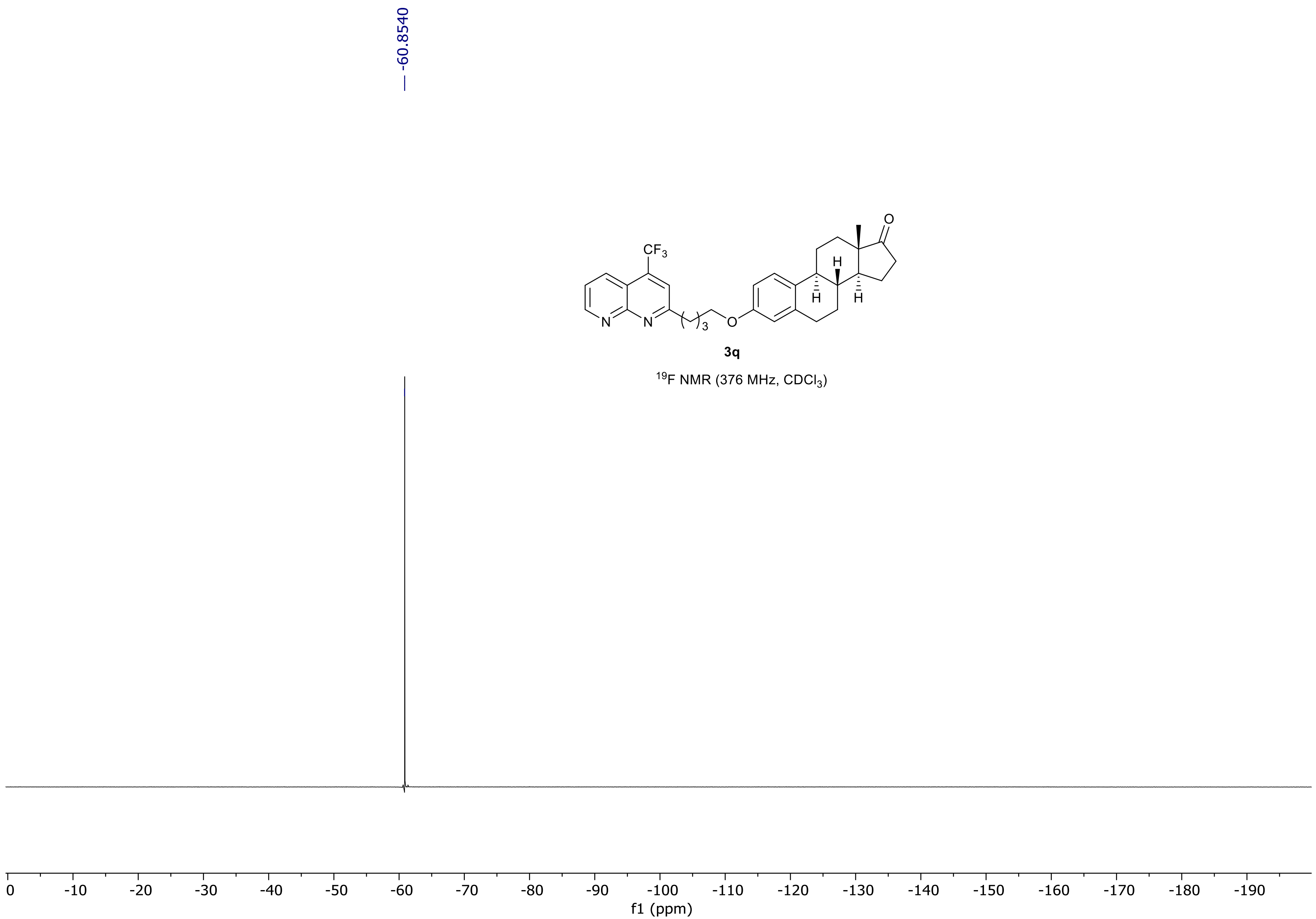
-60.8540
CF3
N
N
O
H
H
H
3
3q
19F NMR (376 MHz, CDCl3)
0 -10 -20 -30 -40 -50 -60 -70 -80 -90 -100 -110 -120 -130 -140 -150 -160 -170 -180 -190
f1 (ppm)

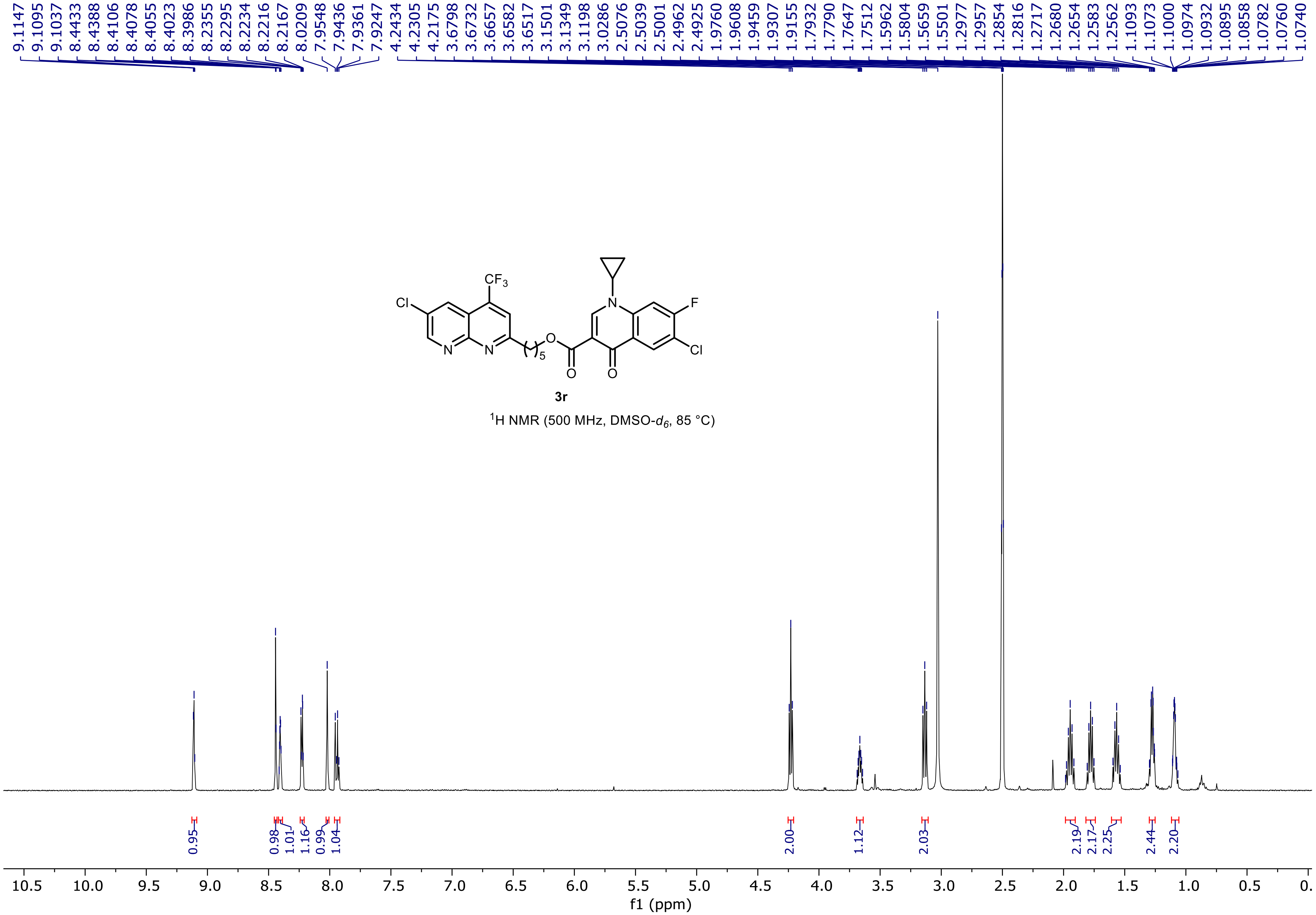
9.1147
9.1095
9.1037
8.4433
8.4388
8.4106
8.4078
8.4055
8.4023
8.3986
8.2355
8.2295
8.2234
8.2216
8.2167
8.0209
7.9548
7.9436
7.9361
7.9247
4.2434
4.2305
4.2175
3.6798
3.6732
3.6657
3.6582
3.6517
3.1501
3.1349
3.1198
3.0286
2.5076
2.5039
2.5001
2.4962
2.4925
1.9760
1.9608
1.9459
1.9307
1.9155
1.7932
1.7790
1.7647
1.7512
1.5962
1.5804
1.5659
1.5501
1.2977
1.2957
1.2854
1.2816
1.2717
1.2680
1.2654
1.2583
1.2562
1.1093
1.1073
1.1000
1.0974
1.0932
1.0895
1.0858
1.0782
1.0760
1.0740
CF3
Cl
N
N
O
O
O
N
F
Cl
5
3r
1H NMR (500 MHz, DMSO-d6, 85 °C)
0.95
0.98
1.01
1.16
0.99
1.04
2.00
1.12
2.03
2.19
2.17
2.25
2.44
2.20
f1 (ppm)

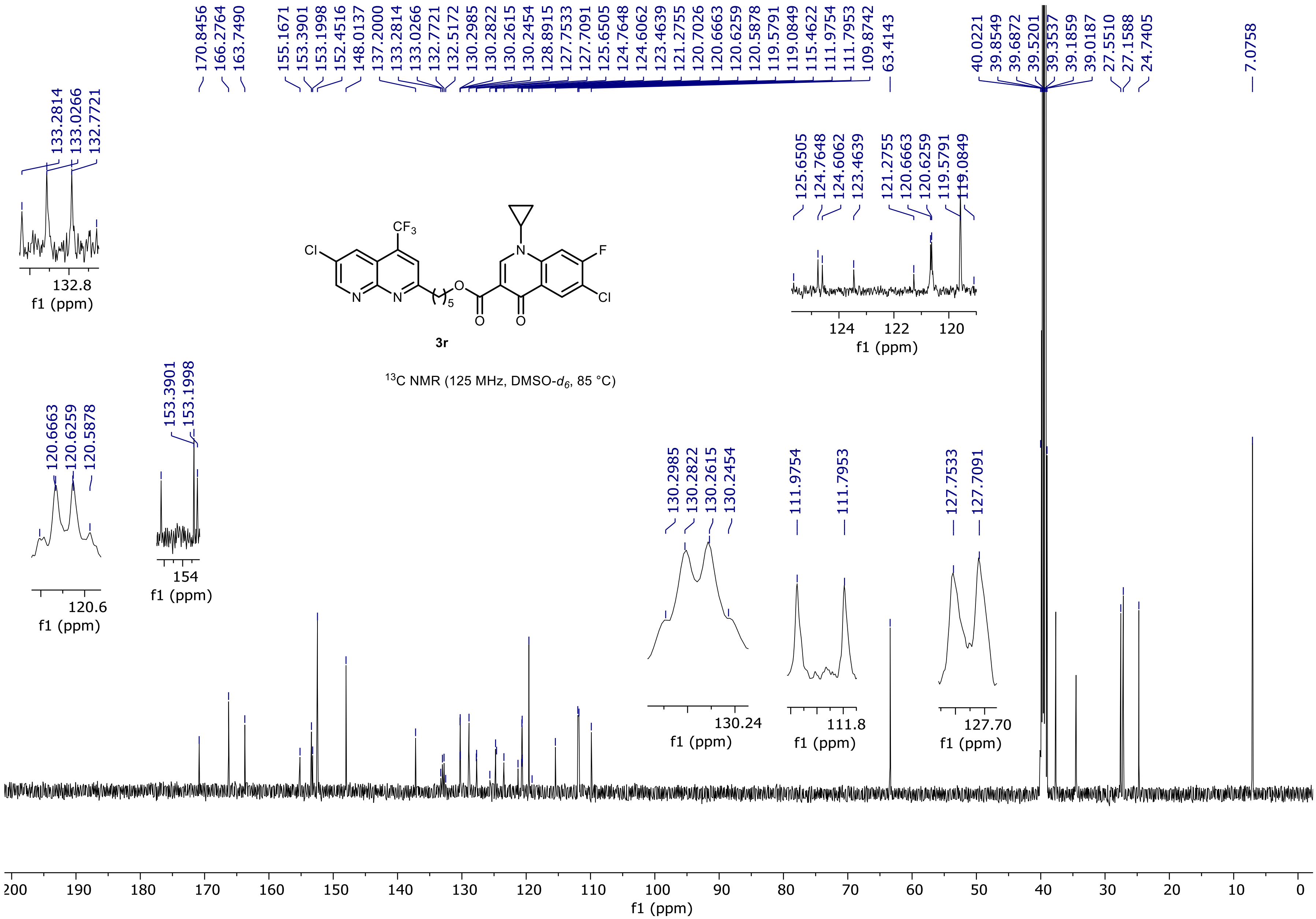

3r

^{13}C NMR (125 MHz, DMSO-d_6, 85 °C)

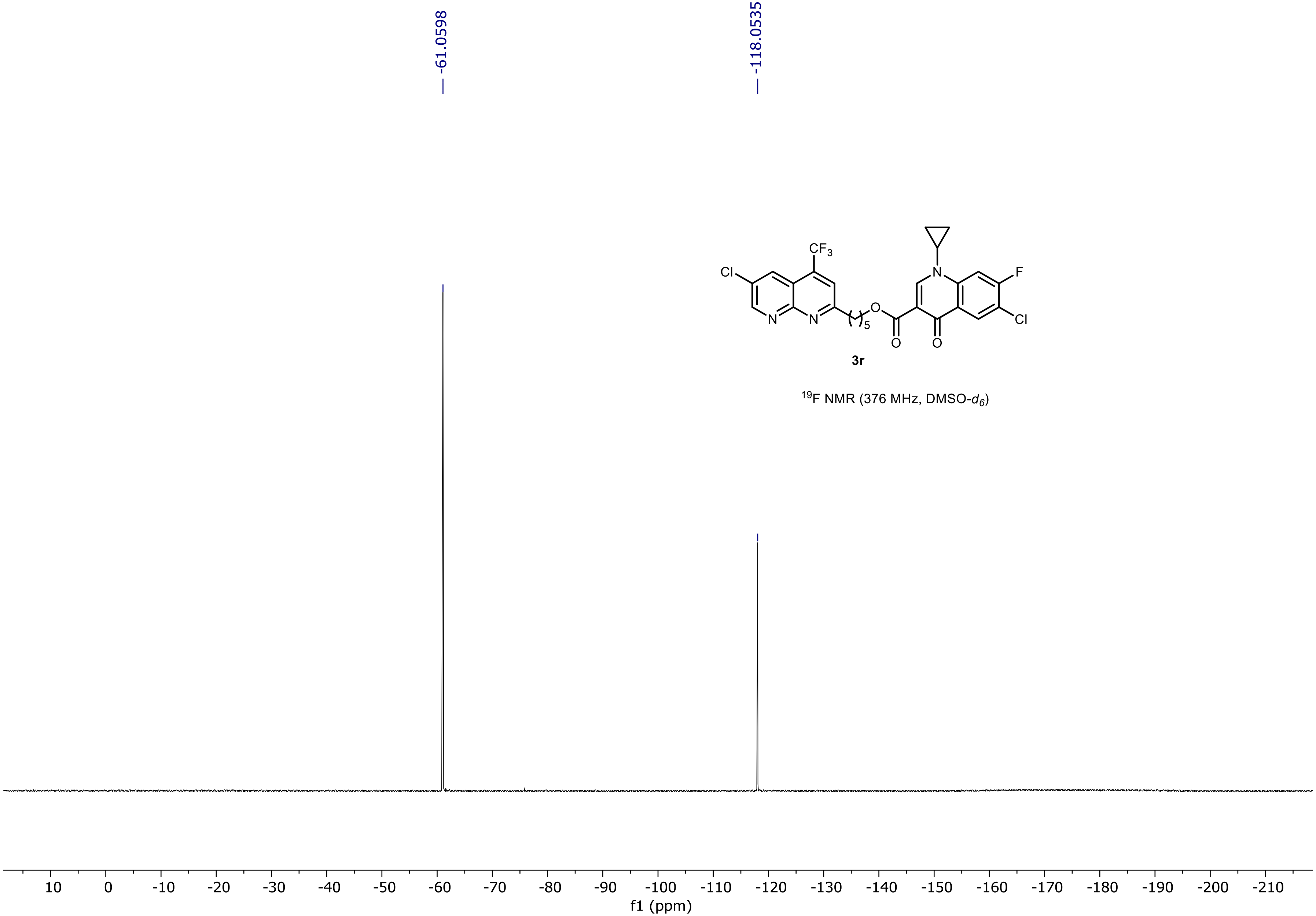
-61.0598
-118.0535
CF3
Cl
N
N
O
5
N
F
Cl
O
O
3r
19F NMR (376 MHz, DMSO-d6)
10 0 -10 -20 -30 -40 -50 -60 -70 -80 -90 -100 -110 -120 -130 -140 -150 -160 -170 -180 -190 -200 -210
f1 (ppm)

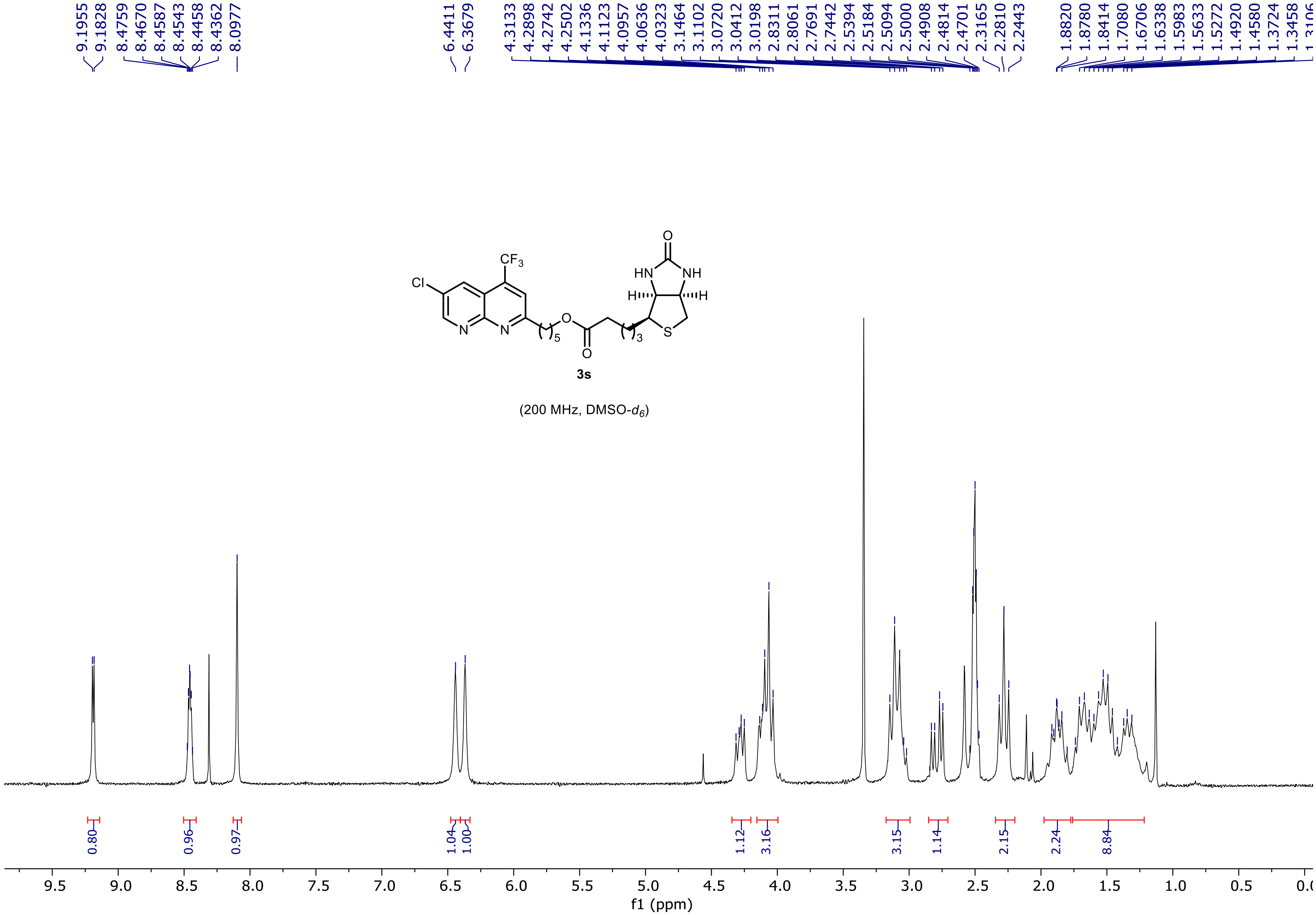
9.1955
9.1828
8.4759
8.4670
8.4587
8.4543
8.4458
8.4362
8.0977
6.4411
6.3679
4.3133
4.2898
4.2742
4.2502
4.1336
4.1123
4.0957
4.0636
4.0323
3.1464
3.1102
3.0720
3.0412
3.0198
2.8311
2.8061
2.7691
2.7442
2.5394
2.5184
2.5094
2.5000
2.4908
2.4814
2.4701
2.3165
2.2810
2.2443
1.8820
1.8780
1.8414
1.7080
1.6706
1.6338
1.5983
1.5633
1.5272
1.4920
1.4580
1.3724
1.3458
CF3
Cl
N
N
O
O
HN
NH
H
H
S
3s
(200 MHz, DMSO-d6)
0.80
0.96
0.97
1.04
1.00
1.12
3.16
3.15
1.14
2.15
2.24
8.84
9.5
9.0
8.5
8.0
7.5
7.0
6.5
6.0
5.5
5.0
4.5
4.0
3.5
3.0
2.5
2.0
1.5
1.0
0.5
f1 (ppm)

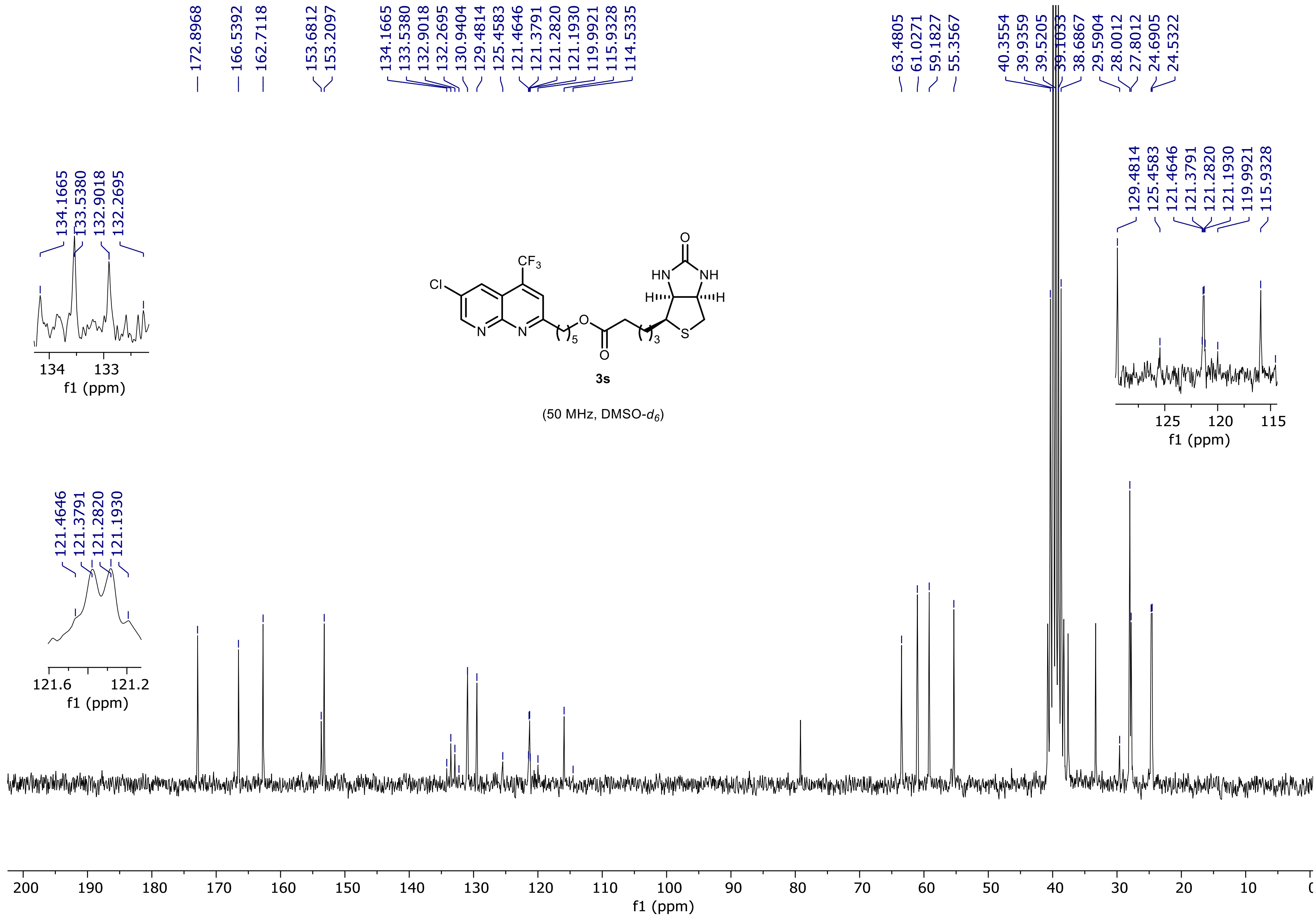
172.8968
166.5392
162.7118
153.6812
153.2097
134.1665
133.5380
132.9018
132.2695
130.9404
129.4814
125.4583
121.4646
121.3791
121.2820
121.1930
119.9921
115.9328
114.5335
63.4805
61.0271
59.1827
55.3567
40.3554
39.9359
39.5205
39.1053
38.6867
29.5904
28.0012
27.8012
24.6905
24.5322
Cl
CF3
N
HN
NH
H
O
S
5
3
3s
(50 MHz, DMSO-d6)
f1 (ppm)

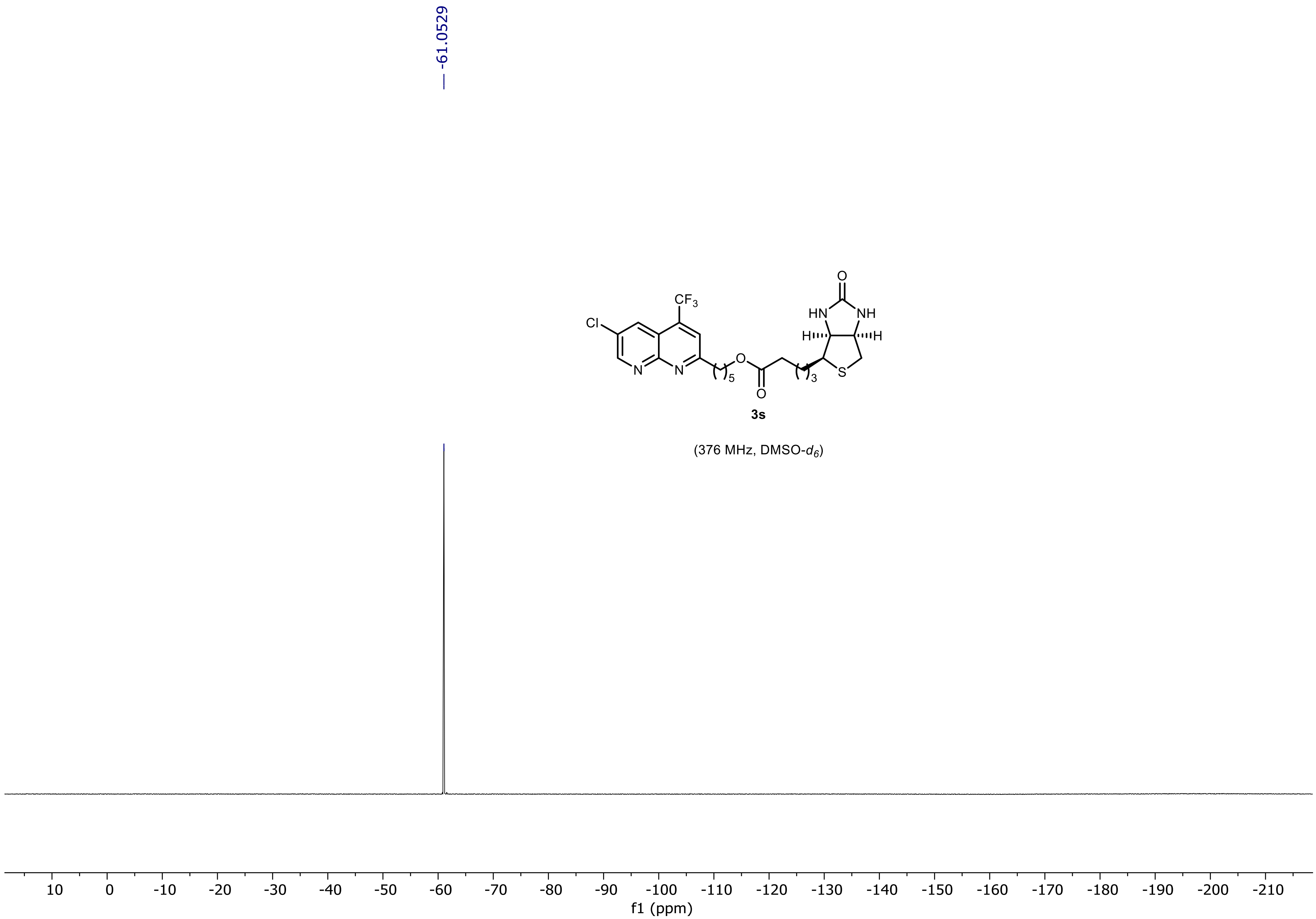
-61.0529
CF3
Cl
N
N
5
O
O
3
HN
NH
H
H
S
O
3s
(376 MHz, DMSO-d6)
10 0 -10 -20 -30 -40 -50 -60 -70 -80 -90 -100 -110 -120 -130 -140 -150 -160 -170 -180 -190 -200 -210
f1 (ppm)

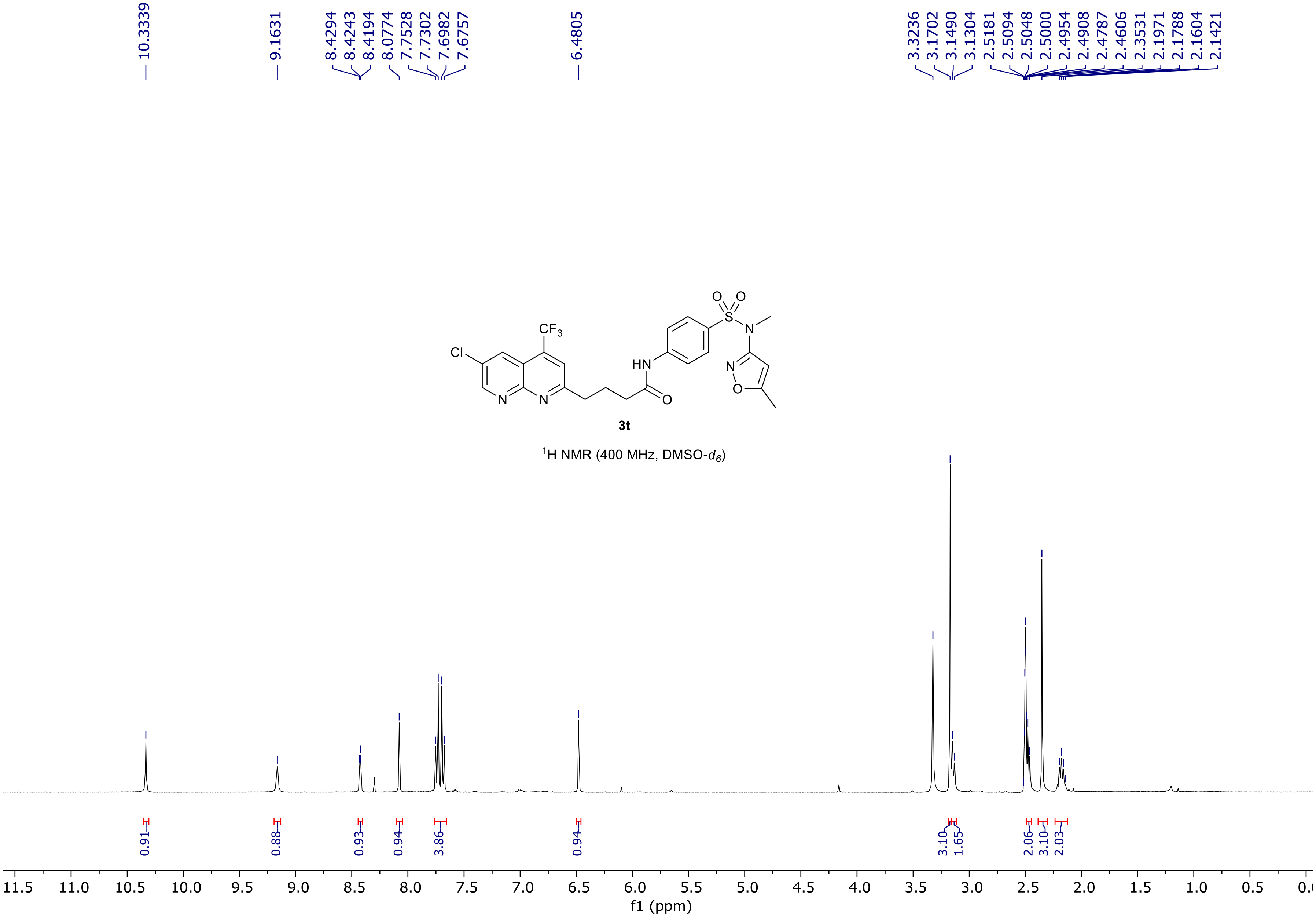

3t

^{1}H NMR (400 MHz, DMSO-d_6)

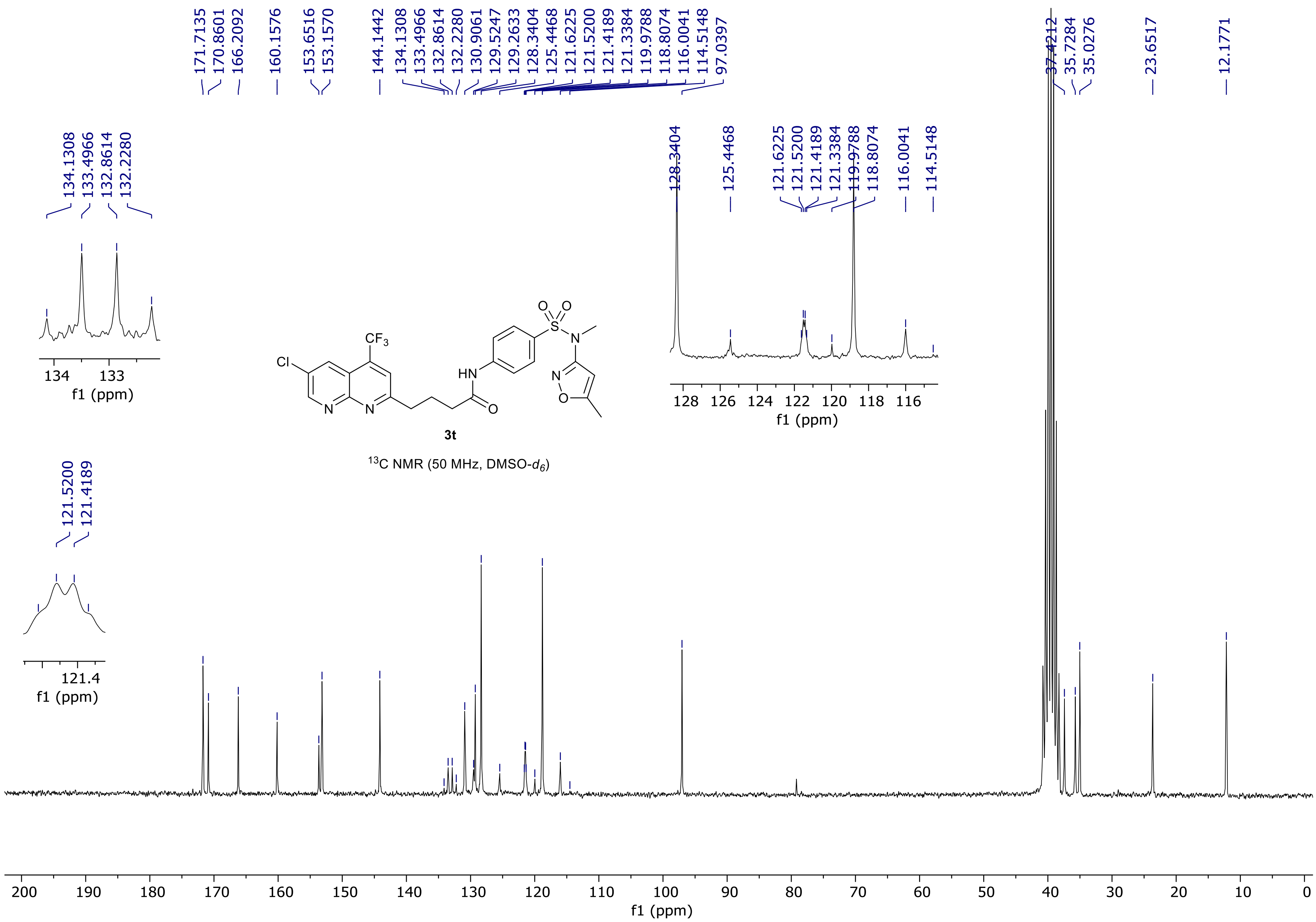

3t

^{13}C NMR (50 MHz, DMSO-d_6)

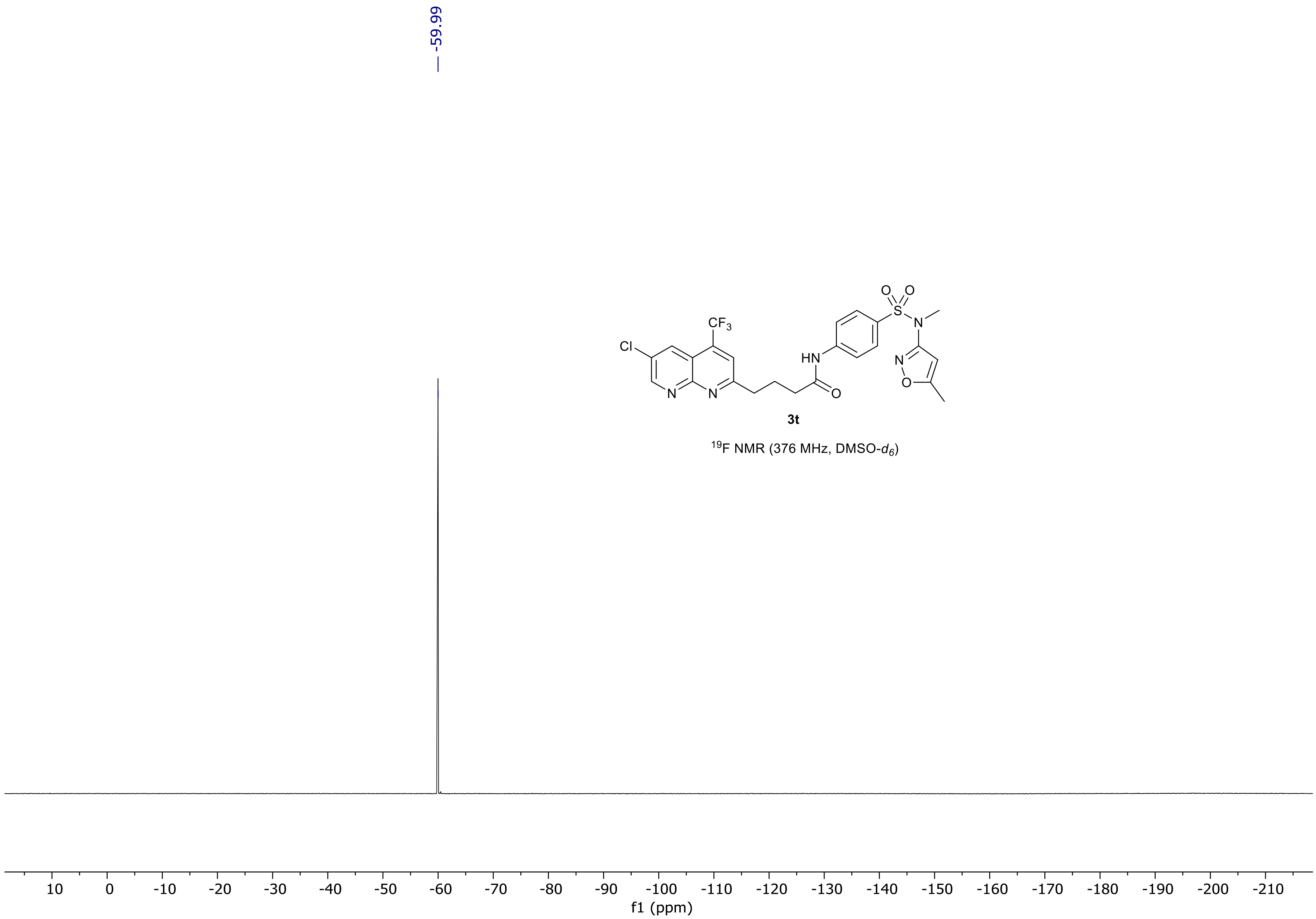
-59.99
Cl
CF3
N
N
HN
O
S
O
O
N
N
O
3t
19F NMR (376 MHz, DMSO-d6)
10 0 -10 -20 -30 -40 -50 -60 -70 -80 -90 -100 -110 -120 -130 -140 -150 -160 -170 -180 -190 -200 -210
f1 (ppm)

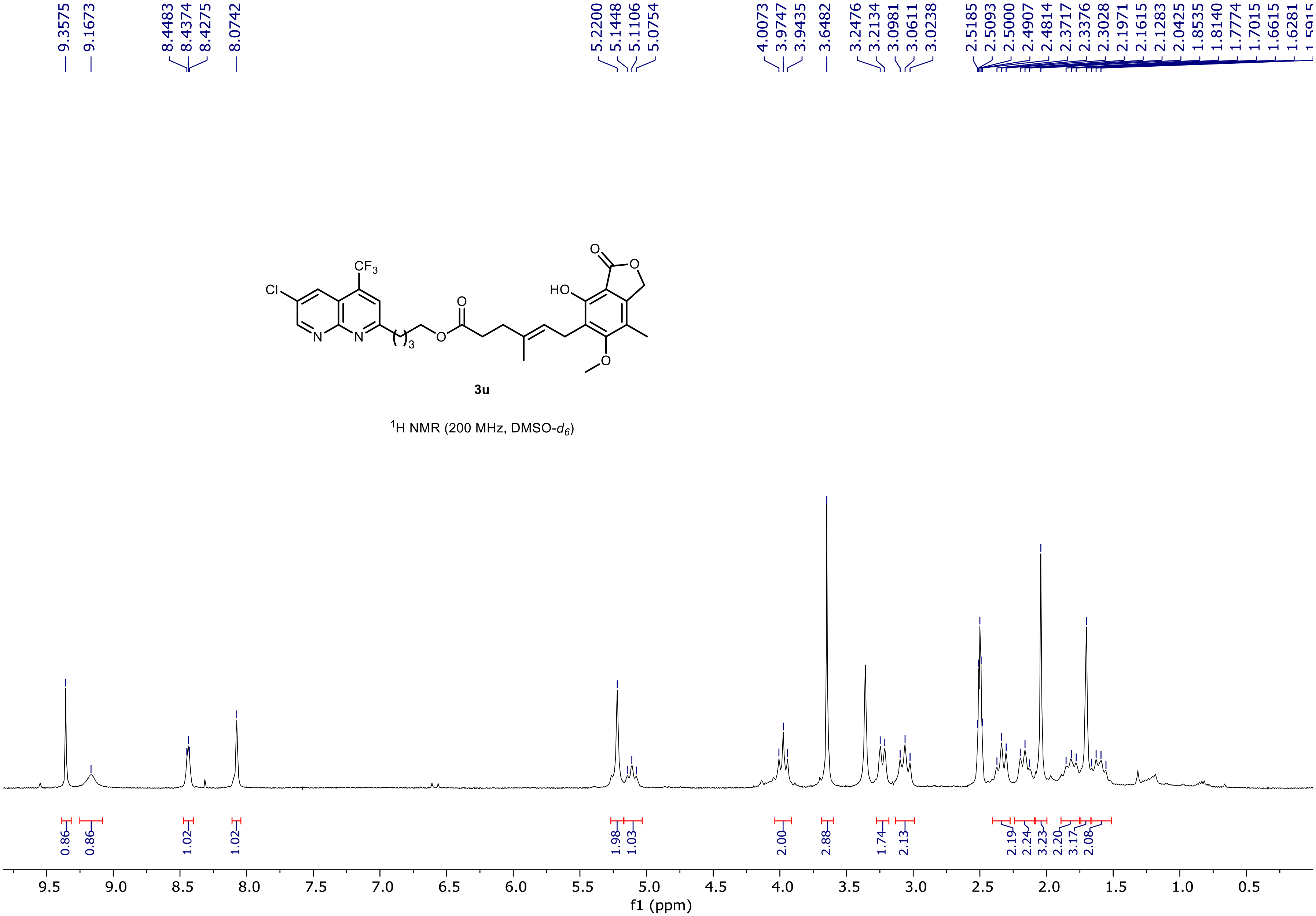

3u

^{1}H NMR (200 MHz, DMSO-d_6)

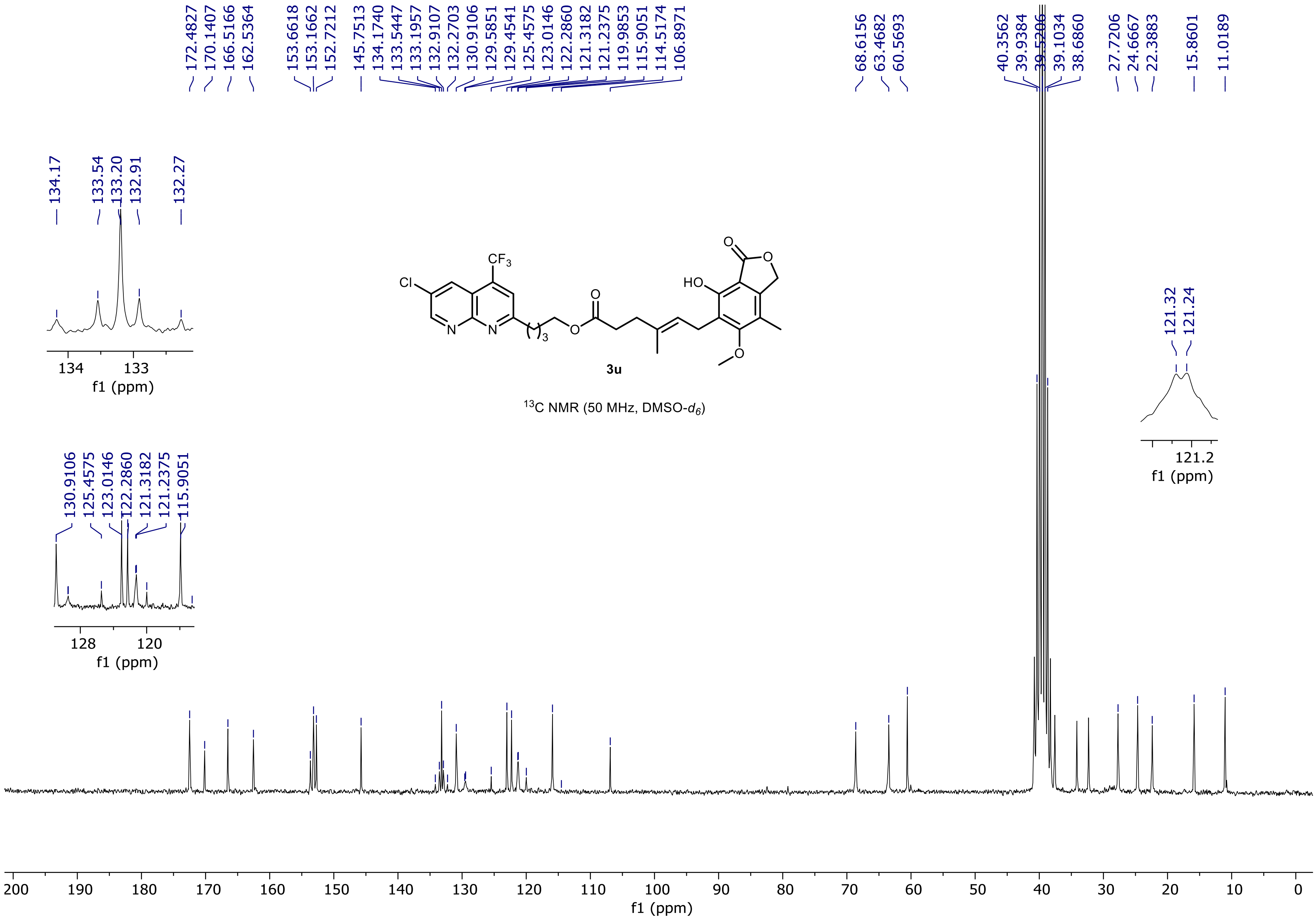
172.4827
170.1407
166.5166
162.5364
153.6618
153.1662
152.7212
145.7513
134.1740
133.5447
133.1957
132.9107
132.2703
130.9106
129.5851
129.4541
125.4575
123.0146
122.2860
121.3182
121.2375
119.9853
115.9051
114.5174
106.8971
68.6156
63.4682
60.5693
40.3562
39.9384
39.5206
39.1034
38.6860
27.7206
24.6667
22.3883
15.8601
11.0189
134.17
133.54
133.20
132.91
132.27
134
133
f1 (ppm)
130.9106
125.4575
123.0146
122.2860
121.3182
121.2375
115.9051
128
120
f1 (ppm)
CF3
Cl
HO
O
O
O
N
N
O
O
3
3u
13C NMR (50 MHz, DMSO-d6)
121.32
121.24
121.2
f1 (ppm)
200
190
180
170
160
150
140
130
120
110
100
90
80
70
60
50
40
30
20
10
0
f1 (ppm)

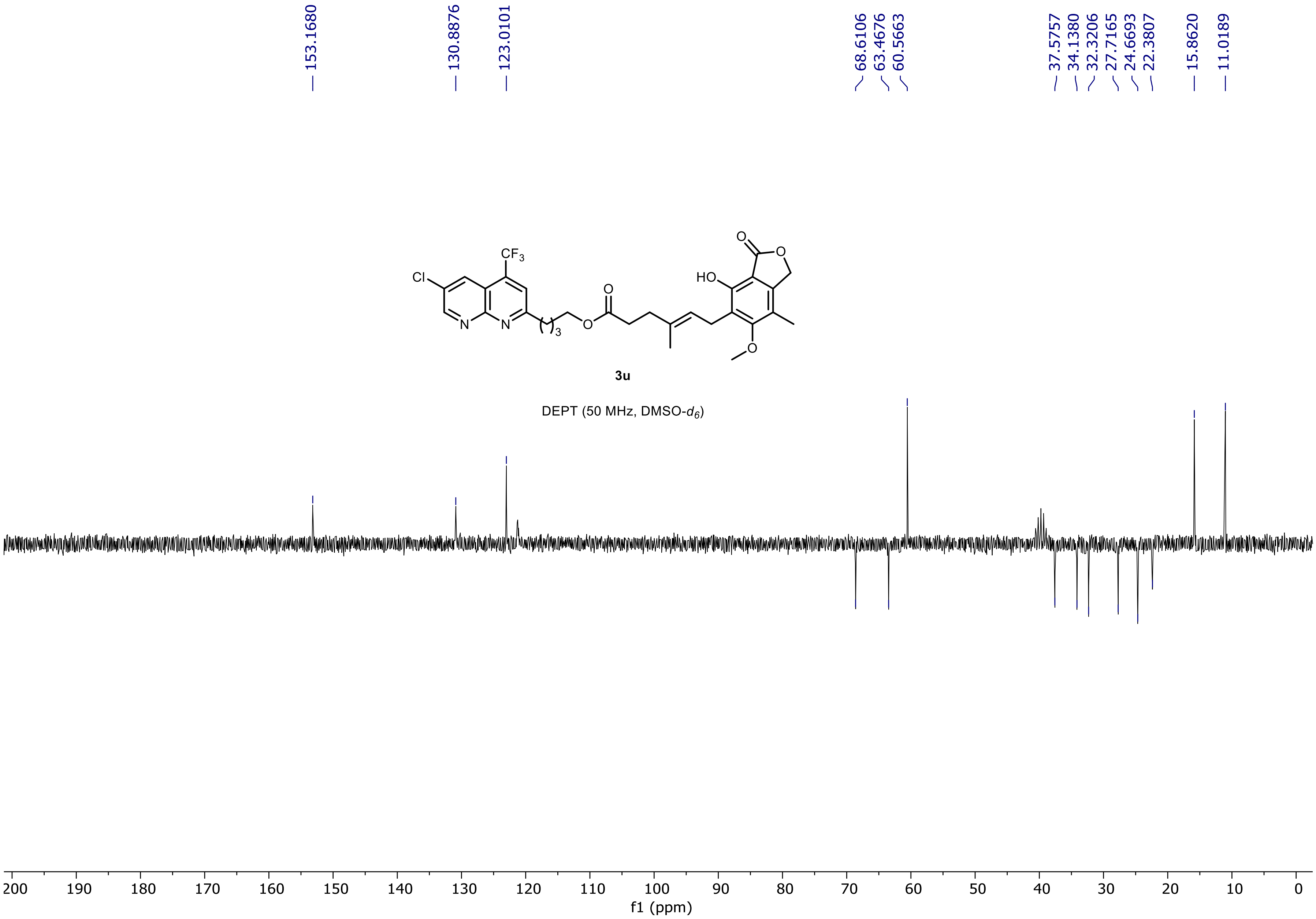
153.1680
130.8876
123.0101
68.6106
63.4676
60.5663
37.5757
34.1380
32.3206
27.7165
24.6693
22.3807
15.8620
11.0189
CF3
Cl
N
N
HO
O
O
O
O
O
3
3u
DEPT (50 MHz, DMSO-d6)
200 190 180 170 160 150 140 130 120 110 100 90 80 70 60 50 40 30 20 10 0
f1 (ppm)

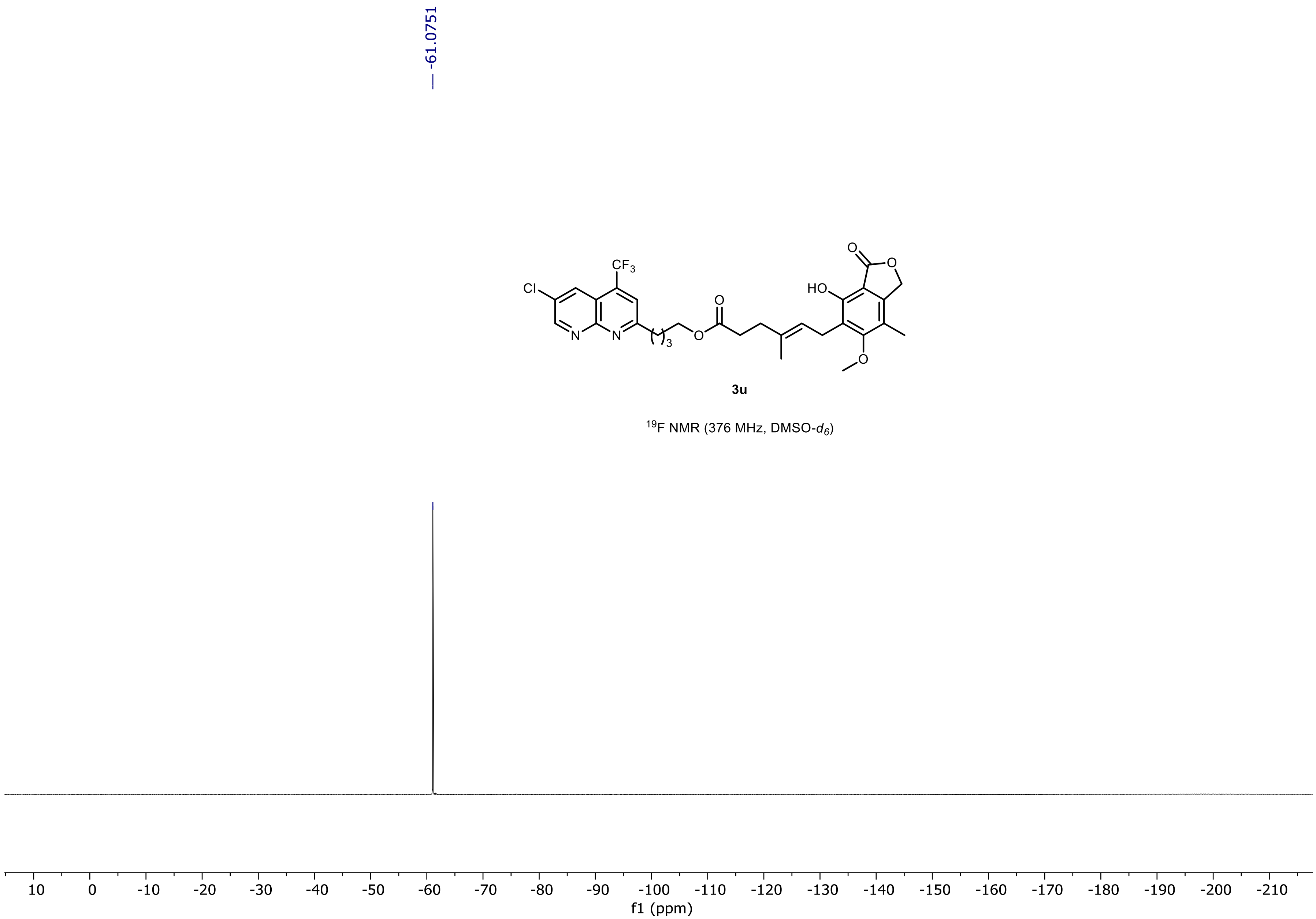

3u

^{19}F NMR (376 MHz, DMSO-d_6)

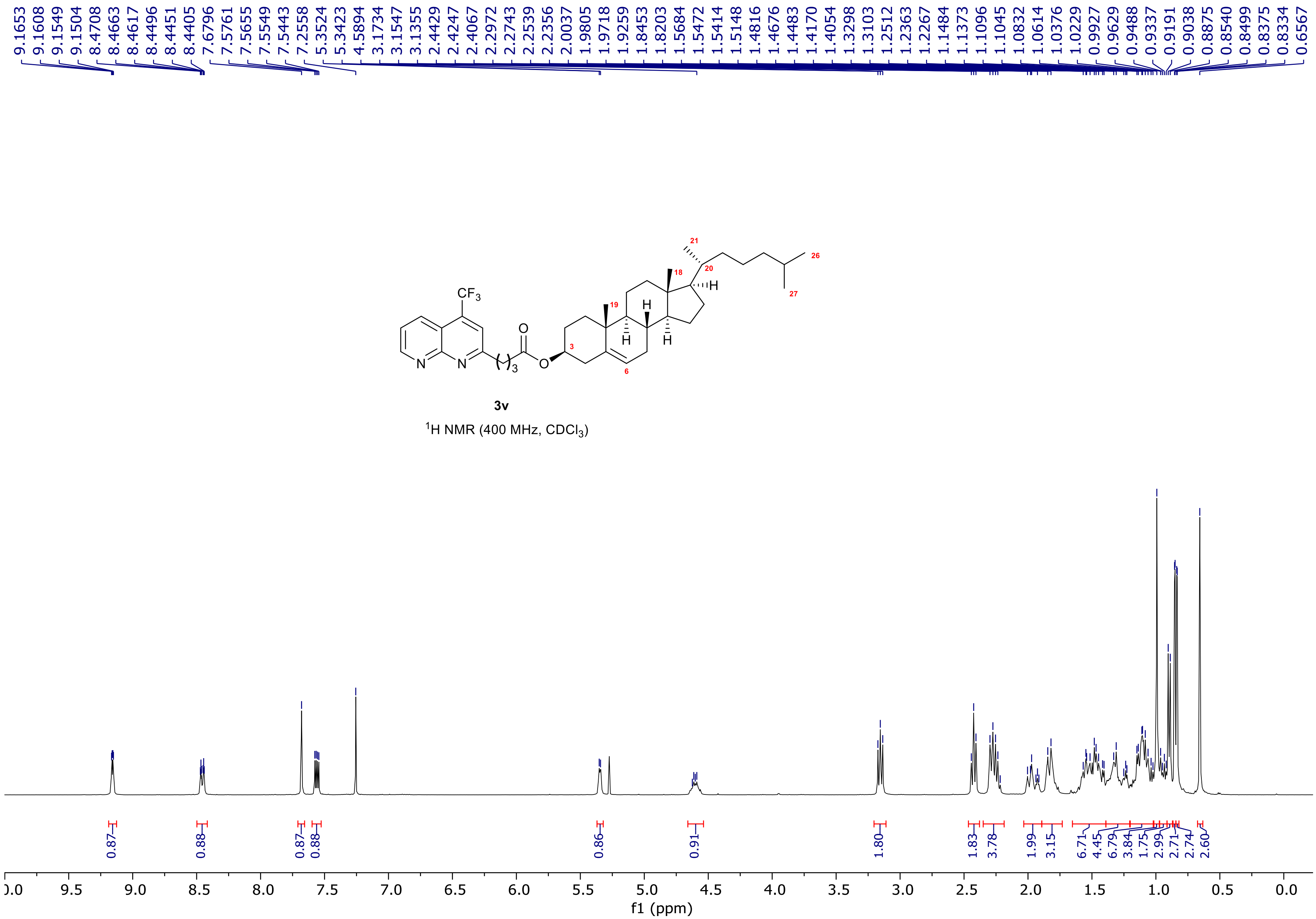

3v
^{1}H NMR (400 MHz, CDCl3)
f1 (ppm)

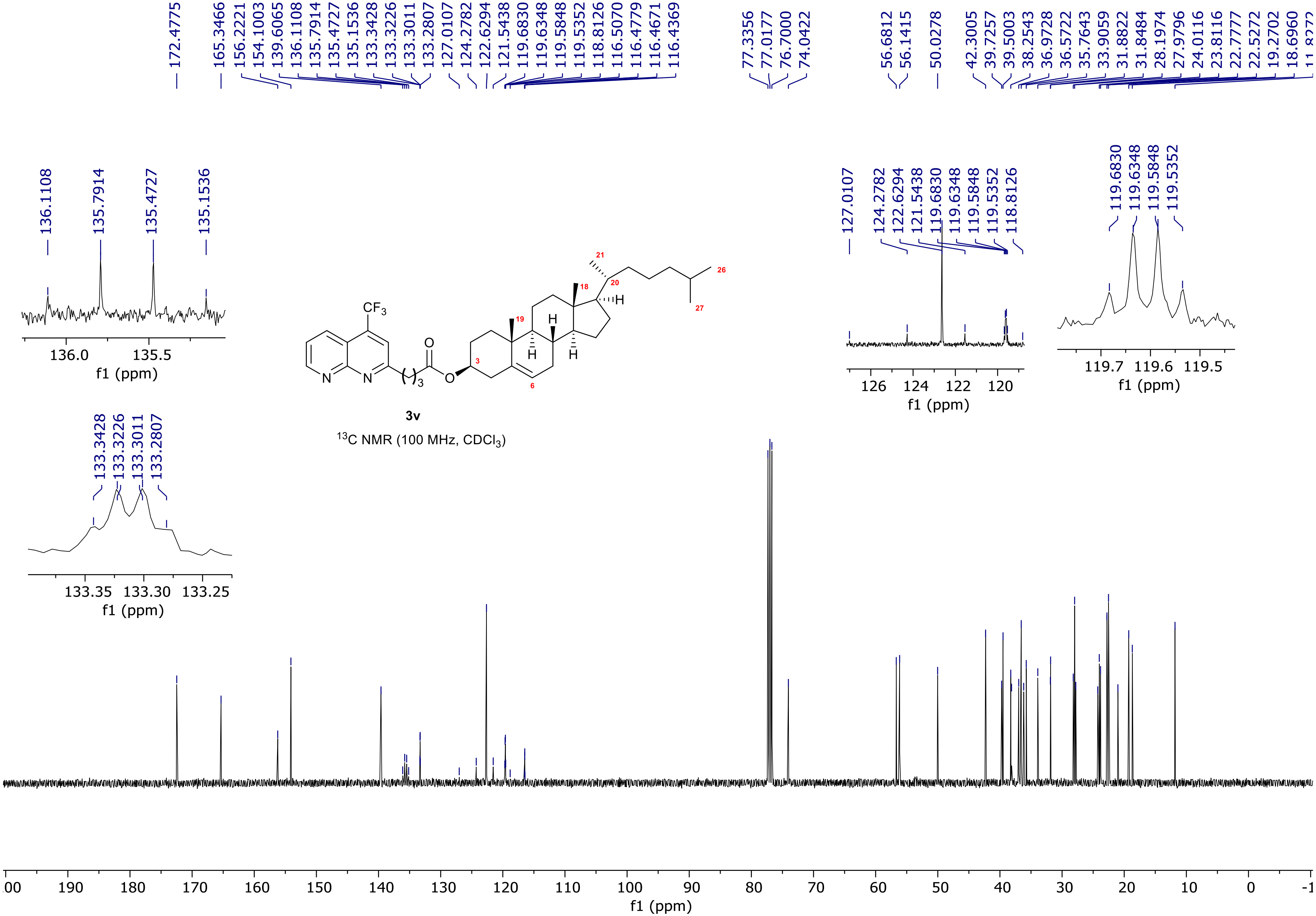

3v

^{13}C NMR (100 MHz, $CDCl_3$)

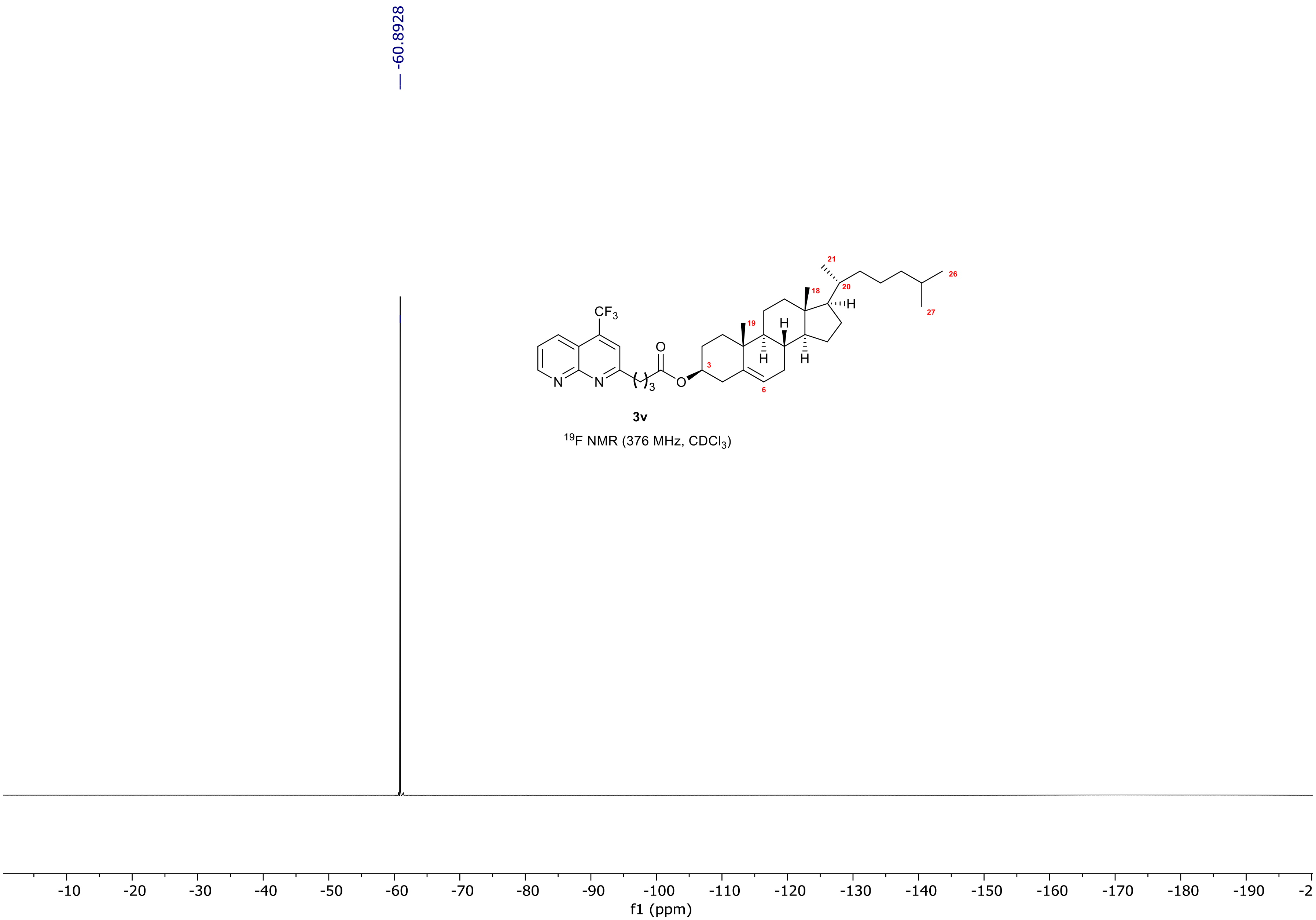

3v

^{19}F NMR (376 MHz, $CDCl_3$)

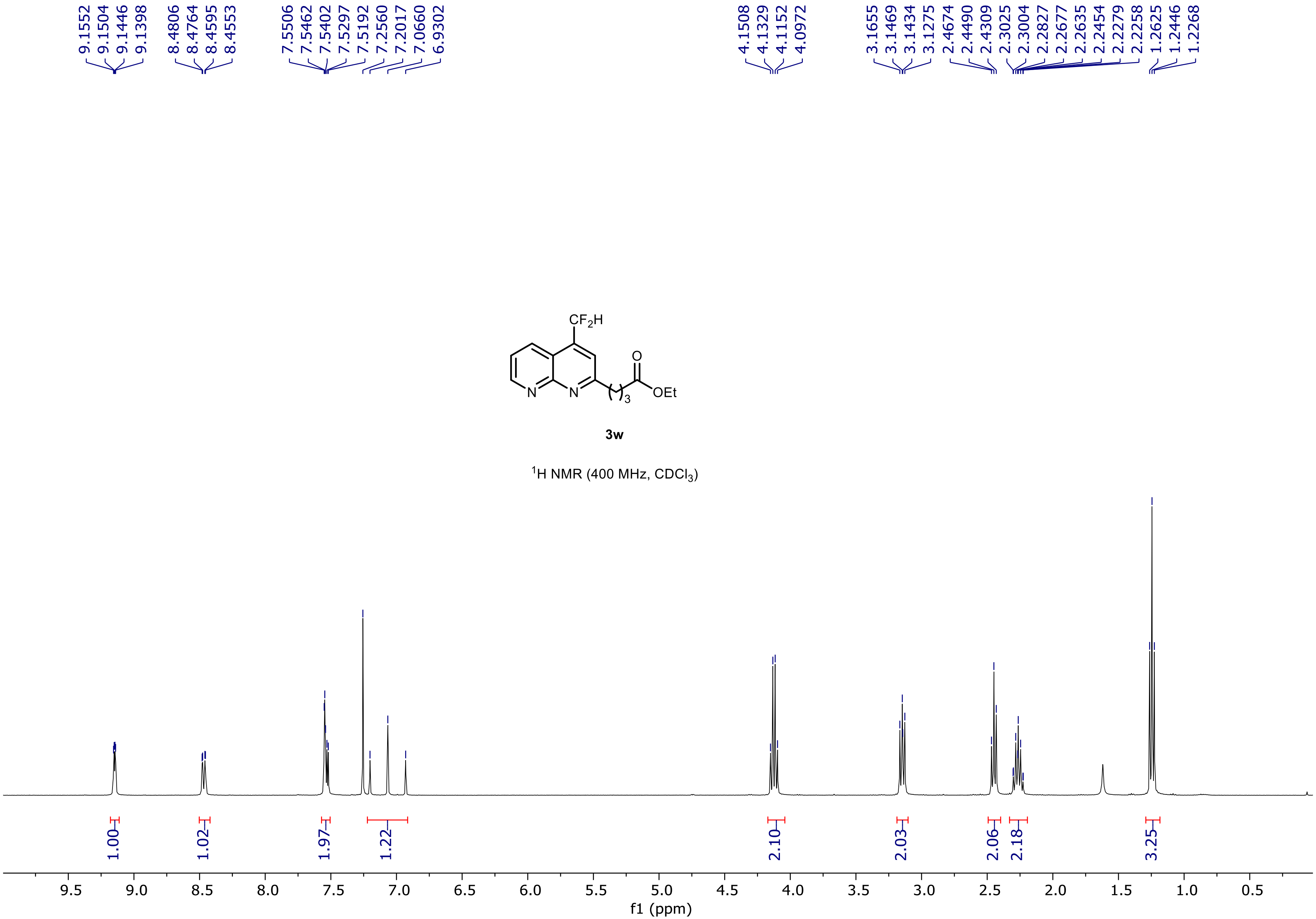

3w

^{1}H NMR (400 MHz, $CDCl_3$)

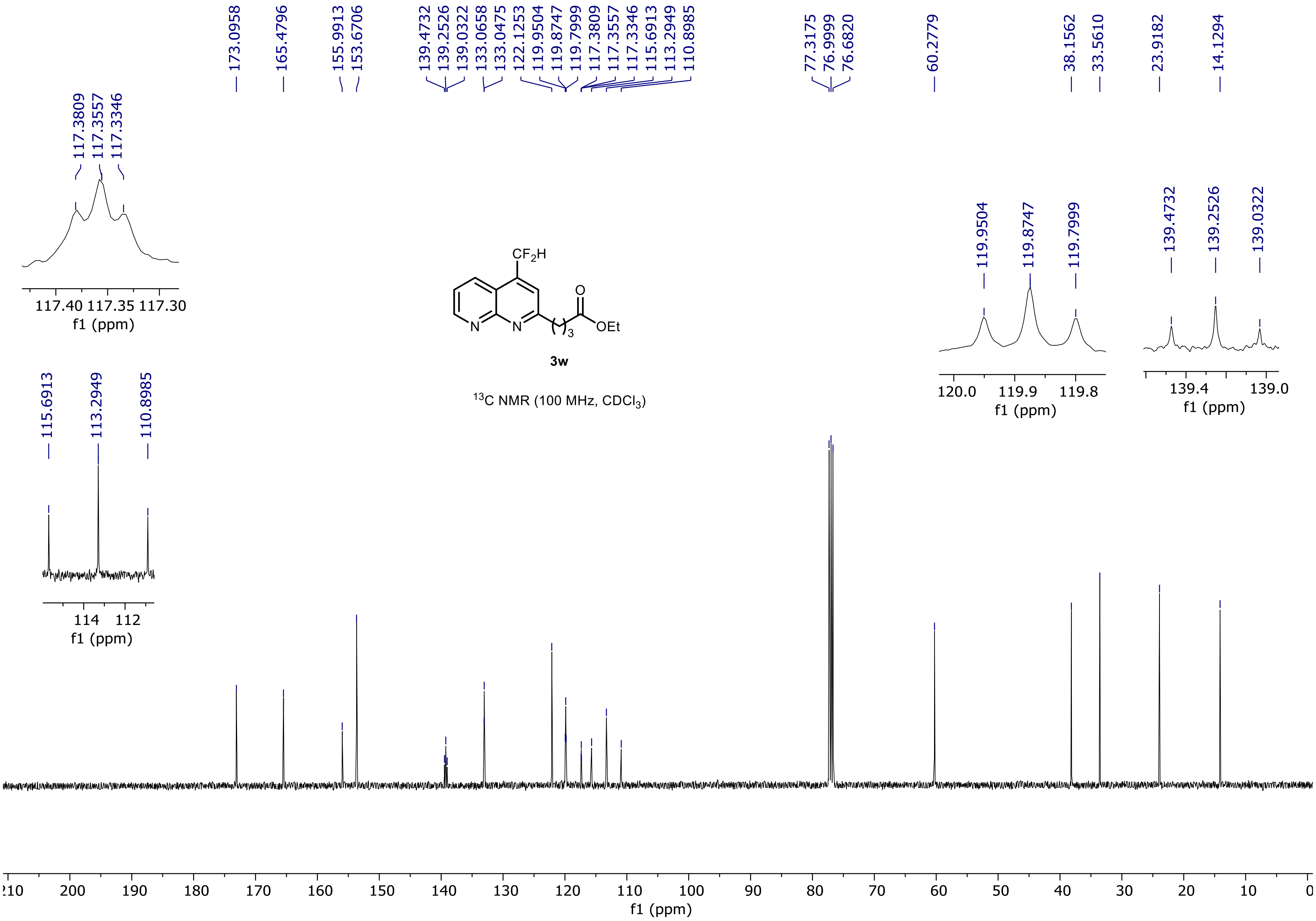

173.0958
165.4796
155.9913
153.6706
139.4732
139.2526
139.0322
133.0658
133.0475
122.1253
119.9504
119.8747
119.7999
117.3809
117.3557
117.3346
115.6913
113.2949
110.8985
77.3175
76.9999
76.6820
60.2779
38.1562
33.5610
23.9182
14.1294
CF_2H
O
N
N
OEt
3
3w
^{13}C NMR (100 MHz, $CDCl_3$)
f1 (ppm)

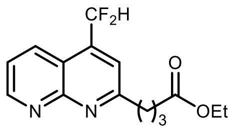

3w

^{19}F NMR (376 MHz, $CDCl_3$)

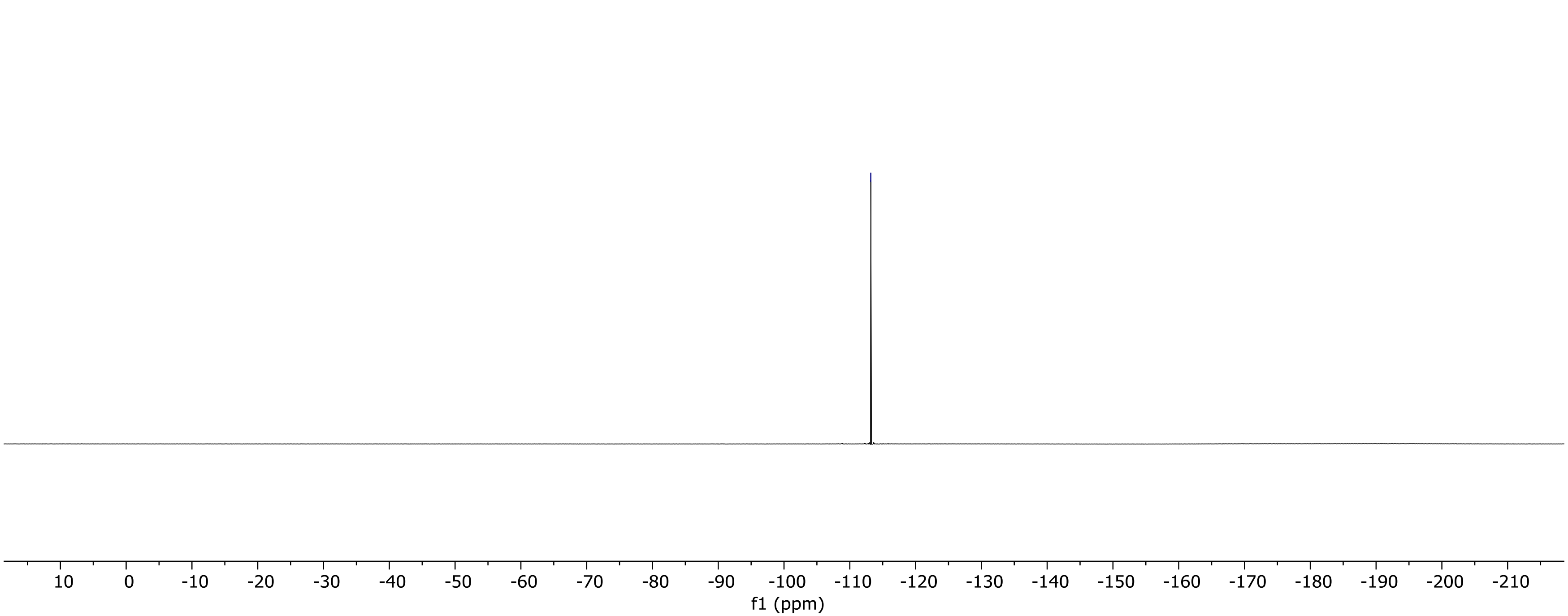

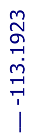

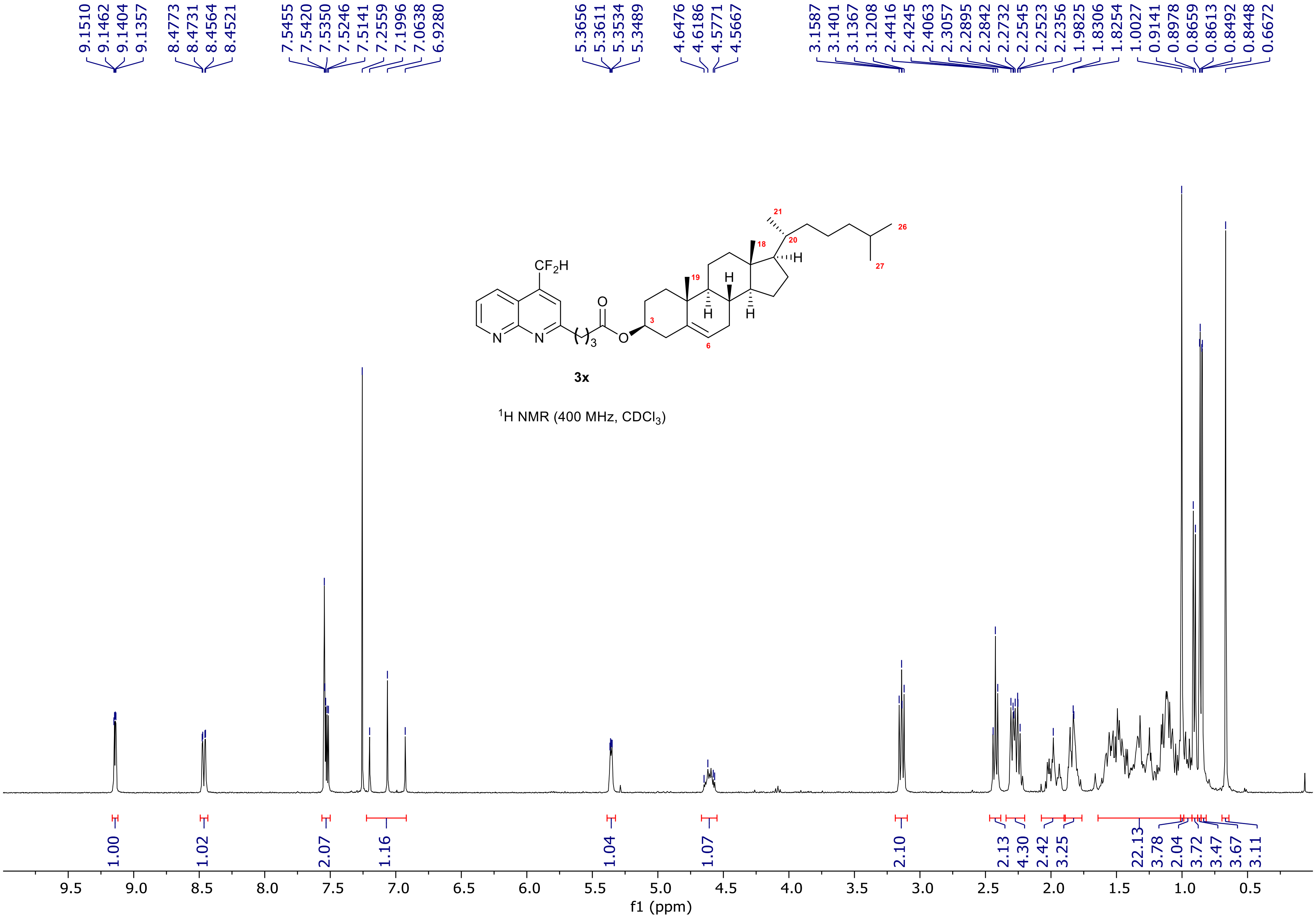
9.1510 9.1462 9.1404 9.1357
8.4773 8.4731 8.4564 8.4521
7.5455 7.5420 7.5350 7.5246 7.5141 7.2559 7.1996 7.0638 6.9280
5.3656 5.3611 5.3534 5.3489
4.6476 4.6186 4.5771 4.5667
3.1587 3.1401 3.1367 3.1208 2.4416 2.4245 2.4063 2.3057 2.2895 2.2842 2.2732 2.2545 2.2523 2.2356 1.9825 1.8306 1.8254 1.0027 0.9141 0.8978 0.8659 0.8613 0.8492 0.8448 0.6672
CF2H
O
N
N
O
3
3
6
19
18
21
20
26
27
H
3x
1H NMR (400 MHz, CDCl3)
1.00
1.02
2.07
1.16
1.04
1.07
2.10
2.13
4.30
2.42
3.25
22.13
3.78
2.04
3.72
3.47
3.67
3.11
9.5 9.0 8.5 8.0 7.5 7.0 6.5 6.0 5.5 5.0 4.5 4.0 3.5 3.0 2.5 2.0 1.5 1.0 0.5
f1 (ppm)

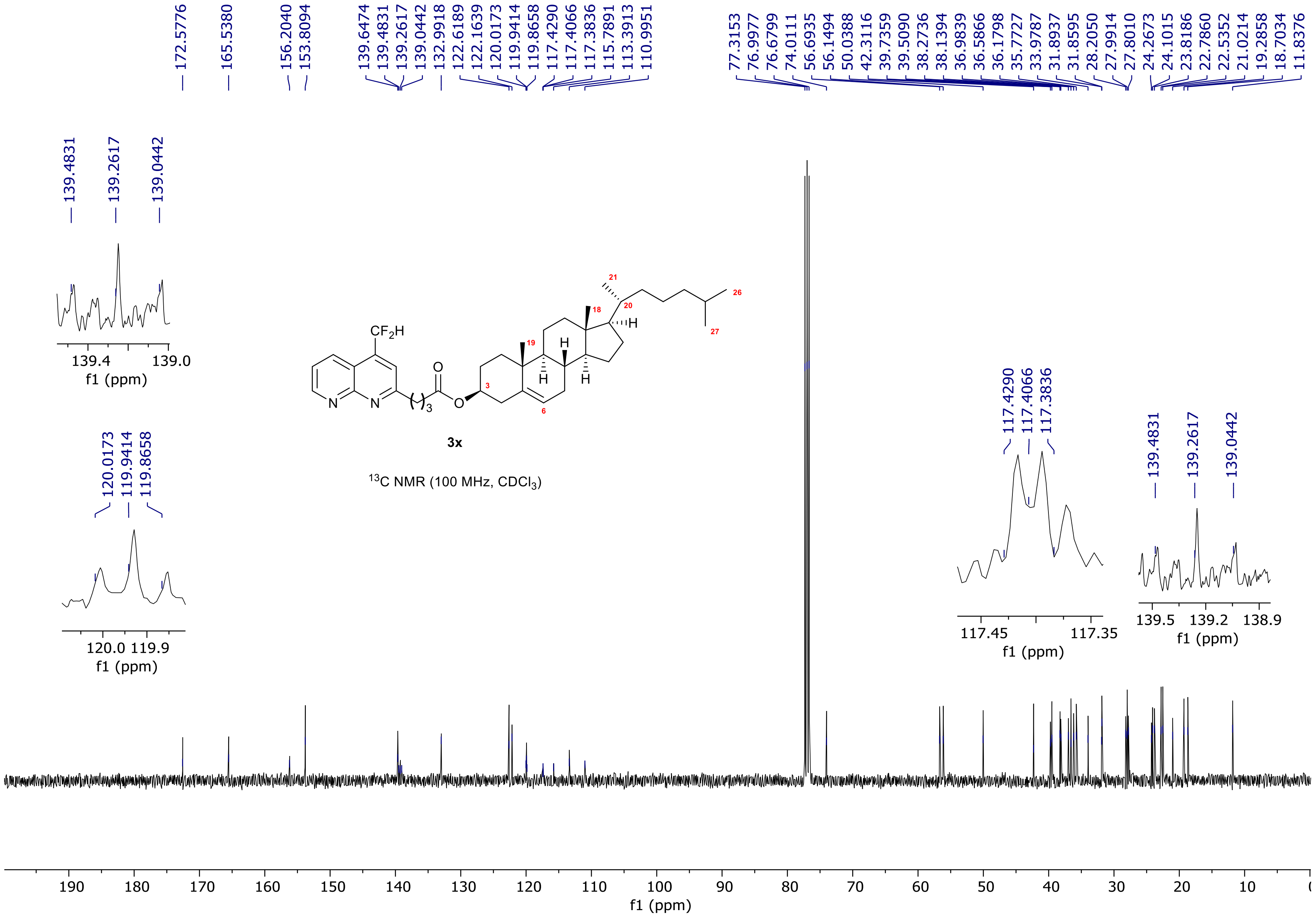

3x

^{13}C NMR (100 MHz, $CDCl_3$)

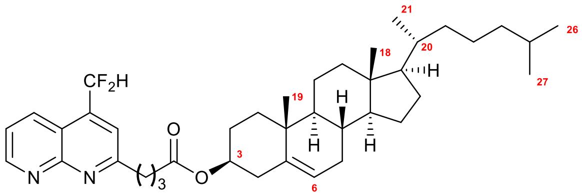

3x

^{19}F NMR (376 MHz, $CDCl_3$)

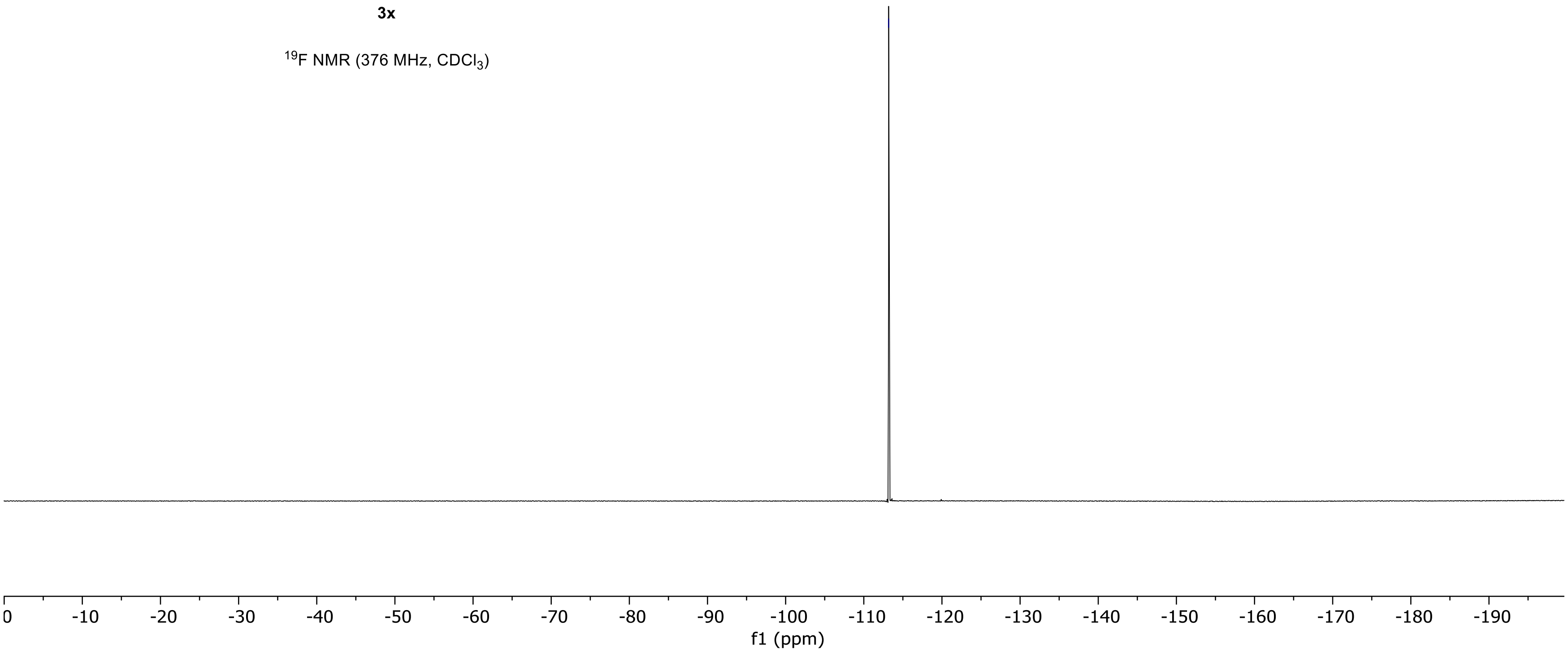

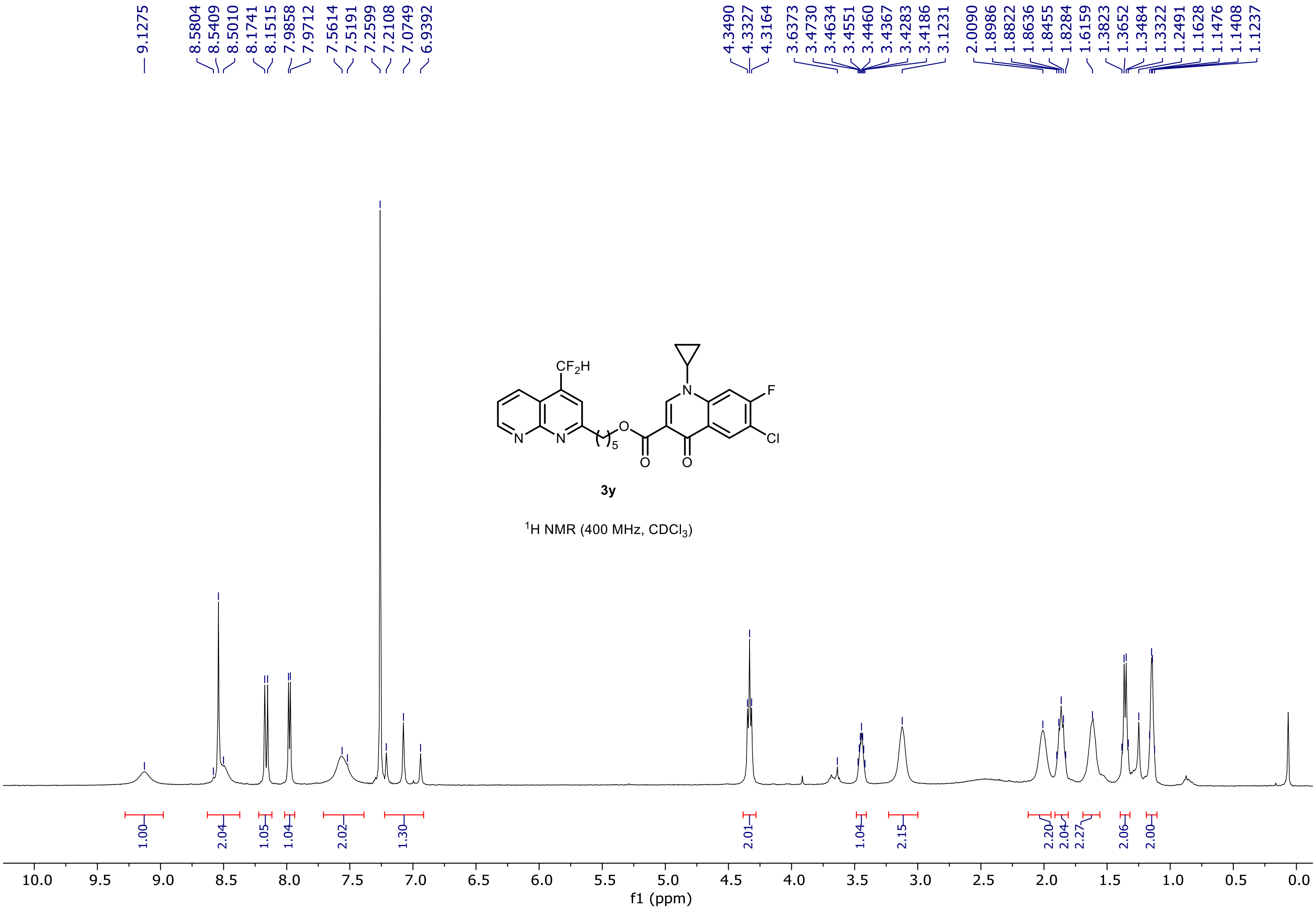
9.1275
8.5804 8.5409 8.5010 8.1741 8.1515 7.9858 7.9712 7.5614 7.5191 7.2599 7.2108 7.0749 6.9392
4.3490 4.3327 4.3164 3.6373 3.4730 3.4634 3.4551 3.4460 3.4367 3.4283 3.4186 3.1231
2.0090 1.8986 1.8822 1.8636 1.8455 1.8284 1.6159 1.3823 1.3652 1.3484 1.3322 1.2491 1.1628 1.1476 1.1408 1.1237
CF2H
N
N
O
5
O
N
F
Cl
O
3y
1H NMR (400 MHz, CDCl3)
1.00
2.04
1.05
1.04
2.02
1.30
2.01
1.04
2.15
2.20
2.04
2.27
2.06
2.00
10.0 9.5 9.0 8.5 8.0 7.5 7.0 6.5 6.0 5.5 5.0 4.5 4.0 3.5 3.0 2.5 2.0 1.5 1.0 0.5 0.0
f1 (ppm)

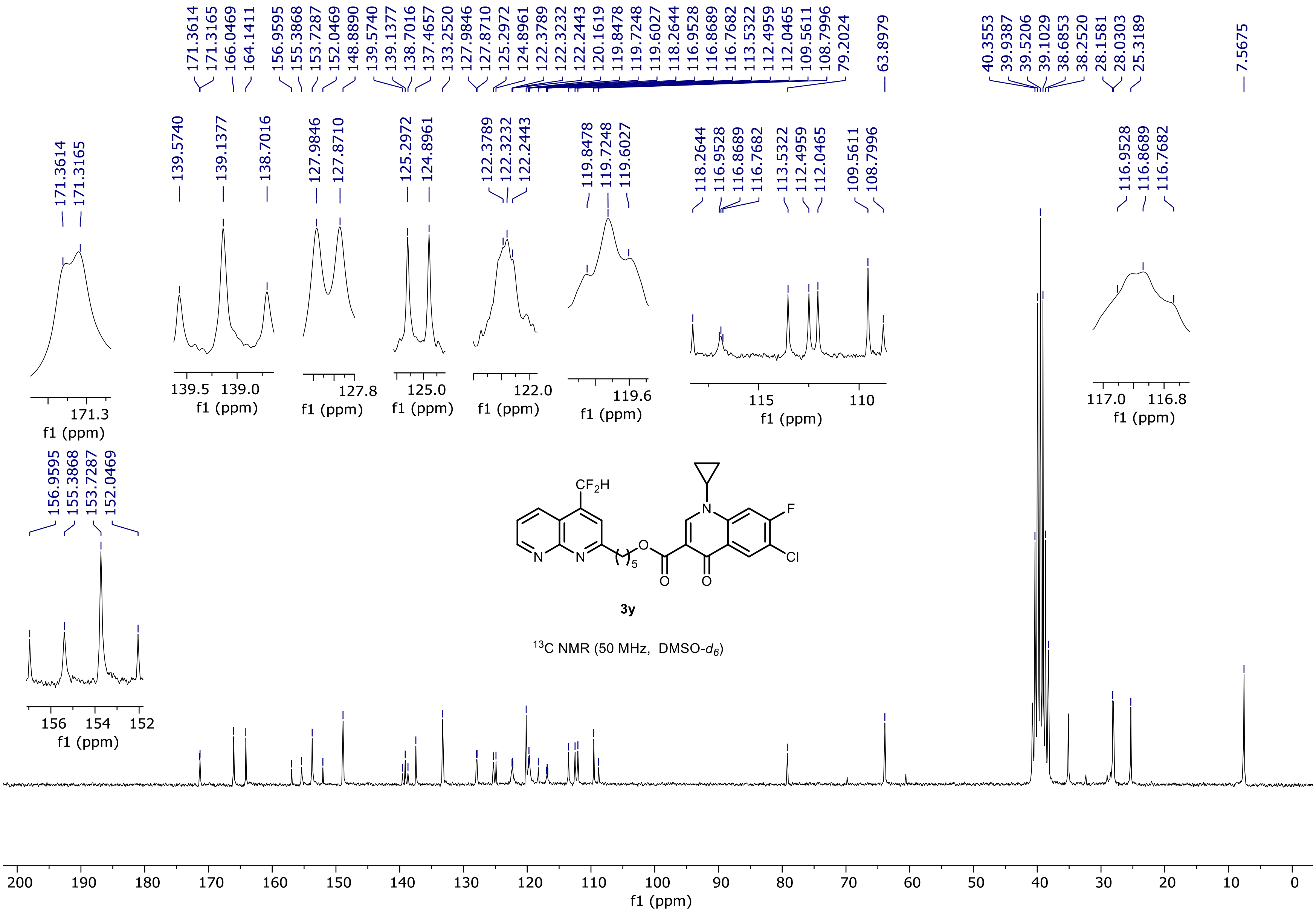
171.3614
171.3165
166.0469
164.1411
156.9595
155.3868
153.7287
152.0469
148.8890
139.5740
139.1377
138.7016
137.4657
133.2520
127.9846
127.8710
125.2972
124.8961
122.3789
122.3232
122.2443
120.1619
119.8478
119.7248
119.6027
118.2644
116.9528
116.8689
116.7682
113.5322
112.4959
112.0465
109.5611
108.7996
79.2024
63.8979
40.3553
39.9387
39.5206
39.1029
38.6853
38.2520
28.1581
28.0303
25.3189
7.5675
f1 (ppm)
CF2H
N
N
O
O
O
O
N
F
Cl
5
3y
13C NMR (50 MHz, DMSO-d6)

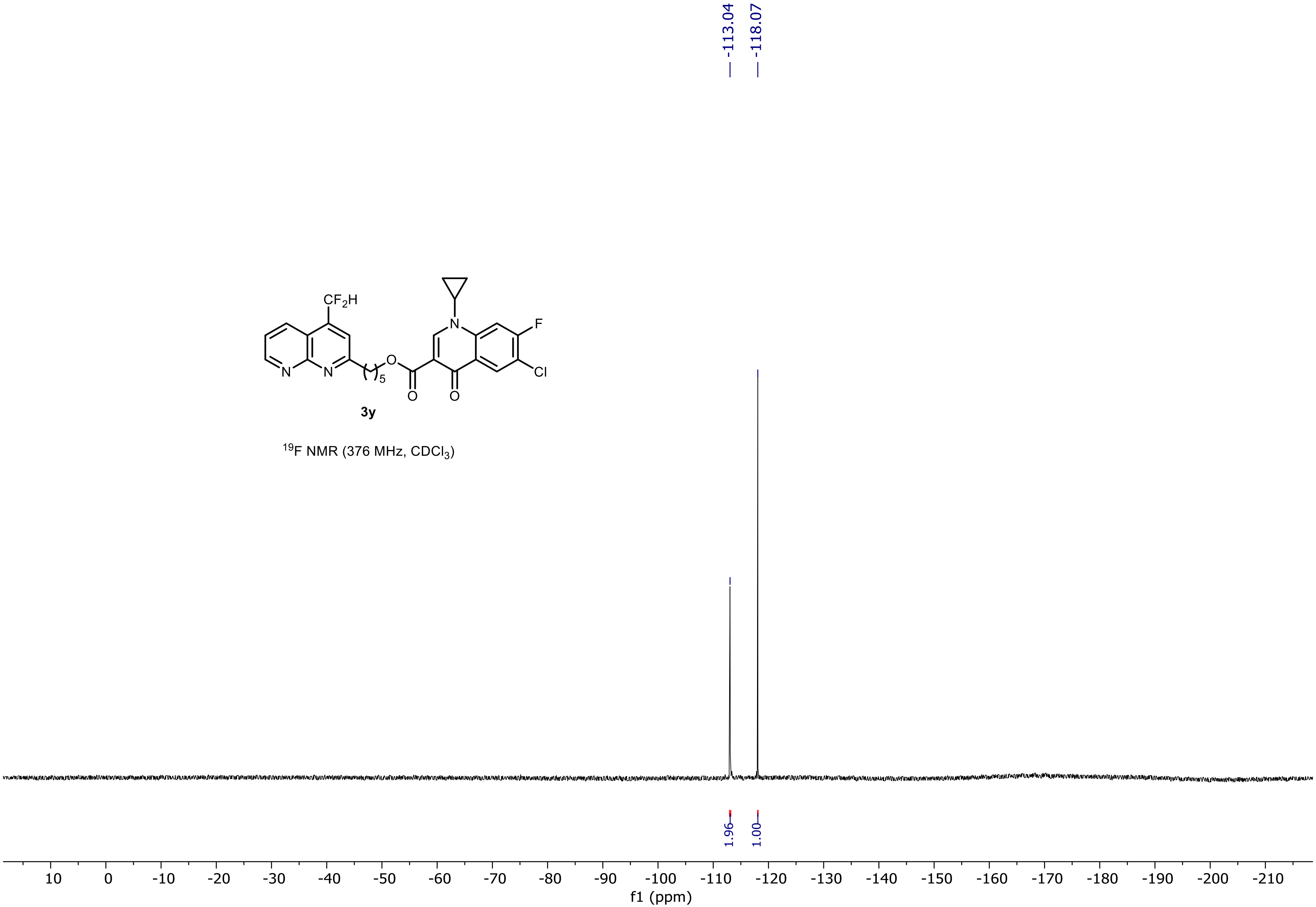

^{19}F NMR (376 MHz, $CDCl_3$)
